# Chemical Distance-Based Acceleration of Large Library Docking with ChemSTEP

**DOI:** 10.1021/acscentsci.6c00396

**Published:** 2026-07-20

**Authors:** Olivier Mailhot, Katie L. Holland, Lu Paris, Dmytro S. Radchenko, Yurii S. Moroz, John J. Irwin, Brian K. Shoichet

**Affiliations:** † Department of Pharmaceutical Chemistry, 207046 University of California, San Francisco, California 94143-2550, United States; ‡ Enamine Ltd, Kyïv 02094, Ukraine; § Chemspace LLC, Kyïv 02094, Ukraine; ∥ Department of Chemistry, Taras Shevchenko National University of Kyïv, Kyïv 01601, Ukraine

## Abstract

While make-on-demand libraries now span trillions of molecules, full library docking struggles beyond a few billion, motivating prioritization that recovers top-scoring compounds while evaluating only a fraction of a library. Here we introduce a similarity-based prioritization approach, ChemSTEP, and define the effective size of a library treated by any prioritization algorithm, N_eff_. ChemSTEP docks a representative seed set, selects diverse high-scoring “beacons”, and iteratively traverses the library through cycles of beacon selection, similarity search, and docking. Retrospectively on eight targets, ChemSTEP recovered over 75% of high-scoring compounds while docking less than 5% of a library. We then tested ChemSTEP prospectively against AmpC β-lactamase using a 13.2 billion molecule library. Because AmpC recognizes negatively charged inhibitors, we explicitly docked all 360 million library anions, synthesizing and testing 241 high-ranking ones in parallel to the ChemSTEP 13.2B run. Compared with previous docking of 99 million and 1.7 billion molecules against AmpC, the 13.2 billion library had higher hit-rates (2% vs 25% vs 37%, respectively) and found more potent compounds. Meanwhile, ChemSTEP retrieved 80% of the 241 high-ranking compounds within the first 0.5% docked. Trillion-molecule libraries might be in reach with this approach.

## Graphical abstract

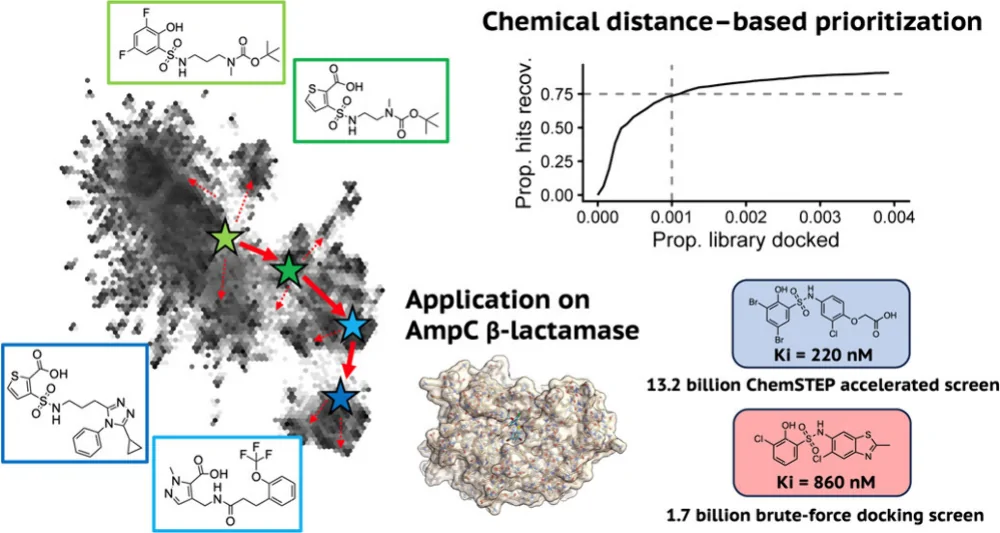

## Introduction

With the advent of ultralarge make-on-demand chemical libraries, molecular docking screens have often found sub-μM actives unrelated to previously known ligands.^1–8^ Indeed, evidence from both standalone large-library screens^2,9–13^ and side-by-side comparisons of larger and smaller libraries^14,15^ suggest that experimental hit-rates and potencies improve with library size. Accordingly, it seems attractive to scale molecular docking to tackle the ever-increasing number of readily available molecules. Today, enumerated tangible space spans close to 100 billion purchasable molecules,^16^ while on-demand but unenumerated spaces now exceed 4 trillion accessible compounds.^17^

The potential gains to be realized by exploring larger spaces have sparked innovation in prioritization algorithms: surrogate models or strategies that allow the recovery of most top-scoring compounds of a virtual library while explicitly docking a fraction of it. Since investigators have typically focused on the top-ranking 0.01% or 0.001% of molecules from a docking screen, or even less,^18^ the maximum theoretical speedup from prioritization algorithms can be large (10^4^–10^5^ or more).

Three broad categories of prioritization algorithms exist: machine learning (ML)-based,^9,19–21^ synthon-based,^12,22,23^ including evolutionary-based variations of that idea,^24,25^ and what we will call distance-based methods,^26,27^ a category that also encompasses multireference similarity searches that have been used in ligand-based virtual screening for decades.^28^ ChemSTEP, which we introduce here, falls in the latter category as it uses simple chemical similarity to guide its search through the large libraries. It differs from prior distance-based methods in three respects: it bootstraps from a docked seed set rather than requiring known actives; it iterates beacon selection and similarity-based prioritization by round; and it is here tested prospectively at the 13.2-billion molecule scale, among the largest such campaigns with experimental testing to date. Synthon-based and distance-based methods, as opposed to ML methods, are “transparent”: their choices, right or wrong, may be interrogated and readily understood at every step. ChemSTEP depends on three user-specified parameters: the score threshold for a virtual hit, the number of molecules to prioritize from the larger library each round, and the number of “beacons” to use (see below). ChemSTEP does not require training and should work with any virtual screening method that produces scores.

The prioritization problem is in principle simple: given unlimited compute and storage, one would just dock the whole library, irrespective of size, and select the top fraction for further filters and inspection before synthesis. From testing across a range of docking scores, this fraction might be somewhere between top 1 in 10k to top 1 in 100M.^15,18^ The goal of the prioritization is then to recover as many of the virtual hits as possible while docking as little as possible of the full library. Because the number of virtual hits (N_vhits_)—those molecules in the top fraction of the library—may be closely approximated based on scores from a small representative subset of the library, the prioritization curve can be drawn prospectively without the need to dock the full library (Figure 1B). The virtual hits are fully determined by the docking model, and the specific prioritization algorithm used to recover them does not change their identity. From this, it follows that once a high recovery is achieved, even different prioritization algorithms will largely agree such that the specific choice of prioritization algorithm becomes mostly a matter of compute budget, ease of setup, and personal preference.

**Figure 1:**
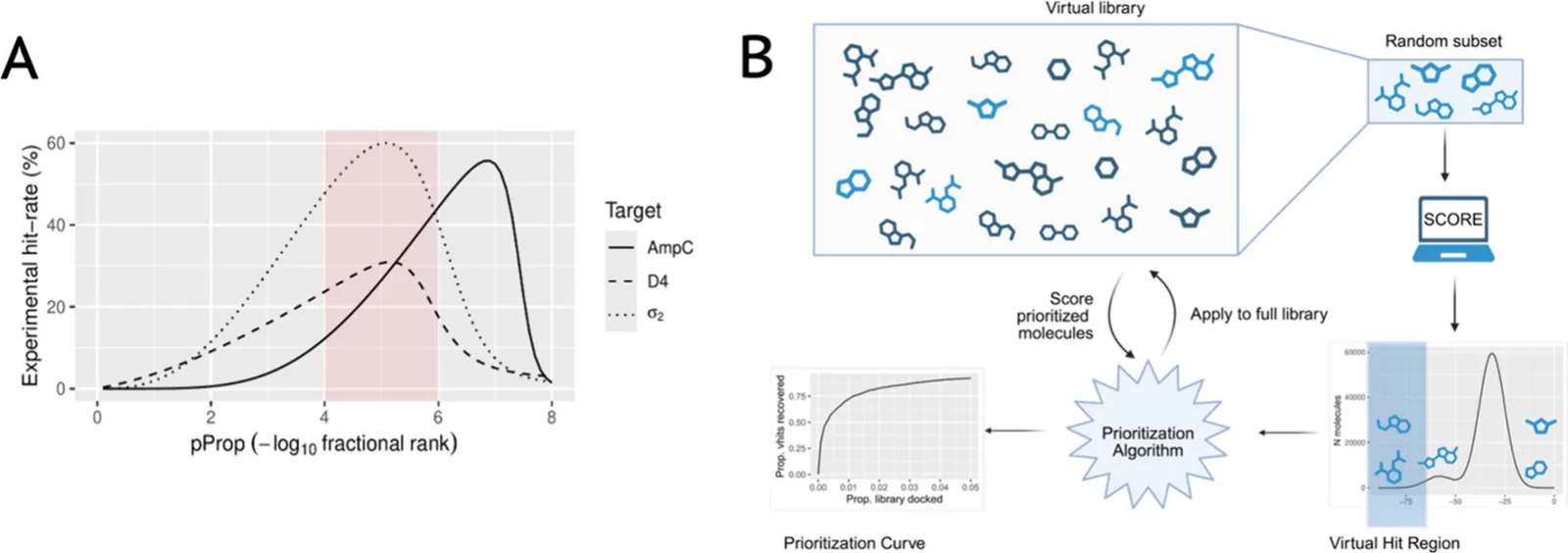
**Prioritization algorithms. A)** Fitted empirical hit-rate curves vs pProp across prior ultralarge campaigns (dopamine D4 receptor, Sigma2 receptor and AmpC β-lactamase). **B)** Establishing an empirical background from a uniform random subset enables mapping docking scores to pProp without docking the full library.

To compare different docking screens, we have found pProp rescaling^15^ useful, where a molecule is represented by the negative base-10 logarithm of its fractional rank in the docking screen (pProp = -log10­(fractional rank)), akin to the more familiar p*K*_a_ or pIC_50_. For instance, a top 1% compound has a pProp of 2, top 0.1% corresponds to a pProp of 3, and so on. Figure 1A gives the experimental hit-rates across pProp values for previous docking campaigns of 138 million molecules against the dopamine D4 receptor,^29^ 465 million against the Sigma2 receptor^1^ and 1.7 billion molecules against AmpC β-lactamase.^15^ In these campaigns, a pProp threshold of around 5 maximizes hit-rate while avoiding docking artifacts that, in our experience, start to appear beyond pProp = 6. In a prospective scenario, one can first establish the pProp values of different docking scores by docking a uniformly distributed random subset of the library, obtaining an empirical distribution (Figure 1B). Once a pProp threshold has been selected, it fully determines the number of virtual hits in the library

$$N_{vhits}=N_{library}\times 10^{-pProp}$$ 1

where N_vhits_ is the number of interesting compounds in the library and Nlibrary is the library size.

ChemSTEP biases docking toward library molecules similar to ones that score well in the previous cycle of docking. The method employs widely used metrics of ligand similarity so that at each stage of the large-library screen a chemist or biophysicist may ask why some molecules were prioritized over others and how they performed. The method begins with a relatively small seed set that is explicitly docked. Importantly, the seed set size is independent of the library size, depending instead on the virtual hit pProp threshold selected; even for tera-scale libraries, a seed set of tens of millions can be sufficient (Methods). From the top-ranking seed molecules diverse “beacons” are chosen and the X yet-undocked molecules most similar to the beacons are selected from the much larger library, where X is user-defined and specifies how many molecules will be docked per round. This number is selected to be large enough that most molecules selected are relatively distant from the previous beacons. These loosely beacon-similar molecules are built and docked, and a new set of maximally divergent beacons is selected. The cycle continues until a user-defined stopping criterion is reached, typically a target virtual hit recovery, a fixed compute budget, or a maximum number of rounds (Methods).

We first test ChemSTEP retrospectively against eight large library, explicit docking screens from the LSD benchmarking set (lsd.docking.org)^30^ from which multiple diverse experimental actives have been found, asking how well the method recapitulates the high-ranking and experimentally potent molecules. We then test ChemSTEP in a new prospective docking screen versus the model enzyme AmpC β-lactamase, a target of multiple high-throughput screening^31,32^ and docking^15,29,33^ campaigns with ever larger libraries. In parallel with ChemSTEP prioritization of a 13.2 billion library, we explicitly docked 360 million anions drawn from the same library—since AmpC is both experimentally and by docking heavily biased toward anion and dianion recognition, the 360 million anion subset should contain the great majority of possible inhibitors within the larger library. From the top-ranking molecules of the full anion screen (which also contain all ChemSTEP virtual hits), 241 are de novo synthesized and tested experimentally for enzyme inhibition, establishing what the “ground truth” docking hit rate and affinities would be for the 13.2 billion molecule library. Compound selection is performed after completion of the brute-force docking to ensure a uniformly sampled hit set not biased by ChemSTEP prioritization. We then perform 99 additional replicate cycles of ChemSTEP against the 13.2 billion library (total of 100 runs) and ask how well it retrieves the virtual hits, especially those of the 241 tested that were AmpC inhibitors.

**Figure 2:**
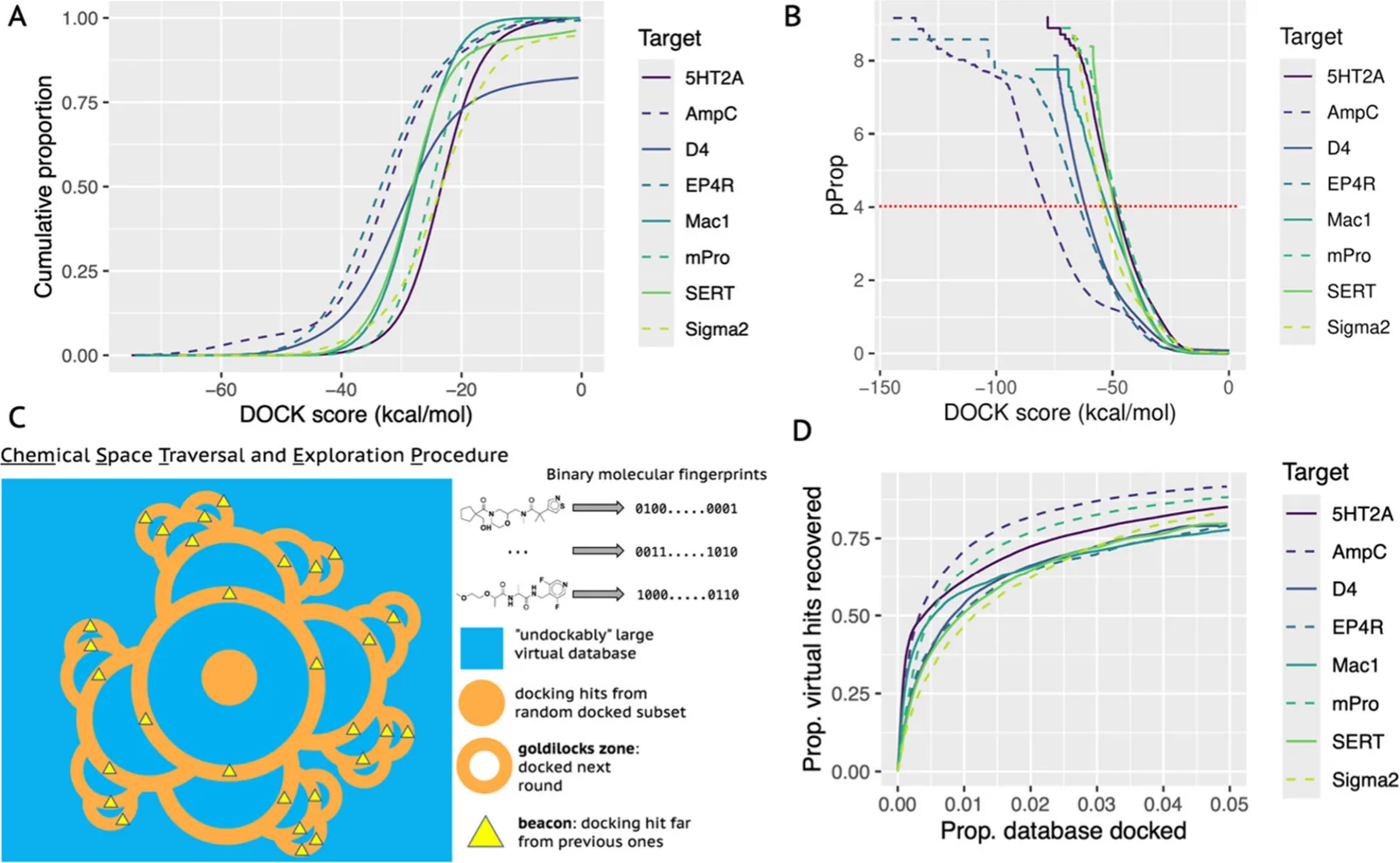
**The ChemSTEP algorithm and its application to eight previous ultralarge docking campaigns. A)** The docking score cumulative distributions for the eight targets from the LSD Web site^30^ with >50 million molecules docked. **B)** The pProp transformation of the score distribution, with the pProp = 4 virtual hit definition used here identified by a red dotted line. **C)** Schematic representation of ChemSTEP. **D)** Prioritization curves for ChemSTEP applied to eight LSD Web site targets.

This parallel design allows a controlled comparison of ChemSTEP versus exhaustive docking, while also enabling us to examine whether the trend of higher docking hit-rates and potencies—seen as the library increases from hundreds of thousands to 99 million to 1.7 billion—continues as we expand to 13.2 billion. In doing so, we compare the performance of a ChemSTEP screen of a larger library with brute-force docking of smaller ones, asking if it recovers more and better actives than full docking of the smaller libraries (by nature, the prioritization algorithm must be worse than brute-force docking of exactly the same library, though it may allow one to dock many-fold fewer molecules). While we have by now much experience with AmpC, arguably improving hit rate in any given docking screen, by comparing the ChemSTEP campaign to a recent brute-force campaign on a smaller library, also conducted by us using the same docking program, we control for expertise bias. By investigating which molecules are prioritized at different stages of the traversal and the computational cost of doing so, we assess not only how successful the method is but why it works and whether it might scale to trillion-molecule libraries.

## Results

### ChemSTEP Logic Flow

ChemSTEP adopts a core assumption of medicinal chemistry, Maggiora’s “similar property principle”,^34^ which posits that similar molecules will behave similarly. For library sampling this implies that if one begins with docked scores of a representative sample of molecules that will ultimately be among the top-scoring, one can bootstrap from them through the library, using similarity, to find most of the high-ranking molecules that a full docking screen would have found. To ensure the initial seed set’s score distribution well-approximates the full library distribution around such high-ranking molecules, one can begin with a hypothesis of the fraction of the library where the best performing molecules will be found and increase that by 100-fold. For instance, if one expects the highest experimental hit rate and the most potent ligands to be ranked among the top 10^–5^ docking scores of the library (pProp = 5), then one would begin with a seed set equal to 10^7^ molecules (100 × 10^pProp^). Docking these 10^7^ molecules identifies, by construction, 100 that score at the seed-set-derived pProp = 5 threshold; the remaining question is how closely that seed threshold approximates the full-library threshold, which is the motivation for the 100 × 10^pProp^ seed size as it gives an RMSE of ∼ 0.05 pProp (Figure S2). From the top-ranking molecules, deemed virtual hits because they score better than the threshold, a diverse set of beacons is selected, with each new beacon picked maximally distant from all those previously chosen. Depending on the number of beacons used each round (typically 100), this first round might select all identified virtual hits as beacons, but as more virtual hits are found in subsequent rounds only a fraction are used as beacons, always picking the maximally distant molecule that scores better than the virtual hit threshold. From these beacons, ChemSTEP screens the whole library by Tanimoto distance, prioritizing a fixed number of similar compounds for each successive round of docking (Figure 2C, see Methods). Here we use the popular 1024-bit folded Morgan fingerprints with radius 2 (equivalent to ECFP4) fingerprint,^35^ though many others, or indeed other representations and even scoring criteria, may be used. An advantage of ECFP4 fingerprints is that they may be encoded in bit-strings that may be rapidly compared (conservatively 2.5 million pairwise similarity calculations per CPU per second, enabling scaling to the trillion-molecule space, see Methods). The three-dimensional structures of the beacon-similar molecules from the full library are calculated and then docked into the target. From the new top-ranking list a new set of maximally distant beacons is selected and the cycle of similarity searching, structure-calculation, docking, beacon selection and similarity search is iterated, with a prioritization curve calculated each round to determine the extent of the library screened based on the number of high-scoring molecules found (below).

### Retrospective Controls

We first tested ChemSTEP retrospectively on eight large library docking screens annotated in the large scale docking database,^30^ setting pProp = 4 as the virtual hit threshold. From simulations conducted on empirical docking score distributions, a seed set size of 100 × 10^pProp^ leads to high confidence in the virtual hit definition (Figure S2). With pProp = 4 a seed set is thus 1 million molecules. For each target, we selected 10 uniformly distributed random seed sets by setting a probability of 10^6^/N of picking each molecule, where N is the library size. This resulted in 80 seed sets in a tight range from 997,072 to 1,002,422 molecules. For each of the eight targets, the full list of scores is available such that “docking” a molecule for this purpose amounts to simply looking up its score. We performed 10 replicate ChemSTEP runs for each target, starting with a different seed set each time, with up to 100 beacons per round. We targeted 0.04% of the library docked per round until 5% of the library had been docked (125 maximum total rounds). The libraries ranged in size from 56 million to 1.56 billion. On the 1.56 billion library, each round of ChemSTEP took on average just over 20 min on a 64-CPU node, while the 56 million library took 3.5 min on the same 64-CPUs (the timing is dominated by similarity look-up, since no new docking nor compound building occurred). Because 100 beacons were used per round, this translates to 2.0 million 1024-bit Tc calculations/CPU/second for the larger library and 417,000 Tc calculations/CPU/second for the smaller library, including all bookkeeping and file reading happening within each round. For all eight targets, 50% or more of the virtual hits are recovered by the time 1% of the library is docked, and a 75% to 92% recovery is achieved by the time 5% of the library is docked (Figure 2D).

### Beacon Selection and Chemical Space Traversal

To understand ChemSTEP prioritization, we investigated beacon selection for the Sigma2 receptor in detail. We began by visualizing the chemical space occupied by virtual hits in relation to the library using Uniform Manifold Approximation and Projection (UMAP)^36^ on all 47,131 Sigma2 virtual hits (vhits; docked molecules ranking better than pProp = 4 threshold) plus an equally sized set of random library compounds (Methods). The main density of library molecules is strikingly devoid of virtual hits, which instead are found in sparsely populated library regions with no single region dominating (Figure 3A). Some ChemSTEP rounds, such as R3, identify vhits in particular regions of the UMAP while others (R20 for instance) find vhits scattered across the whole space (Figure 3B). To investigate prioritization at a molecular level, we started from each seed beacon used for round 1 (selected from the explicitly docked 1 M seed set) and identified its “children” beacons—beacons selected based on proximity to that specific R1 beacon. We then computed the longest streak of descendant beacons from each seed beacon (Methods). Among the 100 R1 beacons, one of the longest descendant chains began from seed beacon compound ‘6660 (Figure 3C). Its immediate descendant beacon ‘1184 is selected in R4 but was docked in R1. Its Tanimoto distance (Td) to its R1 parent, ‘6660, is 0.47. The next beacon, ‘5253, docked in R4 and selected in R5, is 0.47 Td from its immediate parent, and 0.48 Td from the ancestral beacon ‘6660 (barely any further). After more generations, however, the distance to ‘6660 grows, peaking at 0.86 for the beacons selected in R37 and R60 and staying above 0.65 (equivalent to Tc ≤ 0.35) from R28 onward. This is despite the immediate distances between any beacon and its immediate ancestor never going above 0.52. Hence, the chained selection of beacons diverges in chemical space, identifying connected paths leading from one virtual hit to a chemically distant one.

**Figure 3:**
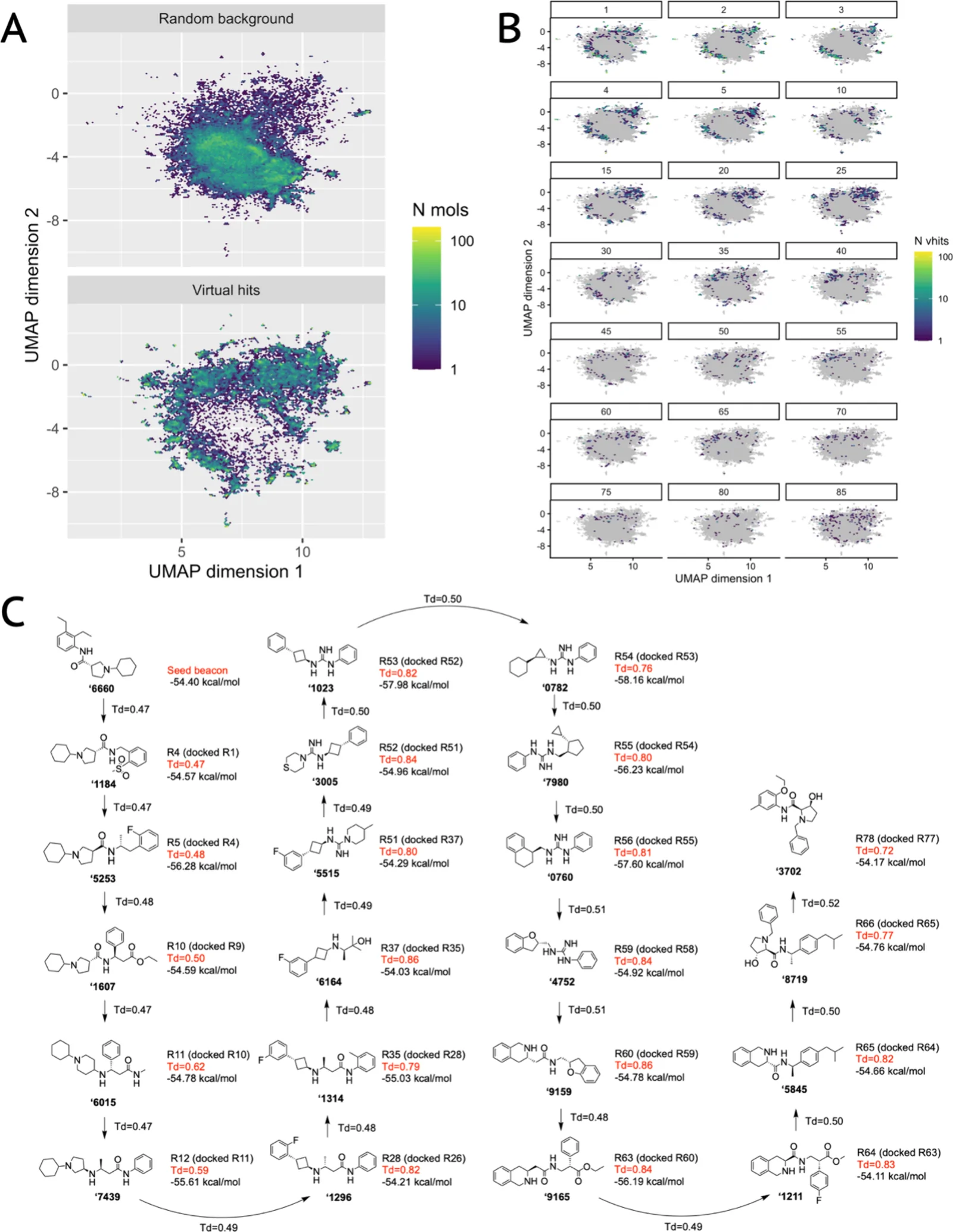
**Chemical space traversal and beacon selection for the Sigma2 docking campaign. A)** Uniform Manifold Approximation and Projection (UMAP) on the ECFP4 molecular fingerprints of the 47,131 Sigma2 virtual hits combined with 47,131 random molecules from the docked library. **B)** Same UMAP as **A** with the random background shown in gray and the virtual hits shown by the ChemSTEP round in which they were identified. The first five rounds are shown, followed by every fifth round. **C)** A representative sequence of beacons, where starting from a seed beacon (selected from the docked random seed set), an immediate descendant beacon is selected, followed by an immediate descendant beacon of that beacon, and so forth. The trajectory shown is one of the longest descendant chains identified by exhaustive tree traversal across the 100 round-1 beacons (Methods). The round where the compound was selected as a beacon is given, along with the round it was docked in. Its Tanimoto distance (Td) to its immediate parent is given on the arrow, with the Td to the initial seed beacon (in red) and the docking score also reported. An additional sequence is given in Figure S3.

### Prospective Docking of 360 Million Anions against AmpC β-Lactamase

Fortified by ChemSTEP’s ability to recover up to 92% of virtual hits in the large library retrospective campaigns, we turned to prospective testing of the method against the model enzyme AmpC β-lactamase (AmpC). In parallel with ChemSTEP prioritization from a library of 13.2 billion make-on-demand molecules from the Fall 2023 release of Enamine REAL Space,^37^ we exhaustively docked 360 million anions drawn from the same library (Methods) (Figure 4). Multiple previous docking,^15,29,32,33^ HTS,^31^ and fragment^38^ campaigns, and much literature^39–44^ suggest that AmpC almost exclusively recognizes anionic inhibitors. This is attributed to its conserved “oxyanion-hole” deriving from unpaired mainchain nitrogen atoms from residues Ser64 and Ala318 (Figure 4A).^33^ Consistent with this, only molecules that were predicted to be anionic at physiological pH were considered for synthesis in recent 99 M and 1.7B large-library docking screens against AmpC.^15,29^ The 360 million anions therefore capture the chemical space expected to contain the vast majority of inhibitors in the full 13.2 billion library, enabling a controlled comparison between ChemSTEP prioritization and exhaustive docking.

**Figure 4:**
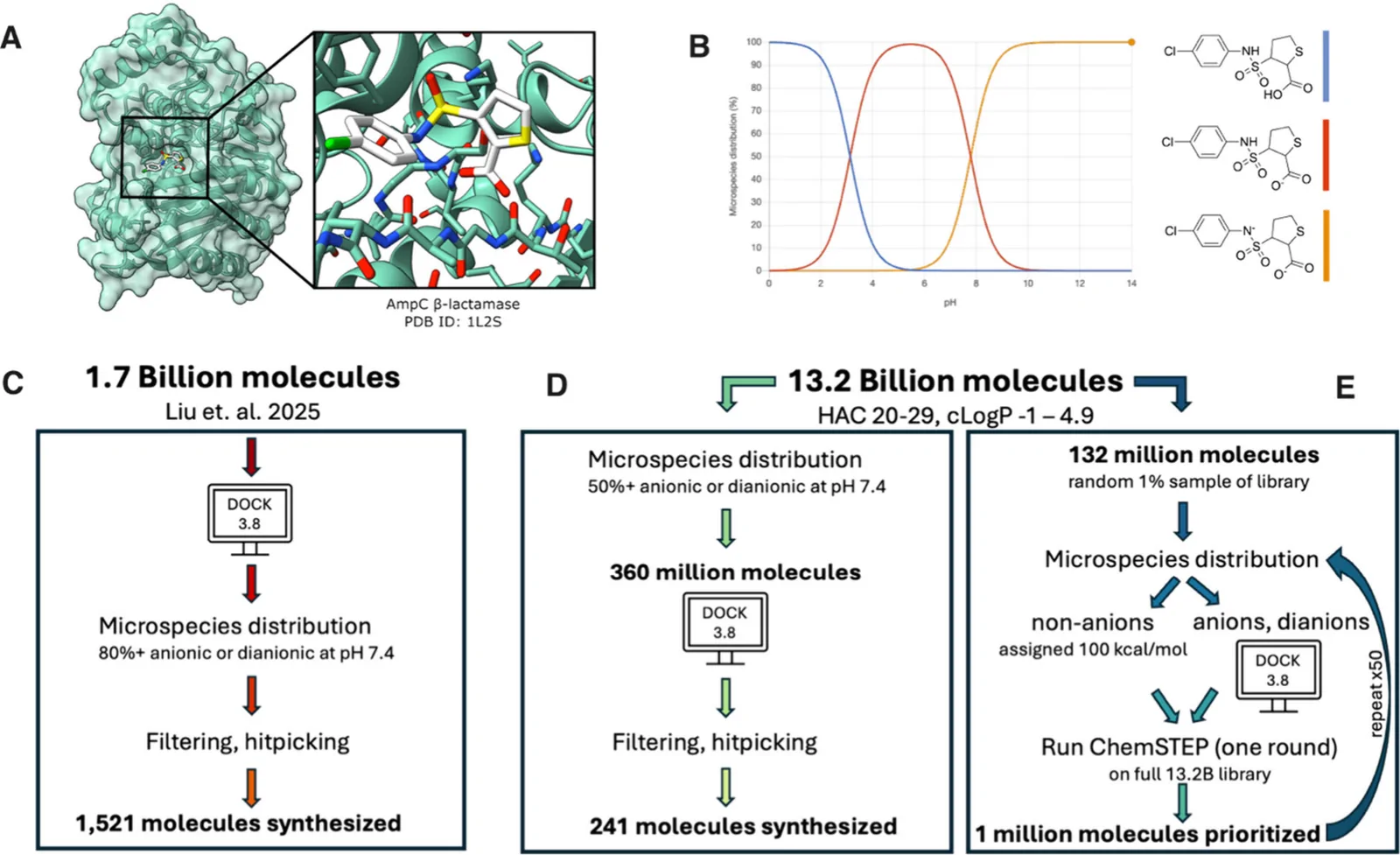
**Prospective ChemSTEP-accelerated docking of 13.2 billion molecules against AmpC β-lactamase. A)** Structure of AmpC used for docking, PDB ID 1L2S, bound to a sulfonamide-carboxylate inhibitor. **B)** An example of a microspecies distribution plot to determine anionic species at relevant pH levels. **C)** Workflow of the docking campaign in Liu et al. 2025:1.7 billion molecules were docked using DOCK3.8, in that case followed rather than preceded by microspecies distribution calculations to eliminate molecules that are not mono- or dianionic. This was followed by typical filtering and visual inspection. 1,521 molecules were synthesized and tested for activity. **D)** Brute-force docking of 360 million anions from the larger 13.2 billion space. Filtering and hit picking yielded 241 synthesized molecules that were experimentally tested. **E)** ChemSTEP docking workflow: microspecies distributions for a seed set of 132 million molecules were predicted to identify anions and dianions, which were docked normally, while nonanions were assigned an artificially high score. We then applied ChemSTEP to the full 13.2-billion-molecule library, prioritizing 1 million molecules per round in 50 rounds.

The 360 million anionic subset was prepared for use with DOCK3.8 ^29,45^ and docked and ranked against the AmpC structure represented by PDB ID 1L2S, with the same docking scoring grids and sampling as previously^15,29^ except that we only docked to one conformation of the enzyme, not four. Molecules prioritized by ChemSTEP in the first prospective run were processed identically: those predicted to be mono- or dianionic at physiological pH were fully built and docked using the same protocol, while nonanionic molecules were excluded by the modified scoring scheme described below. Because the 13.2 billion library is 8-fold larger than the 1.7 billion library, 8-fold more molecules scored with a pProp of 5.4 and above (top 1 in ∼ 250k), which was identified as the start of a high experimental hit-rate plateau.^15^ This is consistent with our previous observation that docking scores improve log–linearly as libraries expand (Figure S1).^46^

To provide a prospective set for the ChemSTEP-prioritized 13.2B screen, which was conducted in parallel with the brute force docking calculation, we prioritized 303 molecules ranked better that the pProp = 5.4 (top 0.0004%) score threshold. This threshold was established at −83.13 kcal/mol after scoring the 132 M ChemSTEP seed set with our modified scheme (Methods) and corresponded to the top 0.015% of the 360 M anions. Of these, 241 (79.5%) were successfully synthesized by Enamine and were tested for AmpC inhibition in a spectrophotometric assay. Of the 241, 106/241 (44%) were considered active, with an apparent *K*_i_ < 400 μM. Of these, 50 molecules inhibited with apparent *K*_i_ values <40 μM; 24 were <10 μM; and several were sub-μM inhibitors, among the most potent inhibitors discovered directly from docking or, indeed, high-throughput screens ^15, 29, 32, 38^ against AmpC. While all assays were performed in the presence of 0.01% v/v Triton X-100, which should disrupt colloidal aggregates^47^—a common source of experimental false positives^48–50^ —we also explicitly counter-screened ten potent inhibitors for aggregation; none formed particles at relevant concentrations by dynamic light scattering (Figure S4). Selected hits were tested in 8- or 12-point concentration–response assays (Figure 5C and Figure S5).

**Figure 5:**
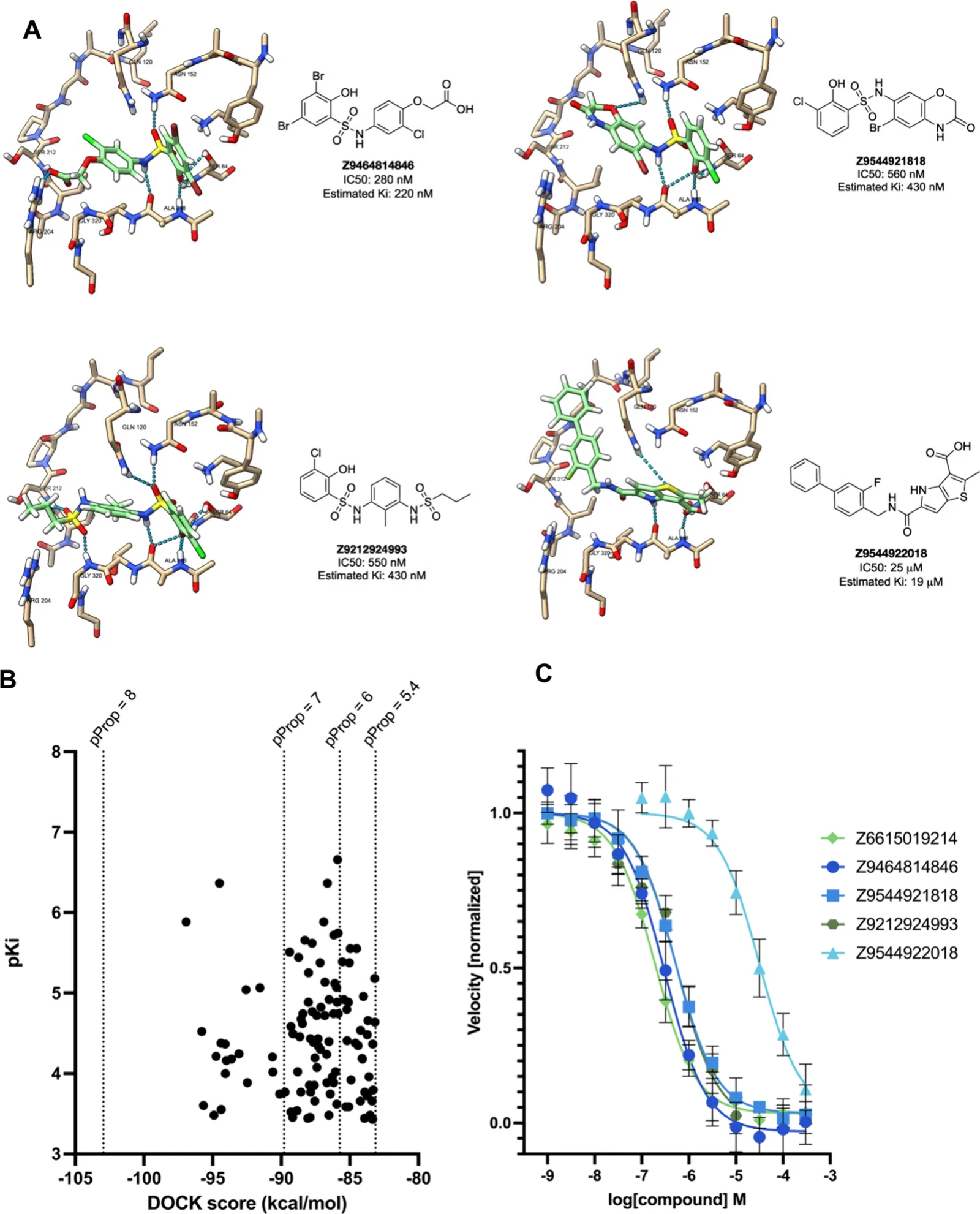
**Docking a 13.2 billion library against AmpC identifies inhibitors. A)** Docking predicted poses, 2D structures, experimental IC_50_ and apparent *K*_i_ values for select hits. AmpC is represented in beige and docked molecules in green. Hydrogen bonds are shown as dashed blue lines. **B)** Scatter plot of experimental pKi values vs DOCK3.8 score for 106 actives. **C)** Concentration–response curves for select actives. Z6615019214^15^ (positive control, diamonds, IC_50_ 220 nM) Z9464814846 (circles, IC_50_ 280 nM), Z9544921818 (squares, IC_50_ 560 nM), Z9212924993 (hexagons, IC_50_ 550 nM), and Z95449222018 (triangles, IC_50_ 25 μM). Additional curves for new docking actives are shown in Figure S5.

### Exploring a 13.2 Billion Virtual Library with ChemSTEP

In parallel to the brute force calculation (Figure 4D), we applied ChemSTEP on the 13.2 billion molecule virtual library from which the 360 million anions were drawn. Our scoring mirrored the constraint of the prior 1.7 billion AmpC campaign in which the full library was docked but of the high-ranking molecules only anions were ultimately prioritized for synthesis. Not only do anions dominate among previously known inhibitors^32,33,42,51–53^ but they made up over 96% of the 727 molecules in the top-scoring tranche from that screen, and most of the remaining were molecules that cheated the scoring function.^54^ Matching that constraint enables a like-for-like comparison between the two campaigns. A second motivation was practical: by docking all anions and dianions exhaustively, we obtained the complete docking score distribution for the plausible actives of the 13.2B library, which in turn enabled performing 100 replicate ChemSTEP runs. Concretely, only molecules predicted to be mono- or dianionic at pH 7.4 were fully built and docked (the 360 million), while other molecules were simply given an unfavorable score and neither built nor docked, but were used in the ChemSTEP similarity calculations. Critically, the modified scoring scheme applied only to the docking step. ChemSTEP itself operated on the entire 13.2 billion compound library using fingerprint similarity alone, with no knowledge of charge state; any molecule could be prioritized, though only those predicted to be anionic were docked. While the separation of the library into an anionic group that is fully docked and the rest of the library that is only screened by similarity is artificial—allowing us to run many replicate simulations—the search space traversed by ChemSTEP itself is nevertheless the full 13.2 billion library (Figure S6 and Methods). A full, real-world prospective test of the method awaits a campaign where all library molecules are available for docking.

The 13.2B library was constructed by selecting all molecules from the Fall 2023 release of Enamine REAL Space with heavy atom count between 20 and 29 and cLogP between −1 and 4.9. We chose pProp = 5.4 (roughly top 1 in 250,000) as our virtual hit definition for docking and selected a random subset of 1% (132 million) from the library to establish the background score distribution (around 5-fold more than our 10^pPop+2^ recommendation). We used 100 beacons per round with a target 1 million docked compounds per round and performed 50 rounds of ChemSTEP prioritized docking.

Because we ultimately docked all anions and dianions, we obtained the docking score distribution for all plausible actives for the 13.2B library. This enabled us to perform an additional 99 replicate ChemSTEP runs with different random seed sets for a total of 100 ChemSTEP runs (Figure 6A). Remarkably, all 100 runs produce similar prioritization curves despite starting from vastly different seed sets. Transforming these curves to show effective (implied) library size on the *y*-axis and the proportion of virtual hits recovered on the *x*-axis (Figure 6B) highlights the importance of the prioritization curve as a metric that allows the effective size of the explored space be estimated. This can be obtained from the following equation:

2 $$N_{\mathrm{eff}}=\frac{N_{\mathrm{vhits\_recovered}}}{N_{\mathrm{vhits}}}\times N_{\mathrm{library}}$$

**Figure 6:**
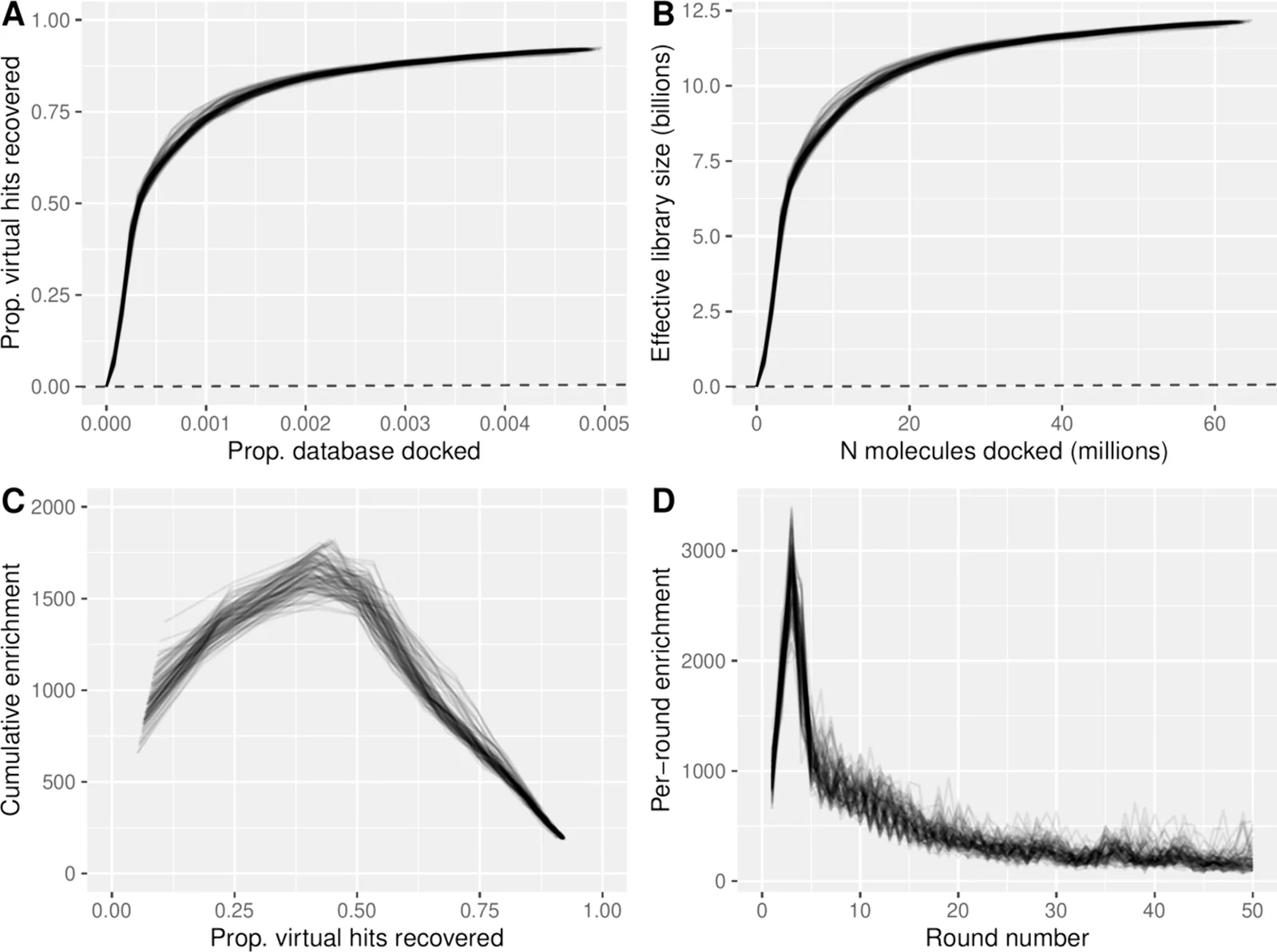
**ChemSTEP prioritization performance on the 13.2 billion virtual library. A)** Prioritization curve for 100 replicate ChemSTEP runs on the 13.2B library. A score threshold of −83.13 kcal/mol is used in all cases to be a virtual hit, and random seed sets of 1% of the library (132 million) are used to start ChemSTEP. The dashed line shows random performance. **B)** Same as A), with the number of molecules docked on the *x*-axis and the effective library size on the *y*-axis. **C)** Cumulative enrichment, which is the average slope over the prioritization curve, is given as a function of the proportion virtual hits recovered. **D)** Per-round enrichment is plotted for every round, correcting for the increasing prioritization difficulty as recovery increases.

Since N_vhits_ can be computed from the background distribution, N_vhits_recovered_ can simply be counted and N_library_ is known, N_eff_ can be reported. Applying ChemSTEP to the 13.2B library docked against AmpC, a recovery of ∼ 75% is reached at 0.1% of the library docked and an average of 91.9% recovery is reached by the time 0.5% of the library is docked. Said another way, by the time 0.1% of the overall library is prioritized by ChemSTEP, 75% of all the high-ranking molecules have been found. The effective size of the library docked is thus 9.9 billion. By the time 0.5% of the overall library is prioritized, the effective size of the docked library is 12.1 billion molecules. N_eff_ is readily calculated and can be used in most virtual screens by estimating a pProp range, which can be done by screening of a representative set, as here, or even by comparing to the scores of another screen where a library of a particular size was explicitly evaluated. N_eff_ can also be computed without knowing the total size of the library

3 $$N_{\mathrm{eff}}=N_{\mathrm{vhits\_recovered}}\times 10^{\mathrm{pProp}}$$

where pProp is the virtual hit threshold used. Consequently, even for relatively unbounded libraries, such as those using synthons^12,22^ or generative AI,^55^ the effective library size explored is readily calculated using N_eff_.

From a prioritization curve, cumulative enrichment curves can be derived as the slope from (0,0) to a given point on the curve. Enrichment directly corresponds to total speedup of the docking cost (assuming a negligible cost of the prioritization algorithm). In Figure 6C, we plot the cumulative enrichment against the recovery of virtual hits for all ChemSTEP runs. Enrichment reaches a peak of ∼ 1500 at 50% virtual hits recovered and steadily declines as recovery further progresses. Naturally, once 50% of the virtual hits have been recovered the remaining library, still over 99.95% of its original size, now contains only half the actives, making prioritization twice as hard. Assuming a negligible docked proportion of the library, the prioritization difficulty is given by

4 $$\mathrm{PD}=\frac{1}{1-P_{\mathrm{vhits}}}$$

where P_vhits_ is the proportion of virtual hits recovered. For instance, after 90% of the virtual hits have been recovered, it becomes 10-fold harder to find more.

Enrichment can also be computed per-round, correcting for this phenomenon by using the remaining proportion of virtual hits as the null expectation (Methods, eq 5). In Figure 6D, we give the per-round enrichment across 50 ChemSTEP rounds for the 100 replicate runs. Despite a sustained drop past the peak enrichment at rounds 3–4, round 50 still averages an enrichment of 200 ± 100. Taken together, these observations are promising for tera-scale docking with ChemSTEP: recovering 75% of virtual hits at 0.1% of the library docked means docking 1 billion molecules from a 1 trillion library to reach an effective size (N_eff_) of 750 billion. Early results suggest that ChemSTEP performance may improve with library size, perhaps reflecting a higher density of related chemotypes.

### Beacon Selection for AmpC

As in the Sigma2 campaign, we investigated how virtual hits (vhits) are distributed in chemical space and how beacons are selected for the first of the 100 replicate ChemSTEP runs on the 13.2B library. Figure 7A shows a UMAP (as in Figure 3A) of the 50,165 vhits (scoring better than −83.13 kcal/mol) from the 13.2B library combined with 50,165 random molecules. As was observed for Sigma2, the AmpC vhits are separated from where most library molecules concentrate. In contrast to Sigma2, AmpC vhits do seem concentrated in specific regions in addition to smaller scattered clusters, perhaps reflecting AmpC’s preference for specific anionic warheads and the well-known promiscuity of the Sigma2 receptor for hydrophobic cations. In Figure 7B, where the UMAP-represented virtual hits are shown for each round against the random background, no specific region seems to be prioritized in any given round. The early recovery is evident, with the highest densities of virtual hits achieved early on. We can then look at where in chemical space ChemSTEP recovers these virtual hits and in what round.

**Figure 7:**
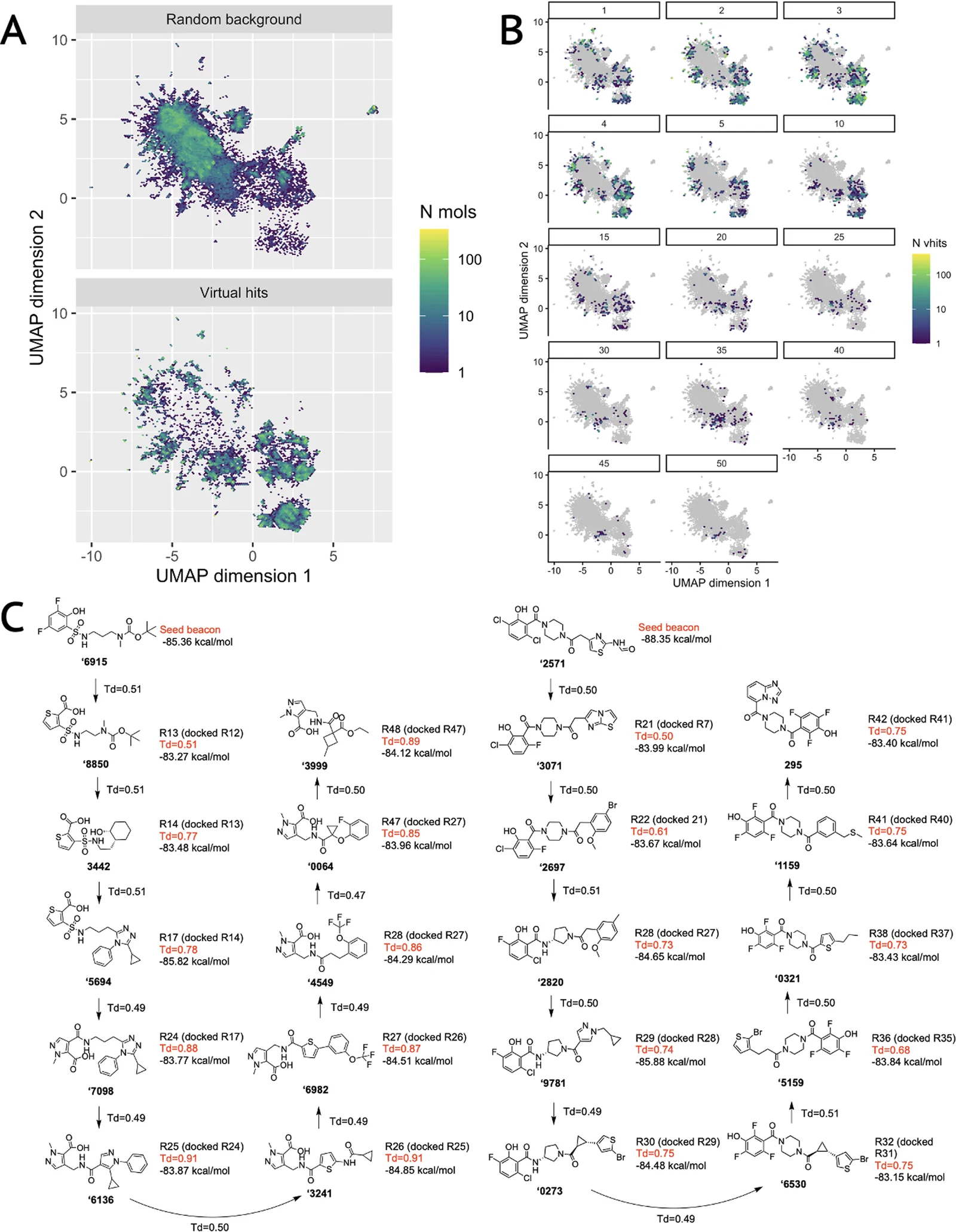
**Chemical space traversal and beacon selection for the 13.2B prospective campaign against AmpC. A)** Uniform Manifold Approximation and Projection (UMAP) on the ECFP4 molecular fingerprints of the 50,162 AmpC virtual hits combined with 50,165 random molecules from the 13.2B library. **B)** Same UMAP as **A**, with the random background shown in gray and the virtual hits shown according to the ChemSTEP round they were identified in. The first five rounds are shown, followed by every fifth round. **C)** Two representative sequences of beacons, where starting from a seed beacon (selected from the docked random seed set), an immediate descendant beacon is selected, followed by an immediate descendant beacon of that beacon, and so forth. The round where the compound was selected as a beacon is given, along with the round it was docked in. Its Tanimoto distance (Td) to its immediate parent is given on the arrow, with the Td to the initial seed beacon (in red) and the docking score also reported. Additional sequences are show in Figure S8.

After docking the 132-million-compound seed set in our first replicate run of ChemSTEP, 526 virtual hits were recovered, defined as having a DOCK score of – 83.13 kcal/mol or better (lower). From these 526, the top-scoring one was picked as the first beacon, followed by 99 additional beacons selected to be maximally diverse by Tanimoto distance (Figure S7), with 100 new beacons selected in each subsequent round of ChemSTEP to guide prioritization. In Figure 7C, we show two beacons selected in round one, and for each beacon, we can follow the prioritization path as determined by ChemSTEP. For example, molecule ‘6915 was selected as a beacon in round one. By round 13, molecule ‘8850 (docked in round 12) had been chosen as a new beacon through prioritization centered on ‘6915. The Tanimoto distances from molecule 18850 to both the original seed beacon and its immediate parent beacon are shown; in this case, they are the same molecule. As ChemSTEP progresses, prioritization remains centered around parent beacons at a Tanimoto distance of roughly 0.5, while distances from the initial seed beacon continue to diverge. In this example, the scaffold evolves from a sulfonamide phenolate to a classical carboxylate, both of which are well-precedented AmpC inhibitor chemotypes.^15,29^

### 13.2B ChemSTEP vs 1.7B Brute Force

Both modeling and empirical experience^9,11,15,29,46^ suggest that going from a 1.7 to a 13.2 billion library should afford higher experimental hit-rates and potencies if it were possible to do so. While brute-force docking a 13.2 billion library is beyond what can currently be done on academic resources, prioritization algorithms can enable thorough exploration of such spaces. We emphasize that ChemSTEP, like other prioritization algorithms, is not intended to outperform brute-force docking on a fixed library—by construction, no prioritization method can recover more virtual hits than exhaustive docking—but rather to extend the reach of docking to library sizes that brute-force cannot address. We thus wanted to learn if this “bigger-is-better” trend would be maintained in practice, and whether ChemSTEP would realize this potential. Because we hit-picked from what is effectively the full library, we can address both questions. To compare the 1.7B brute-force campaign with a representative prospective ChemSTEP run, we stopped our first run when we estimated reaching 80% virtual hit recovery (Methods). This 80% translated to 187/241 or 77.6% synthesized hits recovered. To compare this to the earlier 1.7B campaign against AmpC,^15^ we selected the top-scoring 187 molecules compounds from that campaign for comparison.

Comparing the two equal sets of molecules, one from brute-force docking of a 1.7 billion molecule library, one from ChemSTEP prioritization from the 13.2 billion molecule space, the ChemSTEP set realizes higher hit-rates for all hit definitions (Figure 8). Thus, among the inhibitors with apparent *K*_i_ values better than 13 μM, there are 27 prioritized by ChemSTEP and 11 for brute-force docking. The most potent active identified with ChemSTEP is a 220 nM inhibitor while the most potent identified in the corresponding top 187 scoring compounds from brute-force 1.7 Billion is an 860 nM inhibitor. We note that the 1.7 billion campaign did find a 460 nM inhibitor, but it was the 391th-ranked of the tested compounds by its docking score and so outside of this range. Thus, applying ChemSTEP on a 13.2B library yields more hits and better hit-rates than brute-force docking a 1.7B library. The best hit identified with ChemSTEP is almost 4-fold as potent as the best hit from the matched top-scoring set of brute-force compounds and, more convincingly, ChemSTEP outperformed the brute-force screen on the smaller library across all affinity ranges (Figure 8).

**Figure 8:**
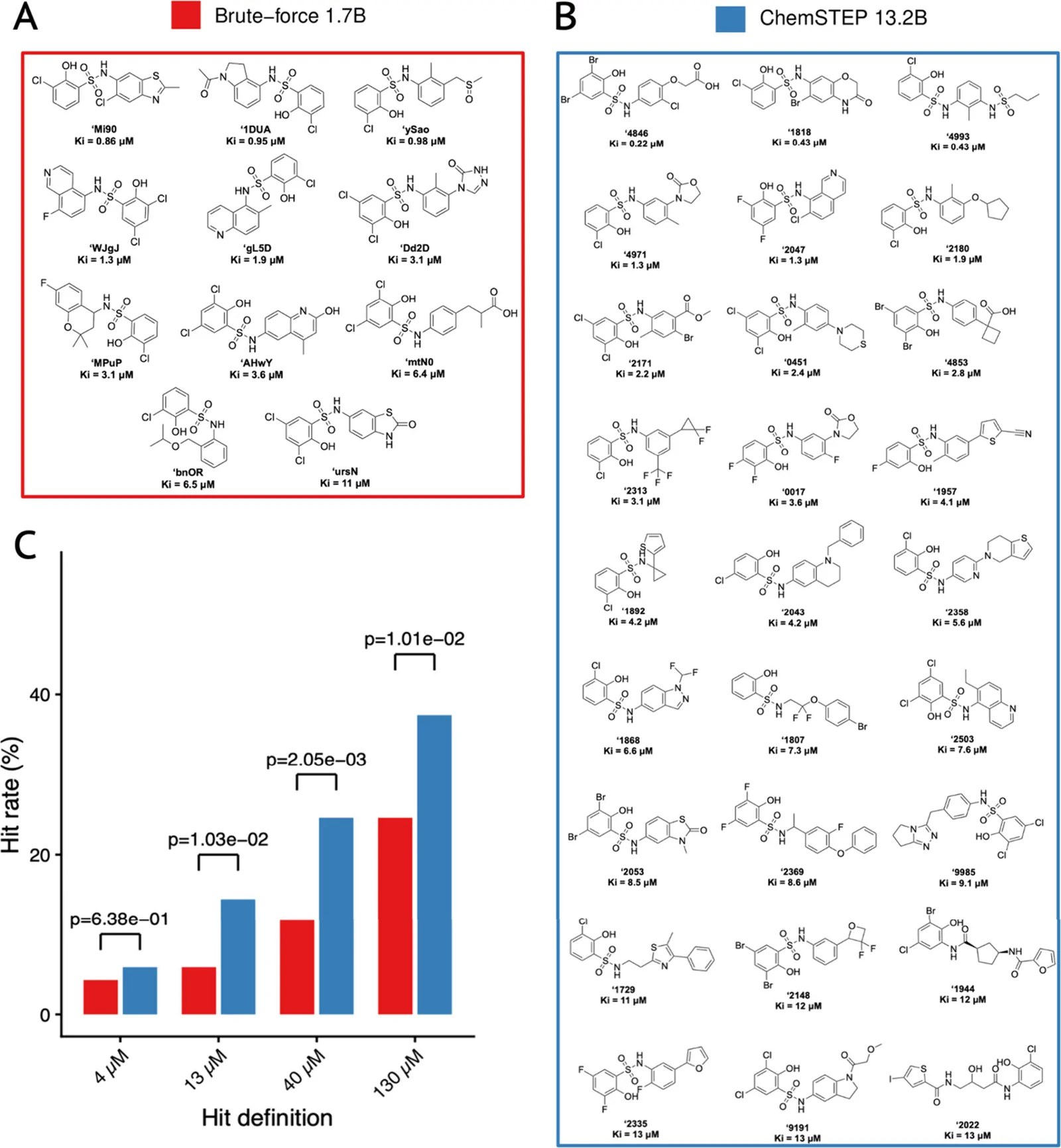
**ChemSTEP-prioritized docking of 13.2B achieves better hit-rates than brute-force docking 1.7B. A)** Inhibitors from the top 187-ranked molecules more potent than 13 μM from the brute-force 1.7 billion docking (11 inhibitors) and from the **B)** ChemSTEP 13.2B (27 inhibitors) screens. Cheng-Prusoff estimated Kis and compound IDs are shown; we estimate that these *K*_i_ values are accurate to ± 20%. **C)** The experimental hit rates from the 187 ChemSTEP-prioritized, manually hit-picked compounds from the first 22 rounds of prioritization (27,482,097 molecules docked) versus the hit rates from the 187 top-scoring, manually hit-picked compounds from the previous 1.7 billion screen. P-values reflect a chi-squared test.

We note that the most potent inhibitors from both the previous docking screen and the new one reported here are dominated by a sulfonamide phenolate warhead, which reduces apparent diversity among the actives. This warhead is ubiquitous among the more potent noncovalent AmpC inhibitors^15,29^ consistent with their dominance of both campaigns. Comparing the newly discovered inhibitors to those previously known, most have Tc values of 0.5 or less (Table S1), making them relatively far from the knowns (for context, Tc values of 0.35 or less for this fingerprint are typically scaffold hops). Moreover, for each of the previously known inhibitors that are closest to the new inhibitors, there are typically several thousand molecules in the 13.2 billion molecule library that are more topologically similar than the inhibitor ultimately prioritized by ChemSTEP (Table S1). For instance, one can ask how likely it would be to find the new 430 nM inhibitor Z9544921818 from similarity to a known. The closest known to ‘1818 is a 40 uM inhibitor that has an ECFP4 Tc of 0.44 (Table S1). Not only is this known 100-fold weaker than ‘1818 discovered here, but if one started from the known and searched the 13.2 billion based on similarity, one would find 55,869 molecules that are more similar to it (the known) than ‘1818. Thus, the likelihood of finding ‘1818 by similarity alone, without the prioritization that comes from docking, seems remote. Similar statistics for characteristic inhibitors may be found in Table S1. Moreover, if we allow ourselves to look beyond the most potent inhibitors, there the ChemSTEP prioritization finds multiple new families of molecules that no longer even have the phenolate sulfonamide and are even more dissimilar to previously known AmpC chemotypes (Figure S9 and Table S2).

As an aside, the potency ranges, from ≤ 130 to ≤ 4 μM, may seem relatively weak for the hit-rate comparisons between the brute-force and ChemSTEP screens (Figure 8), especially since as ligand concentration rises so too can artifacts. To control for artifactual inhibition, all inhibitors were measured in the presence of 0.01% Triton X-100, which will disrupt perhaps the greatest source of artifact for AmpC, colloidal aggregation.^31^ For characteristic inhibitors, we also specifically checked for colloidal aggregation by dynamic light scattering (DLS) finding no substantial scattering beyond baseline (**SI** Figure S4). We also ensured that characteristic weak inhibitors in the 100 μM range had well-formed concentration response curves and did not scatter light by DLS (**SI** Figure S10). Finally, interrogating activity in these concentration ranges allowed us to compare with the previous brute-force docking against AmpC,^56^ which also measured activities and hit rates in these ranges.

### Performance Gains from Stopping Early

Because we tested 241 compounds evenly spread across the virtual hit region, we can investigate whether ChemSTEP-selected subsets of compounds have lower or higher experimental hit-rates than the background performance of the whole library. In Figure 9, we show the hit-rate across the four hit definitions we’ve used previously. The overall hit-rate from the full set of 241 compounds is reported as a dashed red line, and the ChemSTEP hit-rates are averaged across all 100 replicate runs for each early stopping threshold. Early stopping preserves performance in all cases and enhances the hit-rate at higher potencies. Intrigued by this, we investigated where ‘**4846**, the 220 nM inhibitor, was found in each replicate. Surprisingly, it is found on average in round 5, at ∼ 50% virtual hits recovered and as early as the third round 35% of the time.

**Figure 9:**
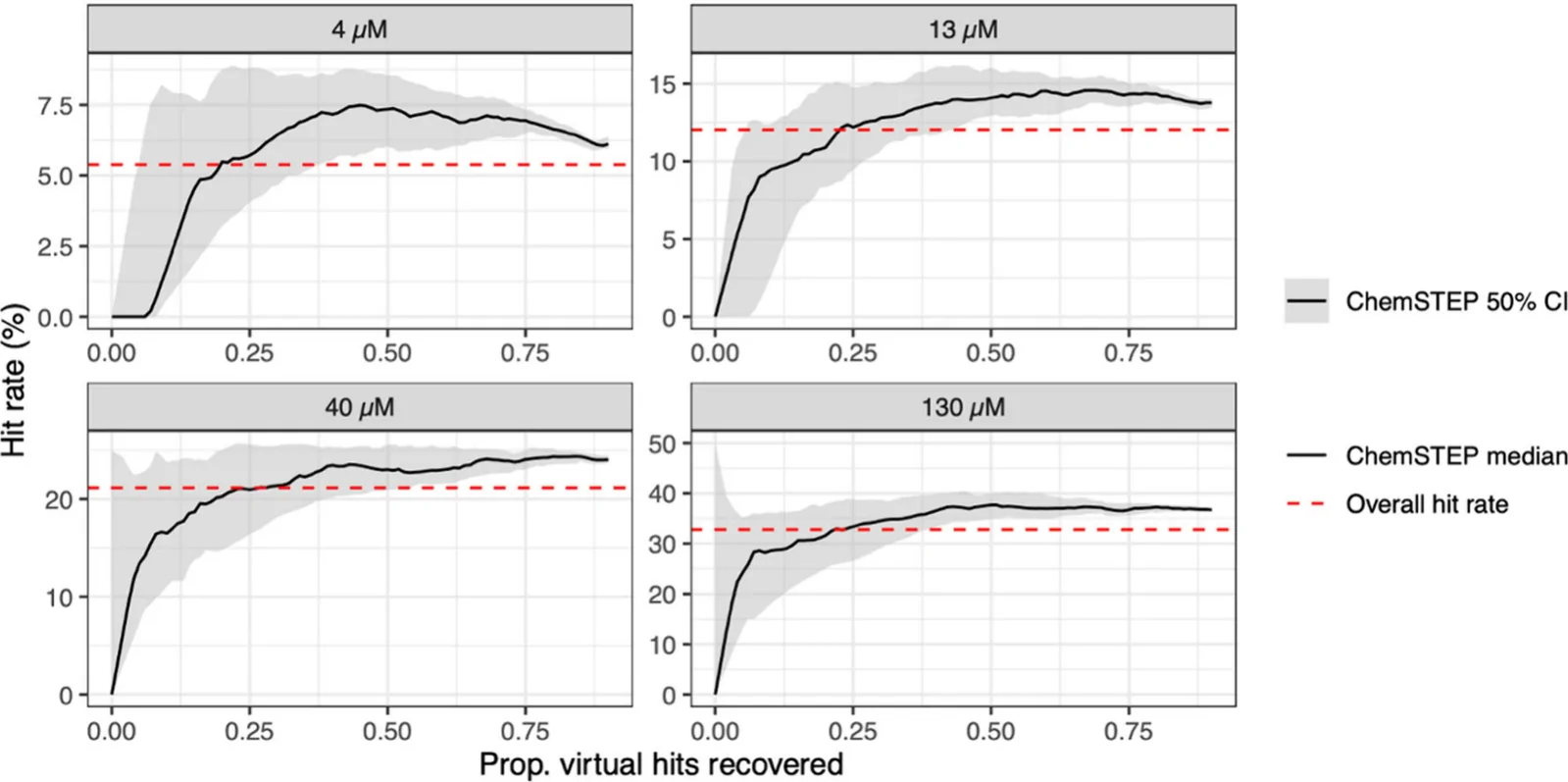
**Early stopping boosts hit-rate.** For each hit definition, the overall hit-rate from the 241 molecules hit-picked and synthesized from the pProp >5.4 virtual hit region is shown as a red dashed line. The hit-rate achieved by stopping early at a given proportion of virtual hit recovery is shown as the median from the 100 replicate ChemSTEP runs, with the 50% confidence interval shown in gray. The same four hit definitions as in Figure 8 are used. The full 100 curves are given in Figure S10.

## Discussion

Four observations from this study merit emphasis. **First**, ChemSTEP prioritizes competitively with brute-force docking while substantially reducing computational cost. Across 100 replicates on 13.2 billion molecules, speedups ranged from ∼ 500-fold at 80% recovery to ∼ 1500-fold at 50% recovery, with little variation between runs. Said otherwise, ChemSTEP recovered 50% of the virtual hits by docking 0.03% of the library, 80% by docking 0.15% and close to 92% by docking 0.5% of it. The method is fast enough that even at the trillion-molecule-library scale, docking the selected subsets of compounds dominates the compute cost (Methods). We anticipate that a 1-trillion library docking campaign may take less than a month on a 5k-CPU cluster and use at most 160 TB of storage (see Methods), and that a 1-billion library may be treated on an 8-CPU laptop in under a month, using under 160 GB of storage (the method scales linearly with library size). **Second**, the method is transparent. It requires no model training, depends on three interpretable user-defined parameters, and its decisions can be interrogated at each step; the chemical similarity-based logic behind ChemSTEP should be intuitive to most chemical biologists and medicinal chemists. Substantially higher experimental hit-rates are achieved if ChemSTEP is stopped early, supporting the notion that more populated families of compounds that are easier to stumble upon in the branching search hold better promise as potent ligands. **Third**, by expanding the library size 8-fold versus the largest screen previously conducted against AmpC, we achieved better experimental hit-rates and potencies; expanding library sizes continue to improve docking performance in the billion-scale regime. This, in turn, makes further expansion toward the tera-scale attractive. **Finally**, we introduce here two terms that may be broadly useful as the community moves toward trillion-molecule libraries. The effective size of the virtual library interrogated, N_eff_, may be calculated using equations **2** or **3**, and provides a way to compare prioritization to exhaustive screening at a given virtual hit definition (pProp threshold). Similarly, the prioritization difficulty, PD, is given by equation **4**, and may be a simple way for investigators to understand when they have reached points of diminishing return in large library campaigns. Together with pProp rescaling (equation **1**), these metrics facilitate comparison across libraries of different sizes.

Several caveats merit mention. **First**, at the scale where brute-force docking currently falls short (10+ billion libraries), we’ve only applied ChemSTEP prospectively to a single target, AmpC β-lactamase. AmpC is a model system for testing new docking and other screening methods^15,29,32,33^ and so is a target with which we are unusually familiar. While we do test ChemSTEP retrospectively on eight other targets for which large library screens are available,^26^ how the method will perform on other targets with other physical preferences remains uncertain, and further prospective studies are needed to test the generality of our observations. Our expectation is that ChemSTEP and related approaches will enable much larger library screens on targets against which structure-based approaches have traditionally worked well, including those with well-formed binding sites suited to metabolite and neurotransmitter recognition, but will struggle with the more open sites characteristic of protein–protein interfaces. **Second**, because we needed to ask questions about the virtual hits ChemSTEP misses, we used a hybrid approach where the library was essentially brute-force docked in parallel. **Third**, because ChemSTEP relies on chemical similarity to prioritize through the large libraries, it is uncertain how it will perform in series where “activity cliffs” are common. Lastly, while computationally applicable to tera-scale libraries, ChemSTEP still requires the computation of a 1024-bit ECFP4 fingerprint for each library molecule. Storing such libraries requires ∼ 128 terabytes of disk space per trillion molecules (Methods). While such storage is accessible to many investigators, it further moves tera-scale library docking into the realm of high-performance computing.

Notwithstanding these caveats, the major observations from this study should be clear. ChemSTEP performs well both by enrichment and by total recovery of virtual hits in docking campaigns. Its transparent nature may afford it a low technical barrier to entry. While the exploration of tera-scale libraries with ChemSTEP will require specialized resources, applying it to virtual libraries on the order of a billion compounds—still often state-of-the-art—is within reach on standard desktop computers. In an apples-to-apples comparison, expanding to a 13.2 billion molecule library from a 1.7 billion molecule library improved hit-rates and potencies, extending earlier trends and supporting exploration of larger libraries still. Finally, we introduce several metrics that may help investigators calculate the effective size of the library they have explored, and how the difficulty of finding the next high-ranking molecule changes over the course of the campaign.

## Methods

### ChemSTEP Algorithm

Running the Chemical Space Traversal and Exploration Procedure (ChemSTEP) prospectively generally consists of three steps: (i) seed set docking and score threshold selection; (ii) iterative cycles of beacon selection, full library prioritization, prioritized subset docking; and (iii) convergence assessment.

In step (i), a uniformly sampled random seed set is explicitly docked to establish an empirical score distribution for the library and to determine a docking score threshold corresponding to a chosen virtual hit definition (pProp). We recommend a seed set size of at least 100 × 10^pProp^ (see Figure S2). All molecules scoring at or beyond the determined threshold are designated virtual hits and constitute the initial pool for beacon selection.

In step (ii), a fixed number of beacons (typically 100 per round) are selected from the current set of virtual hits using a greedy max-min diversity procedure: the highest-scoring virtual hit is selected as the first beacon, and each subsequent beacon is chosen to maximize its minimum Tanimoto distance (Td) to the previously selected beacons, ensuring chemical diversity. Using 1024-bit Morgan (R = 2) fingerprints, every yet-undocked molecule in the full library is ranked by its minimum Td to any beacon. A one-dimensional array of current minimal Td values for all yet-undocked molecules is maintained, such that only Td to the set of newly selected beacons needs to be computed each round (and the minimum value is kept). A user-defined number of top-ranked molecules (e.g., 1 million per round in the 13.2B study) are then docked. Newly identified virtual hits are added to the pool, a new diverse set of beacons is selected, and the process is iterated. Molecules already docked are excluded from further prioritization.

In step (iii), convergence is assessed by monitoring the cumulative number of virtual hits recovered relative to the estimated total number of virtual hits in the library. This yields a prioritization curve (fraction of virtual hits recovered versus fraction of library docked), from which enrichment and effective library size (N_eff_) can be calculated as described in the Results. Iterations continue until a predefined recovery level, round limit, or compute budget is reached.

### Eight Retrospective Targets

For the eight retrospective large-scale docking campaigns annotated in the LSD database,^30^ the complete docking score lists were available. ChemSTEP was simulated by treating score lookup as a proxy for docking. For each target, ten independent seed sets were generated by uniform random sampling such that the expected seed set size was 100 × 10^pProp^ (with pProp = 4, corresponding to 1,000,000 molecules). Up to 100 beacons were selected per round, and 0.04% of the library was prioritized and “docked” per round until 5% of the library had been evaluated.

### UMAP

To visualize chemical space organization of virtual hits, Uniform Manifold Approximation and Projection (UMAP) was applied to 1024-bit Morgan (R = 2) fingerprints. For each target analyzed (Sigma2 and AmpC), all virtual hits were combined with an equal number of uniformly sampled random library molecules. UMAP was run with default nearest-neighbor and minimum distance parameters using cosine distance in fingerprint space. The resulting two-dimensional embeddings were used solely for visualization and qualitative assessment of chemotype clustering and traversal; they were not used in any prioritization step.

### Beacon Genealogy

To analyze chemical space traversal, we reconstructed beacon genealogies by linking each newly selected beacon to the prior beacon to which it had maximum Tanimoto similarity at the time of its prioritization. This establishes parent-child relationships across rounds. For each seed beacon (selected from the seed set for the round 1 library search), we identified descendant beacons in subsequent rounds and computed the longest chain of descendants using exhaustive tree traversal.

### 13.2 Billion Library

To constitute the virtual library, we selected every molecule from the enumerated Fall 2023 release of Enamine REAL Space that had between 20 and 29 heavy atoms, inclusive, and that had a cLogP value between −1 and 4.99. This amounted to 13,178,504,161 molecules. For every molecule in the library, we computed its Morgan fingerprint, R = 2, on 1024 bits with RDKit. These were stored as NumPy binary arrays, taking 128 bytes of disk storage per compound. The total library in ChemSTEP-searchable format thus amounted to ∼ 1.6 TB of disk storage for the fingerprints.

### pProp Threshold for AmpC and Scoring Scheme

Based on the empirical hit-rate curve on AmpC, the start of the plateau in hit-rates happens between pProp = 5 and pProp = 6. For our virtual hit definition, we set the pProp target at 5.4, which corresponds to roughly 1 molecule in 250k. On our initial seed set, this amounted to a score threshold of −83.13 kcal/mol. On all subsequent ChemSTEP runs, we manually set the score threshold to the same −83.13 value. Because we ultimately docked all anionic and dianionic molecules, we could calculate that there are 50,165 such molecules in the library. This allows us to calculate that our −83.13 threshold corresponds to a pProp of 5.42, close to our target pProp of 5.40.

### Modified Scoring Scheme

For the prospective study, only molecules predicted to be mono- or dianionic at pH 7.4 were fully built and docked, while all other molecules were assigned a score of 100 kcal/mol and were not built or docked. The high score prevented these compounds from being selected as virtual hits (top-scoring compounds always have negative scores with well-behaved DOCK3.8 setups). This modified scoring scheme applied only to the docking step; ChemSTEP itself operated on the complete 13.2-billion-molecule library using fingerprint similarity alone, without knowledge of which molecules were anionic. Thus, any molecule in the 13.2B library could in principle be prioritized, and nonanionic molecules were excluded from becoming virtual hits solely through their assigned unfavorable docking score.

Protonation state distributions at pH 7.4 were calculated using ChemAxon’s cxcalc command-line tool (Marvin). For each molecule, microspecies populations were computed at pH 7.4, and compounds were classified as monoanionic if the summed population of microstates with net charge –1 exceeded 50%, and as dianionic if the summed population of microstates with net charge –2 exceeded 50%. Only molecules meeting one of these criteria were fully built and docked in the prospective campaign; all others were assigned a score of 100 kcal/mol as described above.

### Prospective ChemSTEP Run

The seed set contained 131,630,158 molecules, of which 526 scored at or below our score threshold of −83.13 (pProp = 5.4). Extrapolating from this, we expected 526/131,630,158 × (13,178,504,161–131,630,158) = 52,136 virtual hits in the remaining ∼ 99% of the library. Because we wanted to investigate whether the experimental hit-rates would be different between the ChemSTEP-prioritized and full virtual hit region, we decided to stop the prospective ChemSTEP run at 80% recovery, or 41,709 additional virtual hits recovered. The stopping criterion was defined using the estimated total number of virtual hits derived from the seed set (52,136), not from knowledge of the full library distribution (actual number of vhits 50,165). That value (41,709 additional vhits) was reached after 22 rounds of ChemSTEP, with 100 beacons per round and a target of 1 million molecules docked per round. In the context of the full score distribution, the 41,709 additional vhits correspond to a recovery of 84% instead of our targeted 80%. A total of 27,482,097 molecules (or 0.21% of the library) had been docked by that time, with 42,016 virtual hits found, giving an effective speedup of 383-fold. We still continued the ChemSTEP prioritization for a total of 50 rounds and ultimately ran 50 rounds from each of the 100 random seed sets, keeping −83.13 kcal/mol as the virtual hit threshold for consistency.

The per-round enrichment is obtained with the following equation

$$E_{\mathrm{RX}}=\frac{N_{\mathrm{vhits\_Rx}}}{N_{\mathrm{docked\_Rx}}}\times 10^{p\mathrm{Prop}}\;\times \;\mathrm{PD}$$ 5

where E_Rx_ in eq 5 is the per-round enrichment for Round X, and PD (the prioritization difficulty) is obtained for Round X-1 using eq 3.

### Hit Picking

After a target virtual hit recovery or a fixed compute budget has been reached, hit-picking can proceed as usual from all the virtual hits docked so far. Our hit picking strategy closely resembled that of Liu et al. 2025.^15^ From the 360 million anions and dianions, all molecules with a DOCK score of −83.13 kcal/mol or lower (50,165 molecules) were put through LUNA 1024-length fingerprint generation.^57^ We removed molecules with more than one hydrogen bond donor or more than six hydrogen bond acceptors that were not compensated with polar interactions with the protein, leaving 46,746 molecules. These were clustered for diversity at a Morgan-2 Tc = 0.32. This left us with 6,941 total cluster heads, indicating that the virtual hit pool spans thousands of distinct chemotypes despite AmpC’s strong preference for anionic ligands. These 6,941 were filtered for novelty against known AmpC inhibitors at Tc = 0.55. This emerged from replicating as closely as possible the 1.7B AmpC campaign, which used a Tc = 0.50 threshold on ECFP4 fingerprints. Because we used Morgan R = 2 fingerprints as close equivalents to ECFP4, we recomputed the distance to known inhibitors for all the synthesized hits from the 1.7B study and found that the 0.50 ECFP4 Tc threshold corresponds to a 0.55 Morgan R = 2 threshold. We thus used 0.55 as our novelty cutoff, in addition to a Tc = 0.75 cutoff to molecules synthesized in the 1.7B study. Cluster heads, and additional molecules from large clusters, were visually inspected and 241 were chosen for synthesis and experimental testing.

### Compute and Space Cost of 1 Trillion Fingerprint Library

To establish a 1-trillion fingerprint library, compounds need to be enumerated, and 1024-bit fingerprints need to be computed and stored along with the compressed SMILES representations. With RDKit’s implementation of Morgan R = 2 1024-bit fingerprints, ∼ 1,000 fingerprints can be computed per second, per CPU core. Enumeration happens at ∼ 10,000 compounds per second, such that the fingerprint is the bottleneck. We note that there are other similarity searching methods that avoid full enumeration and might plausibly reduce this computational cost.^58^ For 1 trillion compounds, we get

$$\frac{10^{12}molecules}{1,000Fp\sec^{-1}{CPU}^{-1}}=1,000,000,000CPU\sec onds$$

which corresponds to 2.3 days on a 5k-CPU cluster. In terms of space, the fingerprints take 128 bytes × 10^12^, or 128 terabytes of storage. The compressed SMILES strings take ∼ 15 bytes per molecule, or 15 terabytes of storage. The total database can thus be stored with 153 terabytes of disk space. Once the 1-trillion library is computed and stored, it can be reused indefinitely with any docking setup.

### Compute Cost of 50% Recovery from 1 Trillion Molecule Prospective Screen

We’ve empirically observed an overall ChemSTEP performance, including I/O, of 2 M Tanimoto calculations per second per CPU on 1024-bit fingerprints. To perform 50 rounds of ChemSTEP on a 1 trillion library, with 100 beacons per round (same parameters as the 13.2B screen performed here), would thus require

$$\frac{10^{12}molecules\times 100beacons\times 50rounds}{2,000,000Tcs\sec^{-1}{CPU}^{-1}}=2,500,000,000CPU\sec onds$$

which corresponds to 5.8 days on a 5k-CPU cluster. Assuming a similar prioritization curve to what we obtained in Figure 6A, to reach 50% recovery 0.033% of the library needs to be docked, or 330 million compounds in the case of a 1-trillion library. At ∼ 30 s/CPU for building and docking each compound, this requires

$$330,000,000molecules\times 30\sec{CPU}^{-1}=9,900,000,000CPU\sec onds$$

which corresponds to 22.9 days on a 5k-CPU cluster. Thus, the whole endeavor can be over in less than a month, with a N_eff_ of 500 billion (50% recovery). Both the similarity search and the prioritized docking scale linearly with library size, but both are embarrassingly parallel, so wall-clock time is set by the available CPU count rather than by the library size itself.

### Compound Synthesis

All molecules were sourced from Enamine. Synthetic routes and HPLC traces can be found in Supporting Information.

### AmpC Enzymology

All compounds were dissolved in dimethyl sulfoxide (DMSO) at 30 mM, and further diluted as necessary in 50 mM Na Cacodylate, pH 6.5 buffer to maintain a constant 1% v/v DMSO concentration.

Compounds were initially screened in a three-point spectrophotometric inhibition assay at final inhibitor concentrations of 40, 100, and 300 μM. The β-lactamase substrate nitrocefin (Km = 180 μM) was used at a final concentration of 50 μM, such that [S]/Km = 0.28. Using a BMG Labtech Clariostar, substrate and protein (2 nM final concentration) were dispensed into a 96-well plate containing inhibitors in buffer and absorbance of light at 490 nm was measured for 55 s after addition. Rates were measured in triplicate for each compound, and IC50 was calculated by fitting percent inhibition relative to a no-inhibitor control across the three tested concentrations to a dose–response equation with a Hill coefficient of 1. Approximate *K*_i_ values were calculated using the Cheng-Prusoff equation, $\left(Ki=\frac{IC50}{1+\frac{\left[S\right]}{Km}}\right)$ .^59^ For 28 inhibitors from this initial screen, full 8- to 12-point concentration–response curves were measured using a final nitrocefin concentration of 50 μM and inhibitor concentrations ranging from 1 nM to 500 μM (Figure S5).

### Aggregation Testing

For all experimentally tested molecules, water-octanol partition coefficients (cLogP) were calculated using RDKit. The ten highest-affinity molecules with a cLogP > 3.5 were tested for colloidal aggregate particle formation by dynamic light scattering at a 31.6 μM concentration in 50 mM potassium phosphate buffer, pH 7, with 1% DMSO.^47,48,50^ There was no indication of colloidal particle formation for any molecule tested under these conditions. (Figure S4).

## Supplementary Material







## Data Availability

DOCK3.8 is freely available for academic use (https://dock.compbio.ucsf.edu/). We release ChemSTEP as an open-source Python package (https://github.com/mailhot-lab/chemstep_package). All experimental results are included in the manuscript as are many docking results, any others may be found on the LSD Web site^30^ or by writing to the authors.

## Notes

Figures S1–S10, Tables S1–S2, and synthetic procedures (PDF)
HPLC traces for experimental hits (PDF)
Compound SMILES, experimental *K*_i_ values, and DOCK scores for all compounds tested for AmpC inhibition (XLSX)

## ∇

Institute for Research in Immunology and Cancer (IRIC) and Faculty of Pharmacy, Université de Montréal, Montréal, Quebec H3C 3J7, Canada orcid.org/0000-0002-7533-4561
