# Supplementary material for: Chemical Distance-Based Acceleration of Large Library Docking with ChemSTEP
Source: ACS Cent Sci. 2026 Jul 20;12(8):1178–94. doi: 10.1021/acscentsci.6c00396 (PMC13523501; doi:10.1021/acscentsci.6c00396)
Supplement: Supplementary file 1 [file oc6c00396_si_001.pdf]

Supplementary Information for:

Chemical Distance–based Acceleration of Large Library Docking with ChemSTEP

Olivier Mailhot[1,†,‡,*], Katie L. Holland[1,†], Lu Paris[1], Dmytro S. Radchenko[2], Yurii S. Moroz[2,3,4], John J. Irwin[1,*] & Brian K. Shoichet[1,*]

[1]Department of Pharmaceutical Chemistry, University of California, San Francisco, CA 94143-2550, USA.

[2]Enamine Ltd, Kyïv, 02094, Ukraine.

[3]Chemspace LLC, Kyïv, 02094, Ukraine.

[4]Department of Chemistry, Taras Shevchenko National University of Kyïv, Kyïv, 01601, Ukraine.

[†]Equal contribution.

[*]email: o.mailhot@umontreal.ca, jir322@gmail.com, bshoichet@gmail.com

[‡]Current affiliation: Institute for Research in Immunology and Cancer (IRIC) and Faculty of Pharmacy, Université de Montréal, Montréal, Canada.

Table of Contents

I. SUPPLEMENTARY FIGURES……………………………………………………………….. S3

i. Figure S1: Relationship between library size and potency……………………………. S3
ii. Figure S2: Error on estimation of pProp thresholds from library samples.…..…. S4
iii. Figure S3: Sigma2 beacon genealogy………………..…………………………… S5
iv. Figure S4: Dynamic light scattering for aggregation determination……………… S6
v. Figure S5: Concentration response curves for 28 new AmpC inhibitors……….. S7
vi. Figure S6: Number of anions prioritized…………….……………………………….. S10
vii. Figure S7: Round 1 ChemSTEP beacons for AmpC.…………………………… S11
viii. Figure S8: AmpC beacon genealogy………..…………………………………… S12
ix. Figure S9**:** Examples of experimental actives with atypical chemotypes………. S13
x. Figure S10: Characterization of medium-affinity hits…………………………… S14
xi. Figure S11: Early stopping full curves………………………………………….. S15
xii. Table S1: Similarity comparison of hits from the previous brute-force screen…. S16
xiii. Table S2: Similarity comparison of atypical experimental actives……….……. S20

II. SYNTHETIC PROCEDURES………………………………………………………….. S21

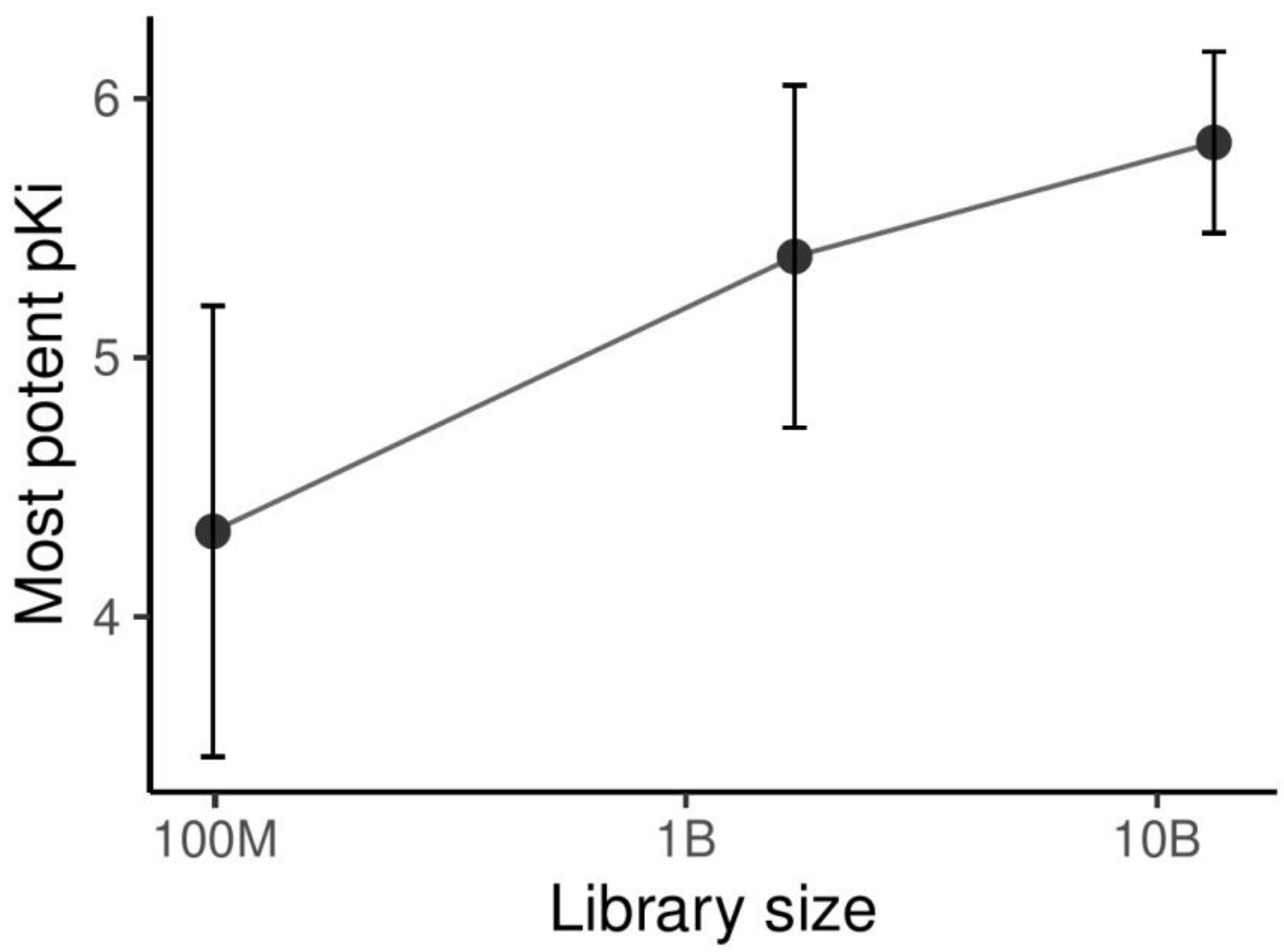

Figure S1. A log-linear relationship between library size and potency. 44 molecules (the number tested in the 99M screen) are sampled from each set of top-scoring tested compounds for a total of 100,000 bootstrap iterations. The mean maximal pKi +- standard deviation is reported.

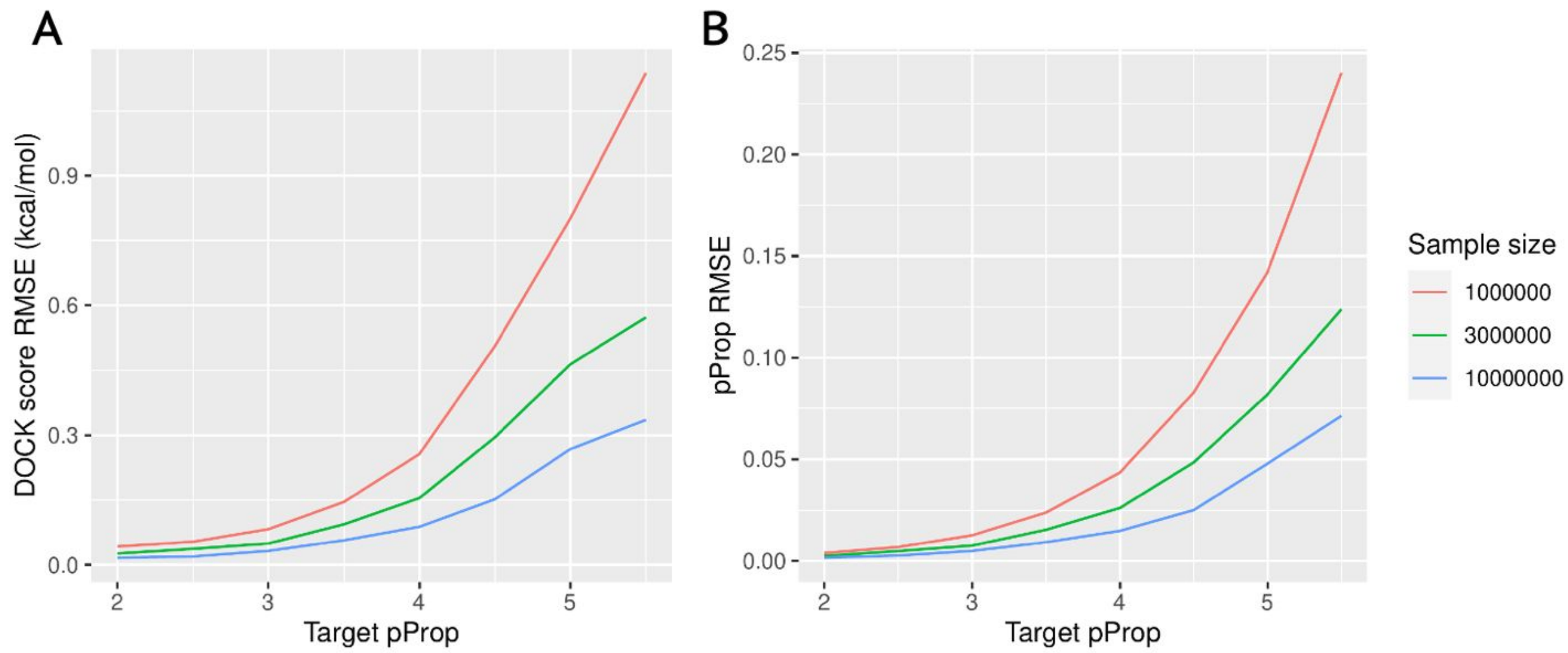

Figure S2. Error on estimation pProp thresholds from library samples. A**)** DOCK score RMSE is reported for estimated pProp values ranging from 2 to 5.5 across sample sizes of 1M, 3M and 10M. The scores from the 1.7B AmpC campaign are used to draw the samples and establish the true score threshold corresponding to the target pProp value. **B)** The RMSE on the pProp value itself is reported (difference between target pProp and actual pProp corresponding to the score threshold selected). On both graphs, it is apparent that using a 10-fold larger sample size right-shifts the curve by a whole unit.

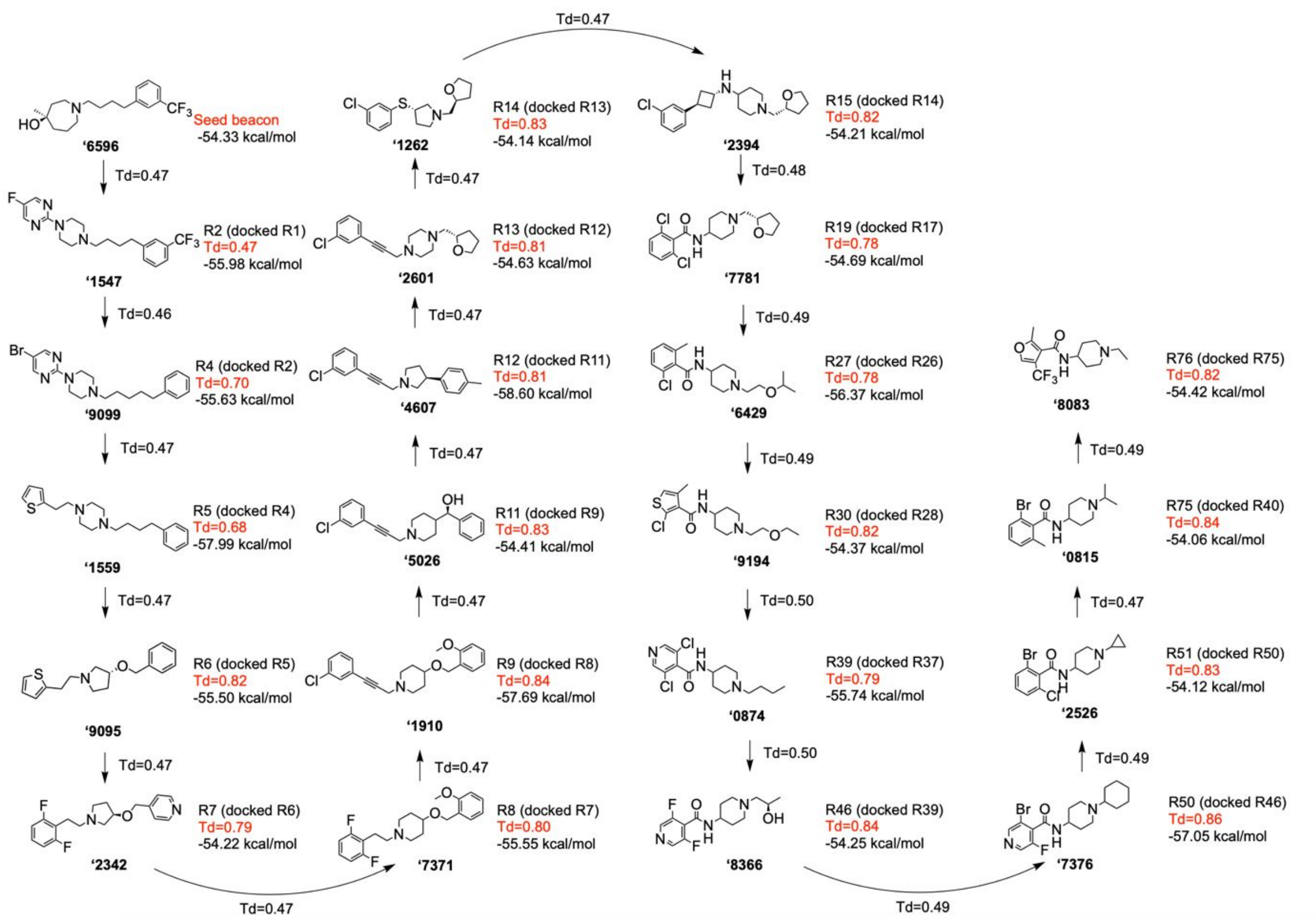

Figure S3. Additional beacon genealogy for retrospective Sigma2 ChemSTEP application.

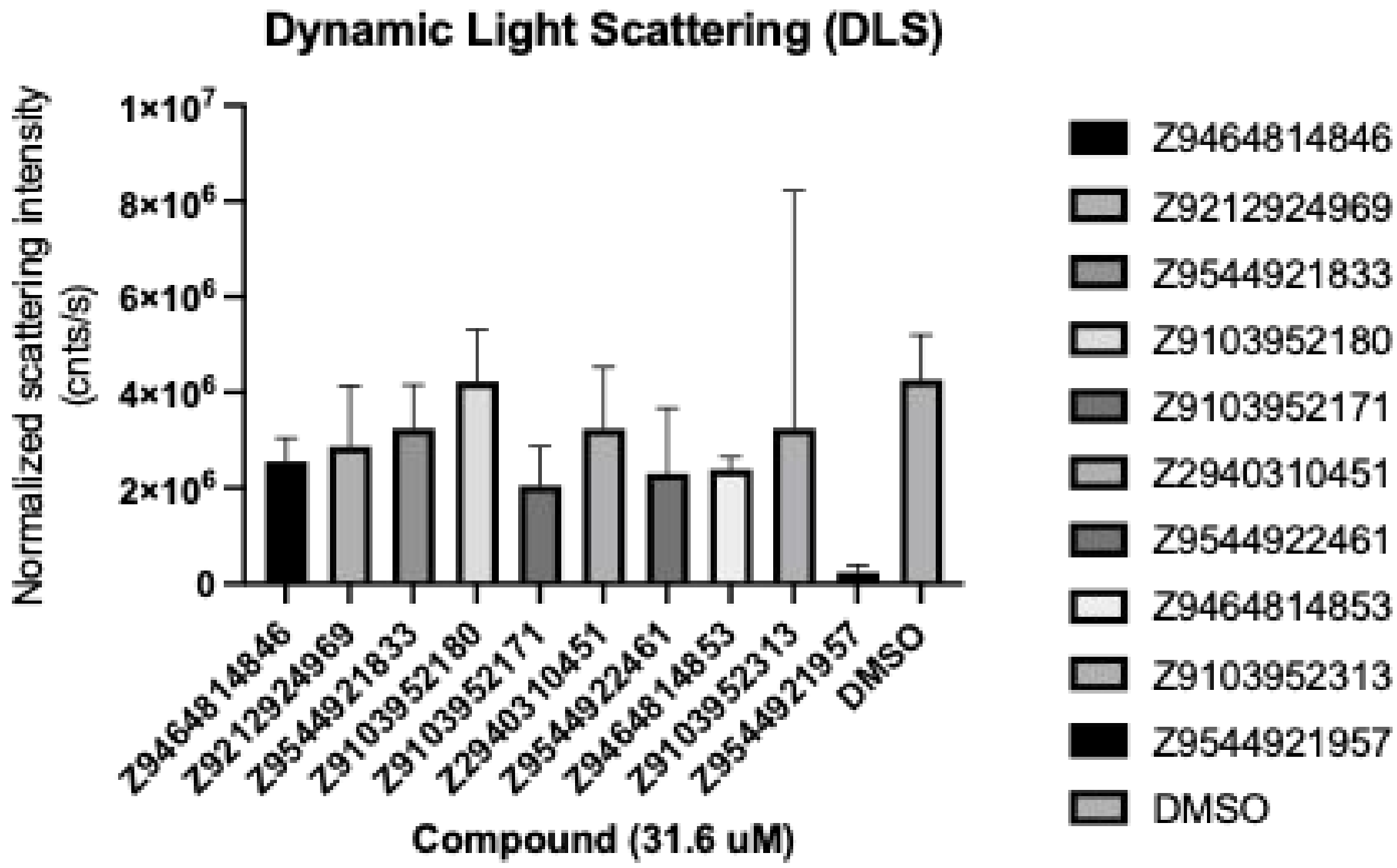

Figure S4. Dynamic light scattering for aggregation determination. Compounds tested at a concentration of 31.6 µM (see Methods).

Z6615019214
Positive Control

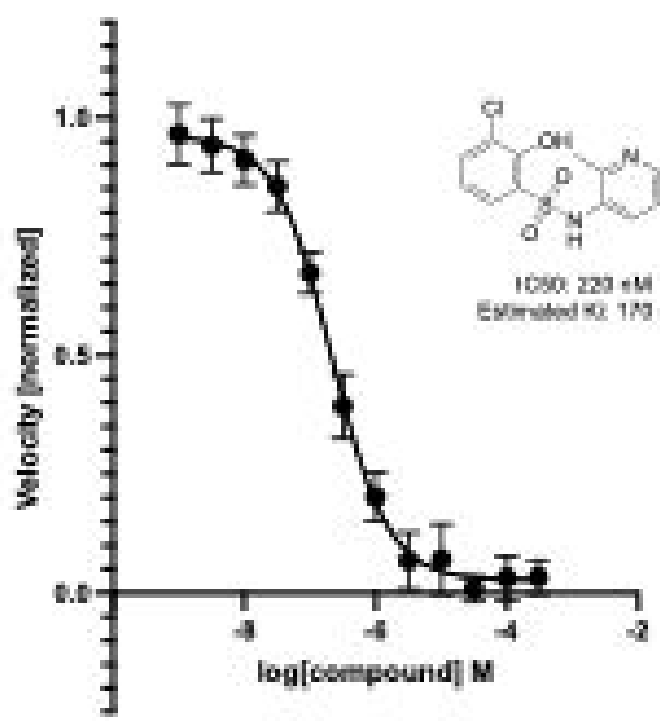

Z9464814846

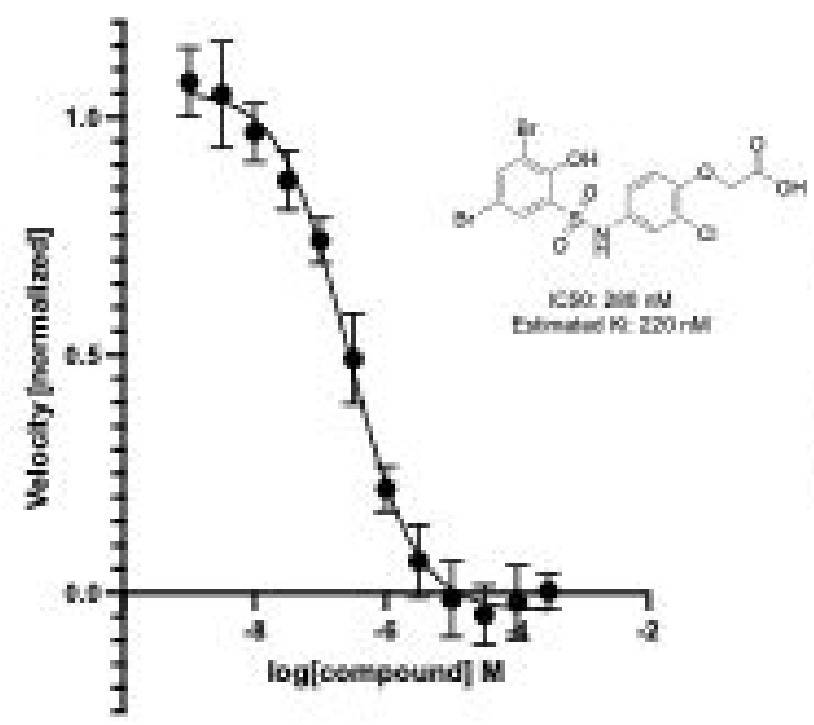

Z9212924993

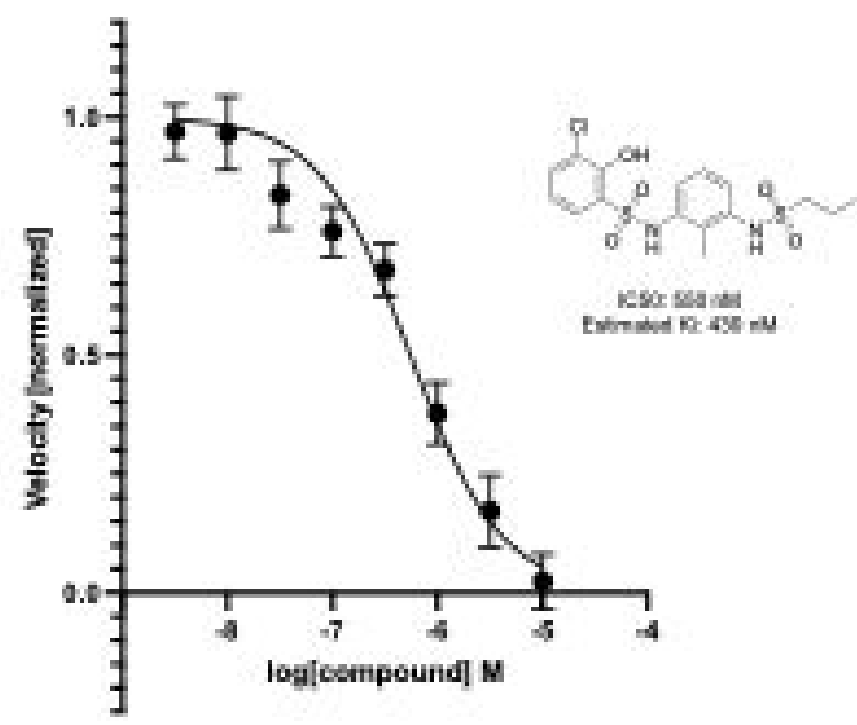

Z9544921818

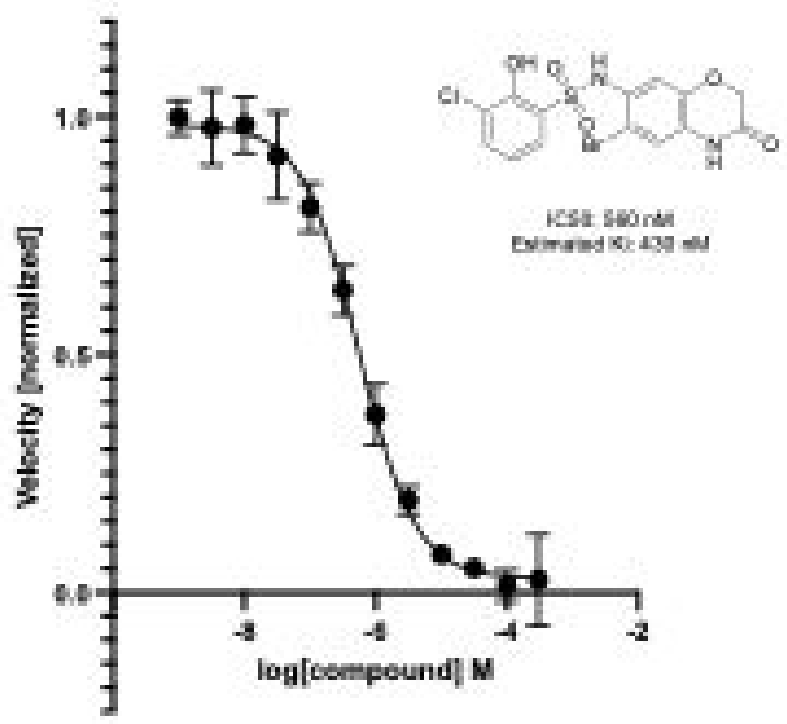

Z9212924971

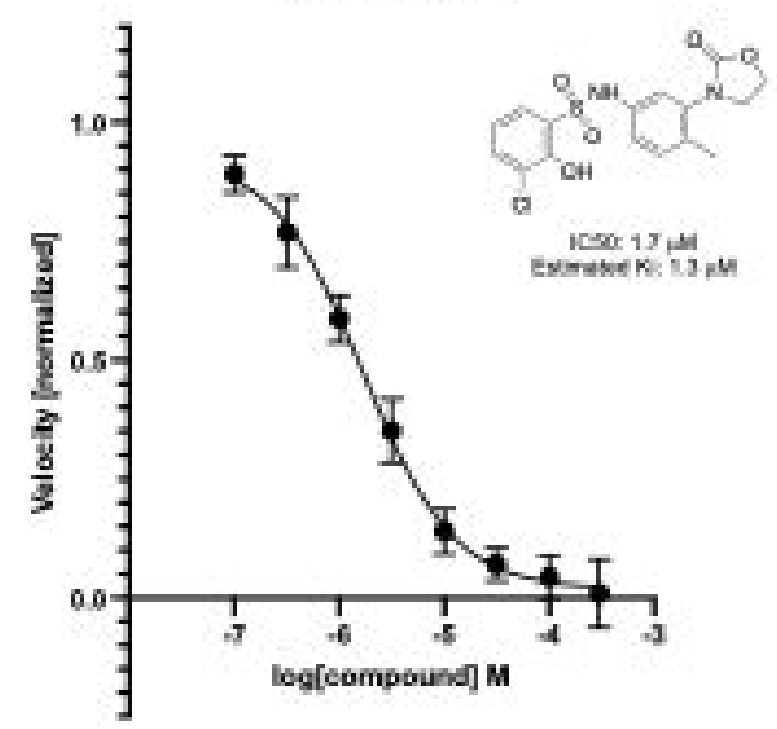

Z9544921833

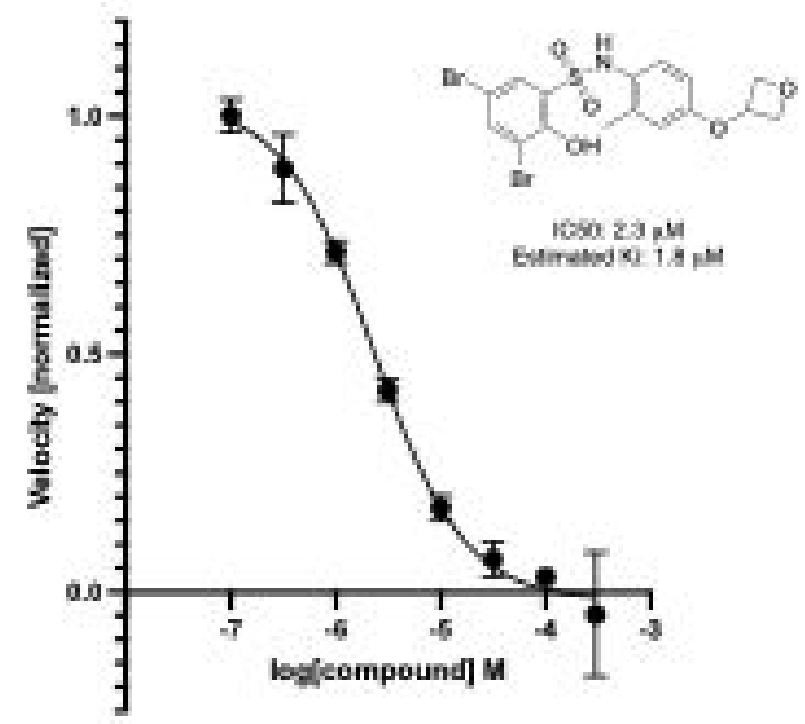

Z9103952180

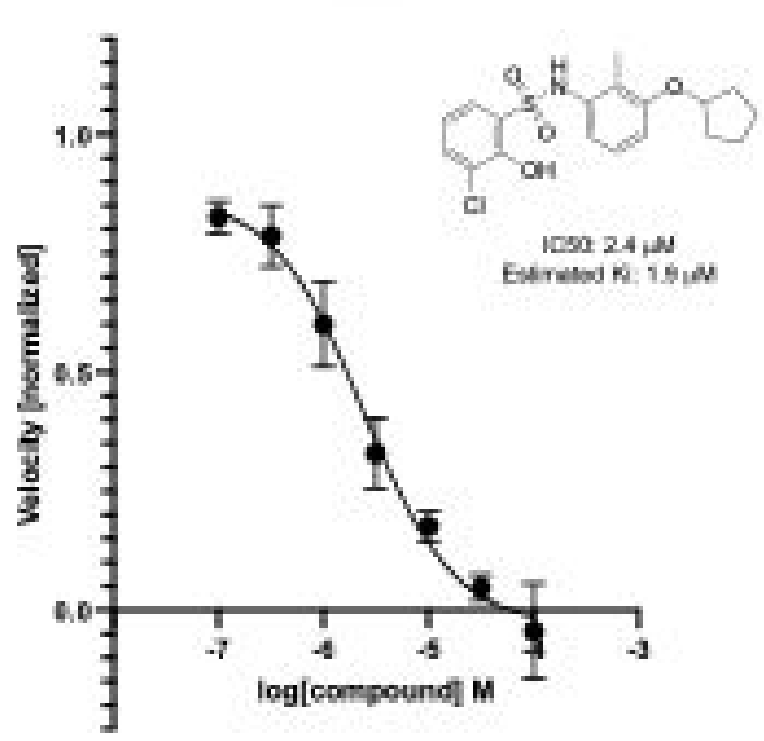

Z9103952171

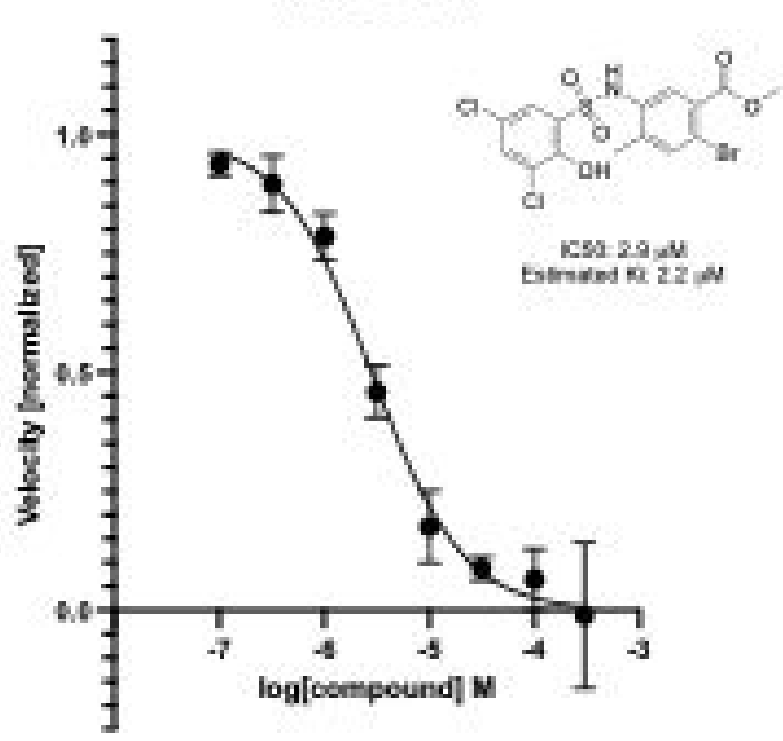

Z2940310451

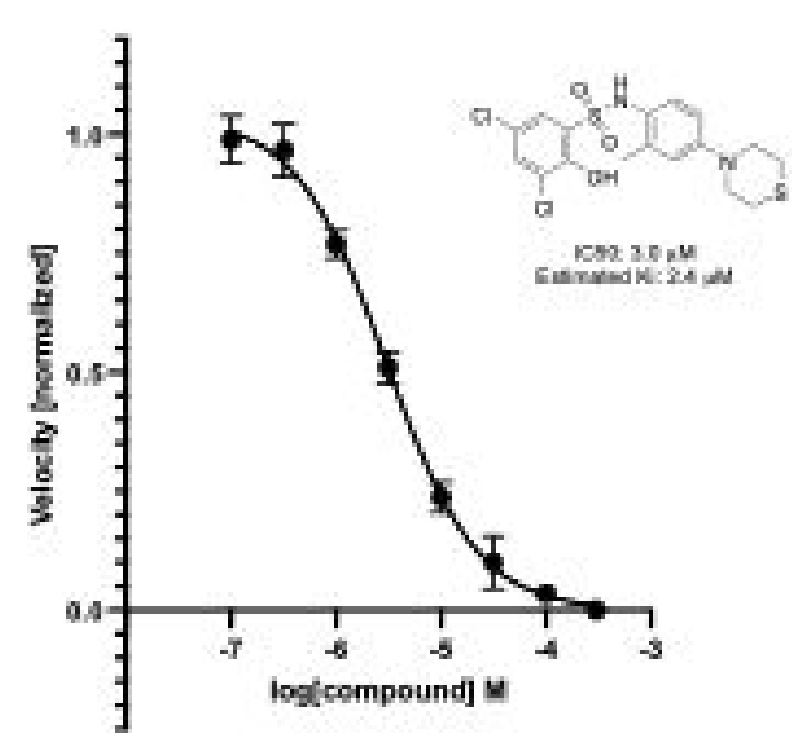

Z9464814853

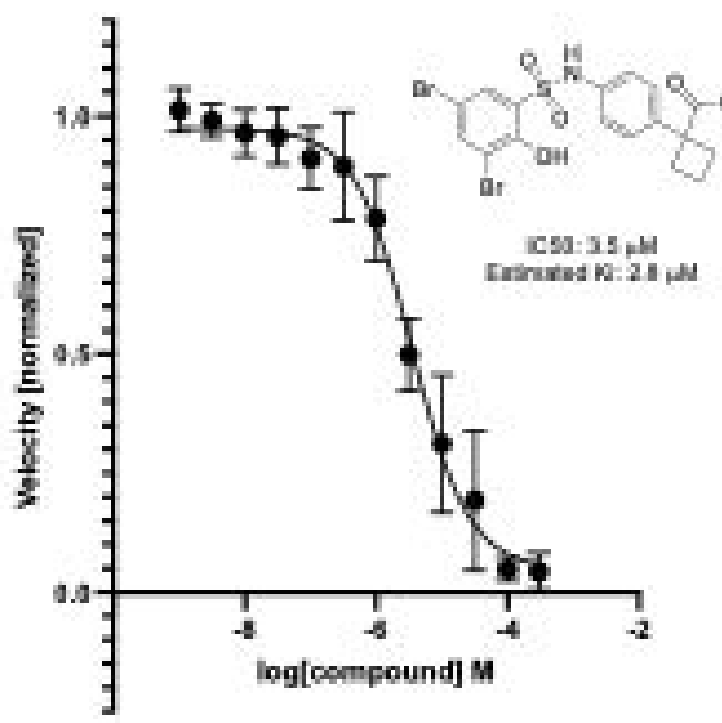

Z9103952313

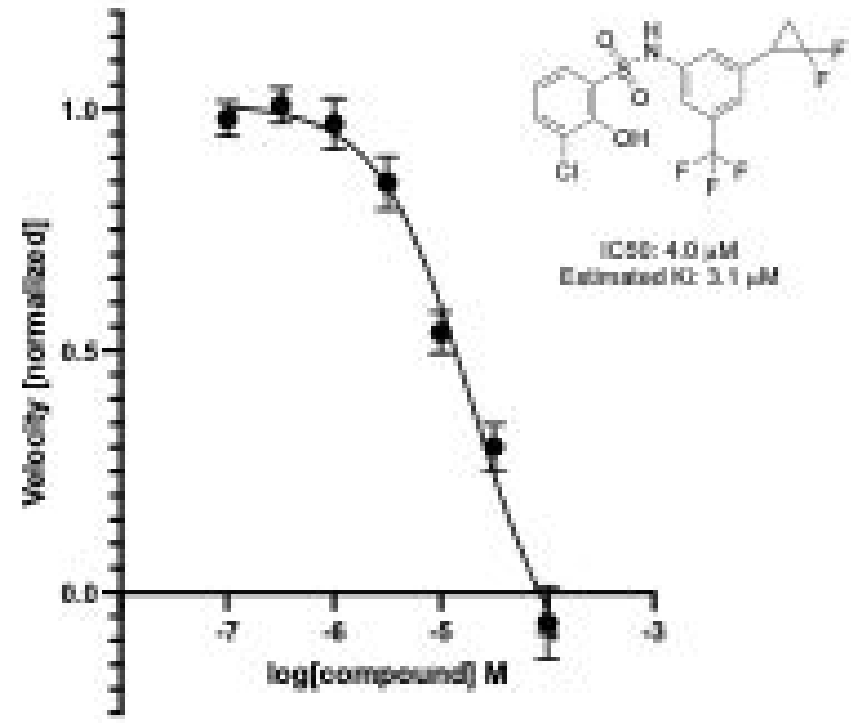

Z8238930017

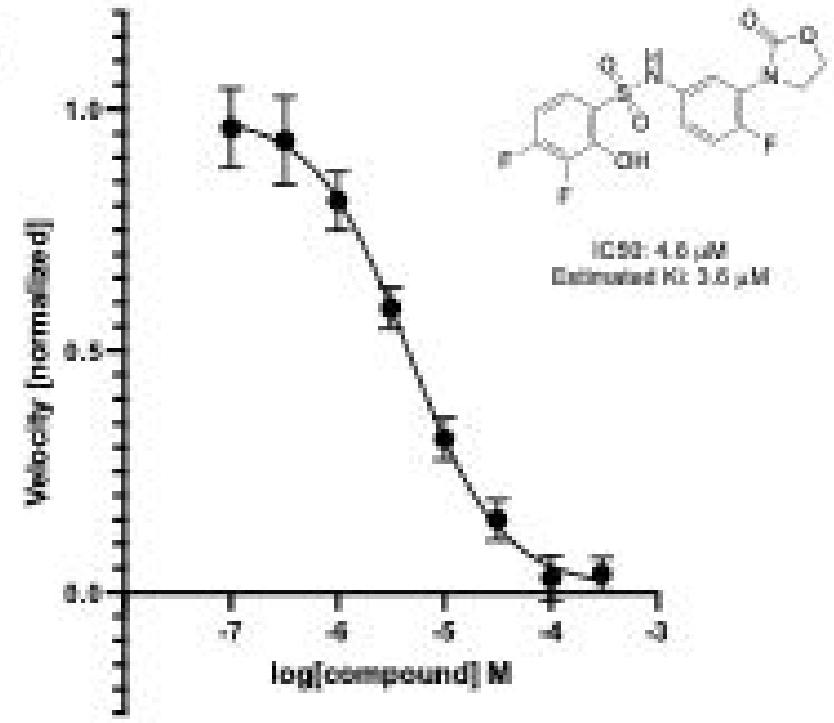

Z9544921957

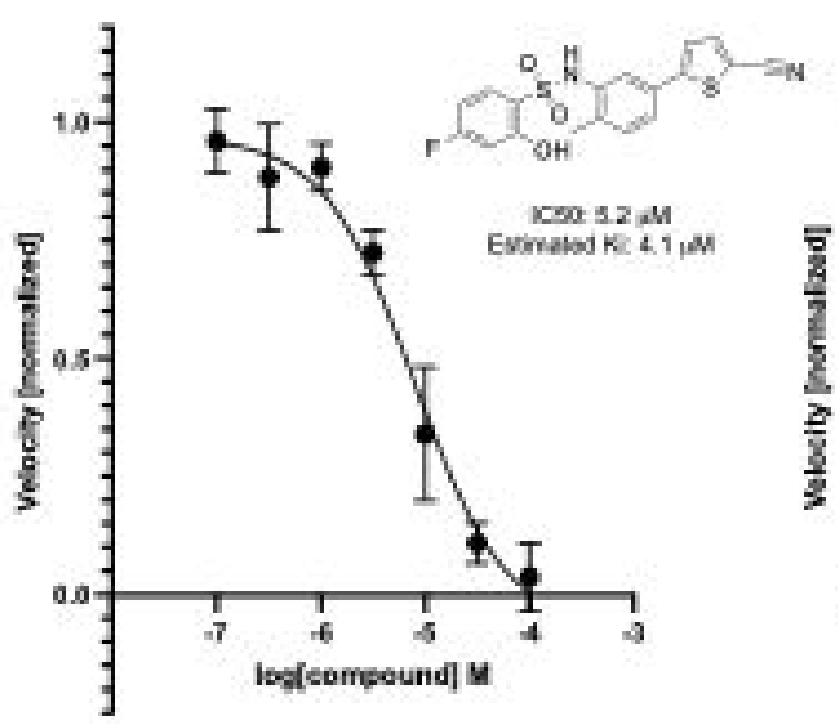

Z9544922043

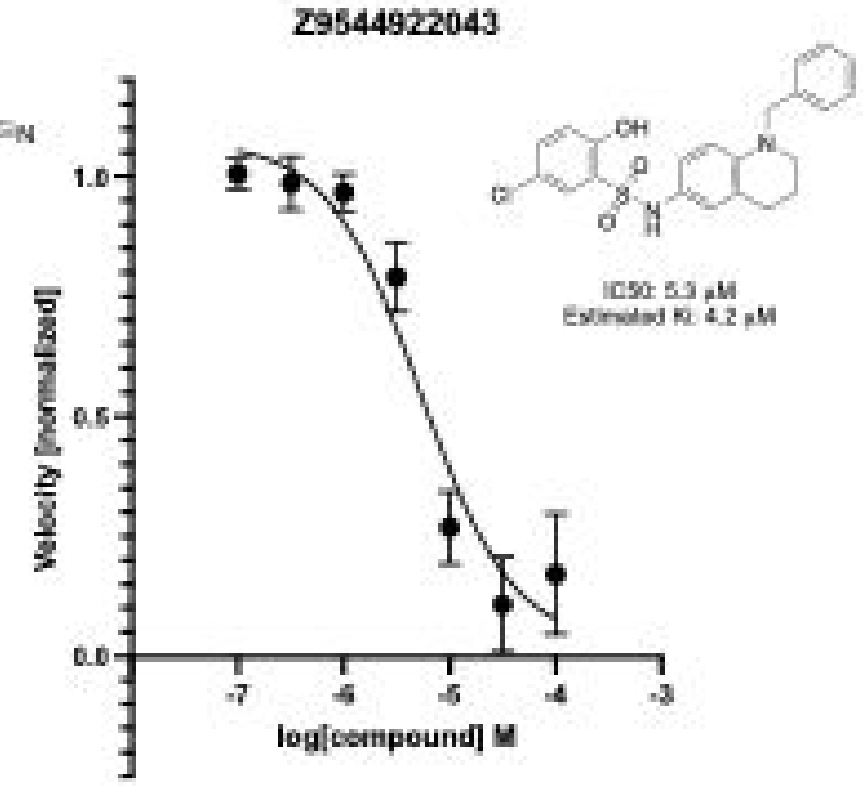

Z9103951892

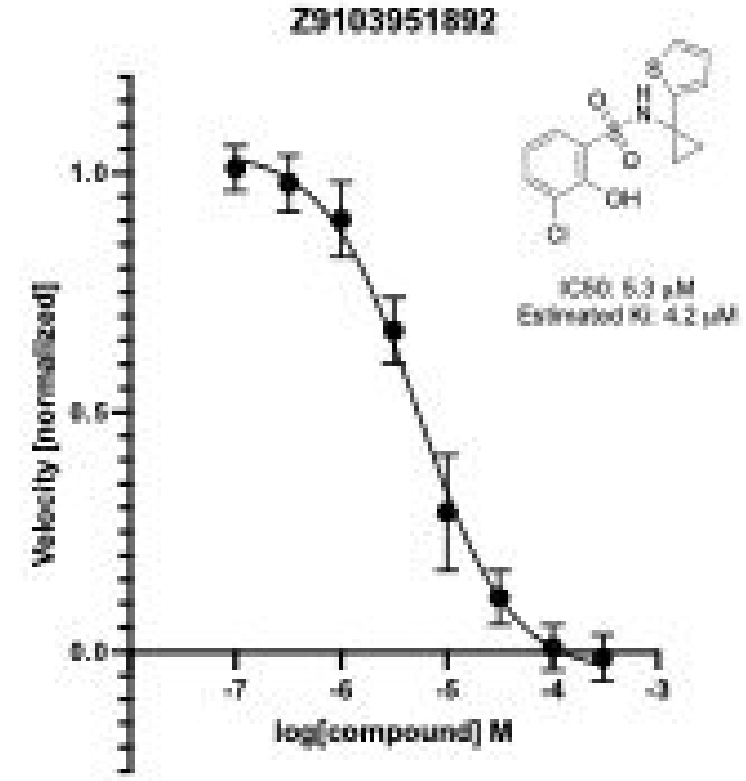

Z9544921868

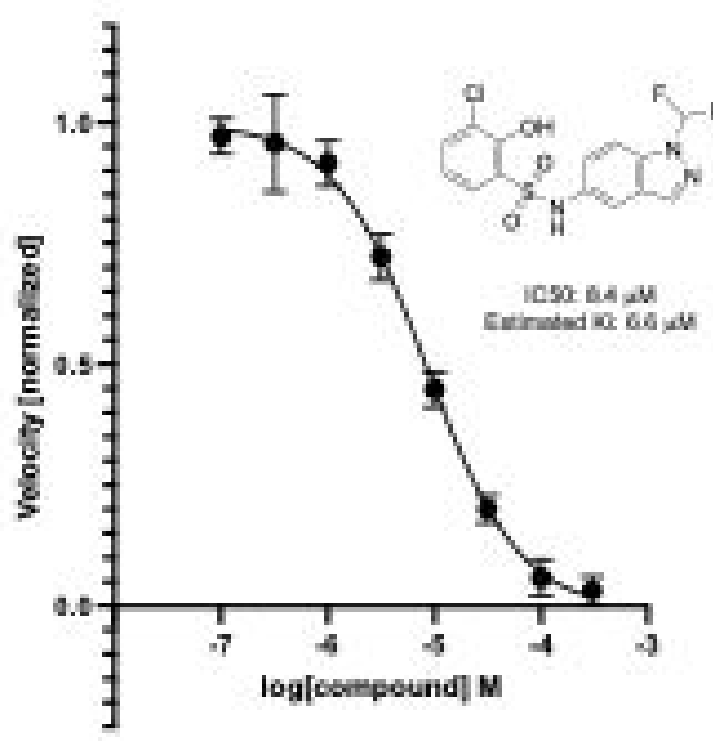

Z9544922503

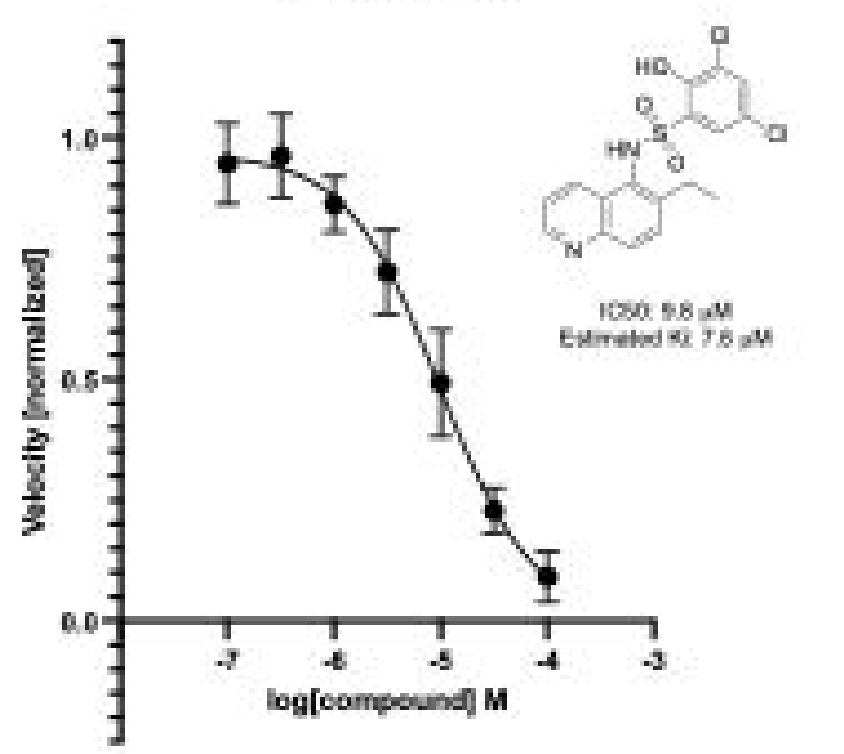

Z9103952053

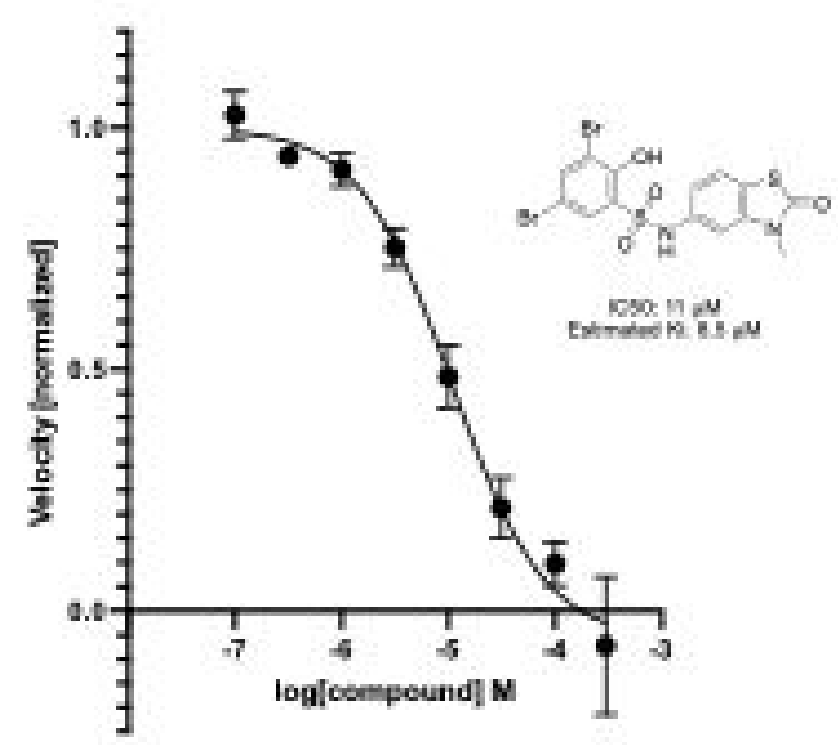

Z9544922287

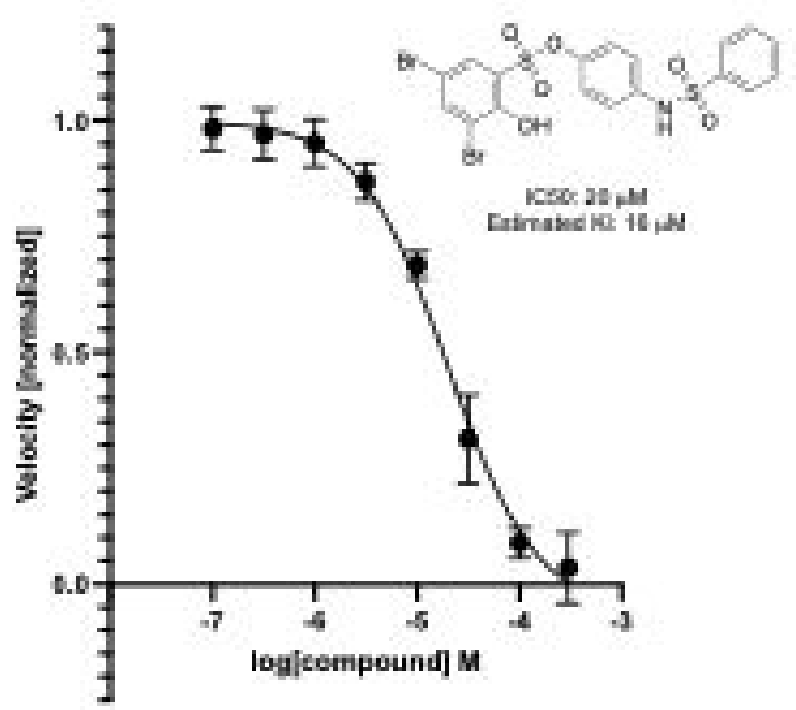

Z2940604882

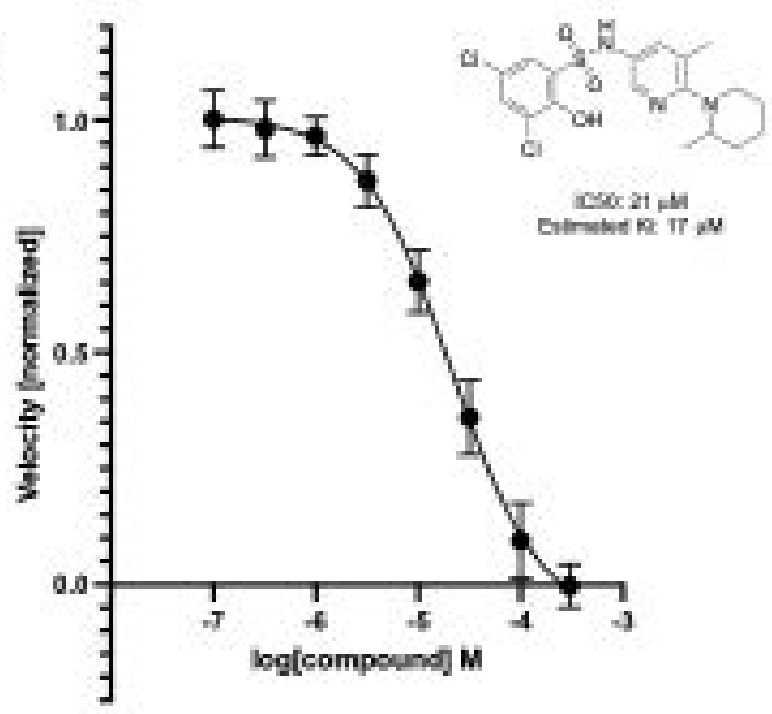

Z9103951943

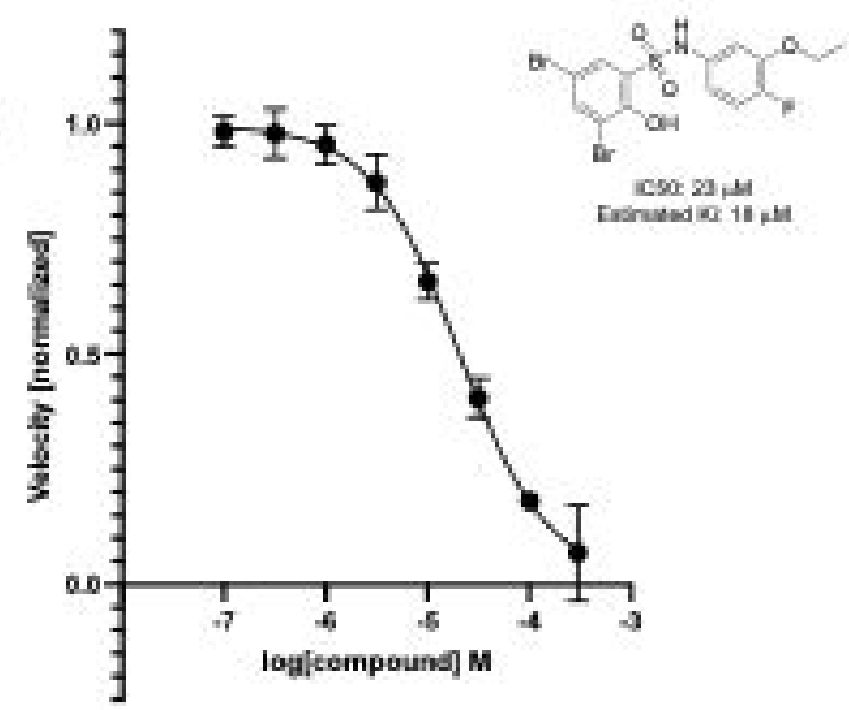

Z9544922018

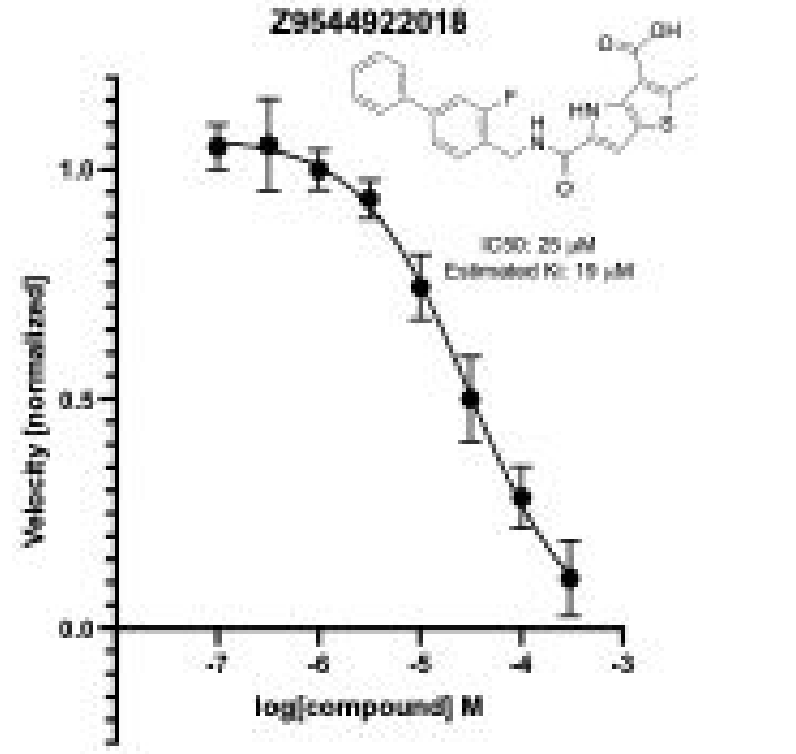

Z9103952375

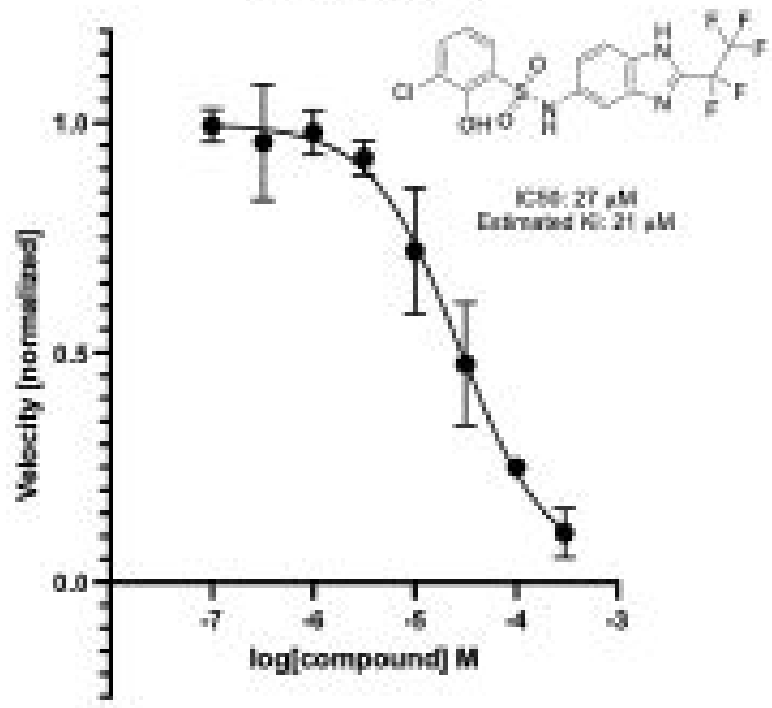

Z9464814798

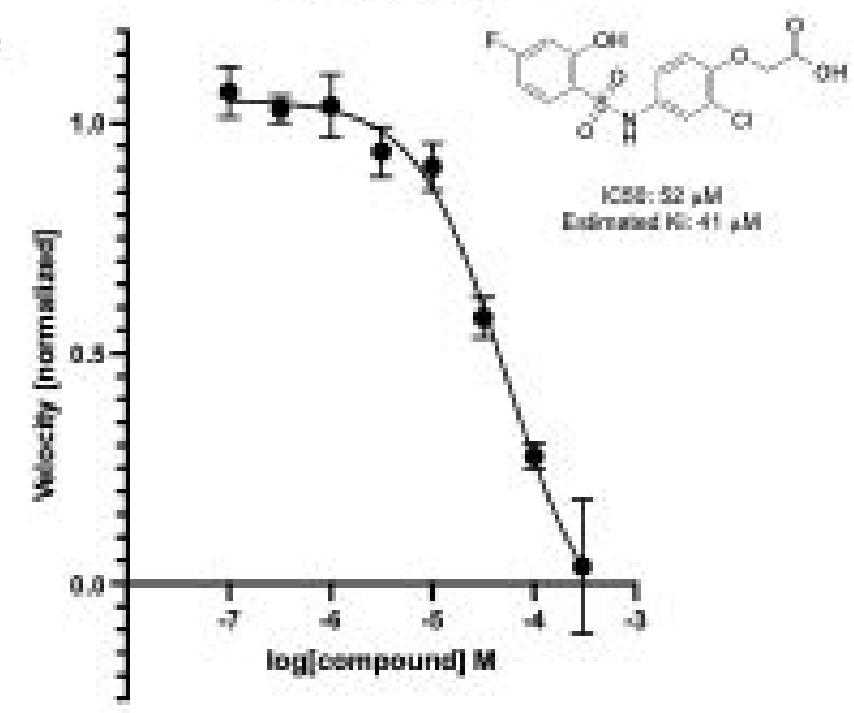

Z9544922144

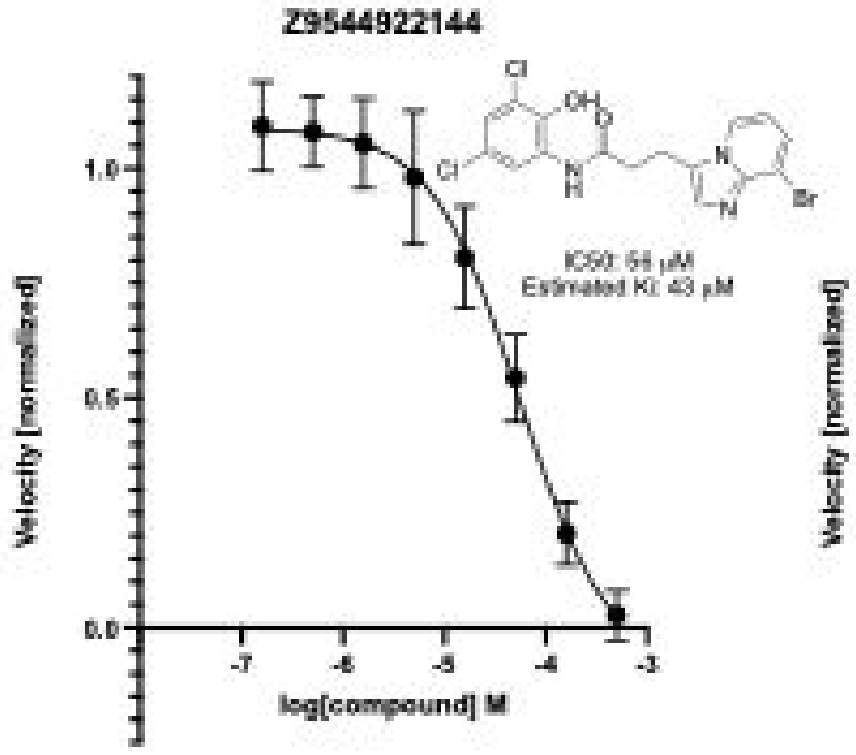

Z8278108755

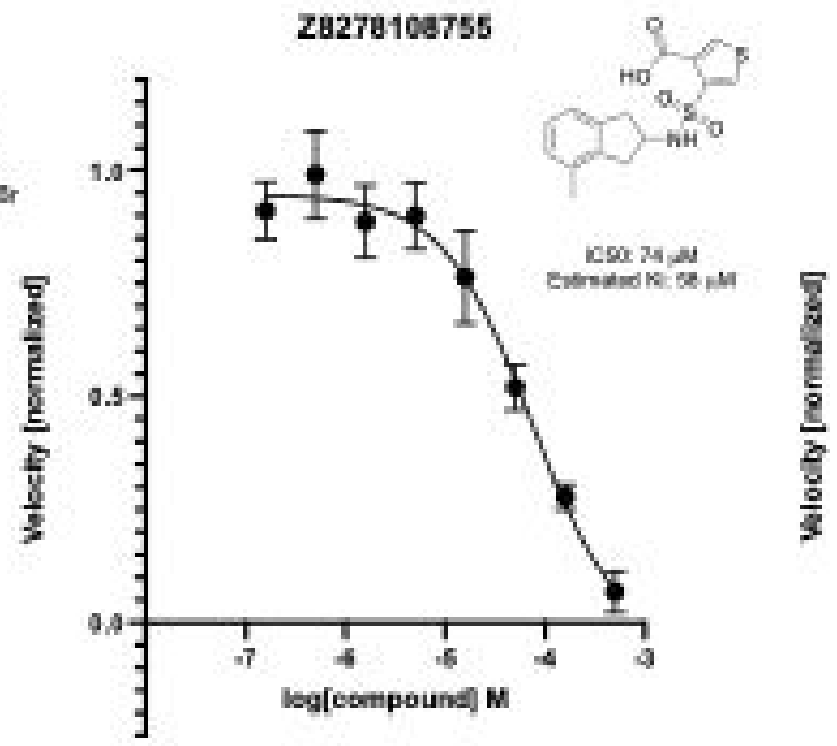

Z9544922113

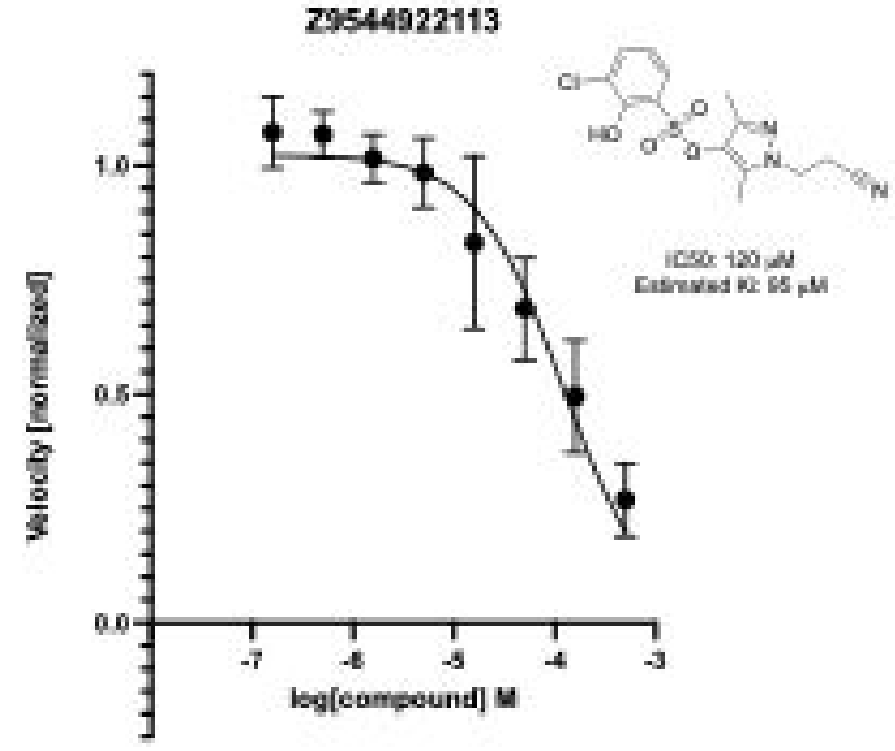

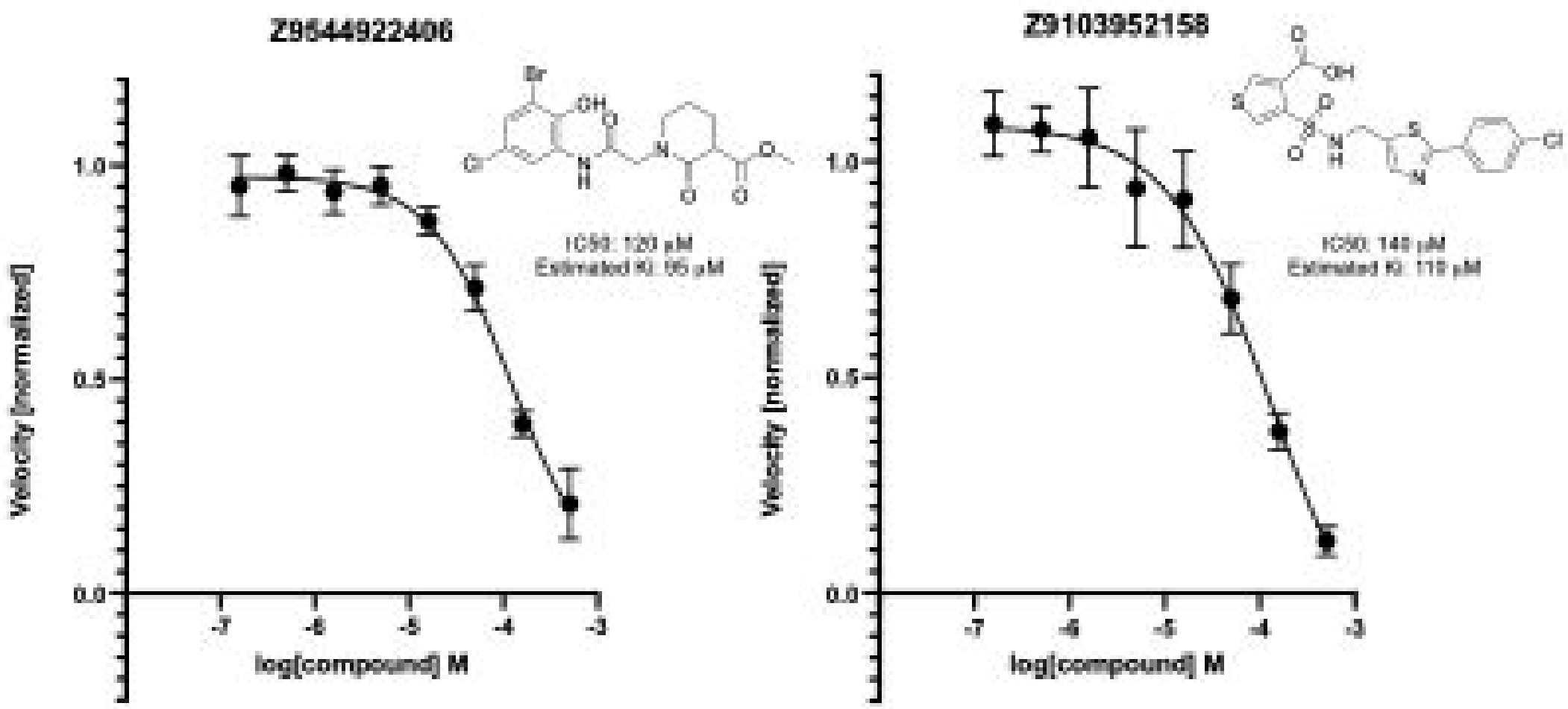

Figure S5. Full concentration response curves for 28 novel compounds. Velocity is normalized to a negative control with no inhibitor present (see Methods).

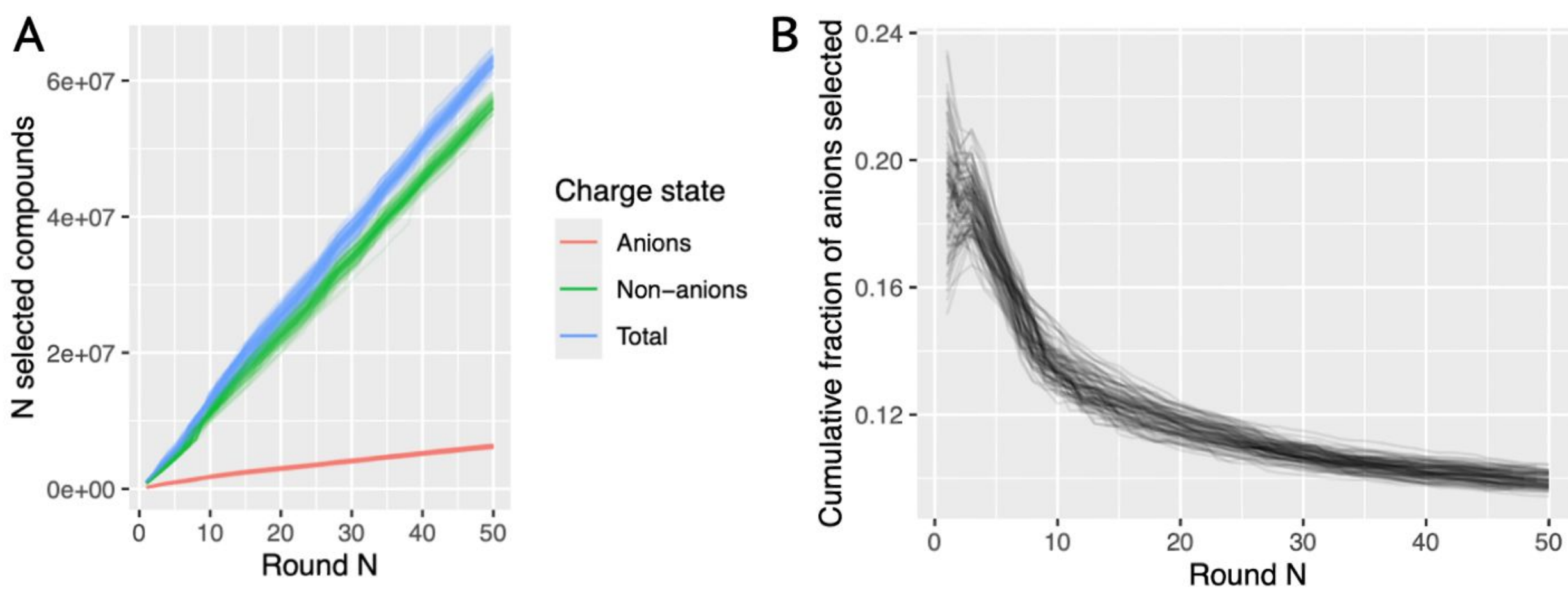

Figure S6. Numbers of anionic and di-anionic (“anions”) compounds prioritized by ChemSTEP versus numbers of non-anionic compounds prioritized. The cumulative fraction of anions prioritized is reported in B). The background proportion of anions in the library is 2.7%, such that over the full run ChemSTEP enriches anions by less than 4-fold.

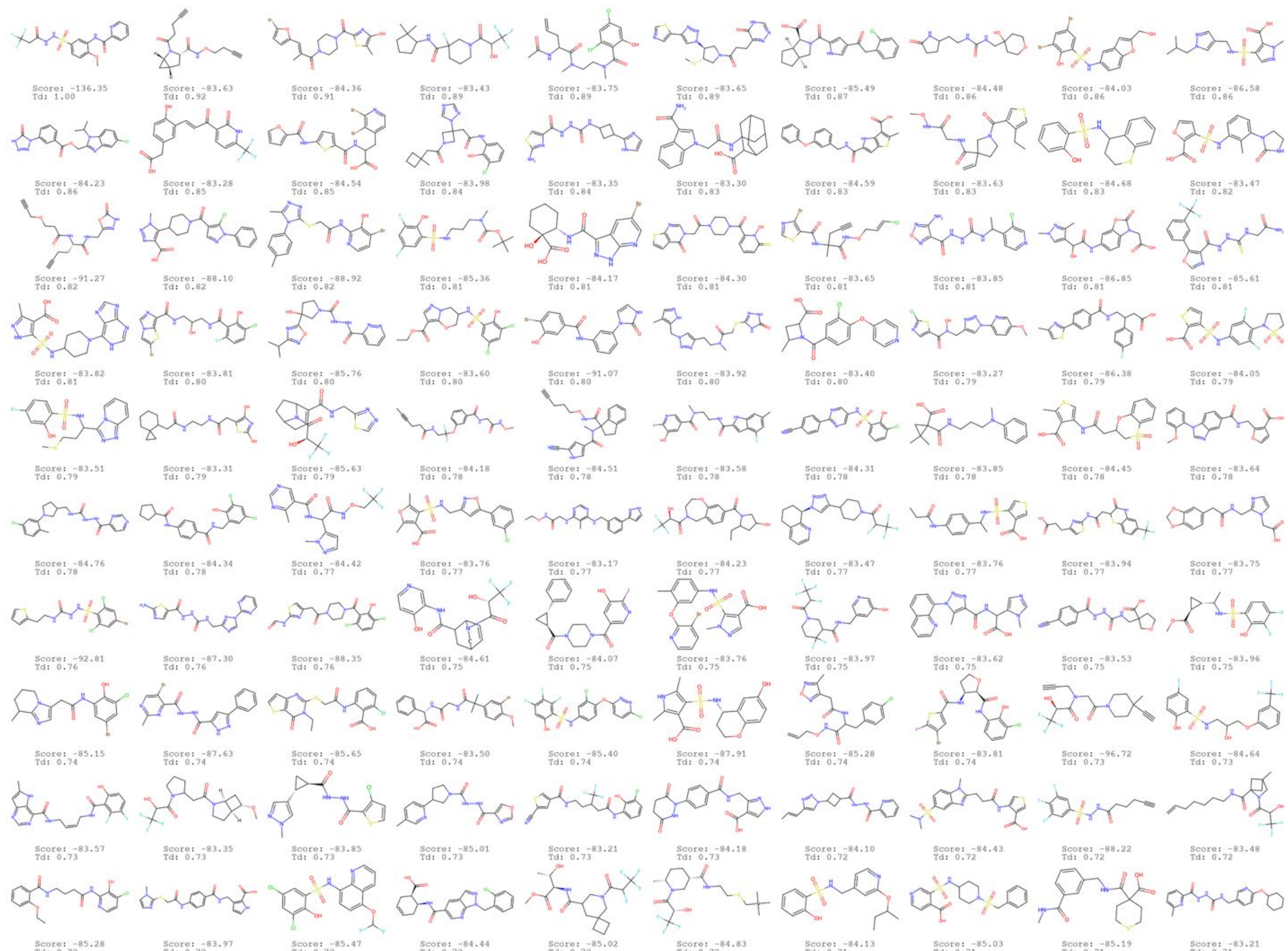

Figure S7. Initial 100 (R1) beacons selected in the first replicate run of ChemSTEP on AmpC β-lactamase. DOCK score and maximum Tanimoto distance to the closest previously assigned beacon are shown.

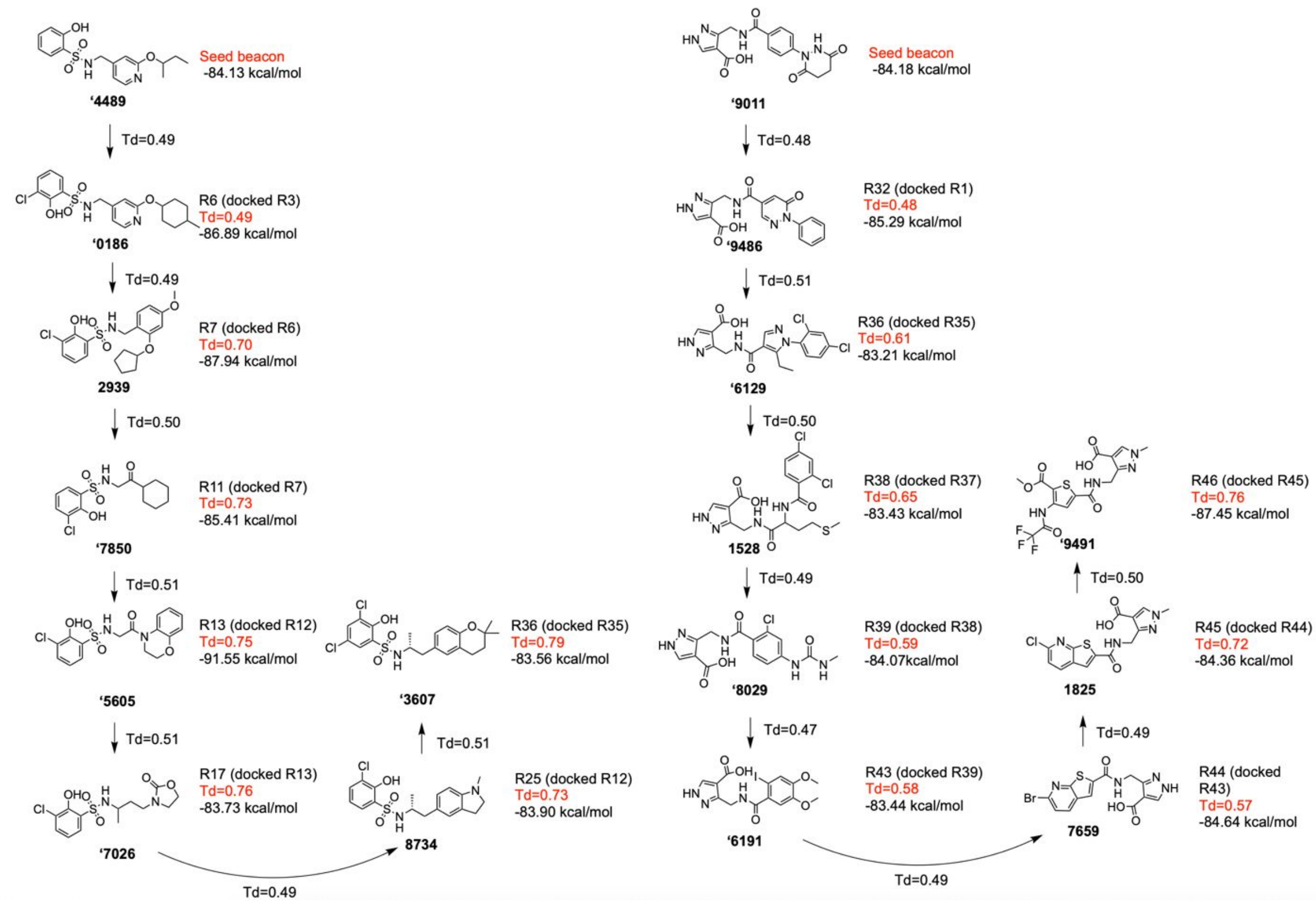

Figure S8. Additional sequences of beacon selections from the first replicate run of ChemSTEP on AmpC β-lactamase.

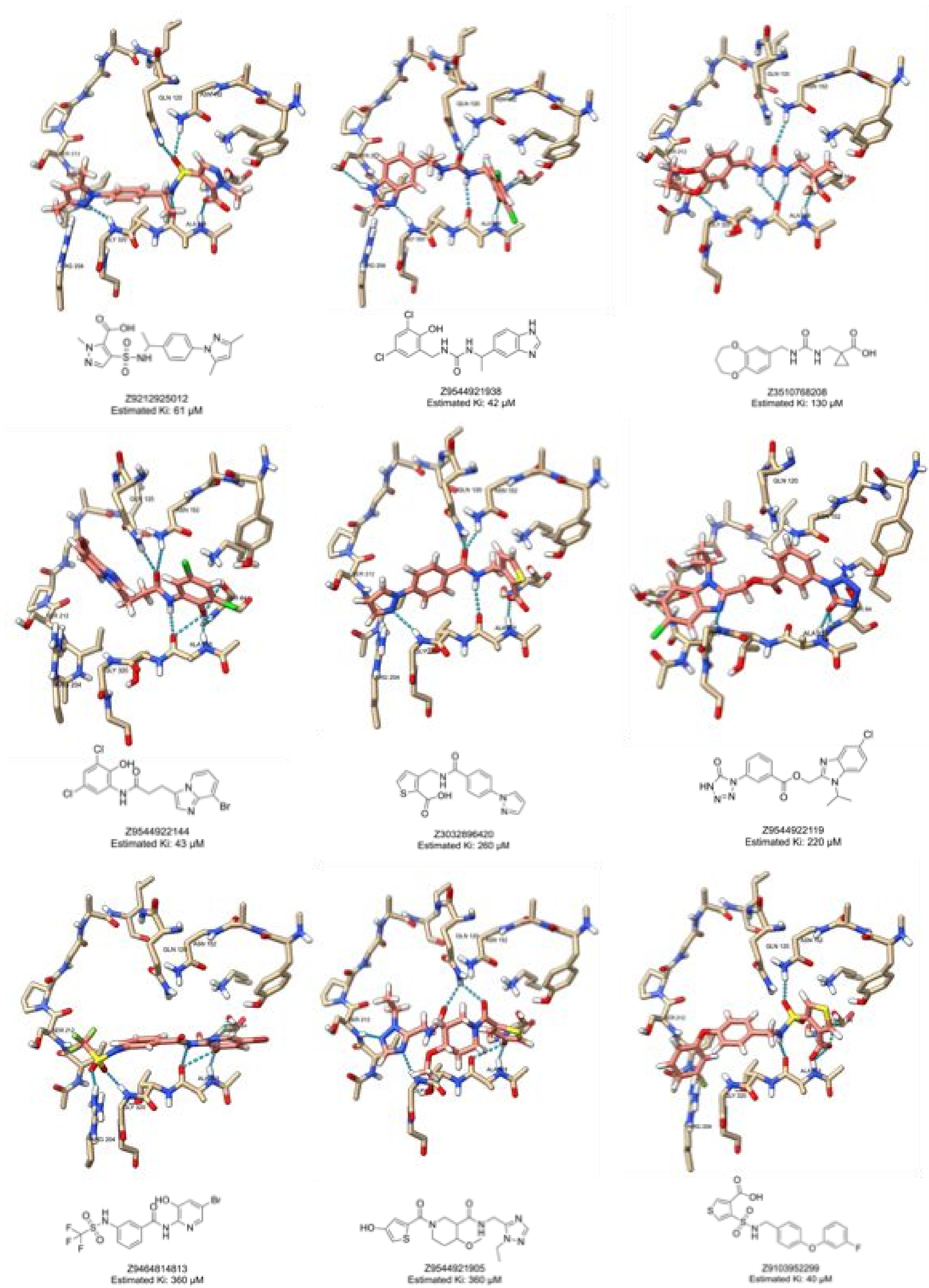

Figure S9. Examples of experimental actives with atypical chemotypes. AmpC is represented in beige, with docked ligands shown in pink with hydrogen bonds shown.

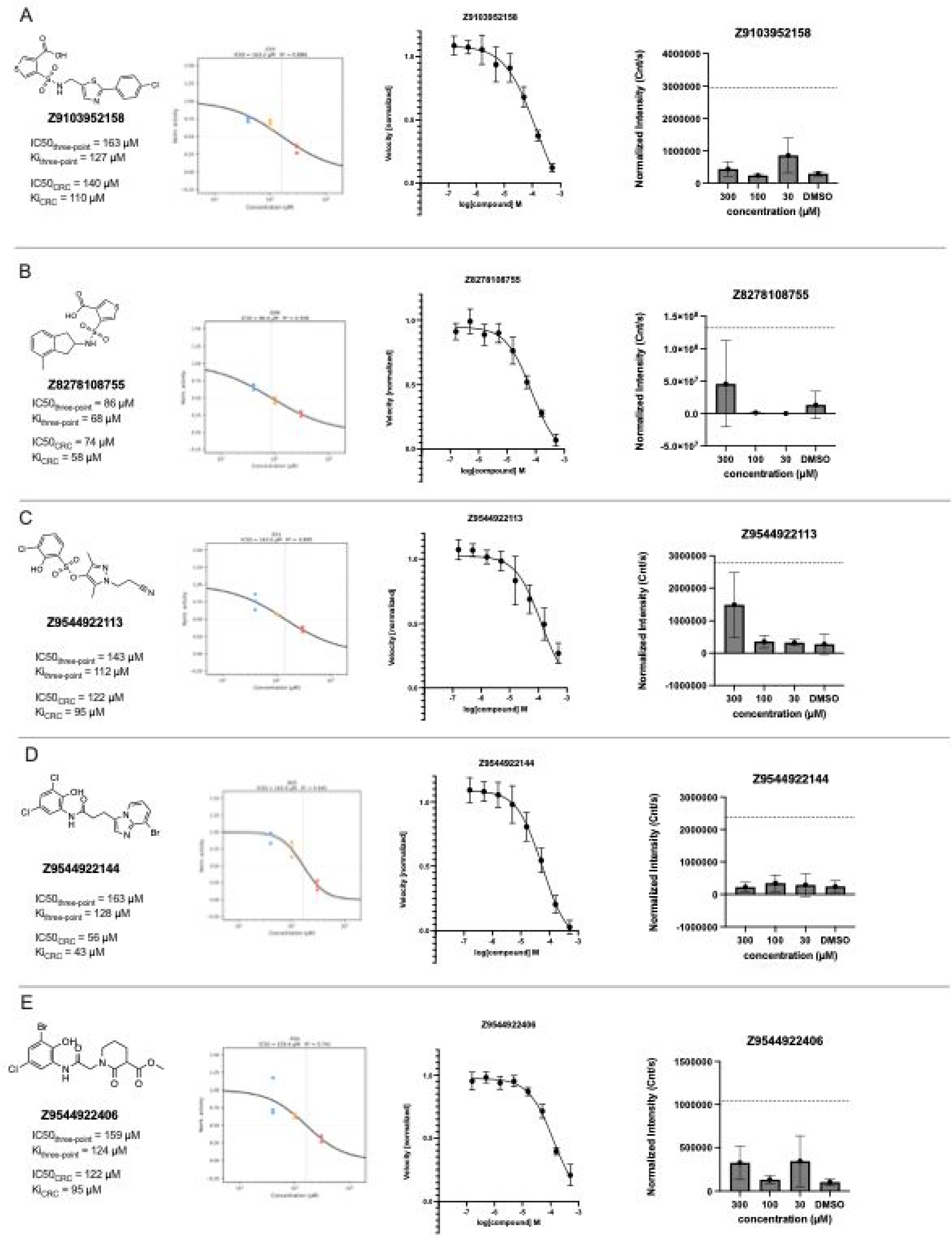

Figure S10. 2D structures, initial three-point curve fits with associated IC50 and Ki calculations (see Methods), full concentration response curves with associated IC50 and Ki calculations, and dynamic light scattering (DLS) data at 300, 100, and 30 µM for five medium-affinity experimental hits. Dotted line represents 10 times the DMSO control scattering count.

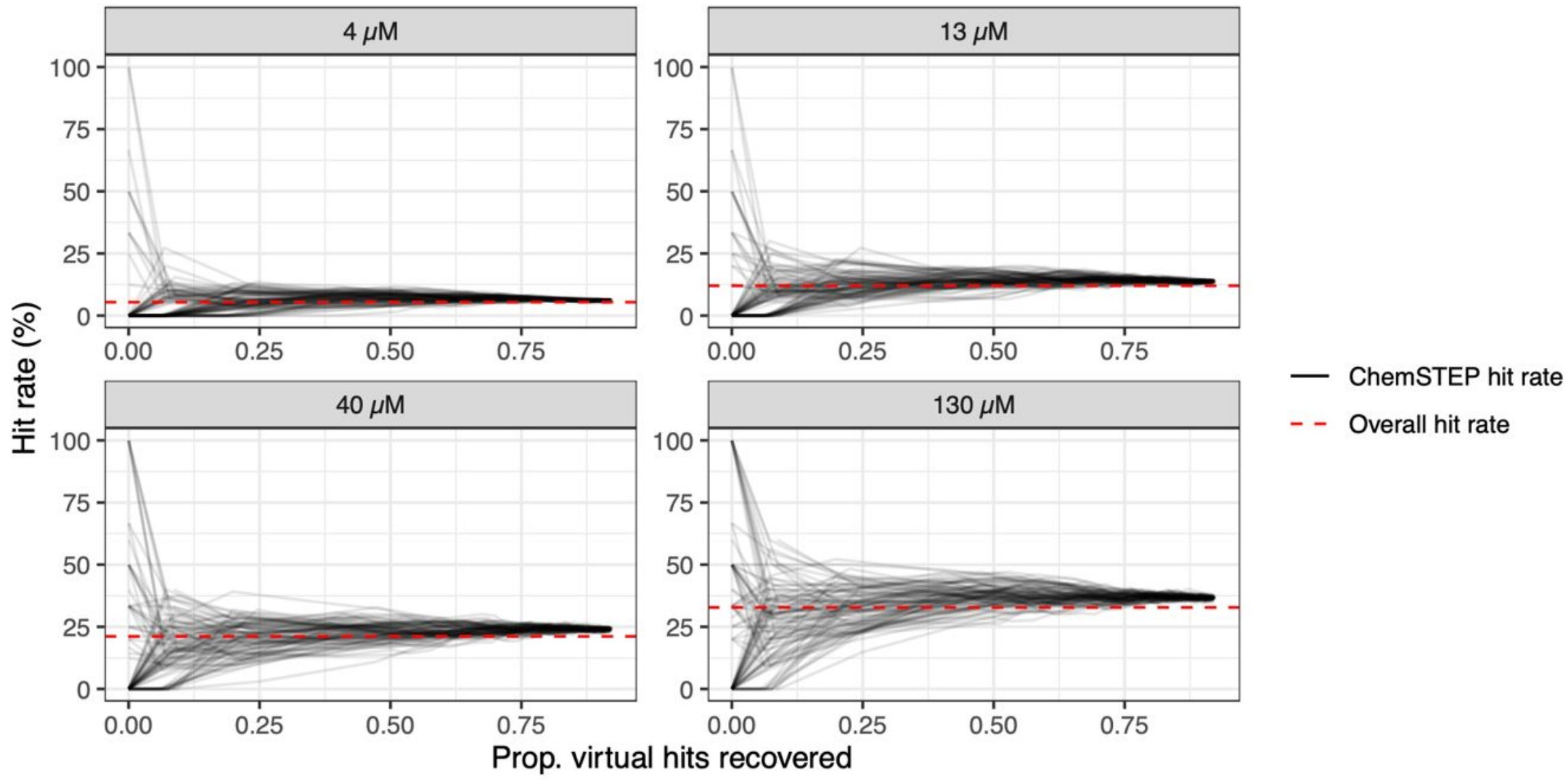

Figure S11. Full curves for early stopping hit rates**.** Same as Figure 9, without averaging.

Table S1. Inhibitors from the top 187-ranked molecules with Ki values 13 uM and under, compared to the most-similar compound from the brute-force 1.7B campaign, with Tc values (Morgan R=2), previously reported Ki values, and the number of molecules in the total 13.2B space that are more similar to the reference ligand than the corresponding new hit.

Compound, Ki	Closest known (Liu et al), Tc, Ki	N Molecules more similar
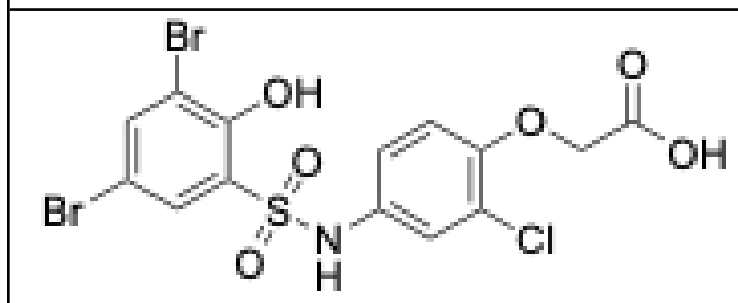 Z9464814846, 220 nM	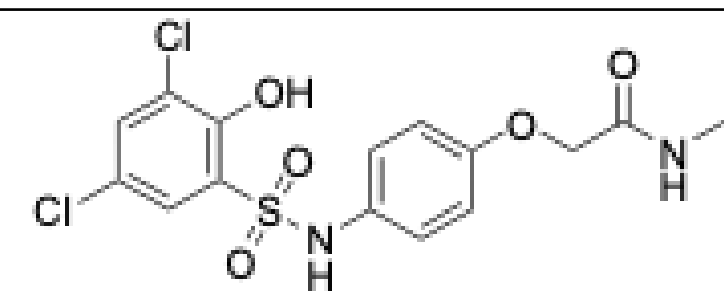 ZINCpw0000078zSl, Tc=0.48, 42 uM	7359
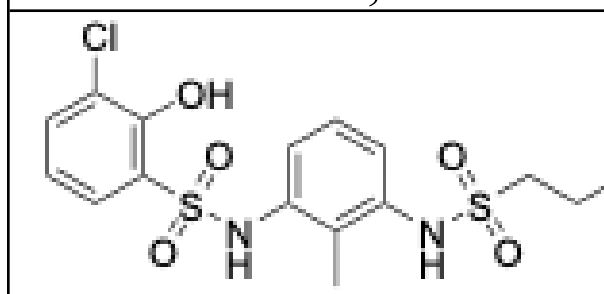 Z9212924993, 430 nM	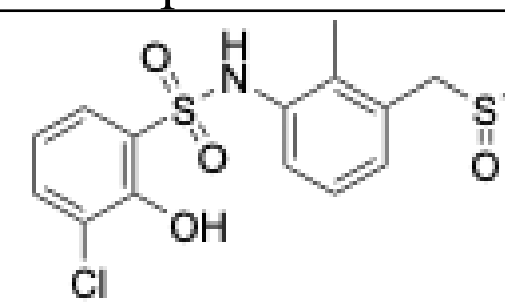 ZINCnA000006ySao, Tc=0.65, 980 nM	133
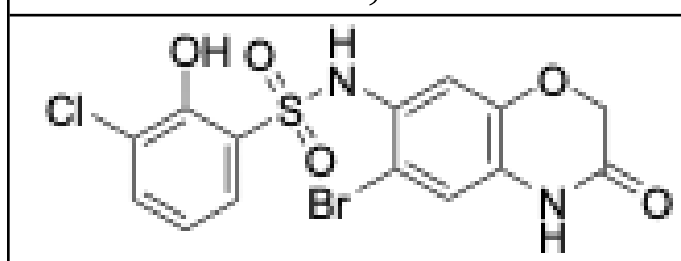 Z9544921818, 430 nM	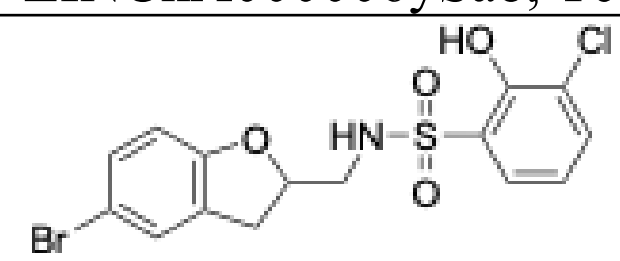 ZINCnA000006CqwN, Tc=0.44, 40 uM	55869
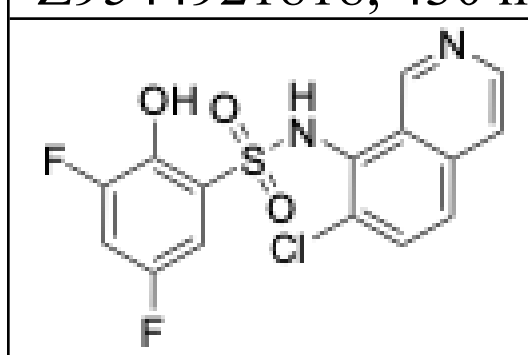 Z9103952047, 1.3 uM	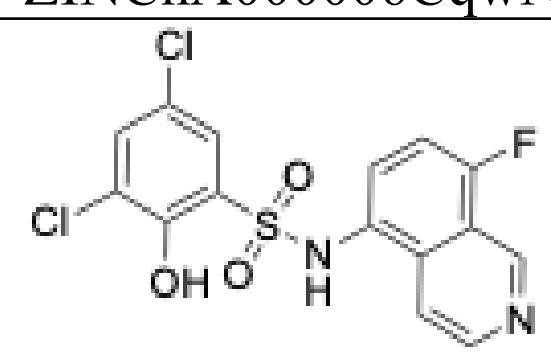 ZINCoL00000dWJgJ, Tc=0.53, 1.3 uM	2494
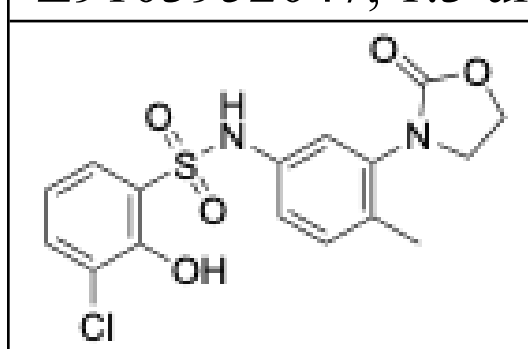 Z9212924971, 1.3 uM	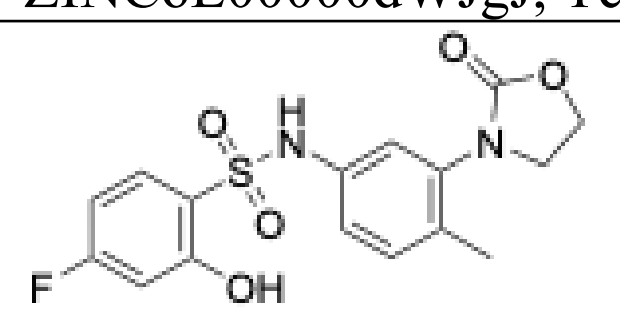 ZINCpv0000009ARJ, Tc=0.67, 14 uM	98
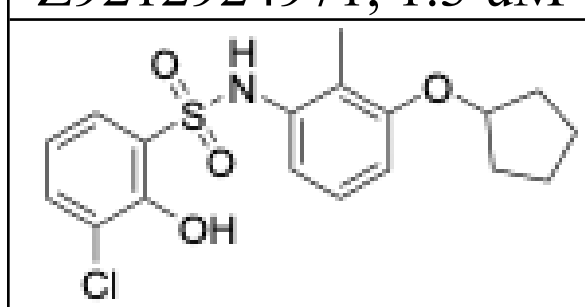 Z9103952180, 1.9 uM	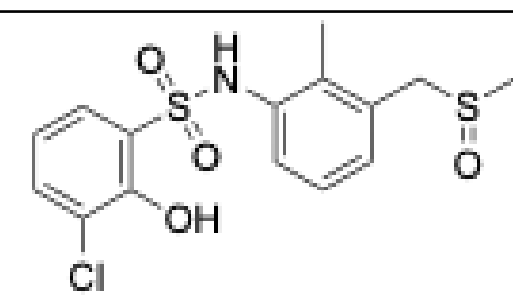 ZINCnA000006ySao, Tc=0.53, 980 nM	1753
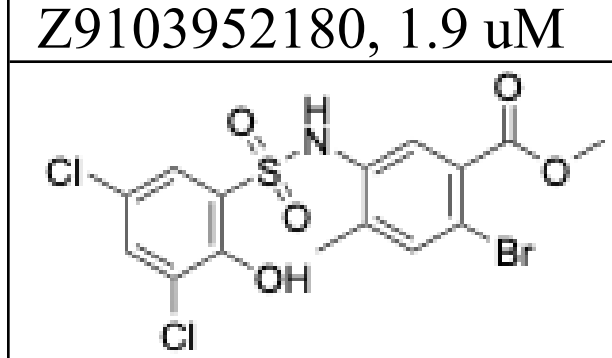 Z9103952171, 2.2 uM	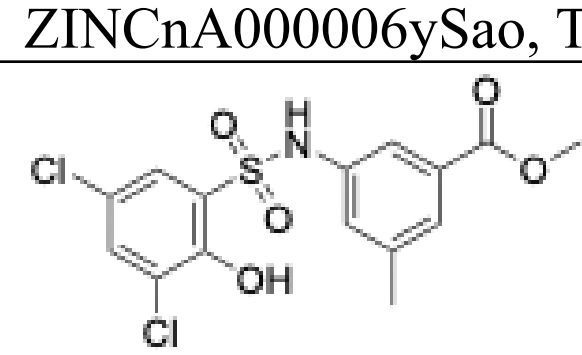 ZINCoF000003N4Sx, Tc=0.57, 13 uM	931
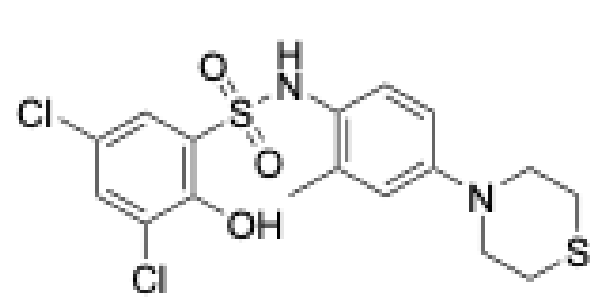 Z2940310451, 2.4 uM	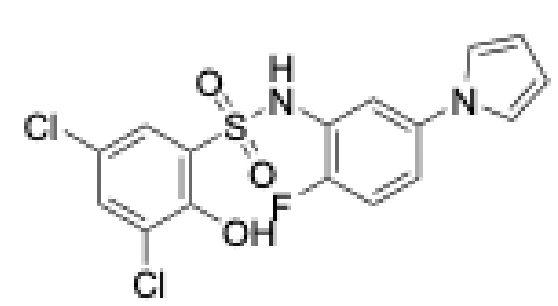 ZINCpO00000deSUE, Tc=0.50, 19 uM	3037

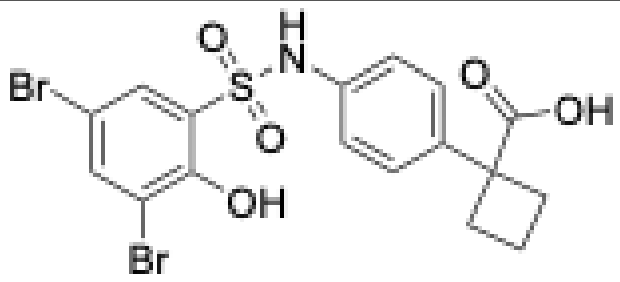<br>Z9464814853, 2.8 uM	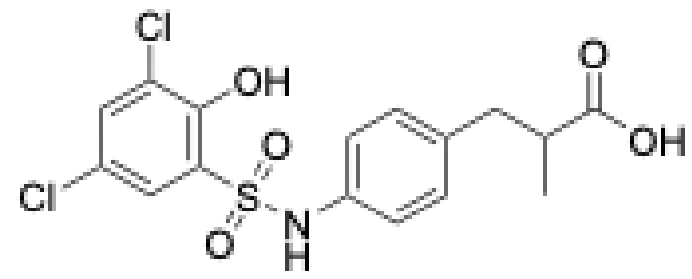<br>ZINCpH000006mtN0, Tc=0.34, 6.4 uM	390007
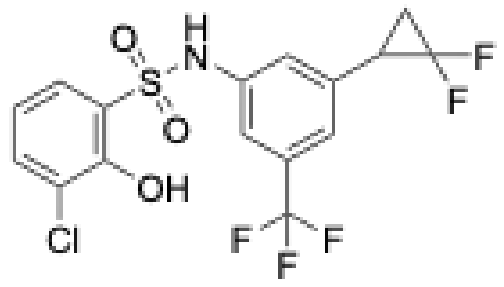<br>Z9103952313, 3.1 uM	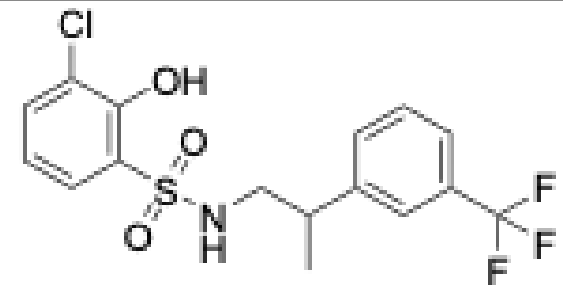<br>ZINCpL00000ezaXQ, Tc=0.52, 300 uM	6593
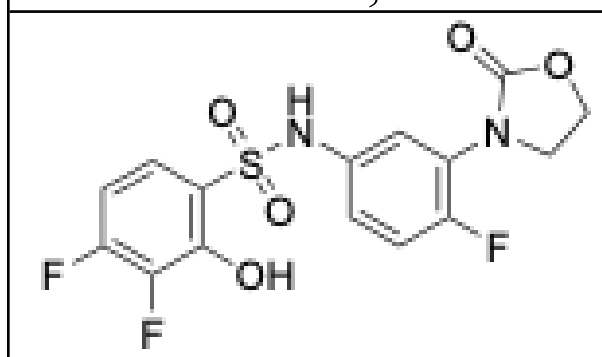<br>Z8238930017, 3.6 uM	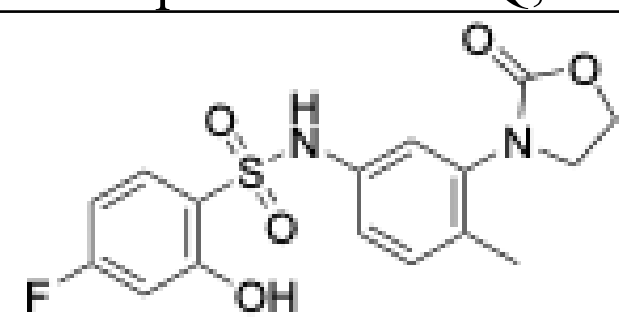<br>ZINCpv0000009ARJ, Tc=0.62, 14 uM	353
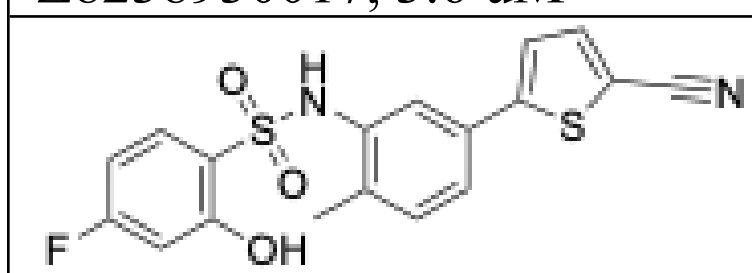<br>Z9544921957, 4.1 uM	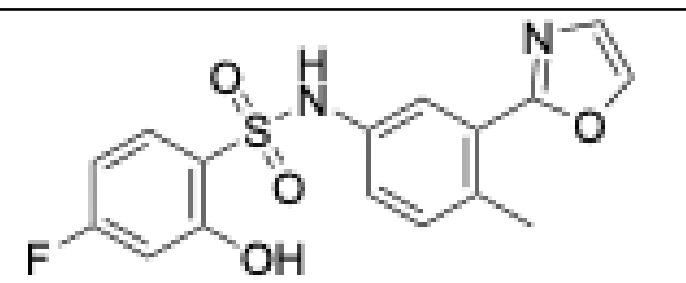<br>ZINCoC000000Odoq, Tc=0.41, 140 uM	48160
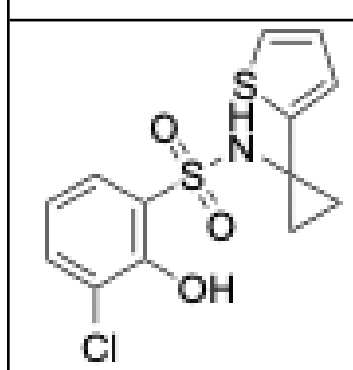<br>Z9103951892, 4.2 uM	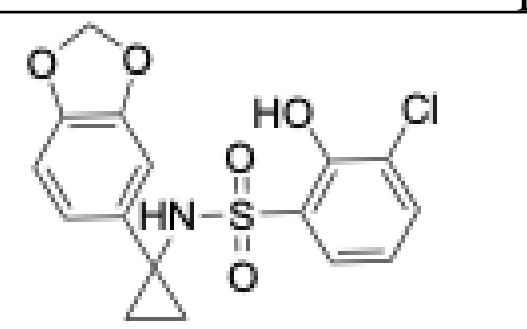<br>ZINCox000008O53Q, Tc=0.56, 26 uM	988
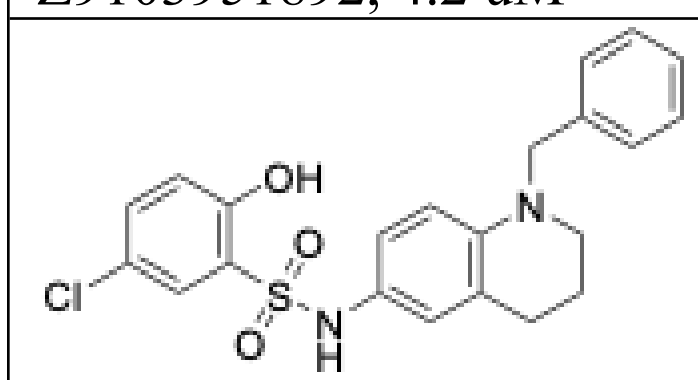<br>Z9544922043, 4.2 uM	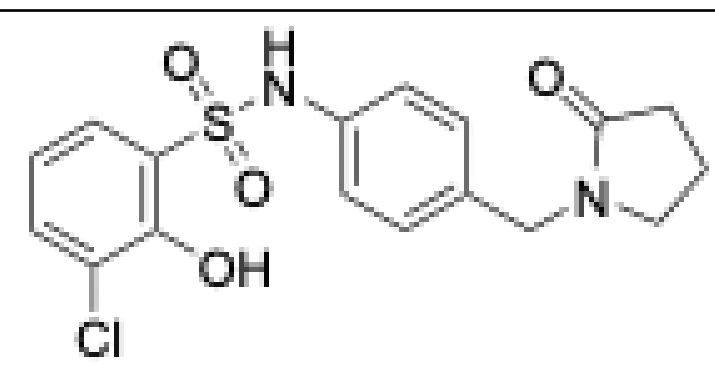<br>ZINCpz000008YxG6, Tc=0.47, 45 uM	15130
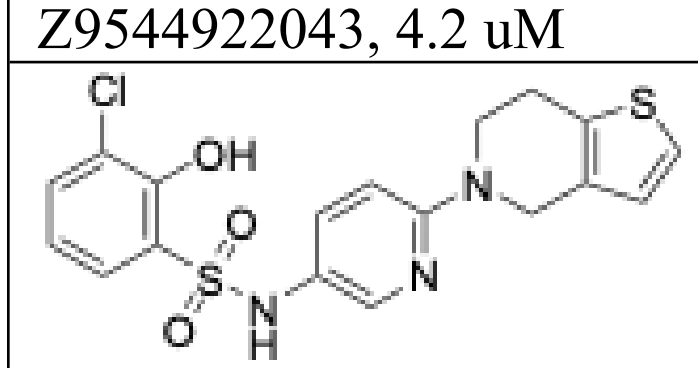<br>Z9544922358, 5.6 uM	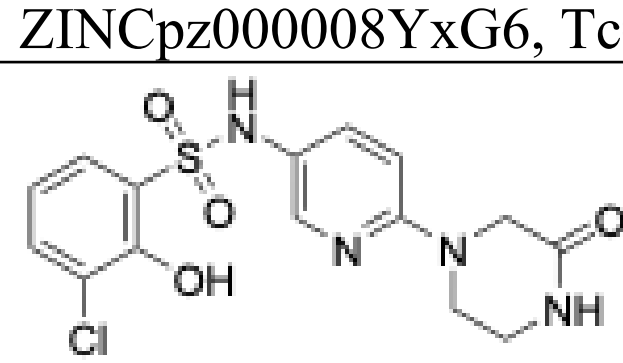<br>ZINCph00000FiMLj, Tc=0.67, 77 uM	113
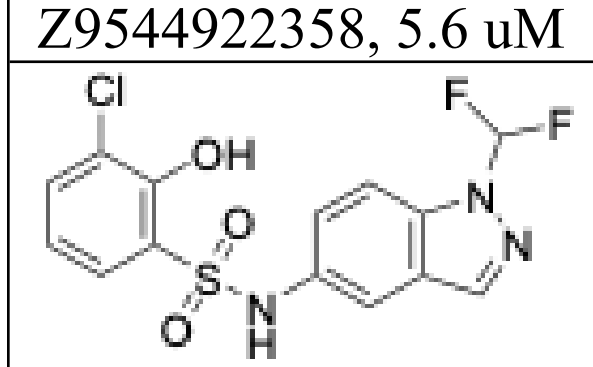<br>Z9544921868, 6.6 uM	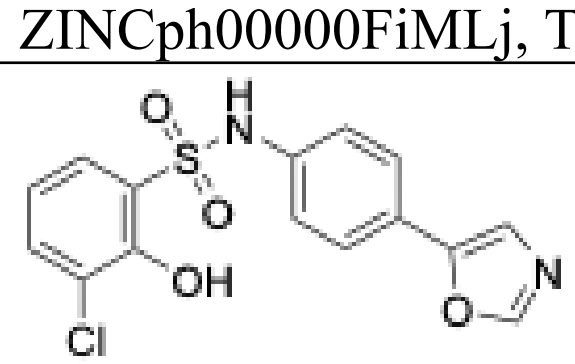<br>ZINCnF000007YVNA, Tc=0.47, 21 uM	4513
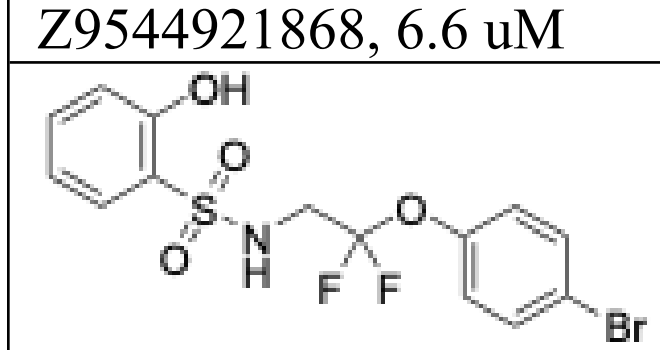<br>Z9544921807, 7.3 uM	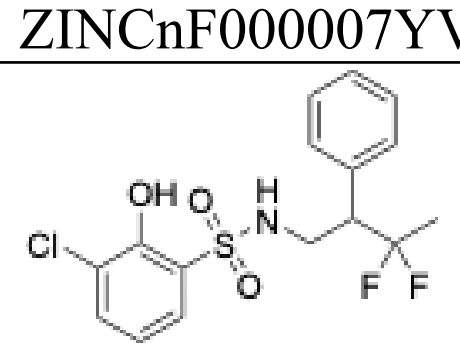<br>ZINCoH000005JAll, Tc=0.38, 120 uM	155159

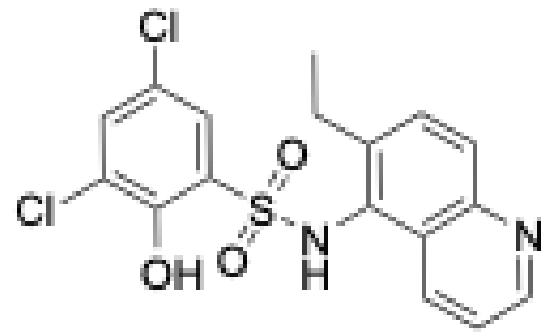 Z9544922503, 7.6 uM	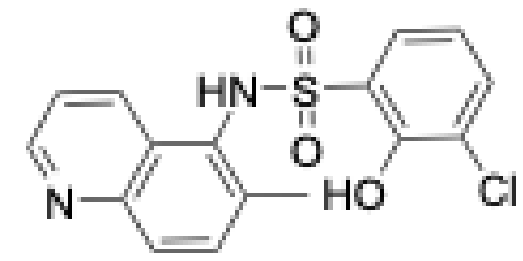 ZINCnH000008gL5D, Tc=0.57, 1.9 uM	666
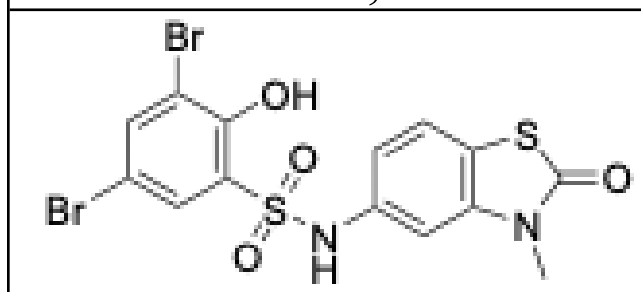 Z9103952053, 8.5 uM	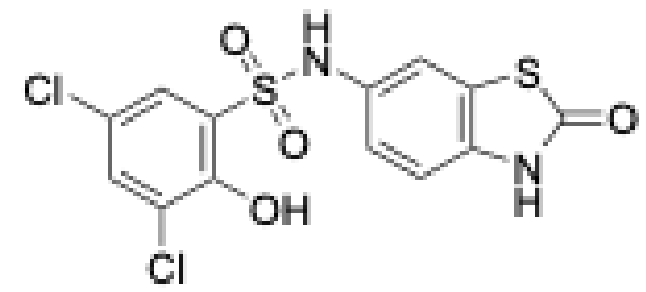 ZINCnE000005ursN, Tc=0.40, 11 uM	20481
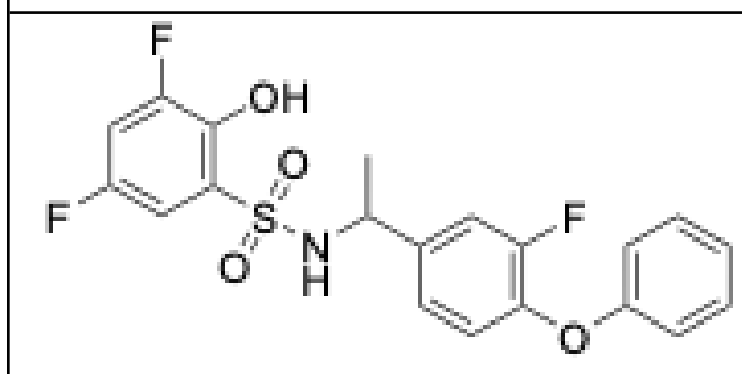 Z9544922369, 8.6 uM	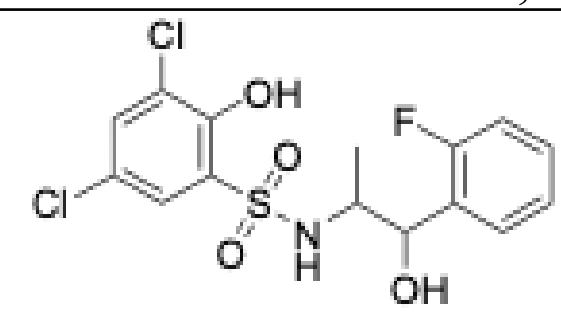 ZINCoC000005A0pc, Tc=0.38, 250 uM	100704
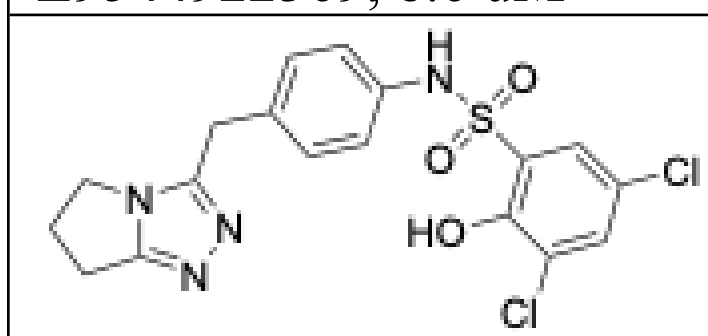 Z2940319985, 9.1 uM	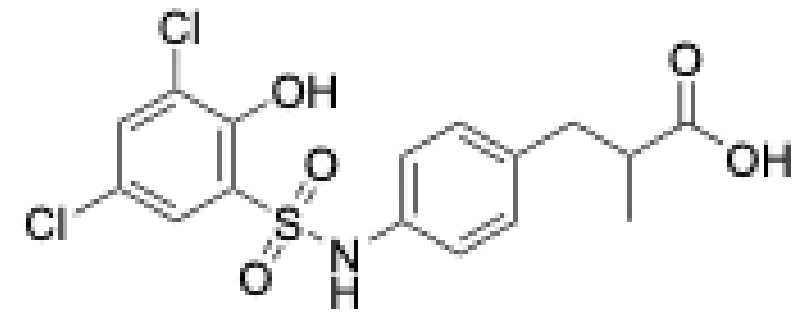 ZINCpH000006mtN0, Tc=0.50, 6.4 uM	3373
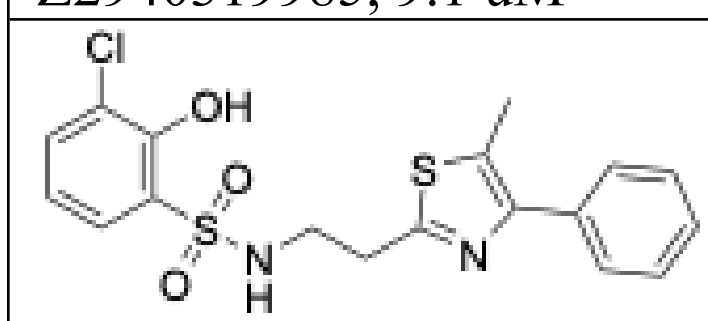 Z9544921729, 11 uM	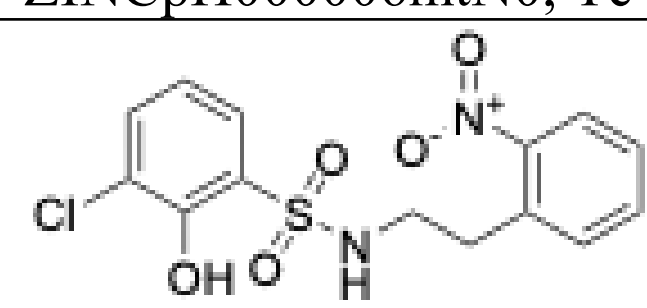 ZINCnu000009DLzG, Tc=0.50, 88 uM	12109
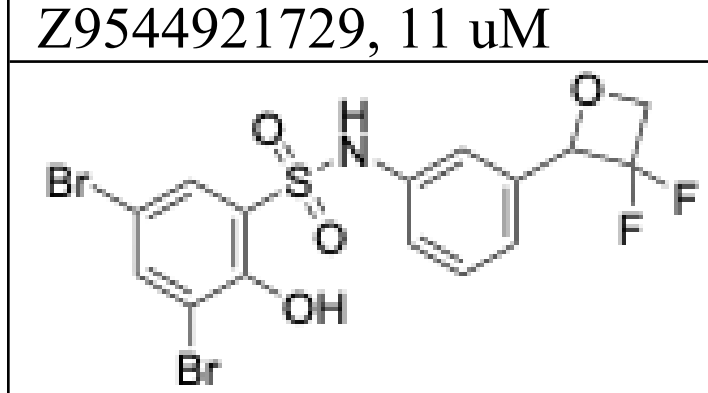 Z9103952148, 12 uM	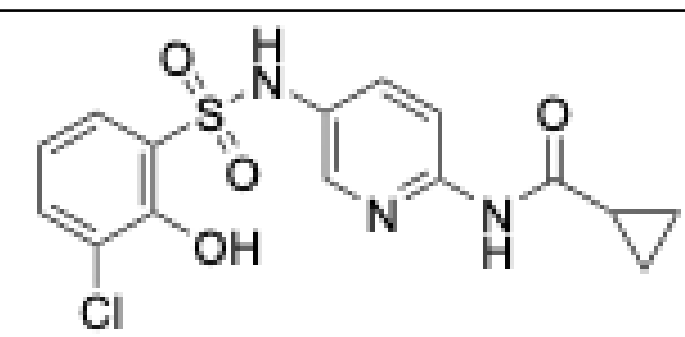 ZINCov000006QgaK, Tc=0.33, 74 uM	1314427
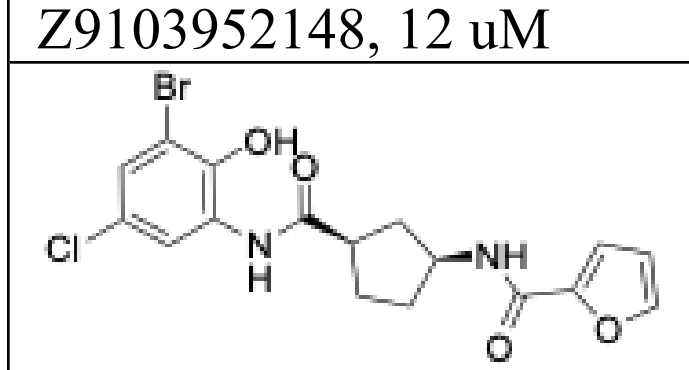 Z9544921944, 12 uM	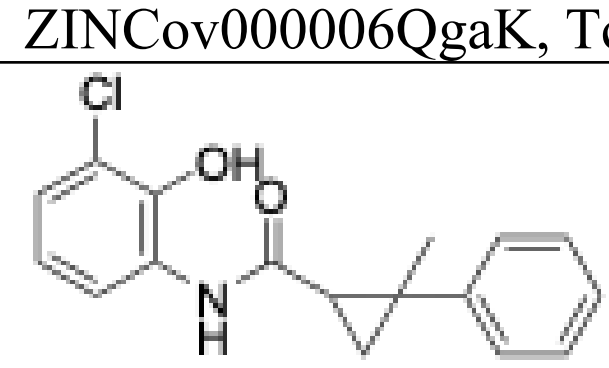 ZINClJ000003SkgO, Tc=0.27, 180 uM	16965271
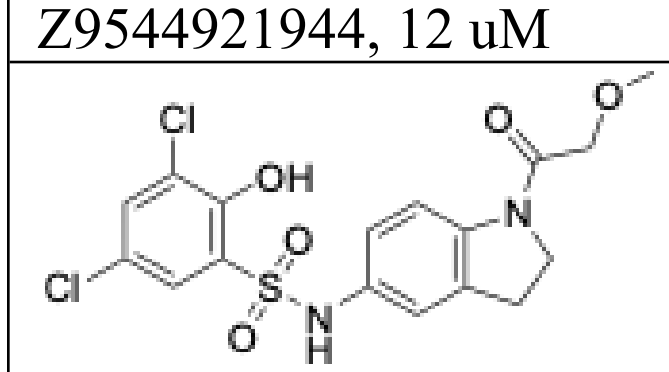 Z2940319191, 13 uM	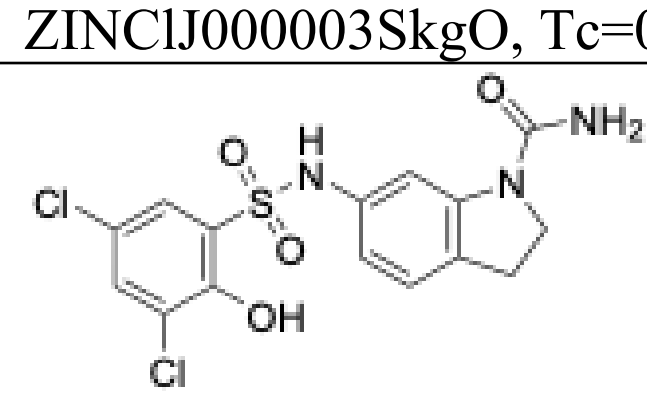 ZINCpz000006Xcju, Tc=0.63, 13 uM	318

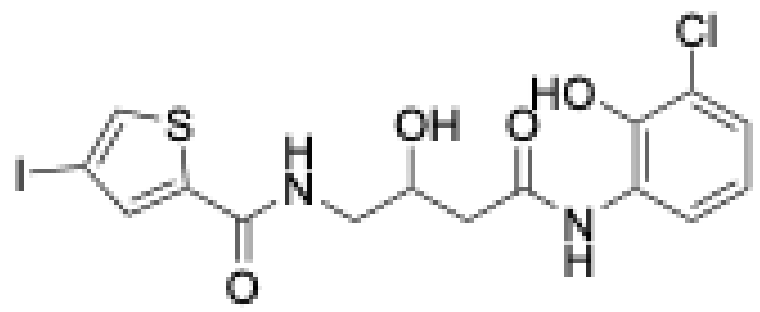 Z9103952022, 13 uM	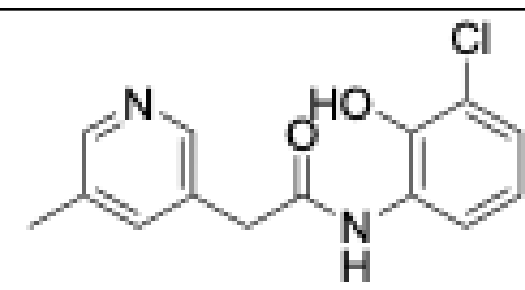 ZINCjz000003xoS3, Tc=0.38, 61 uM	619905
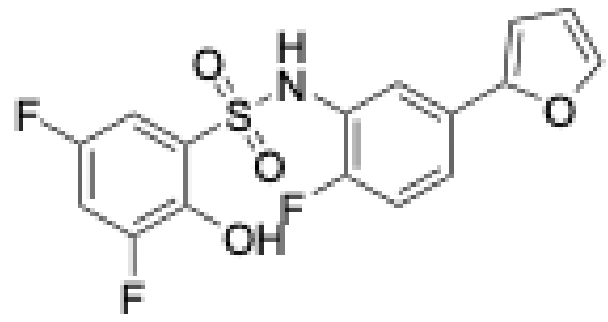 Z9544922335, 13 uM	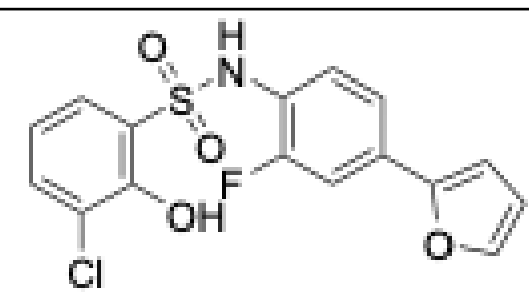 ZINCoM00000e5aKW, Tc=0.59, 4.6 uM	666

Table S2. Atypical AmpC inhibitors (**Figure S9**) compared to the most-similar compound from the brute-force 1.7B campaign, with Tc values (Morgan R=2), and previously reported Ki values.

**Compound, Ki**	**Closest known (Liu et al), Tc, Ki**
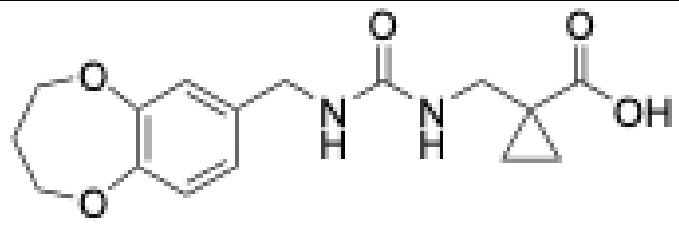 Z3510768208, 130 uM	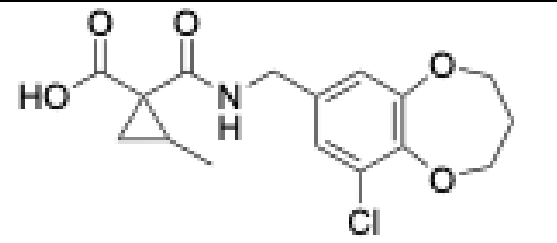 ZINCns000000LyBi, Tc=0.48, 46 uM
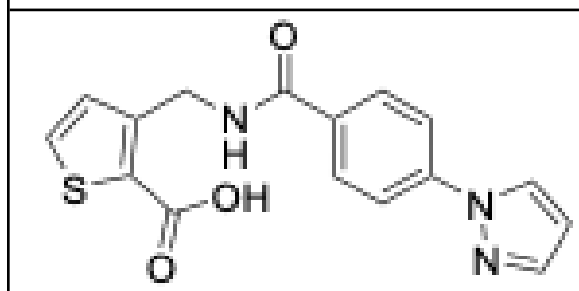 Z3032896420, 260 uM	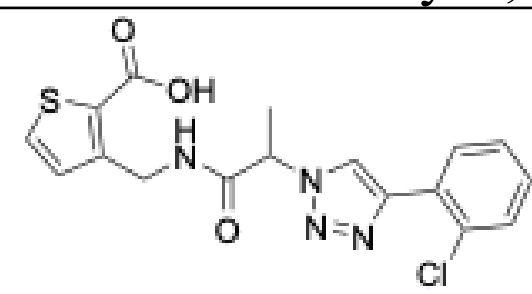 ZINCqC00000ajoRK, Tc=0.39, 320 uM
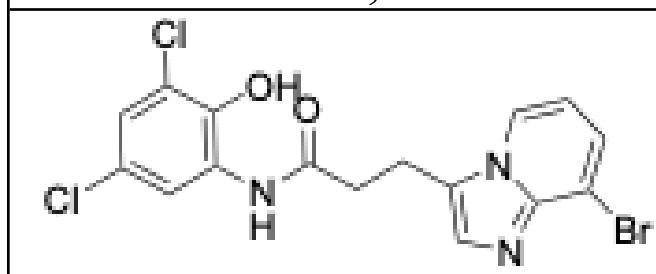 Z9544922144, 43 uM	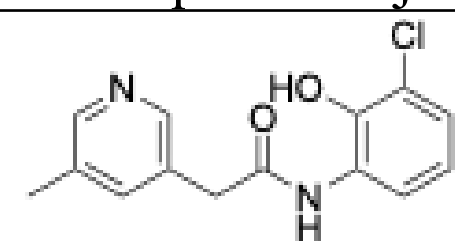 ZINCjz000003xoS3, Tc=0.34, 61 uM
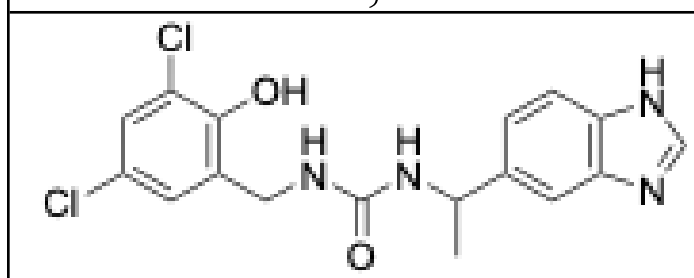 Z9544921938, 42 uM	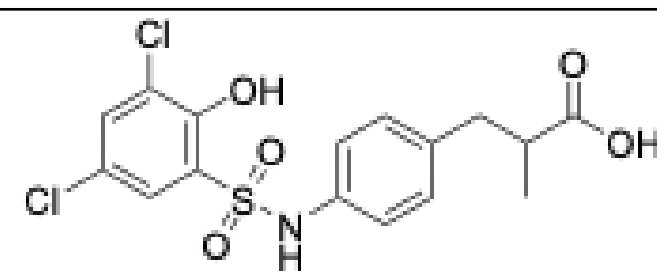 ZINCpH000006mtN0, Tc=0.30, 6.4 uM
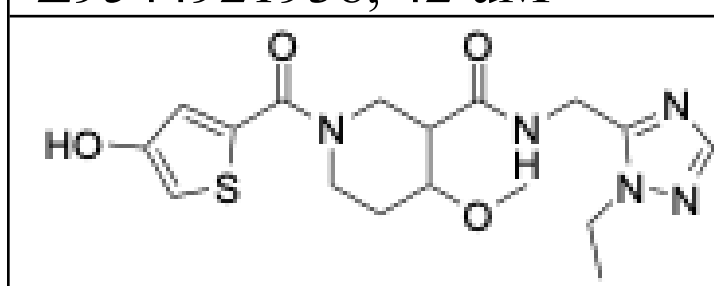 Z9544921905, 360 uM	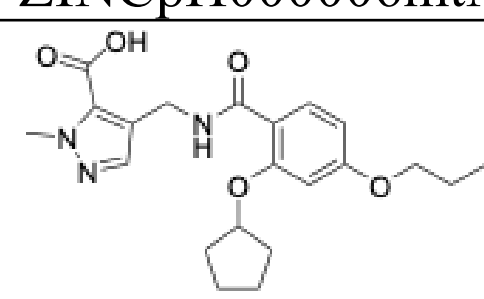 ZINCtB000007j0KP, Tc=0.21, 330 uM
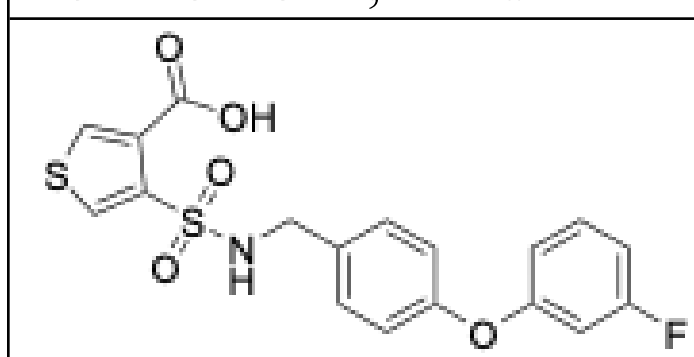 Z9103952299, 40 uM	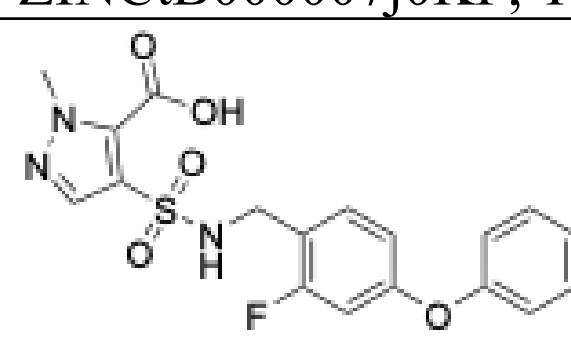 ZINCsv0000066w6Z, Tc=0.43, 200 uM
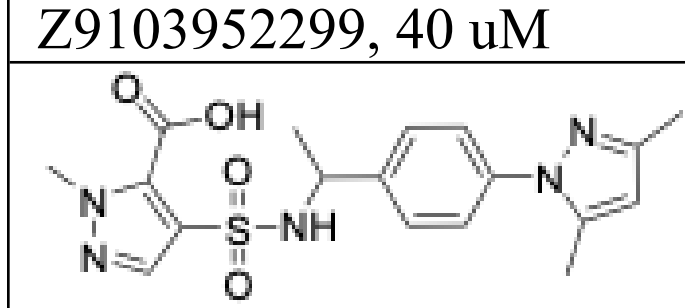 Z9212925012, 61 uM	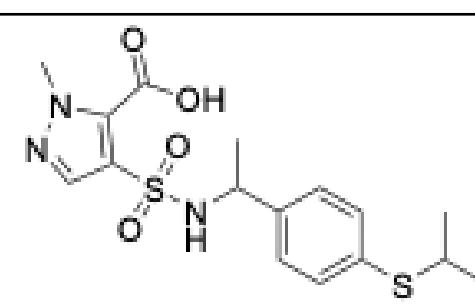 ZINCpw000006KaSB, Tc=0.62, 300 uM
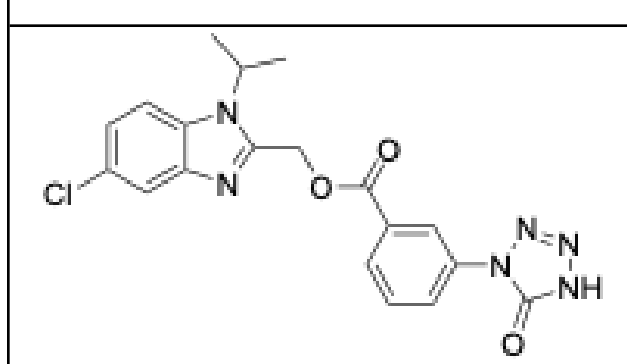 Z9544922119, 220 uM	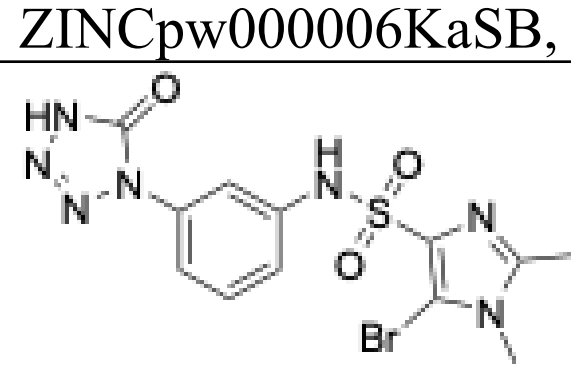 ZINCob000000Fu00, Tc=0.31, 110 uM
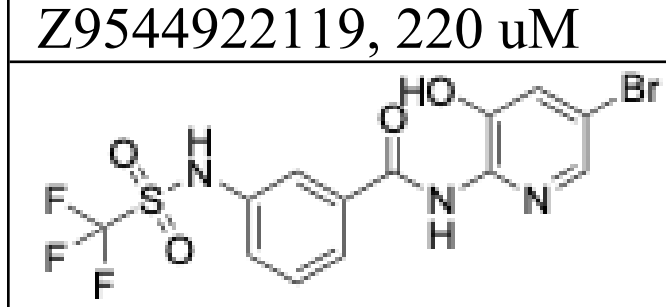 Z9464814813, 360 uM	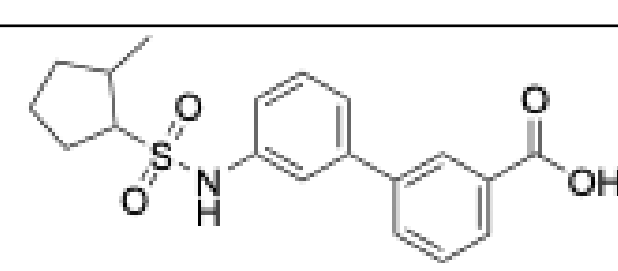 ZINCpJ000005Qtv7, Tc=0.28, 230 uM

General considerations. All chemicals for the synthesis of the studied compounds were provided by Enamine Ltd. (www.enamine.net). All solvents were treated according to standard methods. ^1H NMR spectra were recorded at 500 MHz, ^{13}C NMR spectra at 101 and 151 MHz (Varian or Bruker spectrometers). ^1H chemical shifts are calibrated using residual nondeuterated solvent DMSO, δ = 2.50 ppm. ^{13}C chemical shifts are calibrated using residual solvent DMSO, δ = 39.5 ppm. Coupling constants are given in Hz. LC/MS analysis was performed utilizing an Agilent 1200 Series LC/MSD system with DAD/ELSD (column Zorbax SB-C18 1.8 μm 4.6x15 mm; solvent A (water, 0.1% formic acid) and solvent B (acetonitrile, 0.1% formic acid); gradient 0% – 100% solvent B, run time, 1.8 min; flow rate, 3 mL/min) and Agilent LC/MSD SL (G6130A) or SL (G6140A) mass spectrometer (APCI mode). All the LC/MS data were obtained using positive/negative mode switching.

Synthetic procedures

Method 1. An amine or an alcohol (50 mg) was dissolved in 0.5 mL of glacial acetic acid in a 4 mL vial. Then sodium acetate (1.1 mol. eq. to the amine or the alcohol) and a sulfonyl chloride (1 eq. to the amine) were added. The vial was sealed and kept in an ultrasonic bath for 24 h. Then, the solvent was removed under reduced pressure, and the crude was dissolved in 0.5 mL of DMSO. The sample was further purified by preparative HPLC.

Method 2. An aryl amine (50 mg) and DIPEA (1.5 mol. eq. to the aryl amine) were dissolved in 0.7 mL of acetonitrile in a 4 mL vial. Then, 2,2,2-trifluoroethyl chloroformate (1 mol. eq. to the aryl amine) was added dropwise. The sealed vial was shaken for 30 min at room temperature, and an amino acid *t*-butyl ester (1.1 mol. eq. to the aryl amine) was added. The reaction mixture was kept in an oven for 16 h at 100°C, then the solvent was removed under reduced pressure. The crude was dissolved in 0.5 mL of DMSO and further purified by preparative HPLC.

Method 3. The synthesis was performed according to a previously published procedure (*dx.doi.org/10.1002/ejoc.201501579*).

Method 4. A carboxylic acid (100 mg) was dissolved in 0.5 mL of 10% Hydroxybenzotriazole (HOBt) in DMF, followed by the addition of an amine (1 mol equiv to the carboxylic acid) and 1-ethyl-3-(3-dimethylaminopropyl)carbodiimide (EDC) (1.2 mol equiv to the carboxylic acid). The resulting mixture was shaken for 24 h at room temperature. Then, $CHCl_3$ (2 mL) was added, and the organic phase was washed with water, dried over sodium sulfate, and evaporated under reduced pressure. The crude was dissolved in 0.5 mL of DMSO and further purified by preparative HPLC.

Method 5. An aryl amine (50 mg) and DIPEA (1.5 mol. eq. to the aryl amine) were dissolved in 0.7 mL of acetonitrile in a 4 mL vial. Then, 2,2,2-trifluoroethyl chloroformate (1 mol. eq. to the aryl amine) was added dropwise. The sealed vial was shaken for 30 min at room temperature, and an amino acid *t*-butyl ester (1.1 mol. eq. to the aryl amine) was added. The reaction mixture was kept in an oven for 16 h at 100°C, then the solvent was removed under reduced pressure. The sample was dissolved in 0.6 mL of TFA, placed in an ultrasonic bath for 1 h, and then kept overnight at room temperature. TFA was further removed under reduced pressure, and the crude sample was dissolved in 0.5 mL of DMSO and purified by preparative HPLC.

Method 6. A carboxylic acid (100 mg) was dissolved in 0.5 mL of 10% Hydroxybenzotriazole (HOBt) in DMF, followed by the addition of an amine (1 mol equiv to the carboxylic acid) and 1-ethyl-3-(3-dimethylaminopropyl)carbodiimide (EDC) (1.2 mol equiv to the carboxylic acid). The resulting mixture was shaken for 24 h at room temperature. Then, $CHCl_3$ (2 mL) was added, and the organic phase was washed with water, dried over sodium sulfate, and evaporated under reduced pressure. The sample was dissolved in 0.5 mL of DMSO and subjected to hydrolysis with 0.2 mL of 4M KOH for 12 h at room temperature. Then, 50 uL of formic acid was added, and the reaction vial was shaken for 30 min at room temperature. The solvent was evaporated under reduced pressure, and the crude was dissolved in 0.5 mL of DMSO. The sample was further purified by preparative HPLC.

Method 7. An amine (50 mg) was dissolved in 0.5 mL of glacial acetic acid in a 4 mL vial. Then sodium acetate (1.1 mol. eq. to the amine) and a sulfonyl chloride (1 eq. to the amine) were added. The vial was sealed and kept in an ultrasonic bath for 24 h. Then, the solvent was removed under reduced pressure. After the addition of 0.6 mL of TFA, the sample was placed in an ultrasonic bath for 1 h, and then kept overnight at room temperature. TFA was further removed under reduced pressure, and the crude sample was dissolved in 0.5 mL of DMSO and purified by preparative HPLC.

Method 8. An amine (50 mg) was dissolved in 0.5 mL of glacial acetic acid in a 4 mL vial. Then sodium acetate (1.1 mol. eq. to the amine) and a sulfonyl chloride (1 eq. to the amine) were added. The vial was sealed and kept in an ultrasonic bath for 24 h. Then, the solvent was removed under reduced pressure. The sample was dissolved in 0.5 mL of DMSO and subjected to hydrolysis with 0.2 mL of 4M KOH for 12 h at room temperature. Then, 50 uL of formic acid was added, and the reaction vial was shaken for 30 min at room temperature. The solvent was evaporated under reduced pressure, and the crude was dissolved in 0.5 mL of DMSO. The sample was further purified by preparative HPLC.

Method 9. A carboxylic acid (100 mg) was dissolved in 0.5 mL of 10% Hydroxybenzotriazole (HOBt) in DMF, followed by the addition of an amine (1 mol equiv to the carboxylic acid) and 1-ethyl-3-(3-dimethylaminopropyl)carbodiimide (EDC) (1.2 mol equiv to the carboxylic acid). The resulting mixture was shaken for 24 h at room temperature. Then, $CHCl_3$ (2 mL) was added, and the organic phase was washed with water, dried over sodium sulfate, and evaporated under reduced pressure. After the addition of 0.6 mL of TFA, the sample was placed in an ultrasonic bath for 1 h, and then kept overnight at room temperature. TFA was further removed under reduced pressure, and the crude sample was dissolved in 0.5 mL of DMSO and purified by preparative HPLC.

Method 10. The synthesis was performed according to a previously published procedure (*doi.org/10.1016/j.isci.2020.101681*)

Method 11. An aryl halide (100 mg), boronic derivative (1 mol equiv to the aryl halide), sodium carbonate (1.5 mol equiv to the aryl halide), 1,1′- [bis(diphenylphosphino)ferrocene]-dichloropalladium(II) (0.05 mol equiv to the aryl halide), and a water-dioxane mixture 1:3 (1 mL) were placed in a vial. The vial was then placed into a thermostat and stirred for 24 h at 95 °C. After cooling down the mixture, the solvents were evaporated under reduced pressure. The residue was dissolved in chloroform (3 mL) and washed with water (3 × 1 mL). The catalyst was then filtered, and solvents were evaporated under reduced pressure. The crude sample was dissolved in 0.5 mL of DMSO and purified by preparative HPLC.

Spectral description of the synthesized compounds.
3,5-dichloro-2-hydroxy-N-(2-methyl-4-thiomorpholinophenyl)benzenesulfonamide – Z2940310451 (Method 1).
Yield: 16%; purity, >95% (assessed by LC/MS).
LC/MS (APSI) m/z [M+H] calculated for C17H19Cl2N2O3S2: 433.0; found: 433.0.
^{1}H NMR (500 MHz, DMSO-d_6) δ 10.94 (s, 1H), 9.28 (s, 1H), 7.83 (d, J = 2.7 Hz, 1H), 7.36 (d, J = 2.7 Hz, 1H), 6.70 (d, J = 8.7 Hz, 2H), 6.60 (dd, J = 8.8, 2.9 Hz, 1H), 3.48 – 3.42 (m, 4H), 2.61 – 2.55 (m, 4H), 2.10 (s, 3H).

3,5-dichloro-2-hydroxy-N-[5-methyl-6-(2-methylpiperidin-1-yl)pyridin-3-yl]benzene-1-sulfonamide – Z2940604882 (Method 1).
Yield: 13%; purity, >95% (assessed by LC/MS).
LC/MS (APSI) m/z [M+H] calculated for C18H22Cl2N3O3S: 430.0; found: 430.0.
^{1}H NMR (500 MHz, DMSO-d_6) δ 7.84 (d, J = 2.7 Hz, 1H), 7.79 (d, J = 2.7 Hz, 1H), 7.53 (d, J = 2.6 Hz, 1H), 7.24 (d, J = 2.7 Hz, 1H), 2.88 (dd, J = 11.2, 5.8 Hz, 1H), 2.60 (s, 1H), 2.12 (s, 3H), 1.72 – 1.62 (m, 1H), 1.57 – 1.50 (m, 2H), 1.43 – 1.20 (m, 2H), 0.74 (d, J = 6.2 Hz, 3H).

3,4-difluoro-N-(4-fluoro-3-(2-oxooxazolidin-3-yl)phenyl)-2-hydroxybenzenesulfonamide – Z8238930017 (Method 1).
Yield: 18%; purity, >95% (assessed by LC/MS).
LC/MS (APSI) m/z [M+H] calculated for C15H12F3N2O5S: 389.0; found: 389.0.
^{1}H NMR (500 MHz, DMSO-d_6) δ 7.50 (t, J = 7.4 Hz, 1H), 7.25 (dd, J = 6.9, 2.7 Hz, 1H), 7.17 (dd, J = 10.8, 8.9 Hz, 1H), 6.97 (dt, J = 9.0, 3.5 Hz, 1H), 6.89 (d, J = 8.5 Hz, 1H), 4.42 (t, J = 7.9 Hz, 2H), 3.89 (t, J = 7.9 Hz, 2H).

3-chloro-2-hydroxy-N-(1-(thiophen-2-yl)cyclopropyl)benzenesulfonamide – Z9103951892 (Method 1).
Yield: 14%; purity, >95% (assessed by LC/MS).
LC/MS (APSI) m/z [M-H] calculated for C13H11ClNO3S2: 328.0; found: 328.0.
^{1}H NMR (500 MHz, DMSO-d_6) δ 10.17 (s, 1H), 8.64 (s, 1H), 7.49 (d, J = 7.9 Hz, 1H), 7.47 – 7.42 (m, 1H), 7.13 (d, J = 5.1 Hz, 1H), 6.83 (t, J = 7.9 Hz, 1H), 6.73 (d, J = 3.5 Hz, 1H), 6.67 (t, J = 4.3 Hz, 1H), 1.21 (q, J = 4.9 Hz, 2H), 1.02 (q, J = 4.9 Hz, 2H).

3,5-dibromo-N-(3-ethoxy-4-fluorophenyl)-2-hydroxybenzenesulfonamide – Z9103951943 (Method 1).
Yield: 32%; purity, >95% (assessed by LC/MS).
LC/MS (APSI) m/z [M+H] calculated for C14H13Br2FNO4S: 469.9; found: 469.8.
^{1}H NMR (500 MHz, DMSO-d_6) δ 7.79 (s, 1H), 7.52 (s, 1H), 7.04 (dd, J = 11.3, 8.7 Hz, 1H), 6.82 (dd, J = 7.8, 2.6 Hz, 1H), 6.58 (dt, J = 8.8, 3.3 Hz, 1H), 3.95 (q, J = 7.0 Hz, 2H), 1.27 (t, J = 7.0 Hz, 3H).

3,5-dibromo-2-hydroxy-N-(3-methyl-2-oxo-2,3-dihydrobenzo[d]thiazol-5-yl)benzenesulfonamide – Z9103952053 (Method 1).
Yield: 39%; purity, >95% (assessed by LC/MS).
LC/MS (APSI) m/z [M+H] calculated for C14H11Br2N2O4S2: 494.8; found: 494.8.
^{1}H NMR (500 MHz, DMSO-d_6) δ 10.70 (br s, 2H), 8.00 (d, J = 2.5 Hz, 1H), 7.79 (d, J = 2.5 Hz, 1H), 7.48 (d, J = 8.4 Hz, 1H), 6.97 (d, J = 2.1 Hz, 1H), 6.92 (dd, J = 8.4, 2.1 Hz, 1H), 3.28 (s, 3H).

methyl 2-bromo-5-((3,5-dichloro-2-hydroxyphenyl)sulfonamido)-4-methylbenzoate – Z9103952171 (Method 1).

Yield: 39%; purity, >95% (assessed by LC/MS).
LC/MS (APSI) m/z [M-H] calculated for C15H11BrCl2NO5S: 467.9; found: 467.8.
^{1}H NMR (500 MHz, DMSO-d_6) δ 11.15 (s, 1H), 7.85 (d, J = 2.7 Hz, 1H), 7.59 (s, 1H), 7.50 – 7.46 (m, 2H), 3.79 (s, 3H), 2.18 (s, 3H).

3,5-dibromo-2-hydroxy-N-[2-methyl-4-(oxetan-3-yloxy)phenyl]benzene-1-sulfonamide – Z9544921833 (Method 1).
Yield: 23%; purity, >95% (assessed by LC/MS).
LC/MS (APSI) m/z [M-H] calculated for C16H16Br2NO5S: 491.9; found: 491.8.
^{1}H NMR (500 MHz, DMSO-d_6) δ 7.81 (s, 1H), 7.35 (s, 1H), 6.77 (d, J = 8.7 Hz, 1H), 6.59 (d, J = 2.9 Hz, 1H), 6.47 (dd, J = 8.8, 2.9 Hz, 1H), 5.16 (p, J = 5.5 Hz, 1H), 4.85 (t, J = 6.7 Hz, 2H), 4.46 (dd, J = 7.3, 5.0 Hz, 2H), 2.16 (s, 3H).

3-chloro-N-[1-(difluoromethyl)-1H-indazol-5-yl]-2-hydroxybenzene-1-sulfonamide – Z9544921868 (Method 1).
Yield: 15%; purity, >95% (assessed by LC/MS).
LC/MS (APSI) m/z [M+H] calculated for C14H11ClF2N3O3S: 374.0; found: 374.0.
^{1}H NMR (500 MHz, DMSO-d_6) δ 8.31 (s, 1H), 8.06 (s, 1H), 7.69 (d, J = 8.9 Hz, 1H), 7.61 (d, J = 8.1 Hz, 1H), 7.56 (dd, J = 9.1, 1.8 Hz, 2H), 7.34 (dd, J = 9.0, 2.0 Hz, 1H), 6.88 (t, J = 8.0 Hz, 1H).

N-[5-(5-cyanothiophen-2-yl)-2-methylphenyl]-4-fluoro-2-hydroxybenzene-1-sulfonamide – Z9544921957 (Method 1).
Yield: 22%; purity, >95% (assessed by LC/MS).
LC/MS (APSI) m/z [M+H] calculated for C18H14FN2O3S2: 389.0; found: 388.8.
^{1}H NMR (500 MHz, DMSO-d_6) δ 7.92 (d, J = 4.0 Hz, 1H), 7.55 (dd, J = 8.8, 6.8 Hz, 1H), 7.42 (d, J = 4.0 Hz, 1H), 7.38 (d, J = 2.0 Hz, 1H), 7.30 (dd, J = 7.9, 2.0 Hz, 1H), 7.19 (d, J = 7.9 Hz, 1H), 6.67 (dd, J = 10.9, 2.5 Hz, 1H), 6.60 (td, J = 8.6, 2.6 Hz, 1H), 2.20 (s, 3H).

5-[({3-fluoro-[1,1'-biphenyl]-4-yl}methyl)carbamoyl]-2-methyl-4H-thieno[3,2-b]pyrrole-3-carboxylic acid – Z9544922018 (Method 6).
Yield: 36%; purity, >95% (assessed by LC/MS).
LC/MS (APSI) m/z [M+H] calculated for C22H18FN2O3S: 409.1; found: 409.0.
^{1}H NMR (500 MHz, DMSO-d_6) δ 10.75 (s, 1H), 8.99 (d, J = 6.2 Hz, 1H), 7.70 – 7.65 (m, 2H), 7.54 – 7.42 (m, 5H), 7.37 (t, J = 7.4 Hz, 1H), 7.06 (d, J = 1.7 Hz, 1H), 4.52 (d, J = 5.5 Hz, 2H), 2.72 (s, 3H).

N-(1-benzyl-1,2,3,4-tetrahydroquinolin-6-yl)-5-chloro-2-hydroxybenzene-1-sulfonamide – Z9544922043 (Method 1).
Yield: 17%; purity, >95% (assessed by LC/MS).
LC/MS (APSI) m/z [M+H] calculated for C22H22ClN2O3S: 429.1; found: 429.0.
^{1}H NMR (500 MHz, DMSO-d_6) δ 7.40 (dd, J = 6.3, 2.8 Hz, 2H), 7.27 (t, J = 7.5 Hz, 2H), 7.18 (t, J = 9.4 Hz, 3H), 6.94 (d, J = 9.4 Hz, 1H), 6.65 (d, J = 2.6 Hz, 1H), 6.58 (dd, J = 8.7, 2.6 Hz, 1H), 6.27 (d, J = 8.8 Hz, 1H), 4.37 (s, 2H), 3.25 (d, J = 11.4 Hz, 1H), 2.59 (t, J = 6.3 Hz, 2H), 1.82 (p, J = 6.0 Hz, 2H).

3,5-dichloro-N-(6-ethylquinolin-5-yl)-2-hydroxybenzene-1-sulfonamide – Z9544922503 (Method 1).
Yield: 16%; purity, >95% (assessed by LC/MS).
LC/MS (APSI) m/z [M+H] calculated for C17H15Cl2N2O3S: 397; found: 397.

^{1}H NMR (500 MHz, DMSO-d_6) δ 11.21 (s, 1H), 10.12 (s, 1H), 8.79 (dd, J = 4.2, 1.6 Hz, 1H), 8.30 (d, J = 8.4 Hz, 1H), 7.92 (d, J = 8.8 Hz, 1H), 7.84 (d, J = 2.7 Hz, 1H), 7.66 (d, J = 8.8 Hz, 1H), 7.43 (dd, J = 8.6, 4.2 Hz, 1H), 7.31 (d, J = 2.7 Hz, 1H), 2.65 (q, J = 7.5 Hz, 2H), 0.96 (t, J = 7.5 Hz, 3H).

3-chloro-N-(3-(cyclopentyloxy)-2-methylphenyl)-2-hydroxybenzenesulfonamide – Z9103952180 (Method 1).
Yield: 25%; purity, >95% (assessed by LC/MS).
LC/MS (APSI) m/z [M+H] calculated for C18H21ClNO4S: 382.1; found: 382.0.
^{1}H NMR (500 MHz, DMSO-d_6) δ 7.58 (d, J = 7.9 Hz, 1H), 7.41 (d, J = 7.9 Hz, 1H), 6.95 (t, J = 8.1 Hz, 1H), 6.81 (t, J = 7.9 Hz, 1H), 6.73 (d, J = 8.2 Hz, 1H), 6.55 (d, J = 8.0 Hz, 1H), 4.76 – 4.70 (m, 1H), 1.96 (s, 3H), 1.82 (dt, J = 11.3, 6.3 Hz, 2H), 1.69 – 1.60 (m, 4H), 1.55 (tq, J = 12.2, 8.0, 6.0 Hz, 2H).

ethyl 2-((3,5-dichloro-2-hydroxyphenyl)sulfonamido)-2,3-dihydro-1H-indene-5-carboxylate – Z9103952287 (Method 1).
Yield: 22%; purity, >95% (assessed by LC/MS).
LC/MS (APSI) m/z [M-H] calculated for C18H16Cl2NO5S: 430.0; found: 430.0.
^{1}H NMR (500 MHz, DMSO-$_d$6) δ 7.81 (s, 1H), 7.72 (d, J = 7.3 Hz, 2H), 7.60 (d, J = 2.7 Hz, 1H), 7.28 (d, J = 8.0 Hz, 1H), 4.26 (q, J = 7.1 Hz, 2H), 4.08 (t, J = 7.5 Hz, 1H), 3.04 (dt, J = 15.2, 7.1 Hz, 2H), 2.84 (dt, J = 16.4, 5.7 Hz, 2H), 1.28 (t, J = 7.1 Hz, 3H).

3-chloro-N-(3-(2,2-difluorocyclopropyl)-5-(trifluoromethyl)phenyl)-2-hydroxybenzenesulfonamide – Z9103952313 (Method 1).
Yield: 46%; purity, >95% (assessed by LC/MS).
LC/MS (APSI) m/z [M-H] calculated for C16H10ClF5NO3S: 426.0; found: 425.8.
^{1}H NMR (500 MHz, DMSO-d_6) δ 7.63 (d, J = 7.9 Hz, 1H), 7.52 (d, J = 7.9 Hz, 1H), 7.25 (s, 1H), 7.15 (s, 2H), 6.82 (t, J = 8.0 Hz, 1H), 3.02 (q, J = 11.9 Hz, 1H), 1.97 (s, 1H), 1.82 (s, 1H).

3-chloro-2-hydroxy-N-(4-methyl-3-(2-oxooxazolidin-3-yl)phenyl)benzenesulfonamide – Z9212924971 (Method 1).
Yield: 33%; purity, >95% (assessed by LC/MS).
LC/MS (APSI) m/z [M+H] calculated for C16H16ClN2O5S: 383.1; found: 383.0.
^{1}H NMR (500 MHz, DMSO-d_6) δ 10.55 (s, 1H), 10.29 (s, 1H), 7.67 (dd, J = 8.0, 1.6 Hz, 1H), 7.59 (dd, J = 8.0, 1.7 Hz, 1H), 7.10 (d, J = 8.3 Hz, 1H), 7.02 (d, J = 2.2 Hz, 1H), 6.97 – 6.90 (m, 2H), 4.42 (t, J = 7.9 Hz, 2H), 3.77 (t, J = 7.9 Hz, 2H), 2.05 (s, 3H).

2-(2-chloro-4-((3,5-dibromo-2-hydroxyphenyl)sulfonamido)phenoxy)acetic acid – Z9464814846 (Method 8).
Yield: 52%; purity, >95% (assessed by LC/MS).
LC/MS (APSI) m/z [M-H] calculated for C14H9Br2ClNO6S: 513.8; found: 513.6.
^{1}H NMR (500 MHz, DMSO-d_6) δ 7.94 (d, J = 2.6 Hz, 1H), 7.63 (d, J = 2.5 Hz, 1H), 7.13 (d, J = 2.6 Hz, 1H), 6.97 (dd, J = 8.9, 2.6 Hz, 1H), 6.91 (d, J = 9.0 Hz, 1H), 4.70 (s, 2H).

1-(4-((3,5-dibromo-2-hydroxyphenyl)sulfonamido)phenyl)cyclobutane-1-carboxylic acid – Z9464814853 (Method 1).
Yield: 19%; purity, >95% (assessed by LC/MS).
LC/MS (APSI) m/z [M-H] calculated for C17H14Br2NO5S: 503.9; found: 504.0.
^{1}H NMR (500 MHz, DMSO-d_6) δ 12.24 (s, 1H), 10.76 (s, 1H), 10.31 (s, 1H), 7.99 (d, J = 2.5 Hz, 1H), 7.73 (d, J = 2.5 Hz, 1H), 7.13 (d, J = 8.4 Hz, 2H), 7.04 (d, J = 8.4 Hz, 2H), 2.65 – 2.56 (m,

2H), 2.29 (dt, J = 11.9, 8.9 Hz, 2H), 1.85 (dq, J = 17.0, 8.1 Hz, 1H), 1.71 (dq, J = 14.5, 8.9, 6.5 Hz, 1H).

3-chloro-2-hydroxy-N-(3-((3-oxo-[1,2,4]triazolo[4,3-a]pyridin-2(3H)-yl)methyl)phenyl)benzenesulfonamide – Z2027058992 (Method 1).
Yield: 36%; purity, >95% (assessed by LC/MS).
LC/MS (APSI) m/z [M+H] calculated for $C_{19}H_{16}ClN_4O_4S$: 431.1; found: 431.0.

N-(4-(2,6-dimethylmorpholino)-3-fluorophenyl)-3,5-difluoro-2-hydroxybenzenesulfonamide – Z2027059519 (Method 1).
Yield: 33%; purity, >95% (assessed by LC/MS).
LC/MS (APSI) m/z [M+H] calculated for $C_{18}H_{20}F_3N_2O_4S$: 417.1; found: 417.0.

1-(3,5-dichloro-2-hydroxybenzyl)-3-((2-(furan-2-yl)thiazol-4-yl)methyl)urea – Z2117758721 (Method 2).
Yield: 24%; purity, >95% (assessed by LC/MS).
LC/MS (APSI) m/z [M+H] calculated for $C_{16}H_{14}Cl_2N_3O_3S$: 398.0; found: 398.0.

methyl 5-(2-((3,5-dichloro-2-hydroxybenzyl)amino)-2-oxoacetamido)thiophene-3-carboxylate – Z2127627626 (Method 3).
Yield: 11%; purity, >95% (assessed by LC/MS).
LC/MS (APSI) m/z [M+H] calculated for $C_{15}H_{13}Cl_2N_2O_5S$: 403.0; found: 402.8.

N-(4-((5-chloro-2-hydroxyphenyl)sulfonamido)phenyl)-5-methylisoxazole-4-carboxamide – Z2234672760 (Method 1).
Yield: 16%; purity, >95% (assessed by LC/MS).
LC/MS (APSI) m/z [M+H] calculated for $C_{17}H_{15}ClN_3O_5S$: 408.0; found: 408.0.

allyl 4-(2-((3-chloro-5-fluoro-2-hydroxyphenyl)amino)-2-oxoethyl)piperidine-1-carboxylate – Z2480765487 (Method 4).
Yield: 36%; purity, >95% (assessed by LC/MS).
LC/MS (APSI) m/z [M+H] calculated for $C_{17}H_{21}ClFN_2O_4$: 371.1; found: 371.2.

N-(1-(6-ethylpyrimidin-4-yl)piperidin-4-yl)-2-hydroxybenzenesulfonamide – Z2579117565 (Method 1).
Yield: 16%; purity, >95% (assessed by LC/MS).
LC/MS (APSI) m/z [M+H] calculated for $C_{17}H_{23}N_4O_3S$: 363.2; found: 363.2.

2-((5-fluoro-2-hydroxyphenyl)sulfonamido)-N-methyl-N-(2-(pyridin-2-yl)ethyl)acetamide – Z2850291544 (Method 1).
Yield: 9%; purity, >95% (assessed by LC/MS).
LC/MS (APSI) m/z [M+H] calculated for $C_{16}H_{19}FN_3O_4S$: 368.1; found: 368.2.

3,5-dichloro-N-(1-(2-fluorophenyl)cyclopropyl)-2-hydroxybenzenesulfonamide – Z2940313538 (Method 1).
Yield: 16%; purity, >95% (assessed by LC/MS).
LC/MS (APSI) m/z [M+H] calculated for $C_{15}H_{13}Cl_2FNO_3S$: 375.0; found: 376.0.

3,5-dichloro-2-hydroxy-N-(1-(2-methoxyacetyl)indolin-5-yl)benzenesulfonamide – Z2940319191 (Method 1).
Yield: 19%; purity, >95% (assessed by LC/MS).

LC/MS (APSI) m/z [M+H] calculated for C17H17Cl2N2O5S: 431.0; found: 430.8.

3,5-dichloro-2-hydroxy-N-[4-({5H,6H,7H-pyrrolo[2,1-c][1,2,4]triazol-3-yl}methyl)phenyl]benzene-1-sulfonamide – Z2940319985 (Method 1).
Yield: 12%; purity, >95% (assessed by LC/MS).
LC/MS (APSI) m/z [M+H] calculated for C18H17Cl2N4O3S: 439.0; found: 439.0.

3,5-dichloro-N-[2-(2,2-dichloro-1-methylcyclopropyl)ethyl]-2-hydroxybenzene-1-sulfonamide – Z2940599460 (Method 1).
Yield: 21%; purity, >95% (assessed by LC/MS).
LC/MS (APSI) m/z [M+H] calculated for C12H14Cl4NO3S: 391.9; found: 391.8.

3,5-dichloro-2-hydroxy-N-{[5-(2-methyl-1,3-thiazol-4-yl)furan-2-yl]methyl}benzene-1-sulfonamide – Z2940604667 (Method 1).
Yield: 39%; purity, >95% (assessed by LC/MS).
LC/MS (APSI) m/z [M+H] calculated for C15H13Cl2N2O4S2: 421.0; found: 421.0.

3-({[4-(1H-pyrazol-1-yl)phenyl]formamido}methyl)thiophene-2-carboxylic acid – Z3032896420 (Method 4).
Yield: 13%; purity, >95% (assessed by LC/MS).
LC/MS (APSI) m/z [M+H] calculated for C16H14N3O3S: 328.1; found: 328.0.

3-chloro-2-hydroxy-N-{[6-(3-methoxyphenoxy)pyridin-3-yl]methyl}benzene-1-sulfonamide – Z3352582091 (Method 1).
Yield: 45%; purity, >95% (assessed by LC/MS).
LC/MS (APSI) m/z [M+H] calculated for C19H18ClN2O5S: 421.1; found: 421.0.

1-[({[(3,4-dihydro-2H-1,5-benzodioxepin-7-yl)methyl]carbamoyl}amino)methyl]cyclopropane-1-carboxylic acid – Z3510768208 (Method 5).
Yield: 33%; purity, >95% (assessed by LC/MS).
LC/MS (APSI) m/z [M+H] calculated for C16H21N2O5: 321.1; found: 321.2.

methyl (1S,1'R,2S,2'R)-2'-((3-chloro-2-hydroxyphenyl)sulfonamido)-[1,1'-bi(cyclopropane)]-2-carboxylate – Z4005054825 (Method 1).
Yield: 25%; purity, >95% (assessed by LC/MS).
LC/MS (APSI) m/z [M+H] calculated for C14H17ClNO5S: 346.1; found: 346.0.

N-(1-(2-chloro-5-fluoropyrimidin-4-yl)pyrrolidin-3-yl)-2-hydroxybenzenesulfonamide – Z4278888865 (Method 1).
Yield: 12%; purity, >95% (assessed by LC/MS).
LC/MS (APSI) m/z [M+H] calculated for C14H15ClFN4O3S: 373.1; found: 373.0.

N-(1-(2H-tetrazol-5-yl)ethyl)-2-((4-(2-ethylphenyl)-4H-1,2,4-triazol-3-yl)thio)acetamide – Z605931832 (Method 4).
Yield: 67%; purity, >95% (assessed by LC/MS).
LC/MS (APSI) m/z [M+H] calculated for C15H19N8OS: 359.1; found: 359.0.

1-methyl-4-((3-(4-methyl-4H-1,2,4-triazol-3-yl)propanamido)methyl)-1H-pyrazole-5-carboxylic acid – Z6615014431 (Method 6).
Yield: 14%; purity, >95% (assessed by LC/MS).
LC/MS (APSI) m/z [M+H] calculated for C12H17N6O3: 293.1; found: 293.0.

N-(1-acetylindolin-4-yl)-3-chloro-2-hydroxybenzenesulfonamide – Z6615017782 (Method 1).
Yield: 15%; purity, >95% (assessed by LC/MS).
LC/MS (APSI) m/z [M+H] calculated for C16H16ClN2O4S: 367.1; found: 366.9.

N-(4-benzoylbenzyl)-3,5-dichloro-2-hydroxybenzenesulfonamide – Z7889182816 (Method 1).
Yield: 14%; purity, >95% (assessed by LC/MS).
LC/MS (APSI) m/z [M+H] calculated for C20H16Cl2NO4S: 438.0; found: 438.0.

3-chloro-N-(1-(2,4-difluorophenyl)-1-oxopropan-2-yl)-2-hydroxybenzenesulfonamide – Z8124313298 (Method 1).
Yield: 19%; purity, >95% (assessed by LC/MS).
LC/MS (APSI) m/z [M+H] calculated for C15H13ClF2NO4S: 376.0; found: 376.0.

2-hydroxy-N-(1-(pyridin-3-yl)-3-(tetrahydrofuran-2-yl)propyl)benzenesulfonamide – Z8174370523 (Method 1).
Yield: 10%; purity, >95% (assessed by LC/MS).
LC/MS (APSI) m/z [M+H] calculated for C18H23N2O4S: 363.1; found: 363.2.

2-hydroxy-N-((1r,4r)-4-(pyridin-2-ylamino)cyclohexyl)benzenesulfonamide – Z8190786456 (Method 7).
Yield: 13%; purity, >95% (assessed by LC/MS).
LC/MS (APSI) m/z [M+H] calculated for C17H22N3O3S: 348.1; found: 348.0.

methyl 3-((3,4-difluoro-2-hydroxyphenyl)sulfonamido)-1-methylcyclopentane-1-carboxylate – Z8208146597 (Method 1).
Yield: 20%; purity, >95% (assessed by LC/MS).
LC/MS (APSI) m/z [M+H] calculated for C14H18F2NO5S: 350.1; found: 350.0.

methyl 3-((3,5-difluoro-2-hydroxyphenyl)sulfonamido)-1-methylcyclopentane-1-carboxylate – Z8208146860 (Method 1).
Yield: 11%; purity, >95% (assessed by LC/MS).
LC/MS (APSI) m/z [M-H] calculated for C14H16F2NO5S: 348.1; found: 348.2.

ethyl 5-amino-2-(((3-chloro-2-hydroxyphenyl)sulfonamido)methyl)thiazole-4-carboxylate – Z8233436981 (Method 1).
Yield: 35%; purity, >95% (assessed by LC/MS).
LC/MS (APSI) m/z [M+H] calculated for C13H15ClN3O5S2: 392.0; found: 392.0.

3,4-difluoro-N-(1-(3-fluoropyridin-2-yl)pyrrolidin-3-yl)-2-hydroxybenzenesulfonamide – Z8238874619 (Method 1).
Yield: 46%; purity, >95% (assessed by LC/MS).
LC/MS (APSI) m/z [M+H] calculated for C15H15F3N3O3S: 374.1; found: 374.2.

4-(((3,4-difluoro-2-hydroxyphenyl)sulfonamido)methyl)phenyl acetate – Z8238927651 (Method 1).
Yield: 20%; purity, >95% (assessed by LC/MS).
LC/MS (APSI) m/z [M-H] calculated for C15H12F2NO5S: 356.1; found: 356.0.

N-(1-acetyl-1,2,3,4-tetrahydroquinolin-4-yl)-4-fluoro-2-hydroxybenzenesulfonamide – Z8239890489 (Method 1).
Yield: 16%; purity, >95% (assessed by LC/MS).
LC/MS (APSI) m/z [M+H] calculated for C17H18FN2O4S: 365.1; found: 365.2.

5-chloro-N-(3-(3-ethyl-1,2,4-oxadiazol-5-yl)cyclopentyl)-2-hydroxybenzenesulfonamide – Z8241970648 (Method 1).
Yield: 17%; purity, >95% (assessed by LC/MS).
LC/MS (APSI) m/z [M+H] calculated for C15H19ClN3O4S: 372.1; found: 372.2.

(1S,2R)-2-((N-((6-(azepan-1-yl)pyridin-3-yl)methyl)sulfamoyl)methyl)cyclobutane-1-carboxylic acid – Z8269237535 (Method 8).
Yield: 21%; purity, >95% (assessed by LC/MS).
LC/MS (APSI) m/z [M+H] calculated for C18H28N3O4S: 382.2; found: 382.0.

4-(N-(4-methyl-2,3-dihydro-1H-inden-2-yl)sulfamoyl)thiophene-3-carboxylic acid – Z8278108755 (Method 8).
Yield: 44%; purity, >95% (assessed by LC/MS).
LC/MS (APSI) m/z [M+H] calculated for C15H16NO4S2: 338.1; found: 338.0.

N-(5-(5-cyclopropyl-1H-tetrazol-1-yl)-2-fluorophenyl)-4-fluoro-2-hydroxybenzenesulfonamide – Z8318206664 (Method 1).
Yield: 23%; purity, >95% (assessed by LC/MS).
LC/MS (APSI) m/z [M+H] calculated for C16H14F2N5O3S: 394.1; found: 394.0.

4-(((2-hydroxyphenyl)sulfonamido)methyl)phenyl acetate – Z9103951905 (Method 1).
Yield: 16%; purity, >95% (assessed by LC/MS).
LC/MS (APSI) m/z [M-H] calculated for C15H14NO5S: 320.1; found: 320.0.

3,5-dibromo-2-hydroxy-N-(4-isopropylphenyl)benzenesulfonamide – Z9103951919 (Method 1).
Yield: 17%; purity, >95% (assessed by LC/MS).
LC/MS (APSI) m/z [M+H] calculated for C15H16Br2NO3S: 449.9; found: 449.8.

N-(6-bromo-2-methyl-2,3-dihydrobenzofuran-3-yl)-3-chloro-2-hydroxybenzenesulfonamide – Z9103951948 (Method 1).
Yield: 25%; purity, >95% (assessed by LC/MS).
LC/MS (APSI) m/z [M-H] calculated for C15H12BrClNO4S: 417.9; found: 417.8.

3-((3-(2-iodo-5-methoxyphenyl)propanamido)methyl)-1H-pyrazole-4-carboxylic acid – Z9103952005 (Method 6).
Yield: 28%; purity, >95% (assessed by LC/MS).
LC/MS (APSI) m/z [M+H] calculated for C15H17IN3O4: 430.0; found: 430.0.

N-(4-((3-chloro-2-hydroxyphenyl)amino)-2-hydroxy-4-oxobutyl)-4-iodothiophene-2-carboxamide – Z9103952022 (Method 9).
Yield: 13%; purity, >95% (assessed by LC/MS).
LC/MS (APSI) m/z [M+H] calculated for C15H15ClIN2O4S: 481.0; found: 480.8.

N-(7-chloroisoquinolin-8-yl)-3,5-difluoro-2-hydroxybenzenesulfonamide – Z9103952047 (Method 1).

Yield: 35%; purity, >95% (assessed by LC/MS).
LC/MS (APSI) m/z [M+H] calculated for C15H10ClF2N2O3S: 371.0; found: 371.0.

3,5-dichloro-2-hydroxy-N-(1-oxo-2,3,4,5-tetrahydro-1H-benzo[c]azepin-8-yl)benzenesulfonamide – Z9103952128 (Method 1).
Yield: 47%; purity, >95% (assessed by LC/MS).
LC/MS (APSI) m/z [M+H] calculated for C16H15Cl2N2O4S: 401.0; found: 400.8.

3,5-dibromo-N-(3-(3,3-difluorooxetan-2-yl)phenyl)-2-hydroxybenzenesulfonamide – Z9103952148 (Method 1).
Yield: 29%; purity, >95% (assessed by LC/MS).
LC/MS (APSI) m/z [M-H] calculated for C15H10Br2F2NO4S: 497.9; found: 497.8.

4-(N-((2-(4-chlorophenyl)thiazol-5-yl)methyl)sulfamoyl)thiophene-3-carboxylic acid – Z9103952158 (Method 8).
Yield: 12%; purity, >95% (assessed by LC/MS).
LC/MS (APSI) m/z [M+H] calculated for C15H12ClN2O4S3: 415.0; found: 415.0.

3,5-dibromo-N-(4-fluoro-3-(1H-pyrrol-1-yl)phenyl)-2-hydroxybenzenesulfonamide – Z9103952184 (Method 1).
Yield: 40%; purity, >95% (assessed by LC/MS).
LC/MS (APSI) m/z [M+H] calculated for C16H12Br2FN2O3S: 490.9; found: 490.8.

N-((3-(benzyloxy)cyclobutyl)methyl)-3,5-difluoro-2-hydroxybenzenesulfonamide – Z9103952204 (Method 1).
Yield: 20%; purity, >95% (assessed by LC/MS).
LC/MS (APSI) m/z [M+H] calculated for C18H20F2NO4S: 384.1; found: 384.0.

3,5-dichloro-2-hydroxy-N-(4-(N-methylethylsulfonamido)phenyl)benzenesulfonamide – Z9103952229 (Method 1).
Yield: 44%; purity, >95% (assessed by LC/MS).
LC/MS (APSI) m/z [M-H] calculated for C15H15Cl2N2O5S2: 437.0; found: 437.0.

3-chloro-N-((2-(cyclohexyloxy)pyridin-4-yl)methyl)-2-hydroxybenzenesulfonamide – Z9103952235 (Method 1).
Yield: 16%; purity, 92% (assessed by LC/MS).
LC/MS (APSI) m/z [M+H] calculated for C18H22ClN2O4S: 397.1; found: 397.0.

3,5-difluoro-2-hydroxy-N-(3-(thiophen-2-ylethynyl)phenyl)benzenesulfonamide – Z9103952237 (Method 1).
Yield: 31%; purity, >95% (assessed by LC/MS).
LC/MS (APSI) m/z [M+H] calculated for C18H12F2NO3S2: 392.0; found: 391.8.

5-bromo-3-chloro-N-(9,9-difluoro-1-oxaspiro[5.5]undecan-4-yl)-2-hydroxybenzenesulfonamide – Z9103952244 (Method 1).
Yield: 31%; purity, >95% (assessed by LC/MS).
LC/MS (APSI) m/z [M+H] calculated for C16H20BrClF2NO4S: 474.0; found: 474.0.

4-(N-(4-(3-fluorophenoxy)benzyl)sulfamoyl)thiophene-3-carboxylic acid – Z9103952299 (Method 8).
Yield: 59%; purity, >95% (assessed by LC/MS).

LC/MS (APSI) m/z [M-H] calculated for C18H13FNO5S2: 406.0; found: 406.0.

ethyl 2-((3-chloro-2-hydroxyphenyl)sulfonamido)-3-(2,5-dichlorophenyl)propanoate – Z9103952303 (Method 1).
Yield: 40%; purity, 91% (assessed by LC/MS).
LC/MS (APSI) m/z [M-H] calculated for C17H15Cl3NO5S: 452.0; found: 452.0.

2-(3',4'-dichloro-5-fluoro-[1,1'-biphenyl]-2-carboxamido)-4-hydroxy-4-methylpentanoic acid – Z9103952336 (Method 6).
Yield: 44%; purity, >95% (assessed by LC/MS).
LC/MS (APSI) m/z [M-H] calculated for C19H17Cl2FNO4: 412.1; found: 412.0.

4-(N-((2'-cyano-[1,1'-biphenyl]-4-yl)methyl)sulfamoyl)-1-methyl-1H-pyrazole-5-carboxylic acid – Z9103952349 (Method 8).
Yield: 62%; purity, >95% (assessed by LC/MS).
LC/MS (APSI) m/z [M+H] calculated for C19H17N4O4S: 397.1; found: 397.0.

methyl 1-(4-((3,5-difluoro-2-hydroxyphenyl)sulfonamido)phenyl)cyclopentane-1-carboxylate – Z9103952355 (Method 1).
Yield: 44%; purity, >95% (assessed by LC/MS).
LC/MS (APSI) m/z [M-H] calculated for C19H18F2NO5S: 410.1; found: 410.0.

3-chloro-2-hydroxy-N-(2-(perfluoroethyl)-1H-benzo[d]imidazol-5-yl)benzenesulfonamide – Z9103952375 (Method 1).
Yield: 33%; purity, >95% (assessed by LC/MS).
LC/MS (APSI) m/z [M-H] calculated for C15H8ClF5N3O3S: 440.0; found: 439.8.

4-fluoro-N-(3-fluoro-4-(3-methoxyphenoxy)phenyl)-2-hydroxybenzenesulfonamide – Z9103952401 (Method 1).
Yield: 48%; purity, >95% (assessed by LC/MS).
LC/MS (APSI) m/z [M+H] calculated for C19H16F2NO5S: 408.1; found: 408.0.

(R,E)-N-(1-(2-benzylidenebutanoyl)pyrrolidin-3-yl)-2,4-difluoro-6-hydroxybenzamide – Z9103952406 (Method 10).
Yield: 48%; purity, >95% (assessed by LC/MS).
LC/MS (APSI) m/z [M+H] calculated for C22H23F2N2O3: 401.2; found: 401.2.

3-(N-(8-(tert-butoxycarbonyl)-6,7,8,9-tetrahydro-5H-imidazo[1,2-a][1,4]diazepin-6-yl)sulfamoyl)thiophene-2-carboxylic acid – Z9103952413 (Method 8).
Yield: 18%; purity, >95% (assessed by LC/MS).
LC/MS (APSI) m/z [M+H] calculated for C17H23N4O6S2: 443.1; found: 443.0.

tert-butyl (1R,3s,5S)-3-(((3,4-difluoro-2-hydroxyphenyl)sulfonamido)methyl)-8-azabicyclo[3.2.1]octane-8-carboxylate – Z9103952420 (Method 1).
Yield: 18%; purity, >95% (assessed by LC/MS).
LC/MS (APSI) m/z [M-H] calculated for C19H25F2N2O5S: 431.2; found: 431.2.

tert-butyl (1R,3s,5S)-3-(((3,5-difluoro-2-hydroxyphenyl)sulfonamido)methyl)-8-azabicyclo[3.2.1]octane-8-carboxylate – Z9103952426 (Method 1).
Yield: 40%; purity, >95% (assessed by LC/MS).
LC/MS (APSI) m/z [M-H] calculated for C19H25F2N2O5S: 431.2; found: 431.2.

N-(3-((1H-benzo[d]imidazol-1-yl)methyl)phenyl)-3,5-difluoro-2-hydroxybenzenesulfonamide – Z9103952451 (Method 1).
Yield: 28%; purity, >95% (assessed by LC/MS).
LC/MS (APSI) m/z [M-H] calculated for C20H14F2N3O3S: 414.1; found: 414.0.

3,5-dichloro-N-(1-(2,4-difluorobenzyl)-6-oxo-1,6-dihydropyridin-3-yl)-2-hydroxybenzenesulfonamide – Z9103952464 (Method 1).
Yield: 20%; purity, >95% (assessed by LC/MS).
LC/MS (APSI) m/z [M+H] calculated for C18H13Cl2F2N2O4S: 461.0; found: 460.8.

methyl (1R,3R,5R)-3-((5-bromo-3-chloro-2-hydroxyphenyl)sulfonamido)bicyclo[3.1.0]hexane-1-carboxylate – Z9103996037 (Method 1).
Yield: 17%; purity, >95% (assessed by LC/MS).
LC/MS (APSI) m/z [M+H] calculated for C14H16BrClNO5S: 426.0; found: 426.0.

3-(N-((1R,2S)-2-(4-isopropylphenyl)cyclopropyl)sulfamoyl)thiophene-2-carboxylic acid – Z9103997588 (Method 8).
Yield: 37%; purity, >95% (assessed by LC/MS).
LC/MS (APSI) m/z [M+H] calculated for C17H20NO4S2: 366.1; found: 366.0.

3-chloro-2-hydroxy-N-((5-(2-methylthiazol-4-yl)furan-2-yl)methyl)benzenesulfonamide – Z9212924966 (Method 1).
Yield: 41%; purity, >95% (assessed by LC/MS).
LC/MS (APSI) m/z [M+H] calculated for C15H14ClN2O4S2: 385.0; found: 385.0.

3-chloro-2-hydroxy-N-((5-(2-methylthiazol-4-yl)thiophen-2-yl)methyl)benzenesulfonamide – Z9212924967 (Method 1).
Yield: 47%; purity, >95% (assessed by LC/MS).
LC/MS (APSI) m/z [M+H] calculated for C15H14ClN2O3S3: 401.0; found: 401.0.

3-(N-(3,5-dichloro-4-(methylsulfonamido)phenyl)sulfamoyl)thiophene-2-carboxylic acid – Z9212924969 (Method 8).
Yield: 13%; purity, 95% (assessed by LC/MS).
LC/MS (APSI) m/z [M-H] calculated for C12H11Cl2N2O6S3: 444.9; found: 444.8.

3-chloro-N-(1-(2-chloro-5-fluoropyrimidin-4-yl)pyrrolidin-3-yl)-2-hydroxybenzenesulfonamide – Z9212924973 (Method 1).
Yield: 49%; purity, >95% (assessed by LC/MS).
LC/MS (APSI) m/z [M+H] calculated for C14H14Cl2FN4O3S: 407.0; found: 407.0.

3-chloro-2-hydroxy-N-(4-((4-methylthiazol-2-yl)thio)phenyl)benzenesulfonamide – Z9212924975 (Method 1).
Yield: 24%; purity, >95% (assessed by LC/MS).
LC/MS (APSI) m/z [M+H] calculated for C16H14ClN2O3S3: 413.0; found: 413.0.

3-chloro-2-hydroxy-N-(1-(4-methyl-3-oxo-3,4-dihydropyrazin-2-yl)piperidin-4-yl)benzenesulfonamide – Z9212924980 (Method 1).
Yield: 31%; purity, >95% (assessed by LC/MS).
LC/MS (APSI) m/z [M+H] calculated for C16H20ClN4O4S: 399.1; found: 399.0.

(R)-3-chloro-2-hydroxy-N-(1-(thiophen-2-ylsulfonyl)piperidin-3-yl)benzenesulfonamide – Z9212924983 (Method 1).
Yield: 29%; purity, >95% (assessed by LC/MS).
LC/MS (APSI) m/z [M+H] calculated for C15H18ClN2O5S3: 437.0; found: 437.0.

3-chloro-2-hydroxy-N-((6-phenoxypyridin-3-yl)methyl)benzenesulfonamide – Z9212924996 (Method 1).
Yield: 36%; purity, >95% (assessed by LC/MS).
LC/MS (APSI) m/z [M+H] calculated for C18H16ClN2O4S: 391.1; found: 391.0.

3-(N-((6-(4-fluorophenoxy)pyridin-3-yl)methyl)sulfamoyl)thiophene-2-carboxylic acid – Z9212924999 (Method 8).
Yield: 39%; purity, >95% (assessed by LC/MS).
LC/MS (APSI) m/z [M+H] calculated for C17H14FN2O5S2: 409.0; found: 409.0.

methyl 5-((3-chloro-2-hydroxyphenyl)sulfonamido)-2-(trifluoromethoxy)benzoate – Z9212925003 (Method 1).
Yield: 29%; purity, >95% (assessed by LC/MS).
LC/MS (APSI) m/z [M-H] calculated for C15H10ClF3NO6S: 424.0; found: 424.0.

N-((4-(benzofuran-2-yl)thiazol-2-yl)methyl)-3-chloro-2-hydroxybenzenesulfonamide – Z9212925005 (Method 1).
Yield: 20%; purity, >95% (assessed by LC/MS).
LC/MS (APSI) m/z [M+H] calculated for C18H14ClN2O4S2: 421.0; found: 421.0.

3-chloro-2-hydroxy-N-(3-((phenylsulfinyl)methyl)phenyl)benzenesulfonamide – Z9212925007 (Method 1).
Yield: 31%; purity, >95% (assessed by LC/MS).
LC/MS (APSI) m/z [M+H] calculated for C19H17ClNO4S2: 422.0; found: 422.0.

4-(N-(4-(3-fluorophenoxy)benzyl)sulfamoyl)-1-methyl-1H-pyrazole-5-carboxylic acid – Z9212925009 (Method 8).
Yield: 36%; purity, >95% (assessed by LC/MS).
LC/MS (APSI) m/z [M+H] calculated for C18H17FN3O5S: 406.1; found: 406.2.

4-(N-(1-(4-(3,5-dimethyl-1H-pyrazol-1-yl)phenyl)ethyl)sulfamoyl)-1-methyl-1H-pyrazole-5-carboxylic acid – Z9212925012 (Method 8).
Yield: 43%; purity, >95% (assessed by LC/MS).
LC/MS (APSI) m/z [M+H] calculated for C18H22N5O4S: 404.1; found: 404.2.

4-(N-(2-(3-(4-chlorophenyl)-6-oxopyridazin-1(6H)-yl)ethyl)sulfamoyl)-1-methyl-1H-pyrazole-5-carboxylic acid – Z9212925015 (Method 8).
Yield: 51%; purity, >95% (assessed by LC/MS).
LC/MS (APSI) m/z [M+H] calculated for C17H17ClN5O5S: 438.1; found: 438.0.

3-chloro-2-hydroxy-N-(1-(2-(2-methoxyphenyl)acetyl)piperidin-4-yl)benzenesulfonamide – Z9212925018 (Method 1).
Yield: 13%; purity, >95% (assessed by LC/MS).
LC/MS (APSI) m/z [M+H] calculated for C20H24ClN2O5S: 439.1; found: 439.0.

4-(4-((4-bromo-2-fluoro-3-methylphenyl)sulfonamido)phenyl)-4-oxobutanoic acid – Z9464814789 (Method 1).
Yield: 11%; purity, >95% (assessed by LC/MS).
LC/MS (APSI) m/z [M+H] calculated for $C_{17}H_{16}BrFNO_5S$: 444.0; found: 444.0.

2-(2-chloro-4-((4-fluoro-2-hydroxyphenyl)sulfonamido)phenoxy)acetic acid – Z9464814798 (Method 8).
Yield: 28%; purity, >95% (assessed by LC/MS).
LC/MS (APSI) m/z [M-H] calculated for $C_{14}H_{10}ClFNO_6S$: 374.0; found: 374.0.

2-(1-((3,5-dichloro-2-hydroxyphenyl)sulfonyl)-3-(3-iodo-1H-pyrazol-1-yl)azetidin-3-yl)acetic acid – Z9464814823 (Method 8).
Yield: 55%; purity, >95% (assessed by LC/MS).
LC/MS (APSI) m/z [M+H] calculated for $C_{14}H_{13}Cl_2IN_3O_5S$: 532.0; found: 532.0.

3-((3,5-dibromo-2-hydroxyphenyl)sulfonamido)-1-methylcyclopentane-1-carboxylic acid – Z9464814843 (Method 8).
Yield: 18%; purity, >95% (assessed by LC/MS).
LC/MS (APSI) m/z [M-H] calculated for $C_{13}H_{14}Br_2NO_5S$: 455.9; found: 455.8.

1-((5-oxa-6-azaspiro[3.4]oct-6-en-7-yl)methyl)-3-(3,5-dichloro-2-hydroxybenzyl)urea – Z9544921678 (Method 2).
Yield: 50%; purity, >95% (assessed by LC/MS).
LC/MS (APSI) m/z [M+H] calculated for $C_{15}H_{18}Cl_2N_3O_3$: 358.1; found: 358.0.

1-(4-(4-(3,5-dimethyl-1H-pyrazol-1-yl)phenyl)-1H-1,2,3-triazol-1-yl)cyclopropane-1-carboxylic acid – Z9544921685 (Method 11).
Yield: 30%; purity, >95% (assessed by LC/MS).
LC/MS (APSI) m/z [M+H] calculated for $C_{17}H_{18}N_5O_2$: 324.1; found: 324.2.

3-(1-(3,4-dichlorophenyl)-N,5-dimethyl-1H-pyrazole-3-carboxamido)cyclobutane-1-carboxylic acid – Z9544921693 (Method 6).
Yield: 17%; purity, >95% (assessed by LC/MS).
LC/MS (APSI) m/z [M+H] calculated for $C_{17}H_{18}Cl_2N_3O_3$: 382.1; found: 382.2.

4-(N-methylmethylsulfonamido)phenyl 3,5-dibromo-2-hydroxybenzenesulfonate – Z9544921726 (Method 1).
Yield: 37%; purity, >95% (assessed by LC/MS).
LC/MS (APSI) m/z [M-H] calculated for $C_{14}H_{12}Br_2NO_6S_2$: 513.8; found: 513.8.

3-chloro-2-hydroxy-N-(2-(5-methyl-4-phenylthiazol-2-yl)ethyl)benzenesulfonamide – Z9544921729 (Method 1).
Yield: 63%; purity, >95% (assessed by LC/MS).
LC/MS (APSI) m/z [M+H] calculated for $C_{18}H_{18}ClN_2O_3S_2$: 409.1; found: 409.0.

3-fluoro-2-hydroxy-6-methyl-N-(4-(phenylthio)benzyl)benzamide – Z9544921741 (Method 4).
Yield: 22%; purity, 92% (assessed by LC/MS).
LC/MS (APSI) m/z [M+H] calculated for $C_{21}H_{19}FNO_2S$: 368.1; found: 368.0.

3-({[6-(2-methylphenoxy)pyridin-3-yl]methyl}sulfamoyl)pyridine-2-carboxylic acid – Z9544921759 (Method 8).
Yield: 23%; purity, >95% (assessed by LC/MS).
LC/MS (APSI) m/z [M+H] calculated for $C_{19}H_{18}N_3O_5S$: 400.1; found: 400.0.

2-(1-{4-[(carbamoylamino)sulfonyl]phenyl}-N-propylformamido)-N,N-dimethylacetamide – Z9544921762 (Method 4).
Yield: 50%; purity, >95% (assessed by LC/MS).
LC/MS (APSI) m/z [M+H] calculated for $C_{15}H_{23}N_4O_5S$: 371.1; found: 371.0.

1-[(3,5-dichloro-2-hydroxyphenyl)methyl]-3-{[5-(ethylsulfanyl)-4-methyl-4H-1,2,4-triazol-3-yl]methyl}urea – Z9544921788 (Method 2).
Yield: 19%; purity, 92% (assessed by LC/MS).
LC/MS (APSI) m/z [M+H] calculated for $C_{14}H_{18}Cl_2N_5O_2S$: 390.1; found: 390.0.

N-[3,5-bis(trifluoromethyl)phenyl]-3,5-difluoro-2-hydroxybenzene-1-sulfonamide – Z9544921801 (Method 1).
Yield: 19%; purity, >95% (assessed by LC/MS).
LC/MS (APSI) m/z [M+H] calculated for $C_{14}H_8F_8NO_3S$: 422.0; found: 421.8.

N-(6-bromo-3-oxo-3,4-dihydro-2H-1,4-benzoxazin-7-yl)-3-chloro-2-hydroxybenzene-1-sulfonamide – Z9544921818 (Method 1).
Yield: 11%; purity, >95% (assessed by LC/MS).
LC/MS (APSI) m/z [M+H] calculated for $C_{14}H_{11}BrClN_2O_5S$: 435.0; found: 434.8.

5-{1-[3-(1-benzyl-1H-1,2,3-triazol-4-yl)azetidine-1-carbonyl]cyclobutyl}-2H-1,2,3,4-tetrazole – Z9544921851 (Method 4).
Yield: 51%; purity, >95% (assessed by LC/MS).
LC/MS (APSI) m/z [M+H] calculated for $C_{18}H_{21}N_8O$: 365.2; found: 365.0.

2-[4-(2,4,6-trifluoro-3-hydroxybenzoyl)piperazin-1-yl]benzene-1-sulfonamide – Z9544921860 (Method 4).
Yield: 29%; purity, >95% (assessed by LC/MS).
LC/MS (APSI) m/z [M+H] calculated for $C_{17}H_{17}F_3N_3O_4S$: 416.1; found: 416.2.

3-(5-cyclopropyl-4-phenyl-4H-1,2,4-triazol-3-yl)-N-[(3,5-dichloro-2-hydroxyphenyl)methyl] propanamide – Z9544921884 (Method 4).
Yield: 25%; purity, >95% (assessed by LC/MS).
LC/MS (APSI) m/z [M+H] calculated for $C_{21}H_{21}Cl_2N_4O_2$: 431.1; found: 431.0.

3-[(3,5-dichloro-2-hydroxyphenyl)methyl]-1-{[1-(1,3-thiazol-2-yl)piperidin-3-yl]methyl}urea – Z9544921929 (Method 2).
Yield: 36%; purity, >95% (assessed by LC/MS).
LC/MS (APSI) m/z [M+H] calculated for $C_{17}H_{21}Cl_2N_4O_2S$: 415.1; found: 415.0.

3-[1-(1H-1,3-benzodiazol-5-yl)ethyl]-1-[(3,5-dichloro-2-hydroxyphenyl)methyl]urea – Z9544921938 (Method 2).
Yield: 34%; purity, >95% (assessed by LC/MS).
LC/MS (APSI) m/z [M-H] calculated for $C_{17}H_{15}Cl_2N_4O_2$: 377.1; found: 377.0.

N-[(1S,3R)-3-[(3-bromo-5-chloro-2-hydroxyphenyl)carbamoyl]cyclopentyl]furan-2-carboxamide – Z9544921944 (Method 4).
Yield: 35%; purity, >95% (assessed by LC/MS).
LC/MS (APSI) m/z [M+H] calculated for C17H17BrClN2O4: 427.0; found: 427.0.

N-(3-bromo-5-fluoro-2-hydroxyphenyl)-1-(3-fluorophenyl)-1H-imidazole-5-carboxamide – Z9544921971 (Method 4).
Yield: 20%; purity, >95% (assessed by LC/MS).
LC/MS (APSI) m/z [M+H] calculated for C16H11BrF2N3O2: 396.0; found: 396.0.

methyl 3-(cyclohex-3-en-1-yl)-2-(2,3,5-trichloro-6-hydroxybenzenesulfonamido)propanoate – Z9544921974 (Method 1).
Yield: 25%; purity, >95% (assessed by LC/MS).
LC/MS (APSI) m/z [M+H] calculated for C16H19Cl3NO5S: 442.0; found: 442.0.

1-benzyl-N-(5-bromo-3-hydroxypyridin-2-yl)-1H-1,3-benzodiazole-5-carboxamide – Z9544921992 (Method 4).
Yield: 15%; purity, >95% (assessed by LC/MS).
LC/MS (APSI) m/z [M+H] calculated for C20H16BrN4O2: 425.0; found: 425.0.

4-[(3-chloro-1,2-thiazol-4-yl)formamido]-N-(3-chloro-2-hydroxyphenyl)-3-methylbut-2-enamide – Z9544922022 (Method 4).
Yield: 13%; purity, 92% (assessed by LC/MS).
LC/MS (APSI) m/z [M+H] calculated for C15H14Cl2N3O3S: 386.0; found: 386.0.

3-[(2-{[2-(trifluoromethyl)phenyl]formamido}ethyl)sulfamoyl]furan-2-carboxylic acid – Z9544922024 (Method 8).
Yield: 40%; purity, >95% (assessed by LC/MS).
LC/MS (APSI) m/z [M+H] calculated for C15H14F3N2O6S: 407.1; found: 407.2.

N-(4-bromo-3-hydroxypyridin-2-yl)-4-[(2-ethoxyphenyl)formamido]butanamide – Z9544922050 (Method 4).
Yield: 31%; purity, >95% (assessed by LC/MS).
LC/MS (APSI) m/z [M+H] calculated for C18H21BrN3O4: 422.1; found: 422.0.

ethyl 2-{[(3,5-dichloro-2-hydroxyphenyl)methyl]amino}-1,3-thiazole-4-carboxylate – Z9544922070 (Method 9).
Yield: 20%; purity, >95% (assessed by LC/MS).
LC/MS (APSI) m/z [M+H] calculated for C13H13Cl2N2O3S: 347.0; found: 347.0.

N-{1-[(2,4-difluorophenyl)methyl]-6-oxo-1,6-dihydropyridin-3-yl}-3,5-difluoro-2-hydroxybenzene-1-sulfonamide – Z9544922097 (Method 1).
Yield: 28%; purity, >95% (assessed by LC/MS).
LC/MS (APSI) m/z [M+H] calculated for C18H13F4N2O4S: 429.1; found: 429.2.

5-bromo-3-chloro-N-[2-(4-fluorophenyl)propyl]-2-hydroxybenzene-1-sulfonamide – Z9544922098 (Method 1).
Yield: 42%; purity, >95% (assessed by LC/MS).
LC/MS (APSI) m/z [M-H] calculated for C15H15BrClFNO3S: 422.0; found: 422.0.

3,5-dibromo-N-{3-chloroimidazo[1,2-a]pyridin-6-yl}-2-hydroxybenzene-1-sulfonamide – Z9544922102 (Method 1).
Yield: 24%; purity, >95% (assessed by LC/MS).
LC/MS (APSI) m/z [M+H] calculated for $C_{13}H_9Br_2ClN_3O_3S$: 483.8; found: 483.8.

2-methyl-5-{methyl[3-(phenylcarbamoyl)propyl]carbamoyl}-4H-thieno[3,2-b]pyrrole-3-carboxylic acid – Z9544922108 (Method 6).
Yield: 44%; purity, >95% (assessed by LC/MS).
LC/MS (APSI) m/z [M+H] calculated for $C_{20}H_{22}N_3O_4S$: 400.1; found: 400.0.

N-(5-bromo-2,3-dihydro-1H-inden-1-yl)-2-hydroxybenzene-1-sulfonamide – Z9544922110 (Method 1).
Yield: 13%; purity, >95% (assessed by LC/MS).
LC/MS (APSI) m/z [M-H] calculated for $C_{15}H_{13}BrNO_3S$: 366.0; found: 366.0

1-(2-cyanoethyl)-3,5-dimethyl-1H-pyrazol-4-yl 3-chloro-2-hydroxybenzene-1-sulfonate – Z9544922113 (Method 1).
Yield: 11%; purity, >95% (assessed by LC/MS).
LC/MS (APSI) m/z [M+H] calculated for $C_{14}H_{15}ClN_3O_4S$: 356.0; found: 356.0.

3-methyl-1-(4-phenoxybenzenesulfonyl)piperidine-3-carboxylic acid – Z9544922116 (Method 7).
Yield: 57%; purity, >95% (assessed by LC/MS).
LC/MS (APSI) m/z [M+H] calculated for $C_{19}H_{22}NO_5S$: 376.1; found: 376.2.

[5-chloro-1-(propan-2-yl)-1H-1,3-benzodiazol-2-yl]methyl 3-(5-oxo-4,5-dihydro-1H-1,2,3,4-tetrazol-1-yl)benzoate – Z9544922119 (Method 11).
Yield: 14%; purity, >95% (assessed by LC/MS).
LC/MS (APSI) m/z [M+H] calculated for $C_{19}H_{18}ClN_6O_3$: 413.1; found: 413.2.

N-(3,5-dibromo-2-hydroxyphenyl)-3-[(1-methyl-1H-1,2,3,4-tetrazol-5-yl)sulfanyl]propanamide – Z9544922126 (Method 4).
Yield: 24%; purity, >95% (assessed by LC/MS).
LC/MS (APSI) m/z [M+H] calculated for $C_{11}H_{12}Br_2N_5O_2S$: 437.9; found: 437.8.

2-{4-[(2-hydroxybenzenesulfonamido)methyl]phenyl}acetamide – Z9544922135 (Method 1).
Yield: 13%; purity, >95% (assessed by LC/MS).
LC/MS (APSI) m/z [M+H] calculated for $C_{15}H_{17}N_2O_4S$: 321.1; found: 321.2.

3-{8-bromoimidazo[1,2-a]pyridin-3-yl}-N-(3,5-dichloro-2-hydroxyphenyl)propanamide – Z9544922144 (Method 4).
Yield: 21%; purity, >95% (assessed by LC/MS).
LC/MS (APSI) m/z [M+H] calculated for $C_{16}H_{13}BrCl_2N_3O_2$: 430.0; found: 430.0.

3,5-dichloro-N-[(3,5-dimethyl-4-oxo-1,4-dihydropyridin-2-yl)methyl]-2-hydroxybenzene-1-sulfonamide – Z9544922186 (Method 1).
Yield: 34%; purity, >95% (assessed by LC/MS).
LC/MS (APSI) m/z [M+H] calculated for $C_{14}H_{15}Cl_2N_2O_4S$: 377.0; found: 377.0.

2-methyl-1-({[3-(naphthalen-1-yloxy)phenyl]methyl}carbamoyl)cyclopropane-1-carboxylic acid – Z9544922231 (Method 6).
Yield: 31%; purity, >95% (assessed by LC/MS).
LC/MS (APSI) m/z [M+H] calculated for C23H22NO4: 376.2; found: 376.2.

2-bromo-N-[(3,5-dichloro-2-hydroxyphenyl)methyl]-4-(dimethylsulfamoyl)benzamide – Z9544922262 (Method 4).
Yield: 33%; purity, >95% (assessed by LC/MS).
LC/MS (APSI) m/z [M+H] calculated for C16H16BrCl2N2O4S: 482.9; found: 483.0.

N-(5-bromo-3-chloro-2-hydroxyphenyl)-3-(3-oxo-2,3-dihydro-1,2-benzothiazol-2-yl)propanamide – Z9544922281 (Method 4).
Yield: 30%; purity, >95% (assessed by LC/MS).
LC/MS (APSI) m/z [M+H] calculated for C16H13BrClN2O3S: 429.0; found: 428.8.

4-benzenesulfonamidophenyl 3,5-dibromo-2-hydroxybenzene-1-sulfonate – Z9544922287 (Method 1).
Yield: 12%; purity, >95% (assessed by LC/MS).
LC/MS (APSI) m/z [M-H] calculated for C18H14Br2NO6S2: 561.9; found: 561.8.

1-(3-bromo-2-hydroxyphenyl)-3-(2,6-difluoro-4-methoxyphenyl)urea – Z9544922290 (Method 2).
Yield: 24%; purity, >95% (assessed by LC/MS).
LC/MS (APSI) m/z [M+H] calculated for C14H12BrF2N2O3: 374.0; found: 374.4.

3-ethyl-5-[4-(3-methoxypropanesulfonyl)piperazine-1-carbonyl]benzoic acid – Z9544922296 (Method 6).
Yield: 40%; purity, >95% (assessed by LC/MS).
LC/MS (APSI) m/z [M+H] calculated for C18H27N2O6S: 399.2; found: 399.0.

N-(6-{[(3,6-dichloro-2-hydroxyphenyl)methyl]amino}pyridin-3-yl)methanesulfonamide – Z9544922311 (Method 9).
Yield: 17%; purity, >95% (assessed by LC/MS).
LC/MS (APSI) m/z [M+H] calculated for C13H14Cl2N3O3S: 362.0; found: 362.0.

N-(3,4-difluoro-2-hydroxyphenyl)-1-methyl-4-(2-methyl-1,3-thiazol-4-yl)-1H-pyrrole-2-carboxamide – Z9544922324 (Method 4).
Yield: 17%; purity, >95% (assessed by LC/MS).
LC/MS (APSI) m/z [M+H] calculated for C16H14F2N3O2S: 350.1; found: 350.0.

3,5-difluoro-N-[2-fluoro-5-(furan-2-yl)phenyl]-2-hydroxybenzene-1-sulfonamide – Z9544922335 (Method 1).
Yield: 24%; purity, >95% (assessed by LC/MS).
LC/MS (APSI) m/z [M+Na] calculated for C16H10F3NO4SNa: 392.0; found: 392.0.

3-chloro-2-hydroxy-N-(6-{4H,5H,6H,7H-thieno[3,2-c]pyridin-5-yl}pyridin-3-yl)benzene-1-sulfonamide – Z9544922358 (Method 1).
Yield: 12%; purity, >95% (assessed by LC/MS).
LC/MS (APSI) m/z [M+H] calculated for C18H17ClN3O3S2: 422.0; found: 422.2.

3,5-difluoro-N-[1-(3-fluoro-4-phenoxyphenyl)ethyl]-2-hydroxybenzene-1-sulfonamide – Z9544922369 (Method 1).
Yield: 22%; purity, >95% (assessed by LC/MS).
LC/MS (APSI) m/z [M-H] calculated for $C_{20}H_{15}F_3NO_4S$: 422.1; found: 422.2.

3,5-dibromo-N-[(1-cyclopropyl-1H-imidazol-5-yl)methyl]-2-hydroxybenzene-1-sulfonamide – Z9544922376 (Method 1).
Yield: 58%; purity, >95% (assessed by LC/MS).
LC/MS (APSI) m/z [M+H] calculated for $C_{13}H_{14}Br_2N_3O_3S$: 451.9; found: 451.8.

methyl 7-(3,5-dibromo-2-hydroxybenzenesulfonamido)spiro[3.5]nonane-2-carboxylate – Z9544922381 (Method 1).
Yield: 22%; purity, >95% (assessed by LC/MS).
LC/MS (APSI) m/z [M-H] calculated for $C_{17}H_{22}Br_2NO_5S$: 510.0; found: 510.0.

tert-butyl 4-[2-(3,5-dichloro-2-hydroxybenzenesulfonamido)ethyl]-4-methylpiperidine-1-carboxylate – Z9544922391 (Method 1).
Yield: 17%; purity, >95% (assessed by LC/MS).
LC/MS (APSI) m/z [M-H] calculated for $C_{19}H_{29}Cl_2N_2O_5S$: 465.1; found: 465.2.

tert-butyl 4-(5-hydroxy-6-iodopyridine-3-carbonyl)-7-methyl-1,4-diazepane-1-carboxylate – Z9544922431 (Method 4).
Yield: 19%; purity, >95% (assessed by LC/MS).
LC/MS (APSI) m/z [M-H] calculated for $C_{17}H_{23}IN_3O_4$: 460.1; found: 460.2.

3-chloro-N-[4-chloro-2-fluoro-5-(trifluoromethyl)phenyl]-2-hydroxybenzene-1-sulfonamide – Z9544922461 (Method 1).
Yield: 28%; purity, >95% (assessed by LC/MS).
LC/MS (APSI) m/z [M-H] calculated for $C_{13}H_6Cl_2F_4NO_3S$: 402.0; found: 402.0.

N-(3-bromo-5-chloro-2-hydroxyphenyl)-3-(1-oxo-2,3-dihydro-1H-isoindol-2-yl)propanamide – Z9544922472 (Method 4).
Yield: 18%; purity, >95% (assessed by LC/MS).
LC/MS (APSI) m/z [M+H] calculated for $C_{17}H_{15}BrClN_2O_3$: 411.0; found: 411.0.

5-bromo-3-chloro-2-hydroxy-N-[4-(1H-1,2,4-triazol-1-yl)-3-(trifluoromethyl)phenyl]benzene-1-sulfonamide – Z9544922478 (Method 1).
Yield: 26%; purity, >95% (assessed by LC/MS).
LC/MS (APSI) m/z [M+H] calculated for $C_{15}H_{10}BrClF_3N_4O_3S$: 498.9; found: 499.0.

2-(2-{[1-(2-fluorophenyl)cyclopropyl]sulfamoyl}phenyl)acetic acid – Z9544922551 (Method 8).
Yield: 25%; purity, >95% (assessed by LC/MS).
LC/MS (APSI) m/z [M+H] calculated for $C_{17}H_{17}FNO_4S$: 350.1; found: 350.0.

3-[2-(5-chloro-1,3-thiazol-4-yl)acetyl]-N-(3-chloro-2-hydroxyphenyl)-3-azabicyclo[3.2.1]octane-6-carboxamide – Z9544957371 (Method 4).
Yield: 13%; purity, >95% (assessed by LC/MS).
LC/MS (APSI) m/z [M+H] calculated for $C_{19}H_{20}Cl_2N_3O_3S$: 440.1; found: 440.0.

tert-butyl N-[(1R)-1-{4-[(3-chloro-2-hydroxybenzenesulfonamido)methyl]phenyl}ethyl]carbamate – Z9544960567 (Method 1).

Yield: 23%; purity, >95% (assessed by LC/MS).

LC/MS (APSI) m/z [M-H] calculated for $C_{20}H_{24}ClN_2O_5S$: 439.1; found: 439.2.
