# Supplementary material for: Chemical Distance-Based Acceleration of Large Library Docking with ChemSTEP
Source: ACS Cent Sci. 2026 Jul 20;12(8):1178–94. doi: 10.1021/acscentsci.6c00396 (PMC13523501; doi:10.1021/acscentsci.6c00396)
Supplement: Supplementary file 2 [file oc6c00396_si_002.pdf]

Supplementary Information for:

Chemical Distance–based Acceleration of Large Library Docking with ChemSTEP

Olivier Mailhot[1,†,‡,*], Katie L. Holland[1,†], Lu Paris[1], Dmytro S. Radchenko[2], Yurii S. Moroz[2,3,4], John J. Irwin[1,*] & Brian K. Shoichet[1,*]

[1]Department of Pharmaceutical Chemistry, University of California, San Francisco, CA 94143-2550, USA.

[2]Enamine Ltd, Kyïv, 02094, Ukraine.

[3]Chemspace LLC, Kyïv, 02094, Ukraine.

[4]Department of Chemistry, Taras Shevchenko National University of Kyïv, Kyïv, 01601, Ukraine.

[†]Equal contribution.

[*]email: o.mailhot@umontreal.ca, jir322@gmail.com, bshoichet@gmail.com

[‡]Current affiliation: Institute for Research in Immunology and Cancer (IRIC) and Faculty of Pharmacy, Université de Montréal, Montréal, Canada.

MaxPeak: 100.00%
Ret_Time: 1.509 min

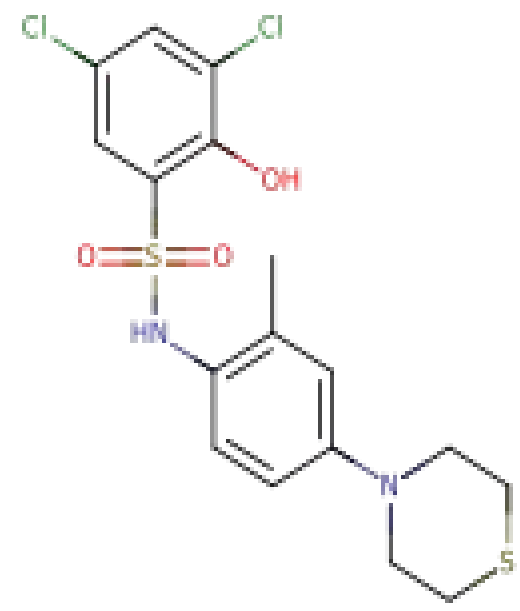

Mol Wt 433.37
Exact Mass 432.03

#	Time	Area%
1	1.509	100.00

BG917659$6

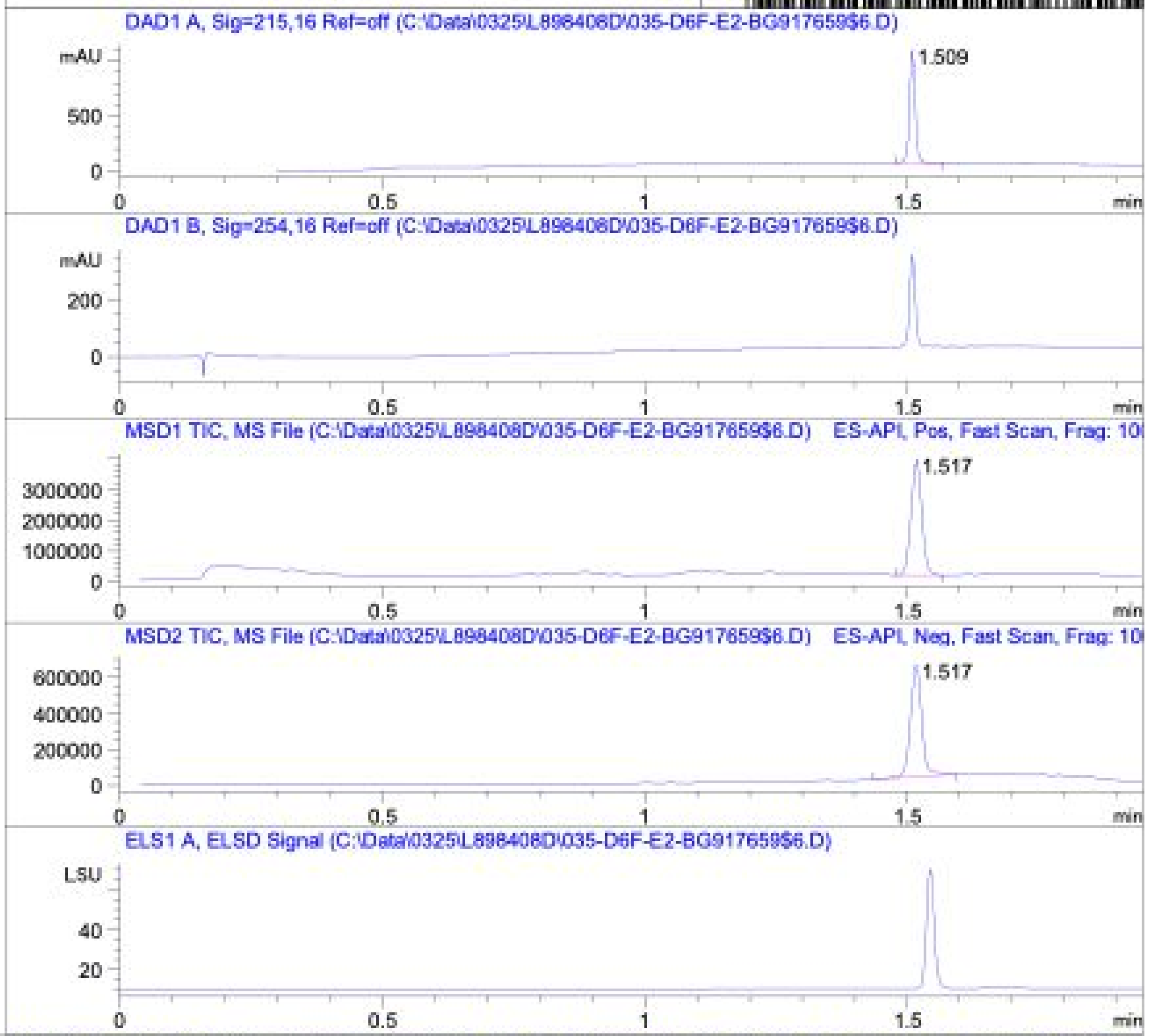

RT 1.517

RT 1.517

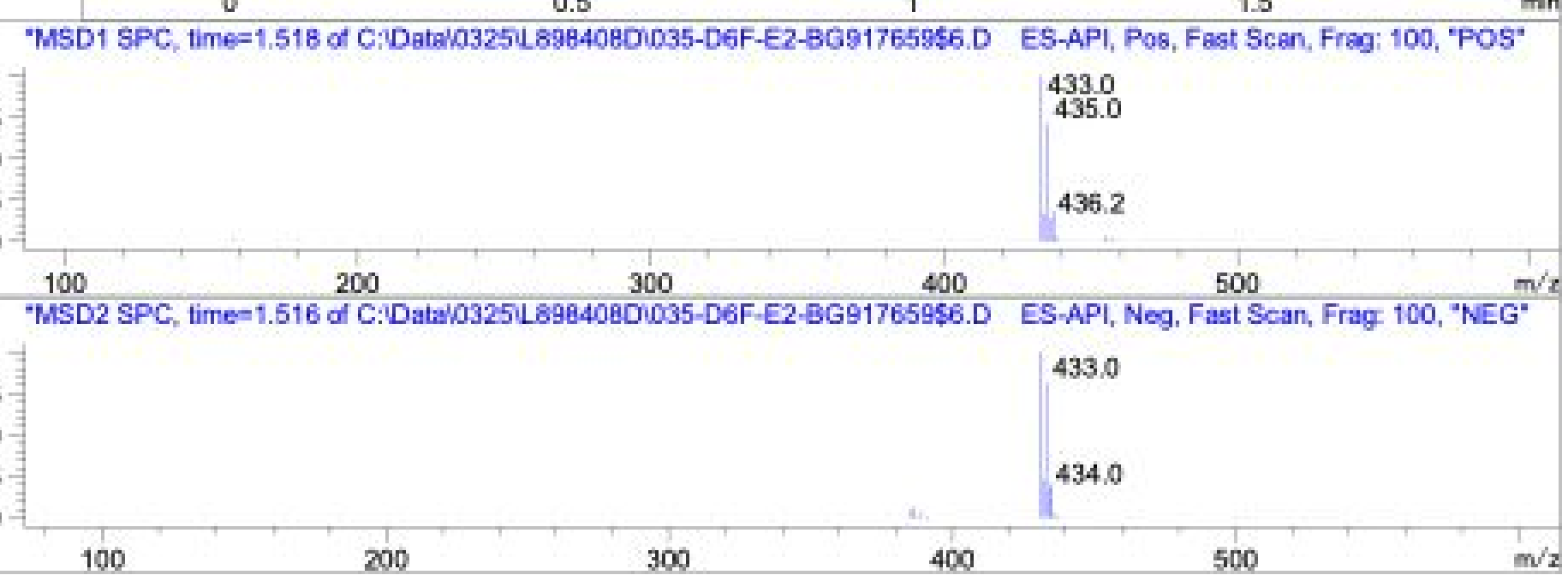

Inj.Date 3/24/2025 NEW -29-
C:\Users\Public\Documents\ChemStation\1\Data\03_24\L898408D\SUPOR_30.M

Z2940310451

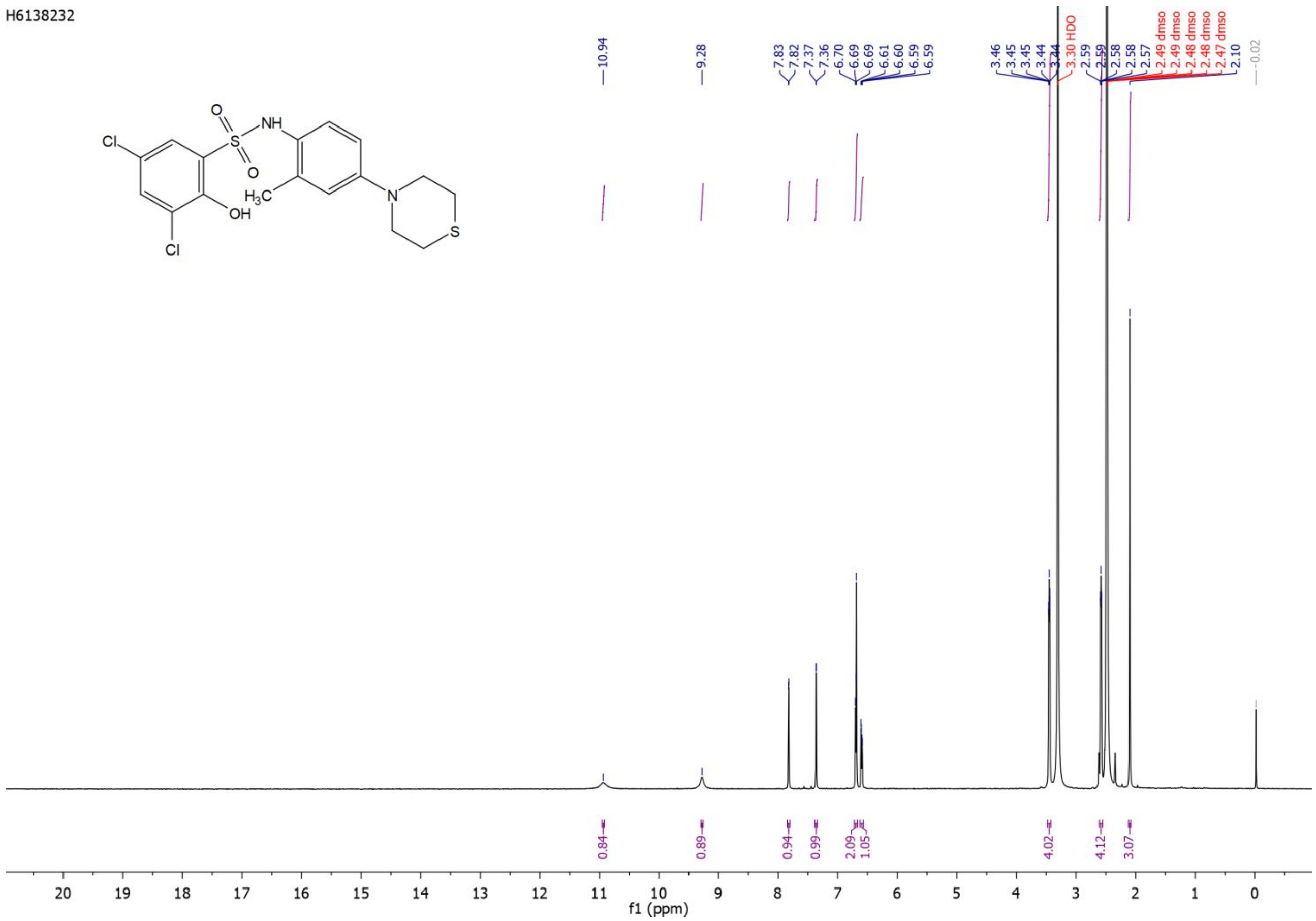

Z2940310451

MaxPeak: 100.00%
Ret_Time: 1.435 min

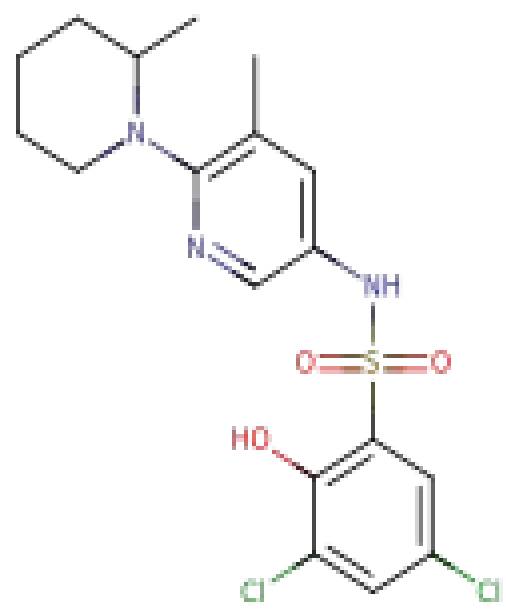

Mol Wt 430.35

Exact Mass 429.09

#	Time	Area%
1	1.435	100.00

BG917661$3

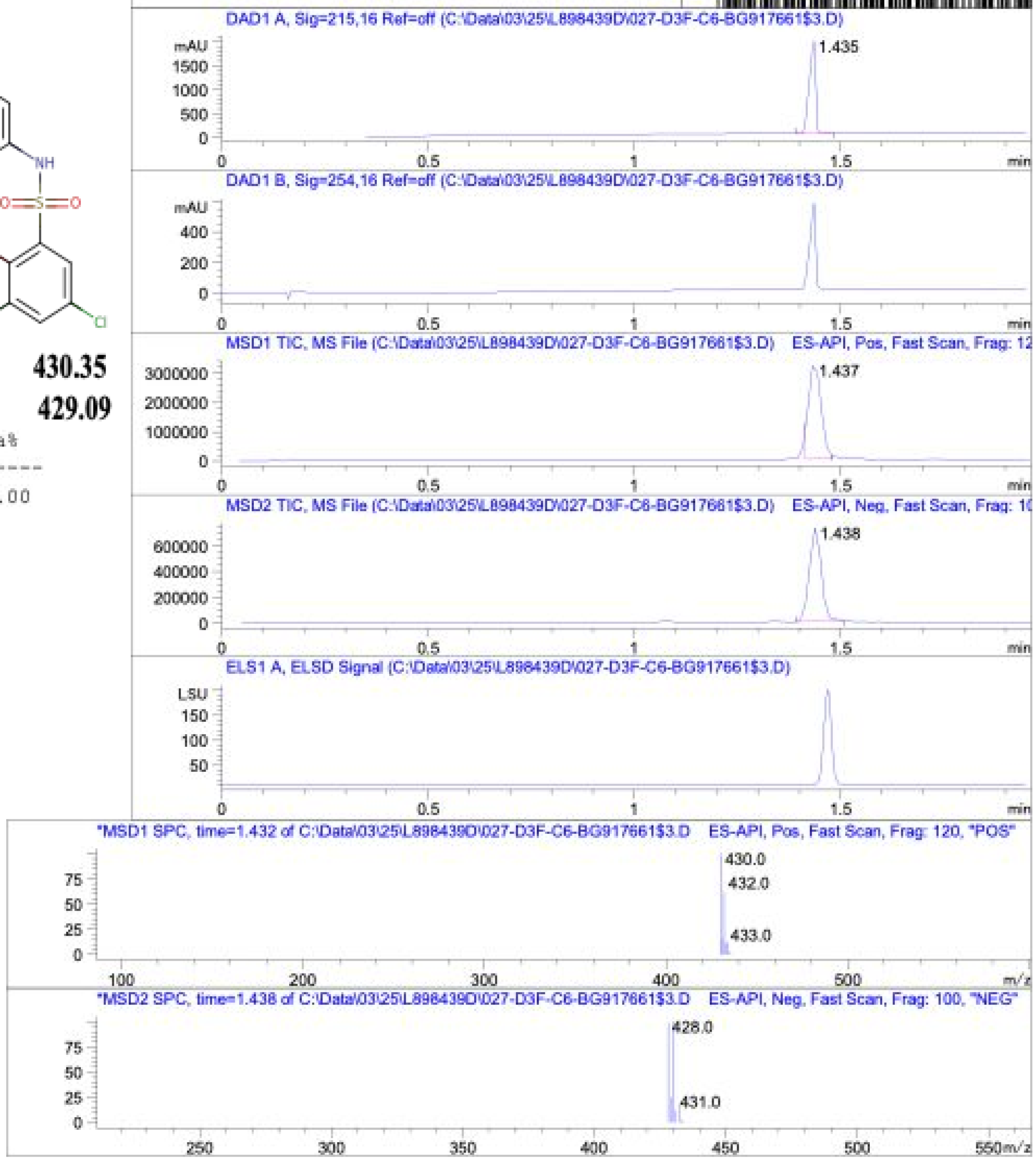

RT 1.437

RT 1.438

Inj.Date 3/24/2025 C -21-
C:\Users\Public\Documents\ChemStation\1\Data\03_24\L898439D\SUPOR_96.M

Z2940604882

H6140745

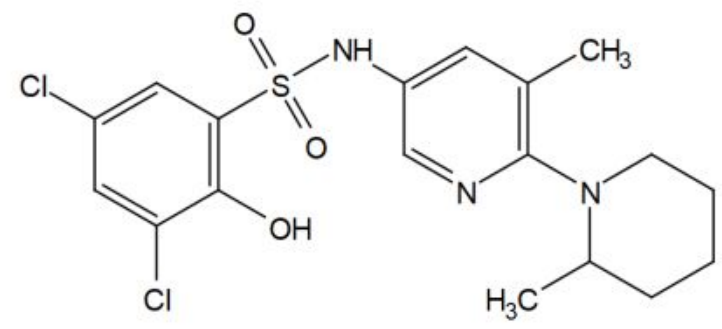

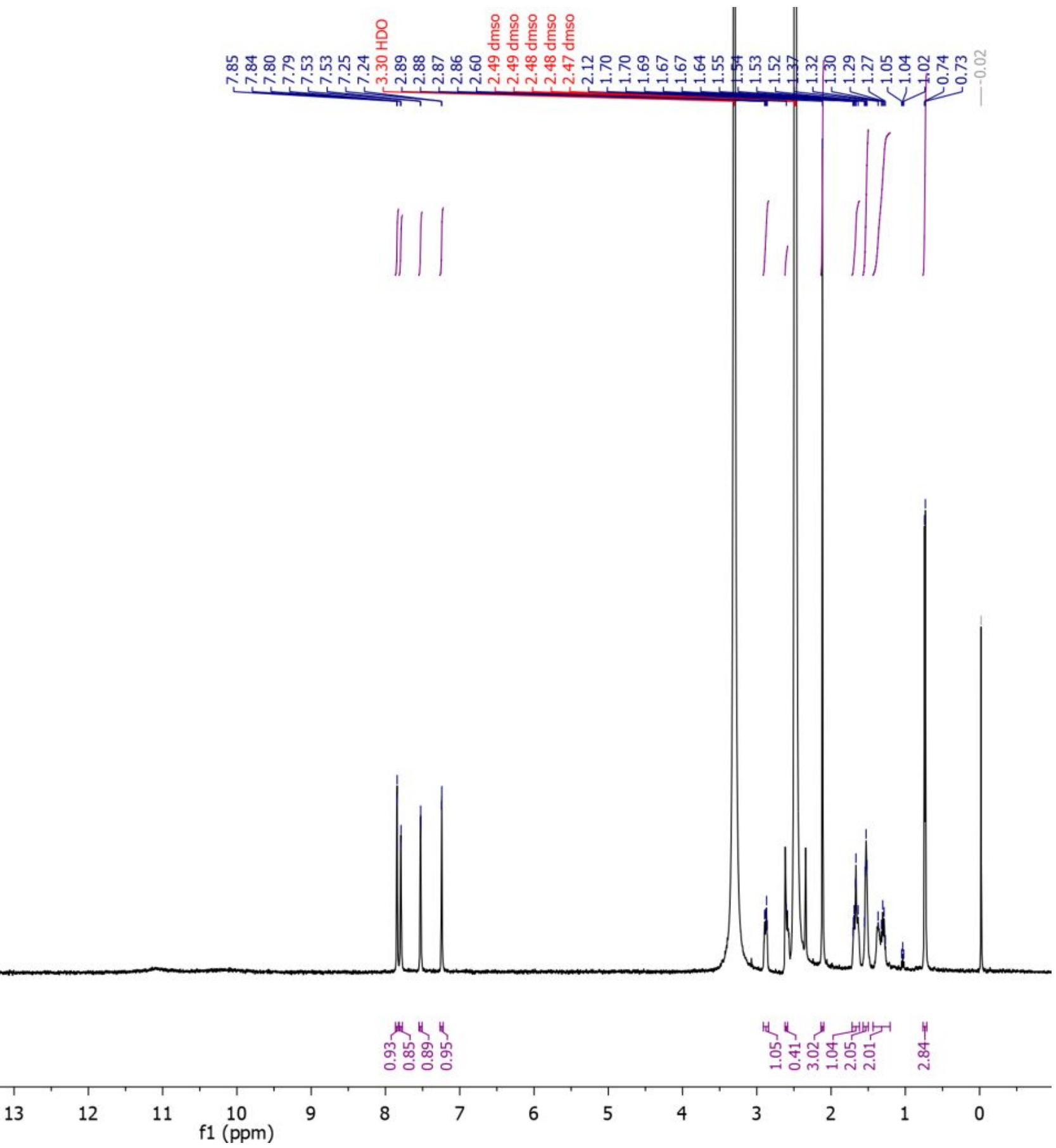

Z2940604882

MaxPeak: 100.00%
Ret_Time: 1.153 min

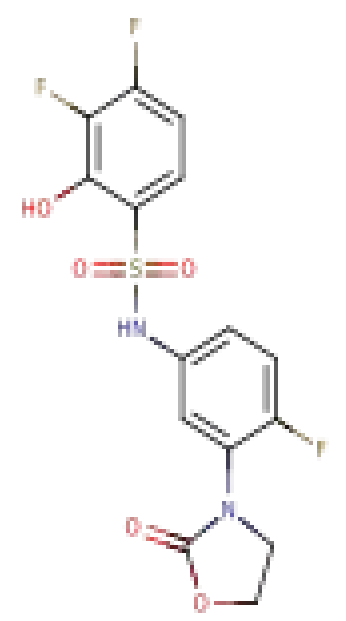

Mol Wt 388.32

Exact Mass 388.03

#	Time	Area%
1	1.153	100.00

RT 1.168

RT 1.168

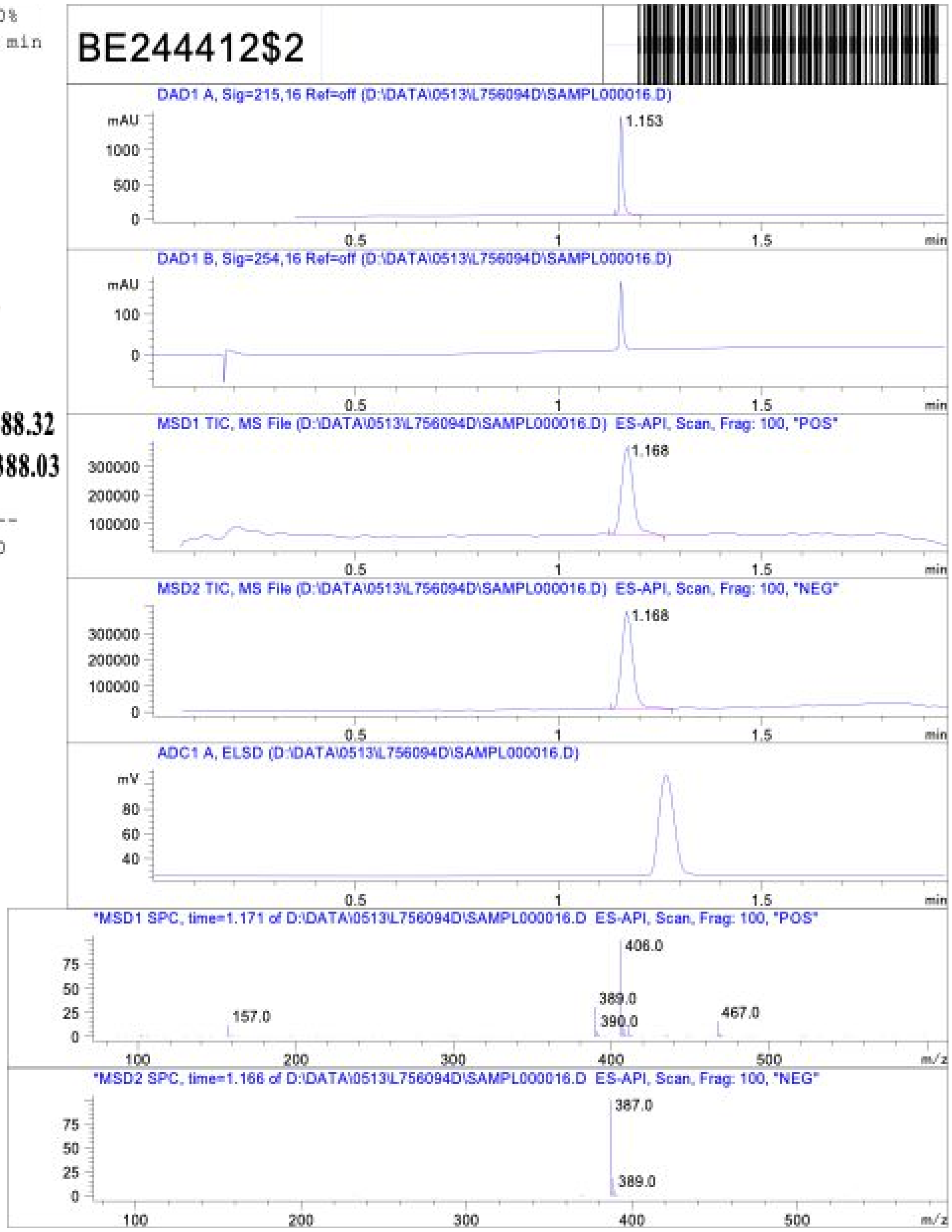

Inj.Date 5/13/2024 LB -6-
C:\CHEM32\1\METHODS\SUPOR_30.M

Z8238930017

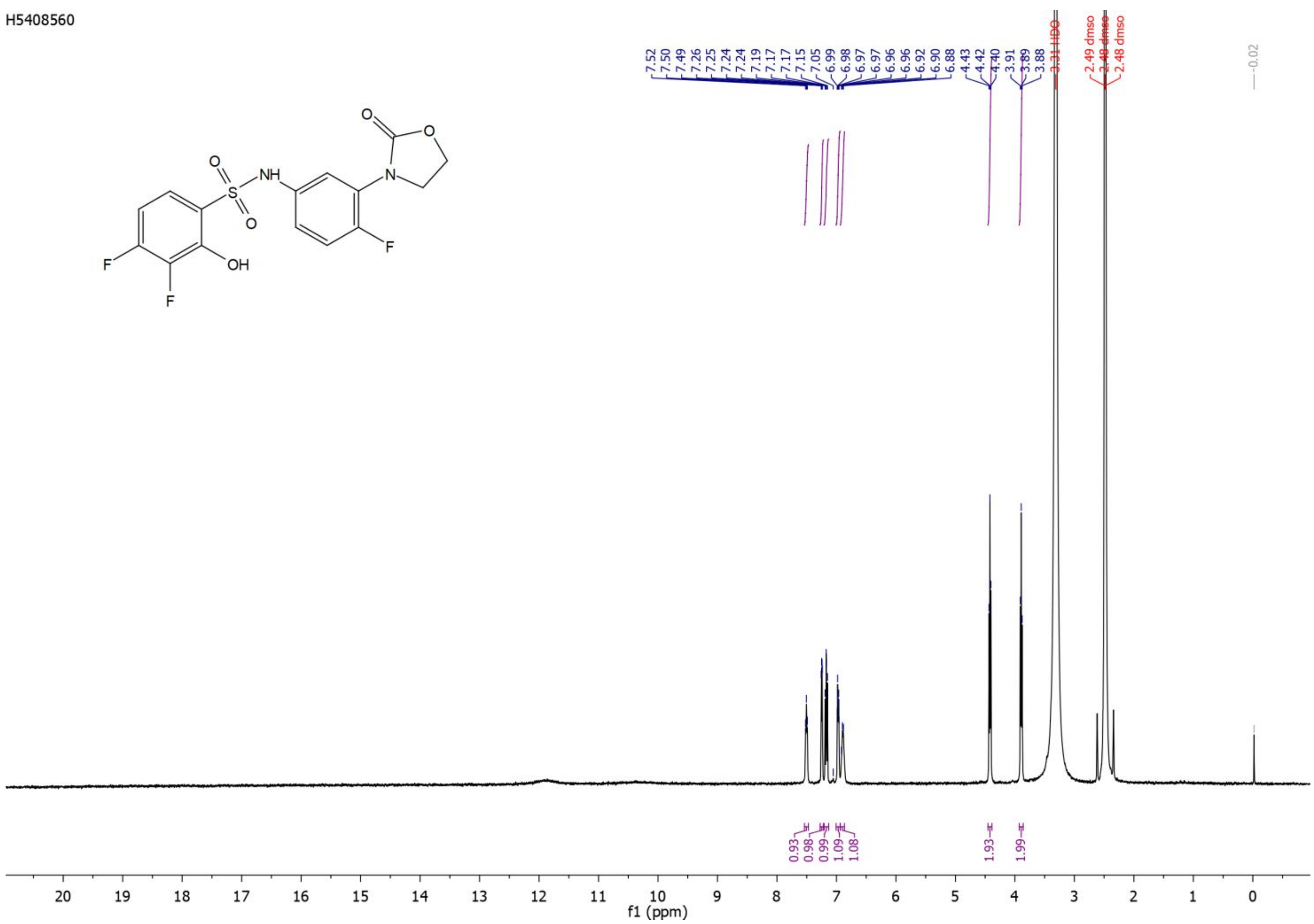

Z8238930017

MaxPeak: 100.00%
Ret_Time: 1.164 min

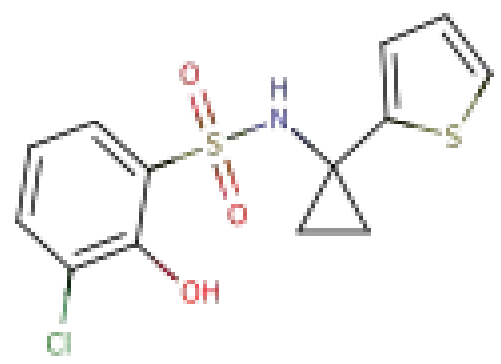

Mol Wt 329.82
Exact Mass 329

#	Time	Area%
1	1.164	100.00

BE375558$11

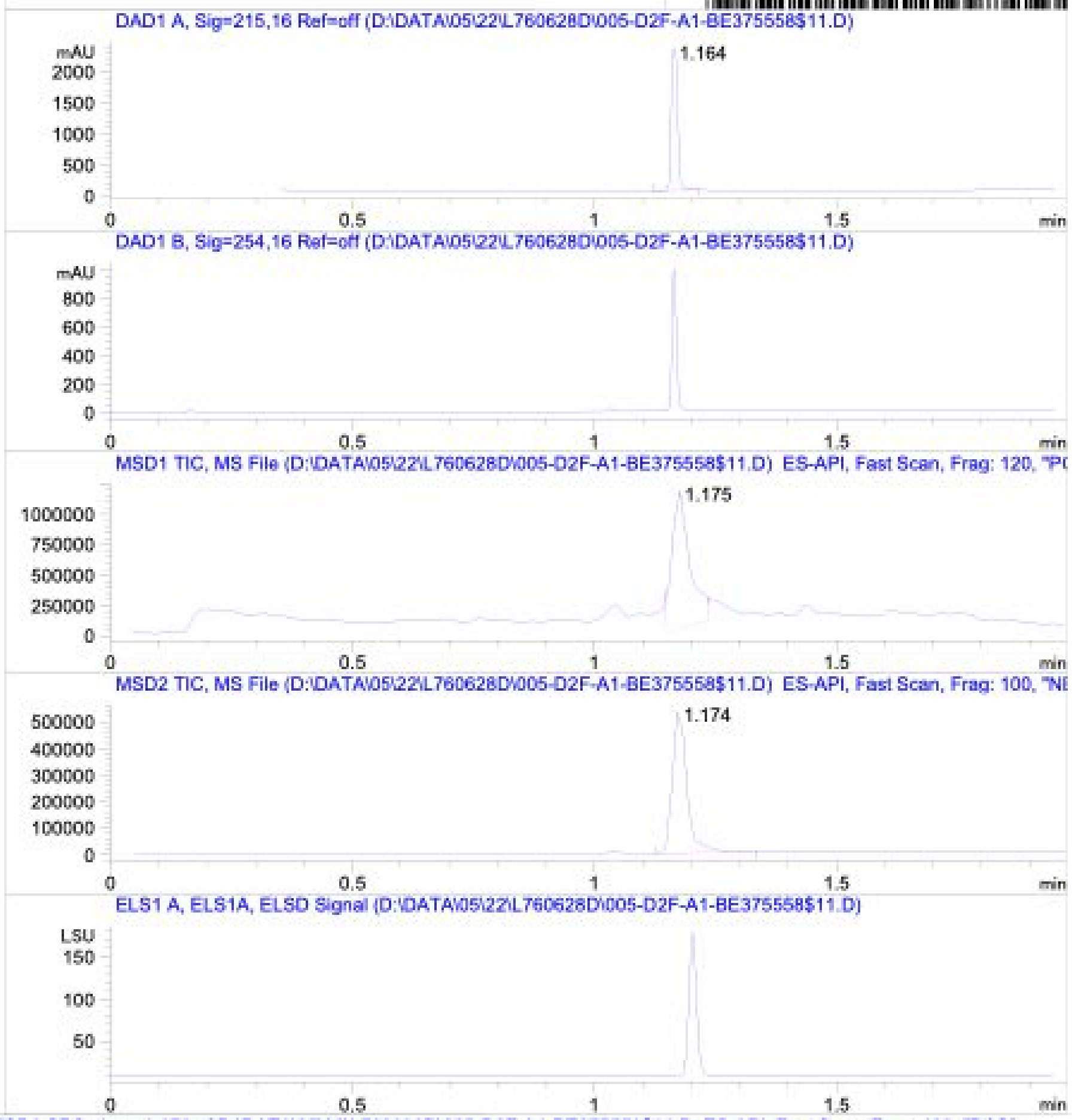

RT 1.175

RT 1.174

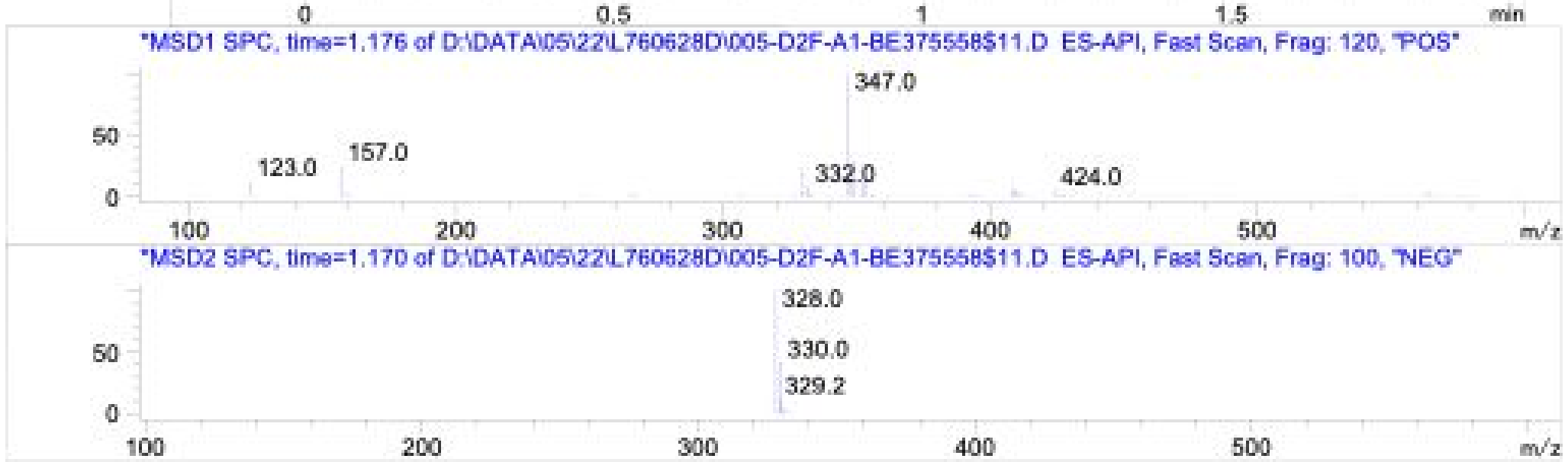

Inj.Date 5/22/2024 CH
C:\Users\Public\Documents\ChemStation\1\Data\05_22\L760628D\SUPOR_96.M

Z9103951892

H5434845

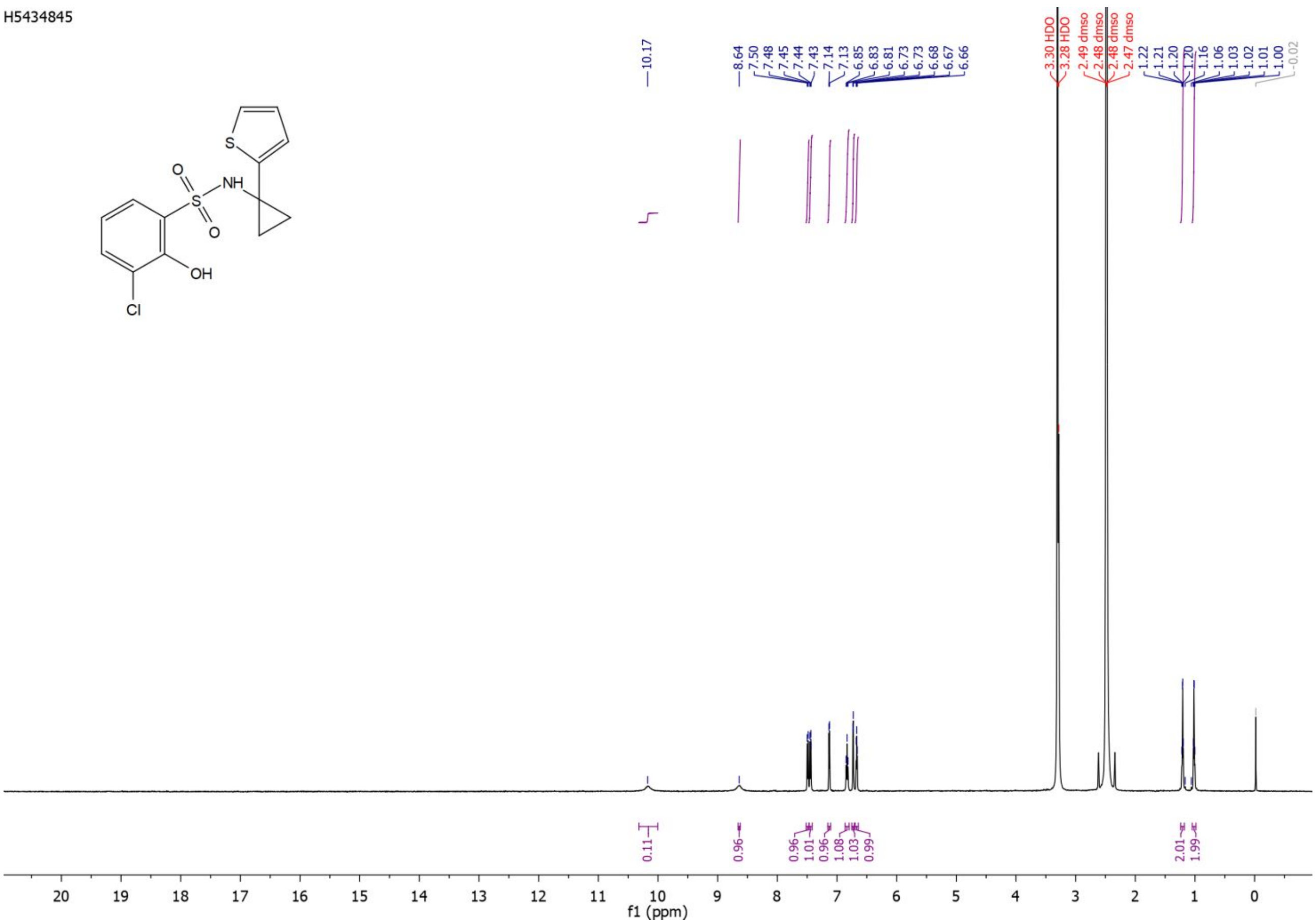

Z9103951892

MaxPeak: 100.00%
Ret_Time: 1.490 min

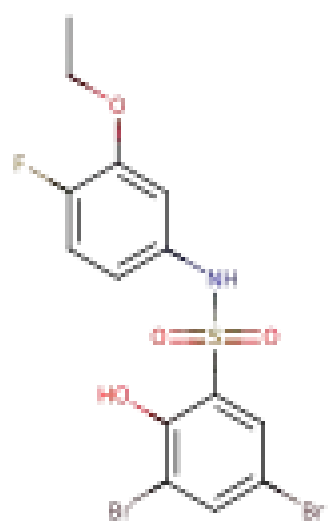

Mol Wt 469.12
Exact Mass 468.89

#	Time	Area%
1	1.490	100.00

BE244419$1

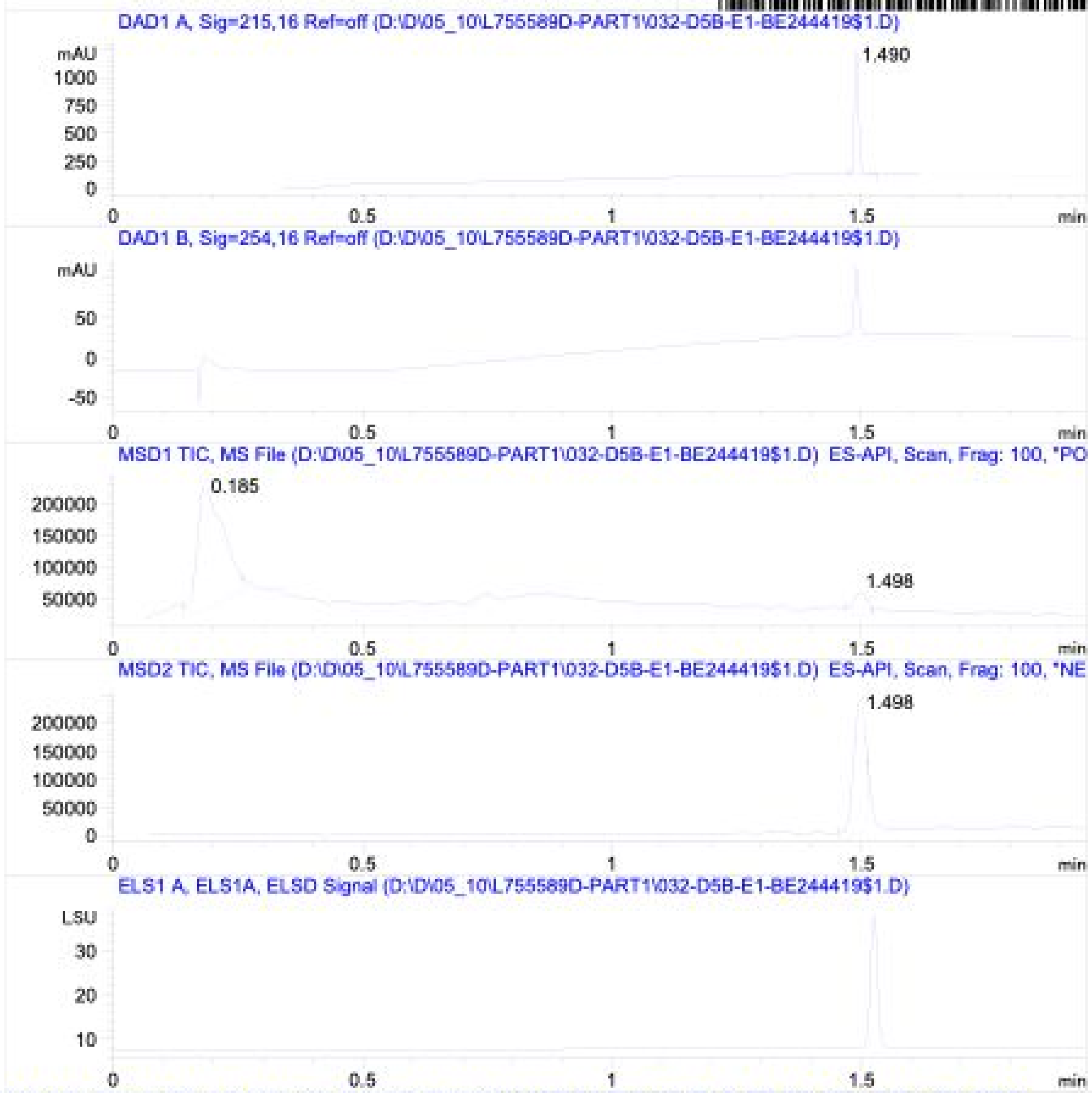

RT 0.185

RT 1.498

RT 1.498

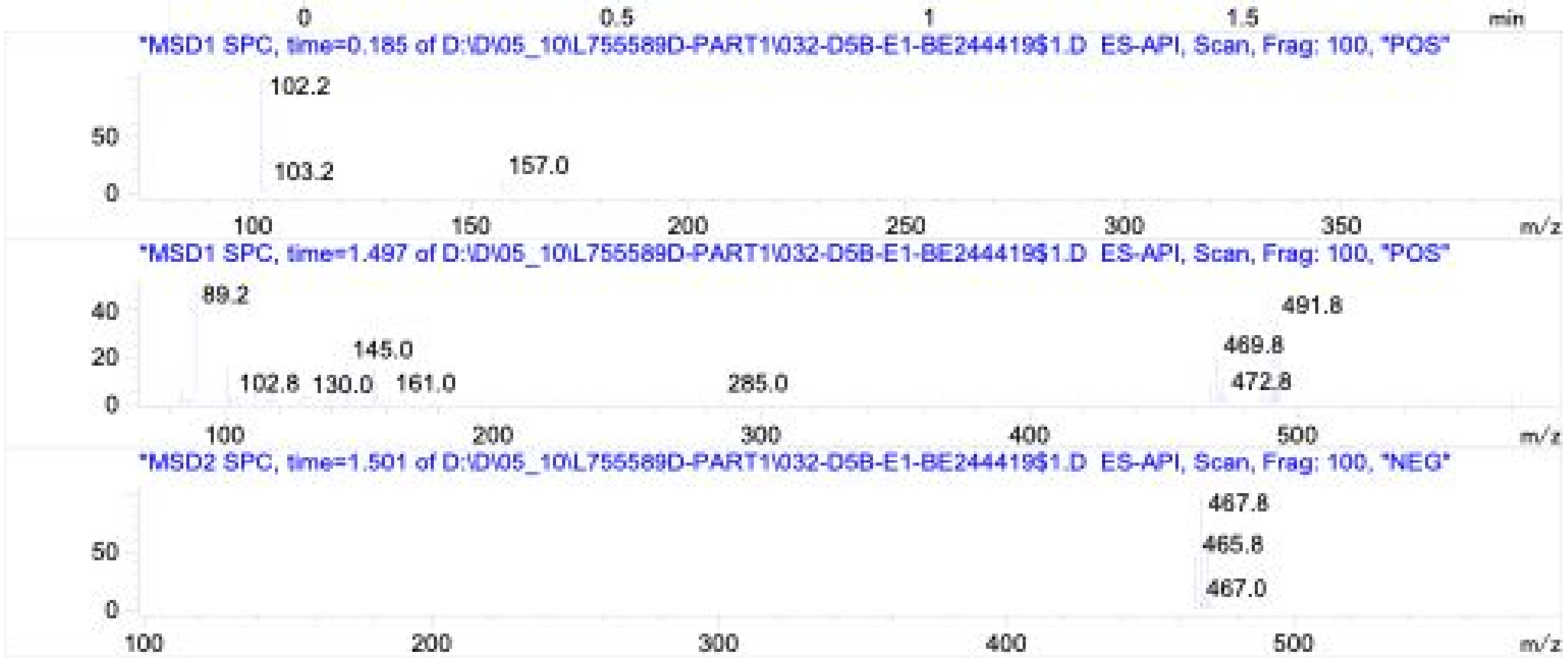

Inj.Date 5/10/2024 N -15-
C:\Chem32\1\Data\05_10\1755589D\SUPOR_30.M

Z9103951943

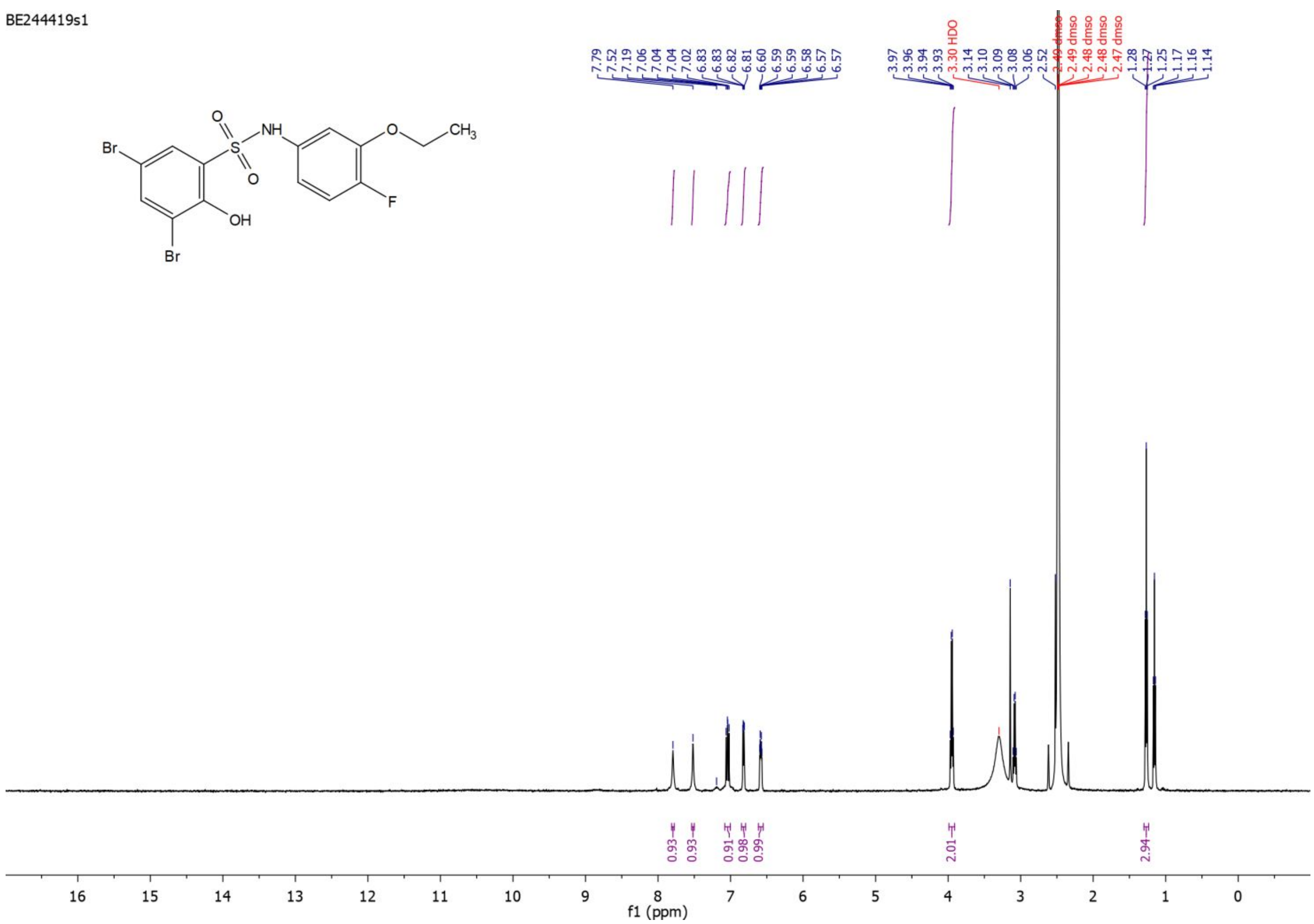

Z9103951943

MaxPeak: 100.00%
Ret_Time: 1.416 min

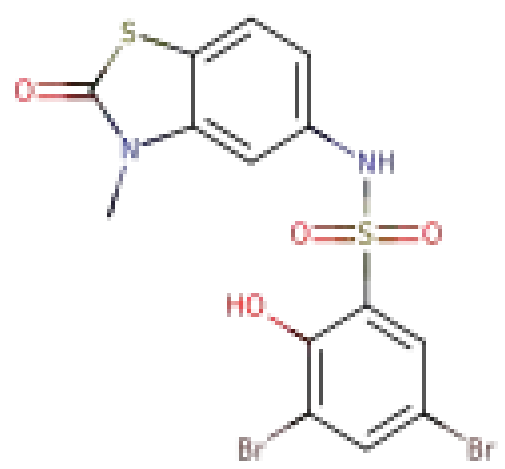

Mol Wt 494.18
Exact Mass 493.84

#	Time	Area%
1	1.416	100.00

BE244424$2

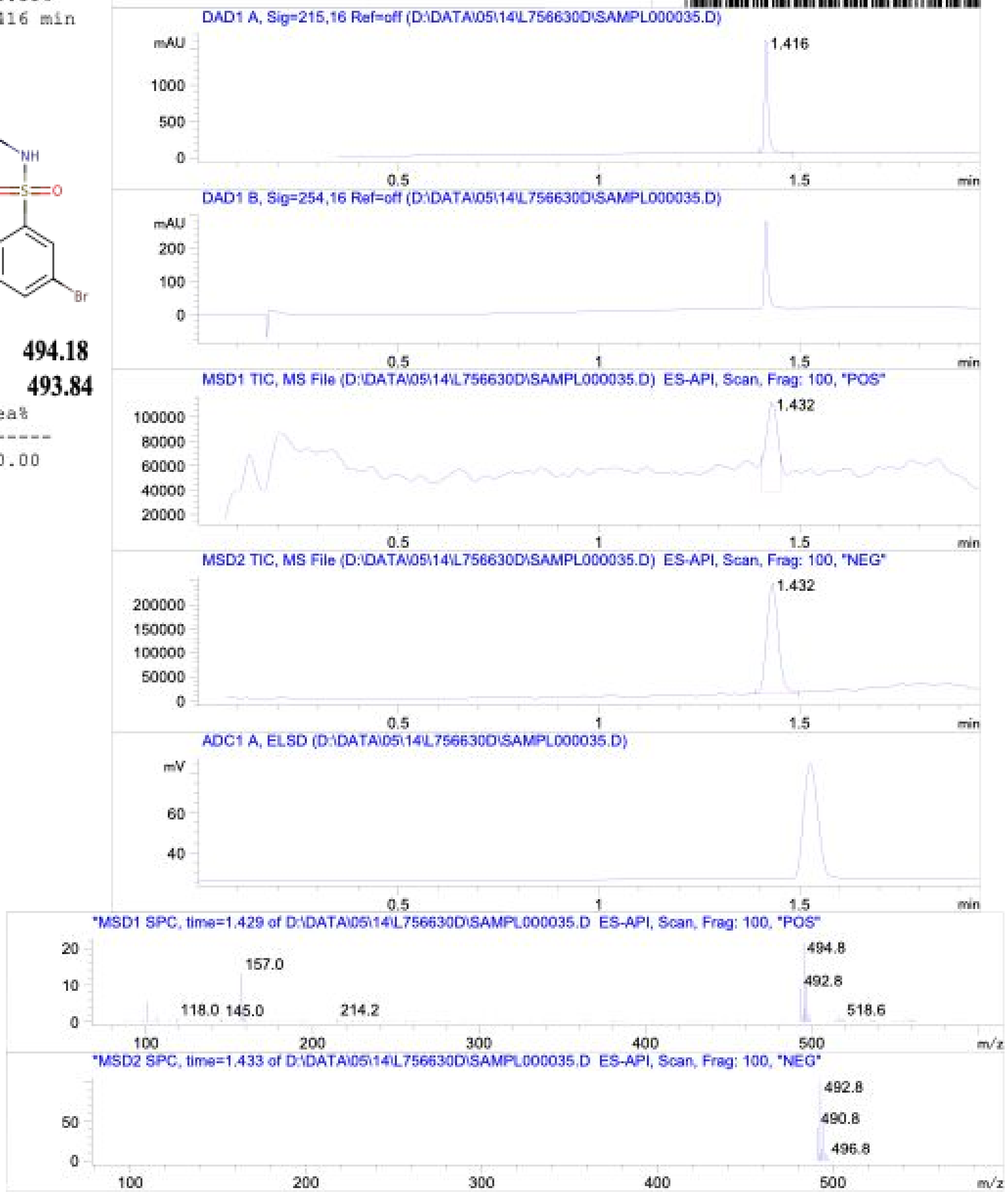

RT 1.432

RT 1.432

Inj.Date 5/14/2024 CH -6-
C:\CHEM32\1\METHODS\SUPOR_30.M

Z9103952053

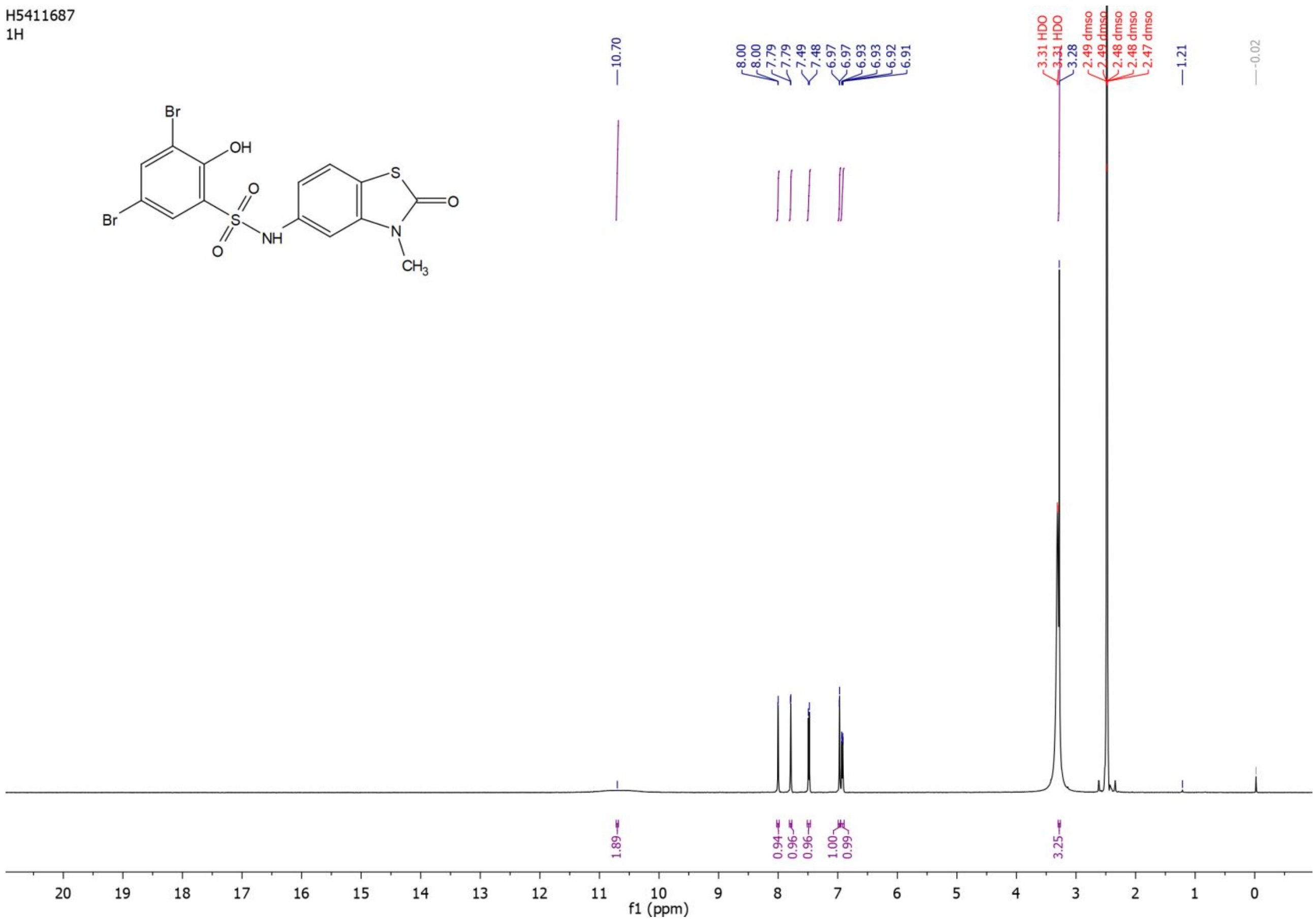

Z9103952053

MaxPeak: 98.87%
Ret_Time: 1.506 min

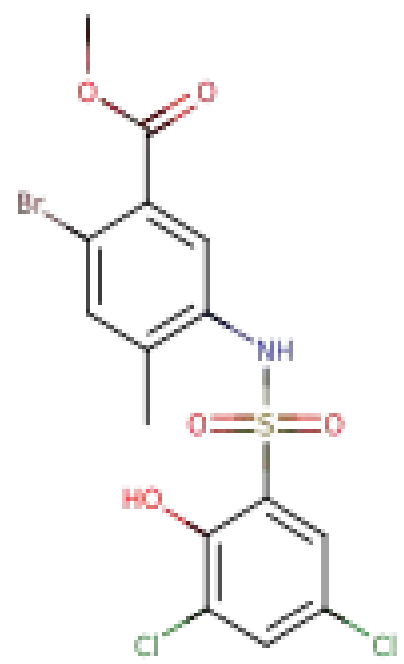

Mol Wt 469.13
Exact Mass 468.9

#	Time	Area%
1	1.220	1.13
2	1.506	98.87

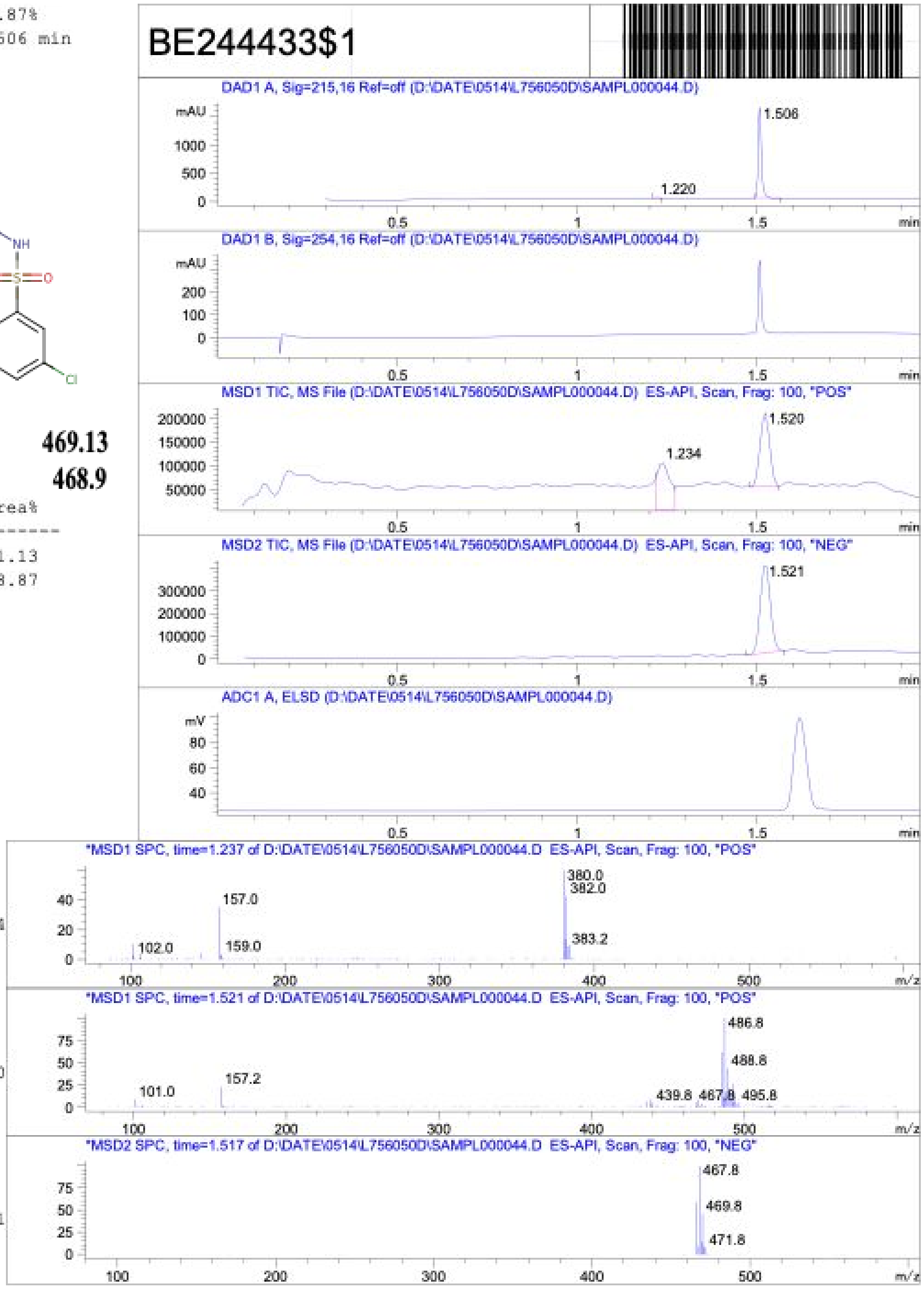

RT 1.234

RT 1.520

RT 1.521

Inj.Date 5/13/2024 CH -6-
C:\CHEM32\1\METHODS\SUPOR_30.M

Z9103952171

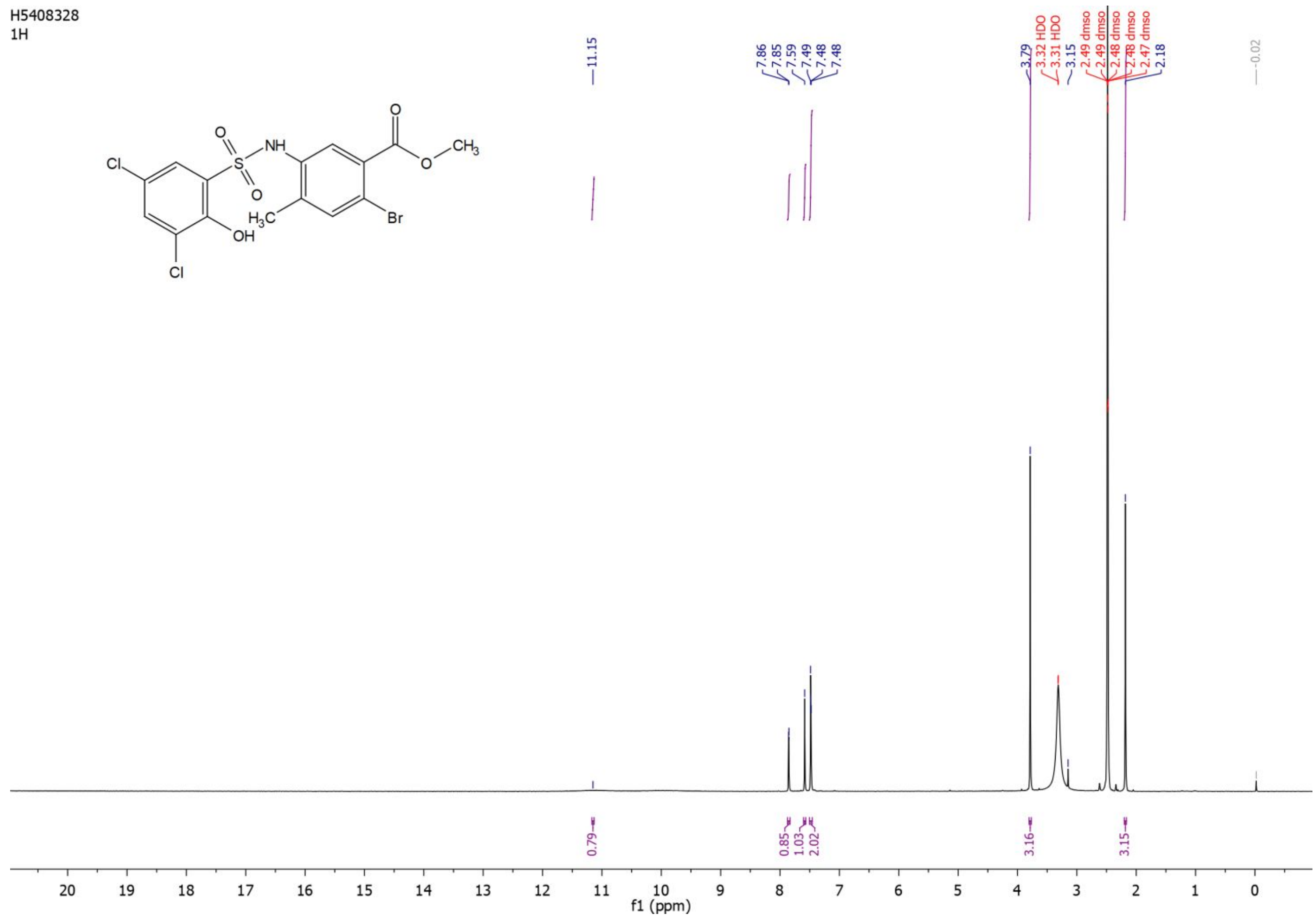

Z9103952171

MaxPeak: 97.53%
Ret_Time: 1.619 min

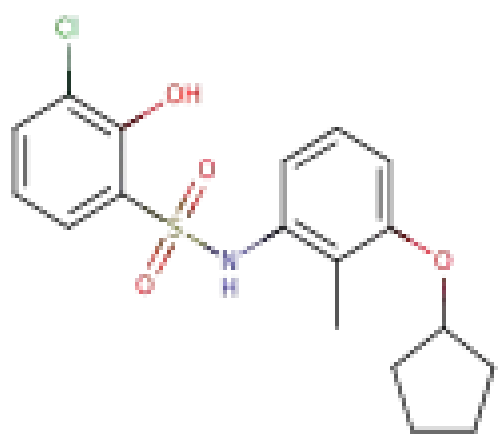

Mol Wt 381.87

Exact Mass 381.1

#	Time	Area%
1	1.619	97.53
2	1.719	2.47

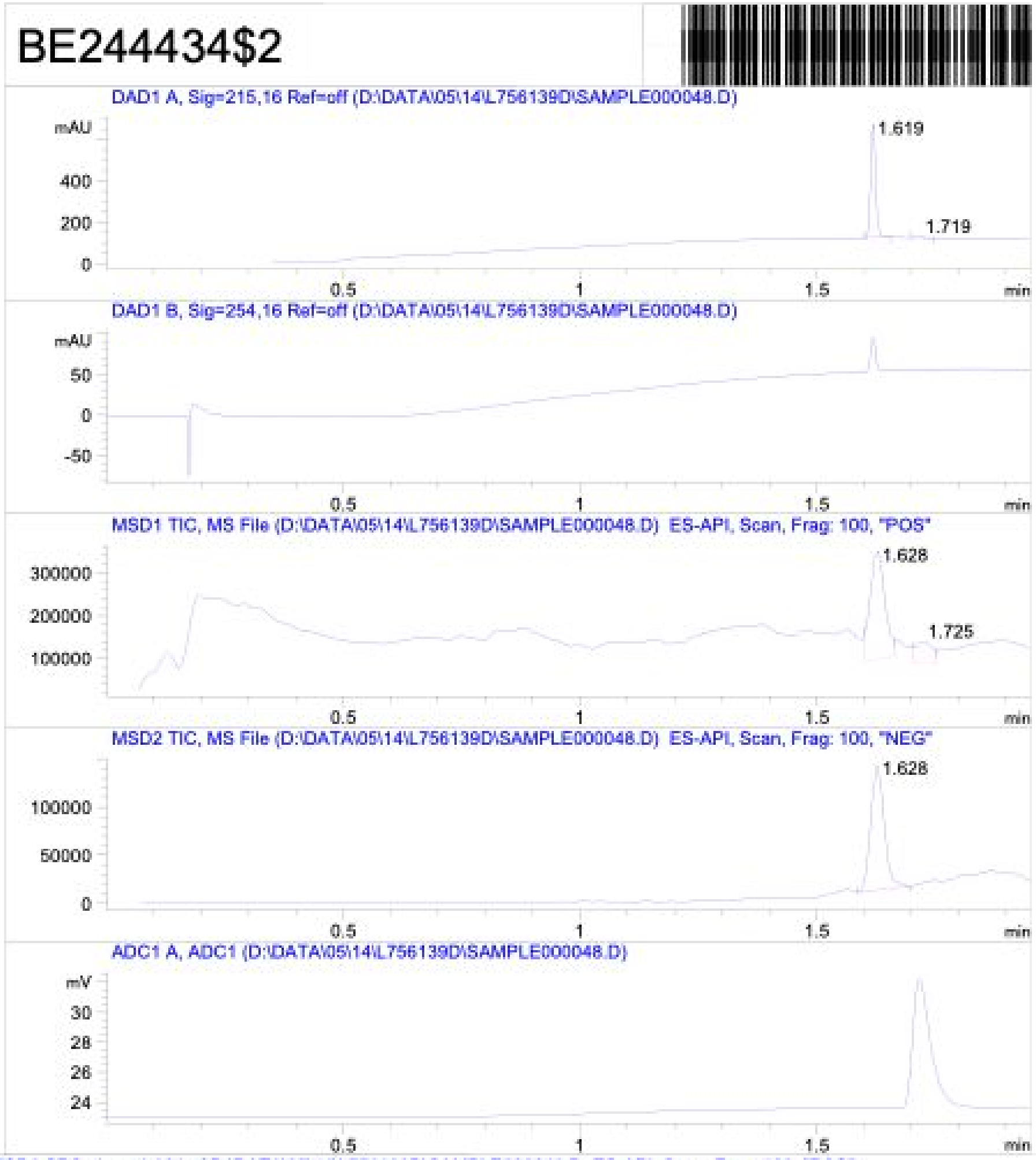

RT 1.628

RT 1.725

RT 1.628

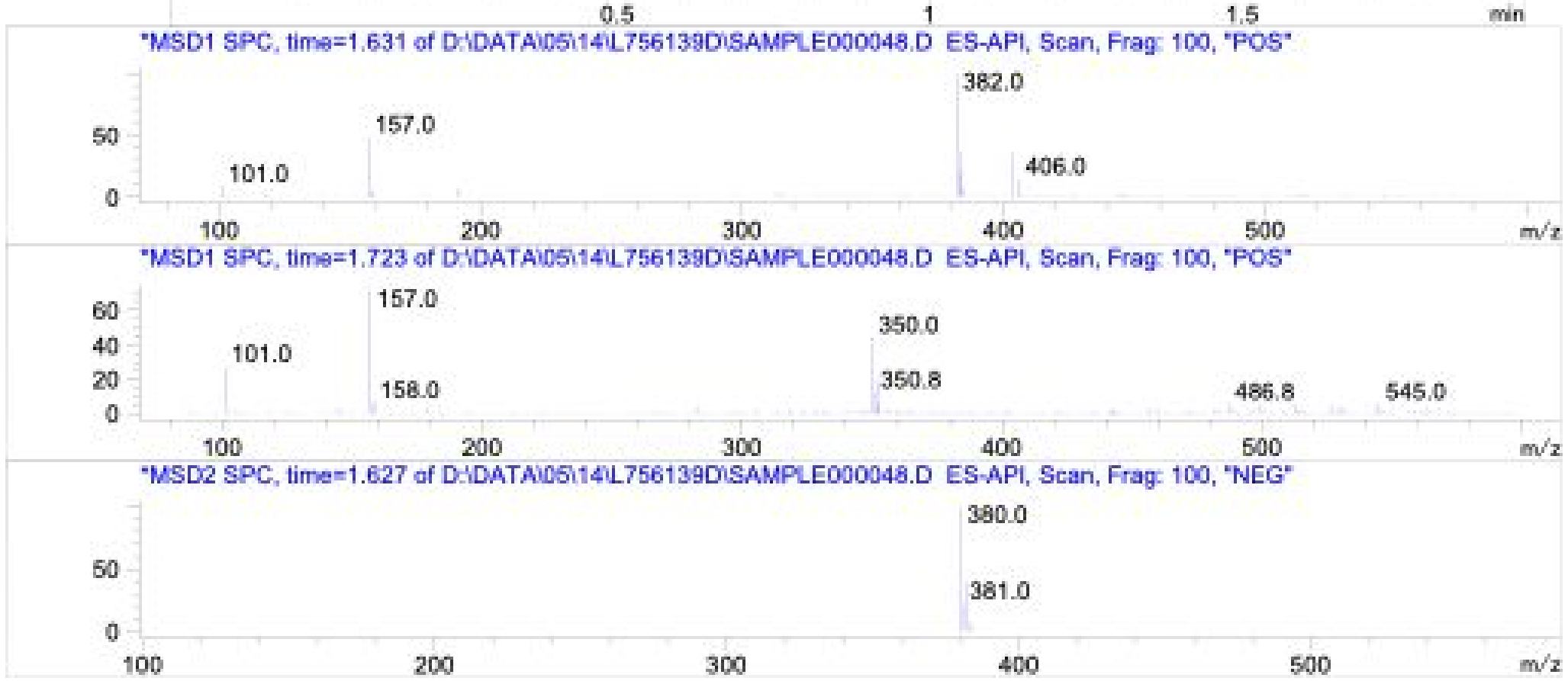

Inj.Date 5/13/2024 CH - 4 -
C:\CHEM32\1\METHODS\SUPOR_30.M

Z9103952180

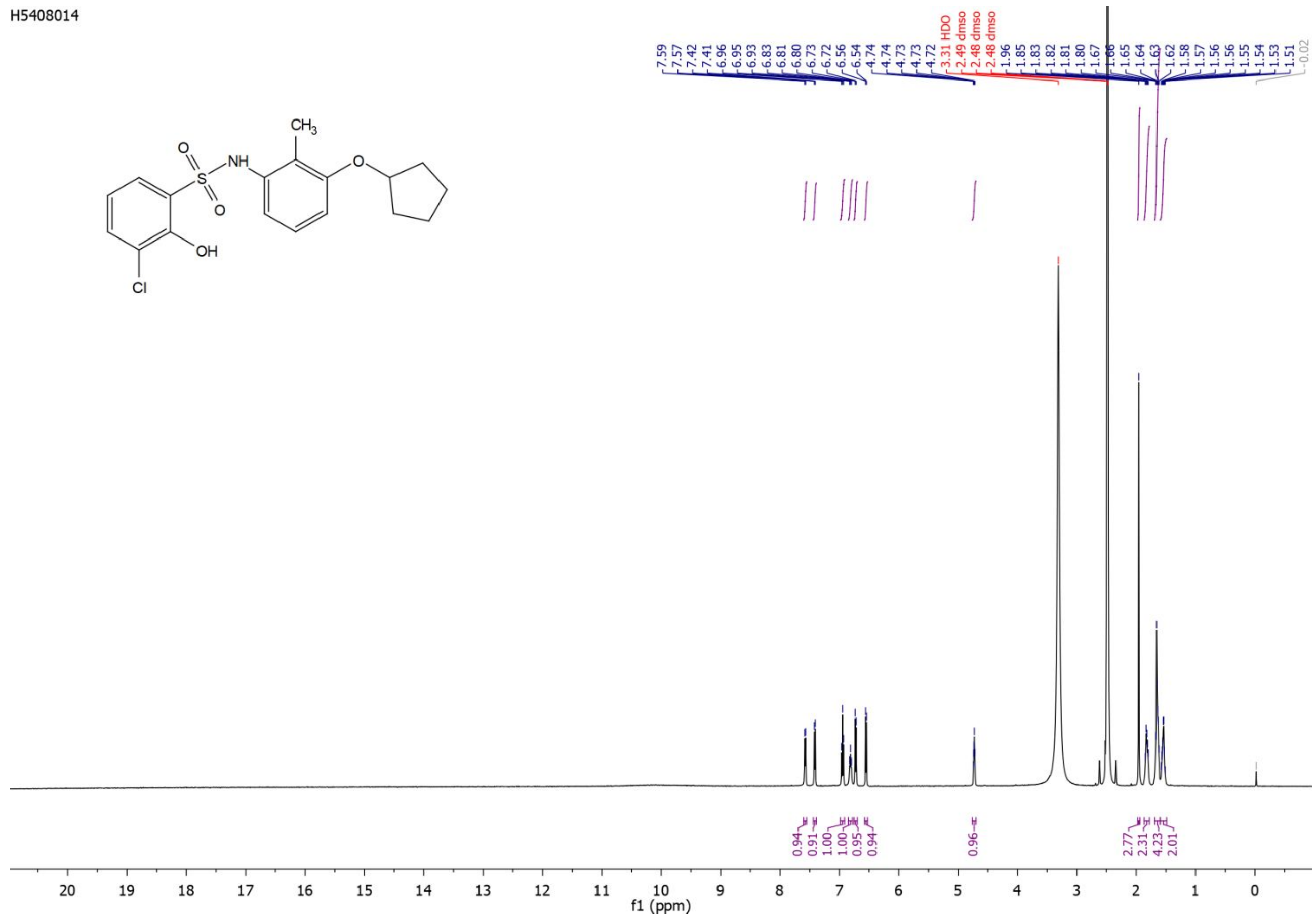

Z9103952180

MaxPeak: 98.24%
Ret_Time: 1.565 min

BE244448$4

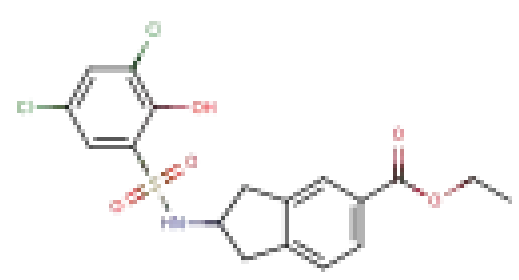

Mol Wt 430.3
Exact Mass 429.03

#	Time	Area%
1	1.054	1.76
2	1.565	98.24

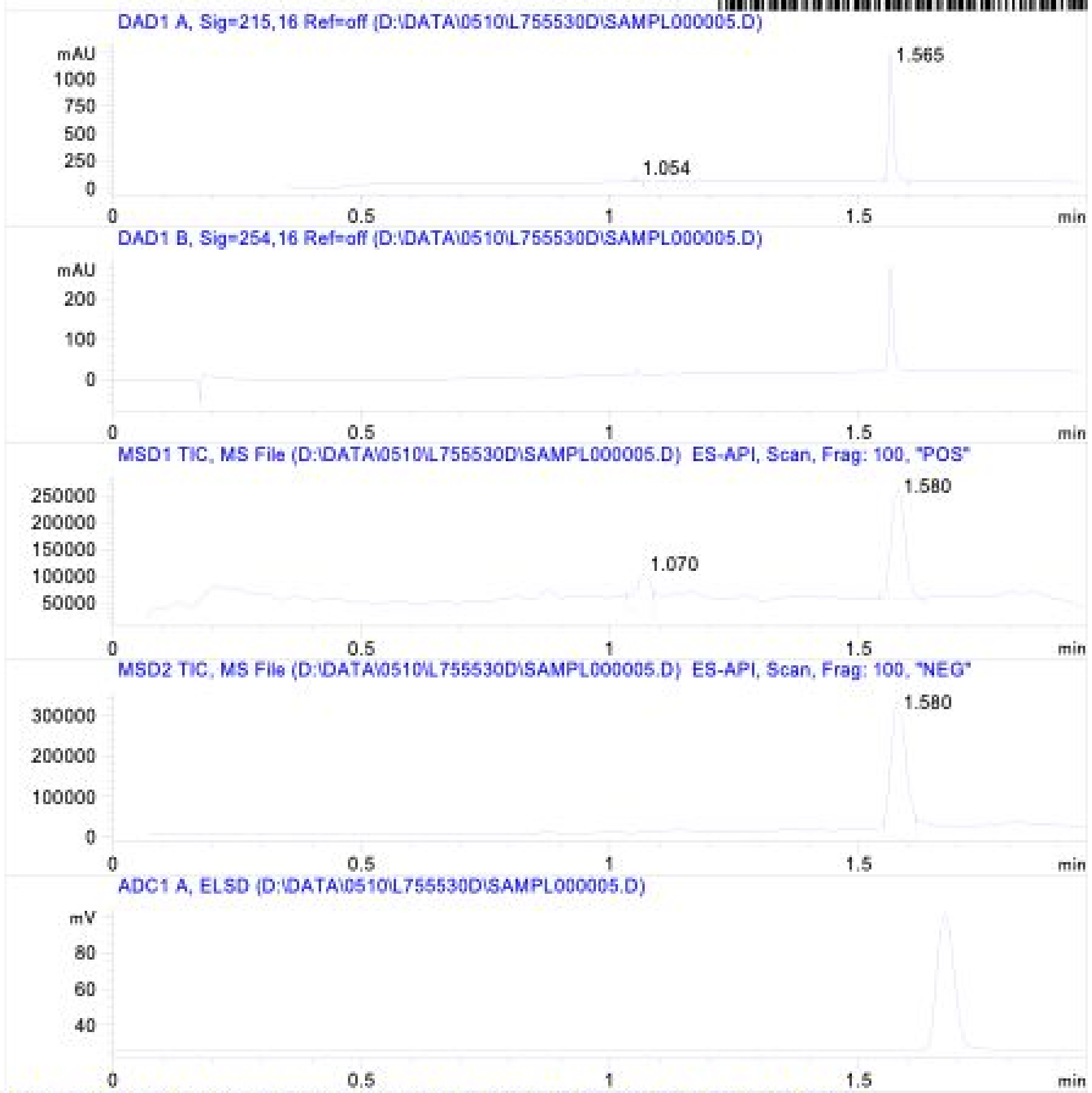

RT 1.070

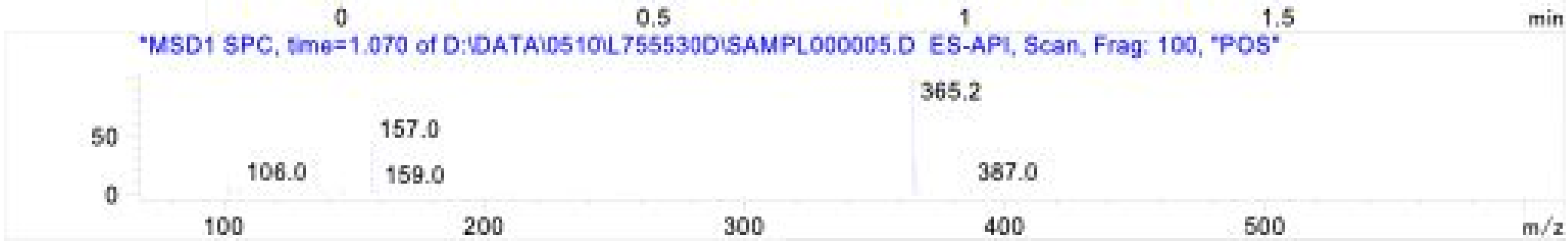

RT 1.580

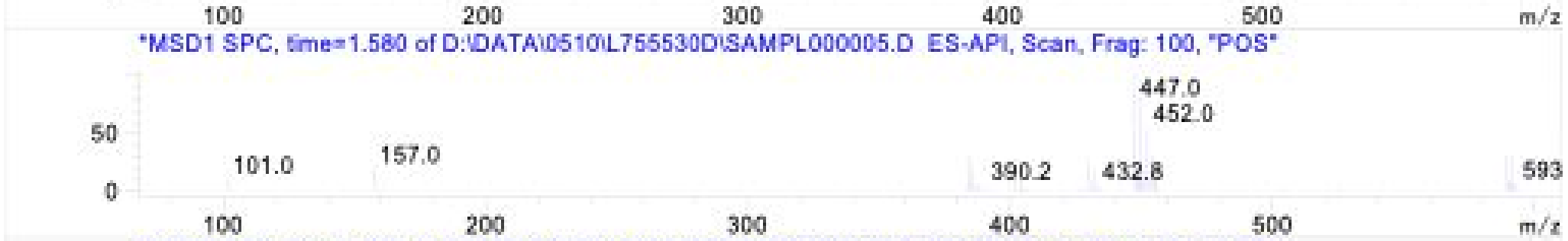

RT 1.580

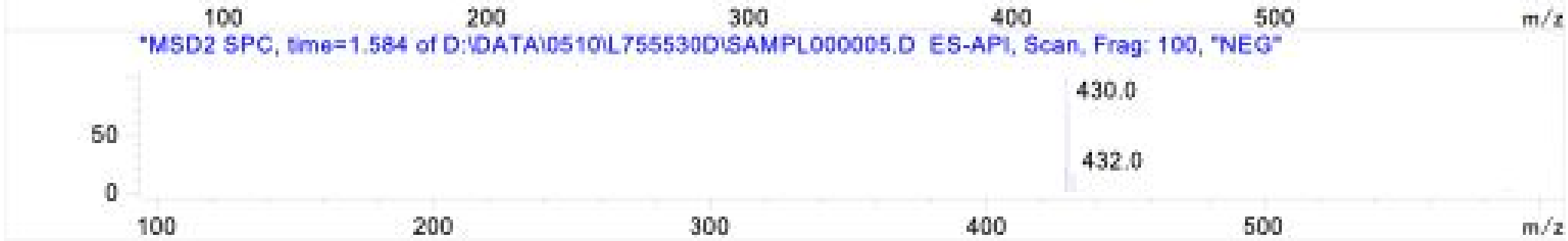

Inj.Date 5/10/2024 H -6-
C:\CHEM32\1\METHODS\SUPOR_30.M

Z9103952287

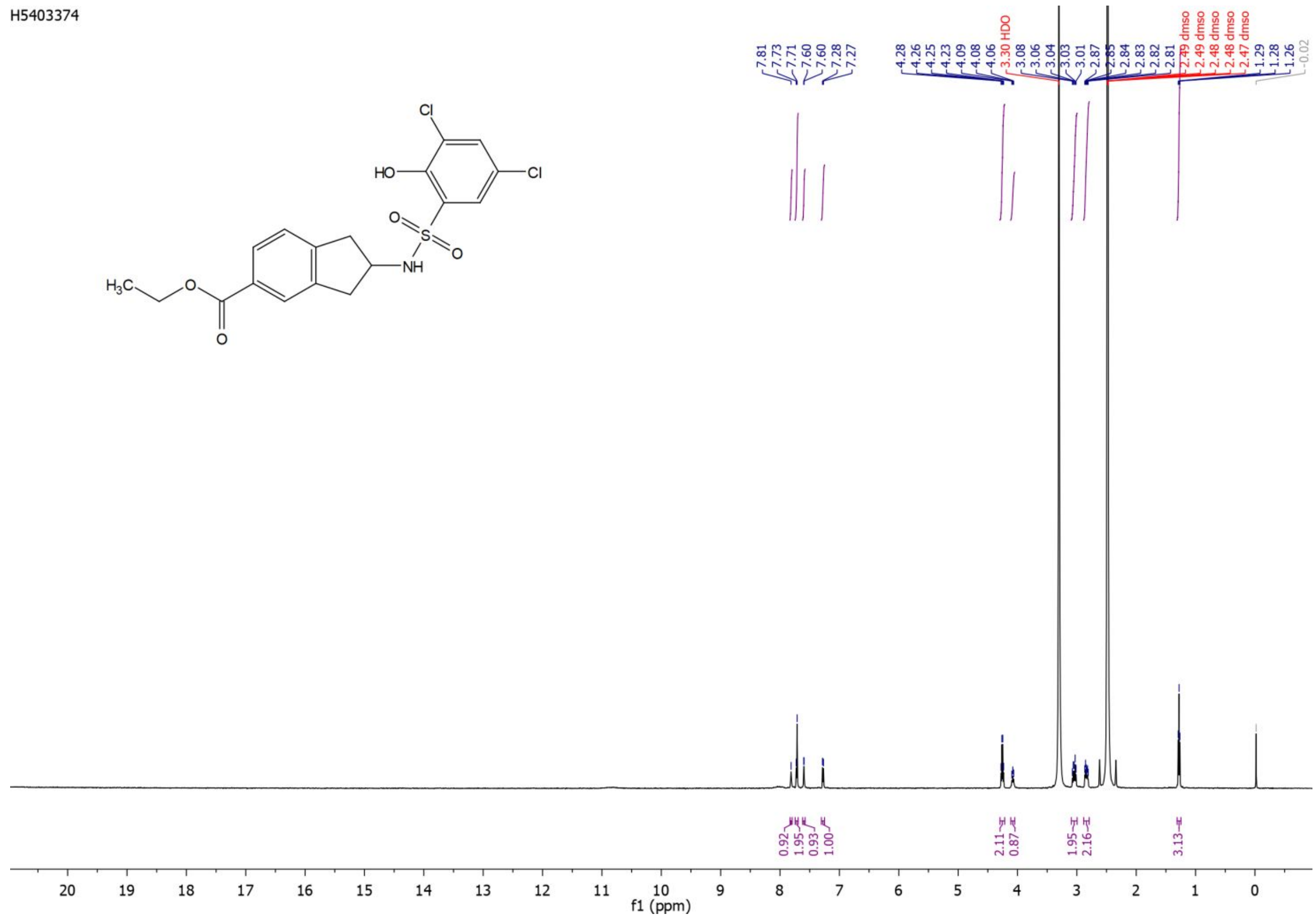

Z9103952287

MaxPeak: 96.65%
Ret_Time: 1.506 min

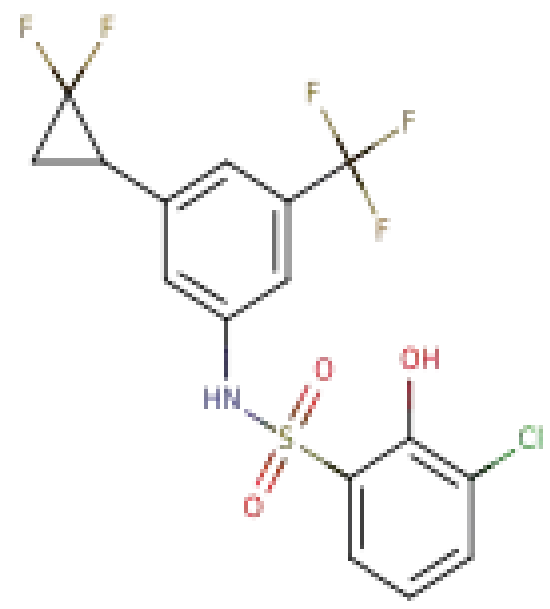

Mol Wt 427.77

Exact Mass 427.02

#	Time	Area%
1	1.506	96.65
2	1.792	3.35

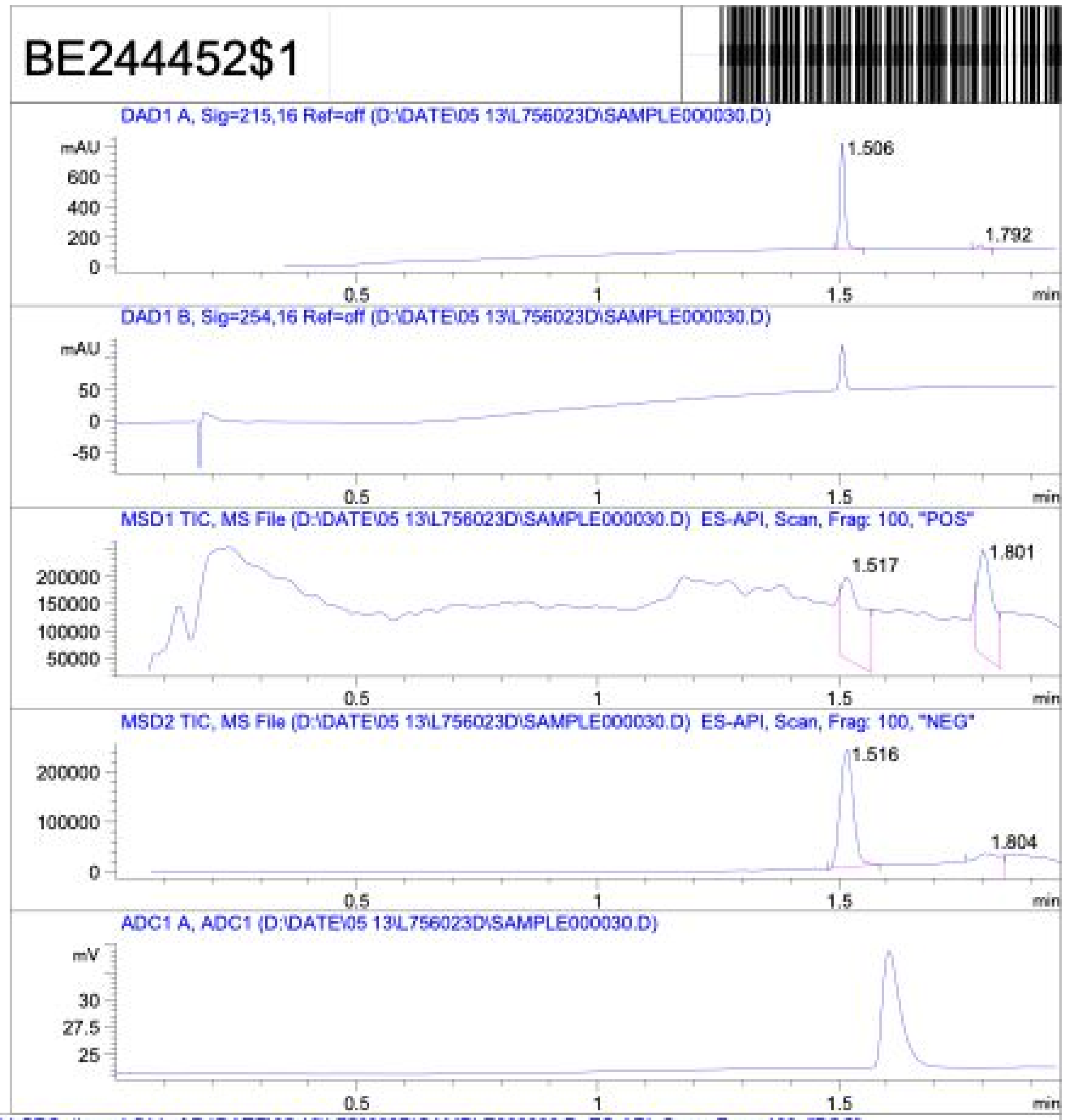

RT 1.517

RT 1.801

RT 1.516

RT 1.804

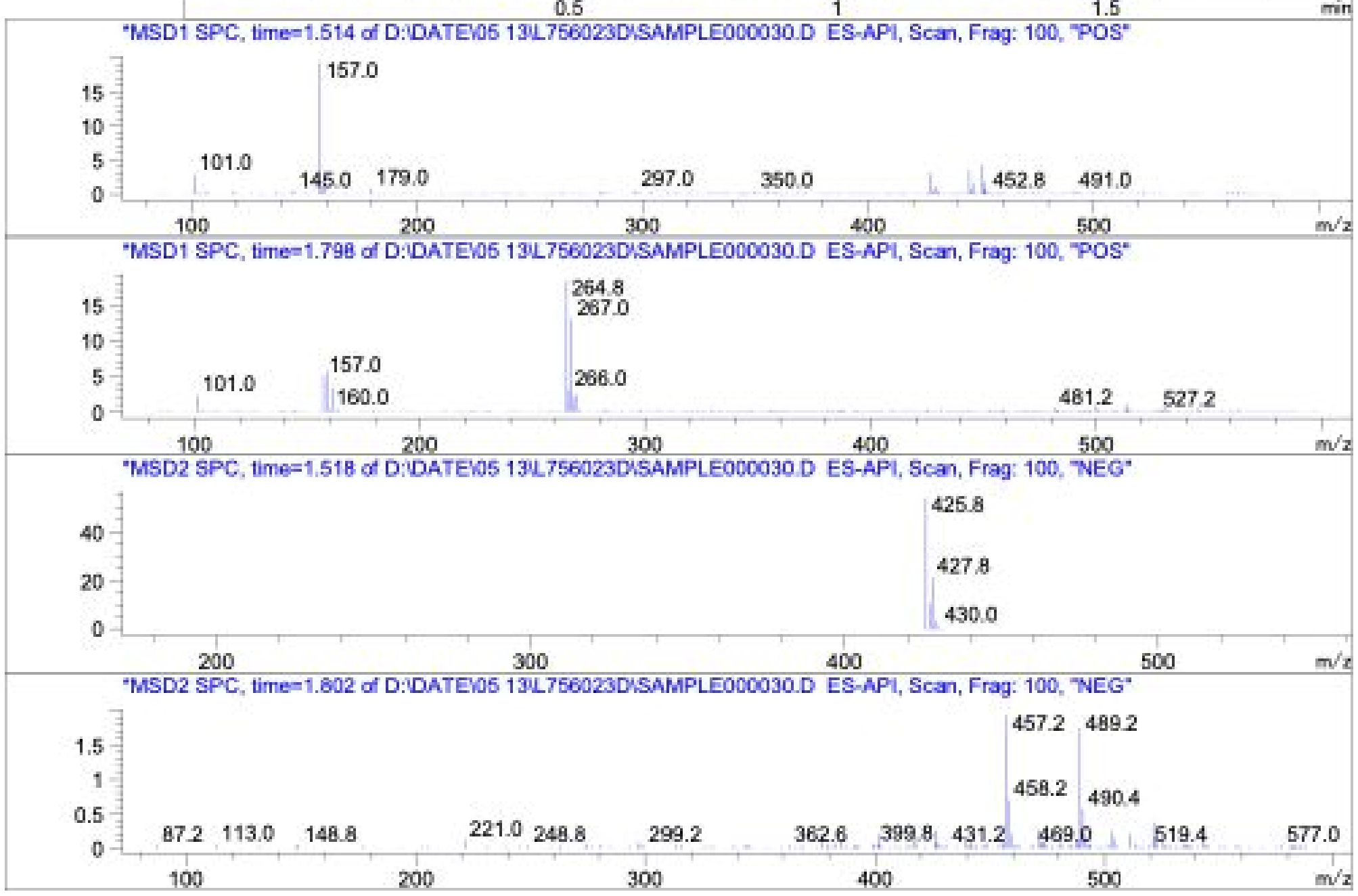

Inj.Date 5/13/2024 E - 4 -
C:\CHEM32\1\METHODS\SUPOR_30.M

Z9103952313

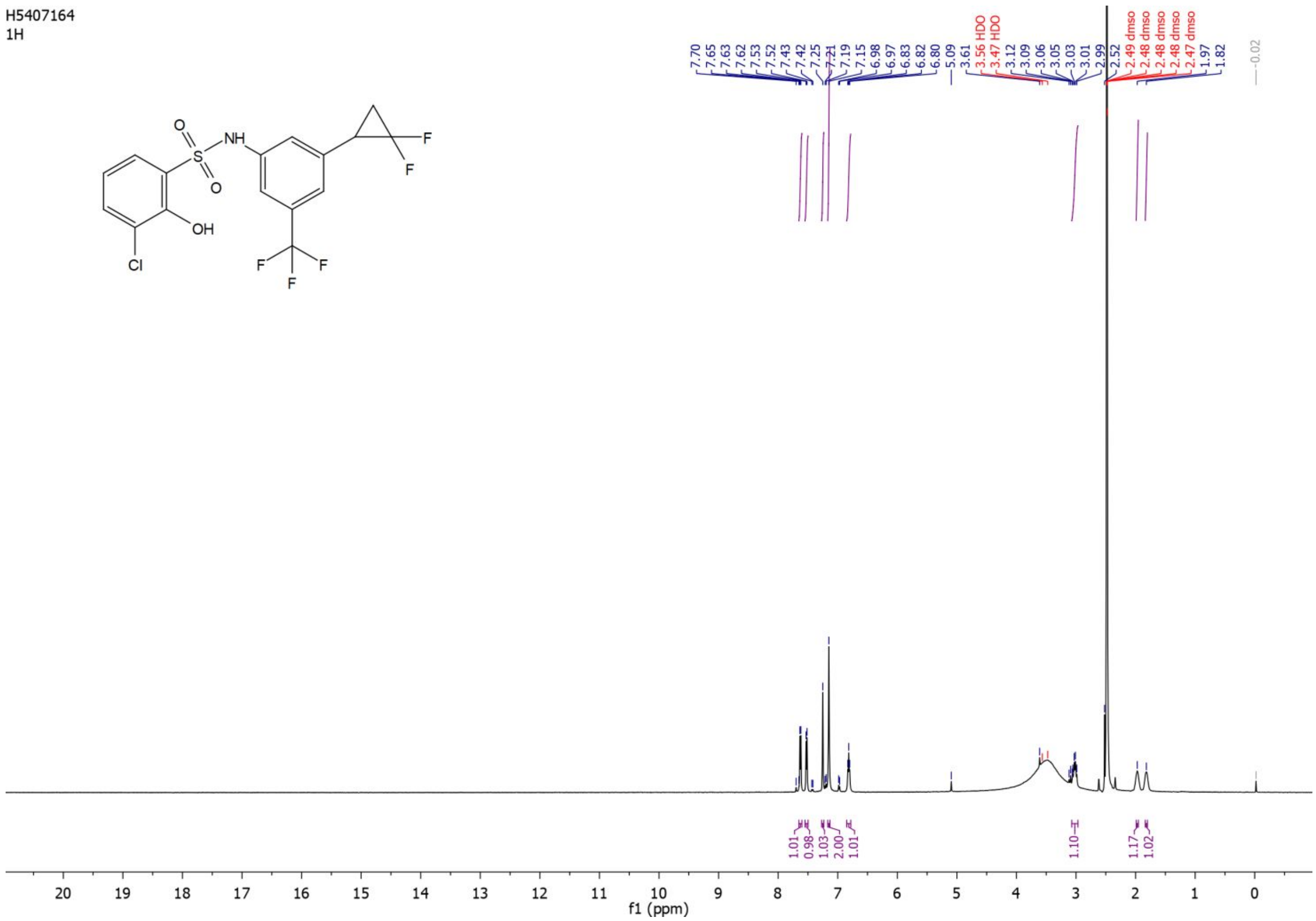

Z9103952313

MaxPeak: 100.00%
Ret_Time: 1.178 min

BF054173$1

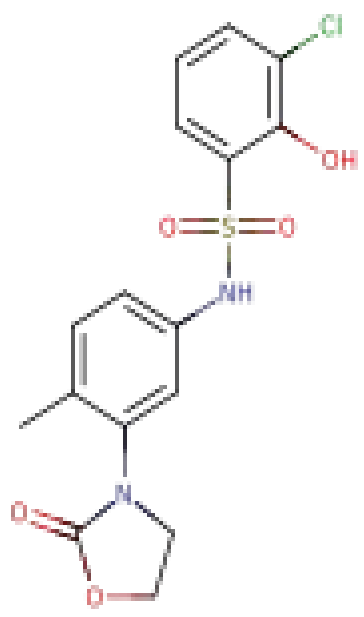

Mol Wt 382.82

Exact Mass 382.04

#	Time	Area%
1	1.178	100.00

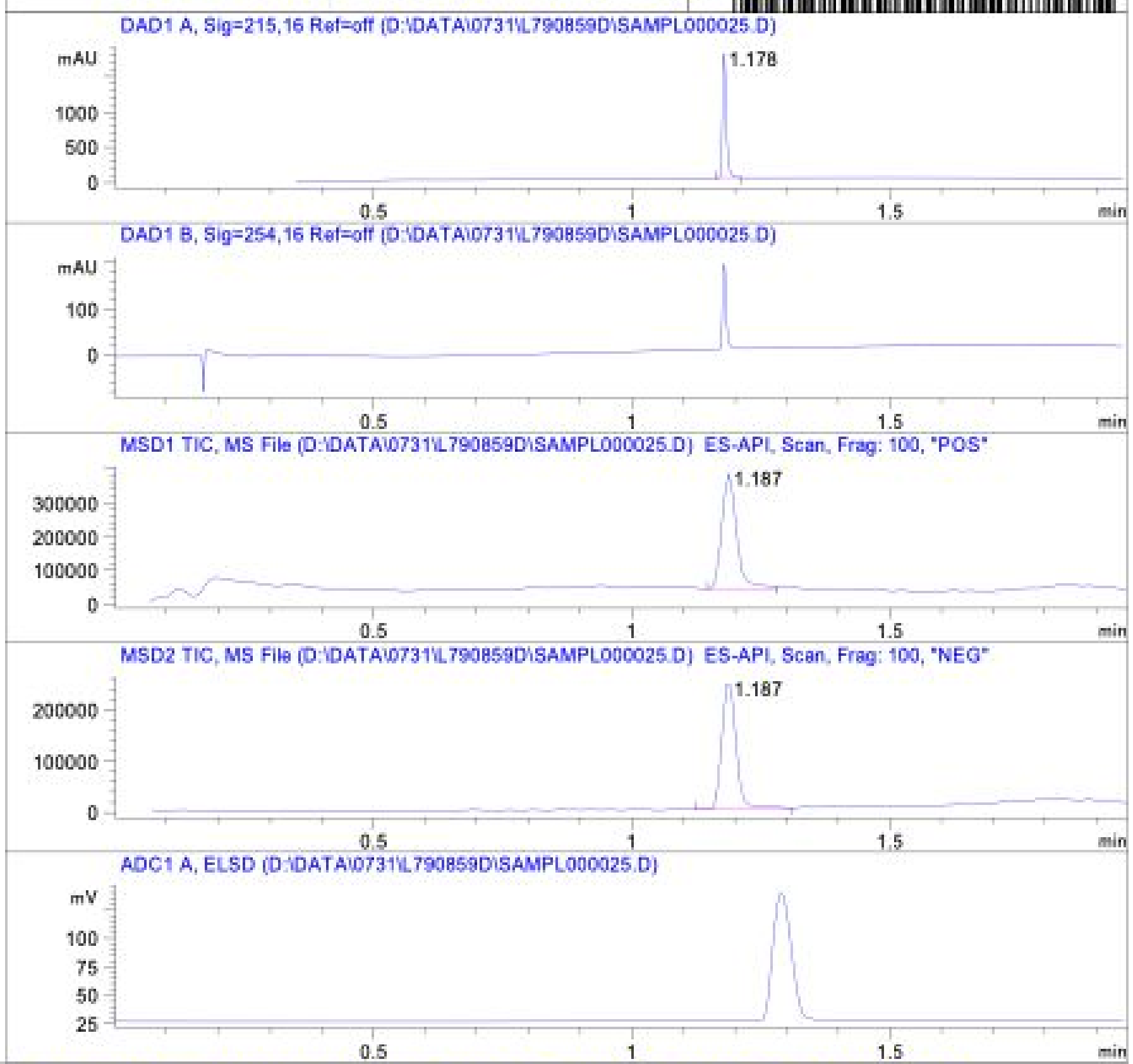

RT 1.187

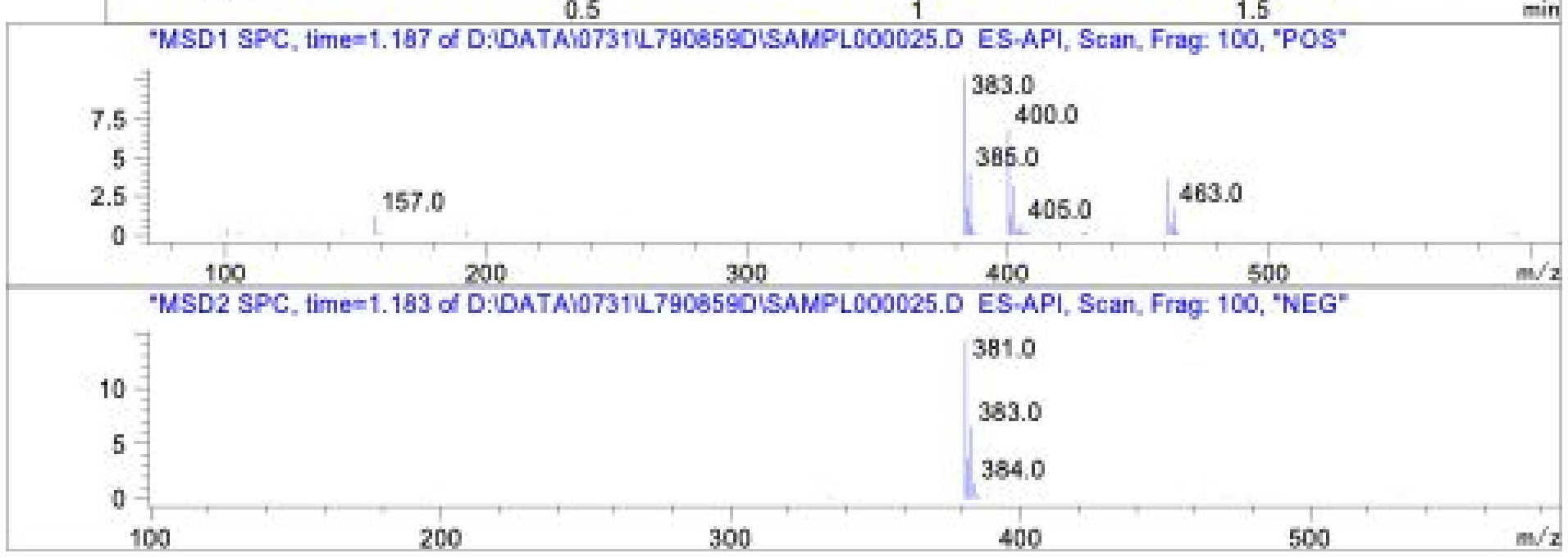

RT 1.187

Inj.Date 7/31/2024 LB -6-
C:\CHEM32\1\METHODS\SUPOR_30.M

Z9212924971

H5599482
1H

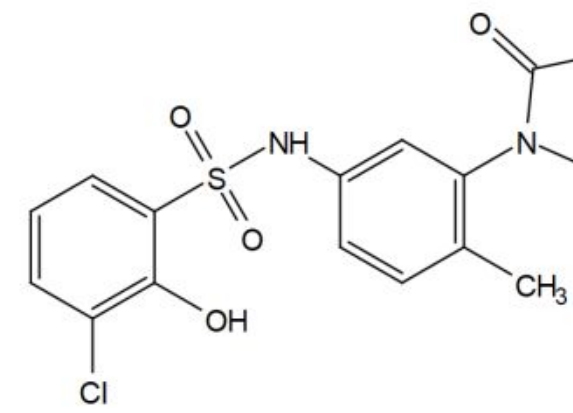

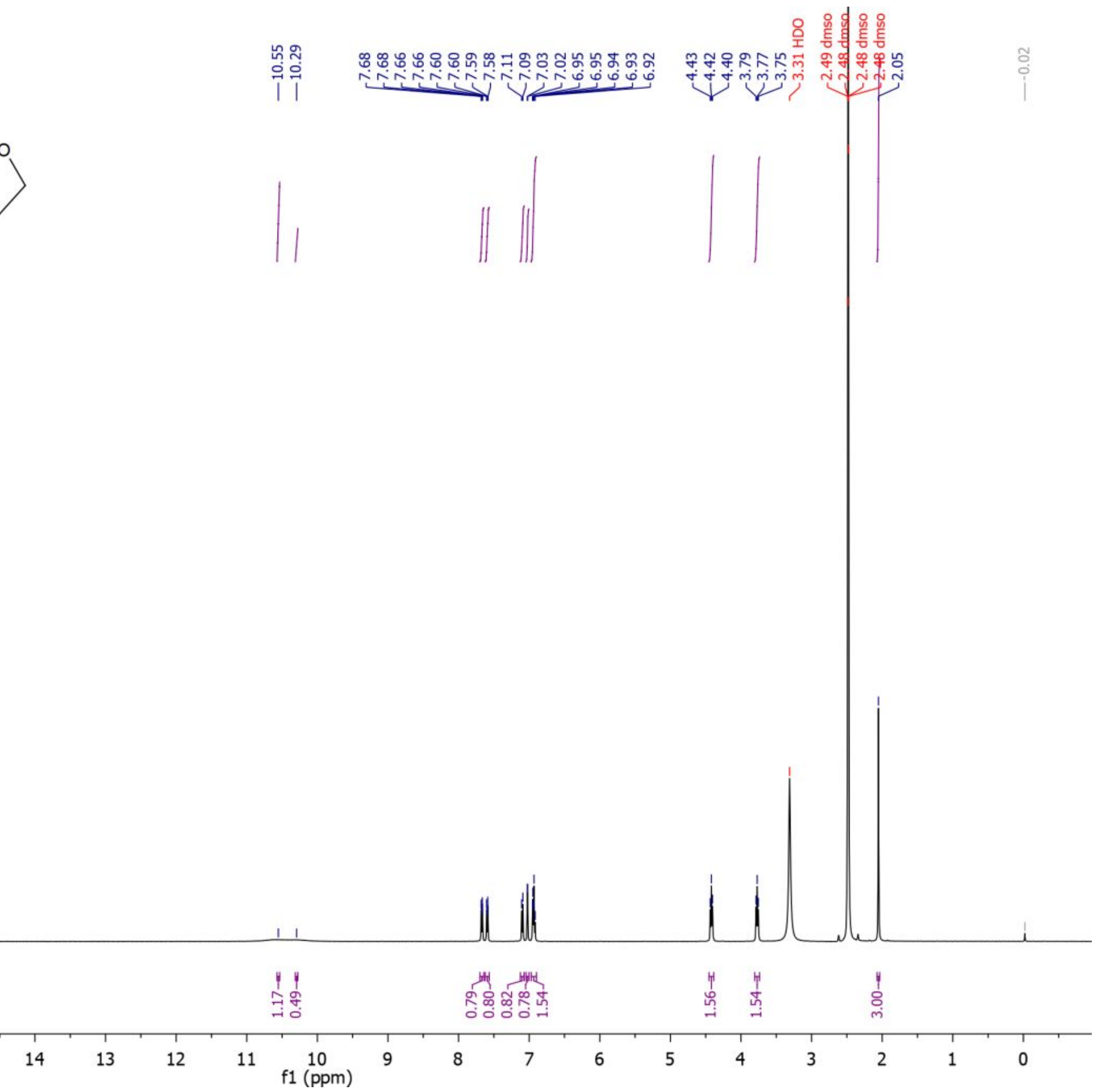

Z9212924971

MaxPeak: 100.00%
Ret_Time: 1.275 min

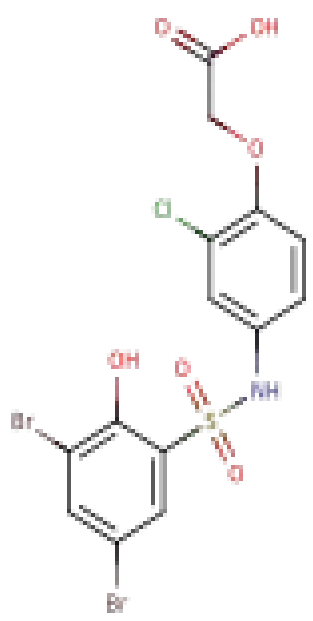

Mol Wt 515.56

Exact Mass 514.82

#	Time	Area%
1	1.275	100.00

RT 1.286

RT 1.284

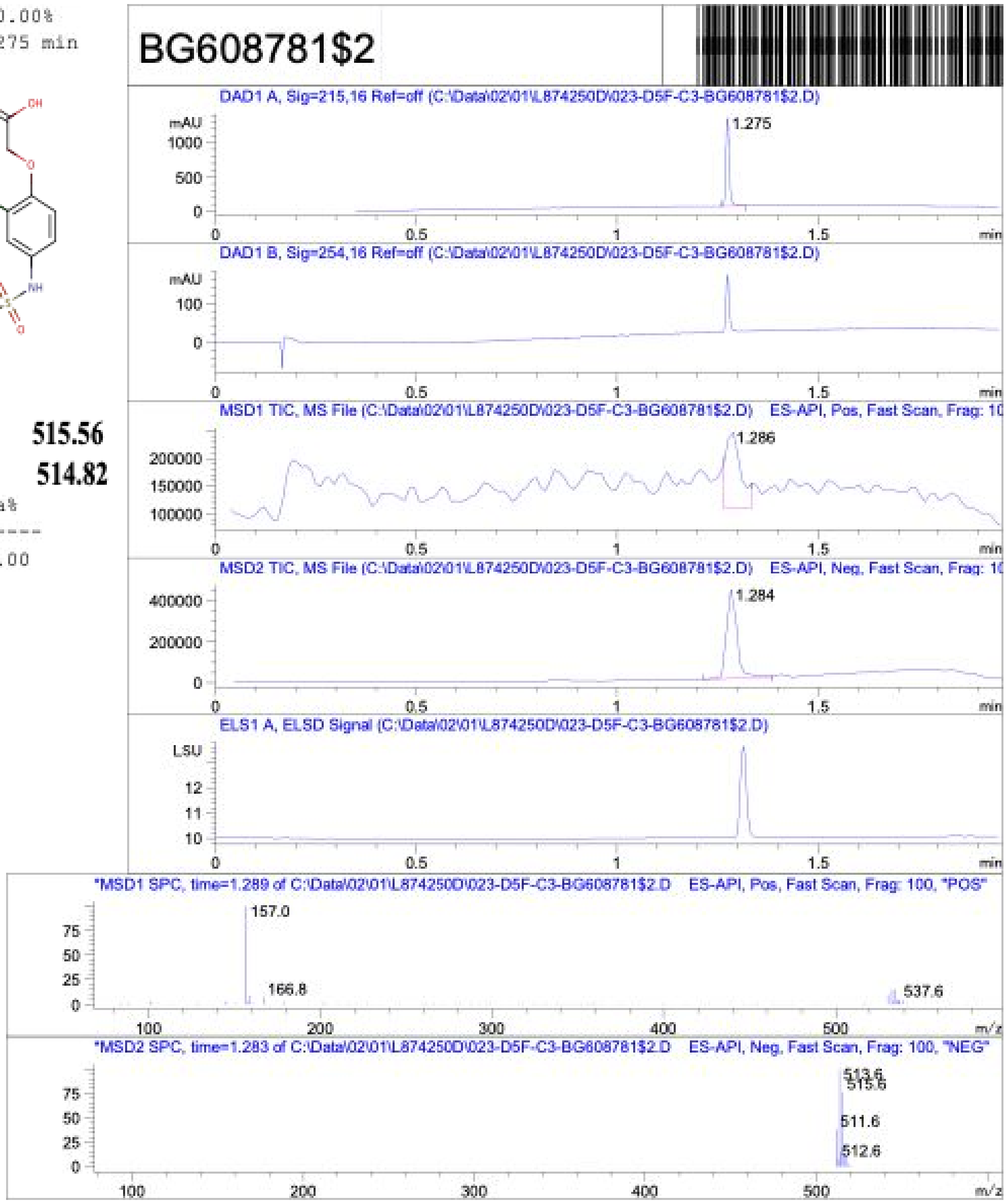

Inj.Date 1/31/2025 C
C:\Users\Public\Documents\ChemStation\1\Data\01_31\L874250D\SUPOR_30.M

Z9464814846

H6034287
1H

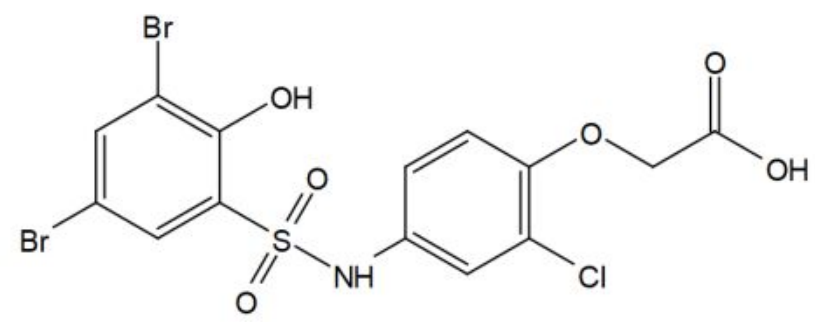

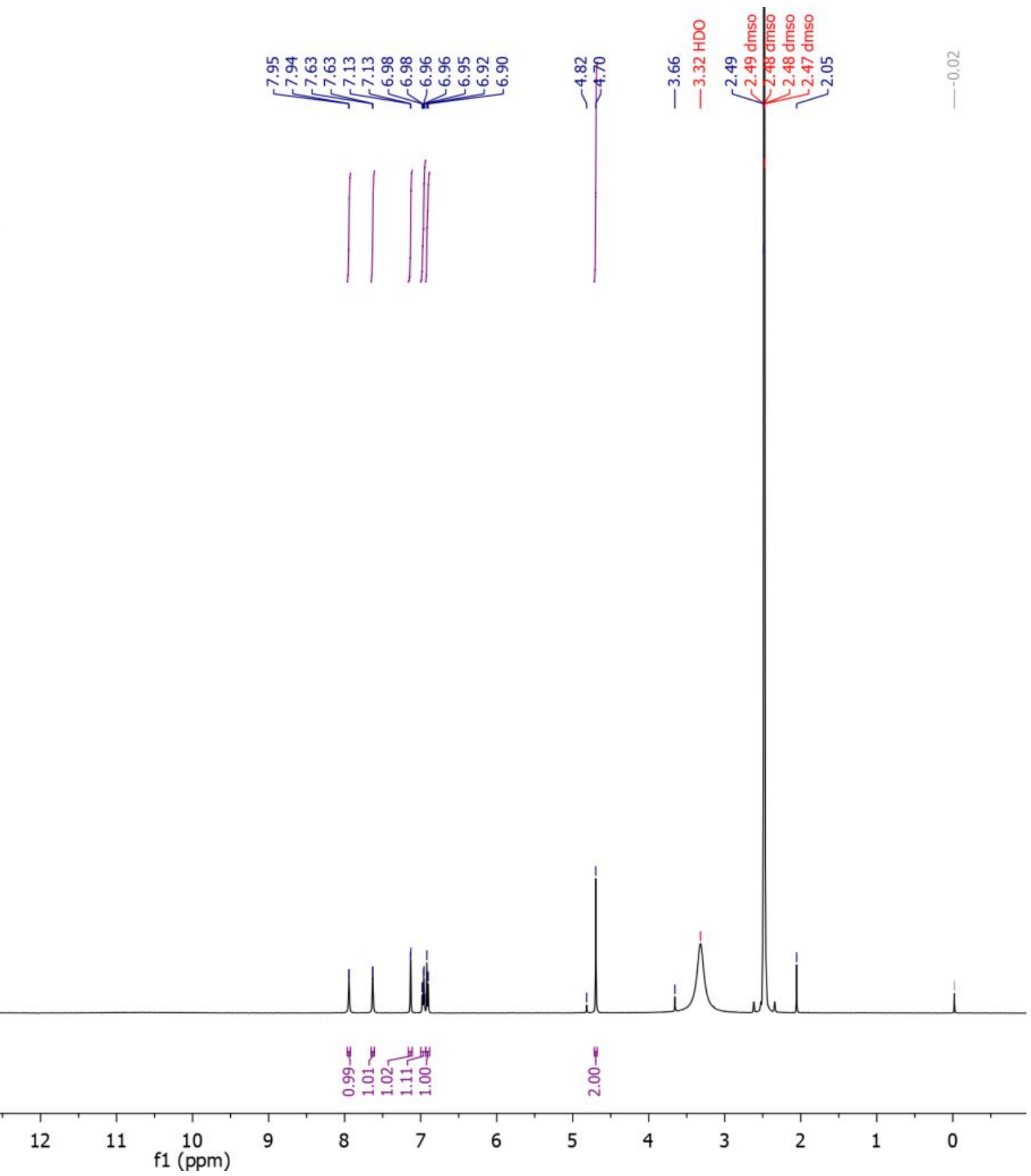

Z9464814846

MaxPeak: 100.00%
Ret_Time: 1.370 min

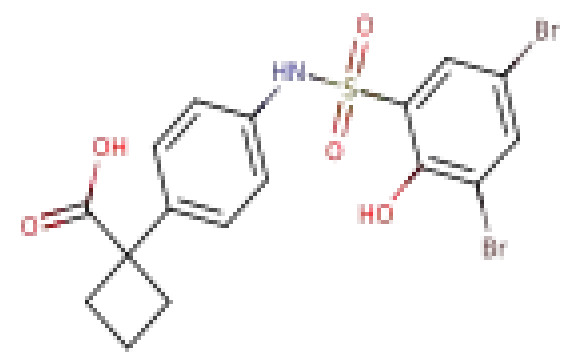

Mol Wt 505.18

Exact Mass 504.91

#	Time	Area%
1	1.370	100.00

BG608782$1

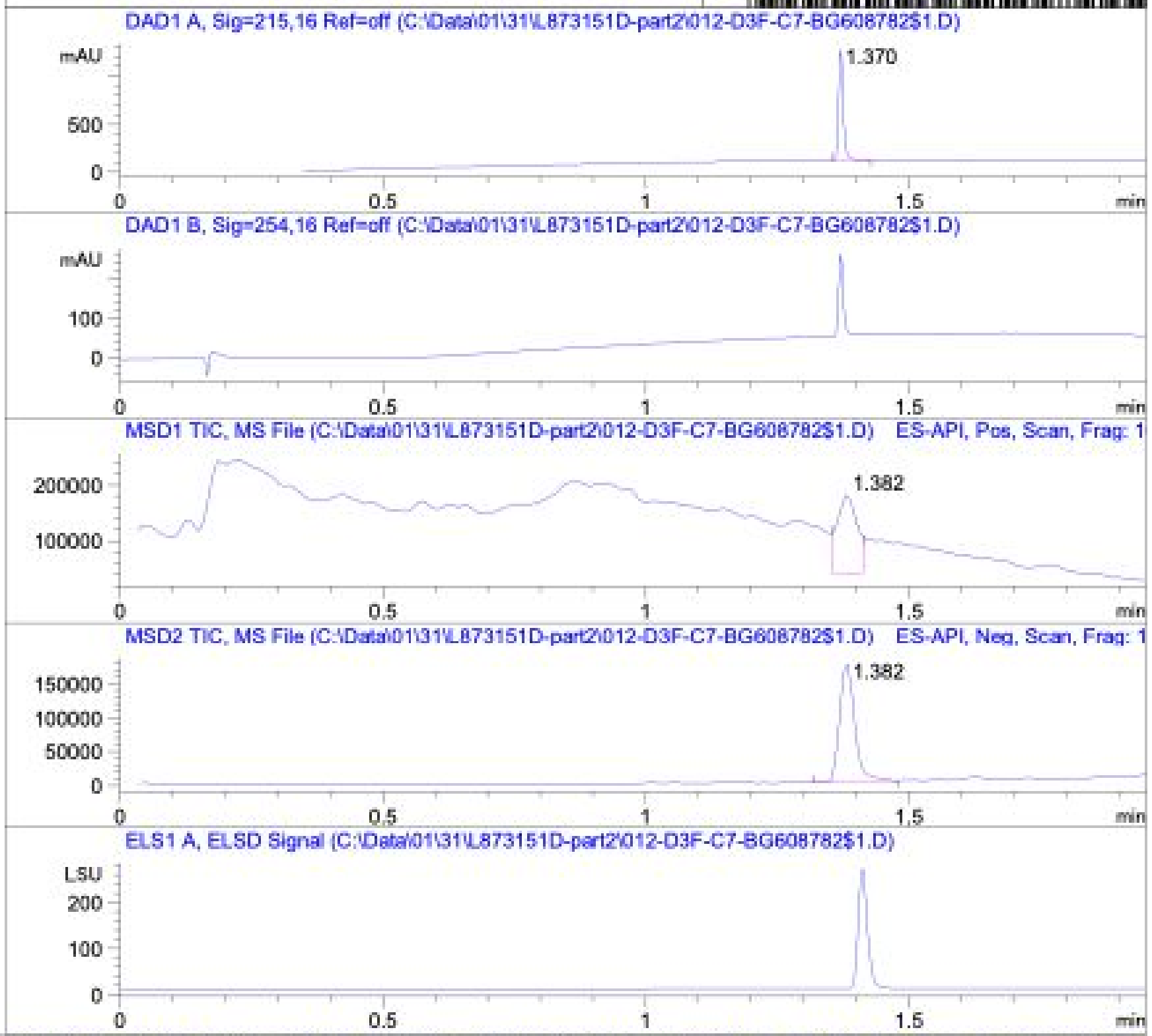

RT 1.382

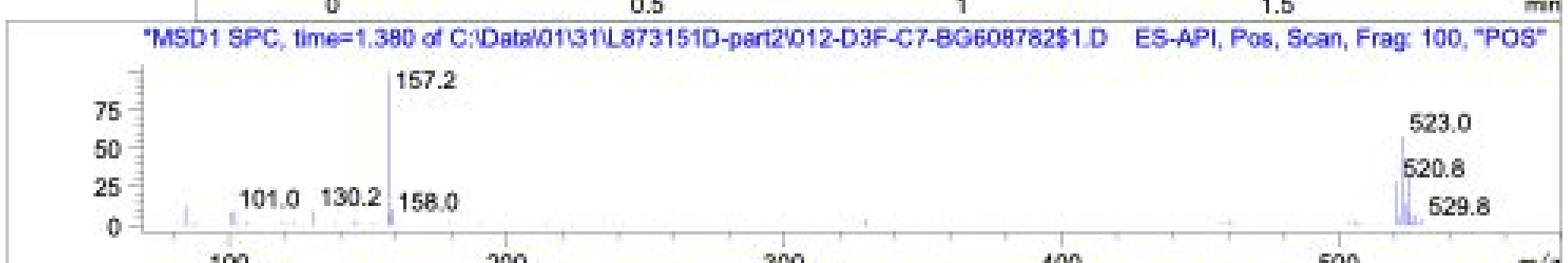

RT 1.382

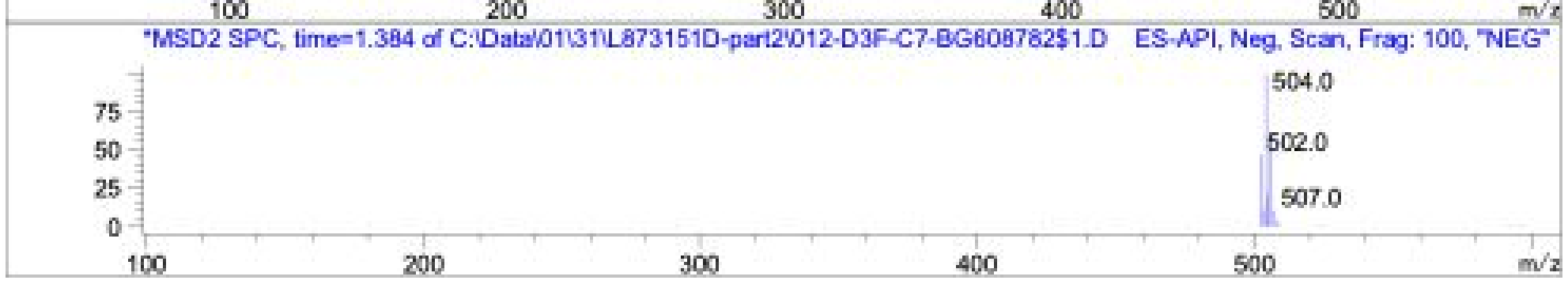

Inj.Date 1/30/2025 C 13
C:\Users\Public\Documents\ChemStation\1\Data\01_30\2873151D\SUPOR_30.M

Z9464814853

H6028463

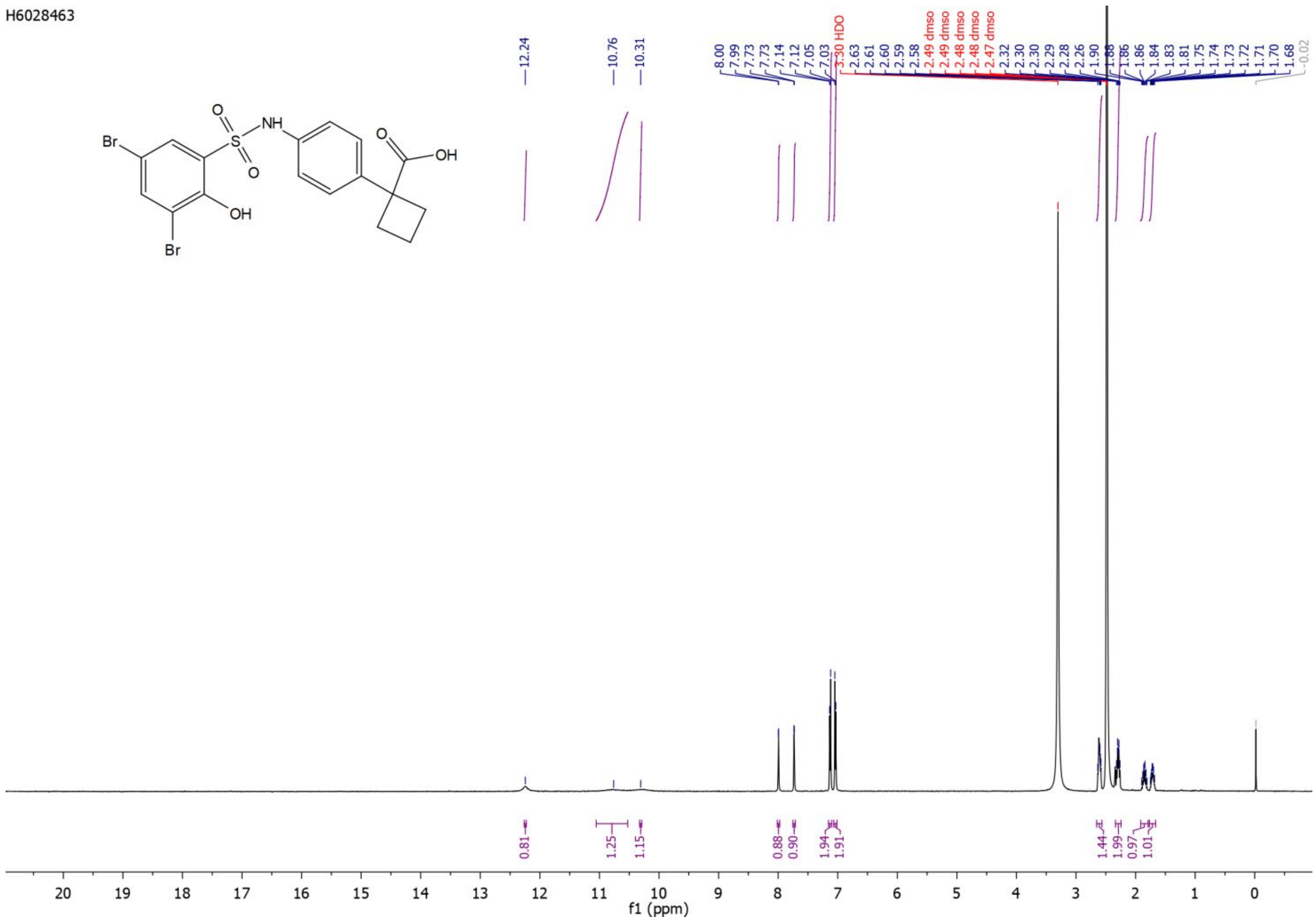

Z9464814853

MaxPeak: 96.78%
Ret_Time: 1.417 min

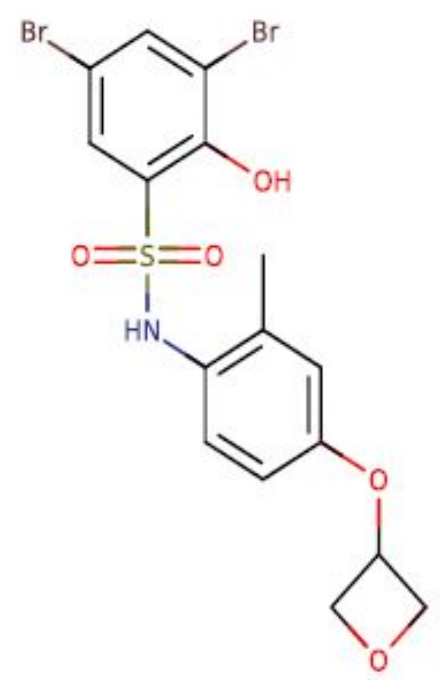

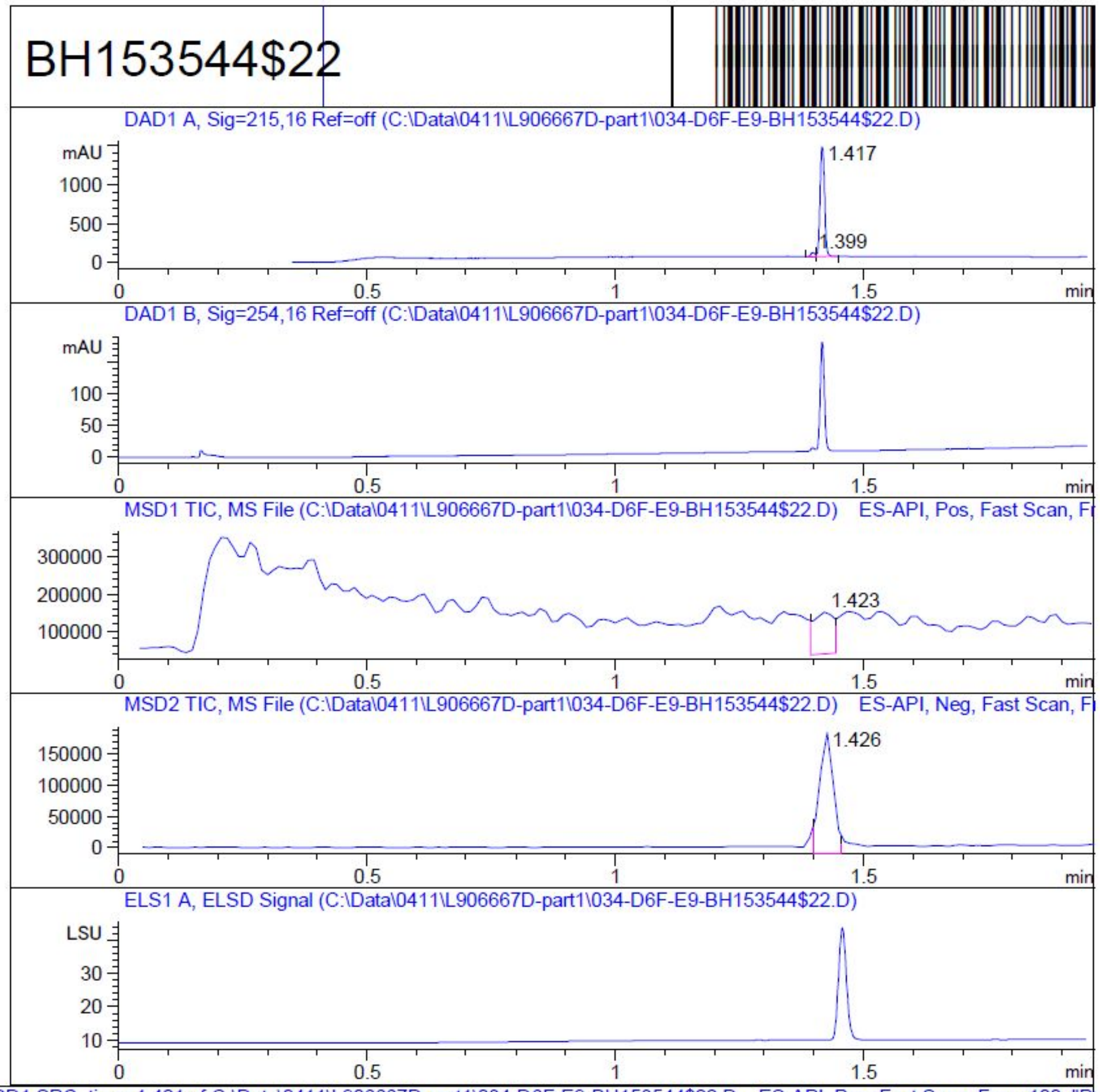

Mol Wt 493.17

Exact Mass 492.91

#	Time	Area%
1	1.399	3.22
2	1.417	96.78

RT 1.423

RT 1.426

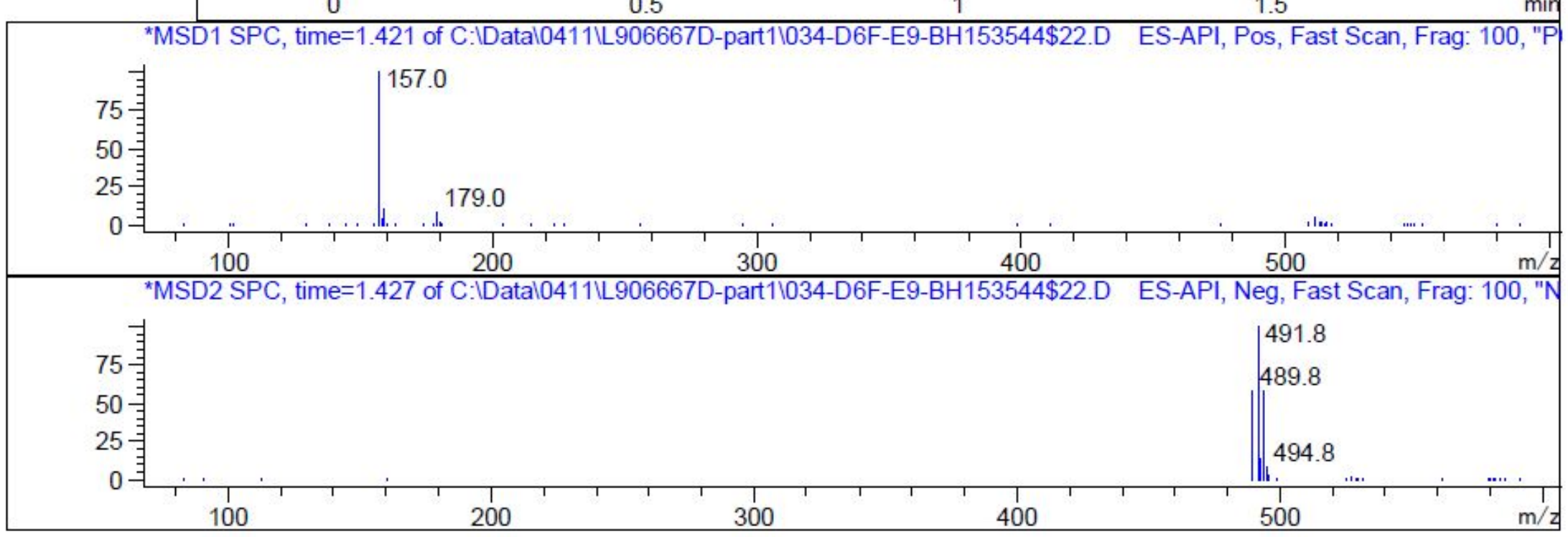

Z9544921833

H6177449

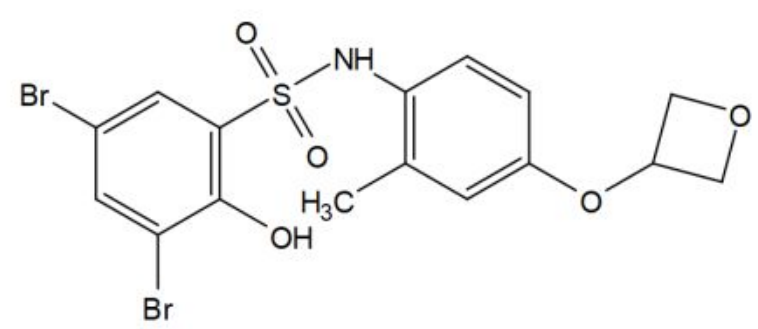

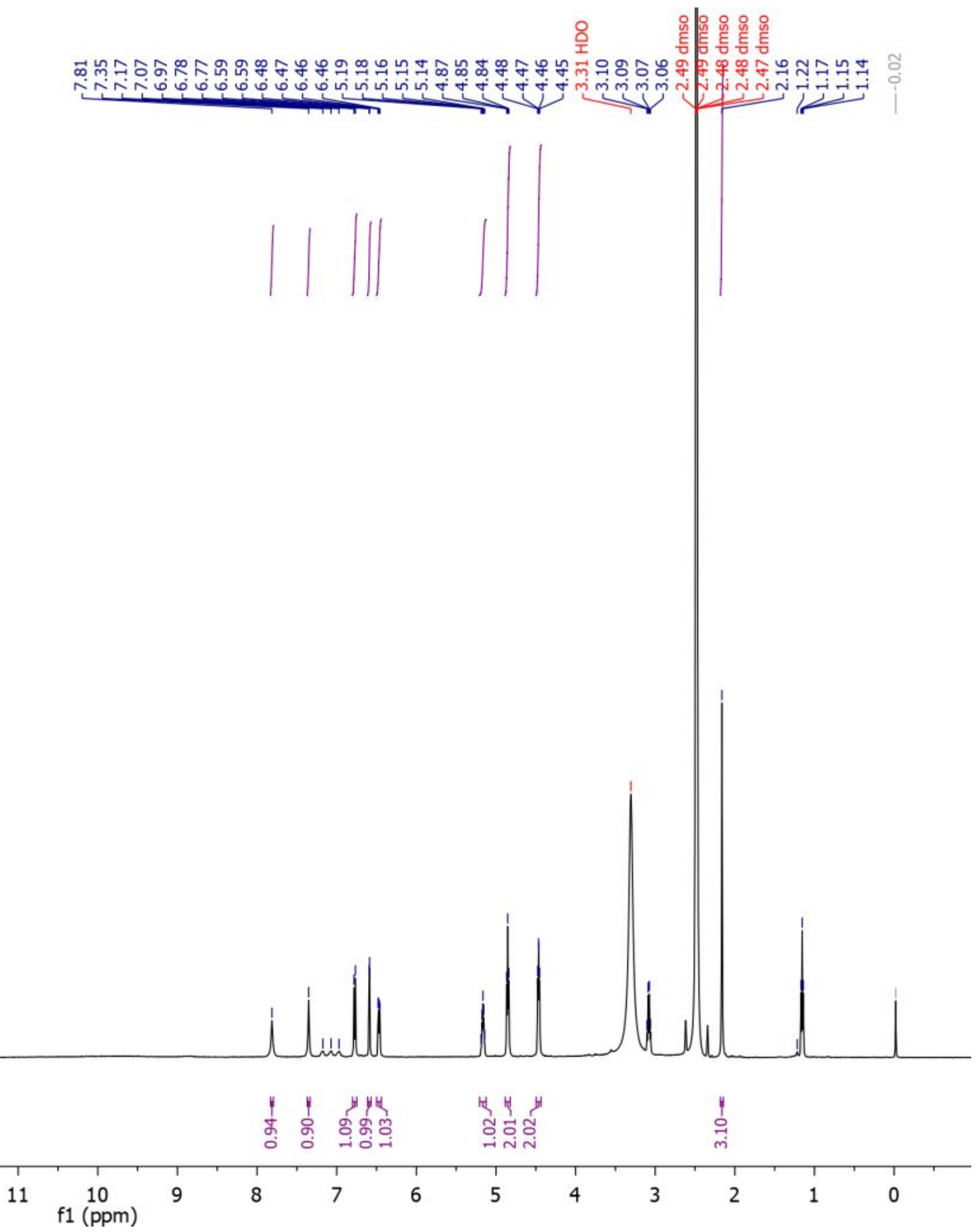

Z9544921833

MaxPeak: 95.39%
Ret_Time: 1.292 min

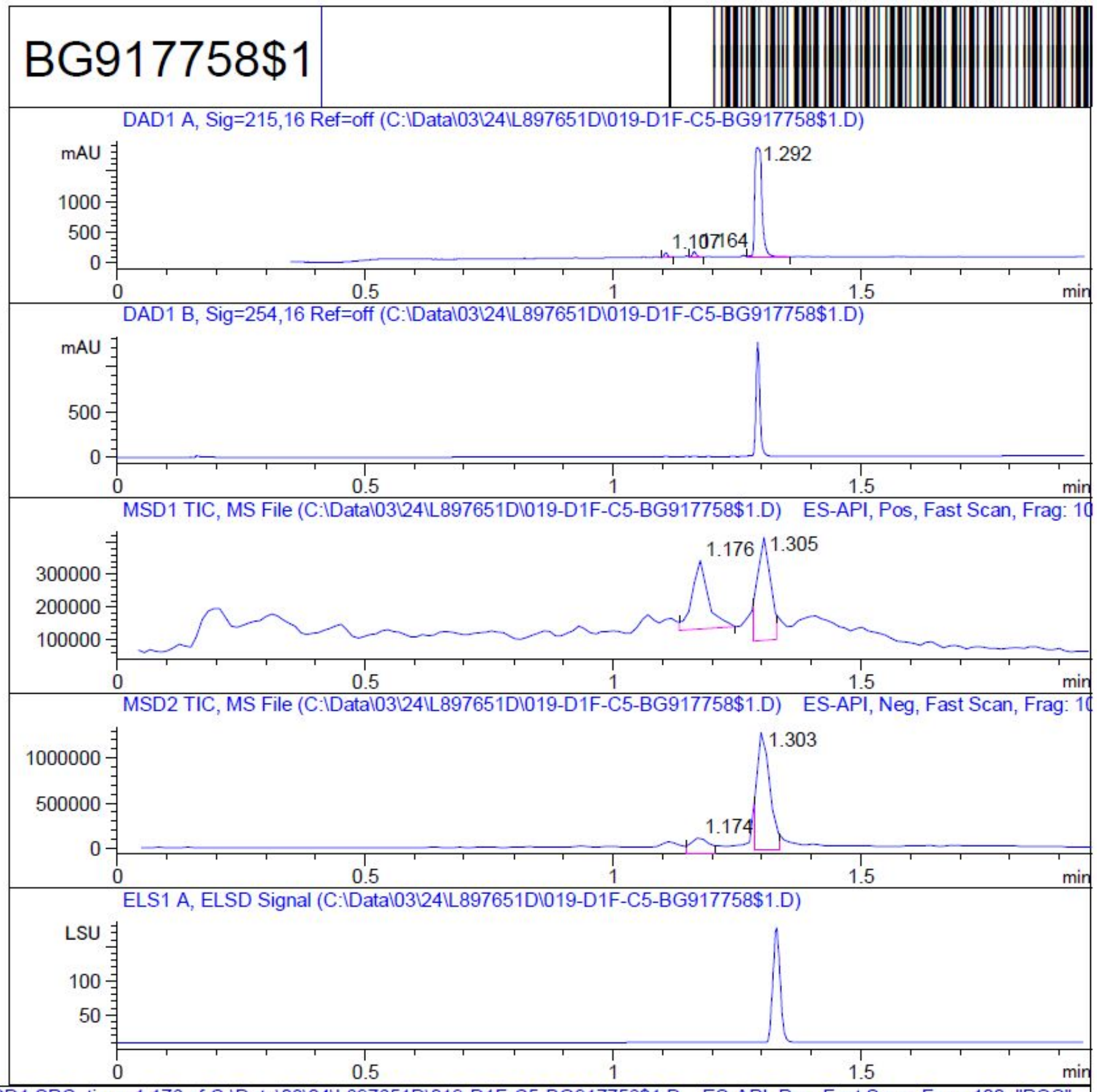

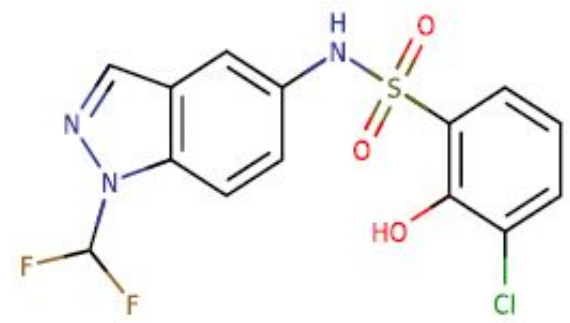

Mol Wt 373.76
Exact Mass 373.01

#	Time	Area%
1	1.107	1.89
2	1.164	2.71
3	1.292	95.39

RT 1.176

RT 1.305

RT 1.174

RT 1.303

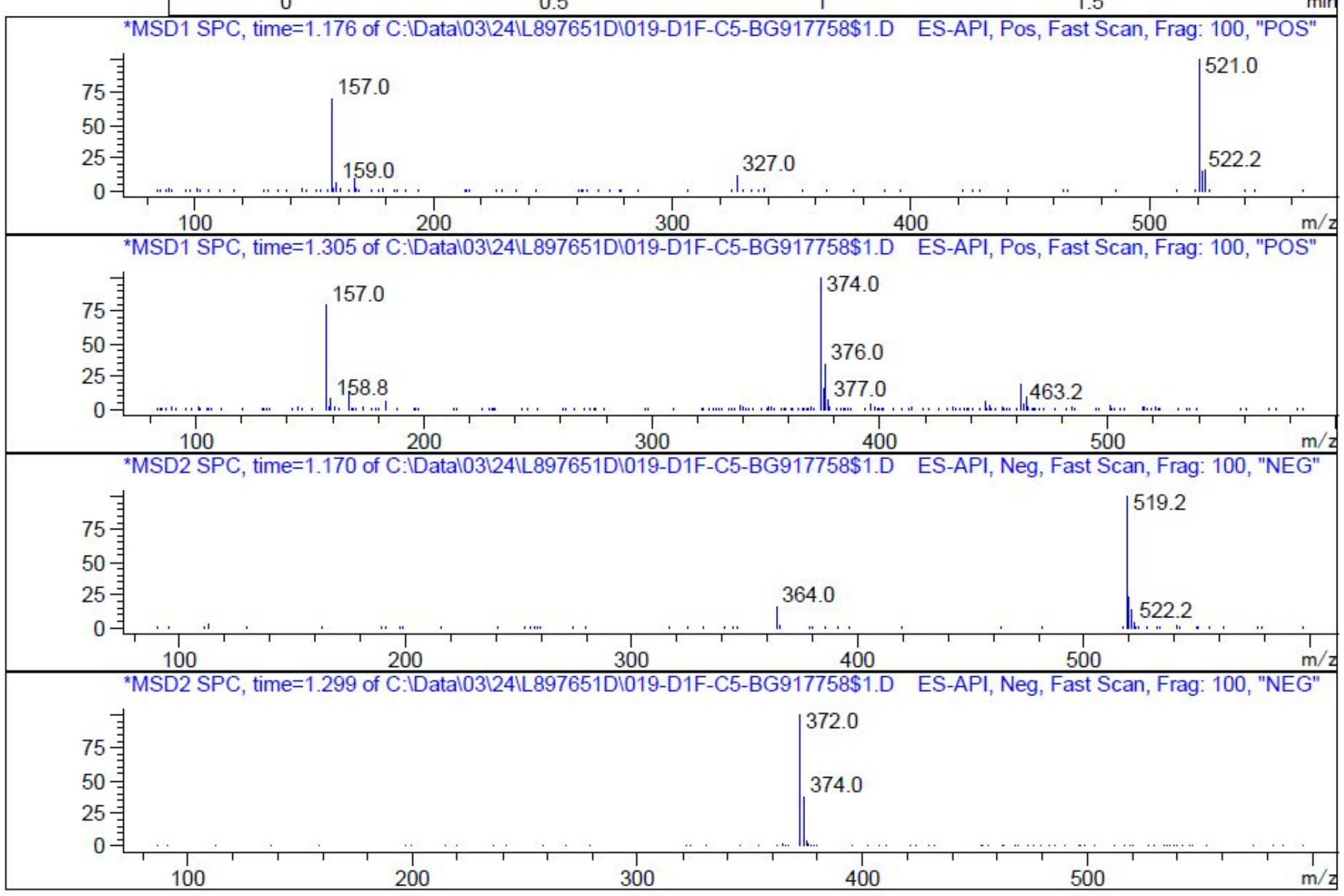

Z9544921868

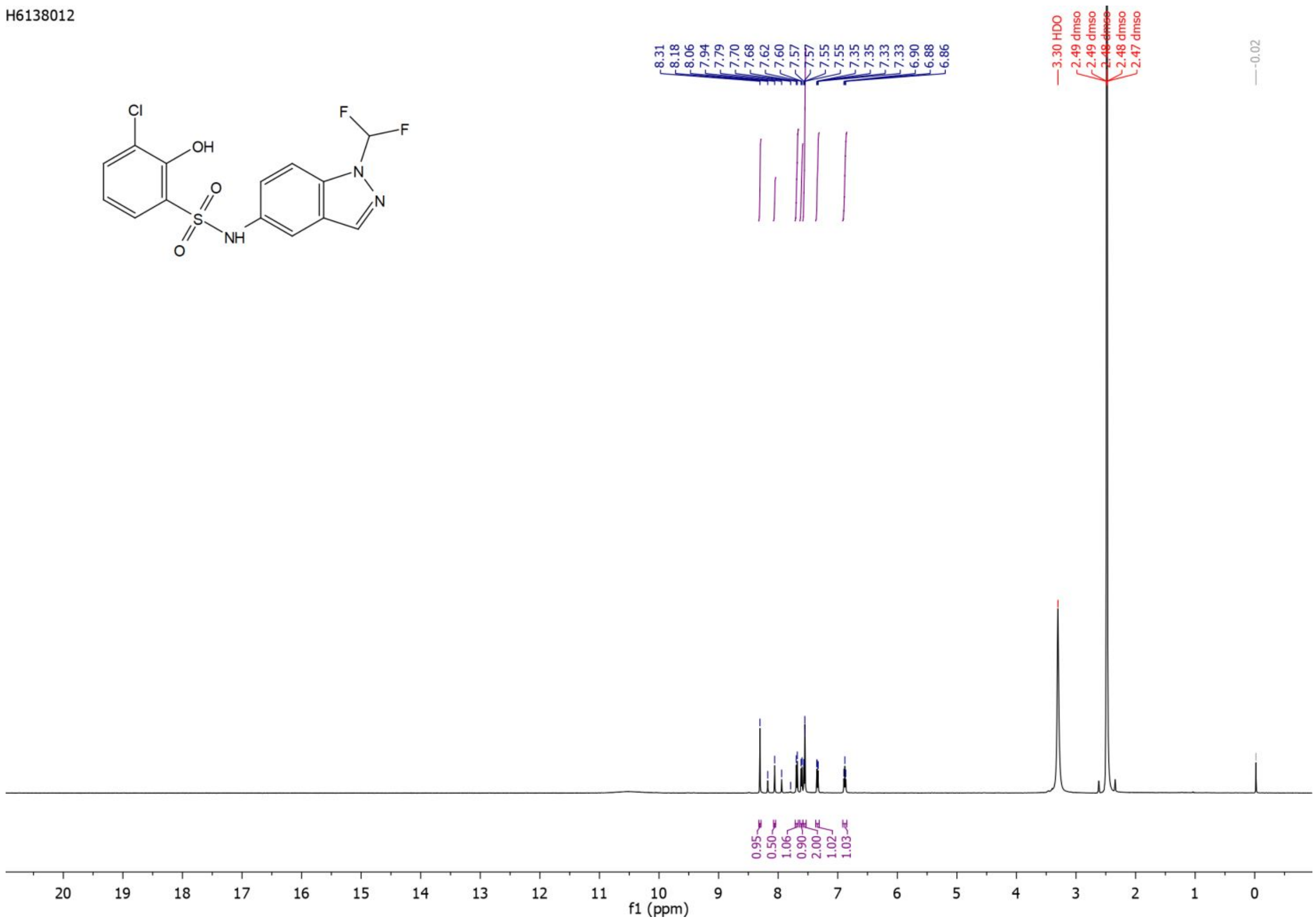

Z9544921868

MaxPeak: 99.34%
Ret_Time: 1.427 min

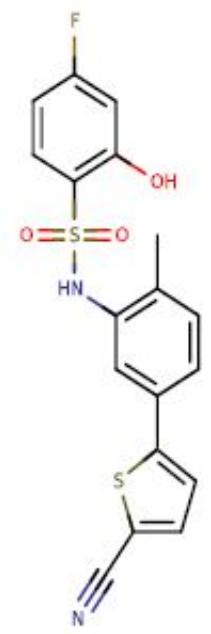

Mol Wt 388.44
Exact Mass 388.04

#	Time	Area%
1	1.106	0.66
2	1.427	99.34

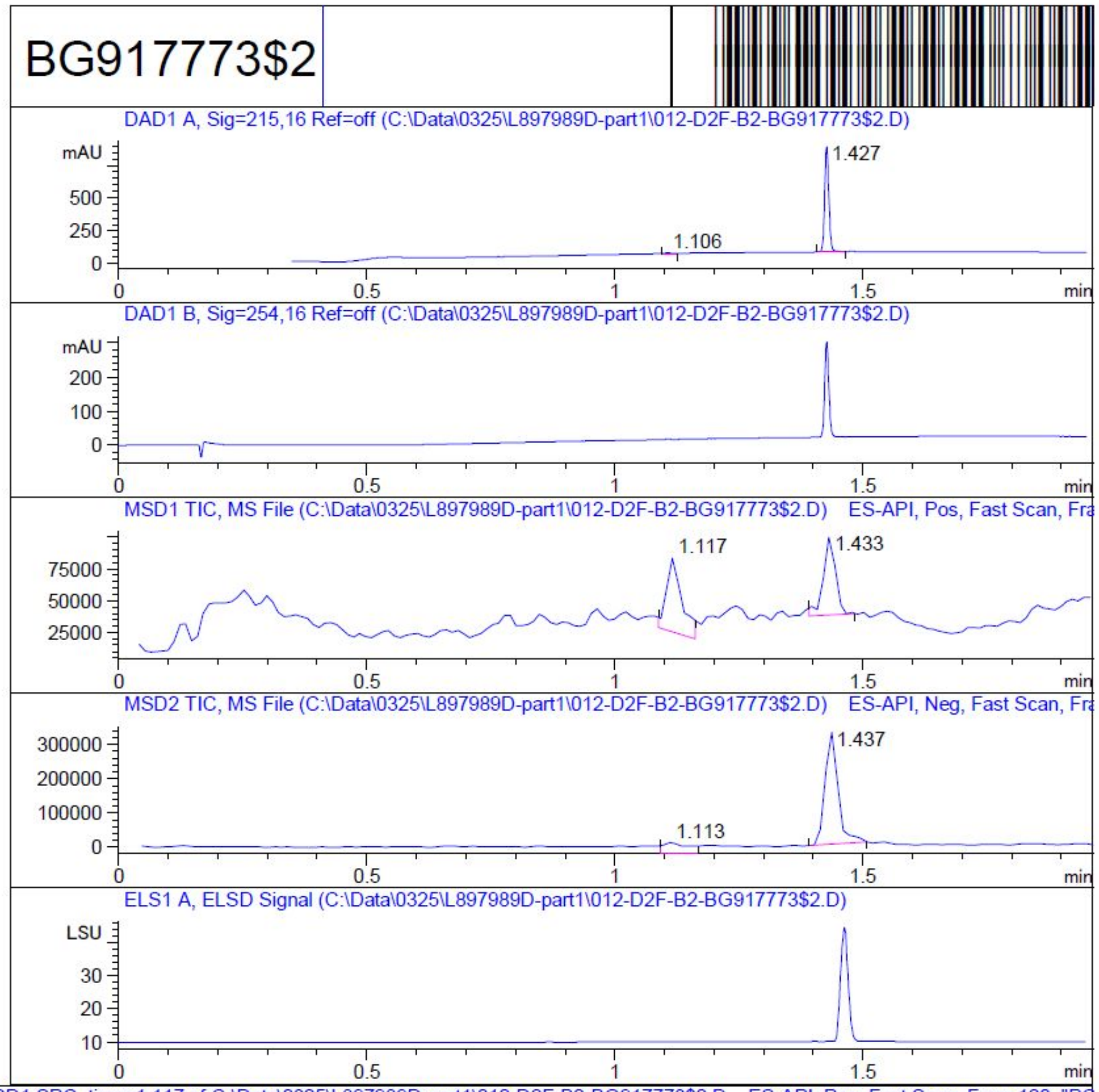

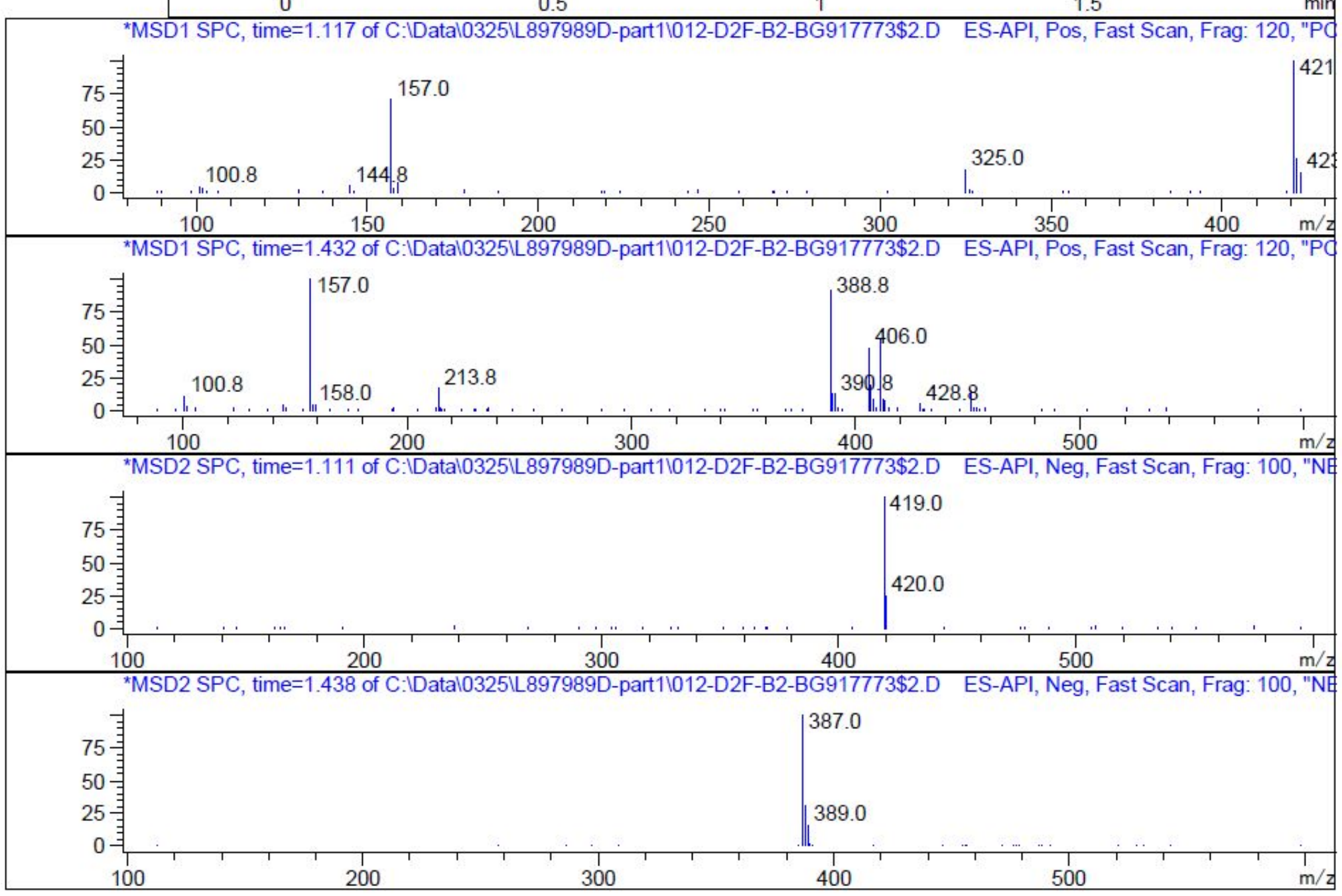

Z9544921957

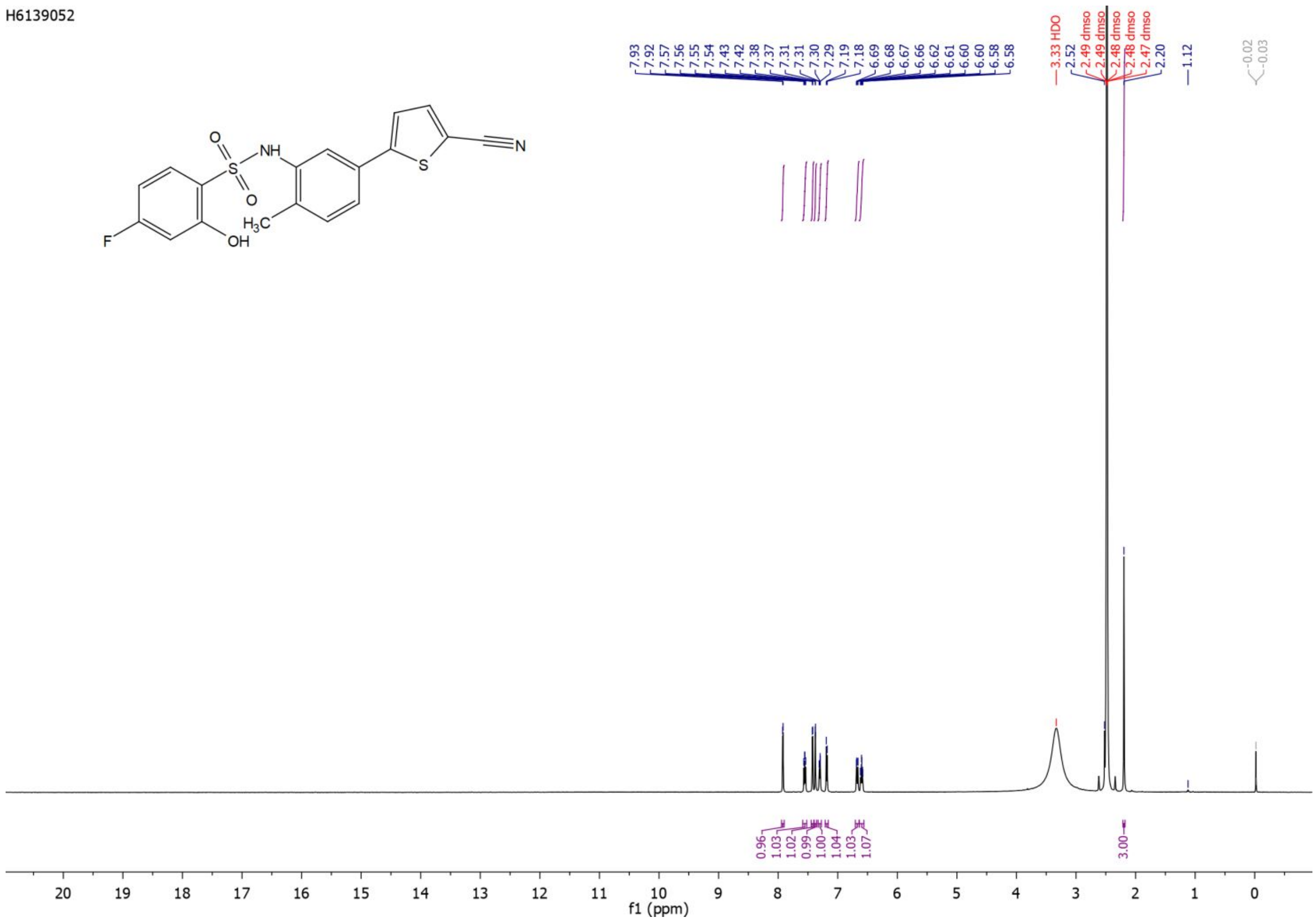

Z9544921957

MaxPeak: 98.20%
Ret_Time: 1.475 min

BG917788$4

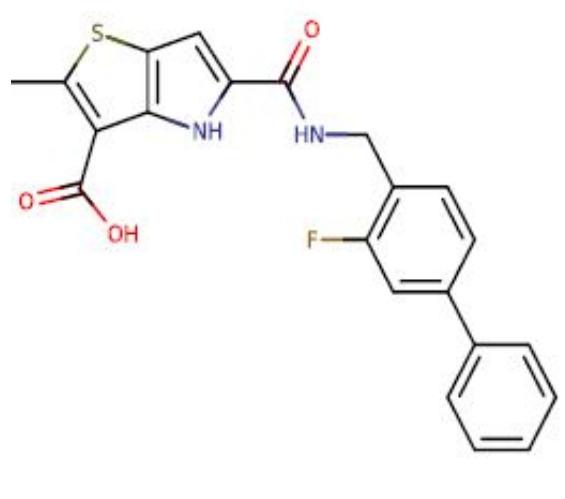

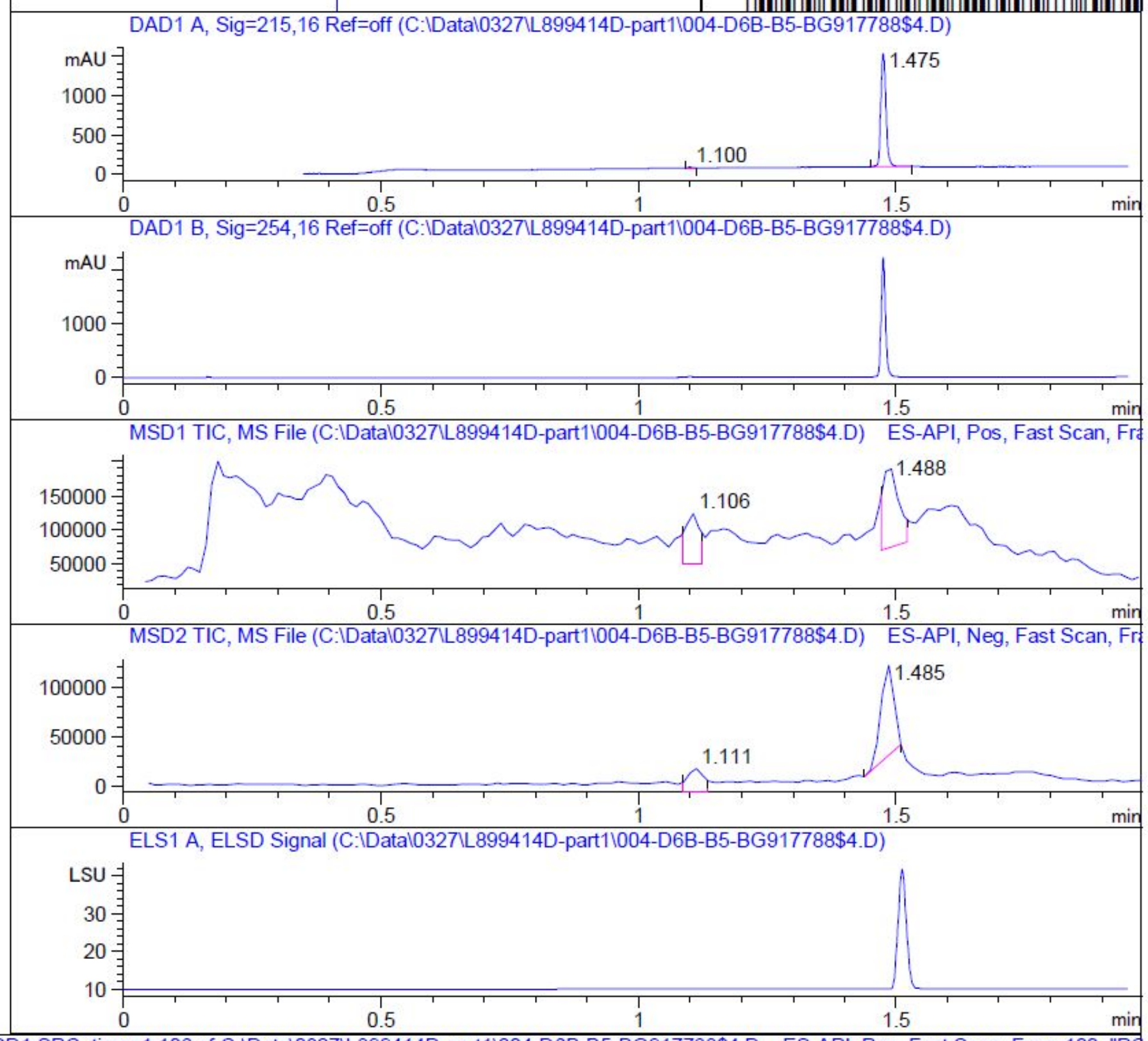

Mol Wt 408.45

Exact Mass 408.11

Time	Area%
1.100	1.80
1.475	98.20

RT 1.106

RT 1.488

RT 1.111

RT 1.485

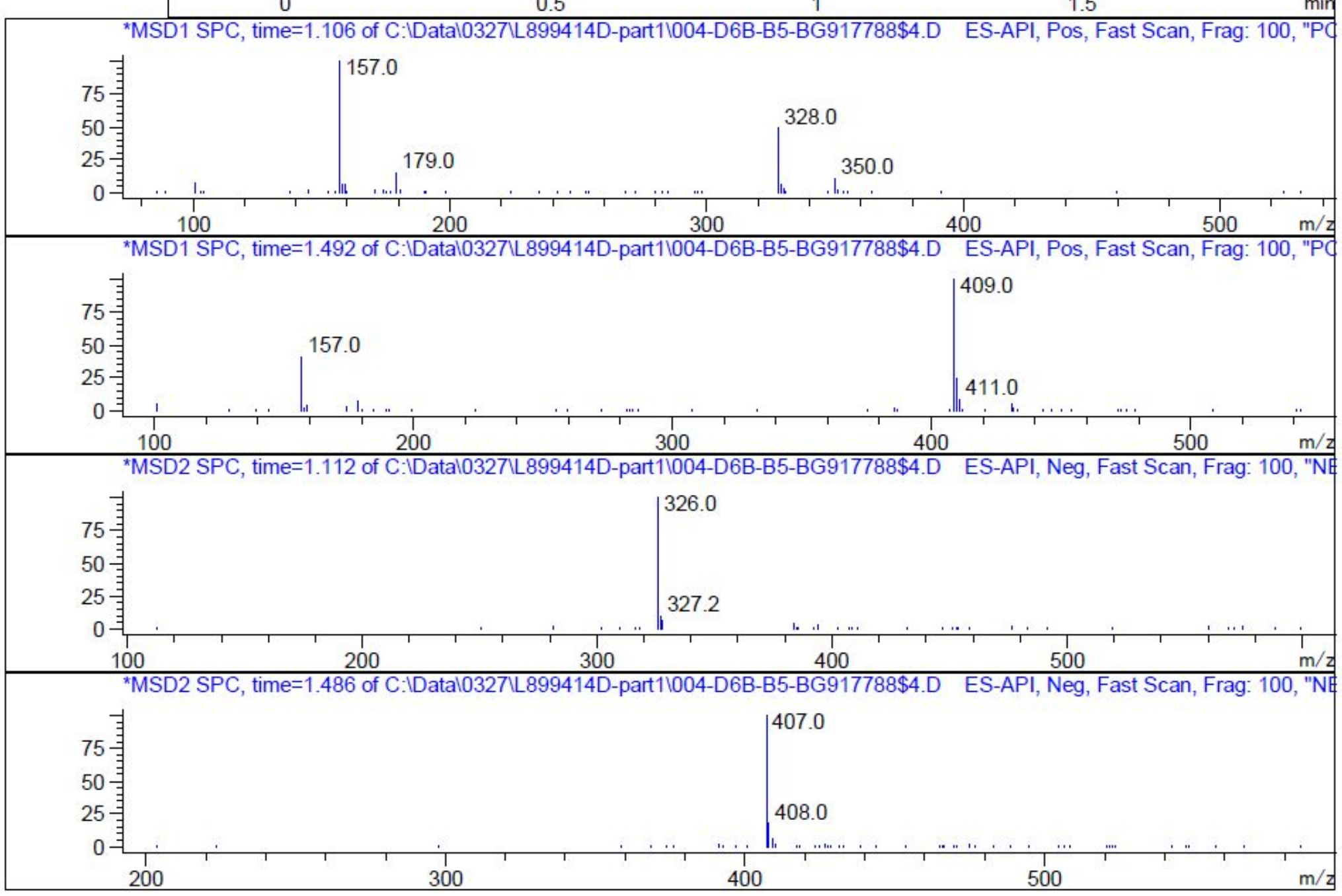

Z9544922018

H6144179

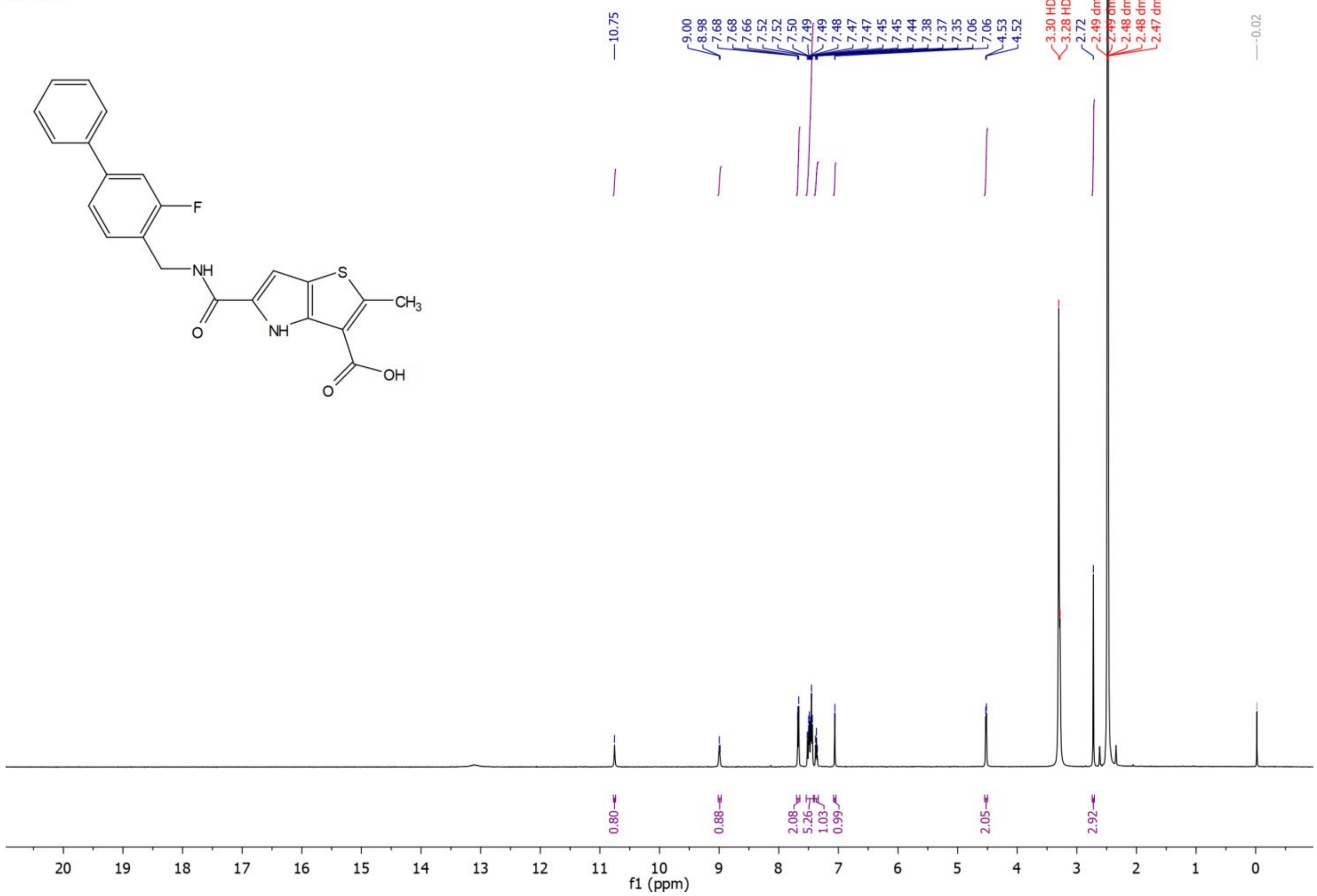

Z9544922018

MaxPeak: 100.00%
Ret_Time: 1.566 min

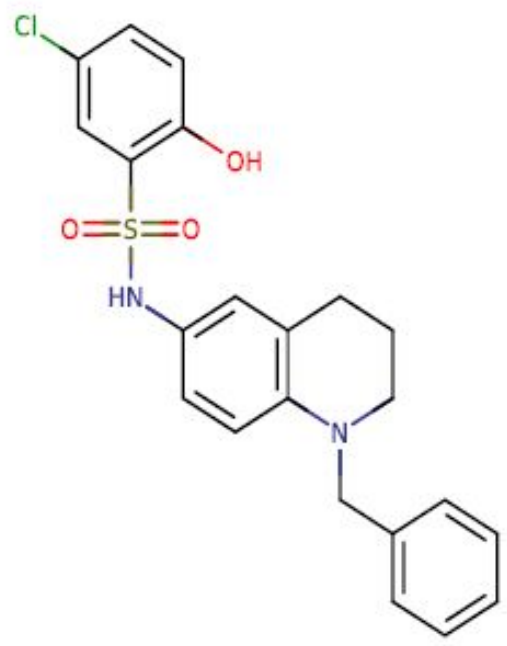

Mol Wt 428.93
Exact Mass 428.12

Time	Area%
1.566	100.00

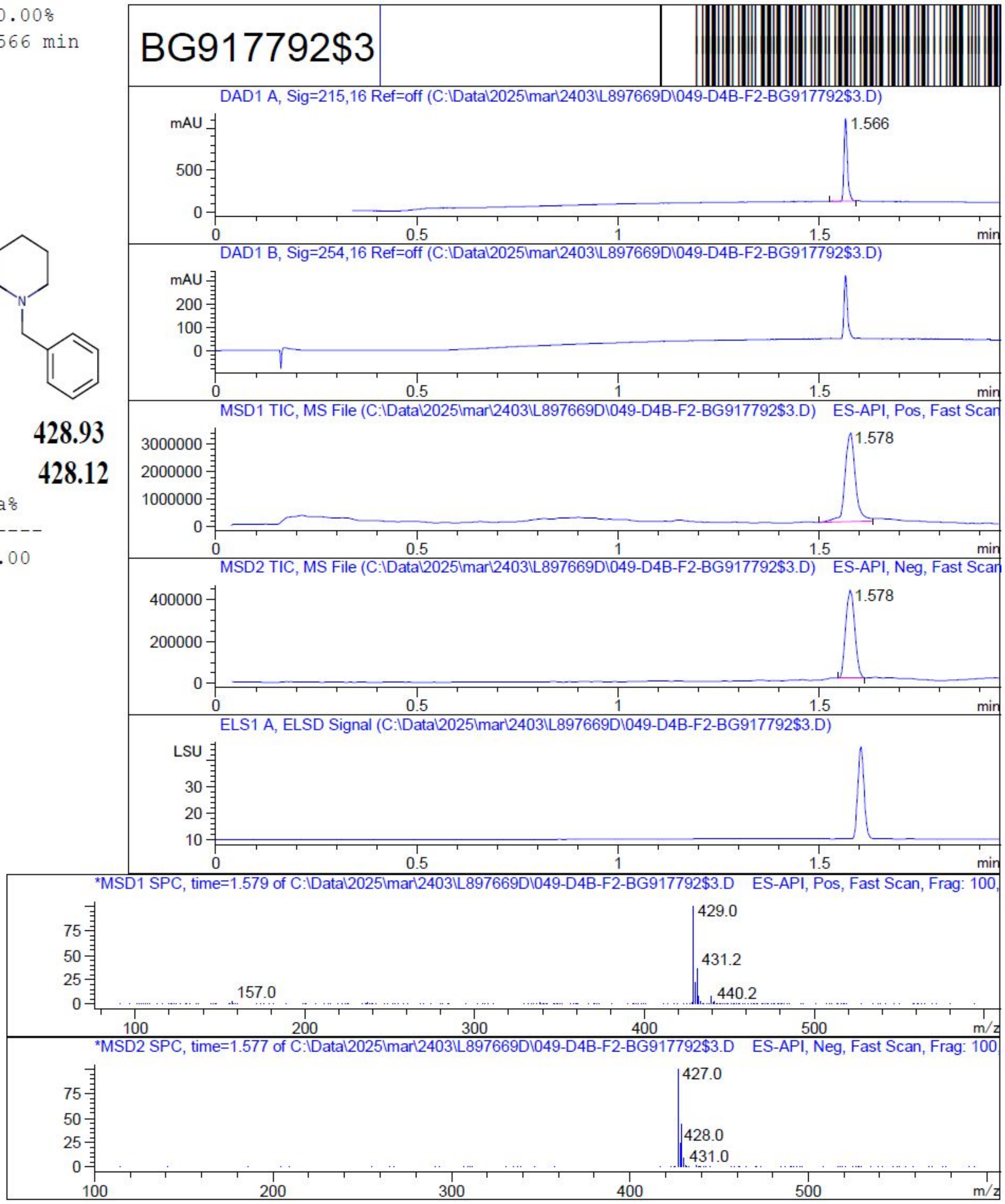

RT 1.578

RT 1.578

Z9544922043

H6137737

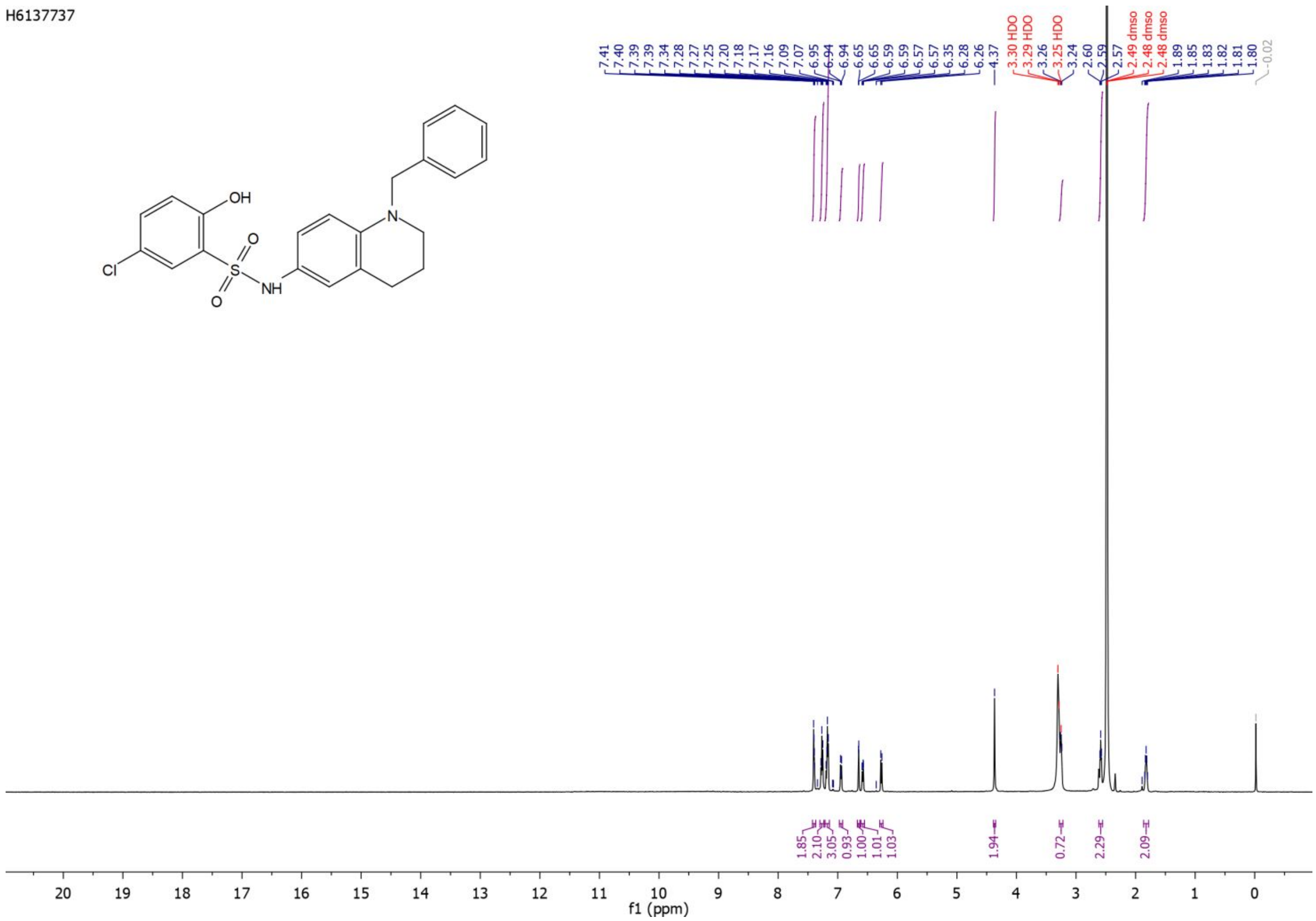

Z9544922043

MaxPeak: 96.54%
Ret_Time: 1.285 min

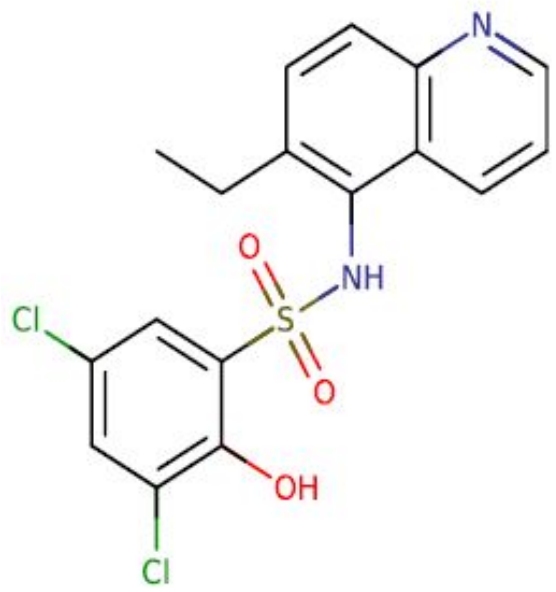

Mol Wt 397.28

Exact Mass 396.02

#	Time	Area%
1	1.170	2.11
2	1.205	1.34
3	1.285	96.54

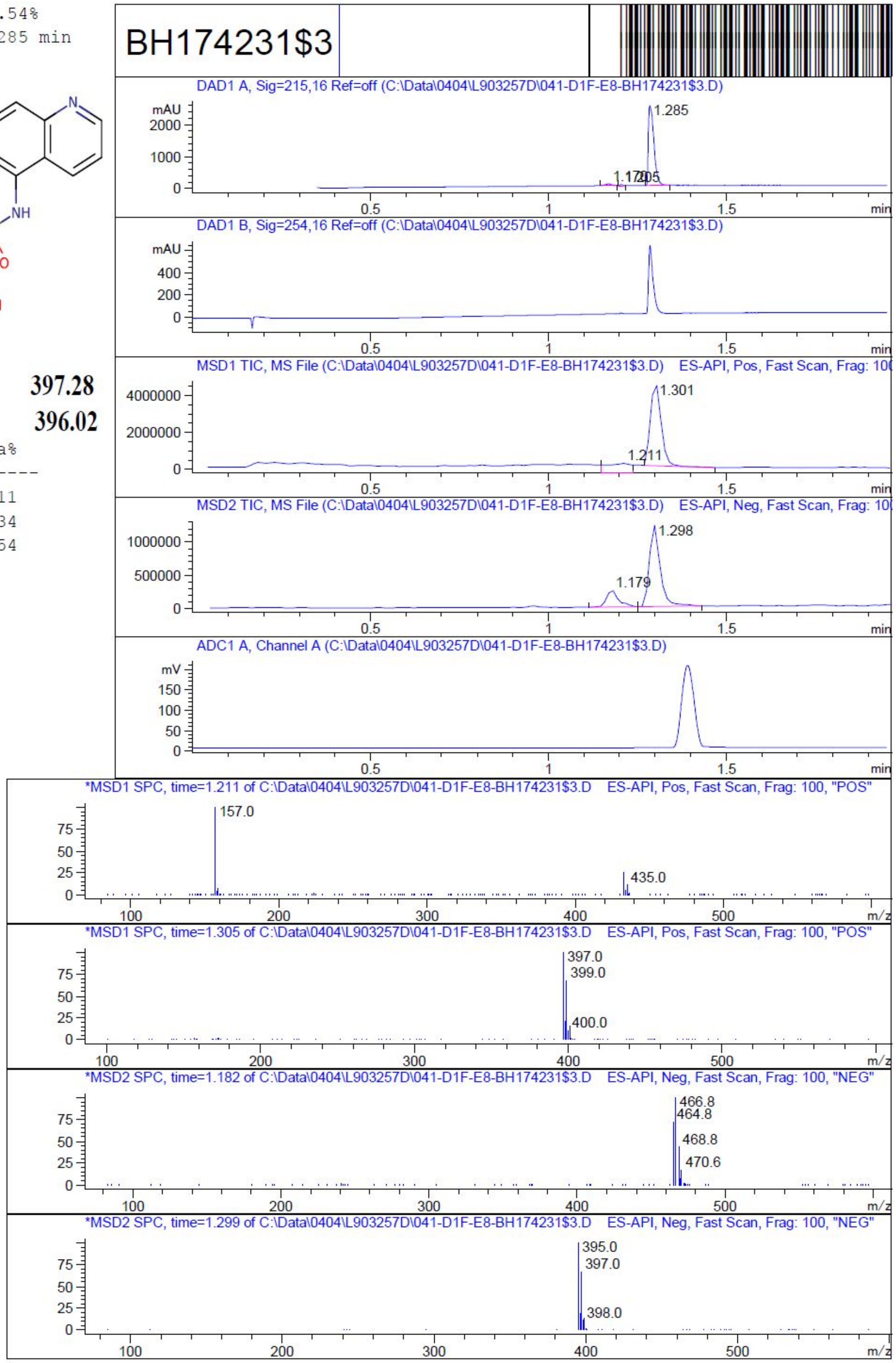

RT 1.211

RT 1.301

RT 1.179

RT 1.298

Z9544922503

H6164335

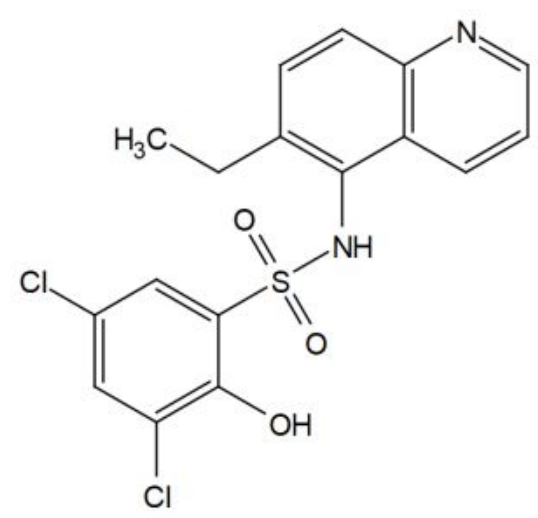

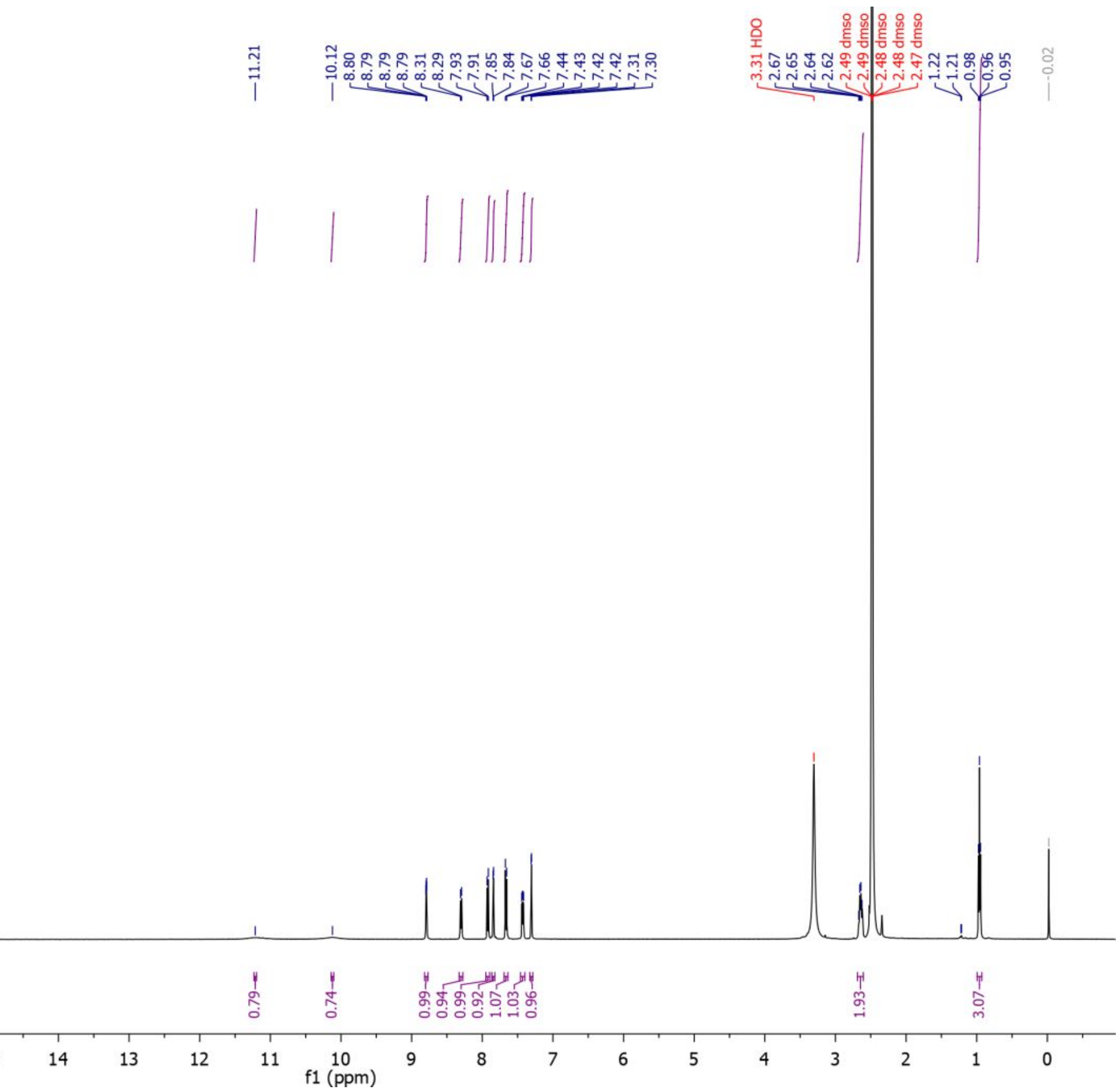

Z9544922503

```
MaxPeak:  96.02%
Ret_Time: 1.153 min
```

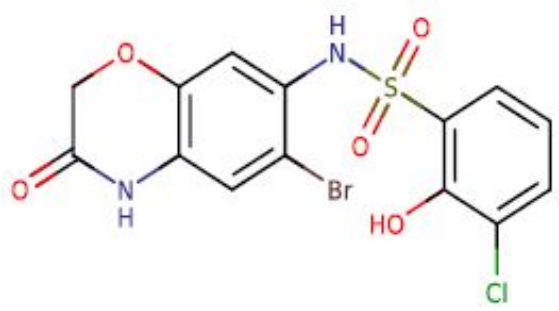

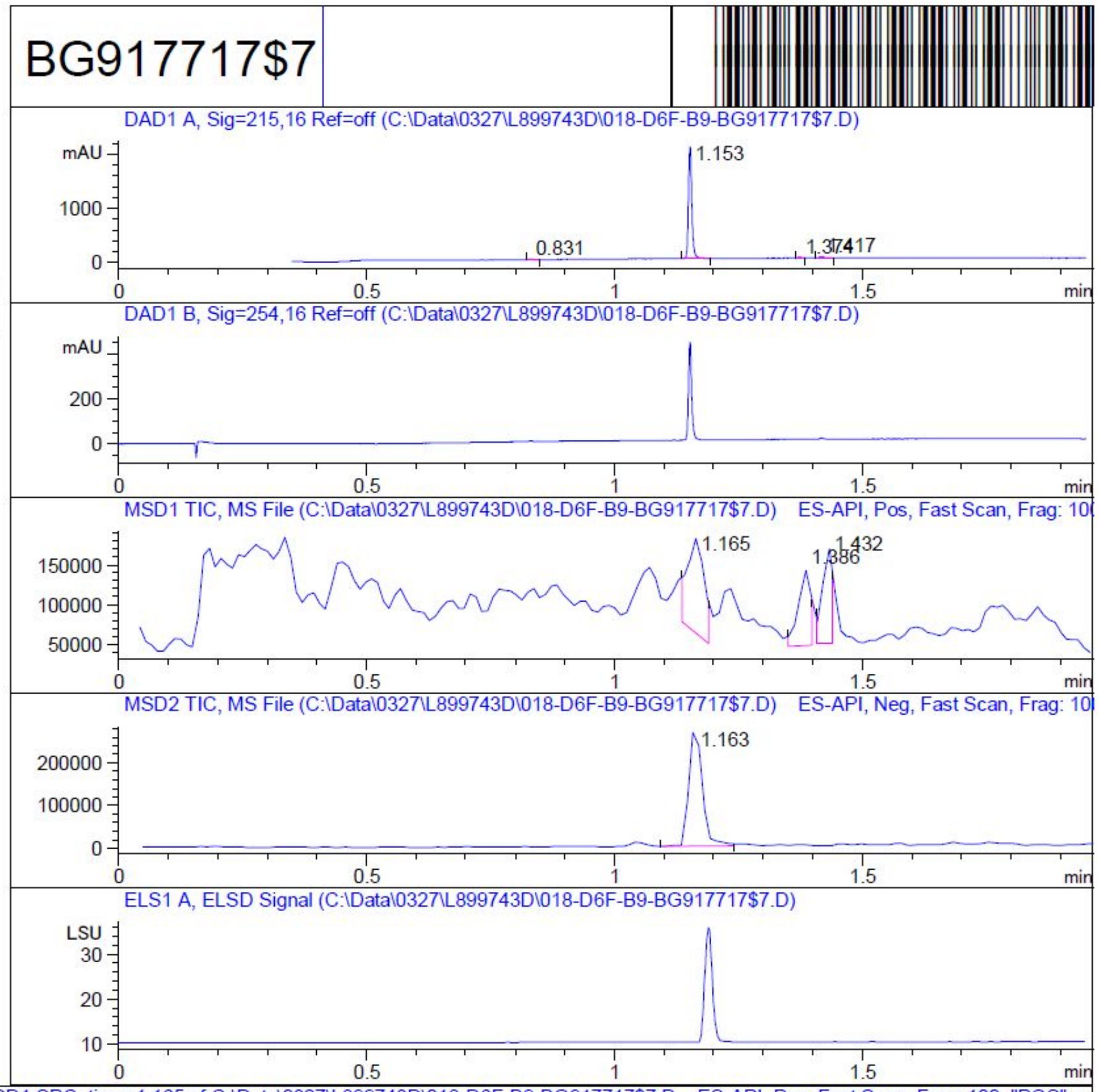

Mol Wt 433.66

Exact Mass 433.91

#	Time	Area%
1	0.831	1.33
2	1.153	96.02
3	1.374	1.09
4	1.417	1.56

RT 1.165

RT 1.386

RT 1.432

RT 1.163

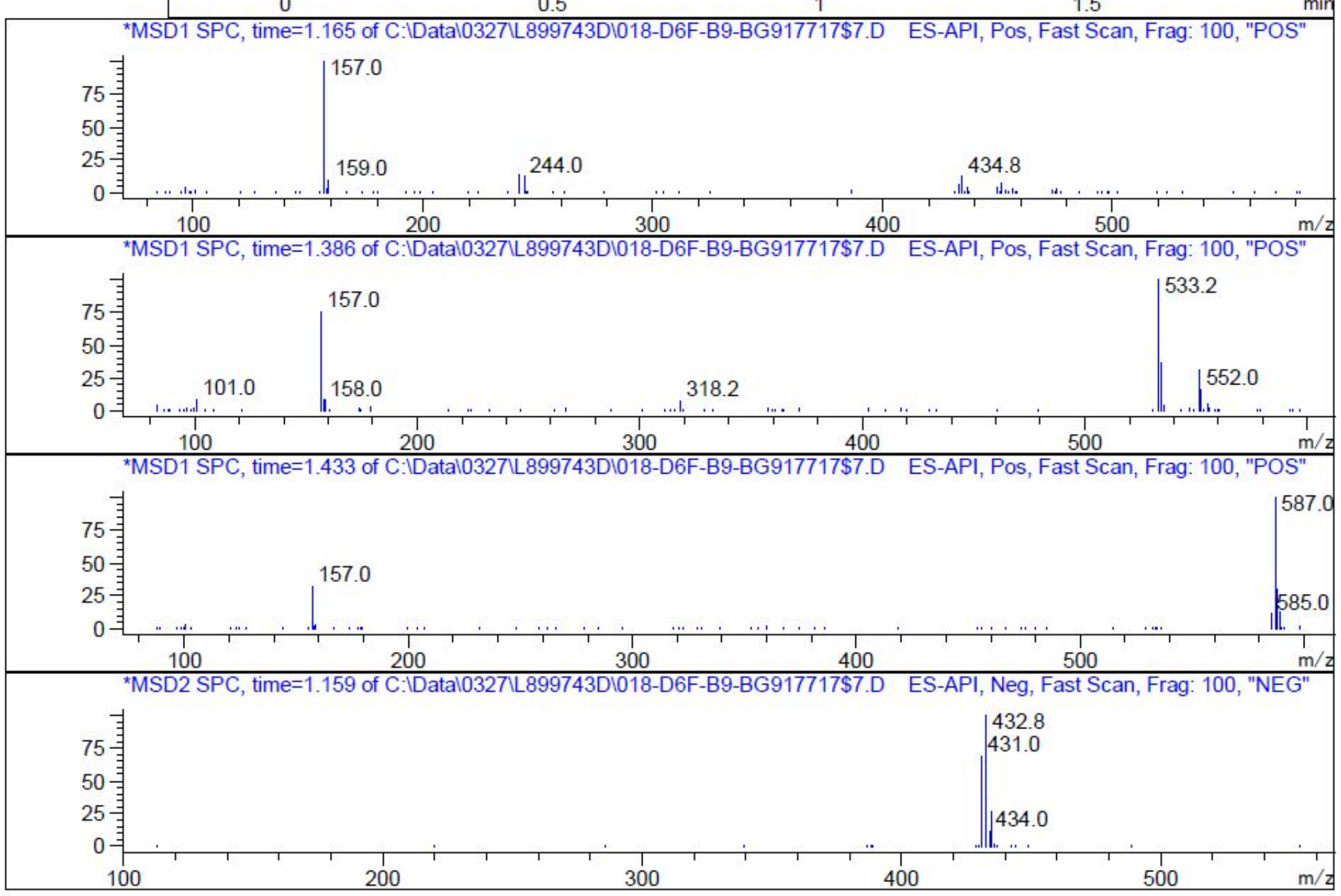

Z9544921818

MaxPeak: 100.00%
Ret_Time: 1.035 min

BG917644$2

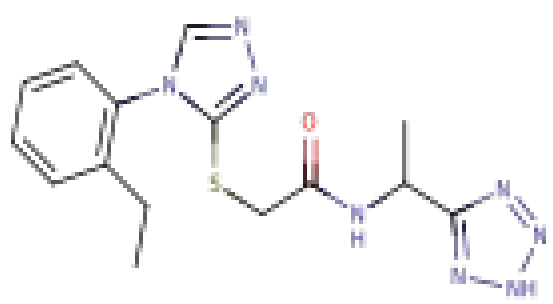

Mol Wt 358.42

Exact Mass 358.14

#	Time	Area%
1	1.035	100.00

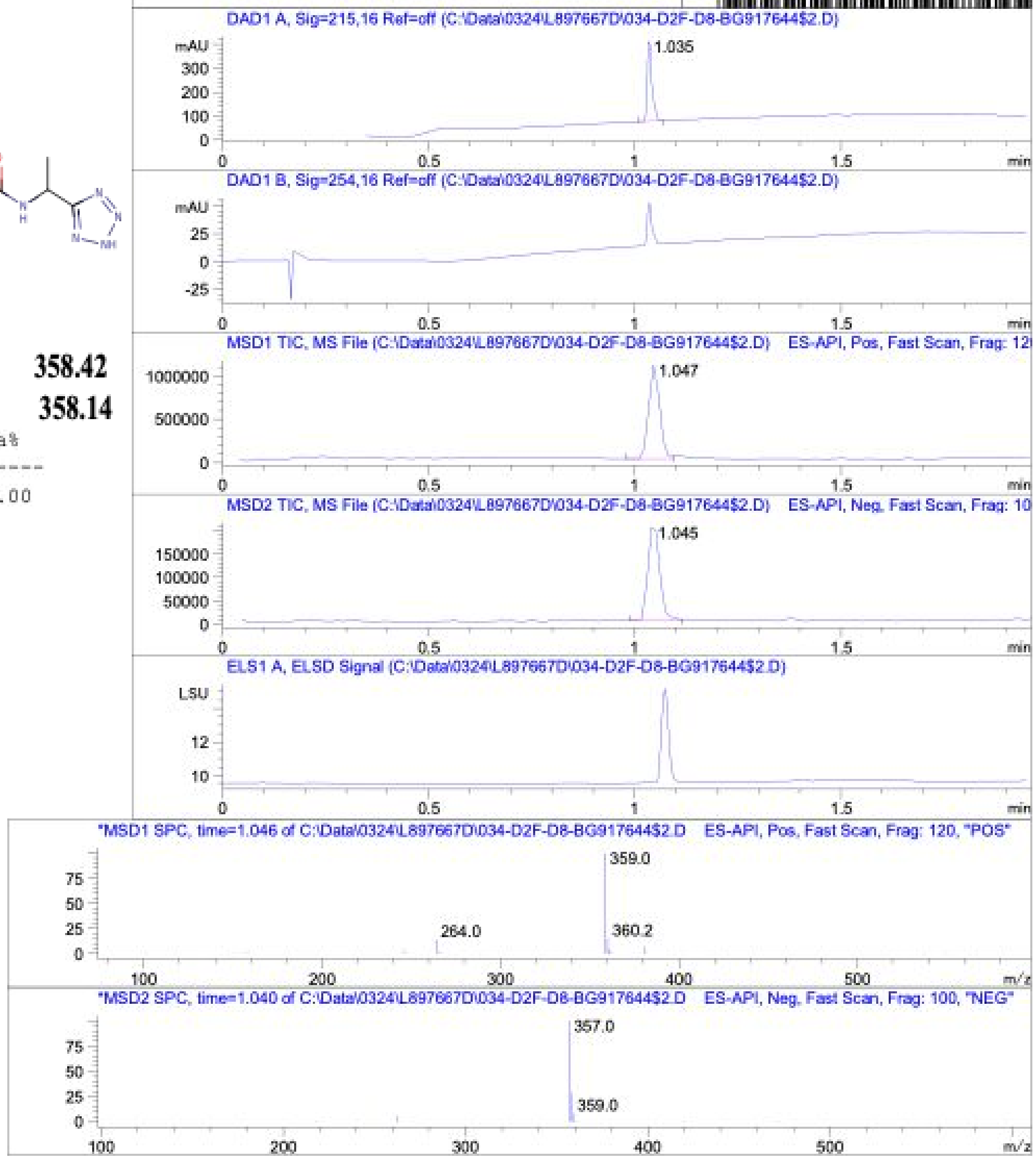

RT 1.047

RT 1.045

Inj.Date 3/22/2025 NEW -21-
C:\Users\Public\Documents\ChemStation\1\Data\03_21\L897667D\SUPOR_30.M

Z605931832

MaxPeak: 100.00%
Ret_Time: 1.193 min

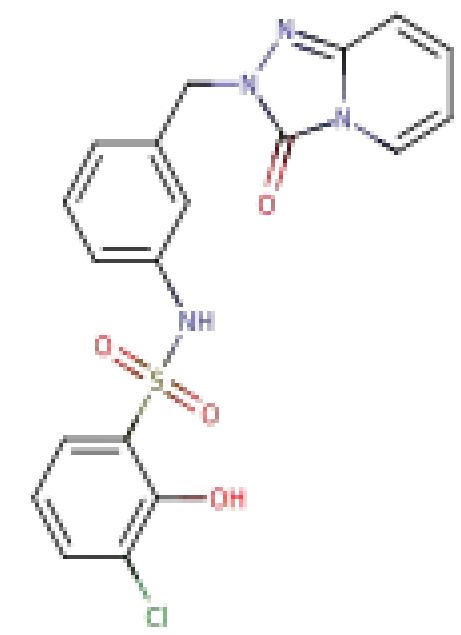

Mol Wt 430.86

Exact Mass 430.05

#	Time	Area%
1	1.193	100.00

BF054166$1

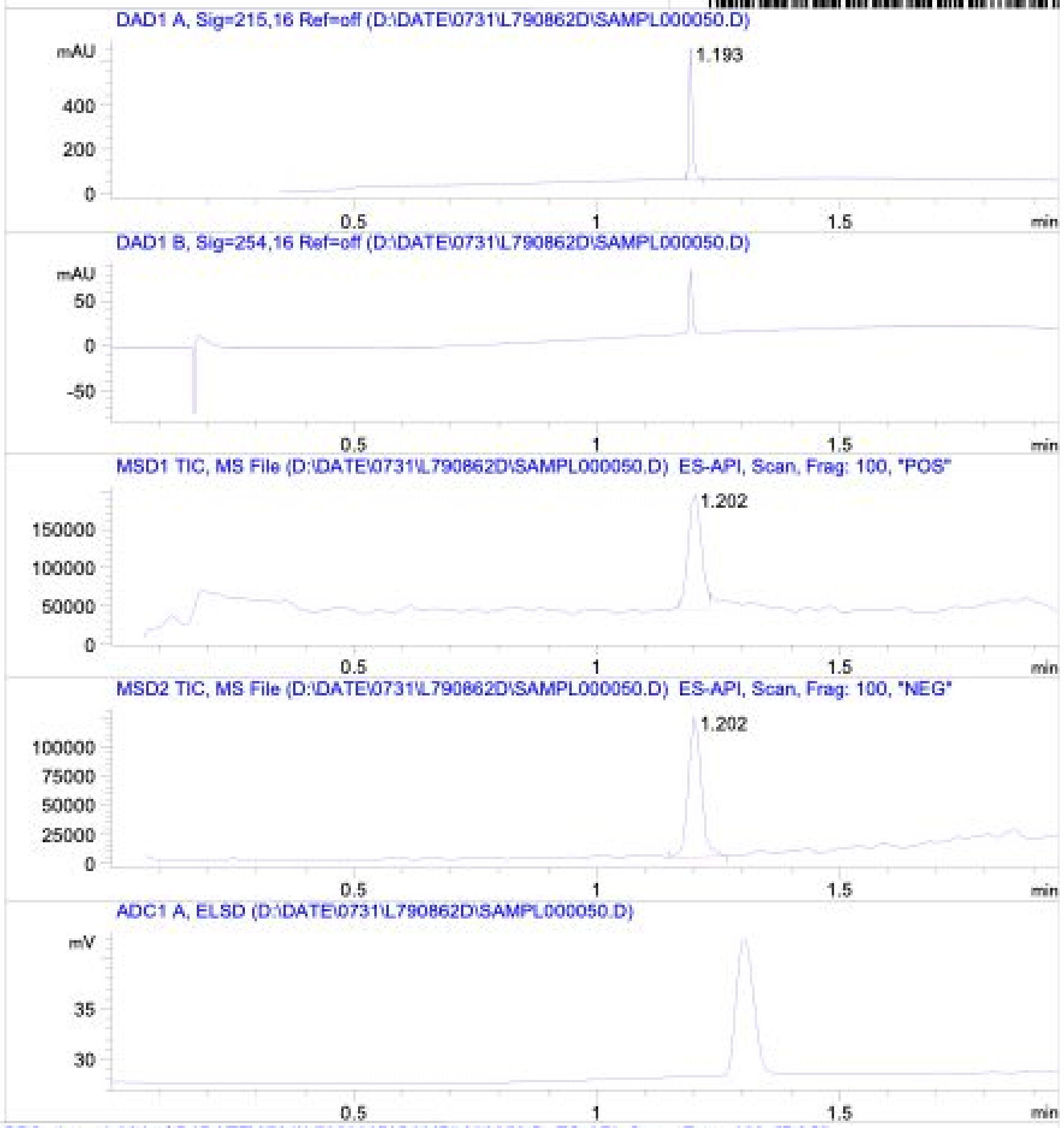

RT 1.202

RT 1.202

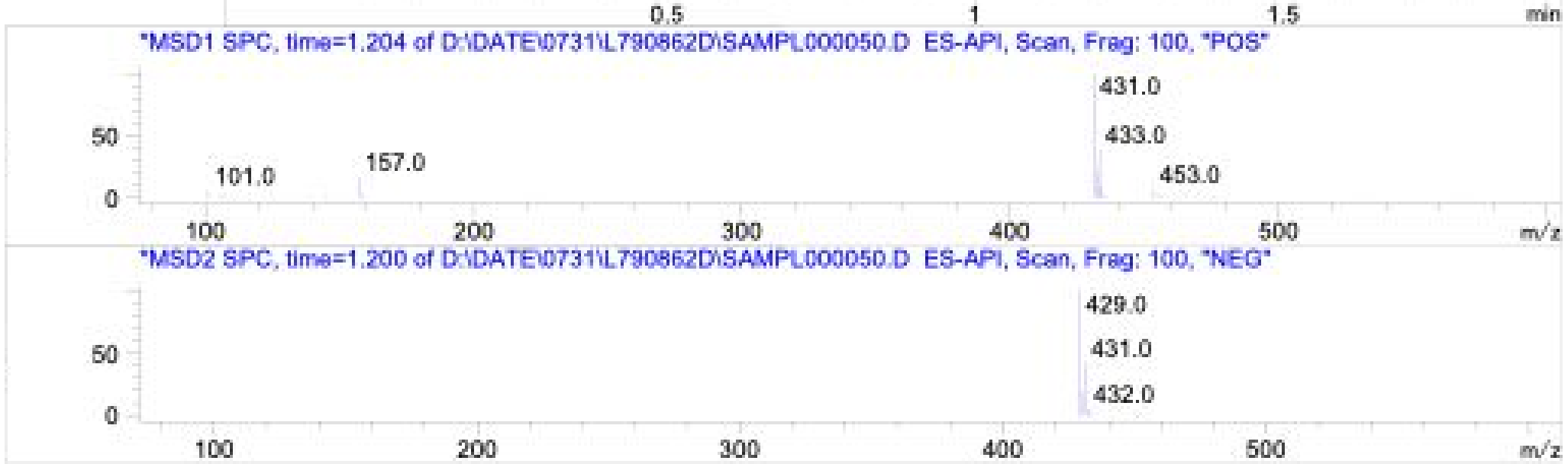

Inj.Date 7/31/2024 O -6-
C:\CHEM32\1\METHODS\SUPOR_30.M

Z2027058992

MaxPeak: 100.00%
Ret_Time: 1.354 min

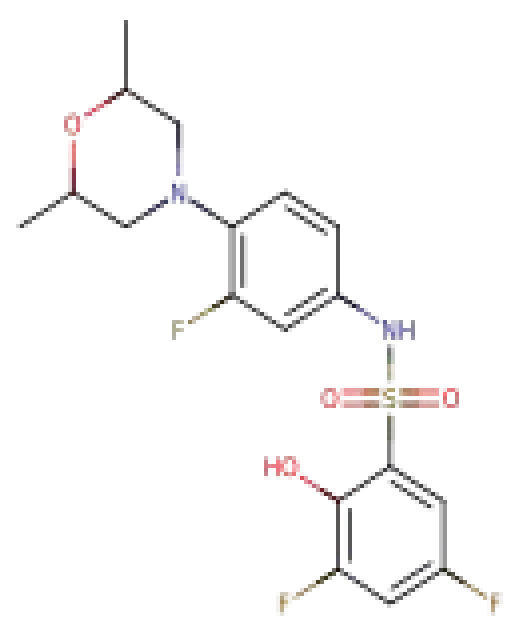

Mol Wt 416.42

Exact Mass 416.12

#	Time	Area%
1	1.354	100.00

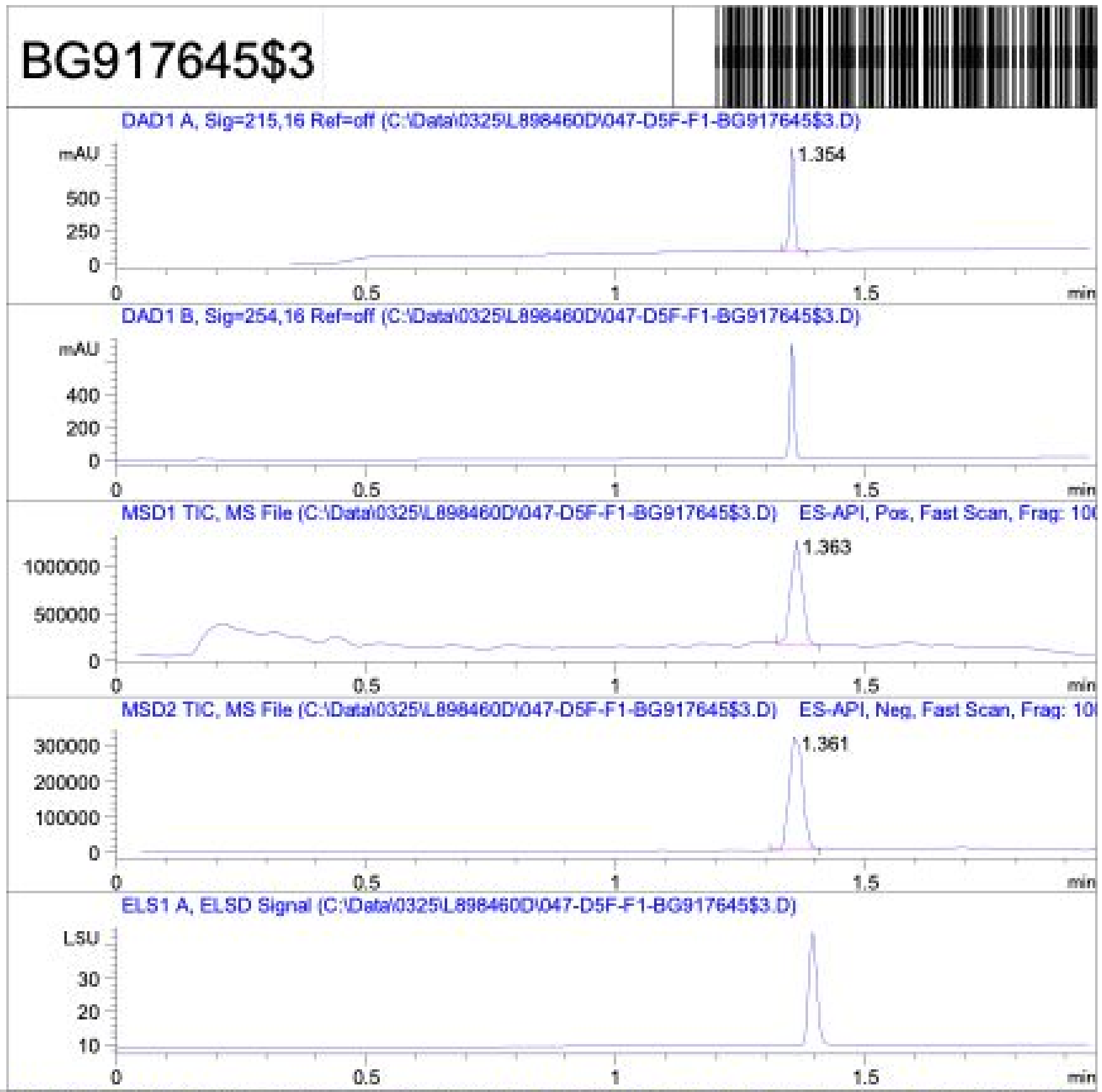

RT 1.363

RT 1.361

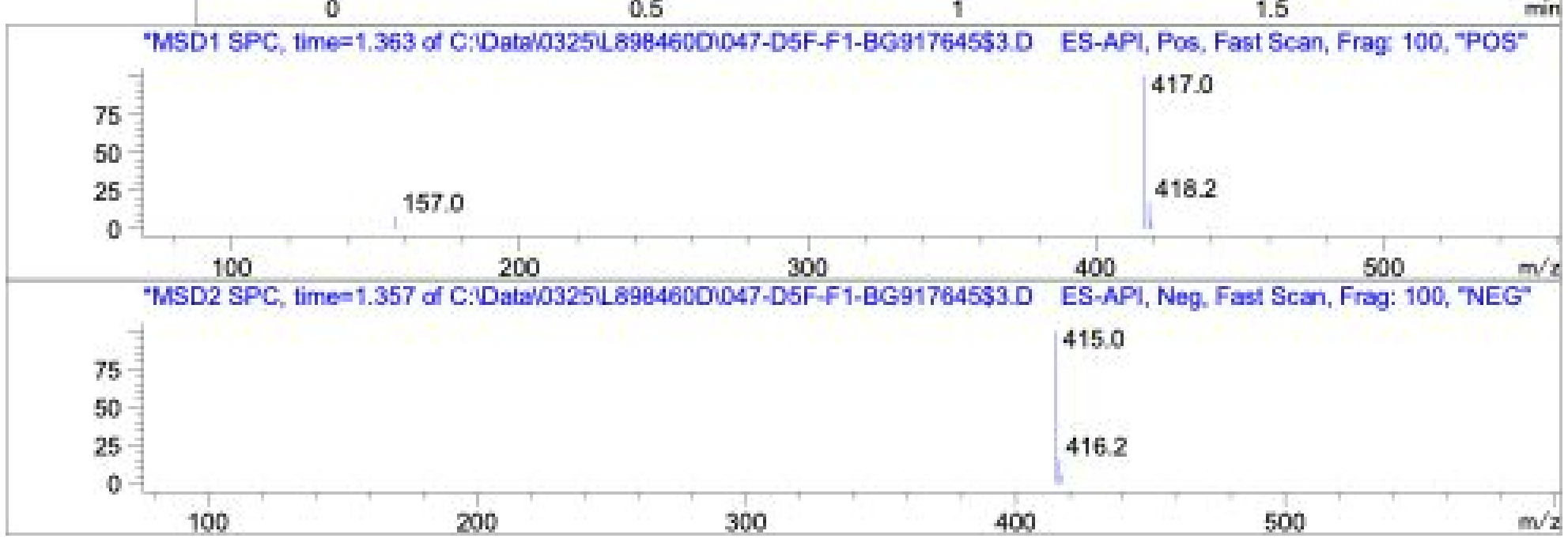

Inj.Date 3/24/2025 KCH -35-
C:\Users\Public\Documents\ChemStation\1\Data\03_24\L898460D\SUPOR_30.M

Z2027059519

MaxPeak: 96.05%
Ret_Time: 1.420 min

BG917648$2

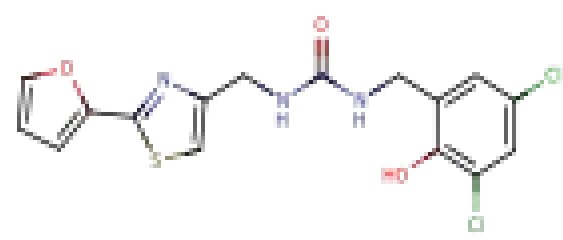

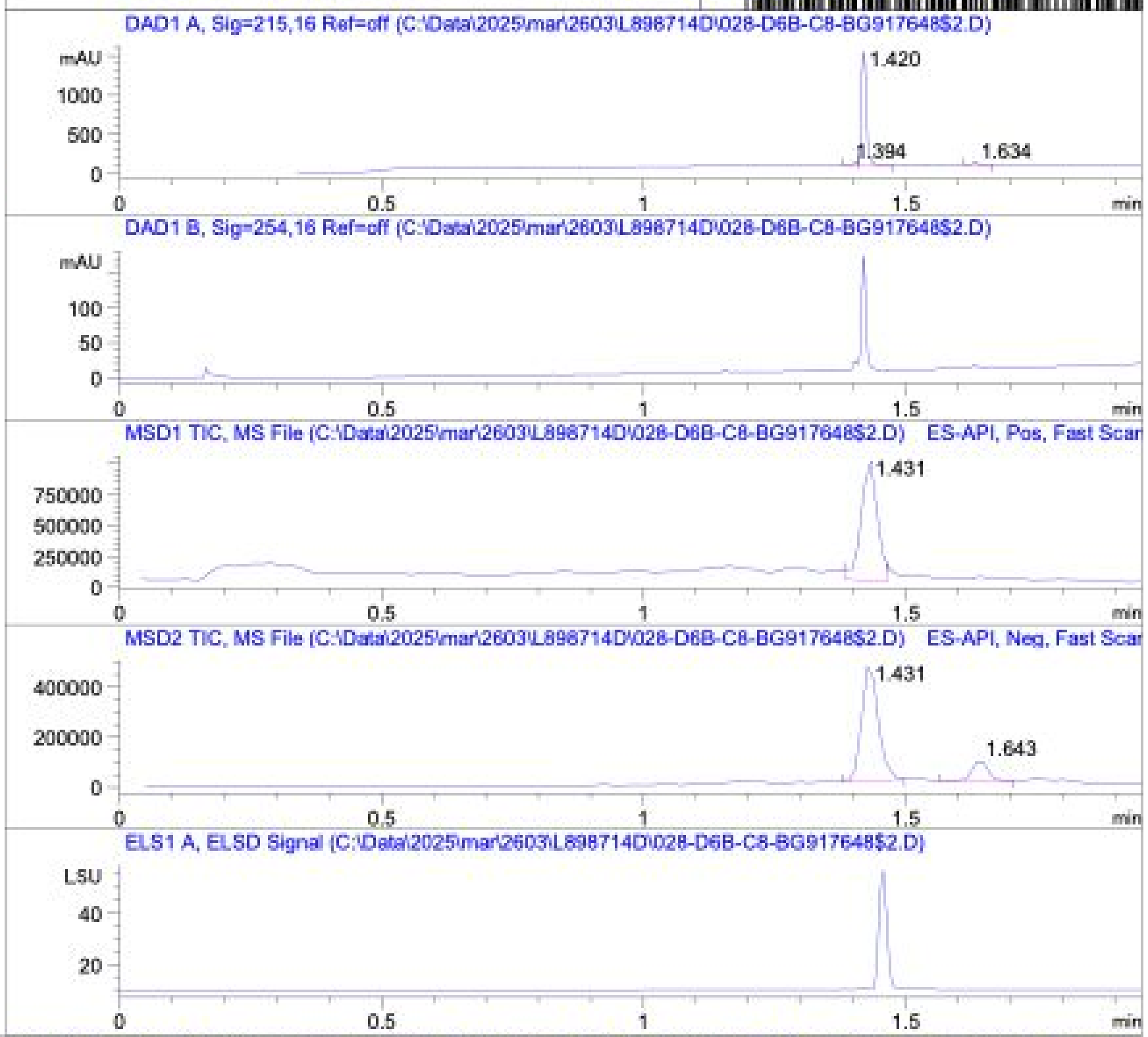

Mol Wt 398.26

Exact Mass 397.01

#	Time	Area%
1	1.394	1.69
2	1.420	96.05
3	1.634	2.26

RT 1.431

RT 1.431

RT 1.643

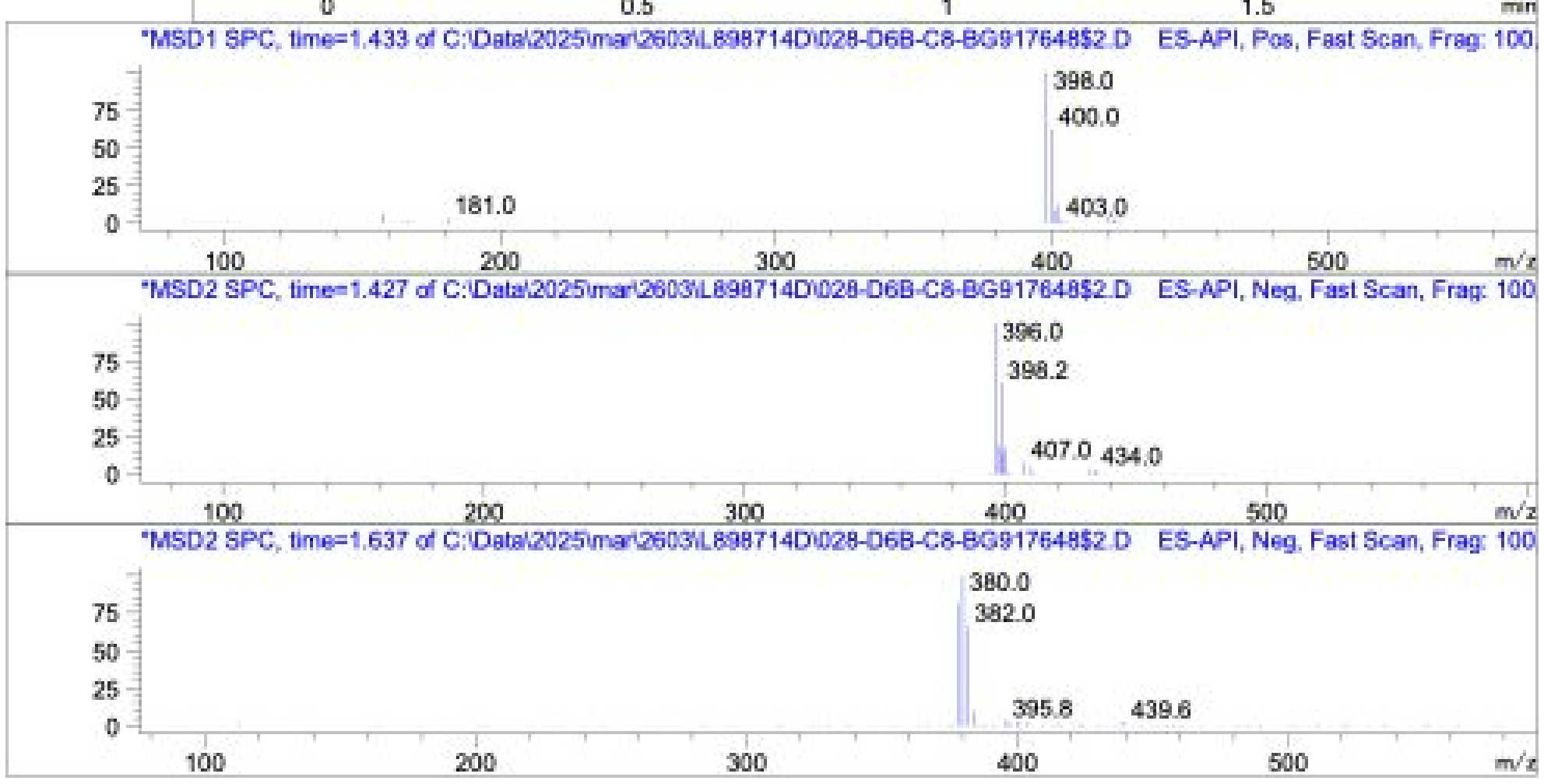

Inj.Date 3/26/2025 NEW -33-
C:\Users\Public\Documents\ChemStation\1\Data\03_25\L898714D\SUPOR_30.M

Z2117758721

MaxPeak: 100.00%
Ret_Time: 1.404 min

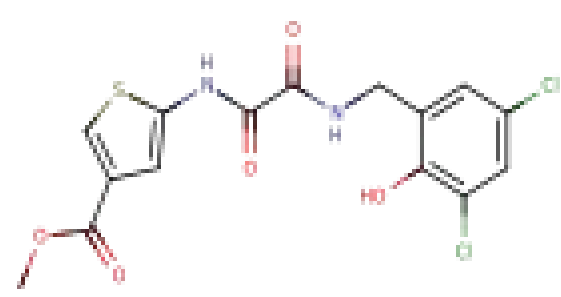

Mol Wt 403.24
Exact Mass 401.98

#	Time	Area%
1	1.404	100.00

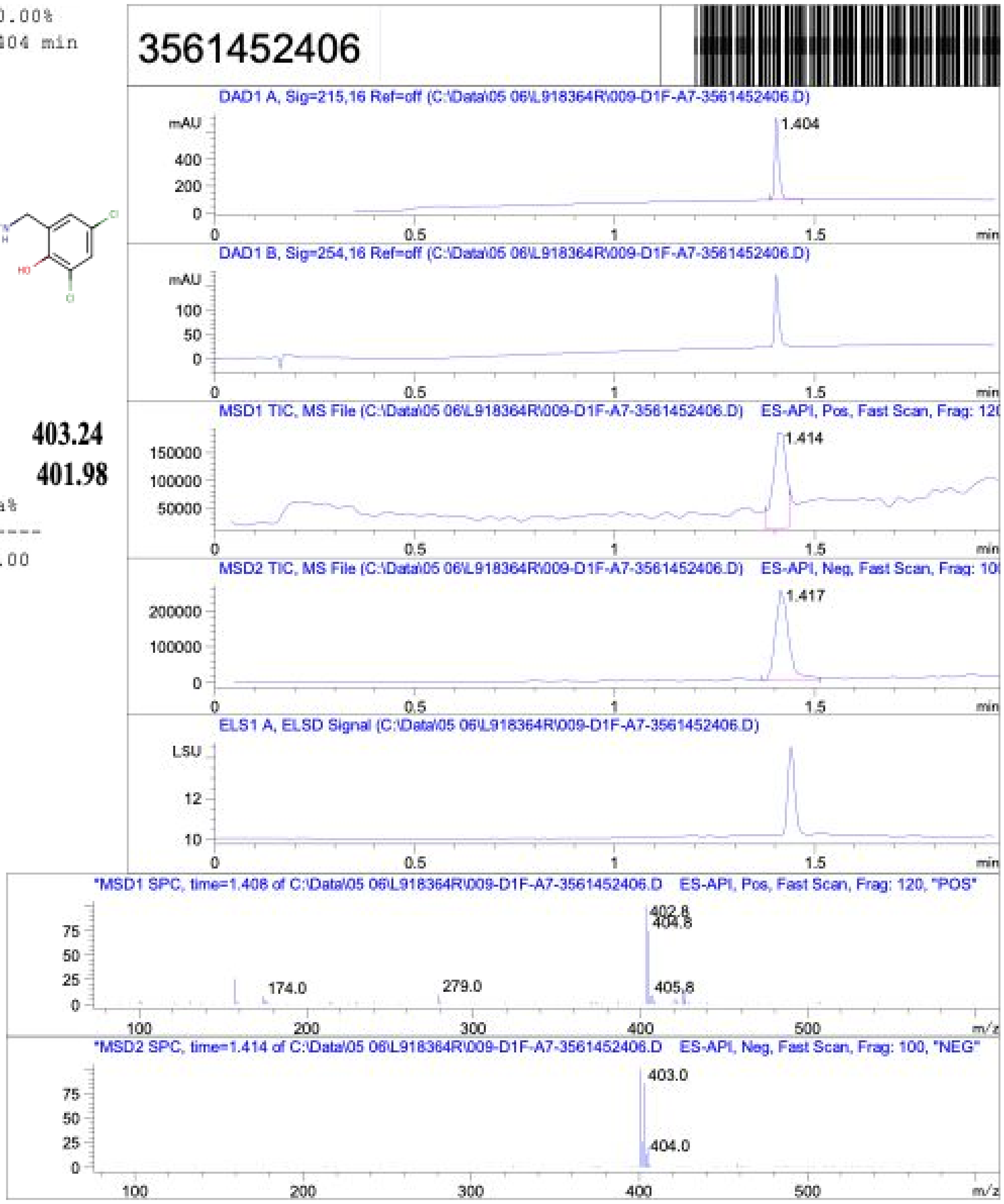

RT 1.414

RT 1.417

Inj.Date 5/6/2025 KCH -21-
C:\Users\Public\Documents\ChemStation\1\Data\05_06\L918364R\SUPOR_30.M

Z2127627626

MaxPeak: 100.00%
Ret_Time: 1.242 min

BG917655$2

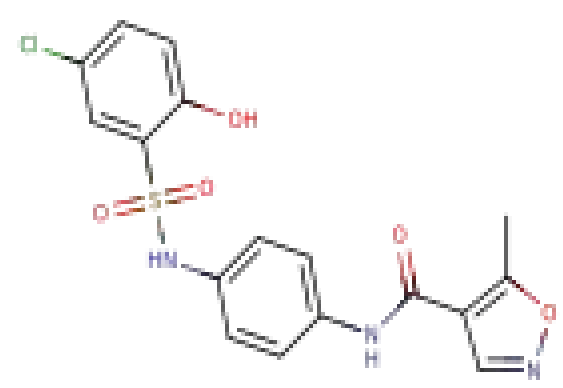

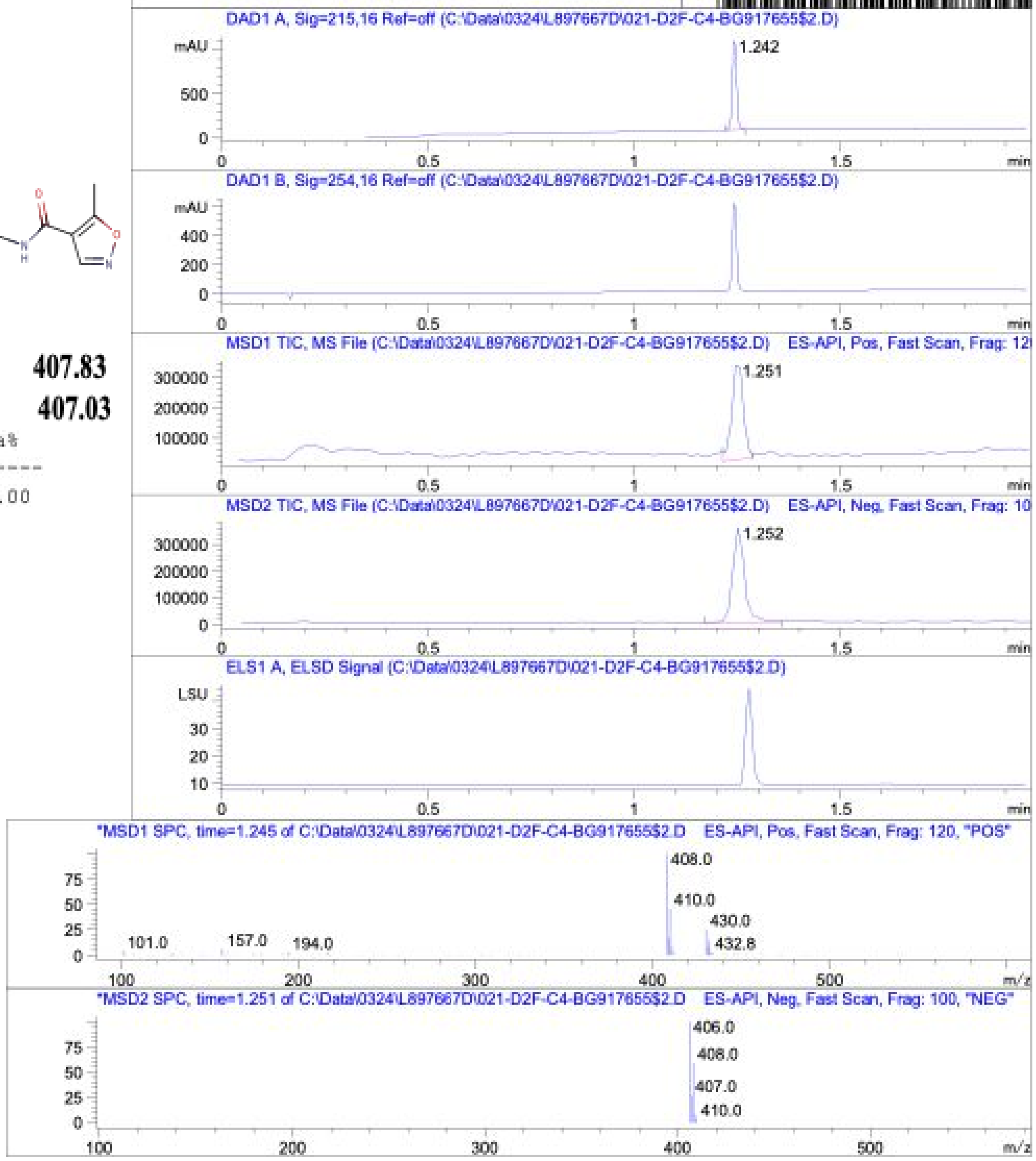

Mol Wt 407.83

Exact Mass 407.03

#	Time	Area%
1	1.242	100.00

RT 1.251

RT 1.252

Inj.Date 3/22/2025 NEW -21-

C:\Users\Public\Documents\ChemStation\1\Data\03_21\L897667D\SUPOR_30.M

Z2234672760

MaxPeak: 100.00%
Ret_Time: 1.392 min

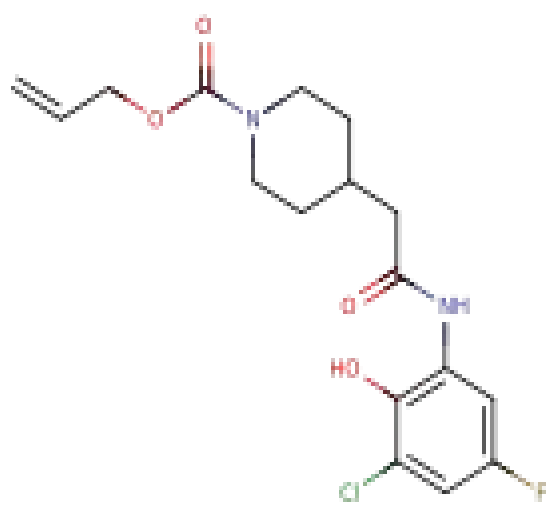

Mol Wt 370.8
Exact Mass 370.13

#	Time	Area%
1	1.392	100.00

BH182988$1

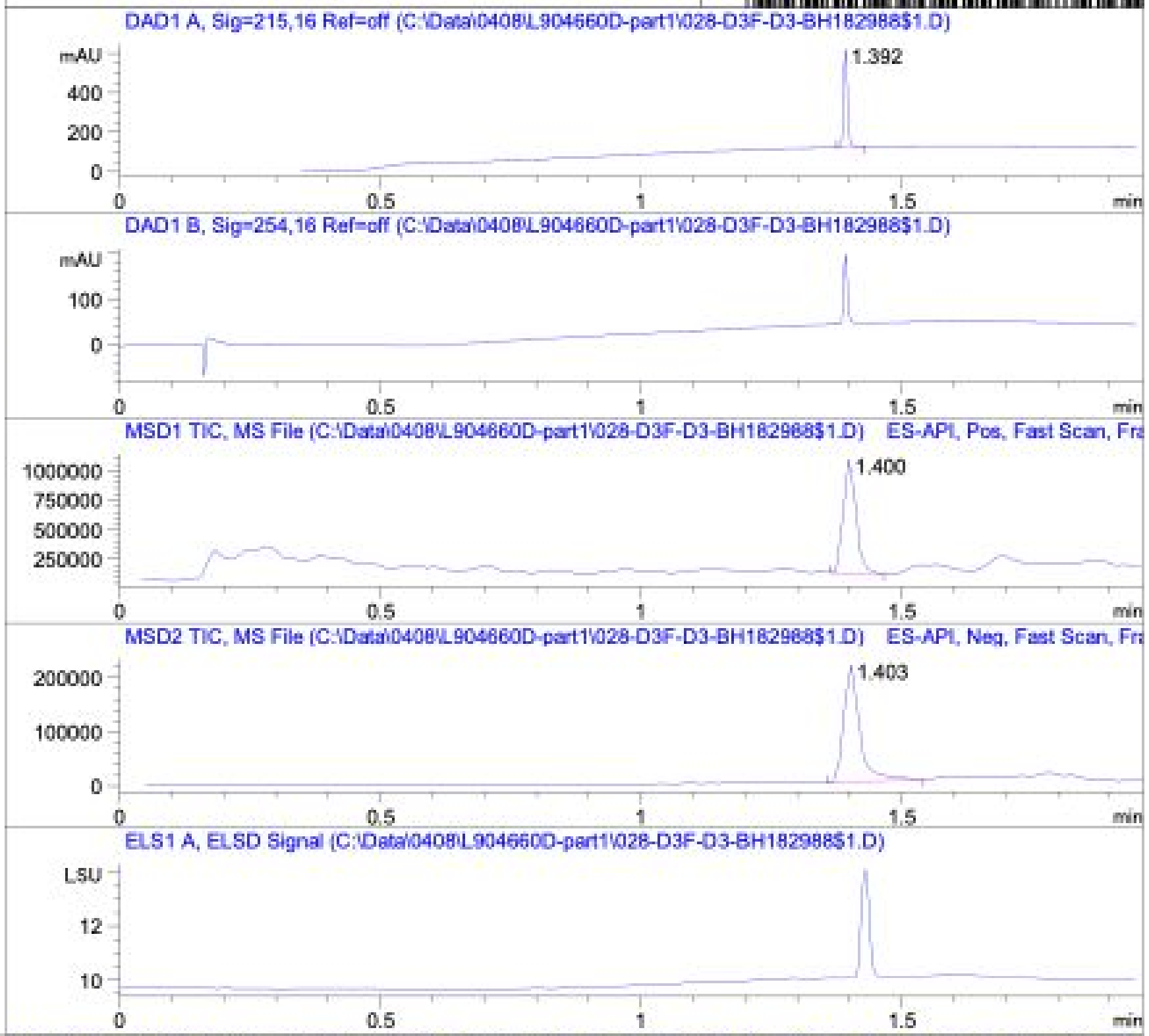

RT 1.400

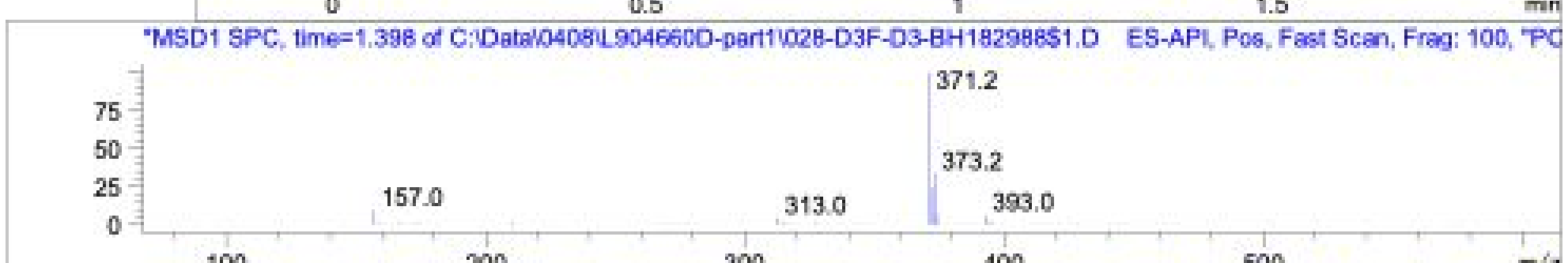

RT 1.403

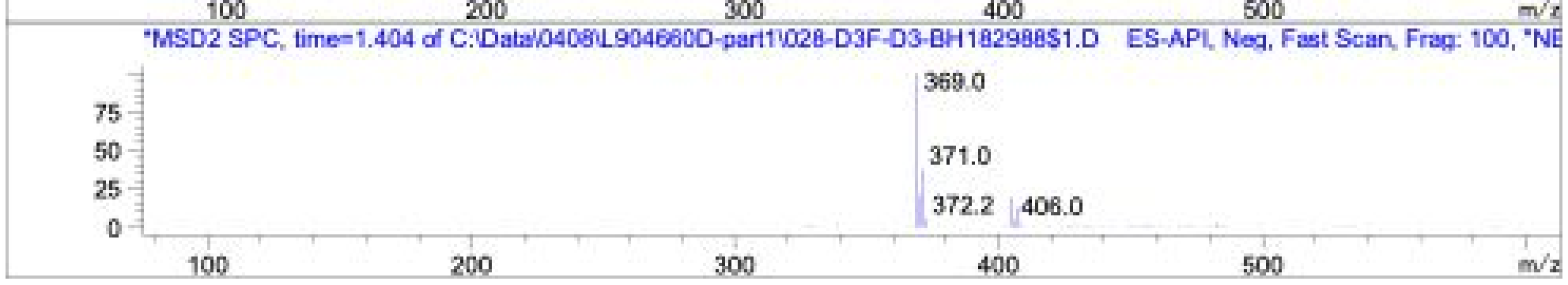

Inj.Date 4/7/2025 NEW
C:\Users\Public\Documents\ChemStation\1\Data\04_07\1904660D\SUPOR_30.M

Z2480765487

MaxPeak: 100.00%
Ret_Time: 0.835 min

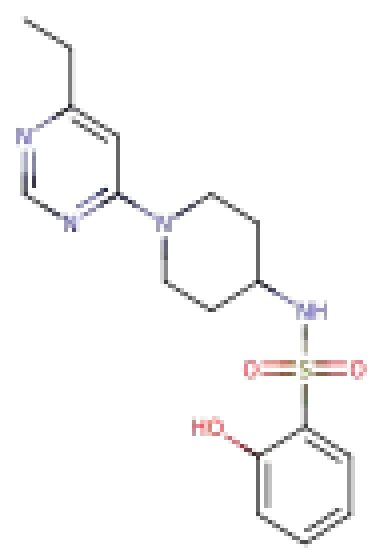

Mol Wt 362.45

Exact Mass 362.16

#	Time	Area%
1	0.835	100.00

BE557271$11

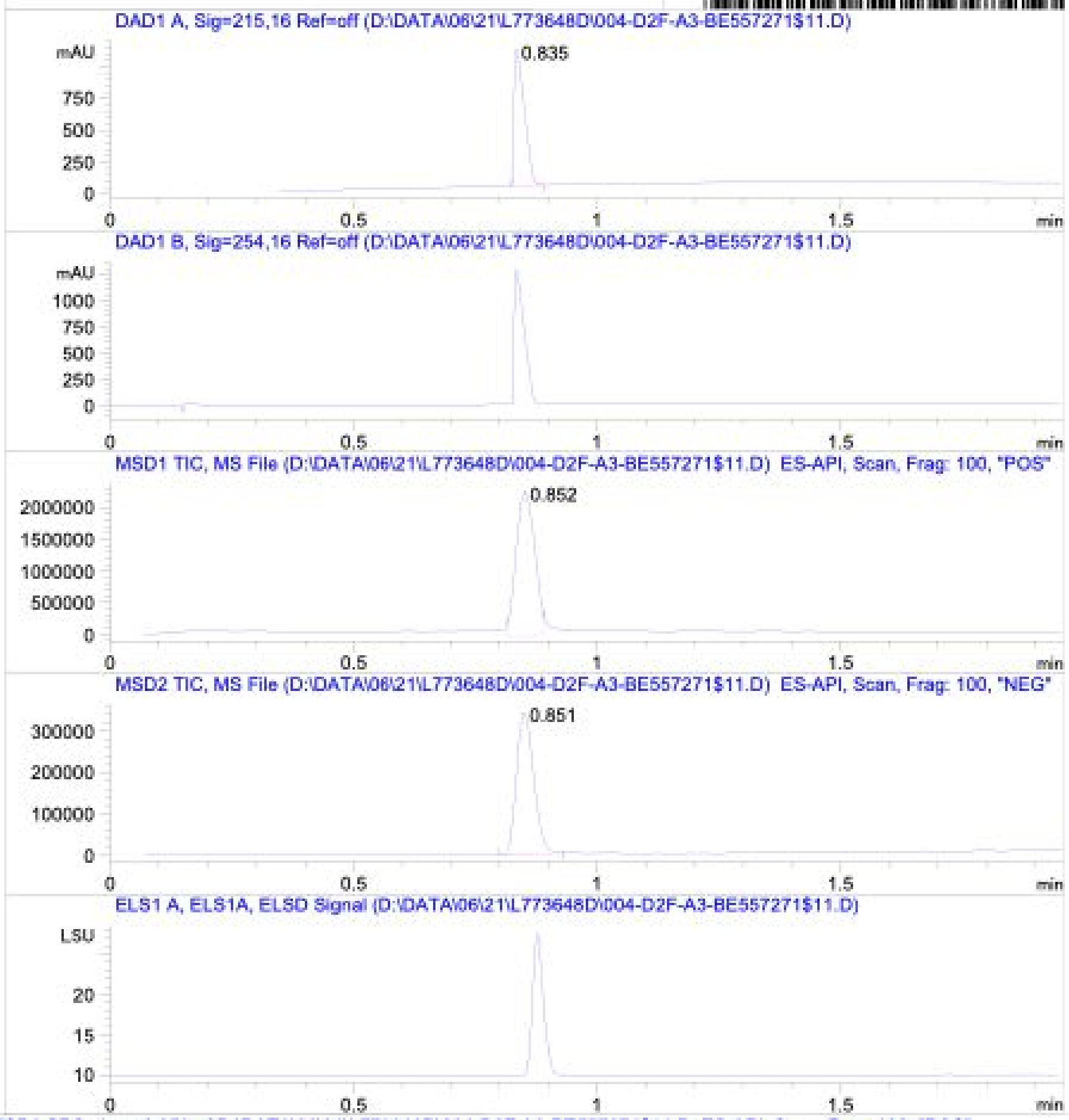

RT 0.852

RT 0.851

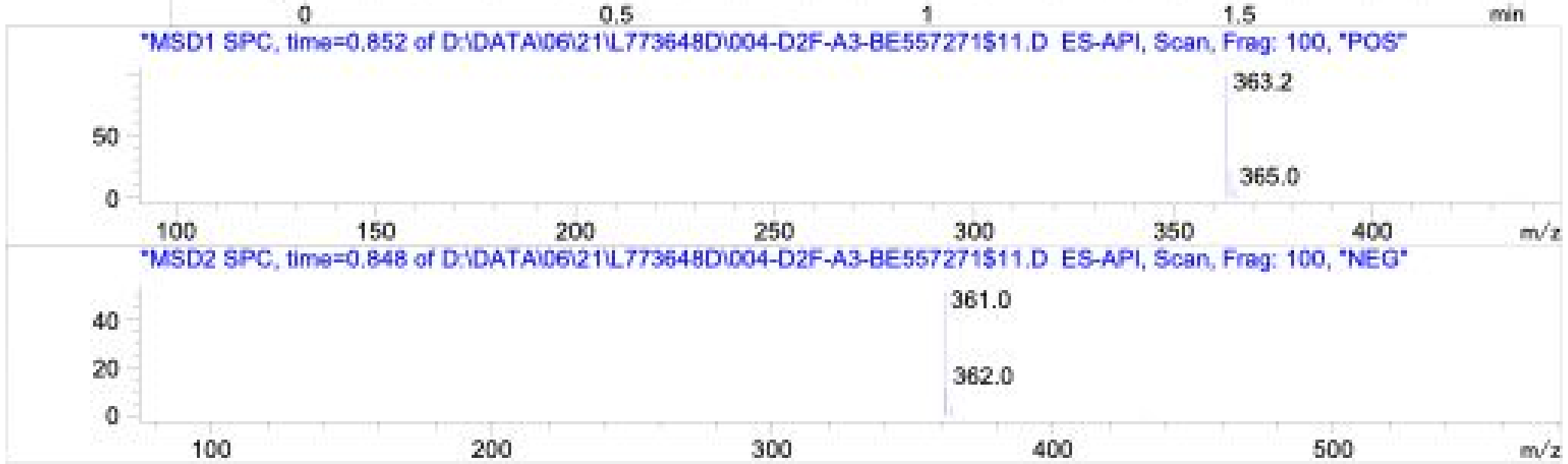

Inj.Date 6/20/2024 CH -18-
C:\Chem32\1\Data\06_20\L773648D\SUPOR_96.M

Z2579117565

MaxPeak: 100.00%
Ret_Time: 0.827 min

BG917657$32

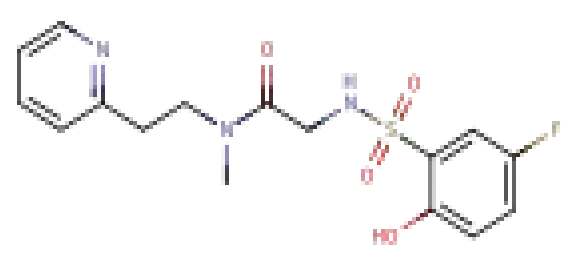

Mol Wt 367.4
Exact Mass 367.11

#	Time	Area%
1	0.827	100.00

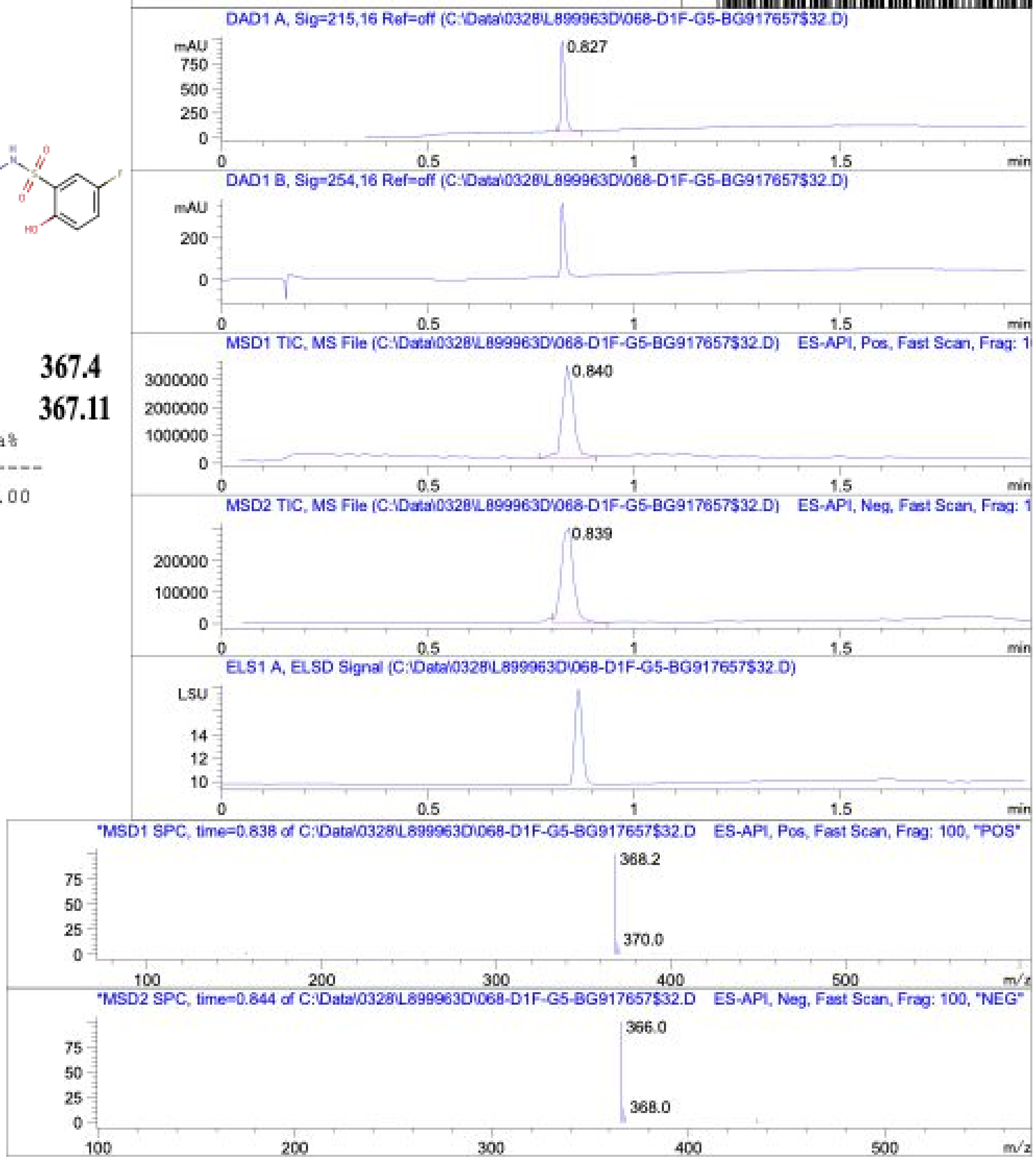

RT 0.840

RT 0.839

Inj.Date 3/27/2025 NEW
C:\Users\Public\Documents\ChemStation\1\Data\03_27\L899963D\SUPOR_96.M

Z2850291544

MaxPeak: 100.00%
Ret_Time: 1.409 min

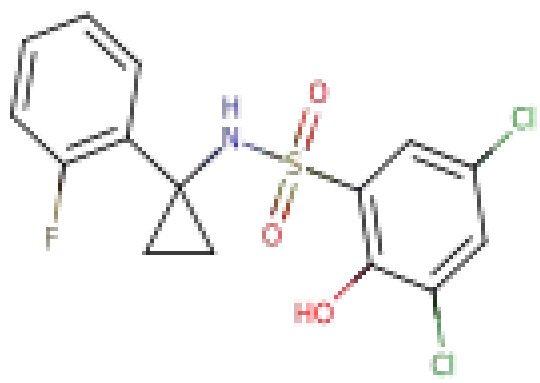

Mol Wt 376.23

Exact Mass 375

#	Time	Area%
1	1.409	100.00

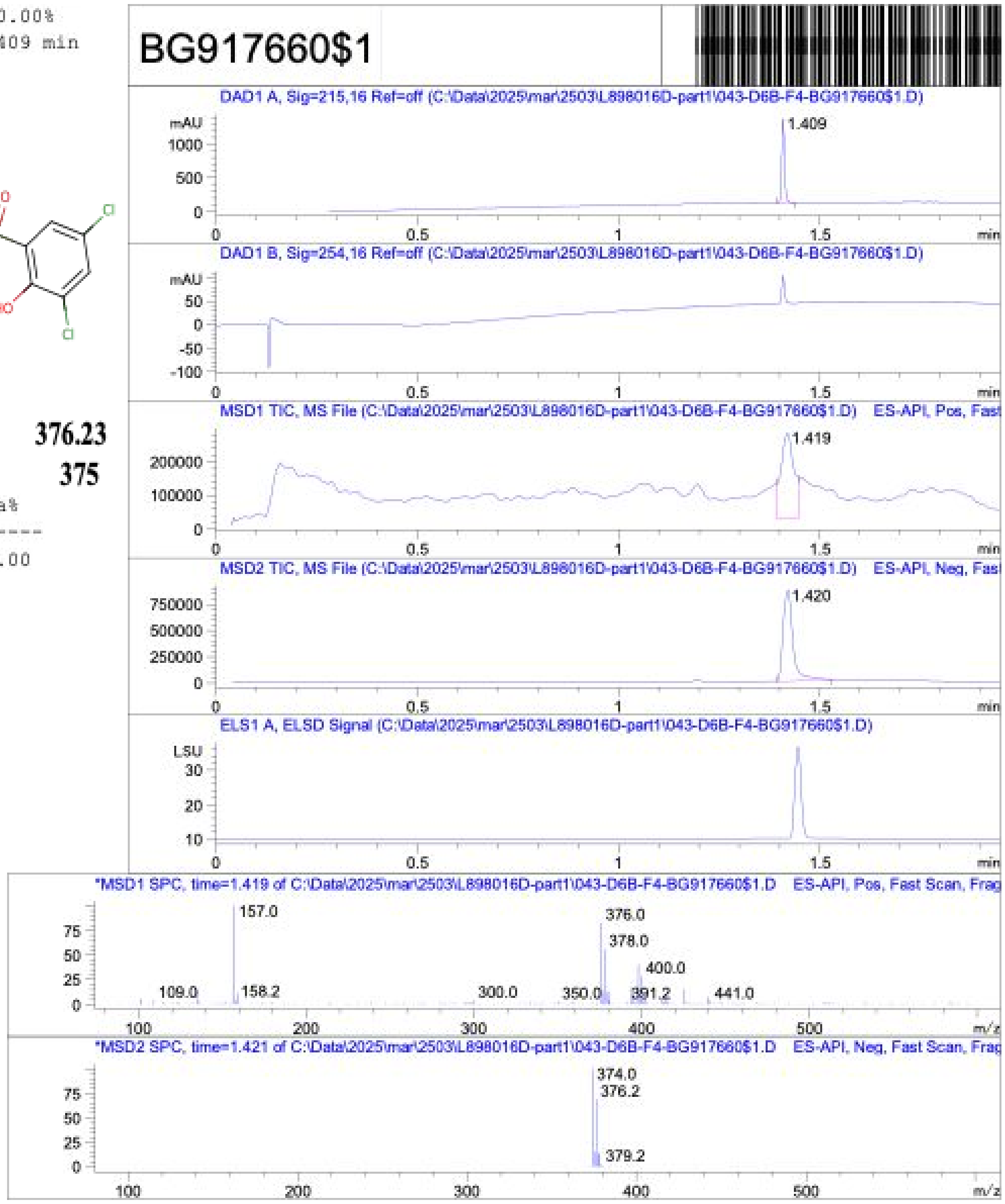

RT 1.419

RT 1.420

Inj.Date 3/24/2025 NEW
C:\Users\Public\Documents\ChemStation\1\Data\03_24\1898016D\SUPOR_30.M

Z2940313538

MaxPeak: 96.57%
Ret_Time: 1.301 min

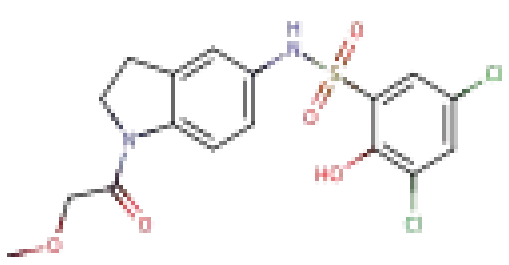

Mol Wt 431.29

Exact Mass 430.02

#	Time	Area%
1	1.003	1.12
2	1.151	2.31
3	1.301	96.57

BE244398$1

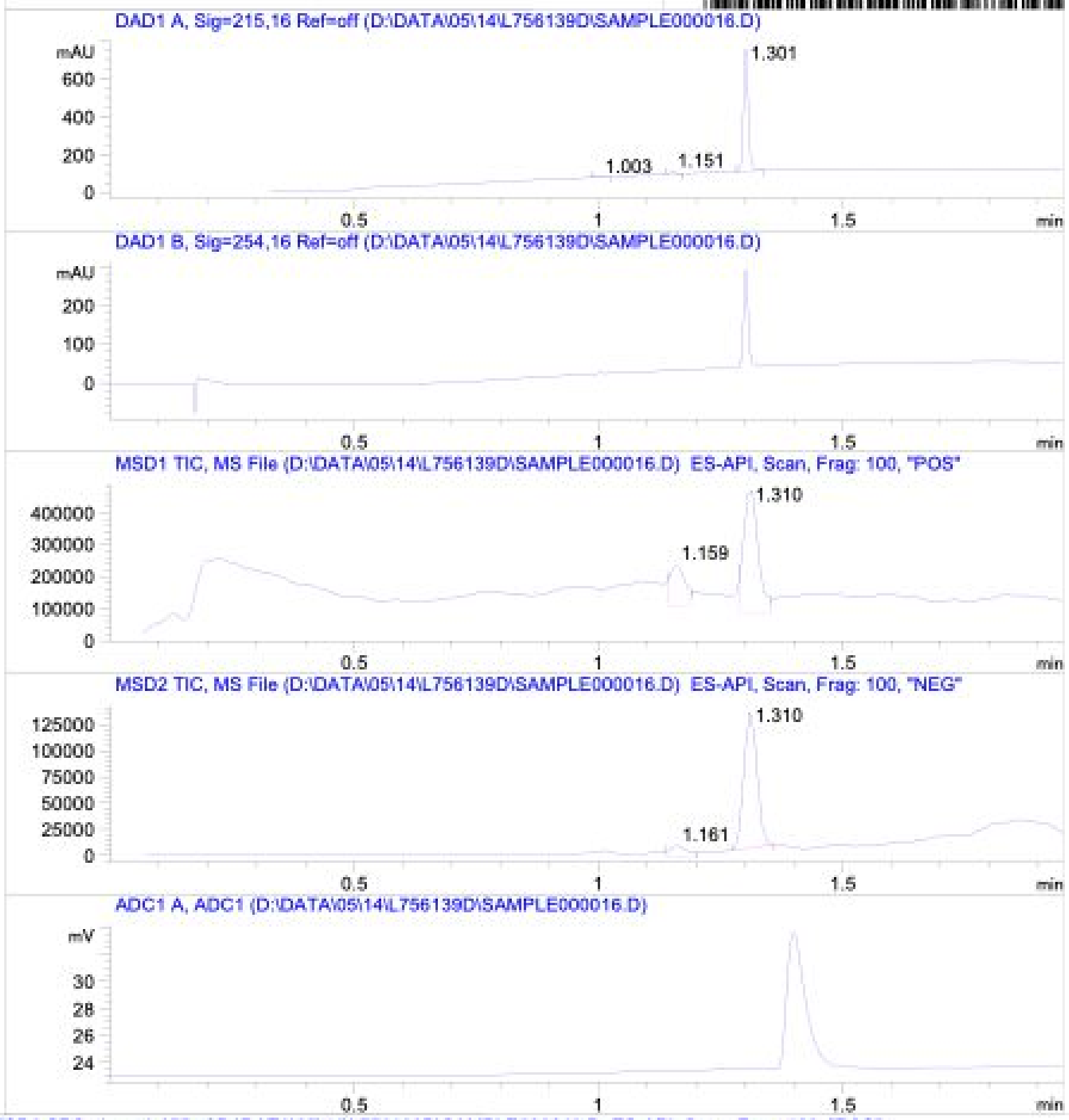

RT 1.159

RT 1.310

RT 1.161

RT 1.310

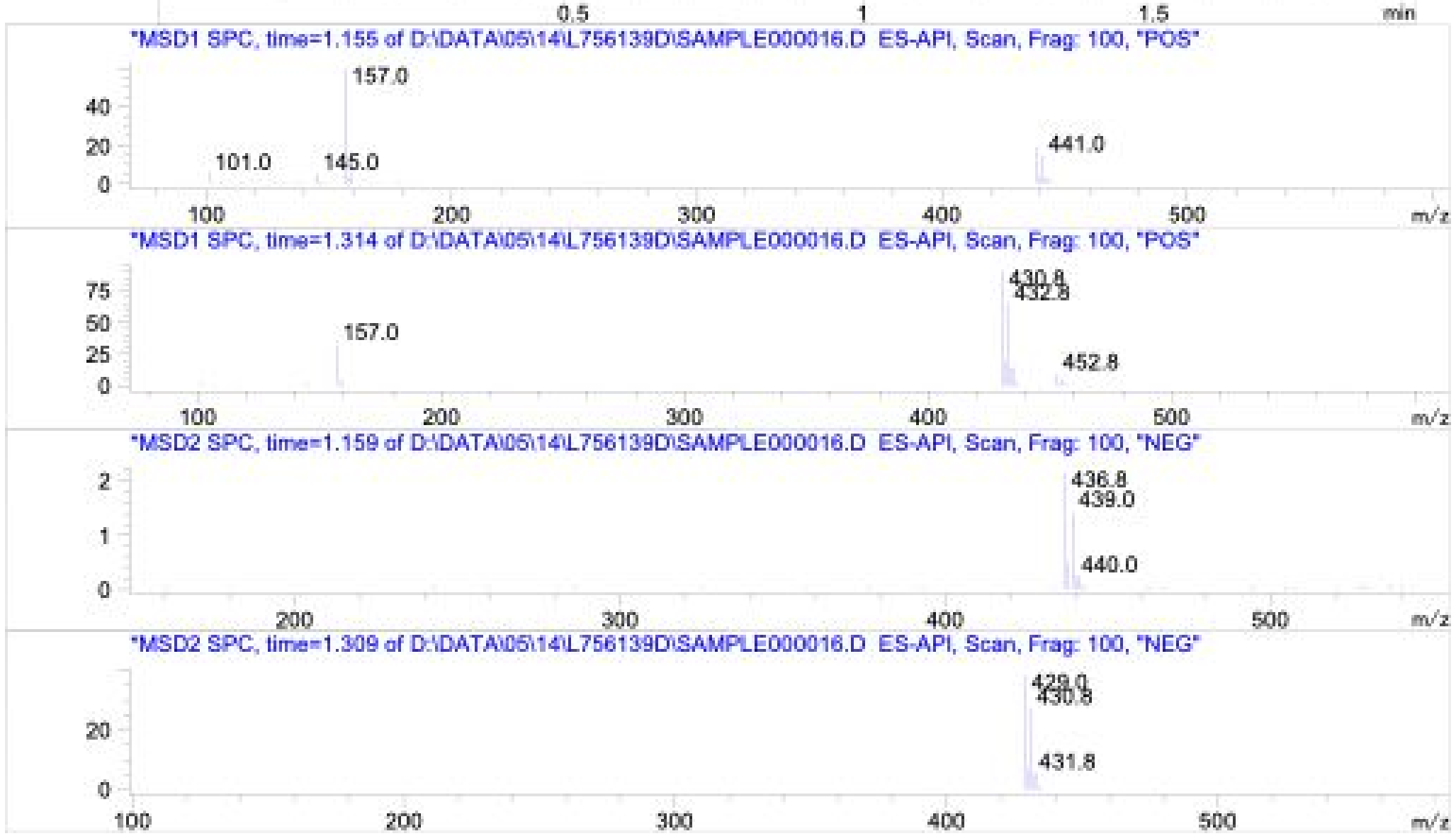

Inj.Date 5/13/2024 CH - 4 -
C:\CHEM32\1\METHODS\SUPOR_30.M

Z2940319191

MaxPeak: 94.68%
Ret_Time: 1.131 min

H5474558

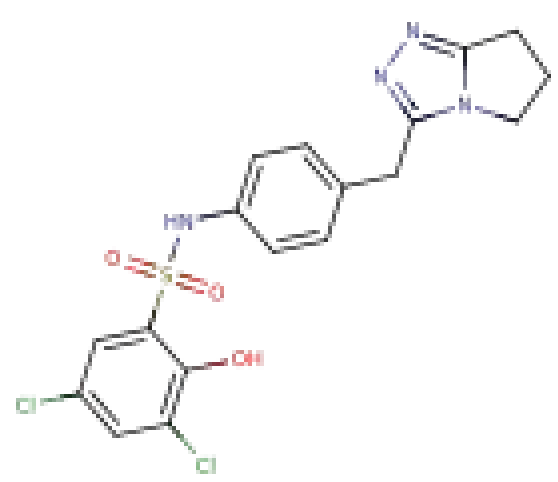

Mol Wt 439.32
Exact Mass 438.04

#	Time	Area%
1	1.076	5.32
2	1.131	94.68

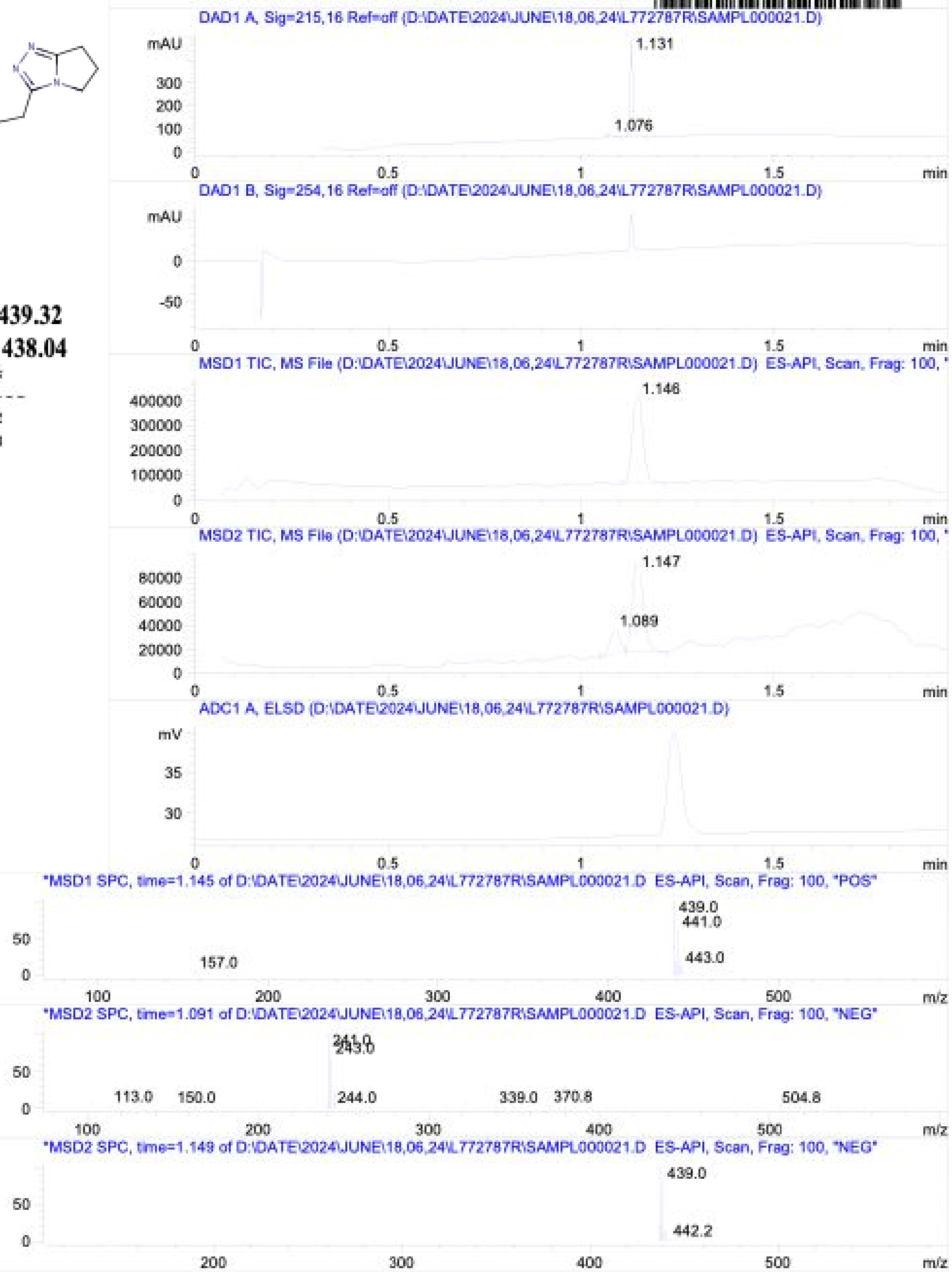

RT 1.146

RT 1.089

RT 1.147

Inj.Date 6/19/2024
C:\CHEM32\1\METHODS\SUPOR_30.M

T -6- ->

Z2940319985

MaxPeak: 97.41%
Ret_Time: 1.578 min

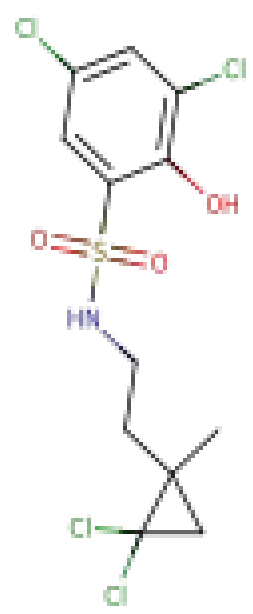

Mol Wt 393.11

Exact Mass 392.95

#	Time	Area%
1	1.034	2.59
2	1.578	97.41

BE244401$3

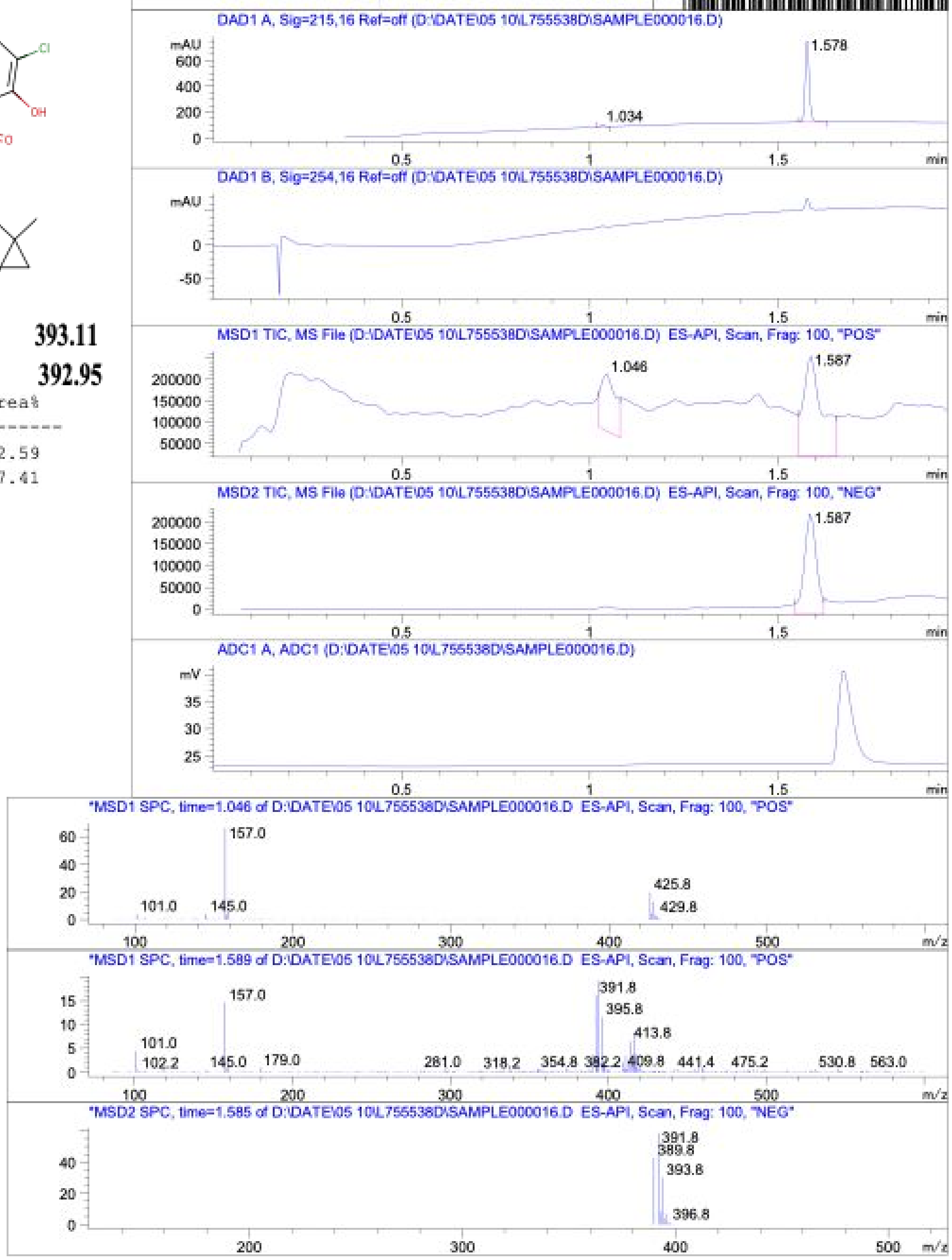

RT 1.046

RT 1.587

RT 1.587

Inj.Date 5/10/2024 E - 4 -
C:\CHEM32\1\METHODS\SUPOR_30.M

Z2940599460

MaxPeak: 100.00%
Ret_Time: 1.088 min

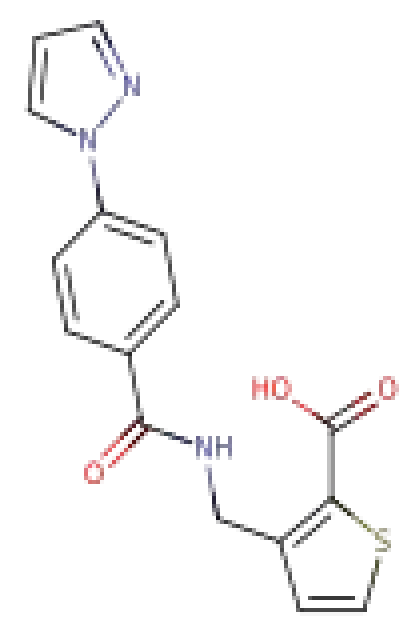

Mol Wt 327.36

Exact Mass 327.07

#	Time	Area%
1	1.088	100.00

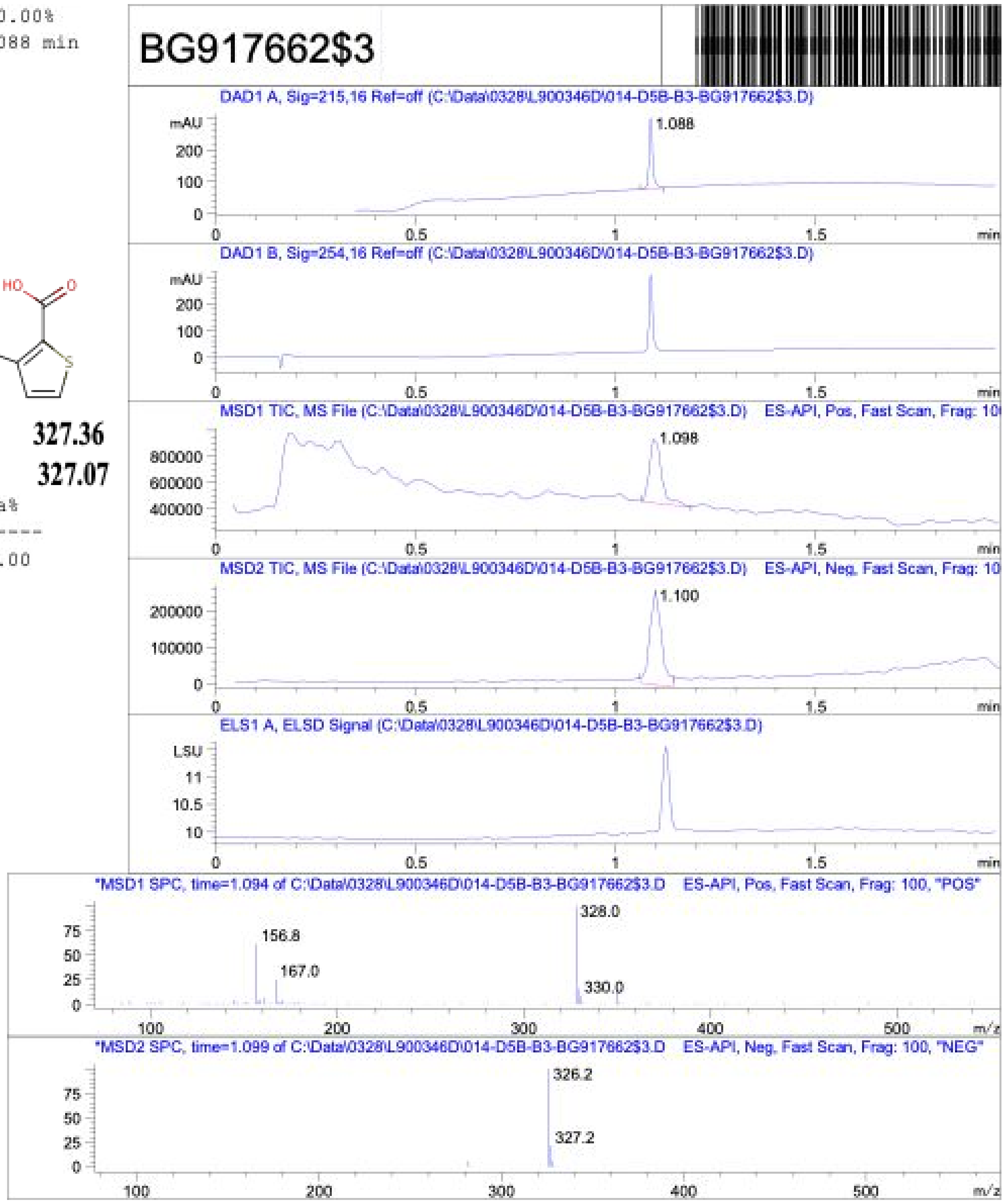

RT 1.098

RT 1.100

Inj.Date 3/27/2025 NEW
C:\Users\Public\Documents\ChemStation\1\Data\03_27\L900346D\SUPOR_30.M

Z3032896420

MaxPeak: 100.00%
Ret_Time: 1.334 min

BF054167$1

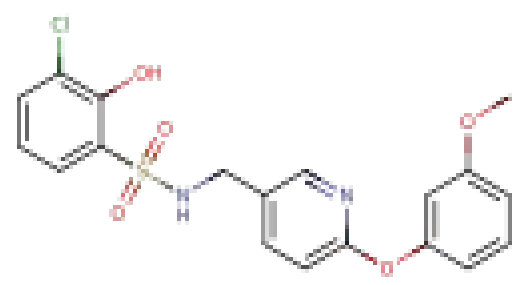

Mol Wt 420.87
Exact Mass 420.06

#	Time	Area%
1	1.334	100.00

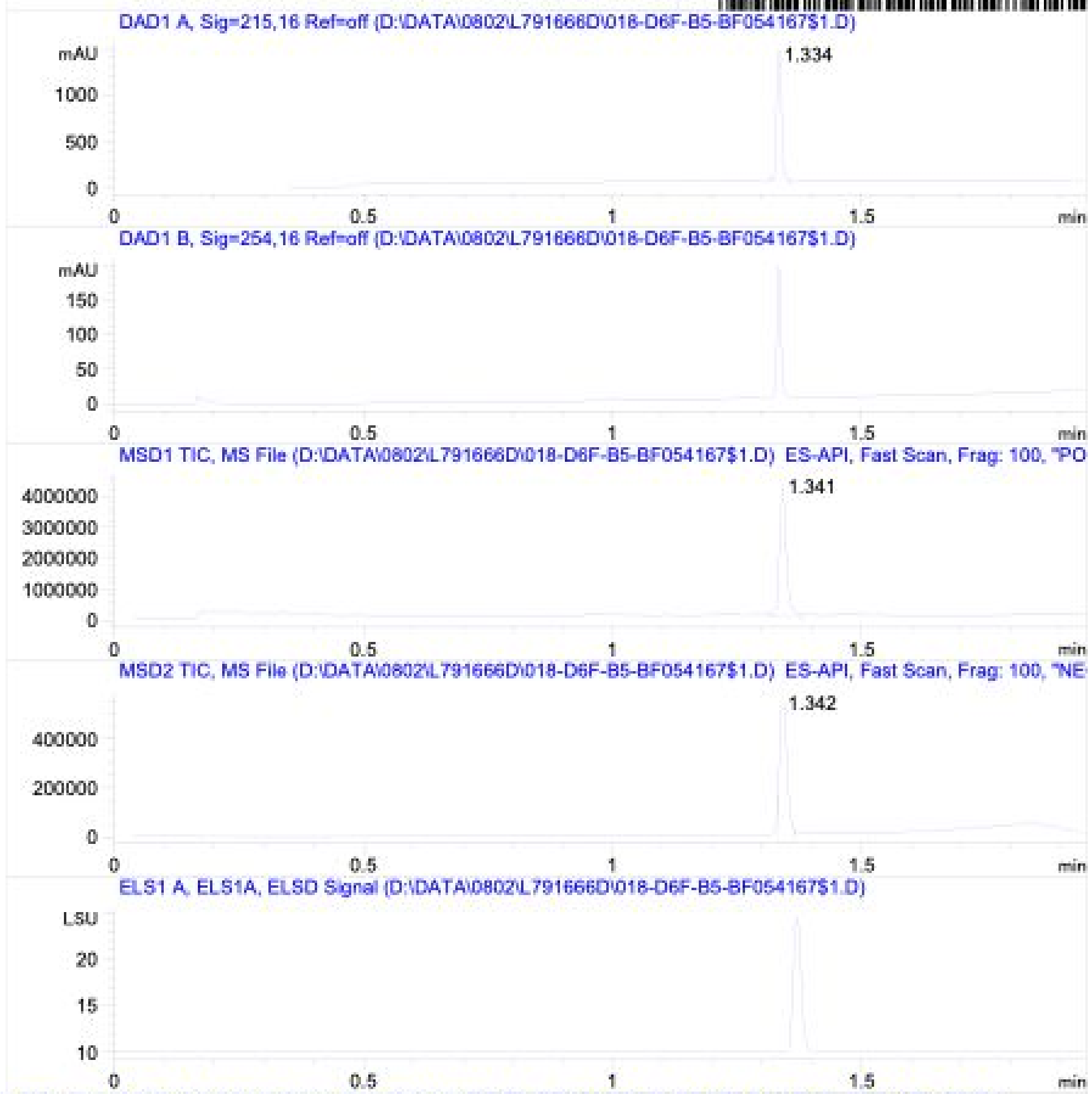

RT 1.341

RT 1.342

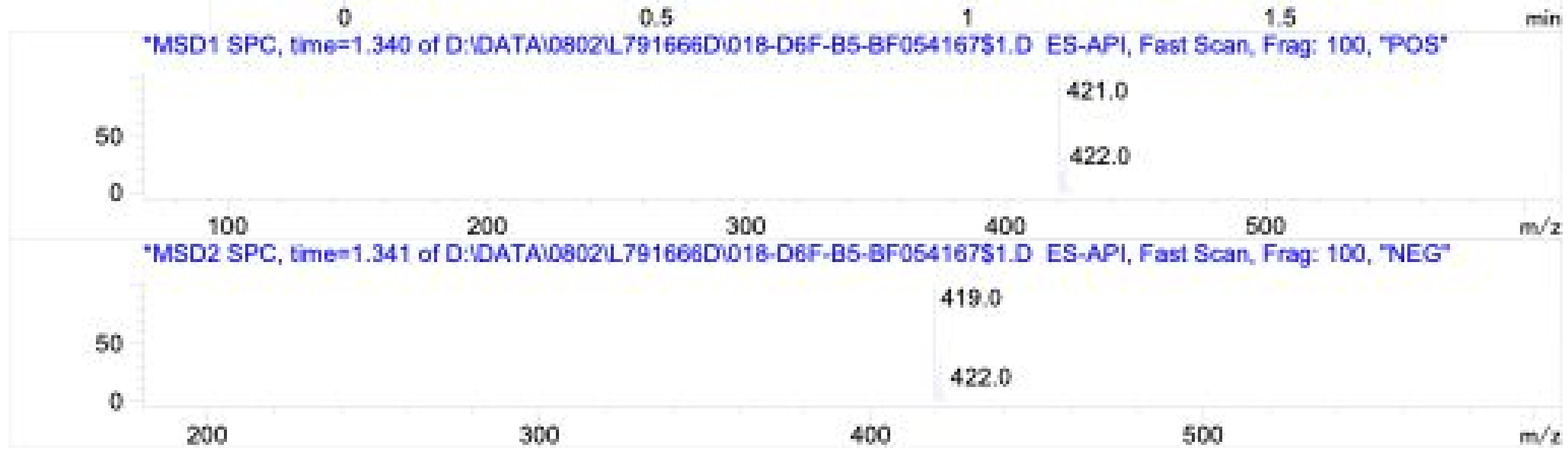

Inj.Date 8/1/2024 H
C:\Users\Public\Documents\ChemStation\1\Data\08_01\L791666D\SUPOR_30.M

Z3352582091

MaxPeak: 100.00%
Ret_Time: 1.021 min

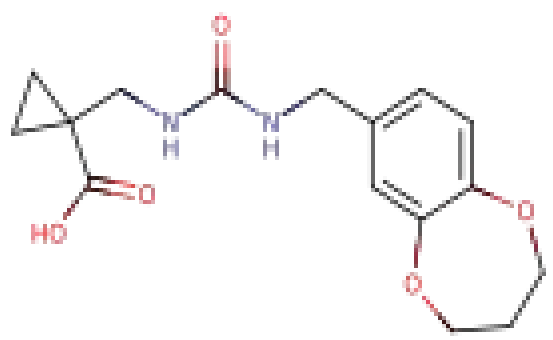

Mol Wt 320.34

Exact Mass 320.15

#	Time	Area%
1	1.021	100.00

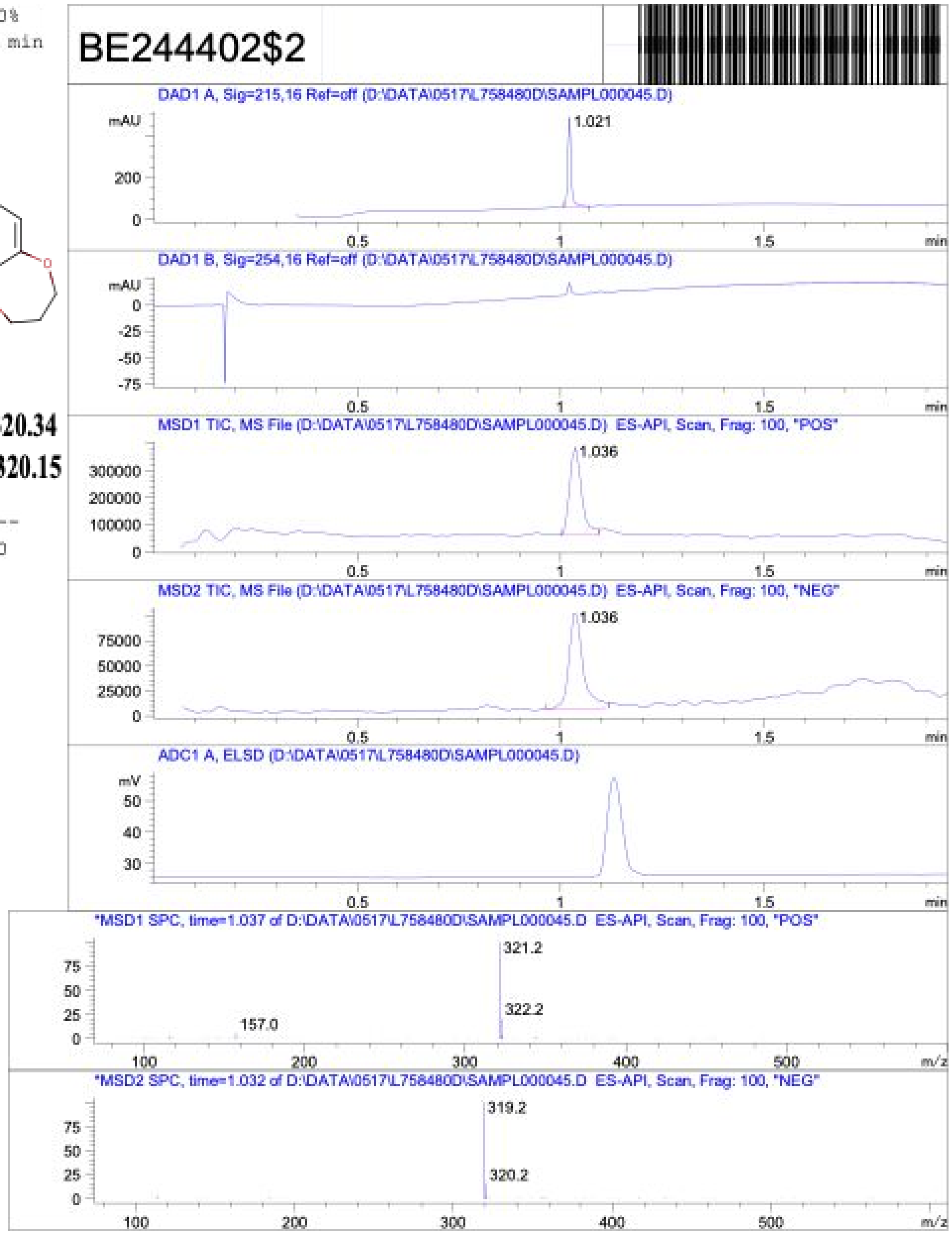

RT 1.036

RT 1.036

Inj.Date 5/17/2024 LB -6-
C:\CHEM32\1\METHODS\SUPOR_30.M

Z3510768208

MaxPeak: 100.00%
Ret_Time: 1.256 min

BG918252$1

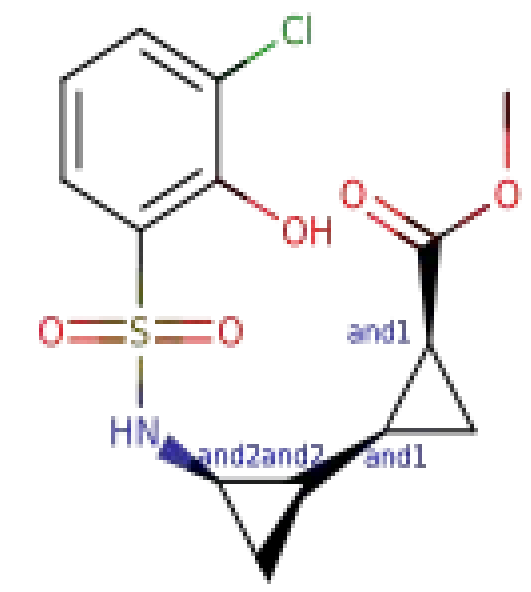

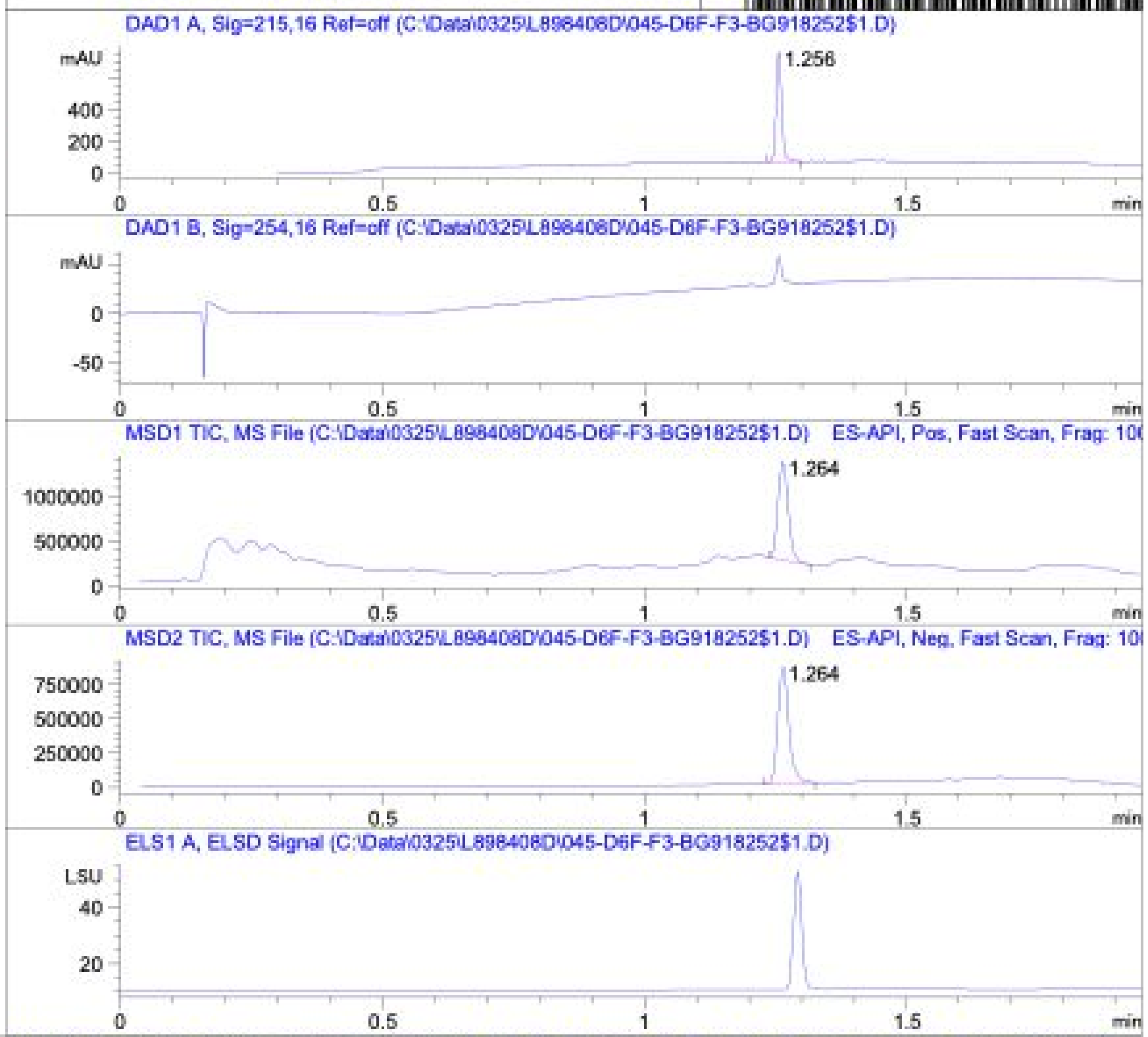

Mol Wt 345.8

Exact Mass 345.05

#	Time	Area%
1	1.256	100.00

RT 1.264

RT 1.264

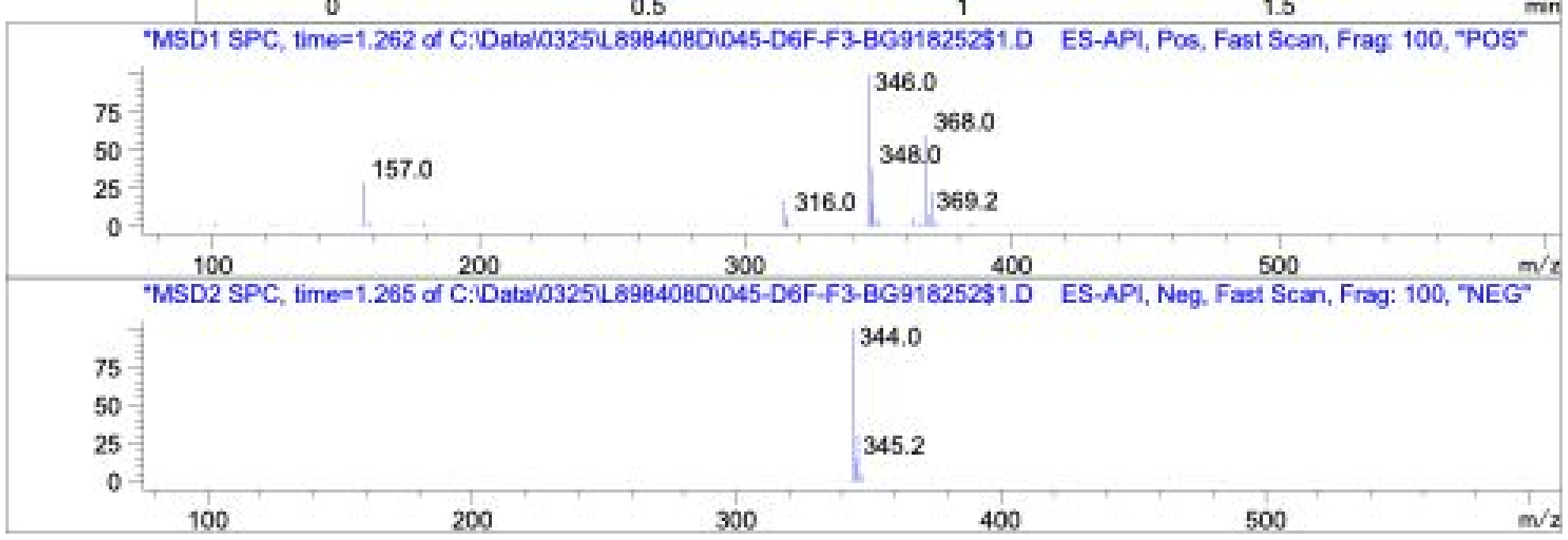

Inj.Date 3/24/2025 NEW -29-
C:\Users\Public\Documents\ChemStation\1\Data\03_24\L898408D\SUPOR_30.M

Z4005054825

MaxPeak: 100.00%
Ret_Time: 1.014 min

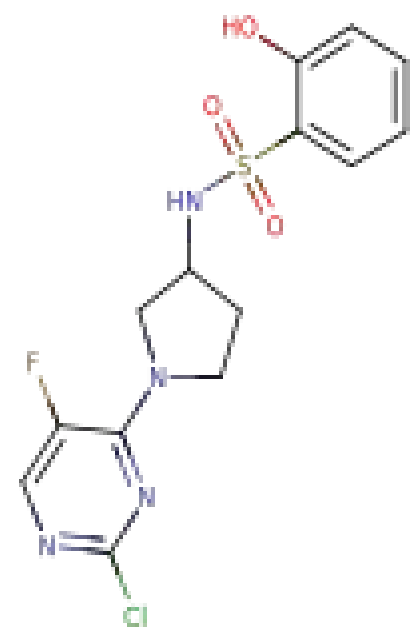

Mol Wt 372.8

Exact Mass 372.05

#	Time	Area%
1	1.014	100.00

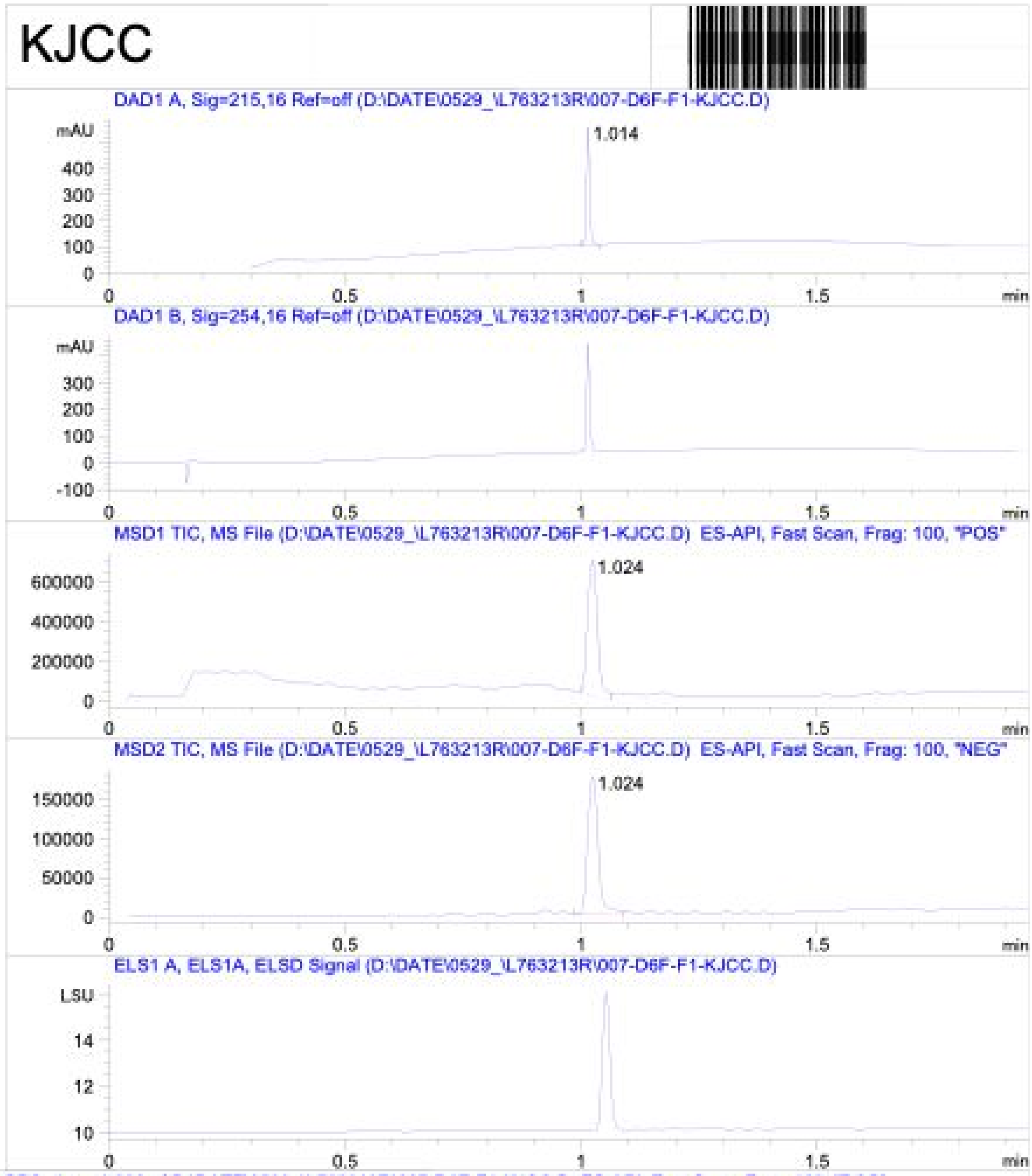

RT 1.024

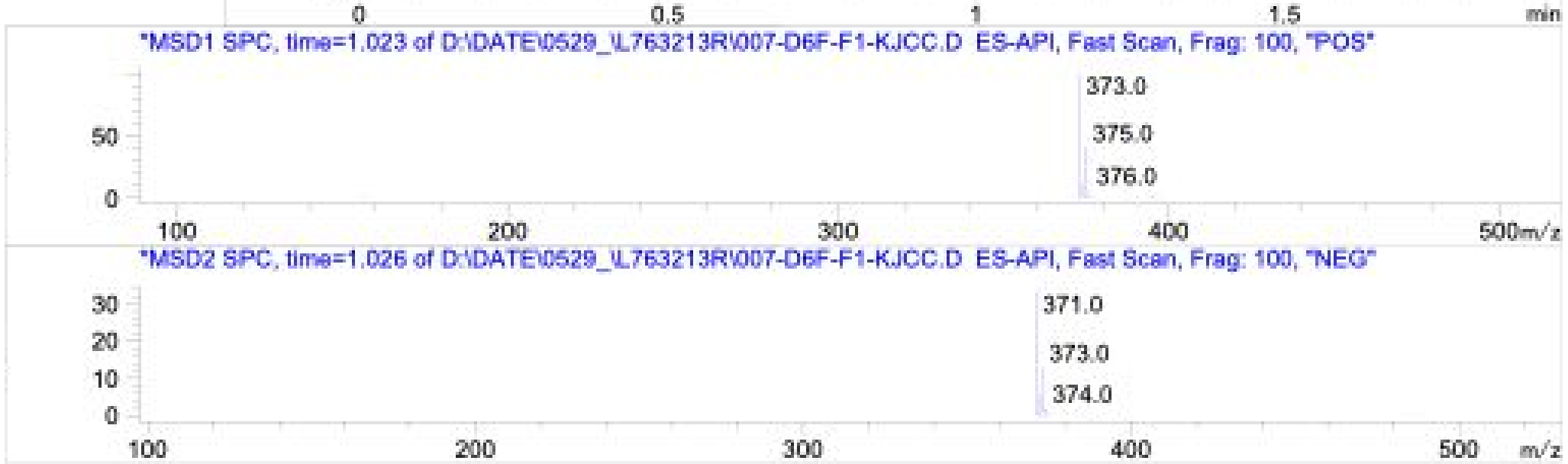

RT 1.024

Inj.Date 5/29/2024 O 30
C:\Users\Public\Documents\ChemStation\1\Data\05_29\L763213R\SUPOR_30.M

Z4278888865

MaxPeak: 100.00%
Ret_Time: 0.658 min

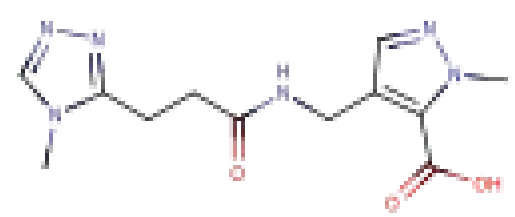

Y872498$1

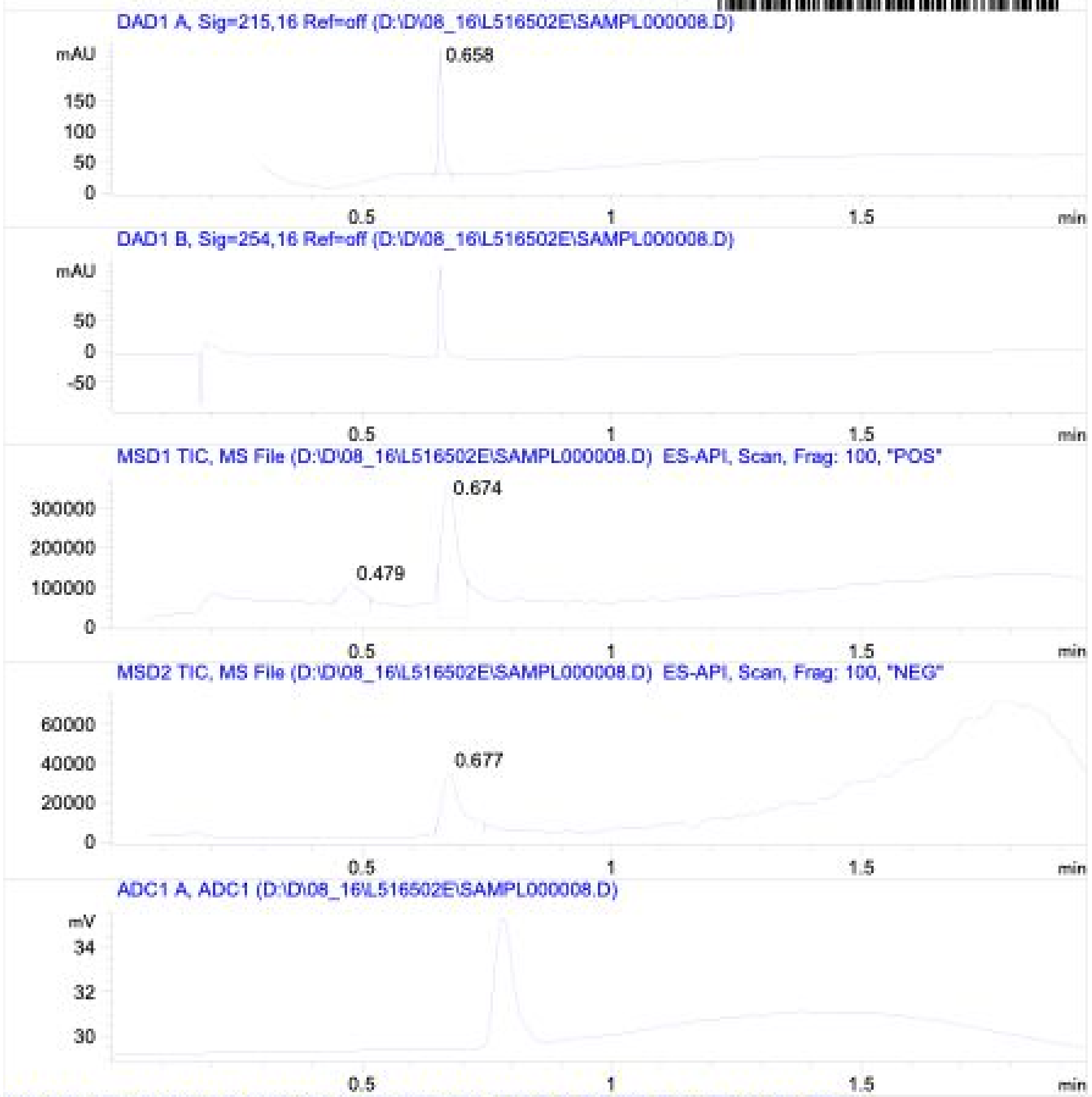

Mol Wt 292.29
Exact Mass 292.13

#	Time	Area%
1	0.658	100.00

RT 0.479

RT 0.674

RT 0.677

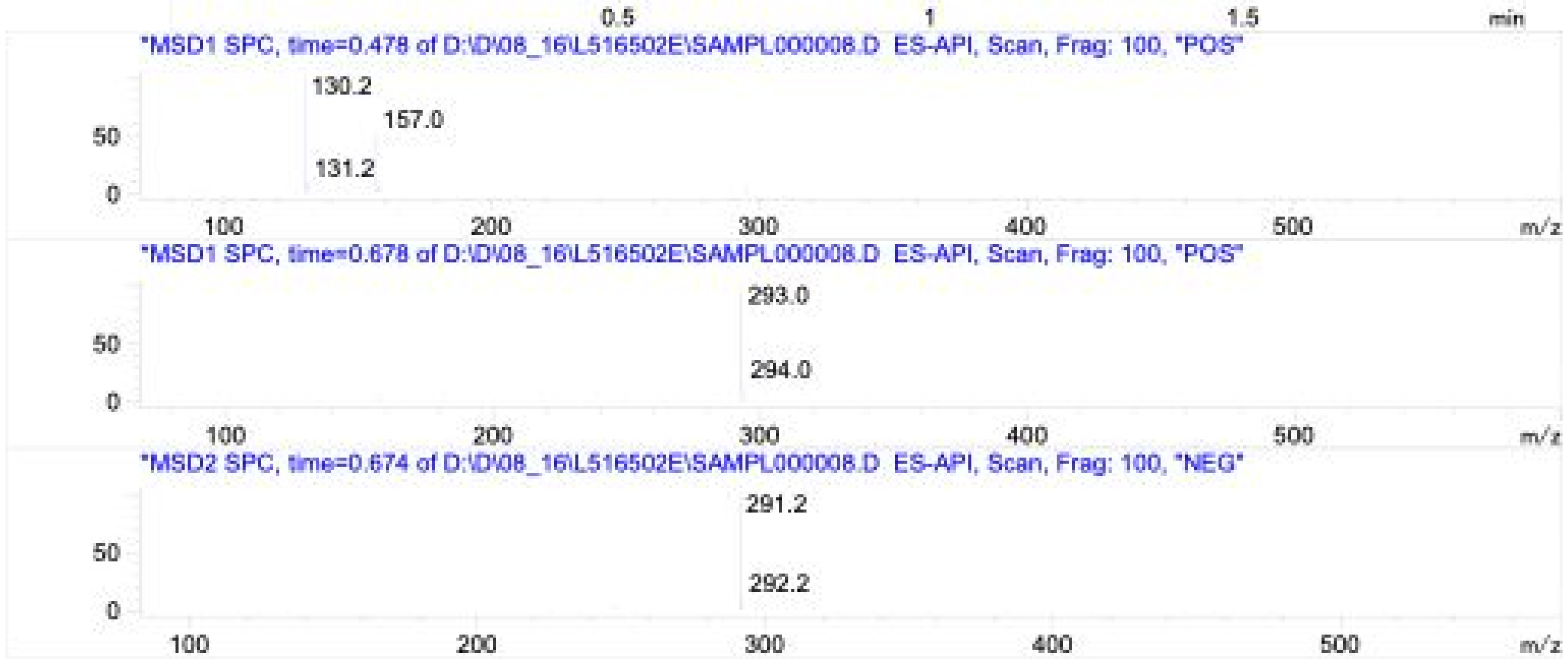

Inj.Date 8/16/2022 N - 4 - Acq. Method C:\CHEM32\-> ->

Z6615014431

MaxPeak: 100.00%
Ret_Time: 1.175 min

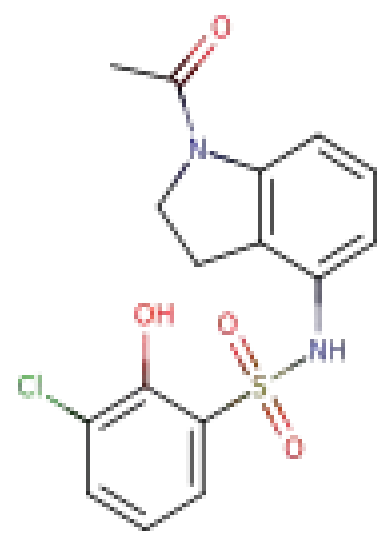

Mol Wt 366.82
Exact Mass 366.05

#	Time	Area%
1	1.175	100.00

Y872800$2

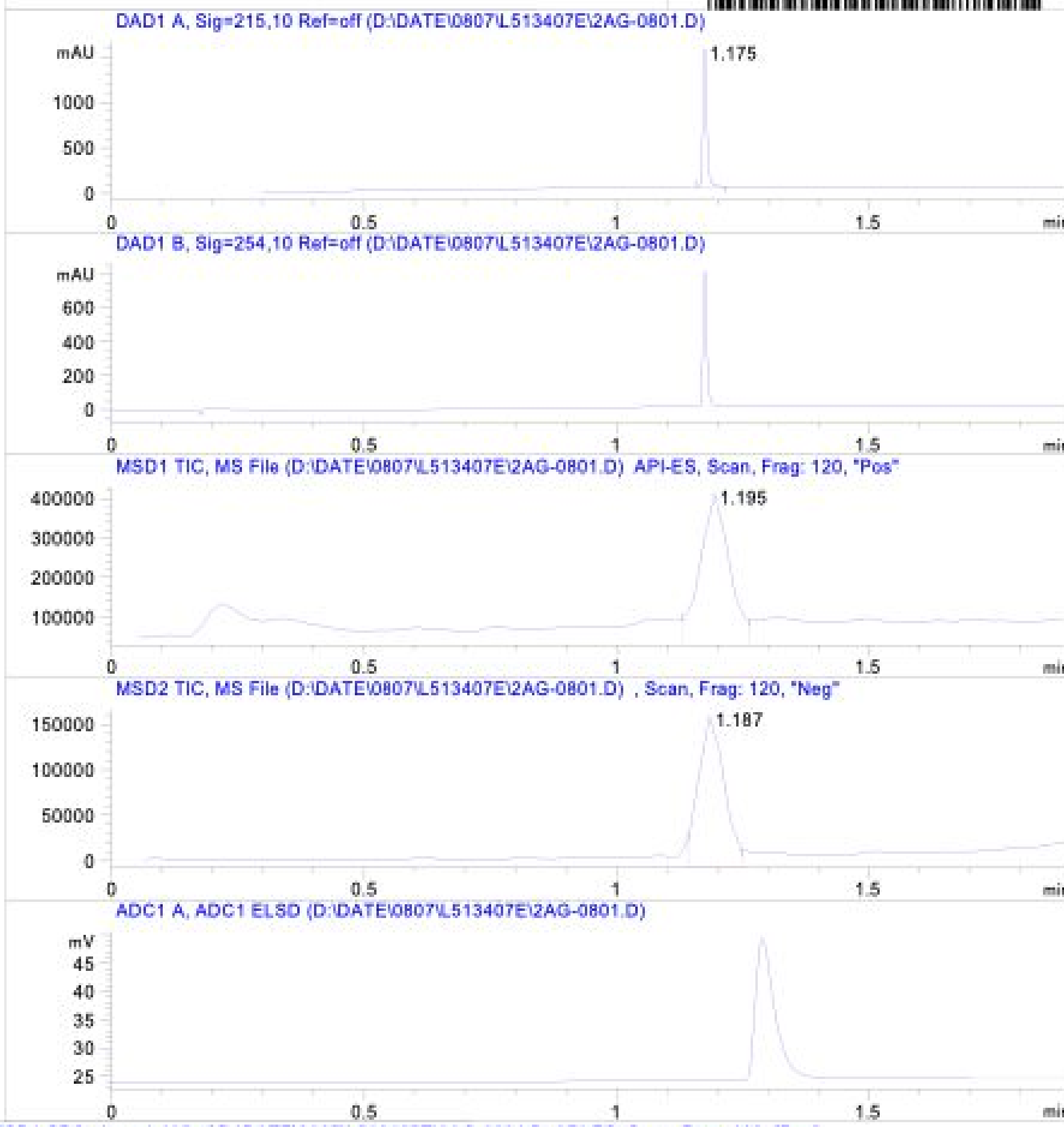

RT 1.195

RT 1.187

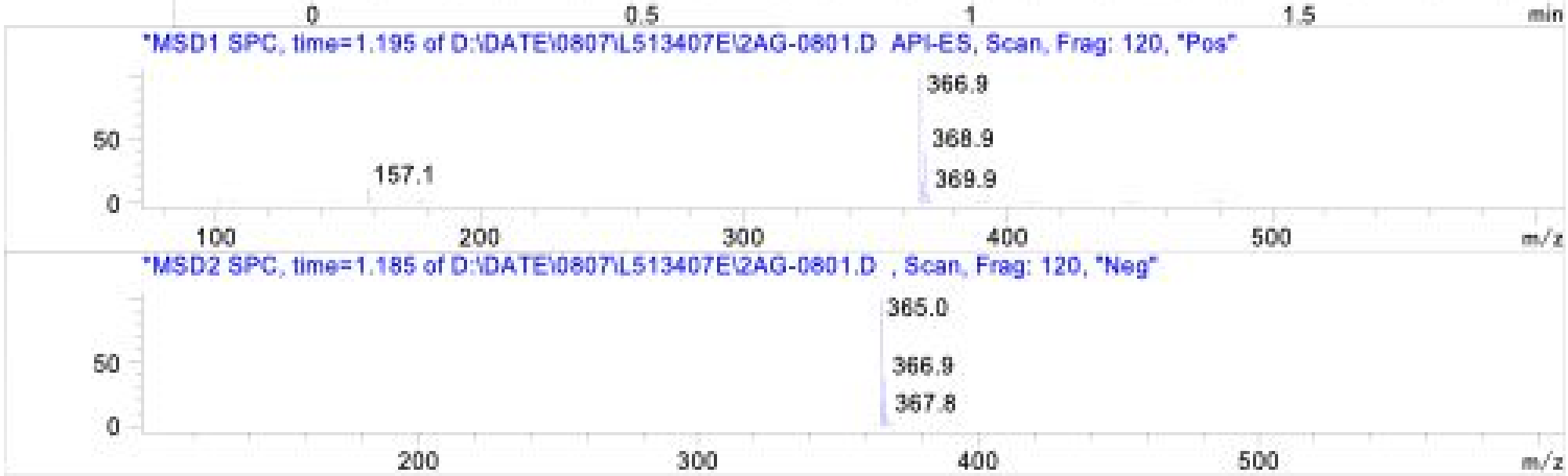

Inj.Date 8/5/2022 E -VL- Acq. Method C:\HPCHEM\-> ->

Z6615017782

MaxPeak: 95.36%
Ret_Time: 1.333 min

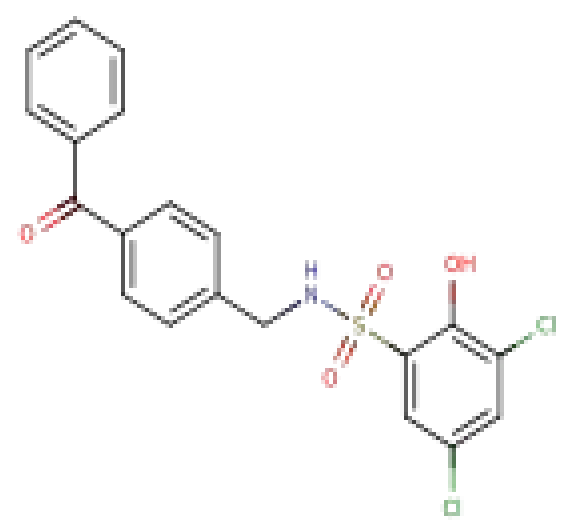

Mol Wt 436.31

Exact Mass 435.02

#	Time	Area%
1	1.296	2.89
2	1.333	95.36
3	1.398	1.75

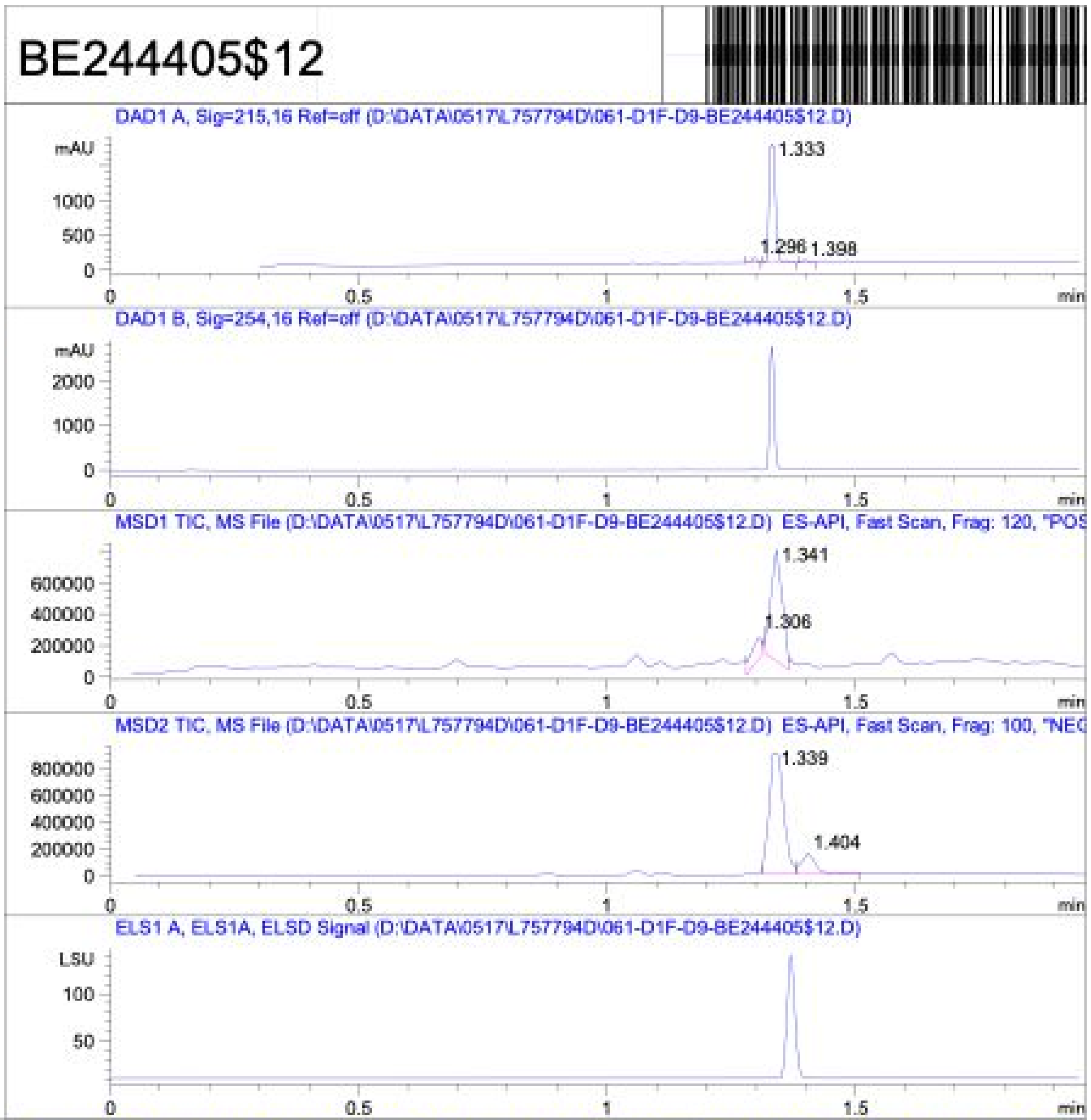

RT 1.306

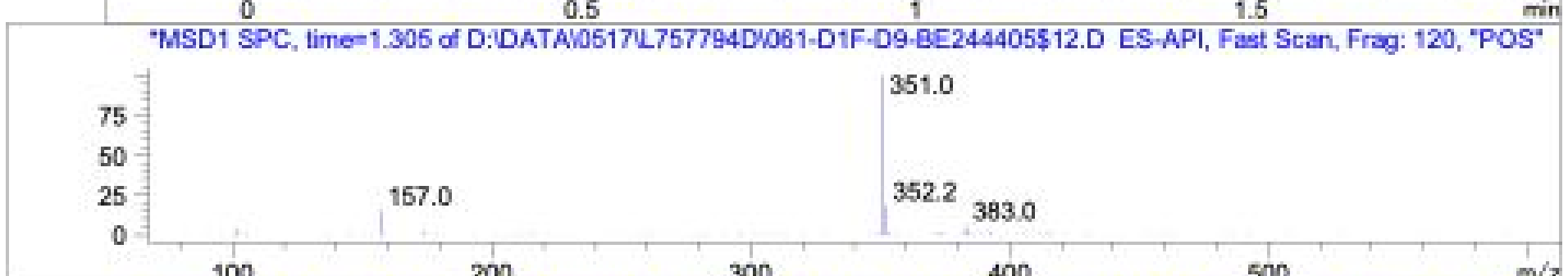

RT 1.341

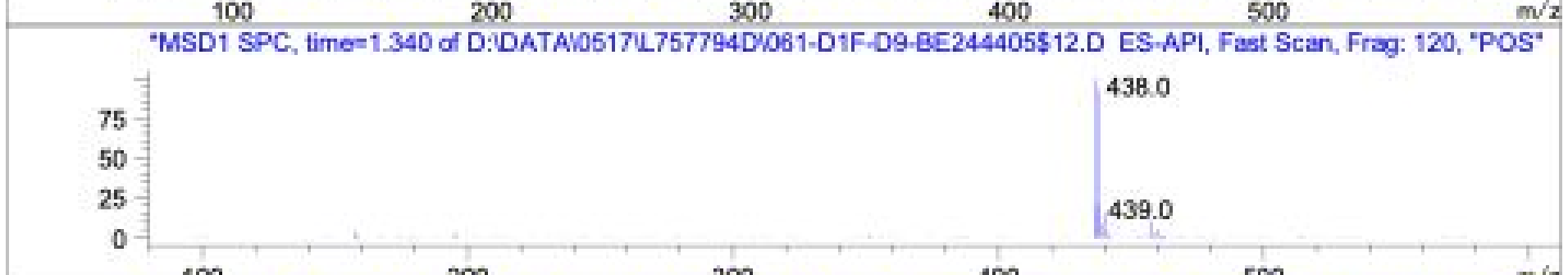

RT 1.339

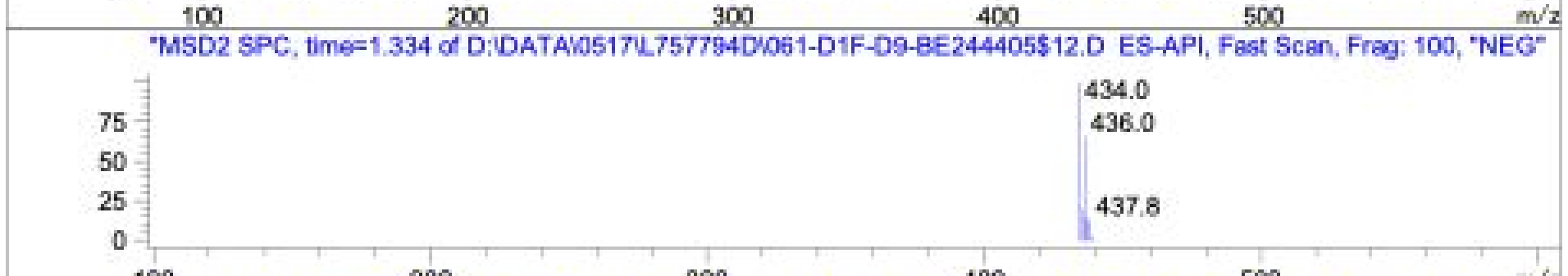

RT 1.404

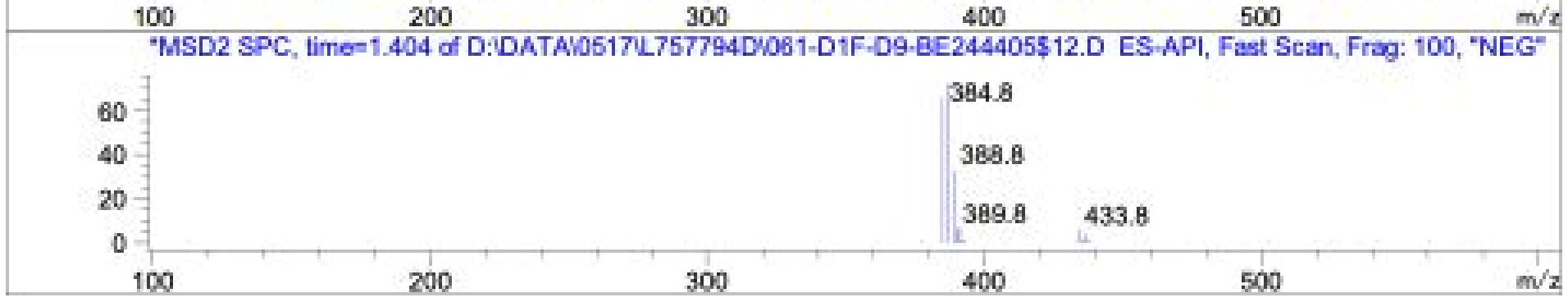

Inj.Date 5/16/2024 LB
C:\Users\Public\Documents\ChemStation\1\Data\05_16\L757794D\SUPOR_96.M

Z7889182816

MaxPeak: 100.00%
Ret_Time: 1.267 min

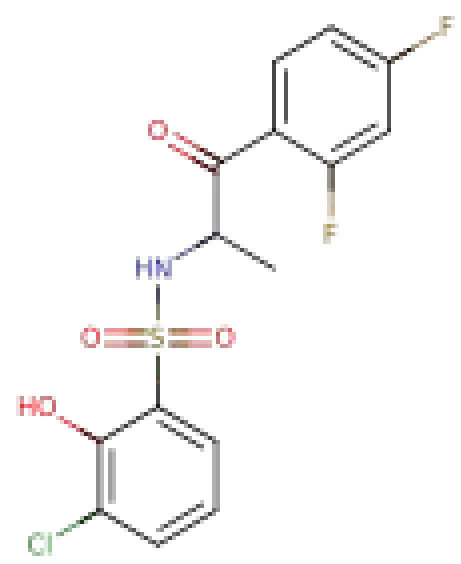

Mol Wt 375.78
Exact Mass 375.02

#	Time	Area%
1	1.267	100.00

BA658503$2

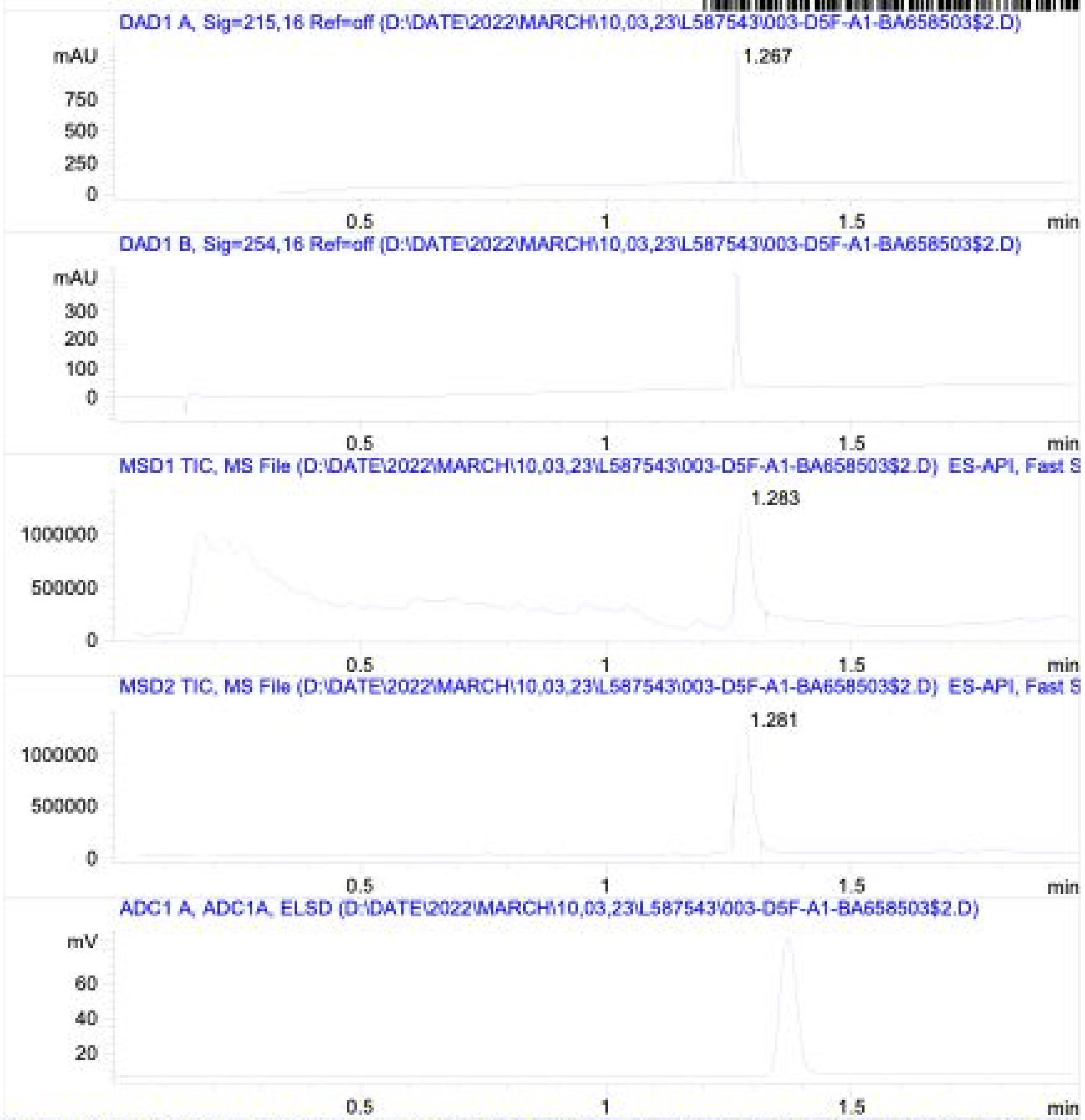

RT 1.283

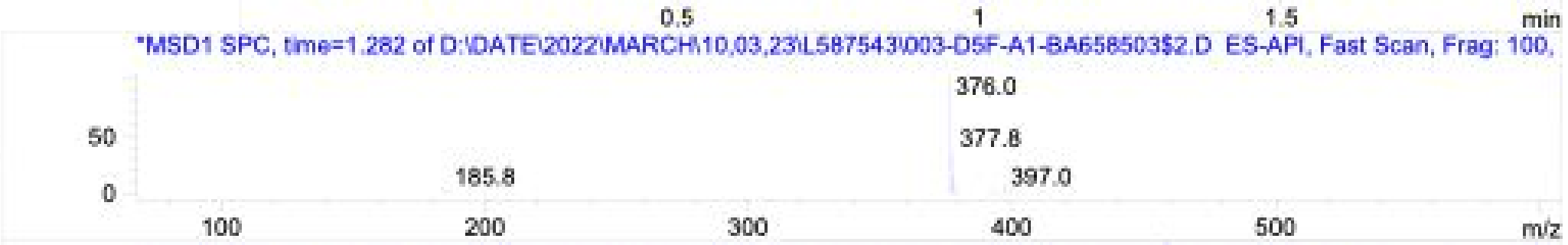

RT 1.281

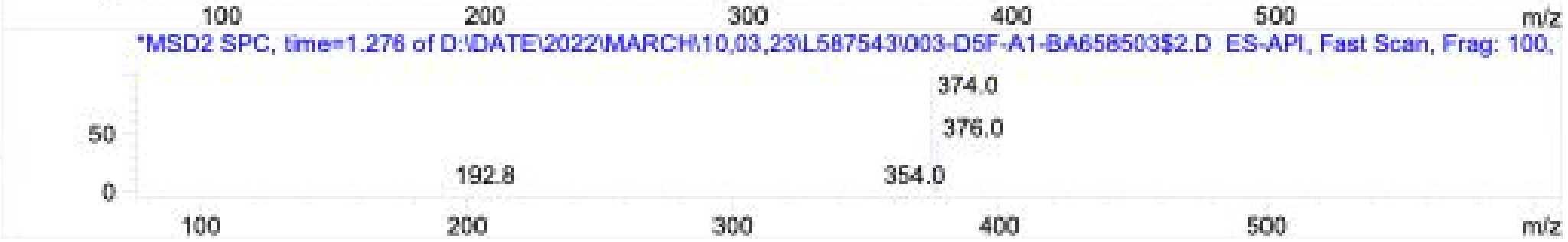

Inj.Date 3/9/2023 T <invalid> -3- Acq. Method C:\Users\ -> ->

Z8124313298

MaxPeak: 97.41%
Ret_Time: 0.709 min

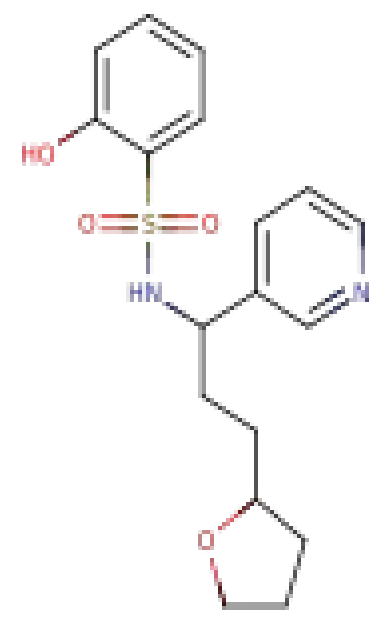

Mol Wt 362.44

Exact Mass 362.15

#	Time	Area%
1	0.709	97.41
2	1.324	2.59

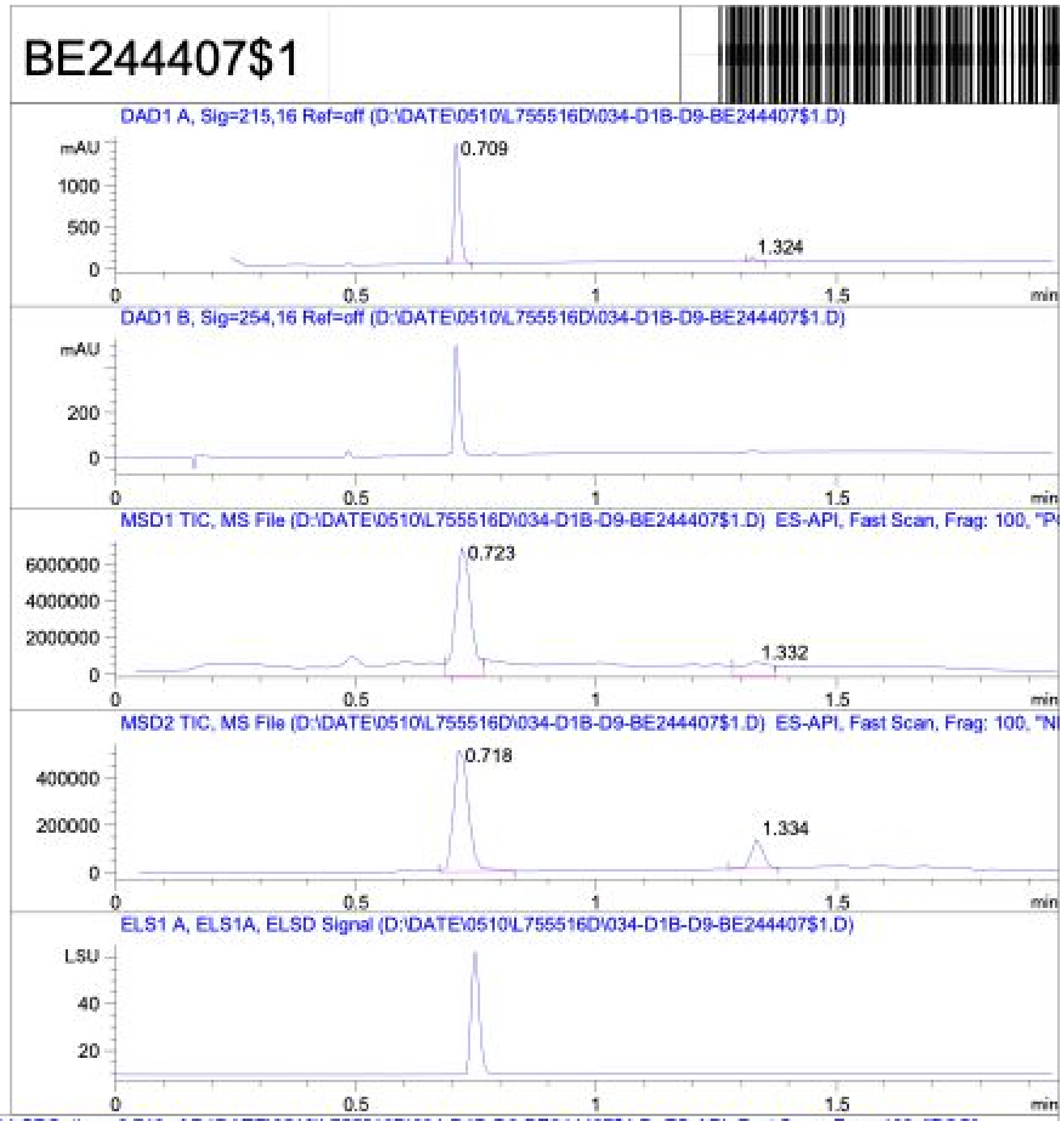

RT 0.723

RT 1.332

RT 0.718

RT 1.334

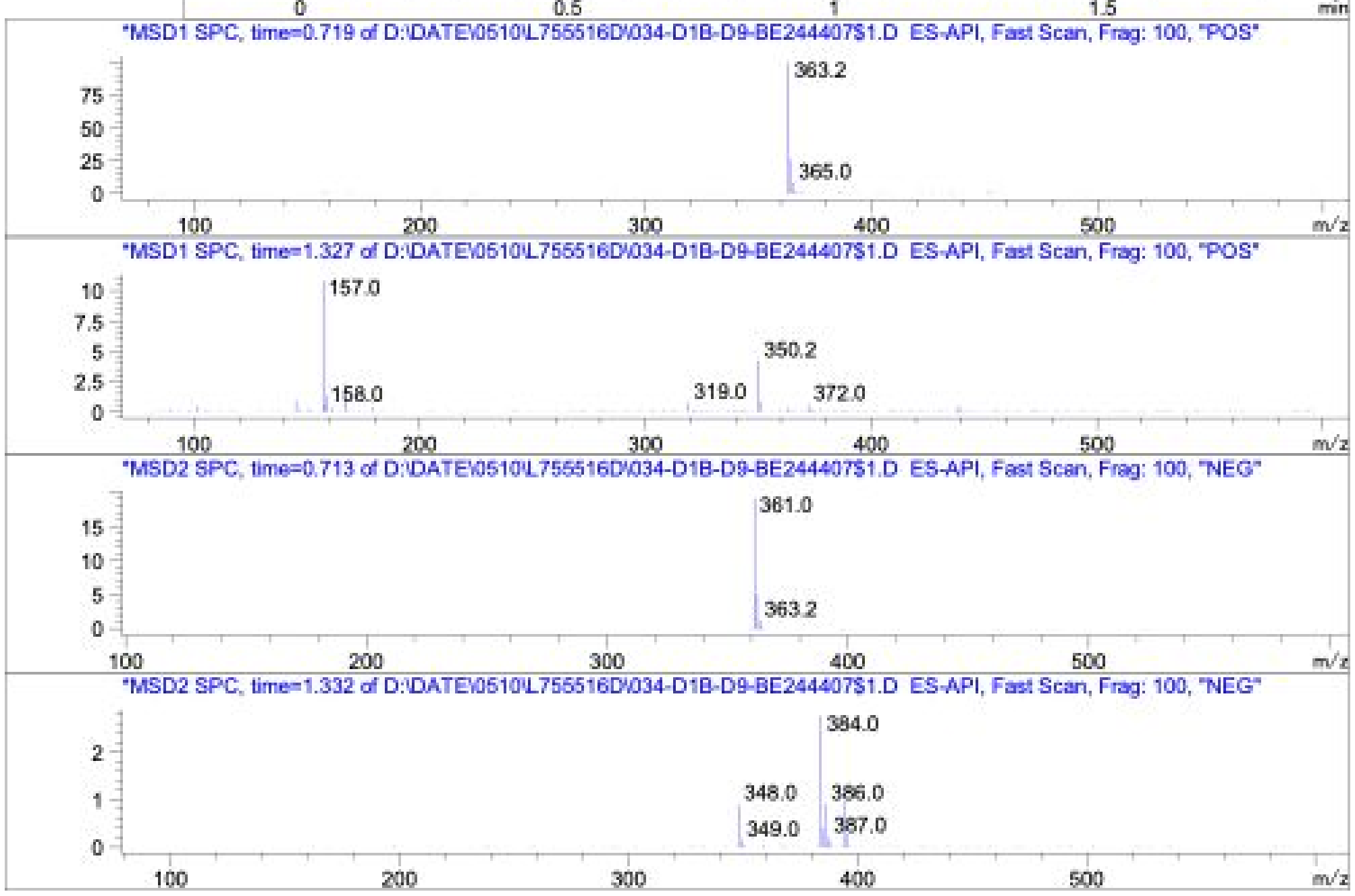

Inj.Date 5/10/2024 E 20
C:\Users\Public\Documents\ChemStation\1\Data\05_10\L755516D\SUPOR_96.M

Z8174370523

MaxPeak: 100.00%
Ret_Time: 0.869 min

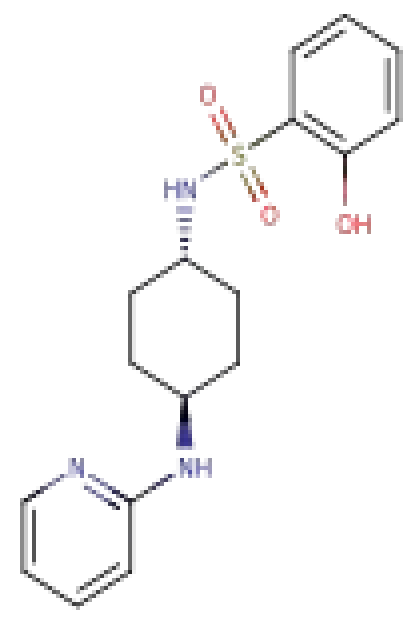

Mol Wt 347.43

Exact Mass 347.15

#	Time	Area%
1	0.869	100.00

BE557588$33

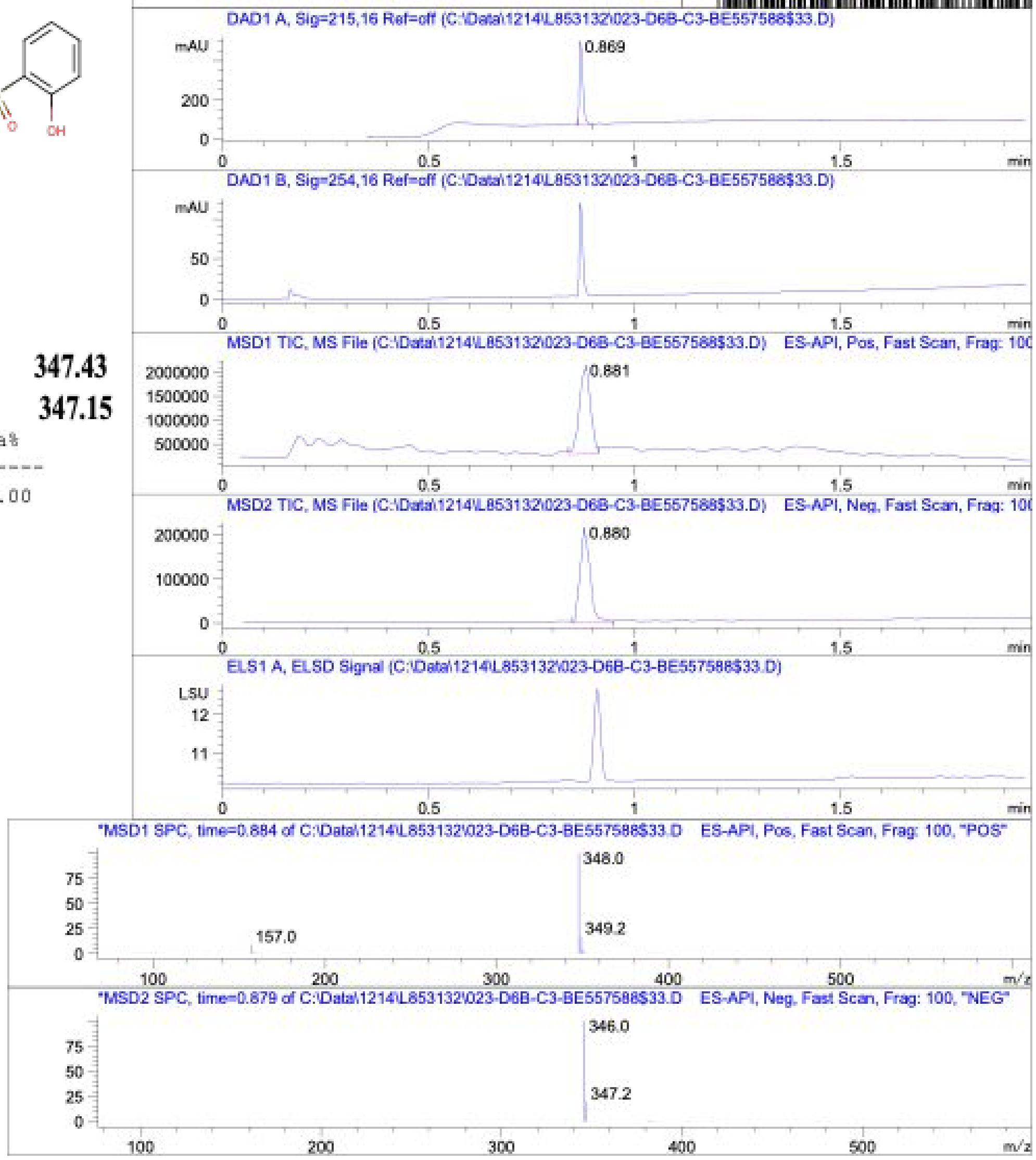

RT 0.881

RT 0.880

Inj.Date 12/13/2024 KCH

C:\Users\Public\Documents\ChemStation\1\Data\12_12\L853132\SUPOR_30.M

Z8190786456

MaxPeak: 100.00%
Ret_Time: 1.101 min

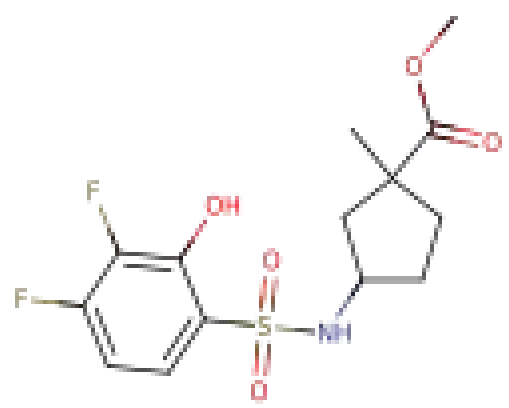

Mol Wt 349.35
Exact Mass 349.09

#	Time	Area%
1	1.101	100.00

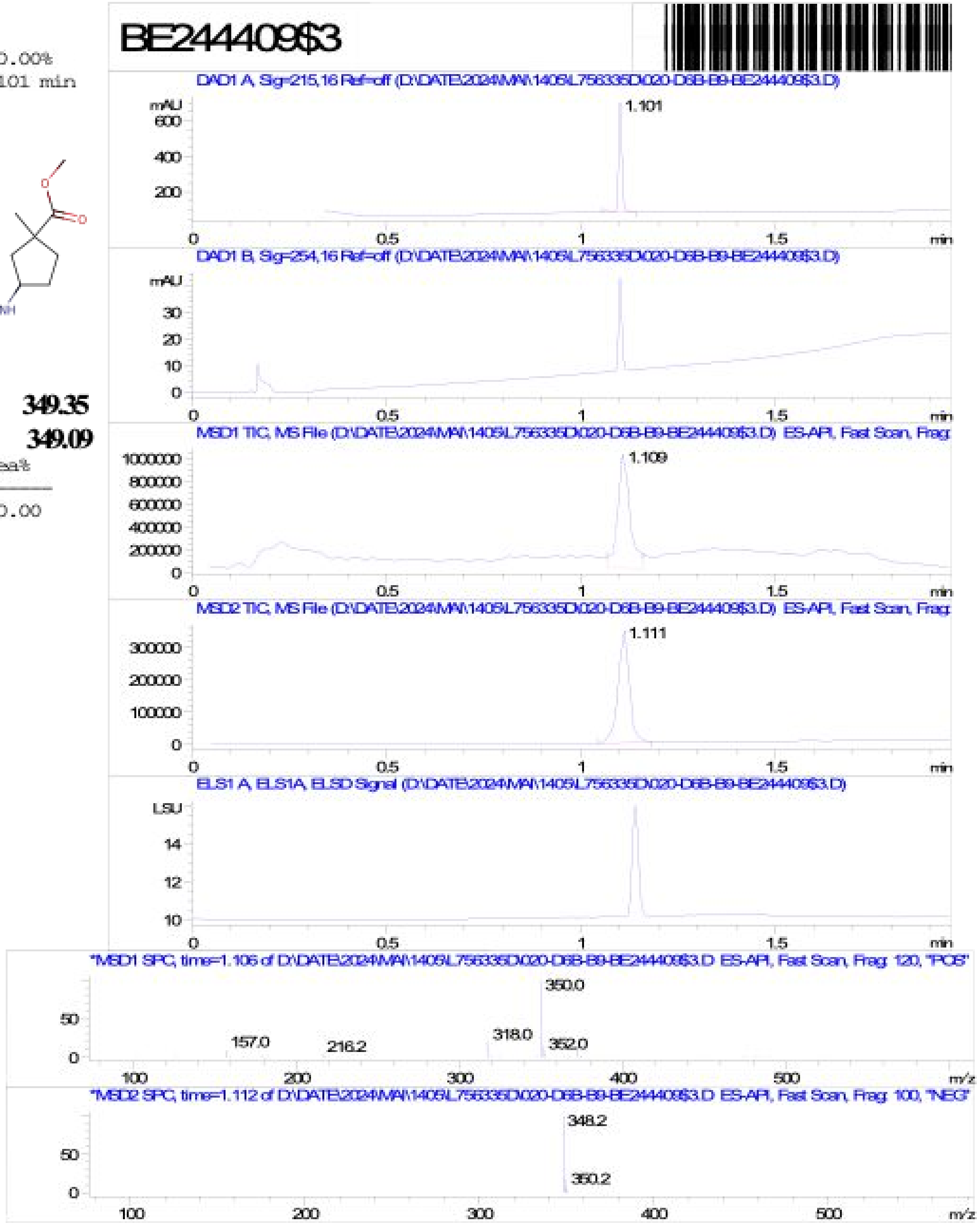

RT 1.109

RT 1.111

Inj.Date 5/13/2024 AN
C:\Users\Public\Documents\ChemStation\1\Data\05_13\L756335D\SUPOR_30.M

Z8208146597

MaxPeak: 98.13%
Ret_Time: 1.237 min

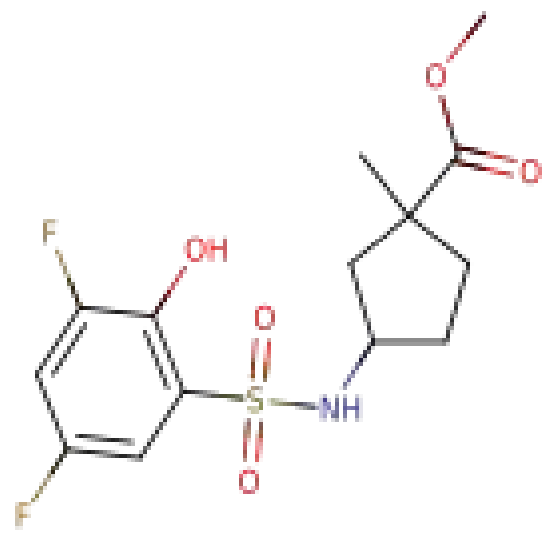

Mol Wt 349.35
Exact Mass 349.09

#	Time	Area%
1	1.237	98.13
2	1.255	1.87

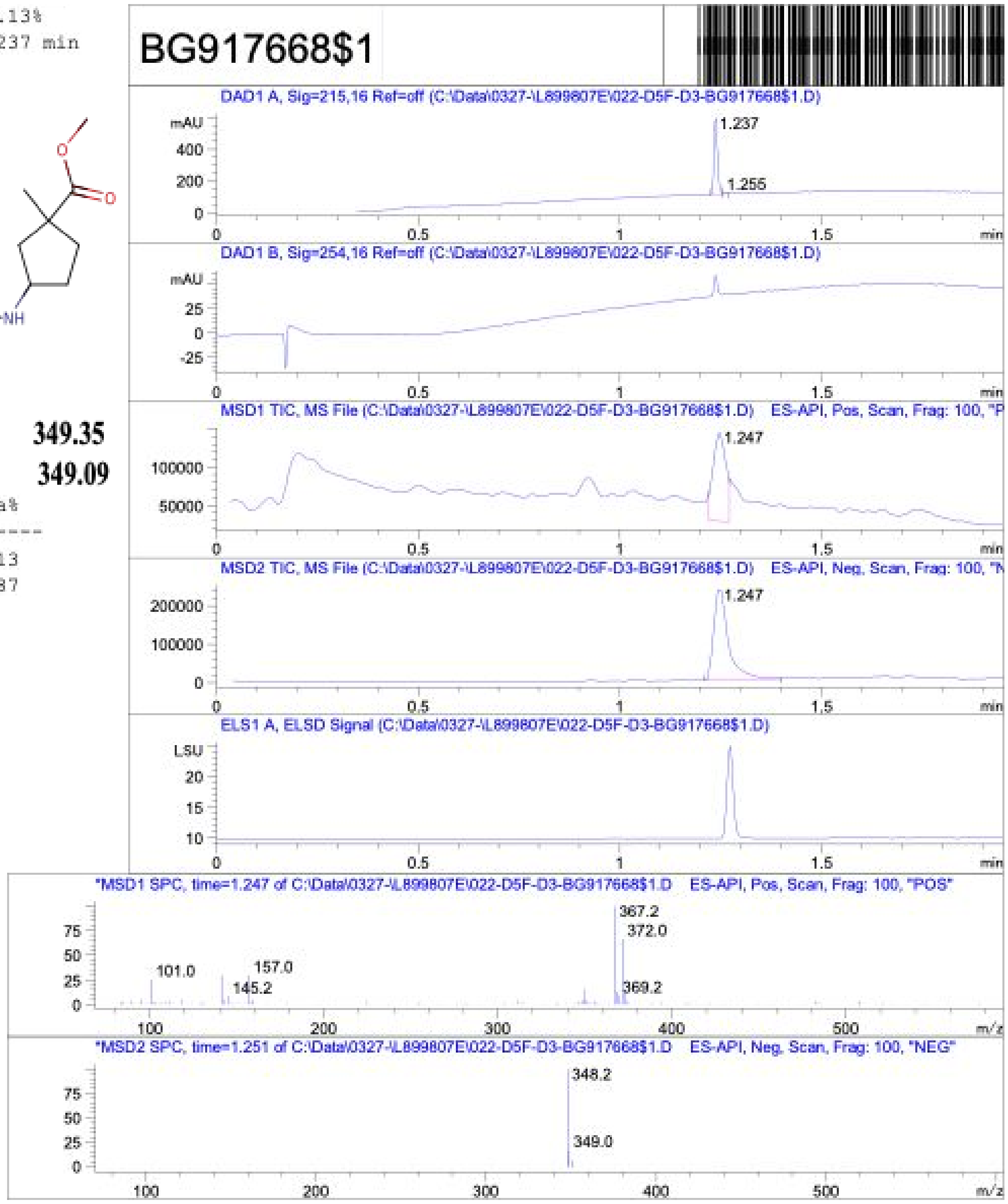

Inj.Date 3/27/2025 KCH -14-
C:\Users\Public\Documents\ChemStation\1\Data\03_27\L899807E\SUPOR_30.M

Z8208146860

MaxPeak: 100.00%
Ret_Time: 1.142 min

BF054169$1

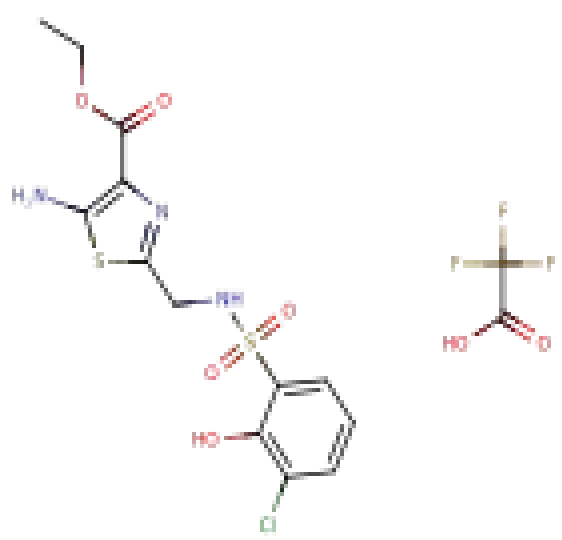

Mol Wt 505.87

Exact Mass 391

#	Time	Area%
1	1.142	100.00

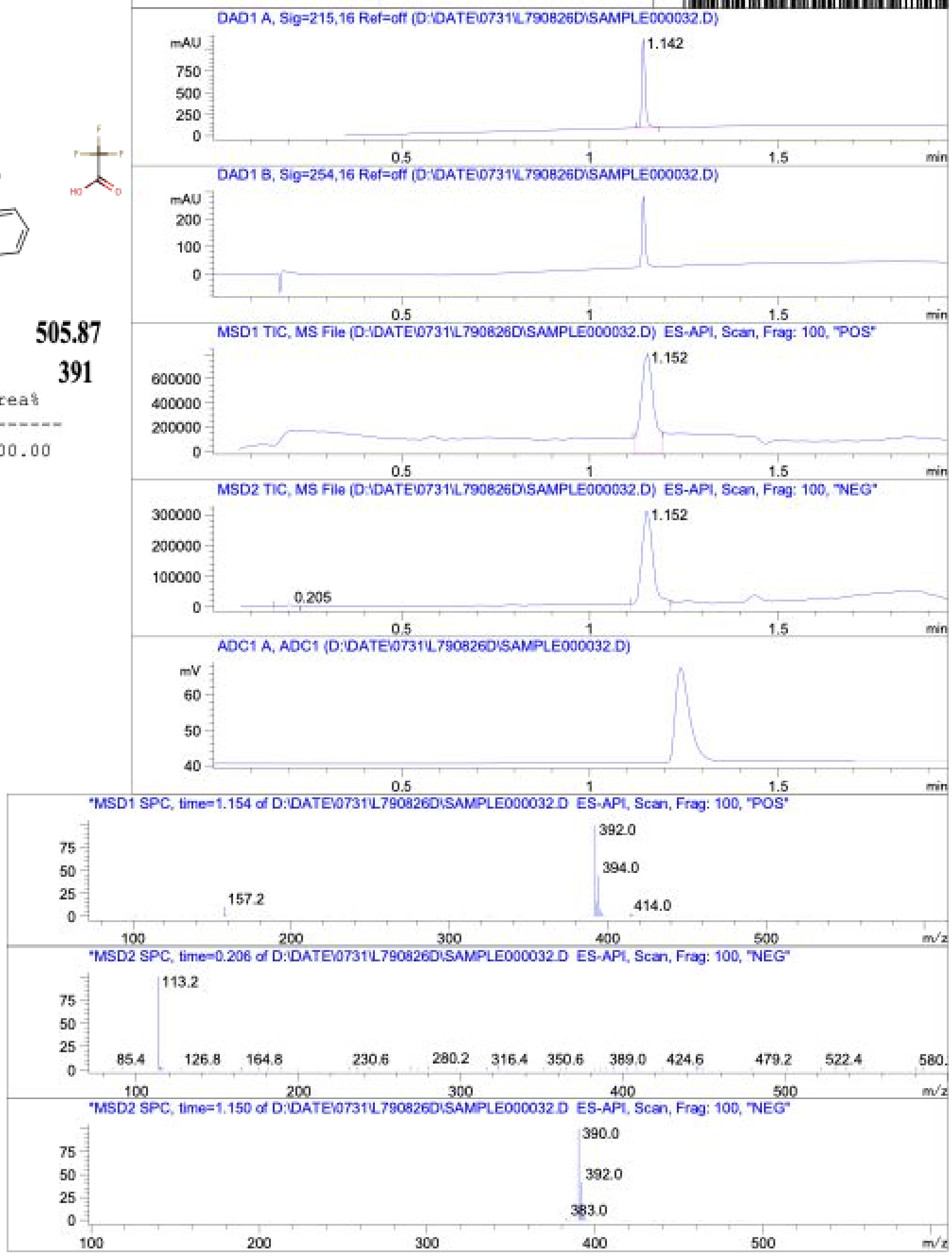

RT 1.152

RT 0.205

RT 1.152

Inj.Date 7/31/2024 E - 4 -
C:\CHEM32\1\METHODS\SUPOR_30.M

Z8233436981

BE244410$21

MaxPeak: 97.24%
Ret_Time: 1.079 min

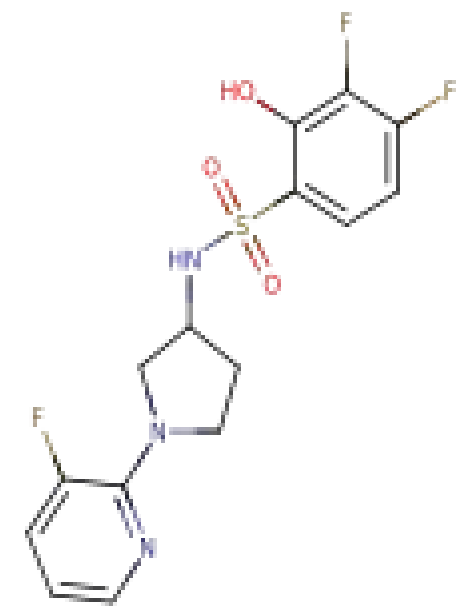

Mol Wt 373.35
Exact Mass 373.08

#	Time	Area%
1	1.055	2.76
2	1.079	97.24

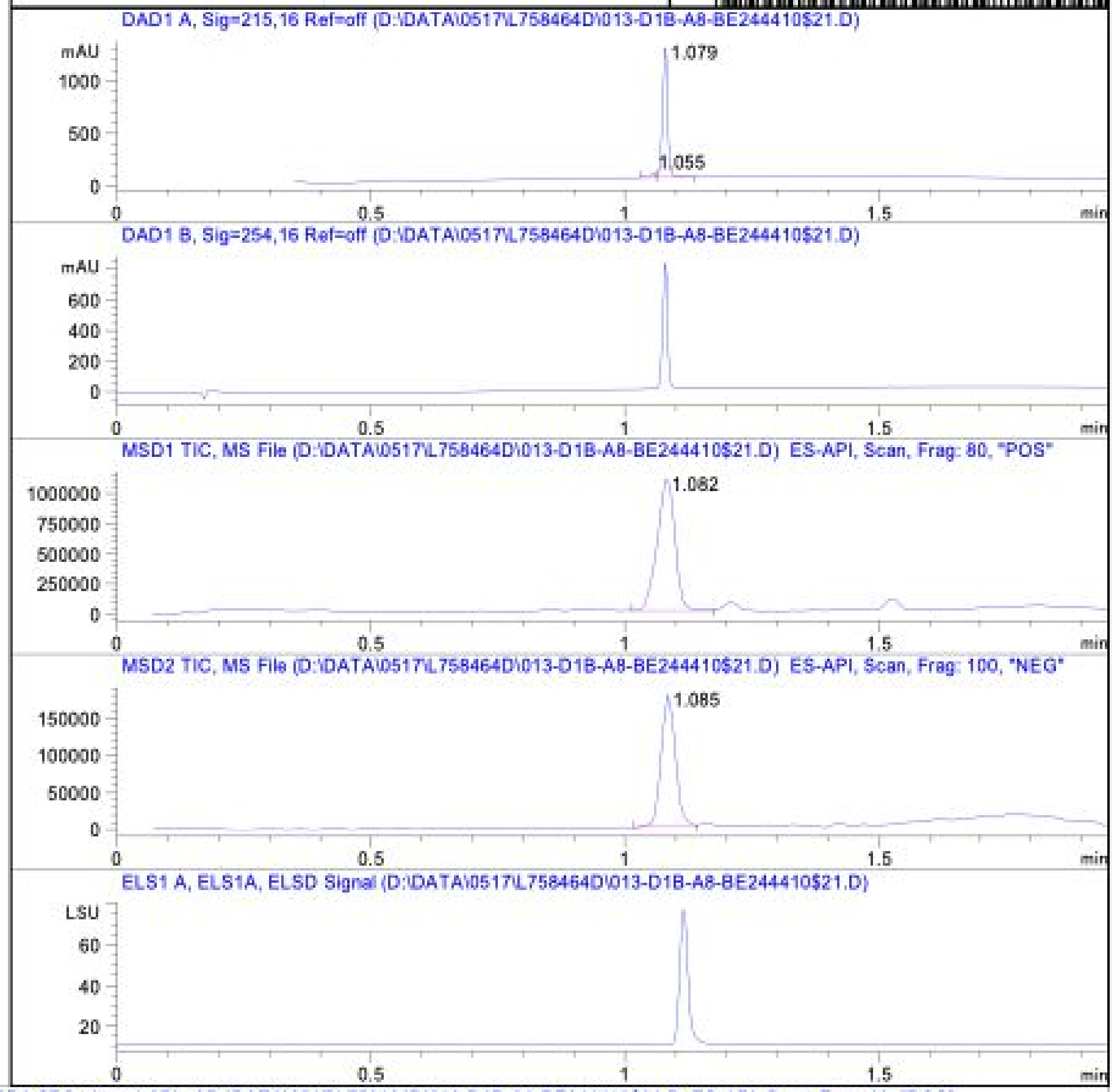

RT 1.082

RT 1.085

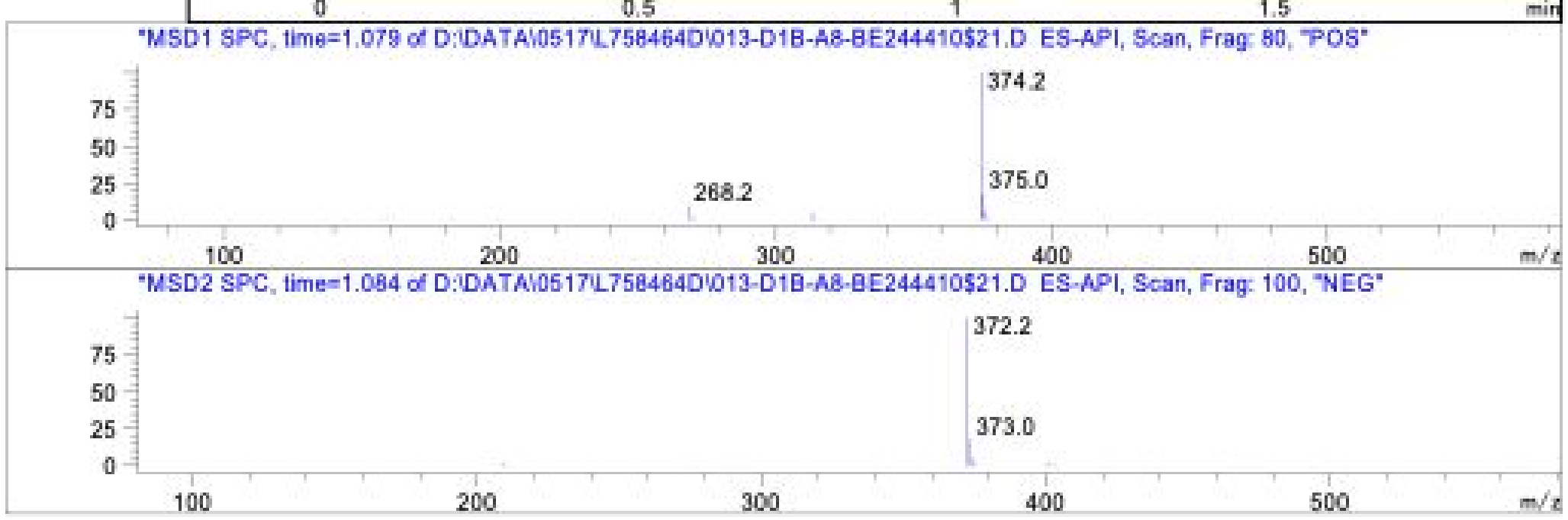

Inj.Date 5/17/2024 VB -16-
C:\Chem32\1\Data\05_17\L758464D\SUPOR_96.M

Z8238874619

MaxPeak: 100.00%
Ret_Time: 1.251 min

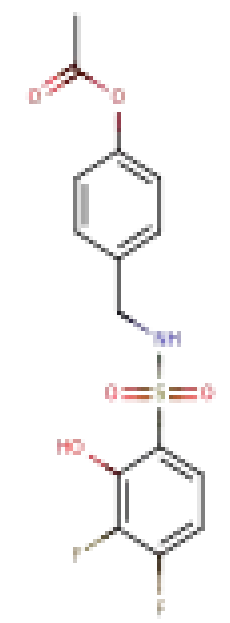

Mol Wt 357.33

Exact Mass 357.05

#	Time	Area%
1	1.251	100.00

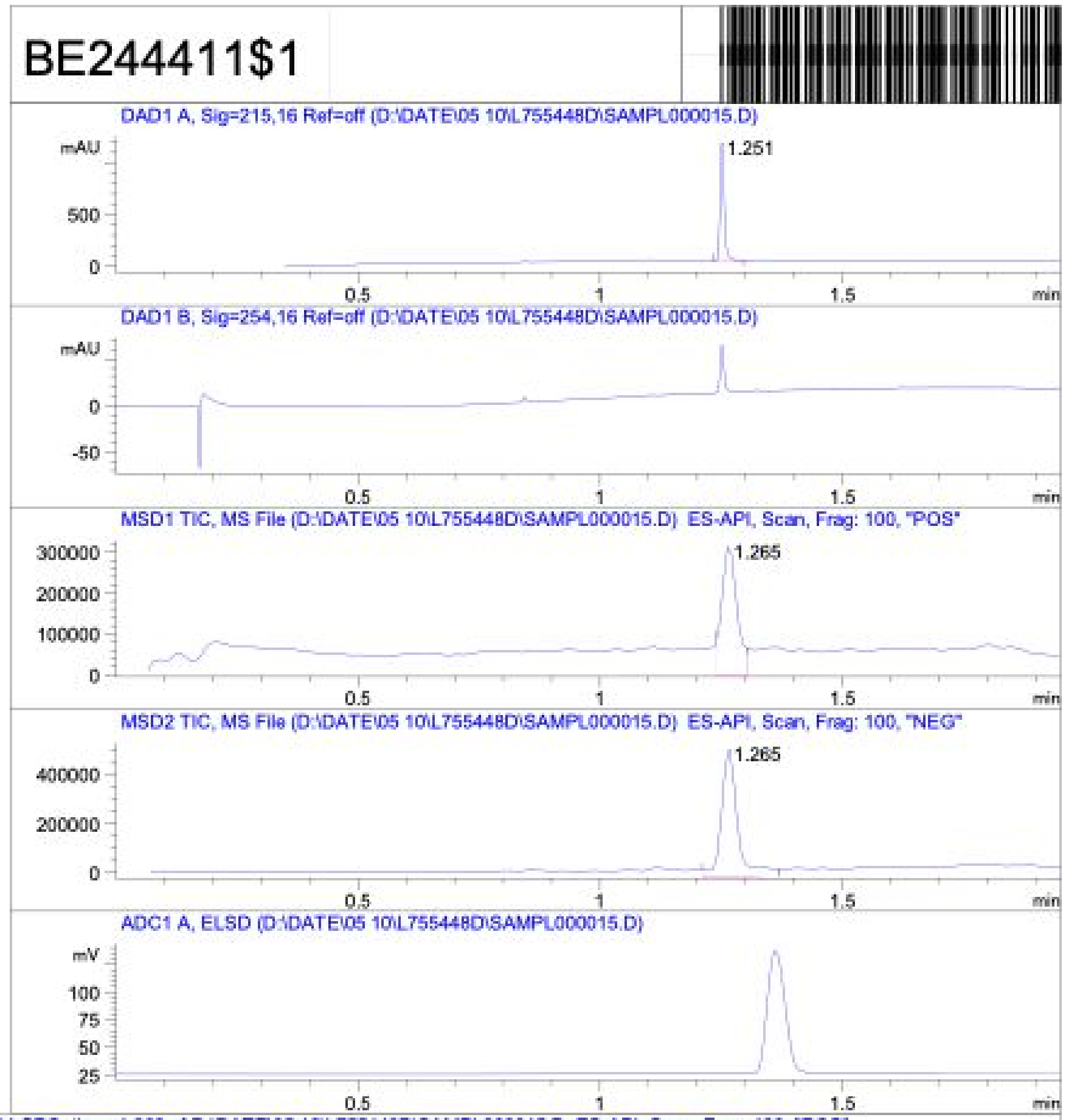

RT 1.265

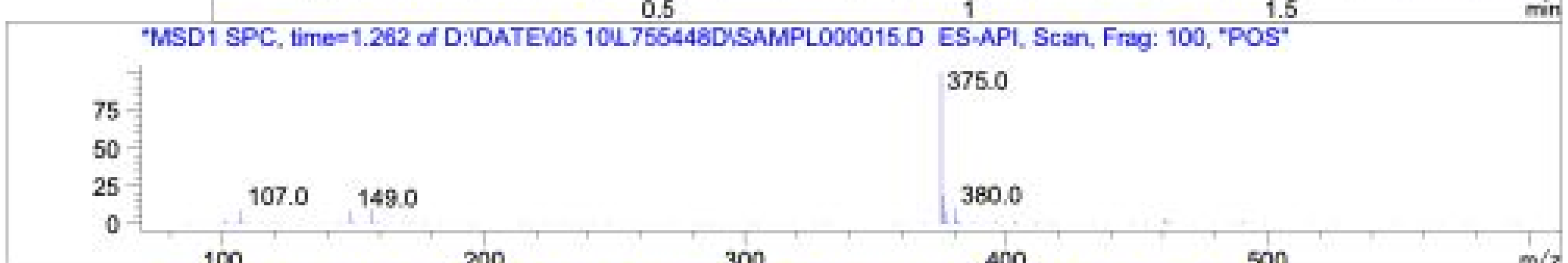

RT 1.265

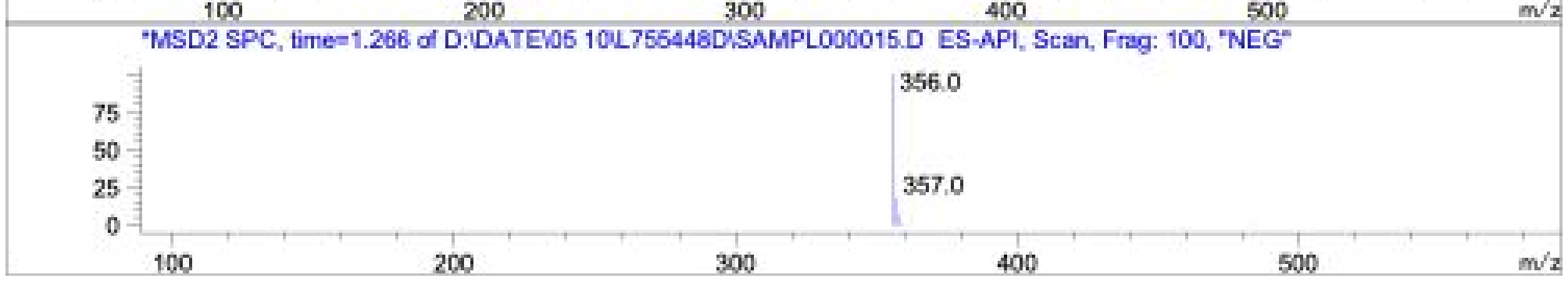

Inj.Date 5/10/2024 E -6-
C:\CHEM32\1\METHODS\SUPOR_30.M

Z8238927651

MaxPeak: 98.96%
Ret_Time: 0.972 min

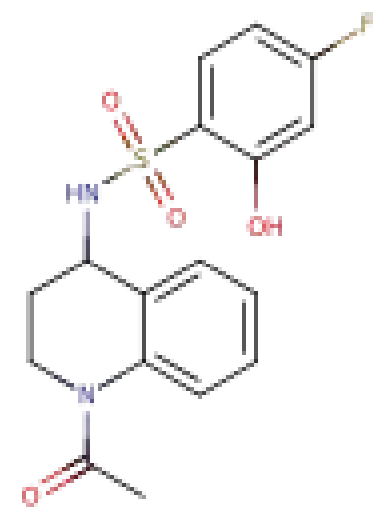

Mol Wt 364.39
Exact Mass 364.1

#	Time	Area%
1	0.972	98.96
2	1.088	1.04

RT 0.979

RT 0.982

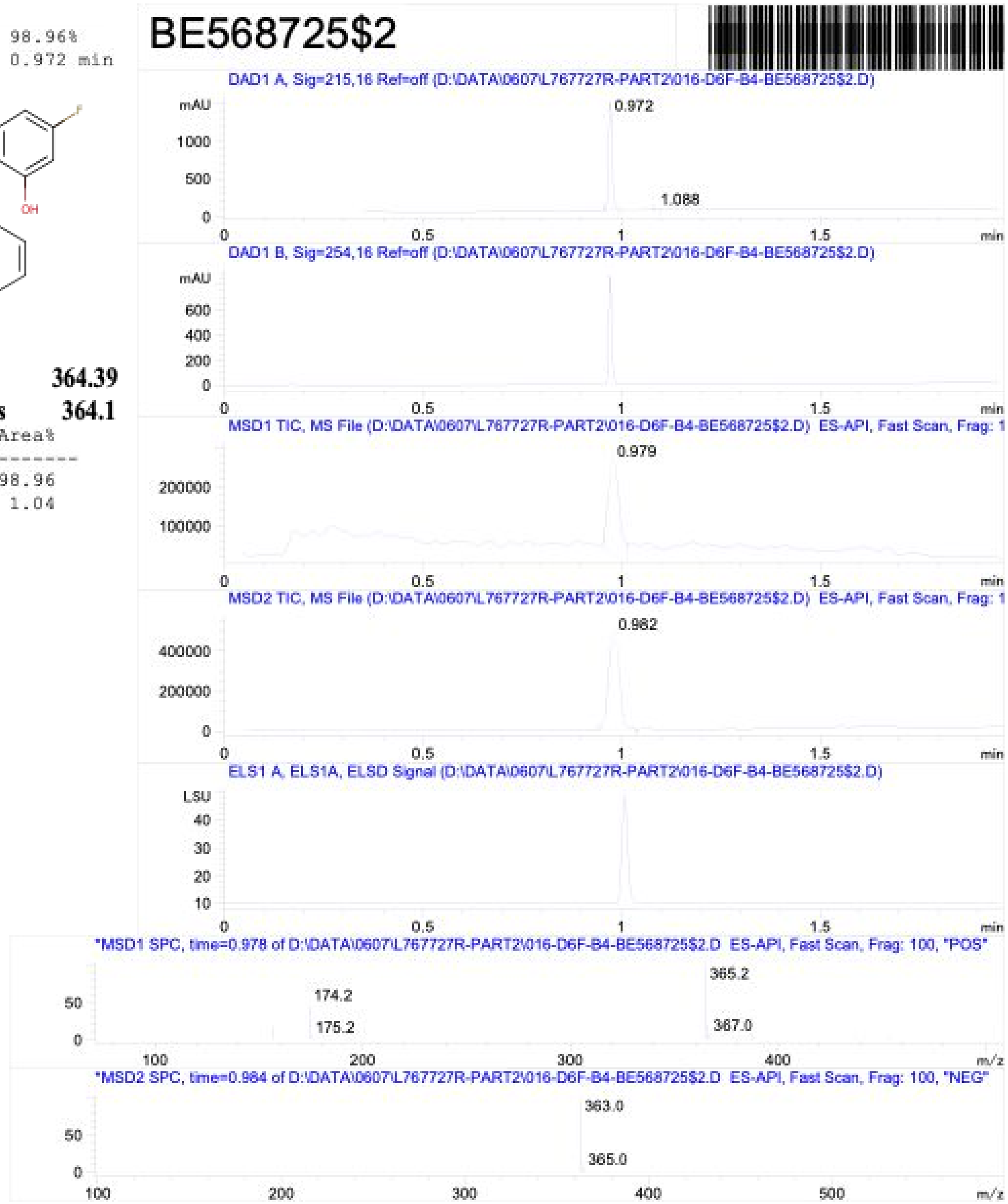

Inj.Date 6/7/2024 H 32
C:\Users\Public\Documents\ChemStation\1\Data\06_07\2767727R\SUPOR_30.M

Z8239890489

MaxPeak: 100.00%
Ret_Time: 1.319 min

BG917677$1

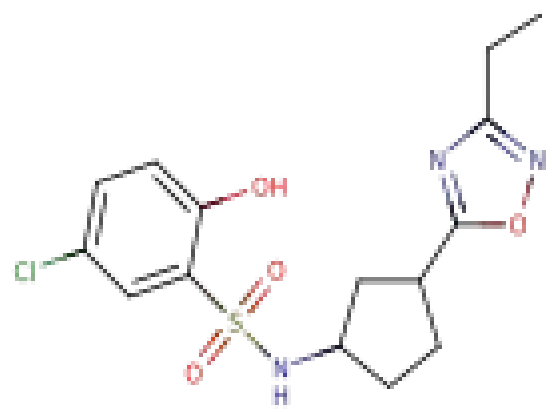

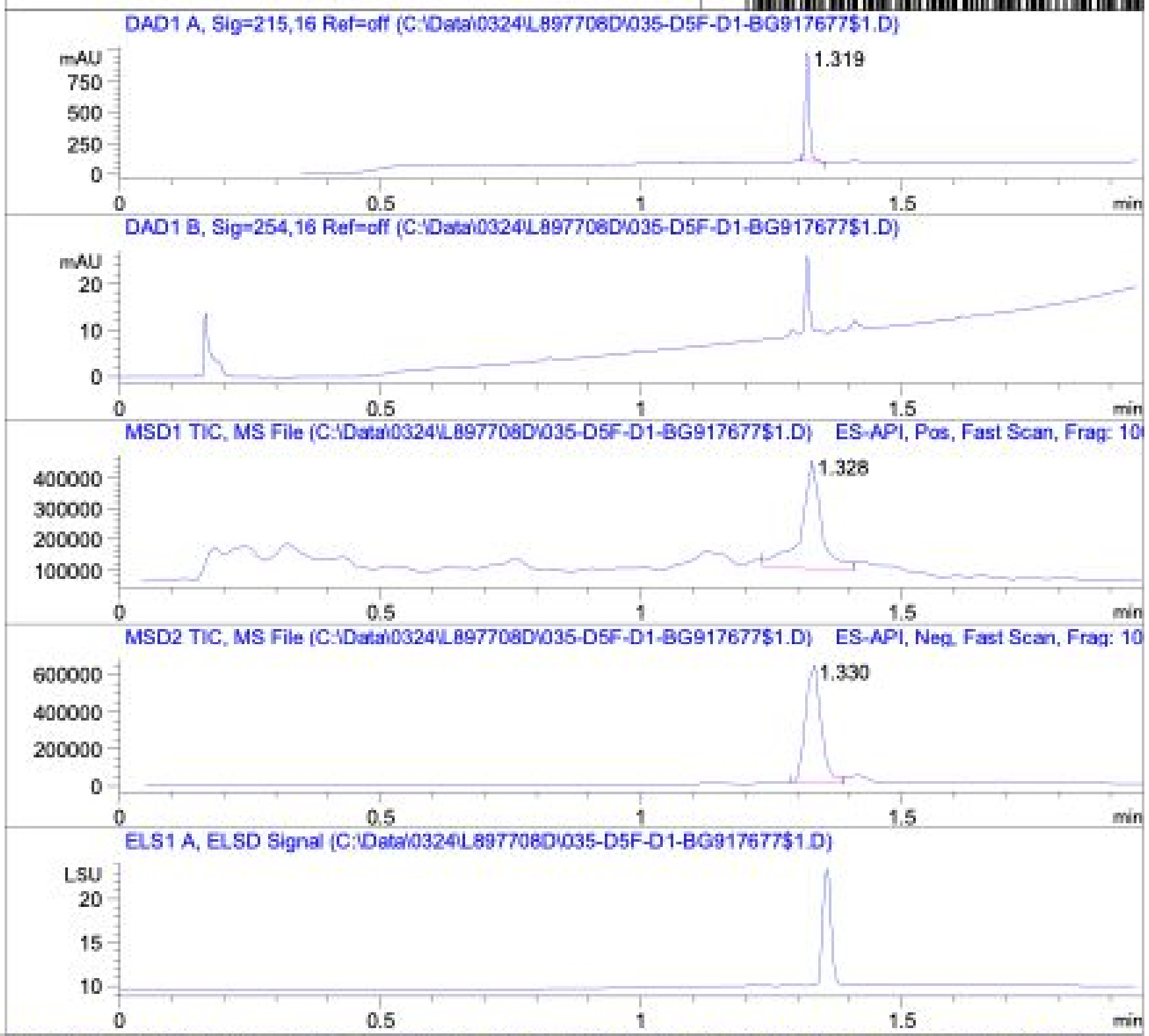

Mol Wt 371.84

Exact Mass 371.08

#	Time	Area%
1	1.319	100.00

RT 1.328

RT 1.330

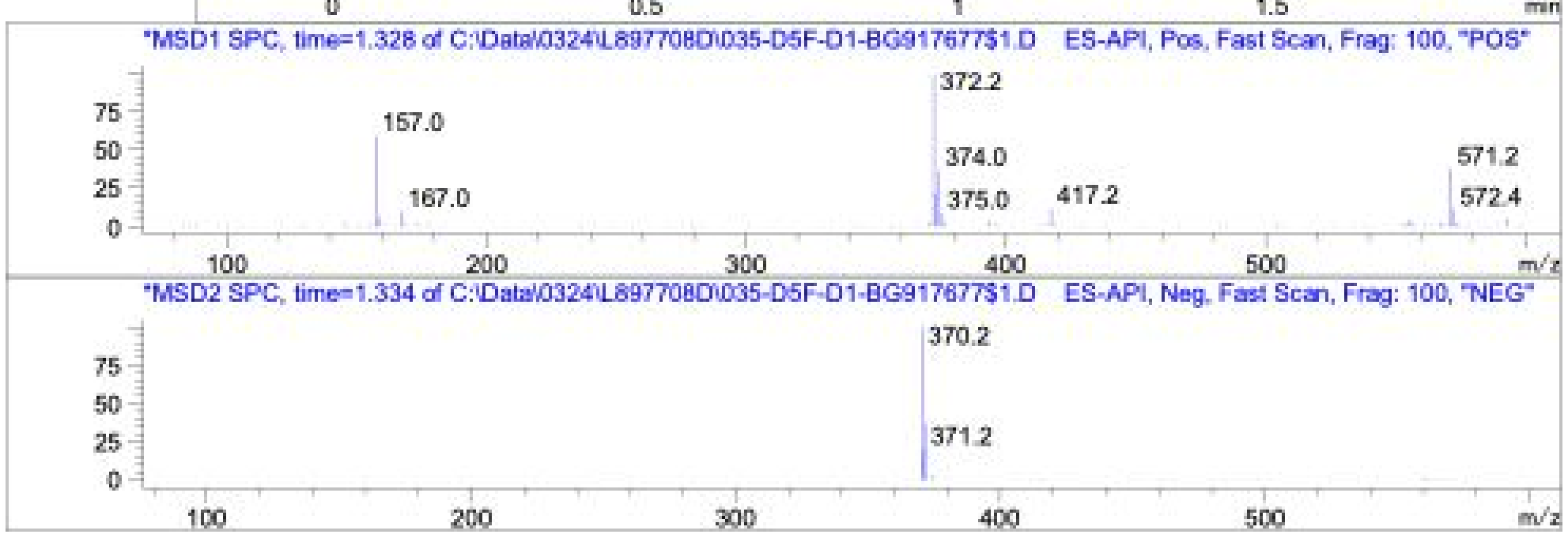

Inj.Date 3/21/2025 NEW -33-
C:\Users\Public\Documents\ChemStation\1\Data\03_21\L897708D\SUPOR_30.M

Z8241970648

MaxPeak: 100.00%
Ret_Time: 0.856 min

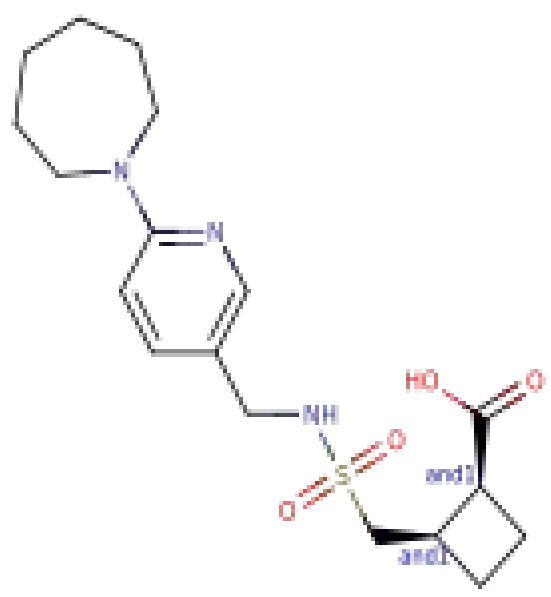

Mol Wt 381.49
Exact Mass 381.2

#	Time	Area%
1	0.856	100.00

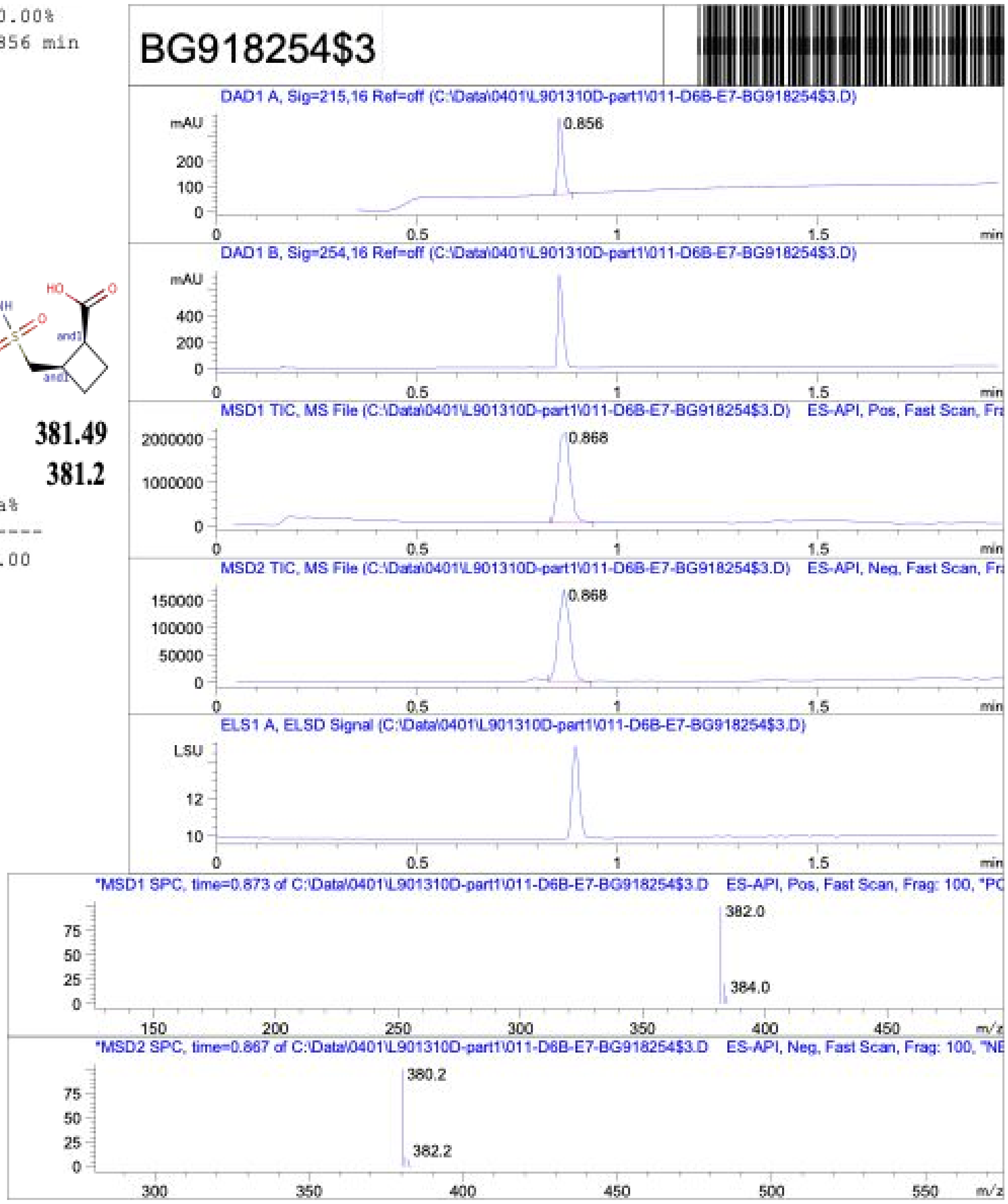

RT 0.868

RT 0.868

Inj.Date 3/31/2025 NEW -34-
C:\Users\Public\Documents\ChemStation\1\Data\03_31\1901310D\SUPOR_30.M

Z8269237535

MaxPeak: 100.00%
Ret_Time: 1.336 min

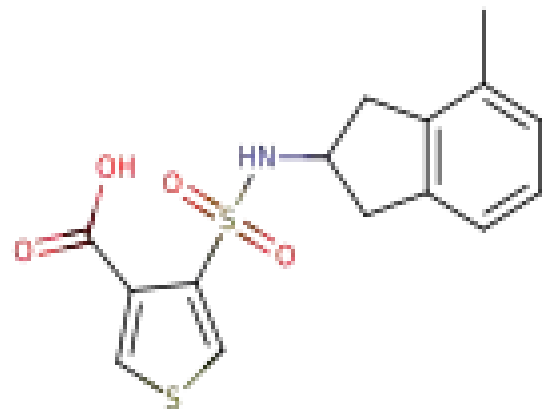

BE244414$2

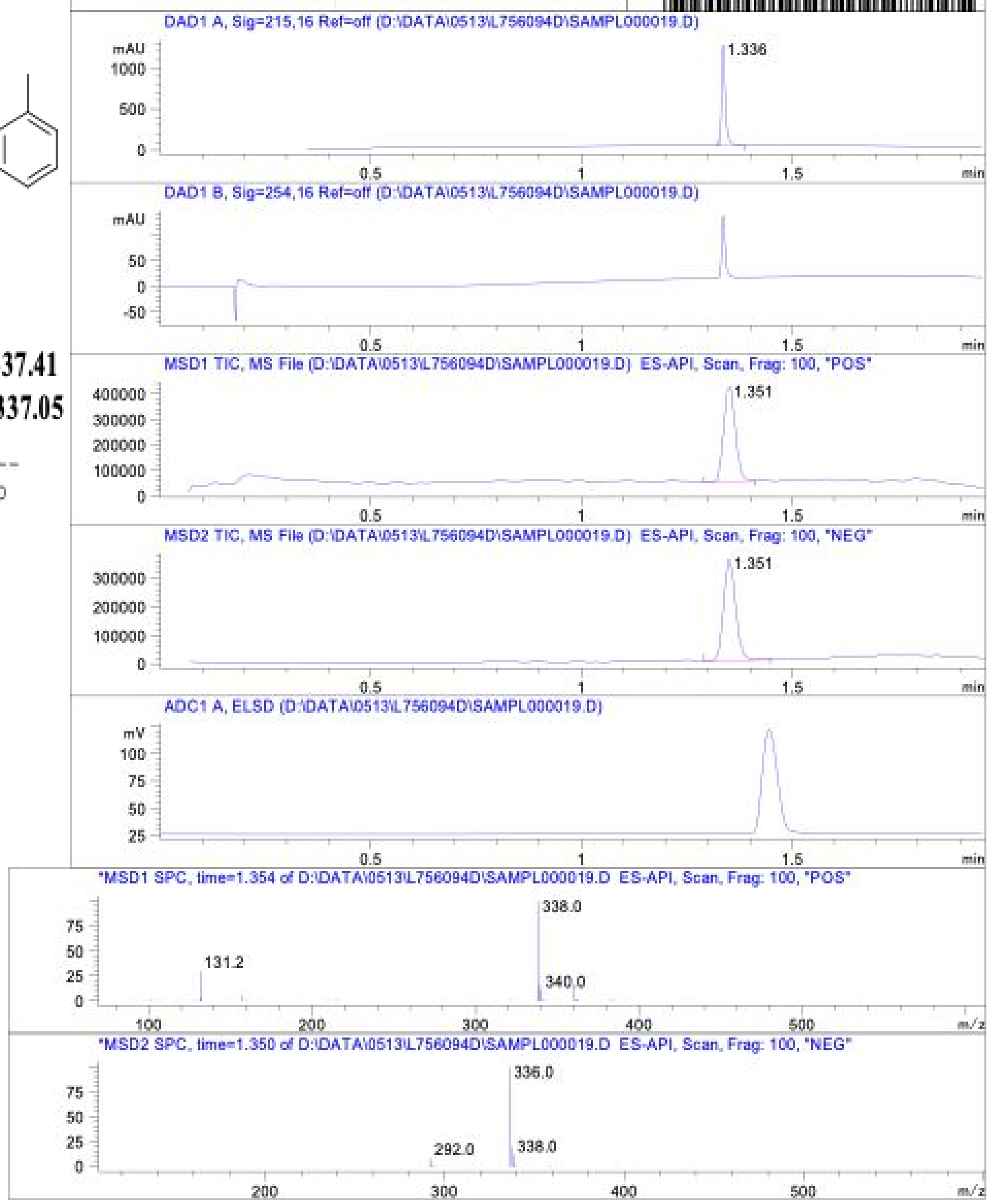

Mol Wt 337.41

Exact Mass 337.05

#	Time	Area%
1	1.336	100.00

RT 1.351

RT 1.351

Inj.Date 5/13/2024 LB -6-
C:\CHEM32\1\METHODS\SUPOR_30.M

Z8278108755

MaxPeak: 100.00%
Ret_Time: 1.185 min

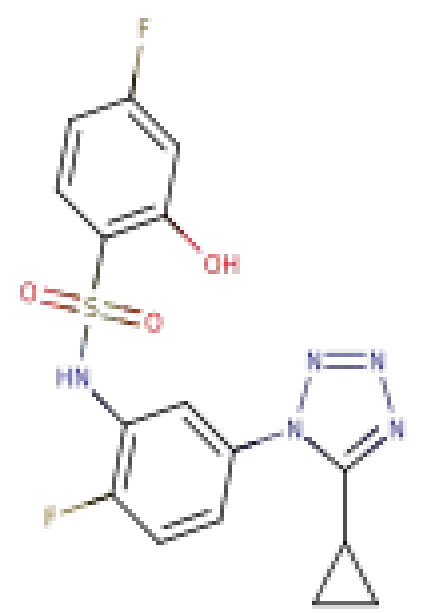

Mol Wt 393.37
Exact Mass 393.07

#	Time	Area%
1	1.185	100.00

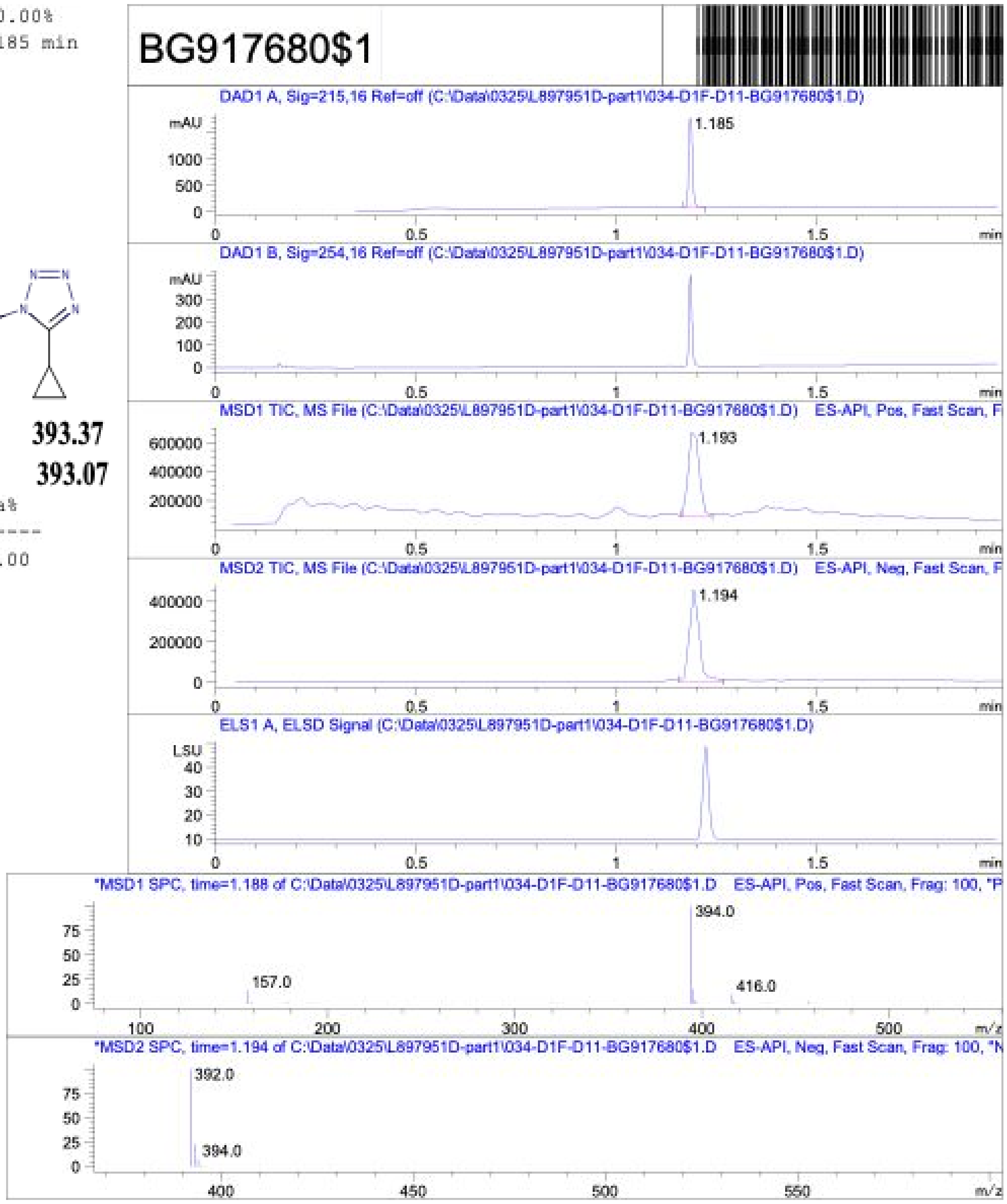

RT 1.193

RT 1.194

Inj.Date 3/25/2025 NEW -36-
C:\Users\Public\Documents\ChemStation\1\Data\03_24\1897951D\SUPOR_96.M

Z8318206664

MaxPeak: 98.12%
Ret_Time: 1.221 min

BE483090$5

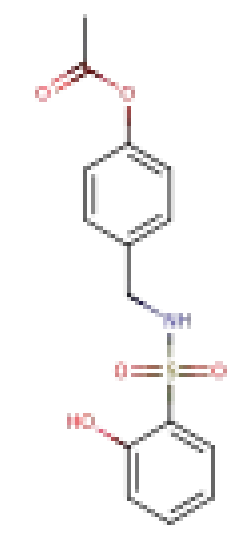

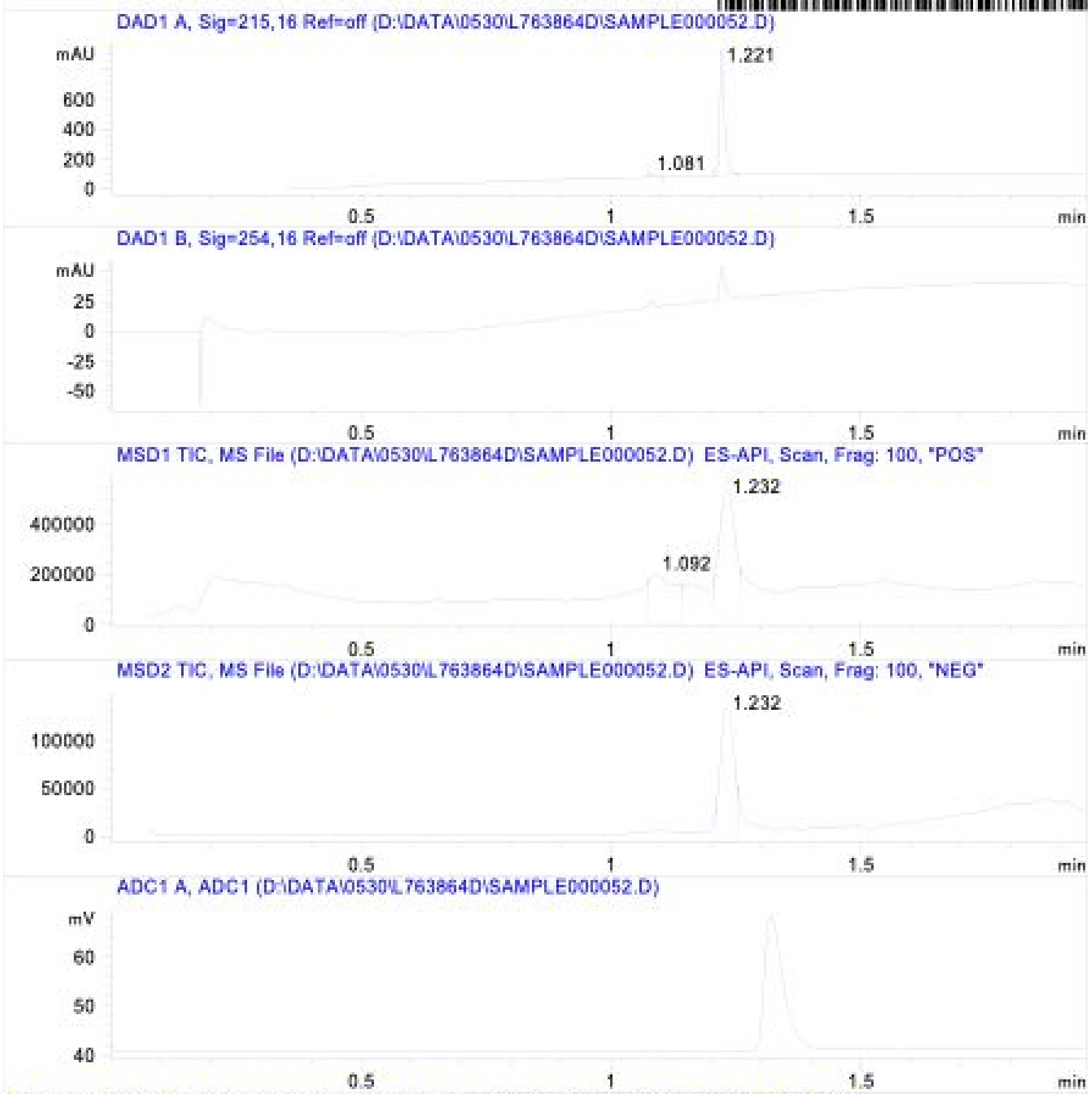

Mol Wt 321.35
Exact Mass 321.07

#	Time	Area%
1	1.081	1.88
2	1.221	98.12

RT 1.092

RT 1.232

RT 1.232

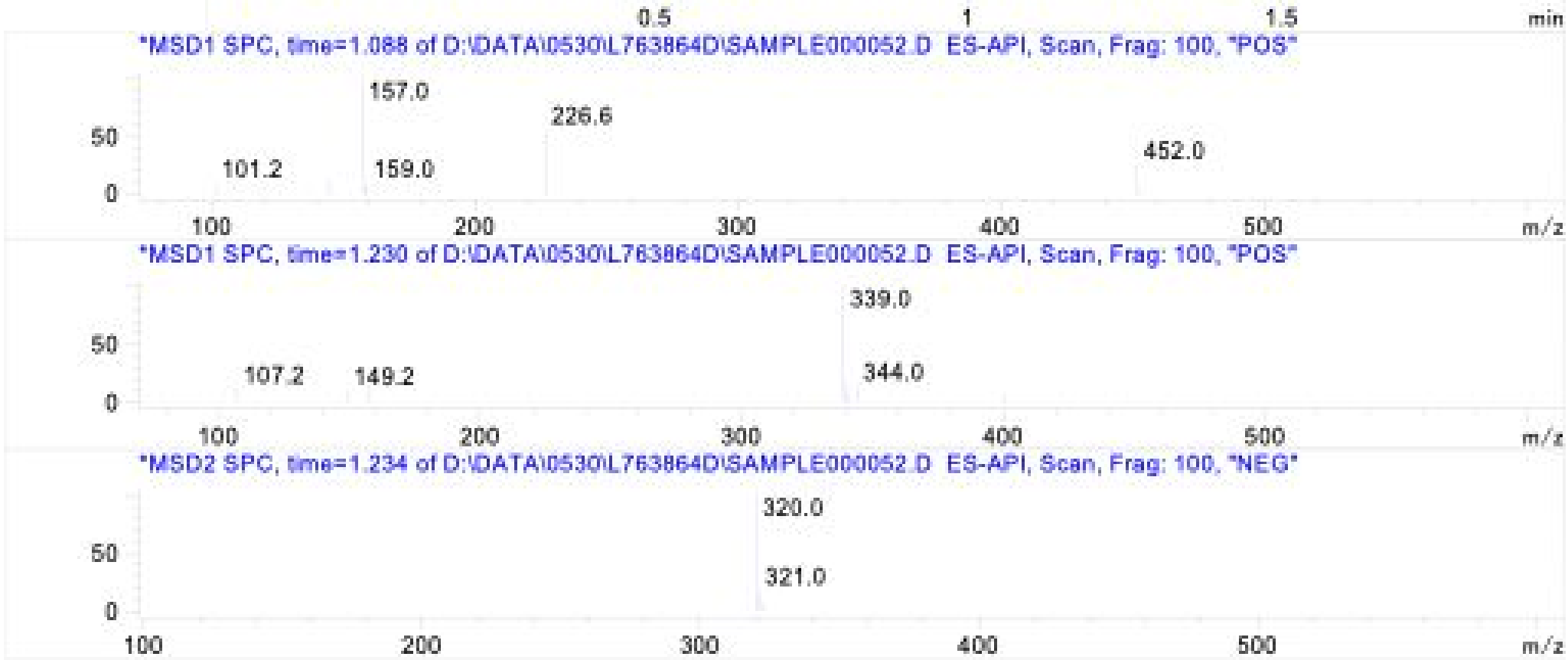

Inj.Date 5/30/2024 H - 4 -
C:\CHEM32\1\METHODS\SUPOR_30.M

Z9103951905

MaxPeak: 100.00%
Ret_Time: 1.641 min

BE244418$11

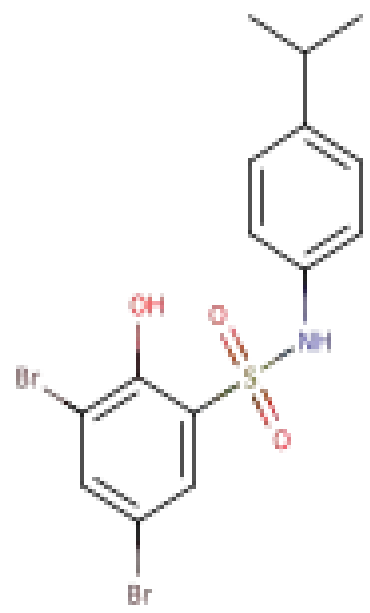

Mol Wt 449.16

Exact Mass 448.93

#	Time	Area%
1	1.641	100.00

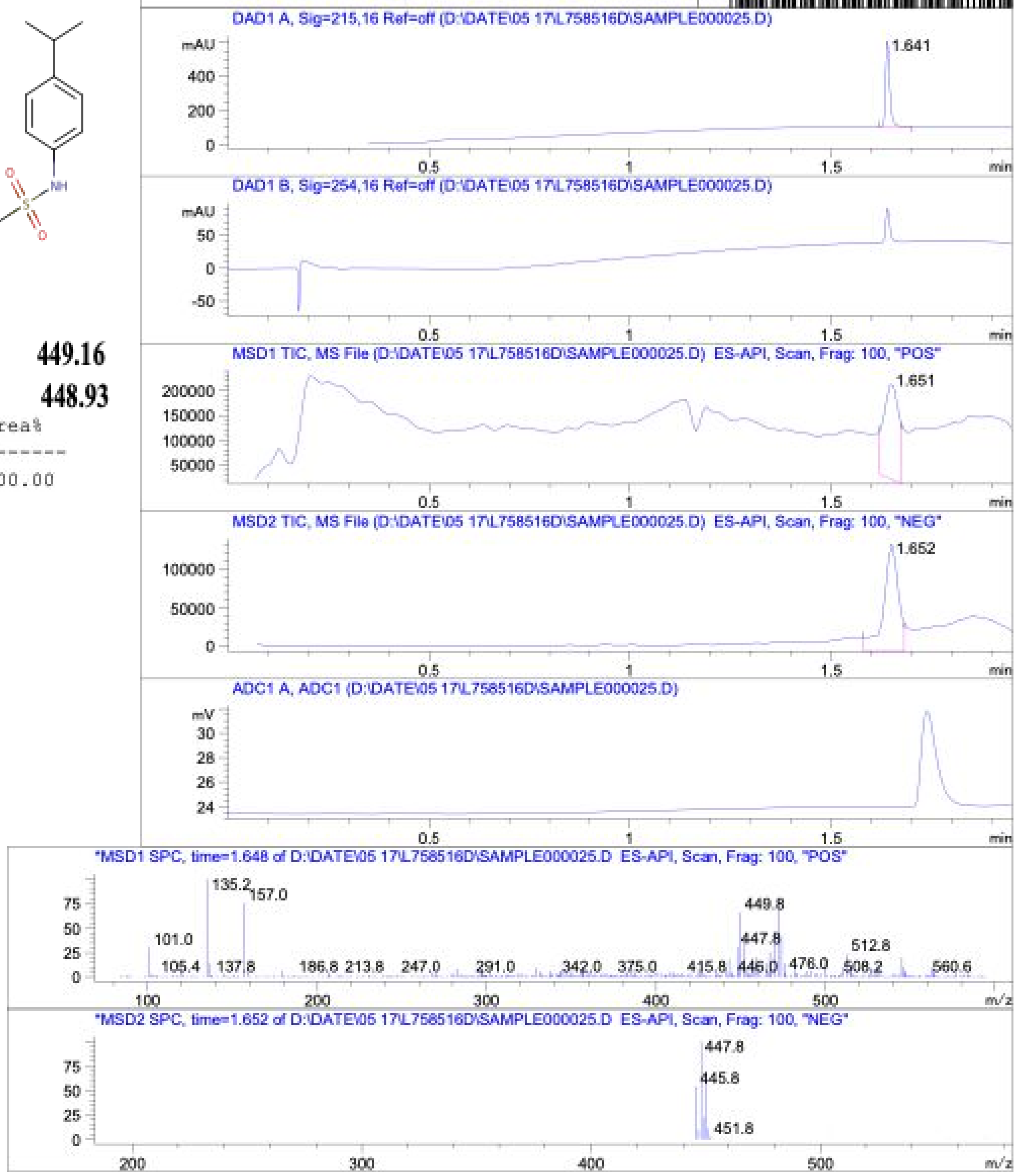

RT 1.651

RT 1.652

Inj.Date 5/17/2024 E - 4 -
C:\CHEM32\1\METHODS\SUPOR_30.M

Z9103951919

MaxPeak: 100.00%
Ret_Time: 1.211 min

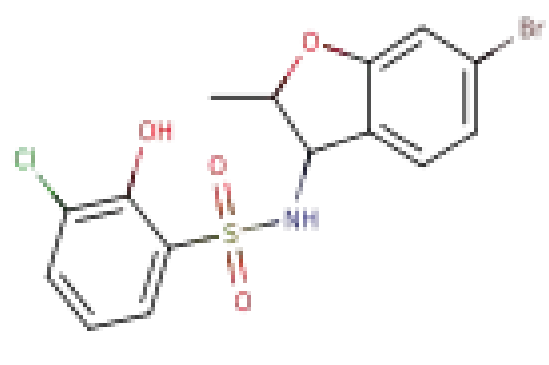

Mol Wt 418.69

Exact Mass 418.95

#	Time	Area%
1	1.211	100.00

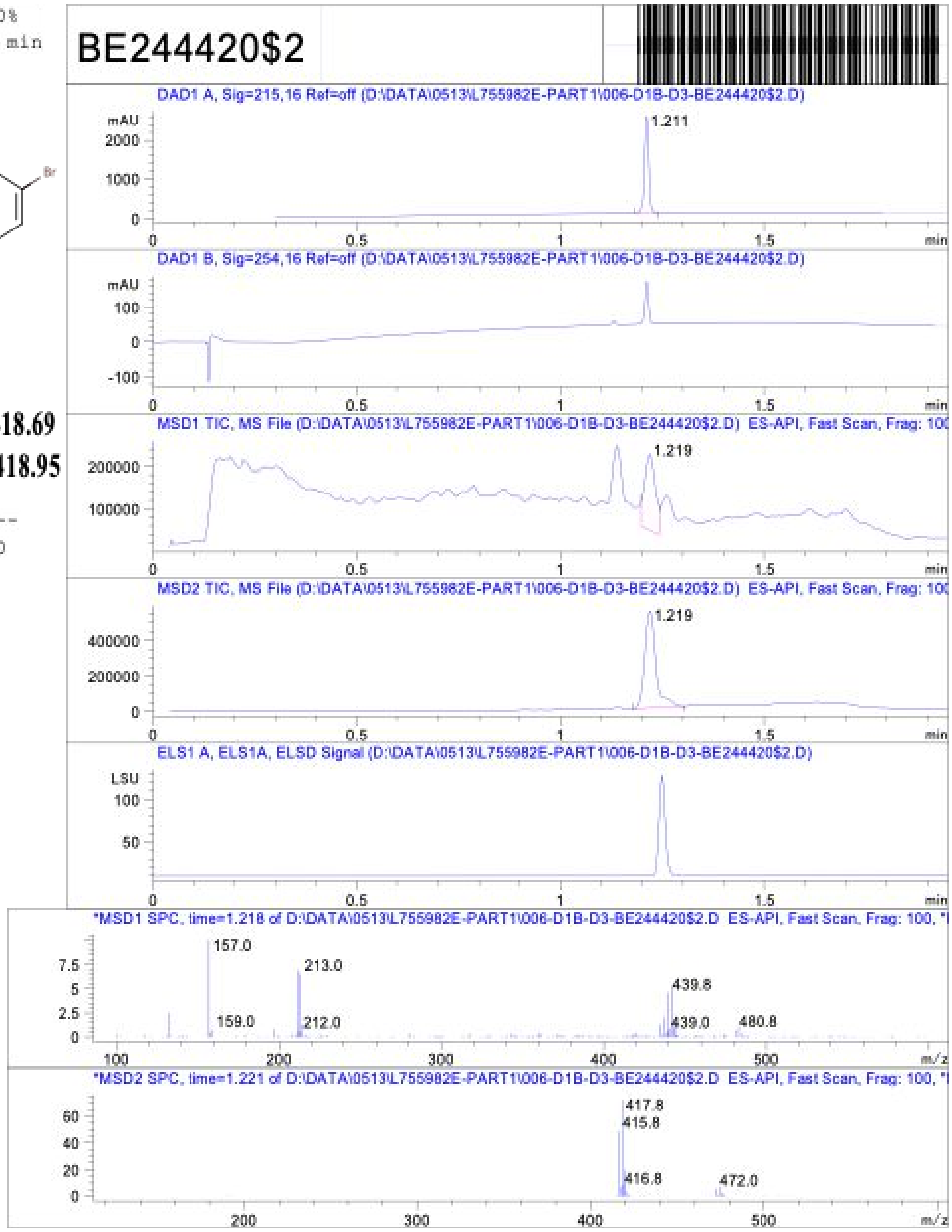

RT 1.219

RT 1.219

Inj.Date 5/13/2024 LB
C:\Users\Public\Documents\ChemStation\1\Data\05_13\1755982E\SUPOR_96.M

Z9103951948

MaxPeak: 100.00%
Ret_Time: 1.035 min

BE244421$3

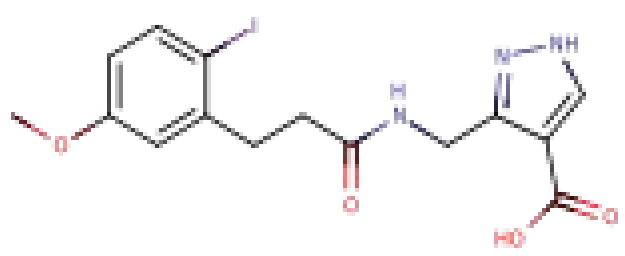

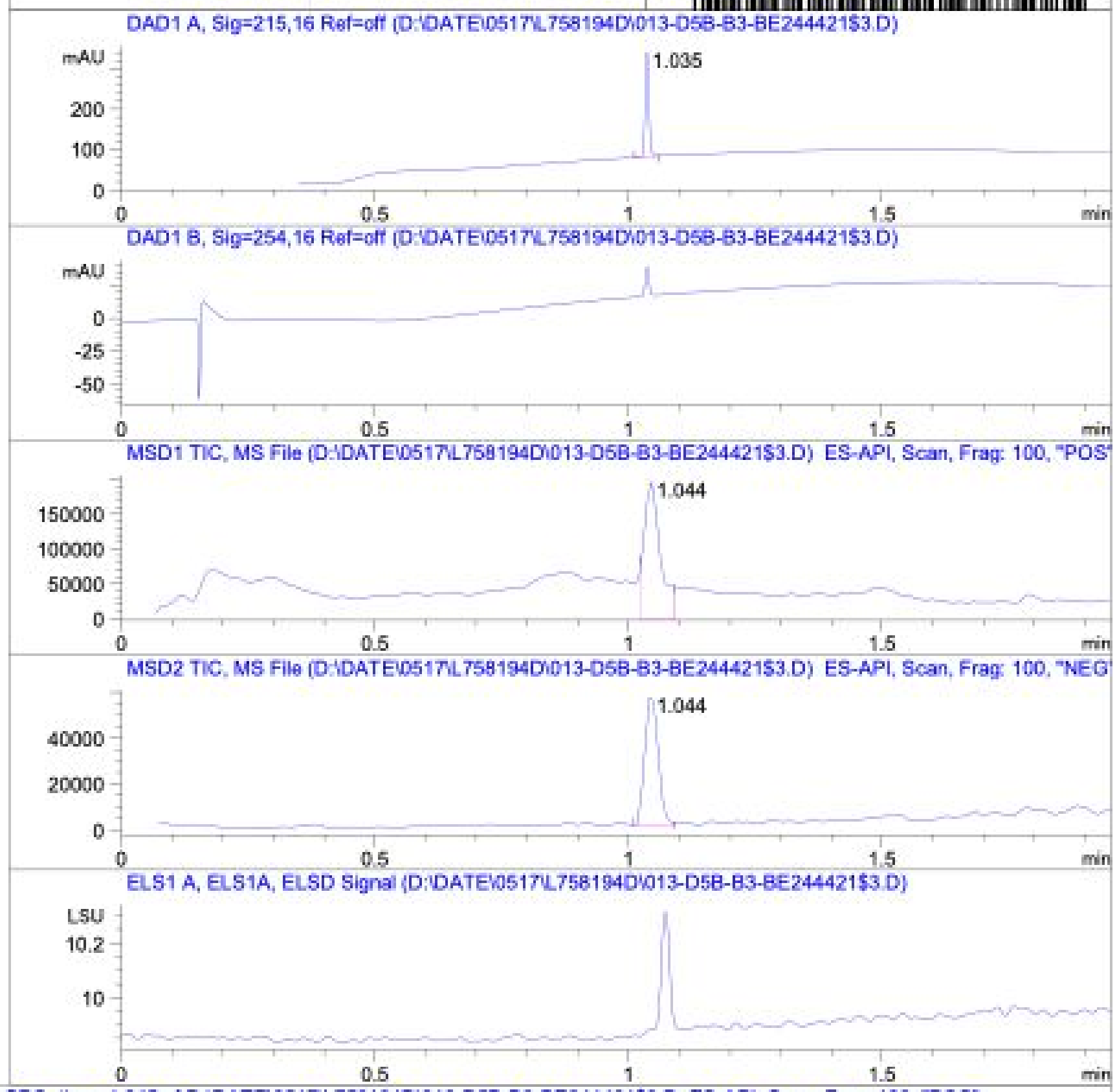

Mol Wt 429.21
Exact Mass 429.02

#	Time	Area%
1	1.035	100.00

RT 1.044

RT 1.044

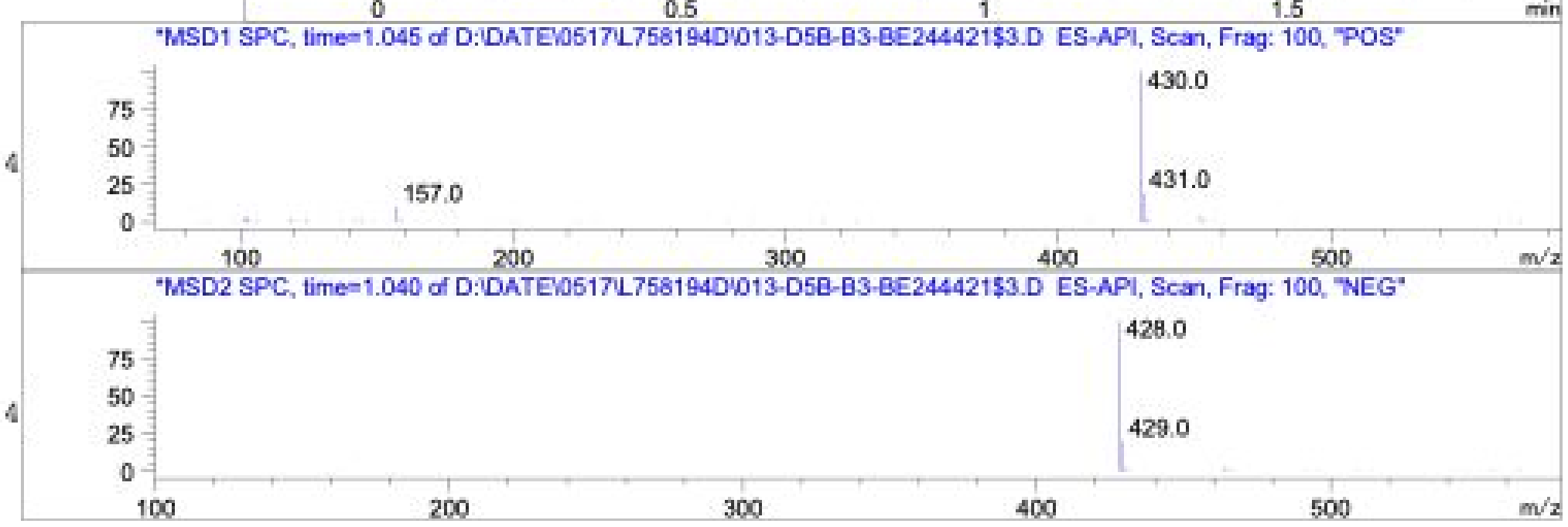

Inj.Date 5/16/2024 CH -18-
C:\Chem32\1\Data\05_16\L758194D\SUPOR_30.M

Z9103952005

MaxPeak: 100.00%
Ret_Time: 1.253 min

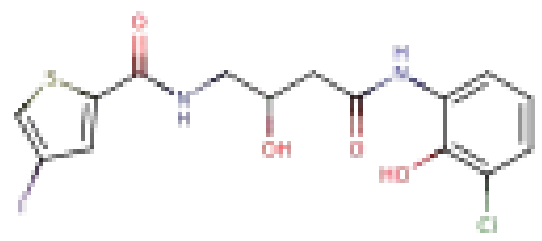

Mol Wt 480.7

Exact Mass 479.94

#	Time	Area%
1	1.253	100.00

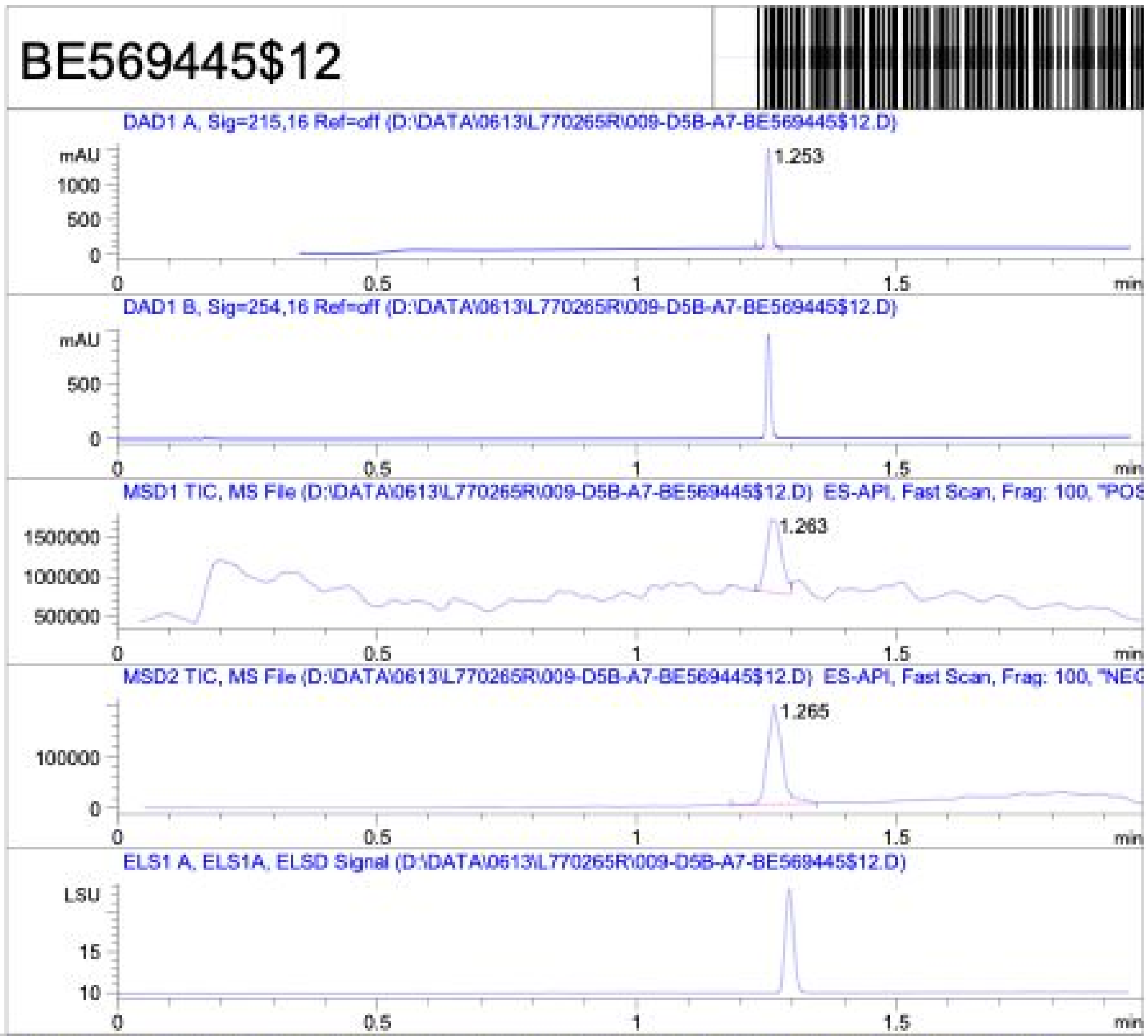

RT 1.263

RT 1.265

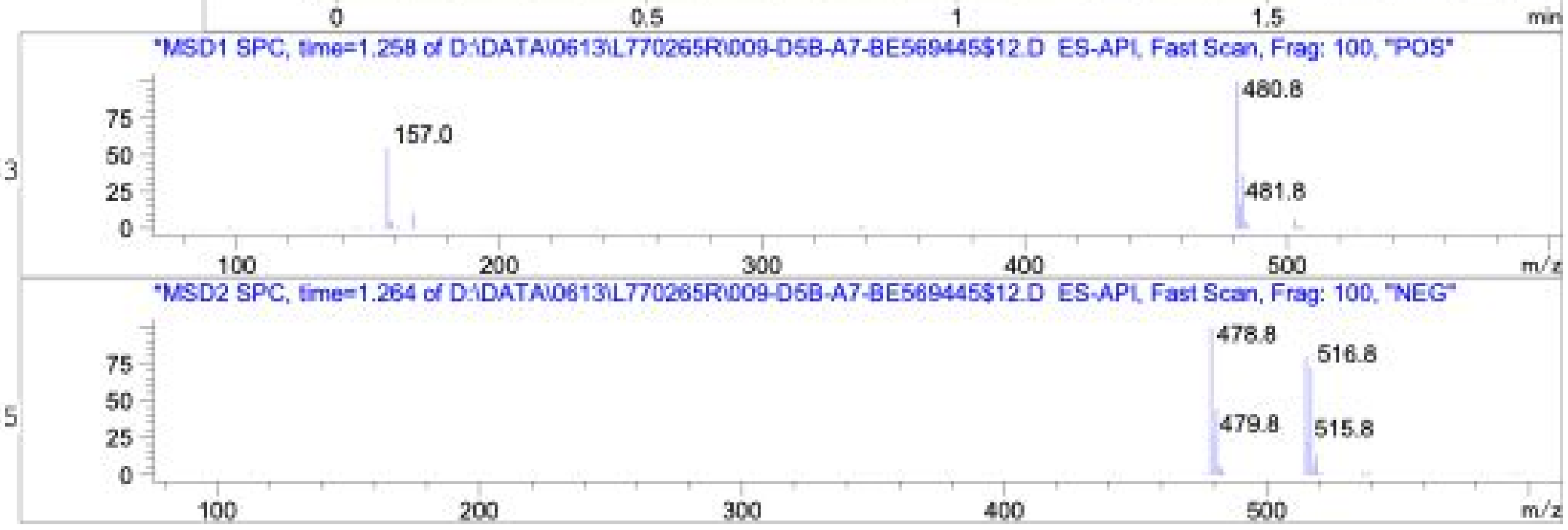

Inj.Date 6/13/2024 M

C:\Users\Public\Documents\ChemStation\1\Data\06_13\L770265R\SUPOR_30.M

Z9103952022

MaxPeak: 98.63%
Ret_Time: 1.065 min

BE639169$2

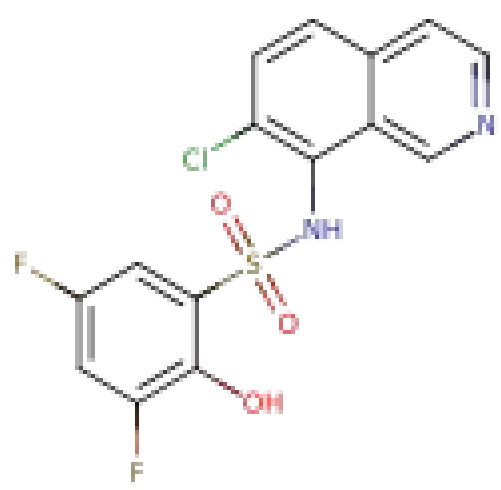

Mol Wt 370.76
Exact Mass 370

#	Time	Area%
1	1.014	1.37
2	1.065	98.63

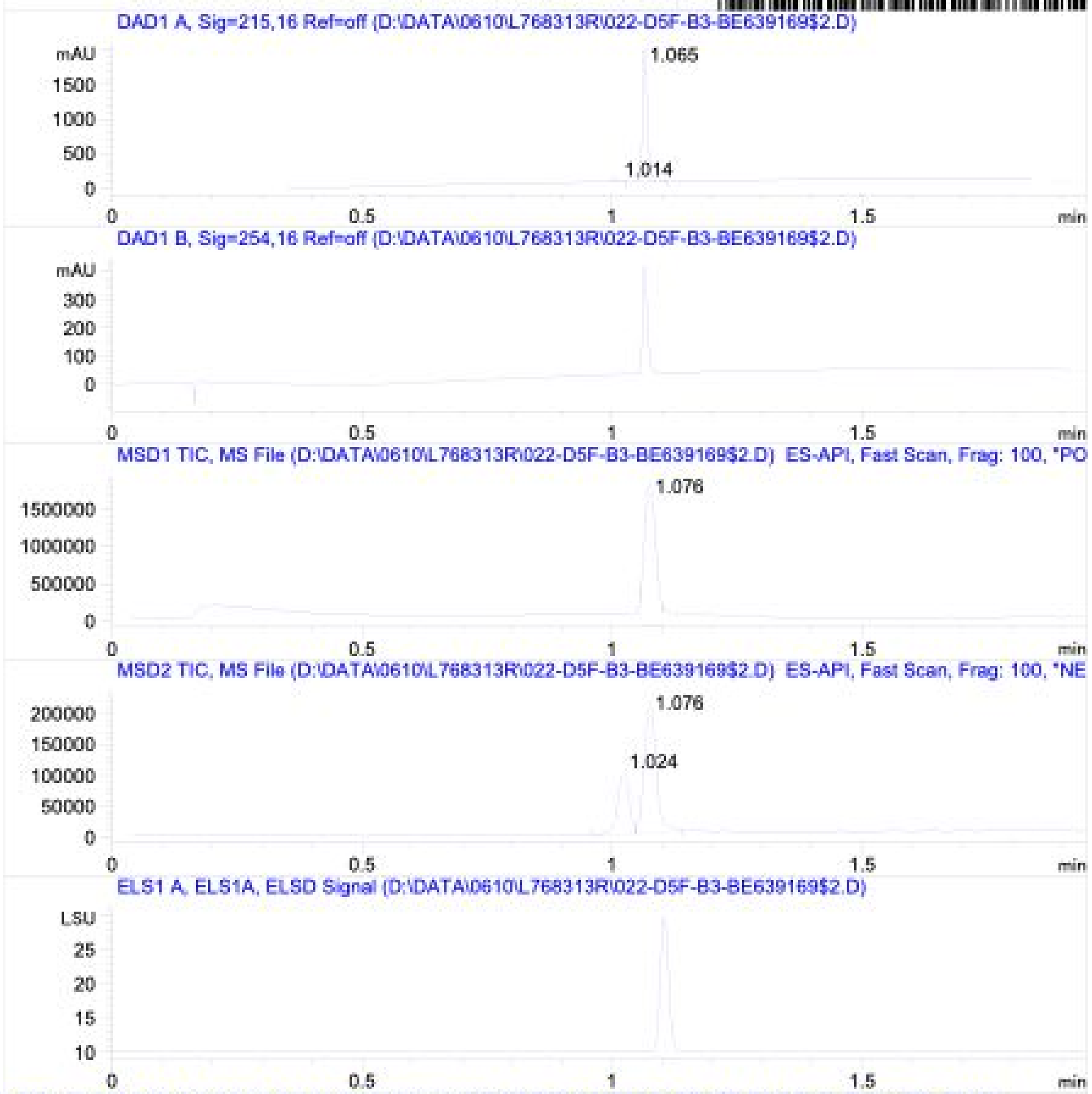

RT 1.076

RT 1.024

RT 1.076

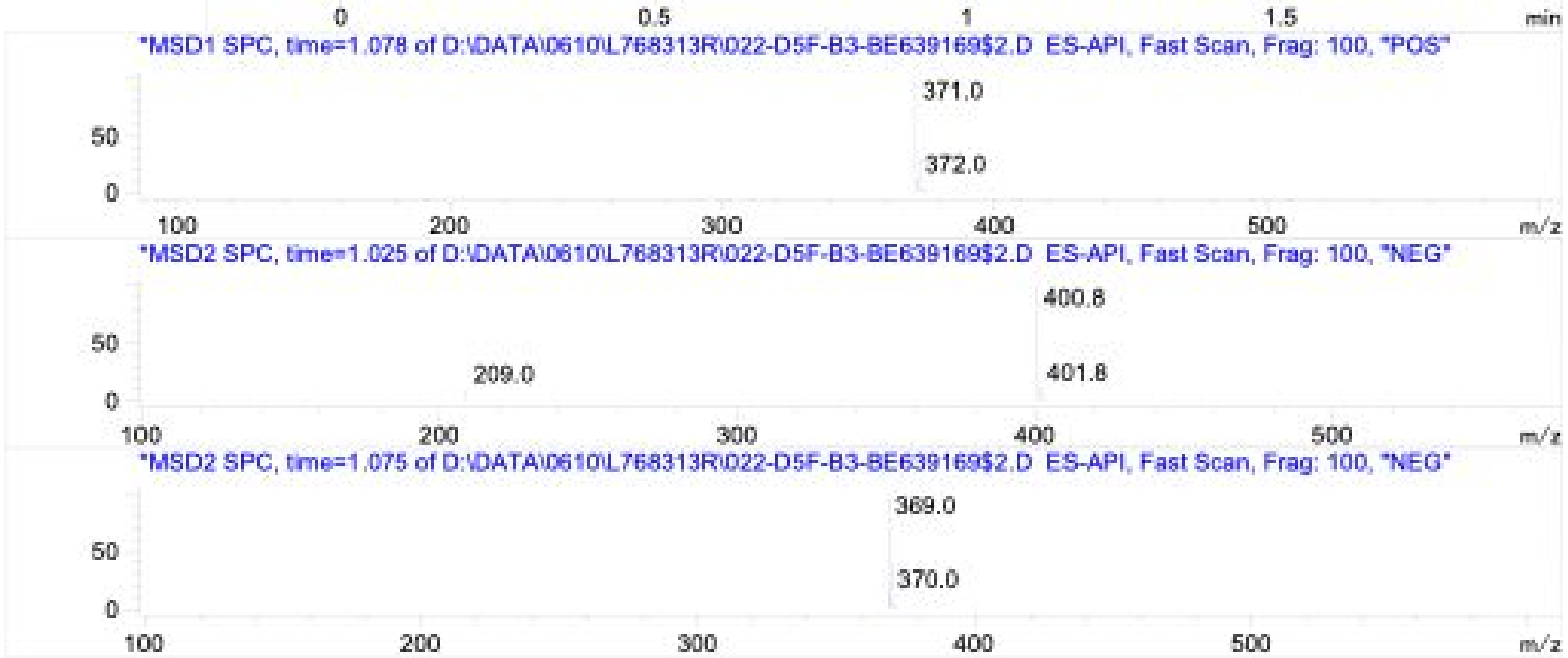

Inj.Date 6/10/2024 H 30
C:\Users\Public\Documents\ChemStation\1\Data\06_10\L768313R\SUPOR_30.M

Z9103952047

BE244429$3

MaxPeak: 100.00%
Ret_Time: 1.284 min

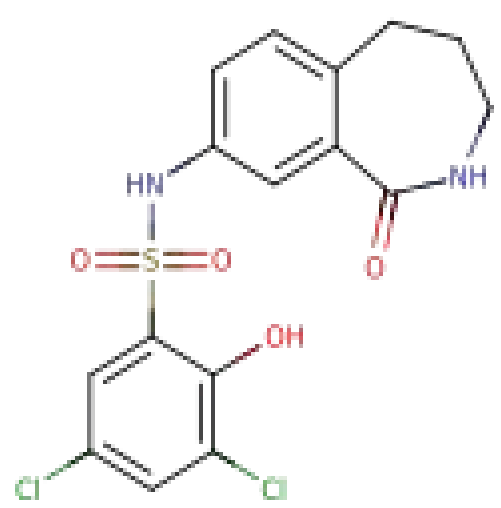

Mol Wt 401.26
Exact Mass 400.01

#	Time	Area%
1	1.284	100.00

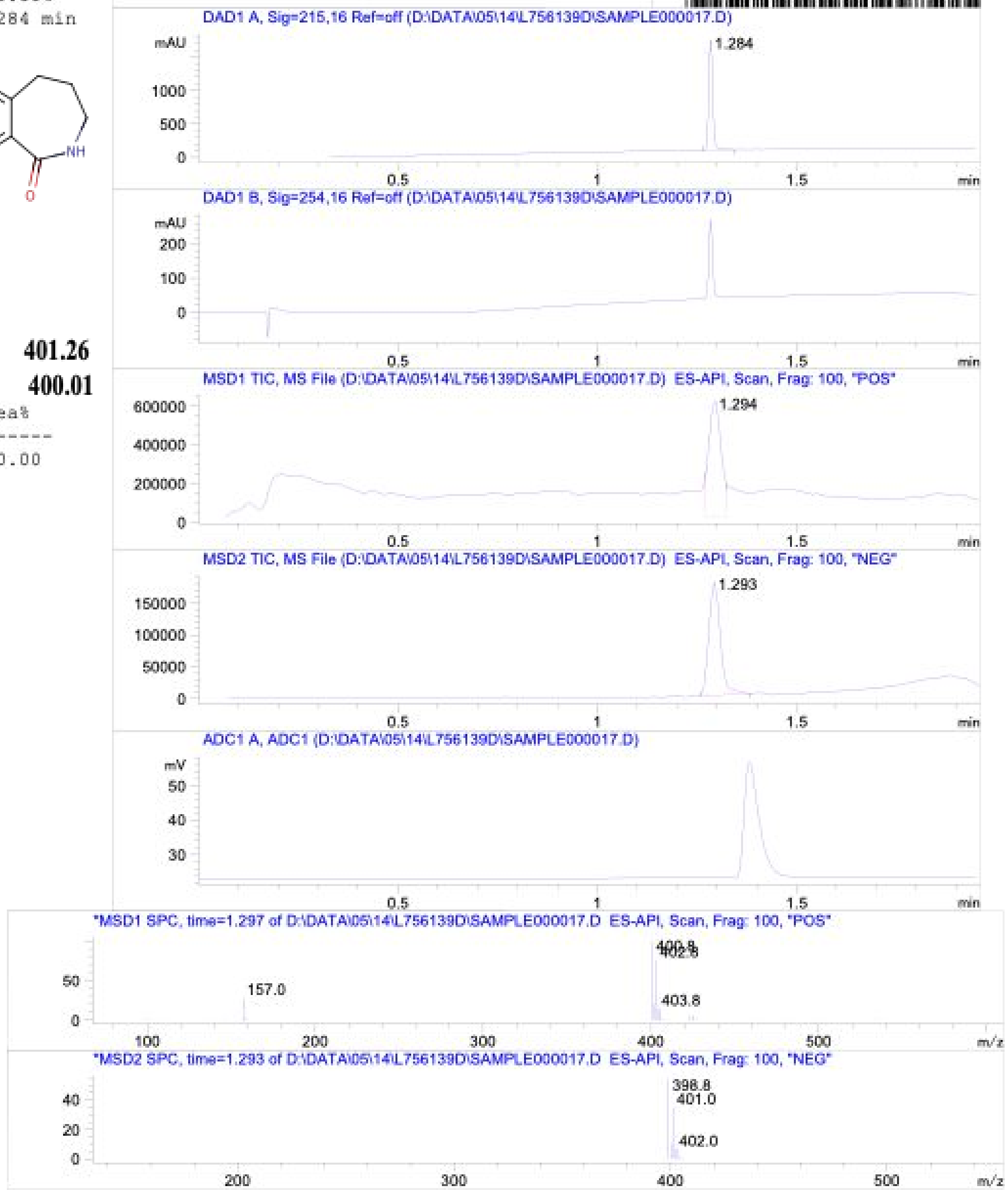

RT 1.294

RT 1.293

Inj.Date 5/13/2024 CH - 4 -
C:\CHEM32\1\METHODS\SUPOR_30.M

Z9103952128

MaxPeak: 97.26%
Ret_Time: 1.180 min

BE244431$2

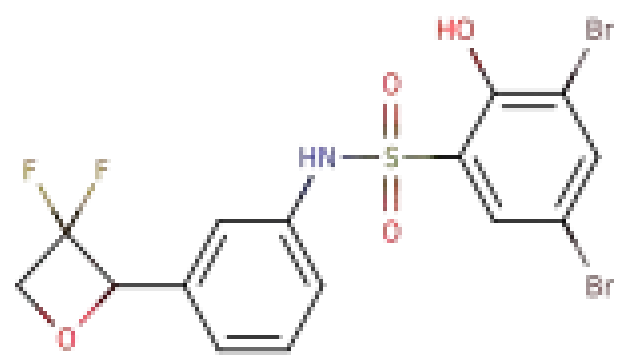

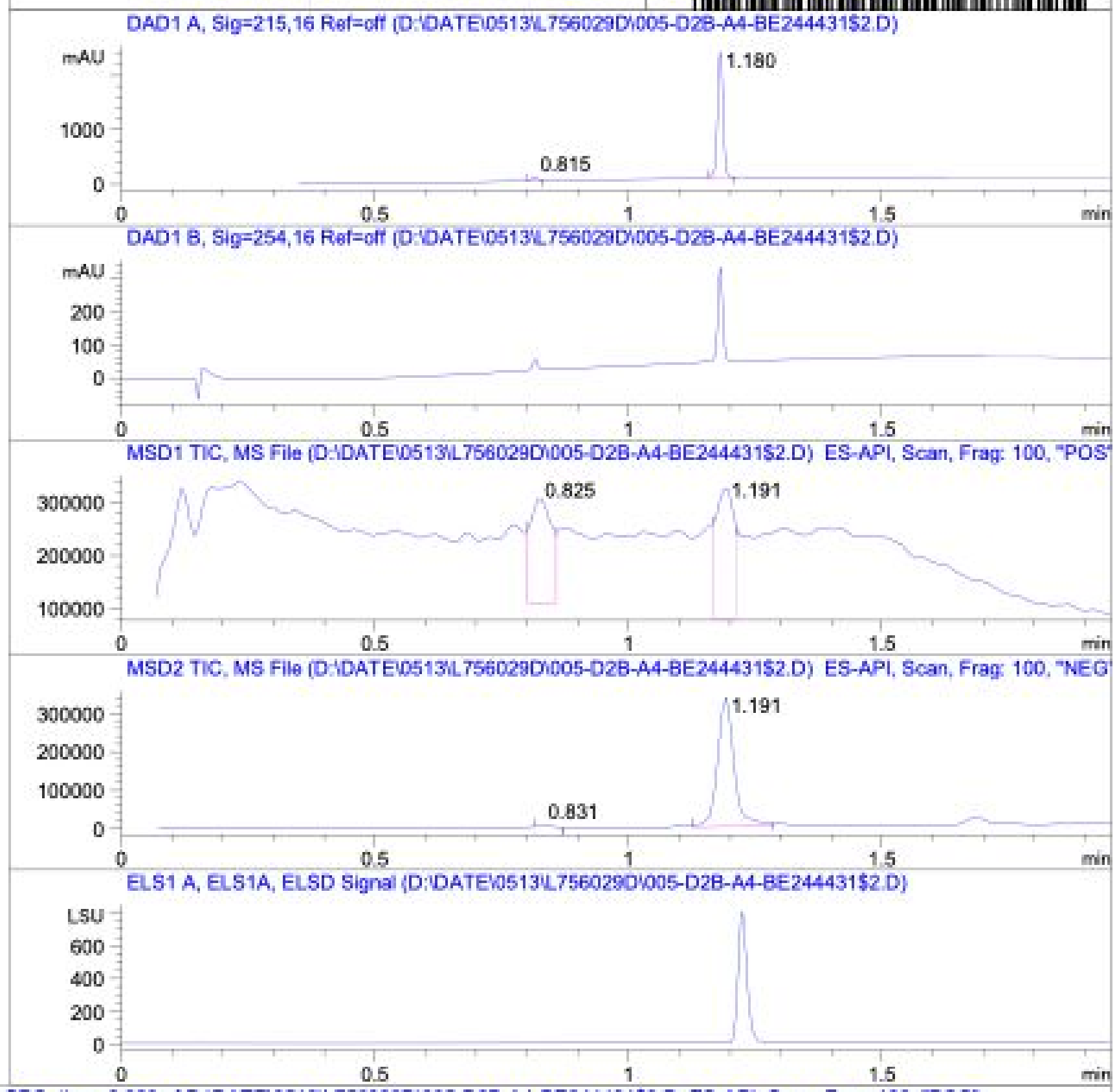

Mol Wt 499.12

Exact Mass 498.88

#	Time	Area%
1	0.815	2.74
2	1.180	97.26

RT 0.825

RT 1.191

RT 0.831

RT 1.191

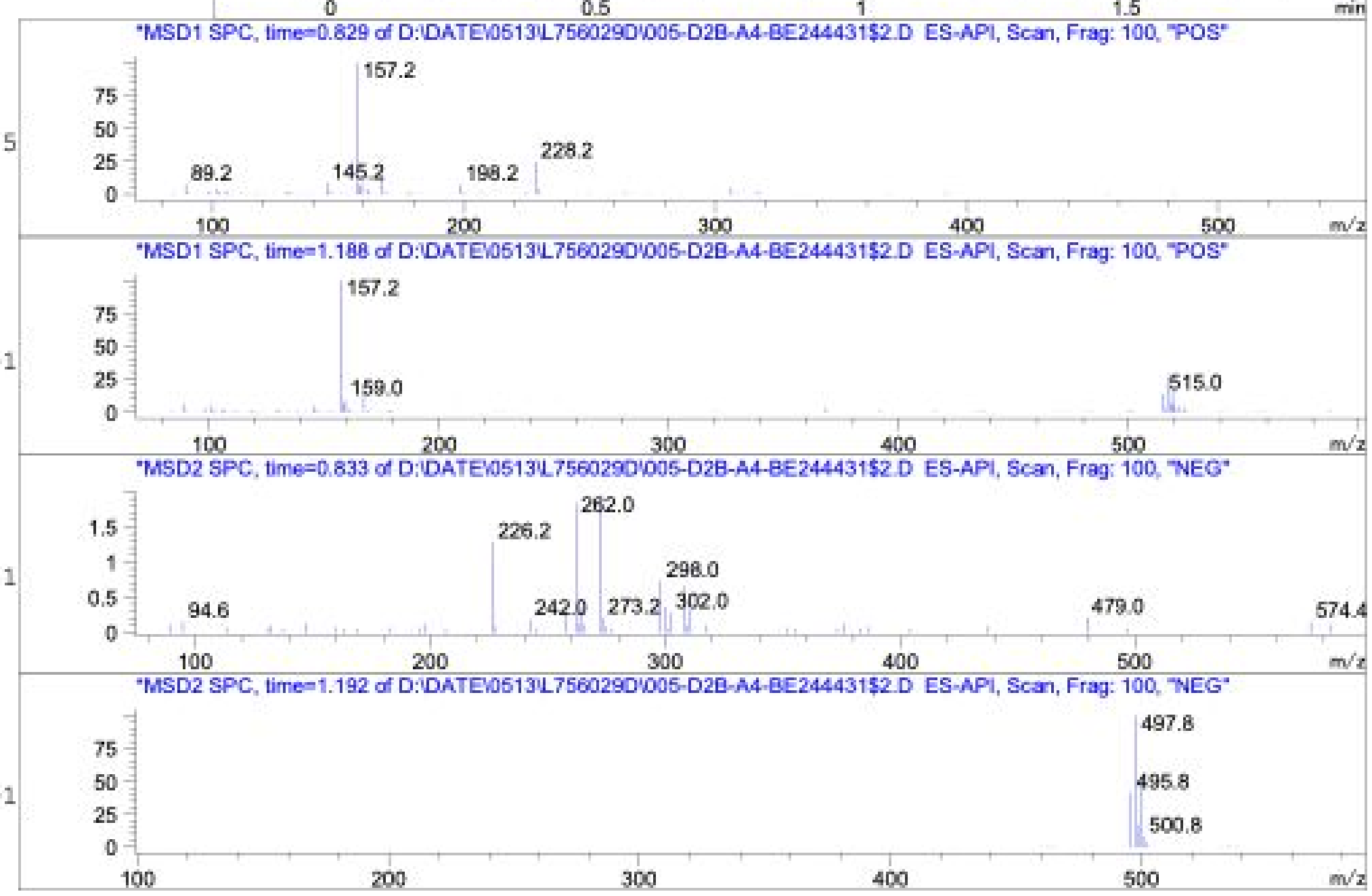

Inj.Date 5/13/2024 CH 13

C:\Chem32\1\Data\05_13\L756029D\SUPOR_96.M

Z9103952148

MaxPeak: 100.00%
Ret_Time: 1.276 min

BE244432$3

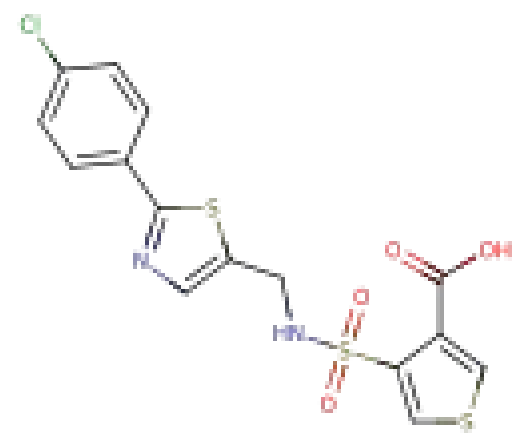

Mol Wt 414.91
Exact Mass 413.95

#	Time	Area%
1	1.276	100.00

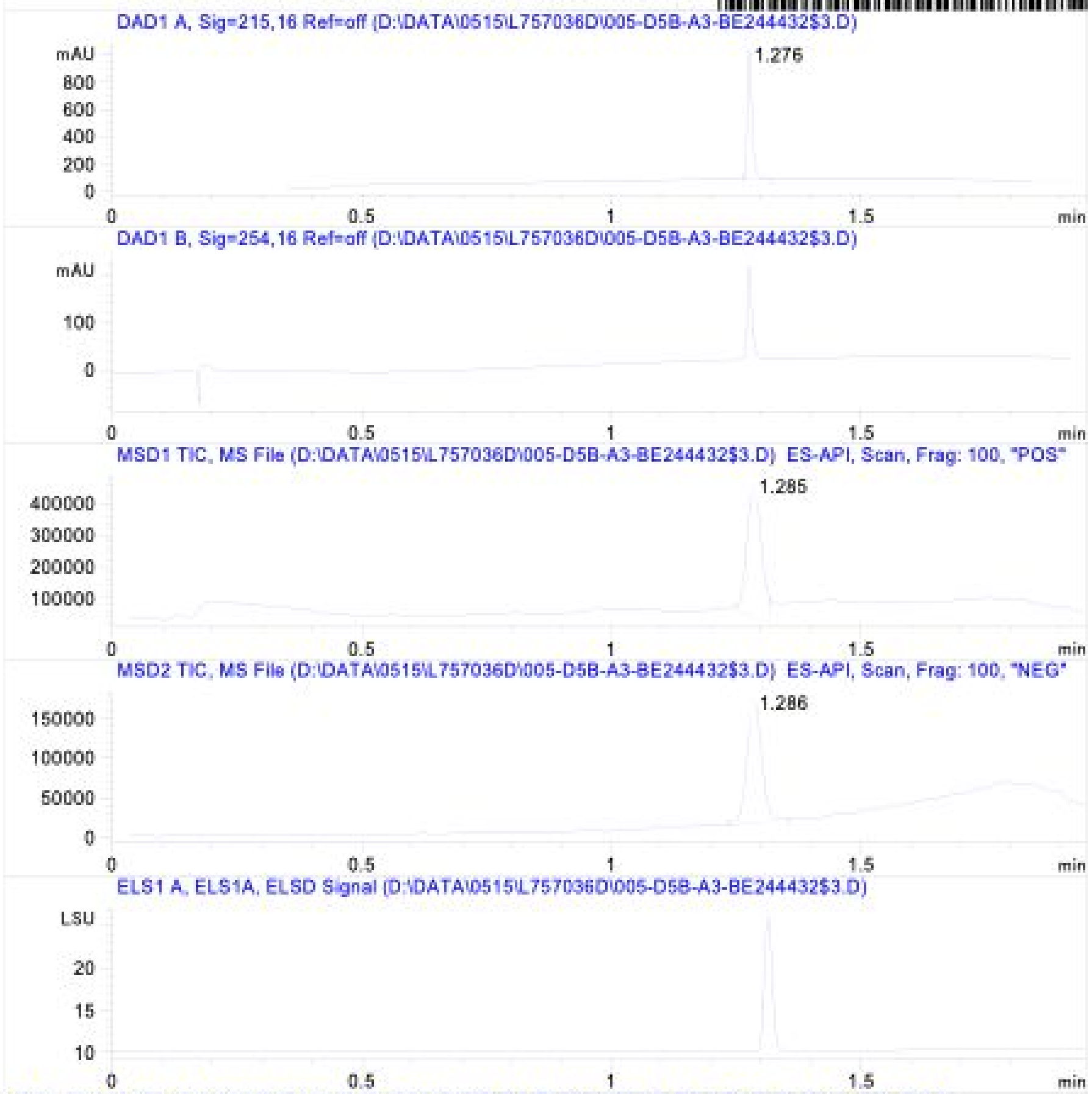

RT 1.285

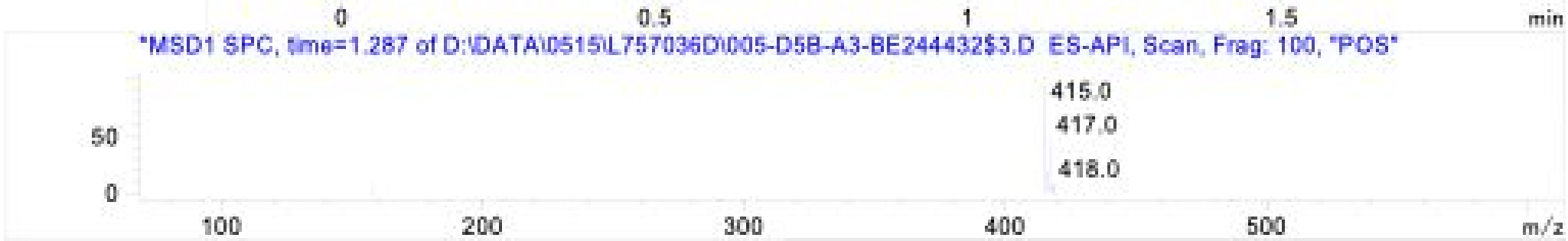

RT 1.286

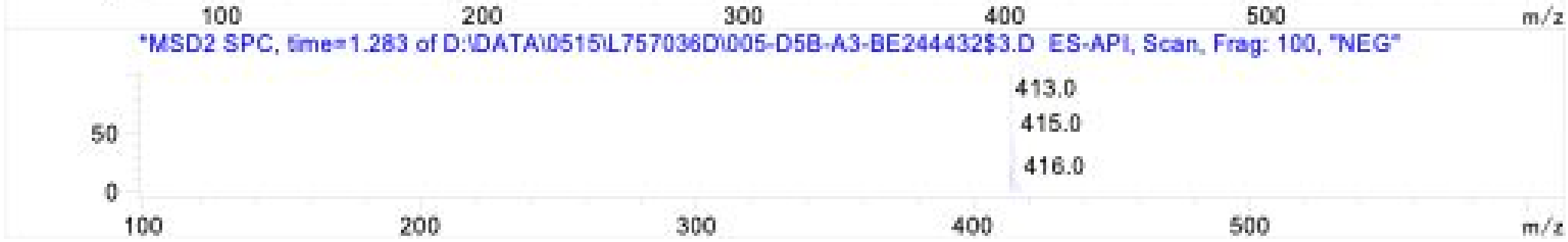

Inj.Date 5/14/2024 H -25-
C:\Users\Public\Documents\ChemStation\1\Data\05_14\L757036D\SUPOR_30.M

Z9103952158

MaxPeak: 98.87%
Ret_Time: 1.506 min

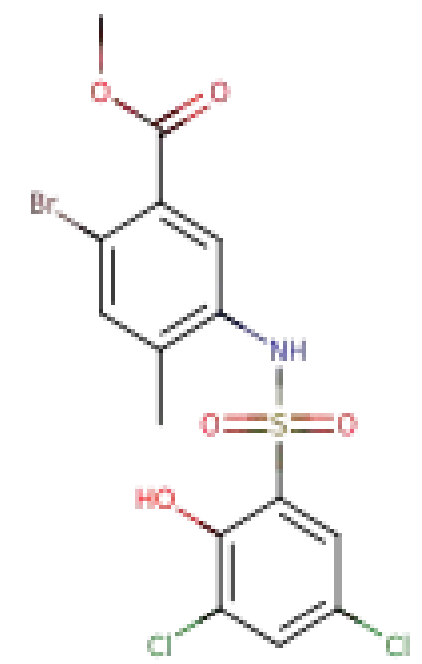

Mol Wt 469.13
Exact Mass 468.9

#	Time	Area%
1	1.220	1.13
2	1.506	98.87

BE244433$1

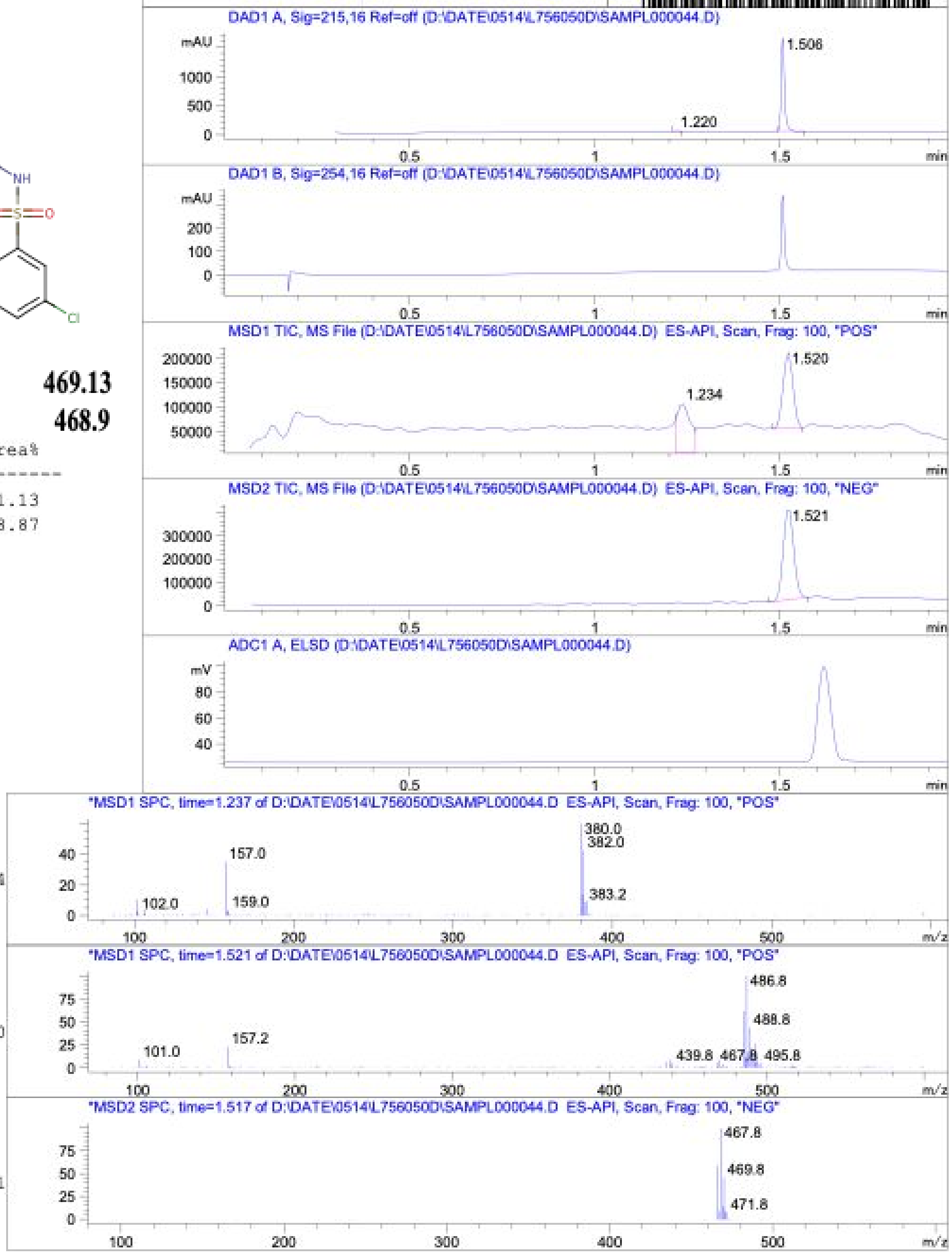

RT 1.234

RT 1.520

RT 1.521

Inj.Date 5/13/2024 CH -6-
C:\CHEM32\1\METHODS\SUPOR_30.M

Z9103952171

MaxPeak: 95.81%
Ret_Time: 1.332 min

BE244435$2

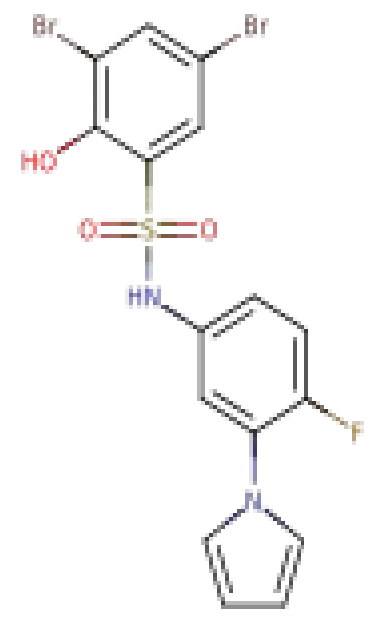

Mol Wt 490.14

Exact Mass 489.89

#	Time	Area%
1	1.021	2.79
2	1.332	95.81
3	1.379	1.40

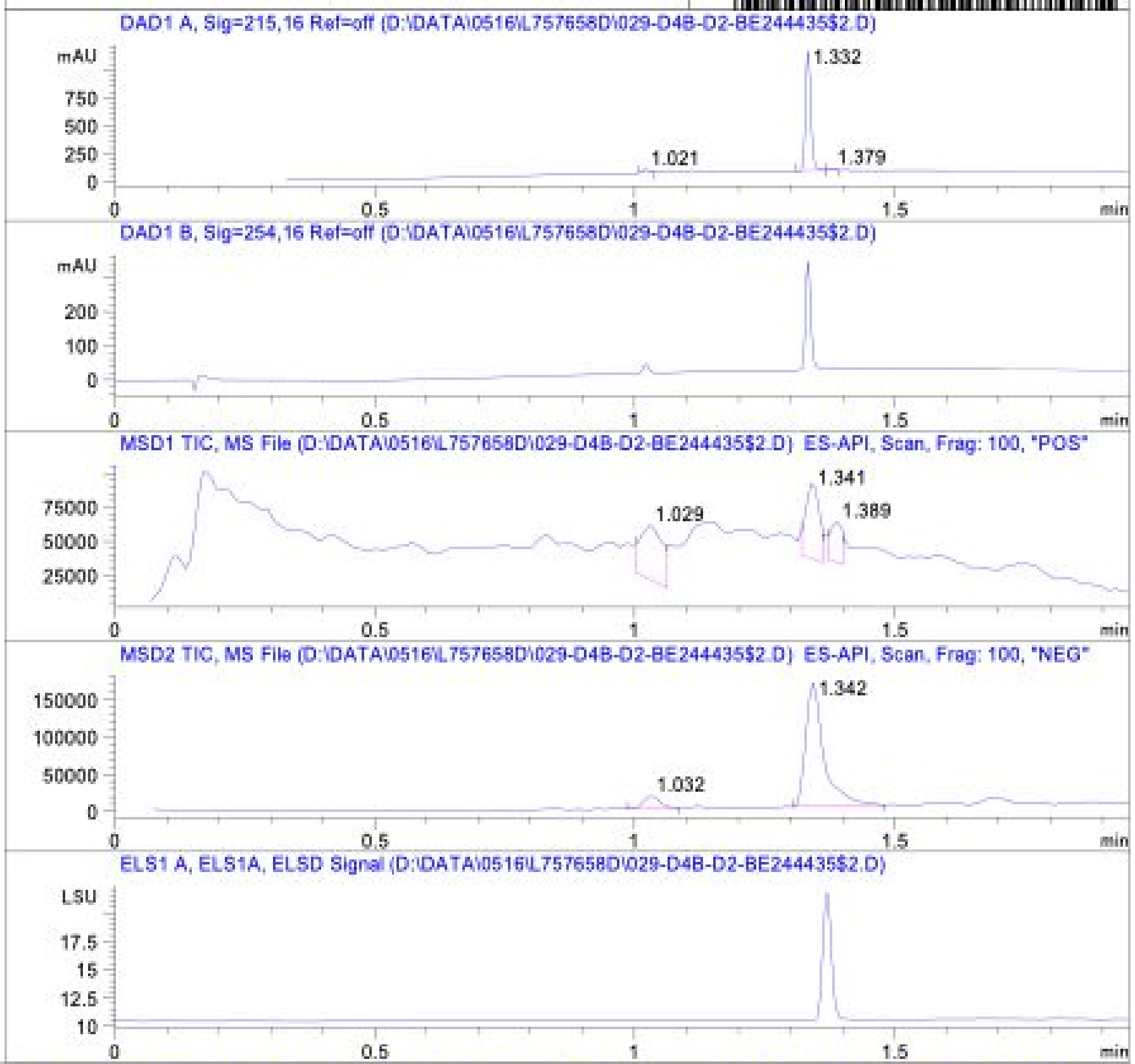

RT 1.029

RT 1.341

RT 1.389

RT 1.032

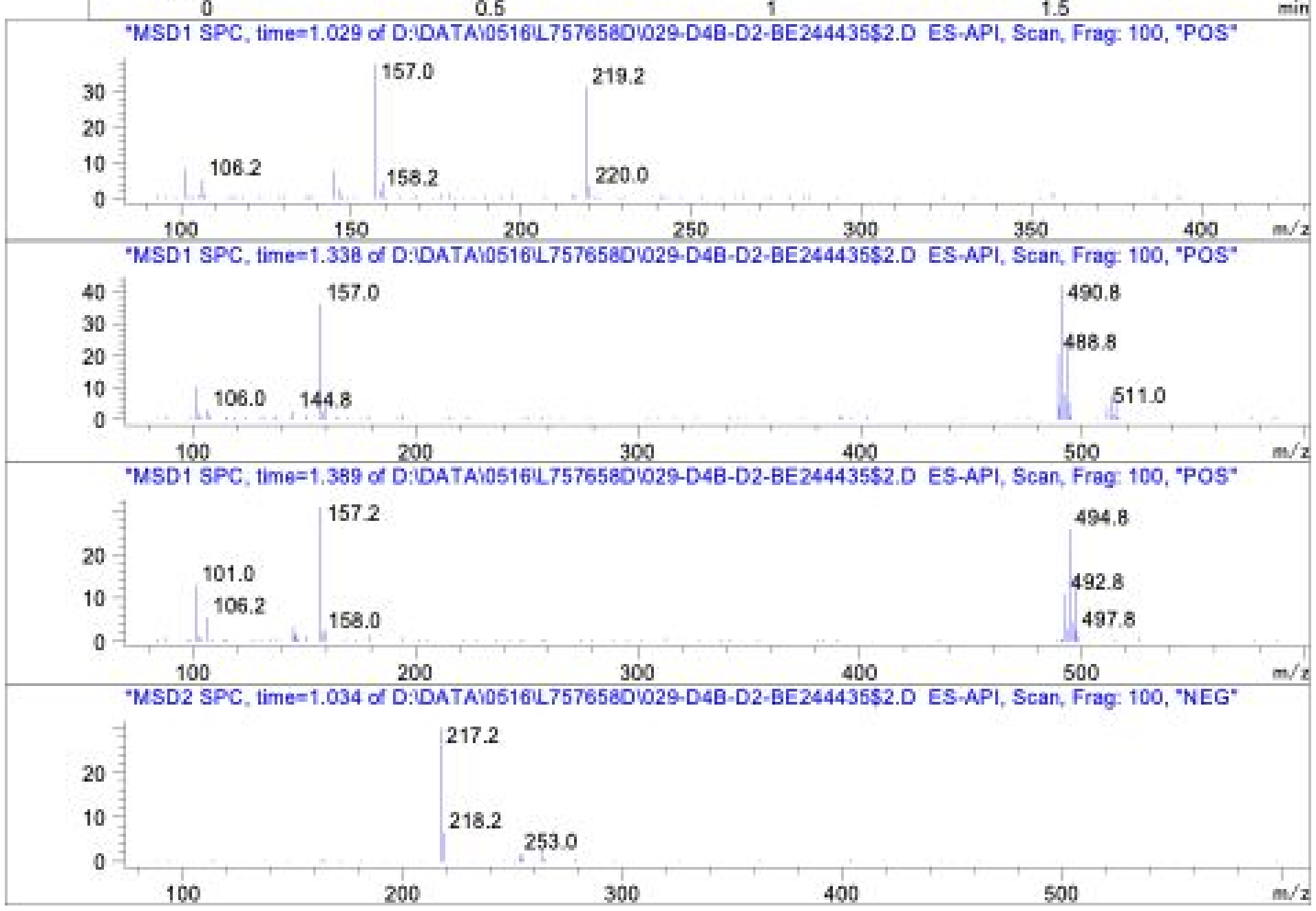

Inj.Date 5/15/2024 LB -14-
C:\Chem32\1\Data\05_15\L757658D\SUPOR_30.M

Z9103952184

MaxPeak: 100.00%
Ret_Time: 1.449 min

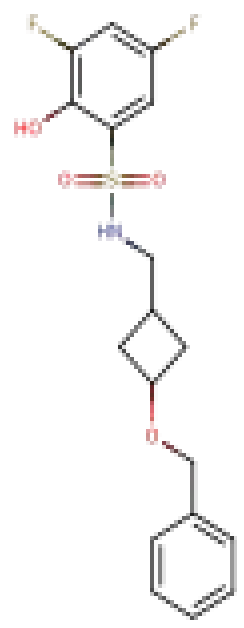

Mol Wt 383.41
Exact Mass 383.12

#	Time	Area%
1	1.449	100.00

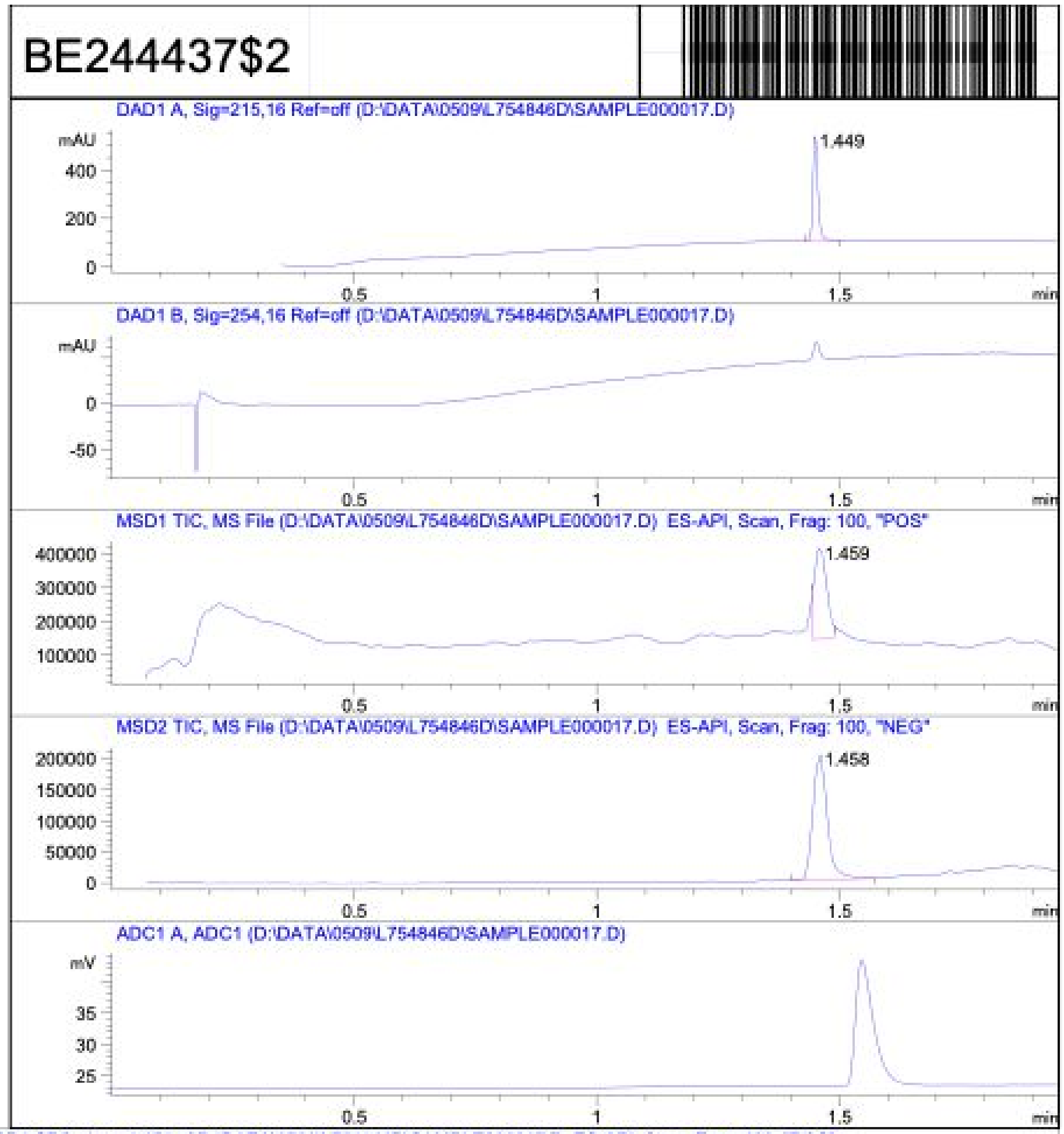

RT 1.459

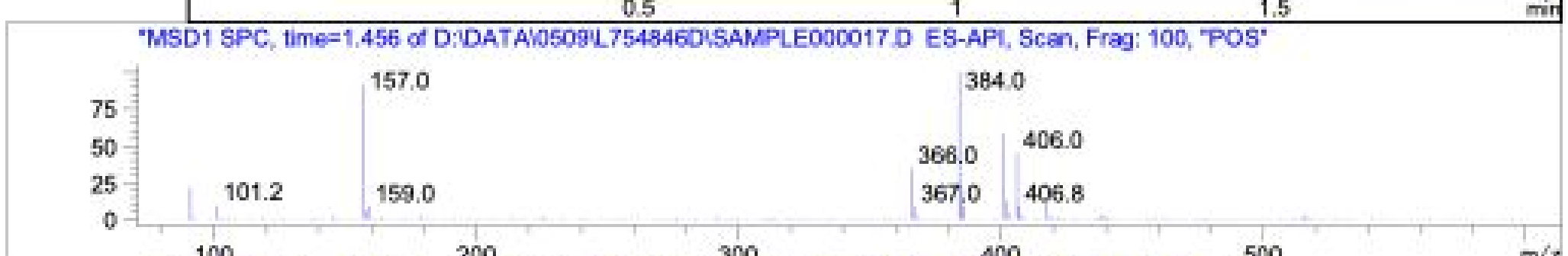

RT 1.458

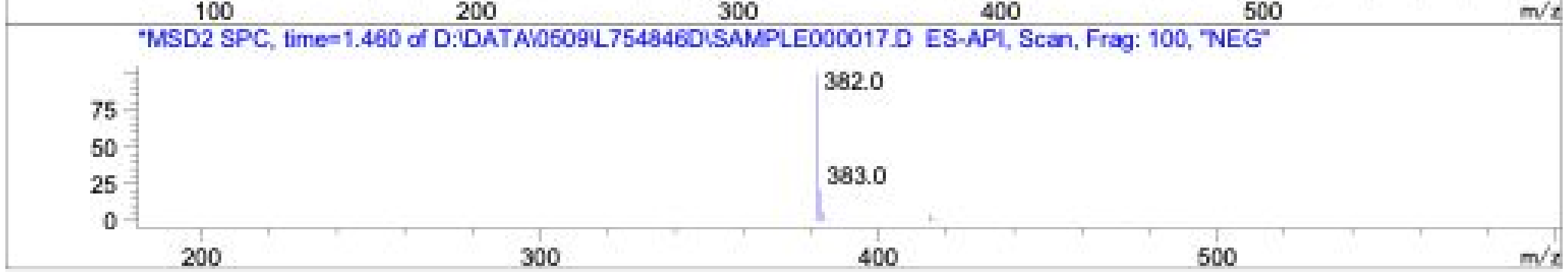

Inj.Date 5/9/2024 VB - 4 -
C:\CHEM32\1\METHODS\SUPOR_30.M

Z9103952204

MaxPeak: 98.61%
Ret_Time: 1.346 min

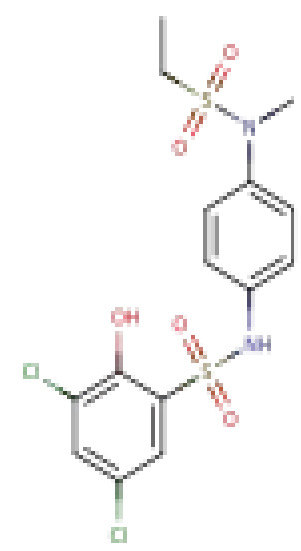

Mol Wt 439.33

Exact Mass 437.99

#	Time	Area%
1	1.346	98.61
2	1.413	1.39

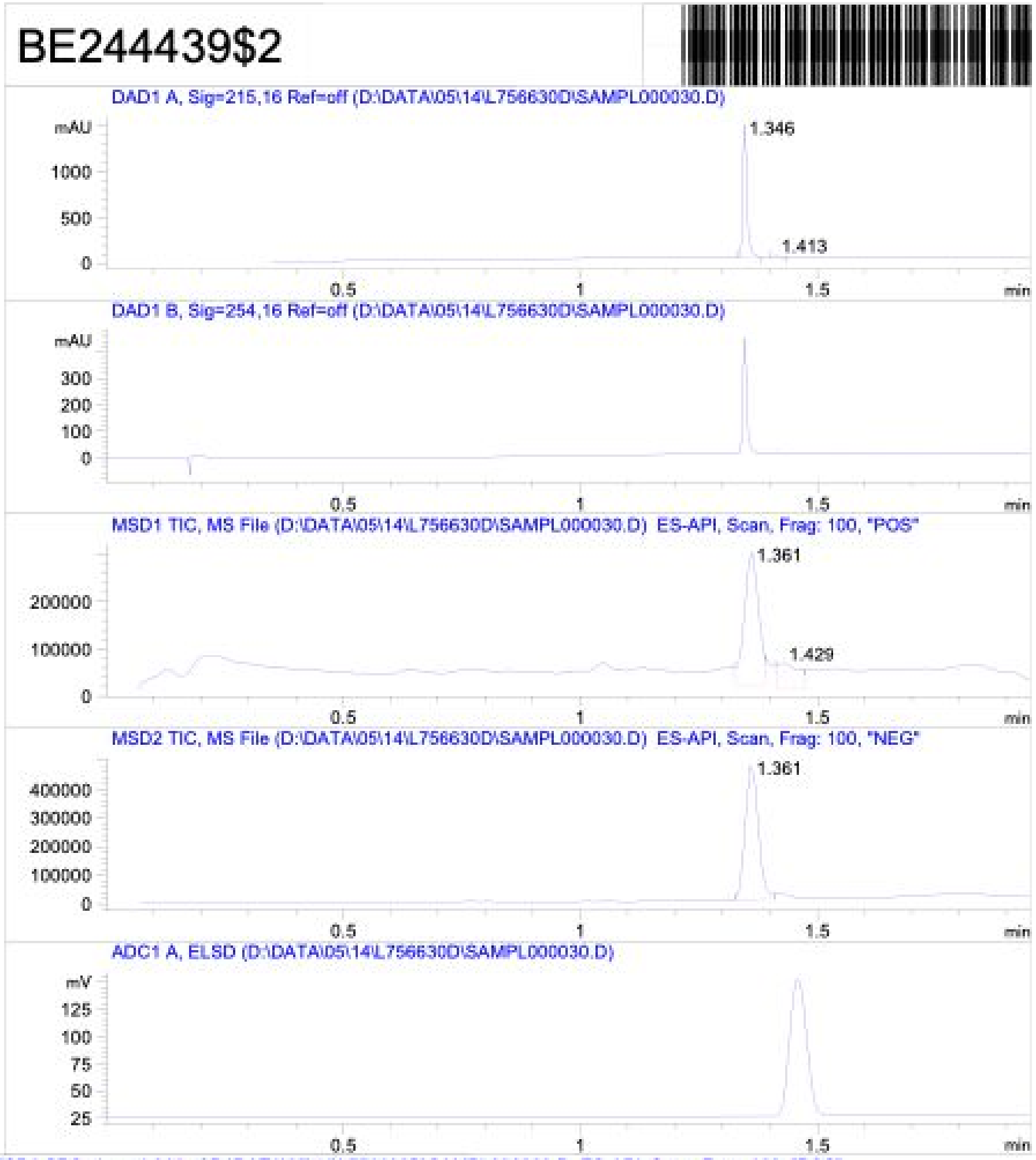

RT 1.361

RT 1.429

RT 1.361

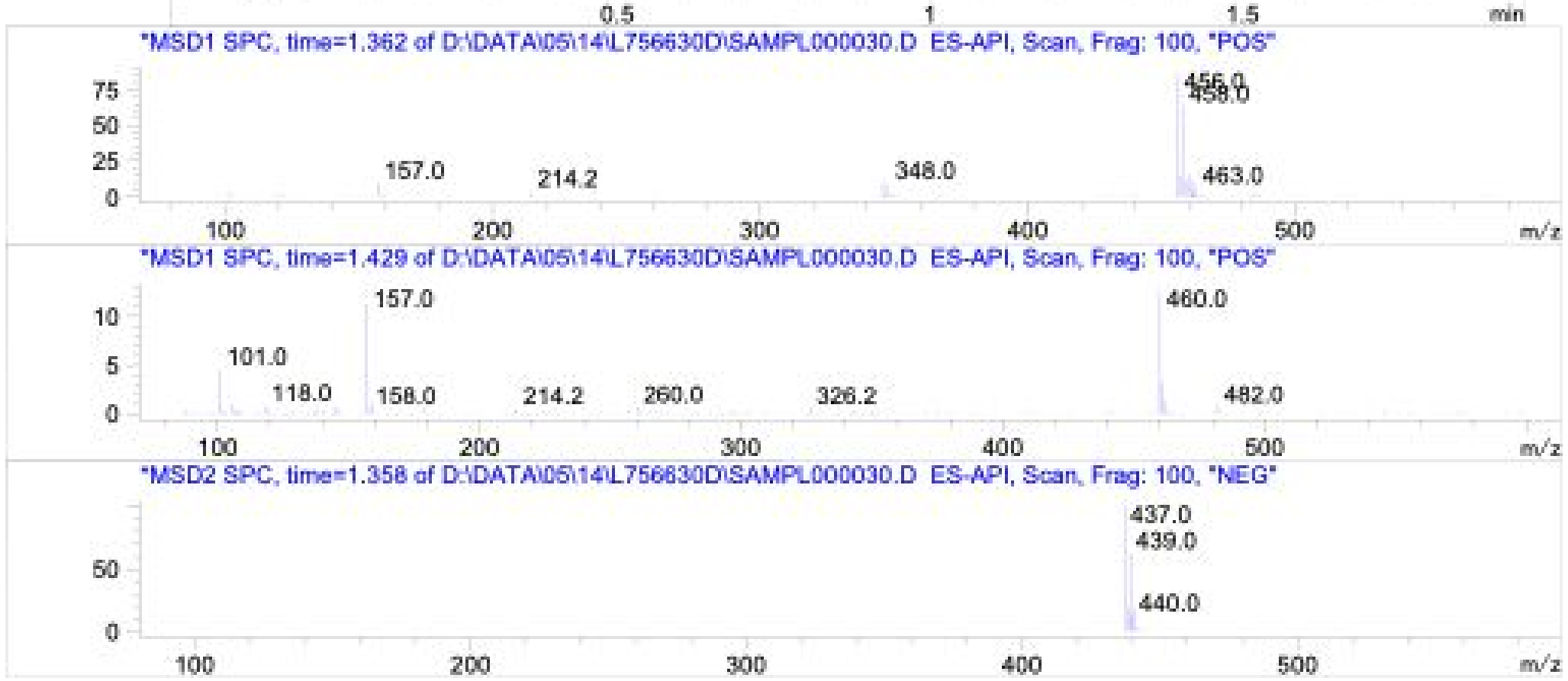

Inj.Date 5/14/2024 CH -6-
C:\CHEM32\1\METHODS\SUPOR_30.M

Z9103952229

MaxPeak: 92.42%
Ret_Time: 1.502 min

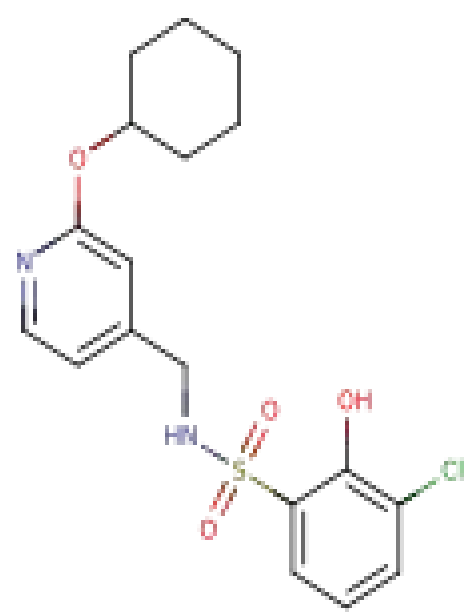

Mol Wt 396.89
Exact Mass 396.11

#	Time	Area%
1	1.030	2.22
2	1.404	3.86
3	1.502	92.42
4	1.684	1.50

BE244440$2

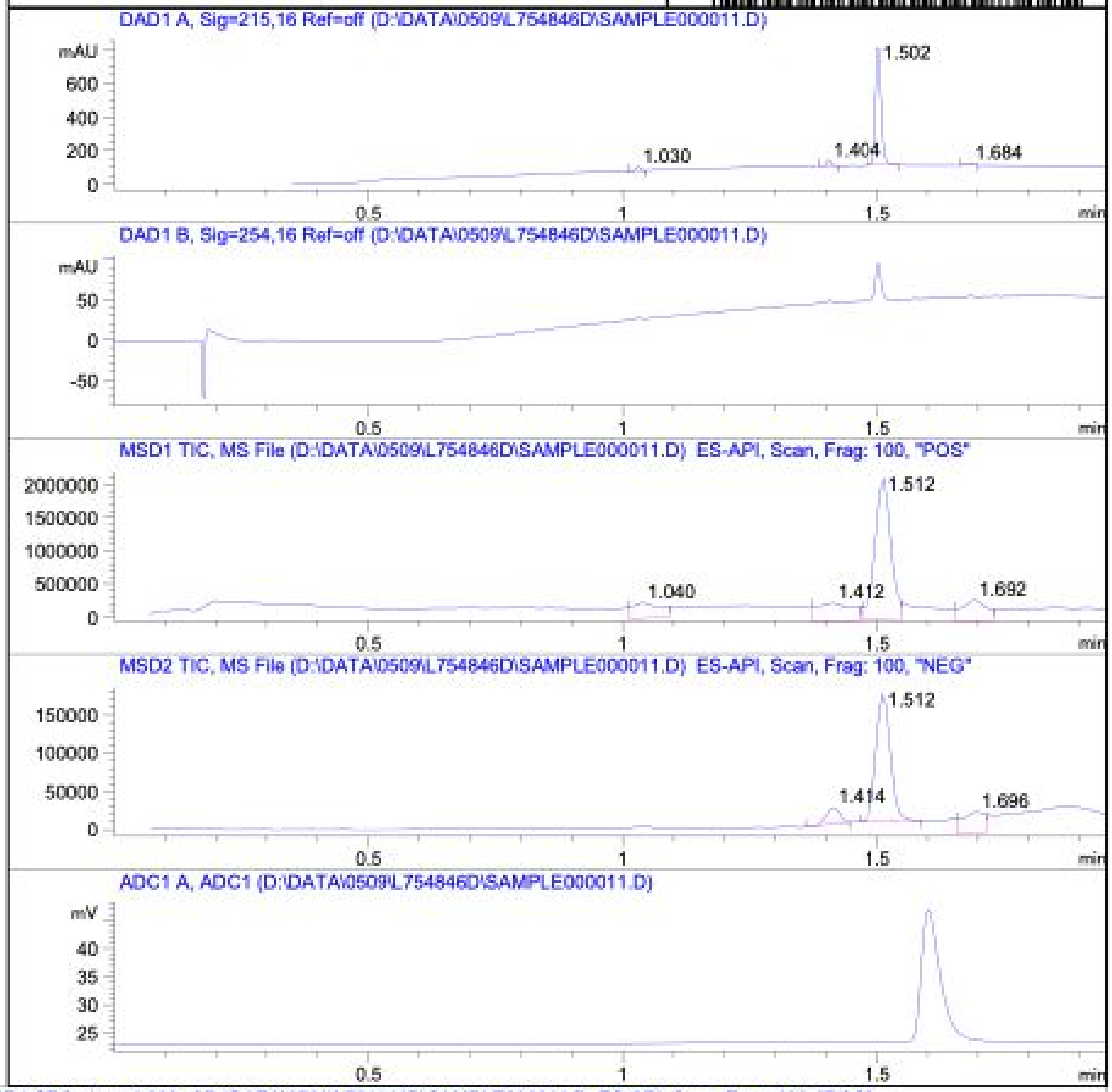

RT 1.040

RT 1.412

RT 1.512

RT 1.692

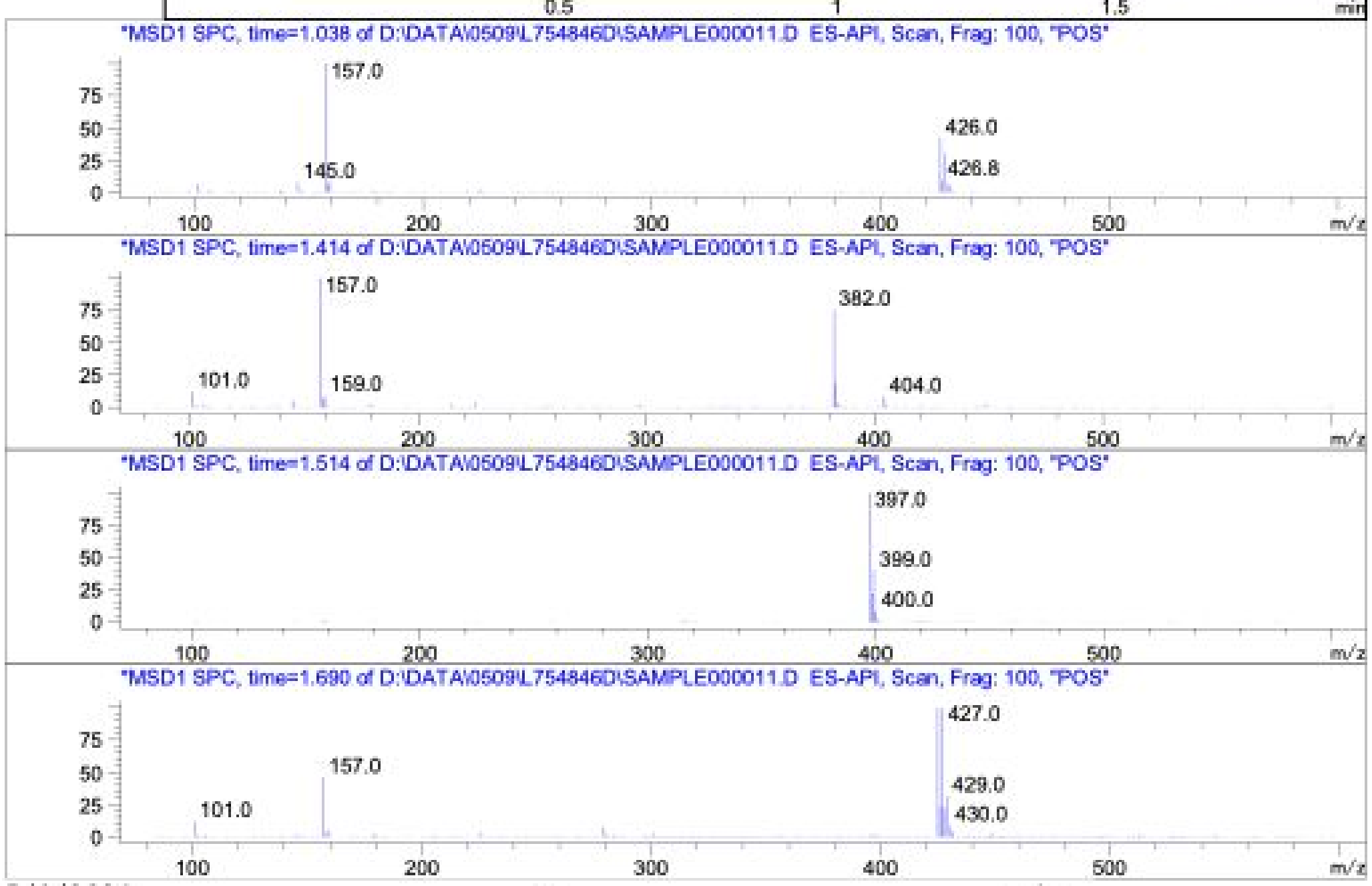

Inj.Date 5/9/2024 VB - 4 -
C:\CHEM32\1\METHODS\SUPOR_30.M

Z9103952235

MaxPeak: 100.00%
Ret_Time: 1.528 min

BE404171$1

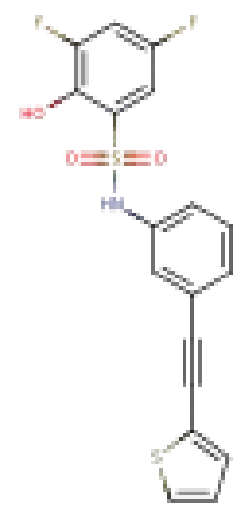

Mol Wt 391.41

Exact Mass 391.02

#	Time	Area%
1	1.528	100.00

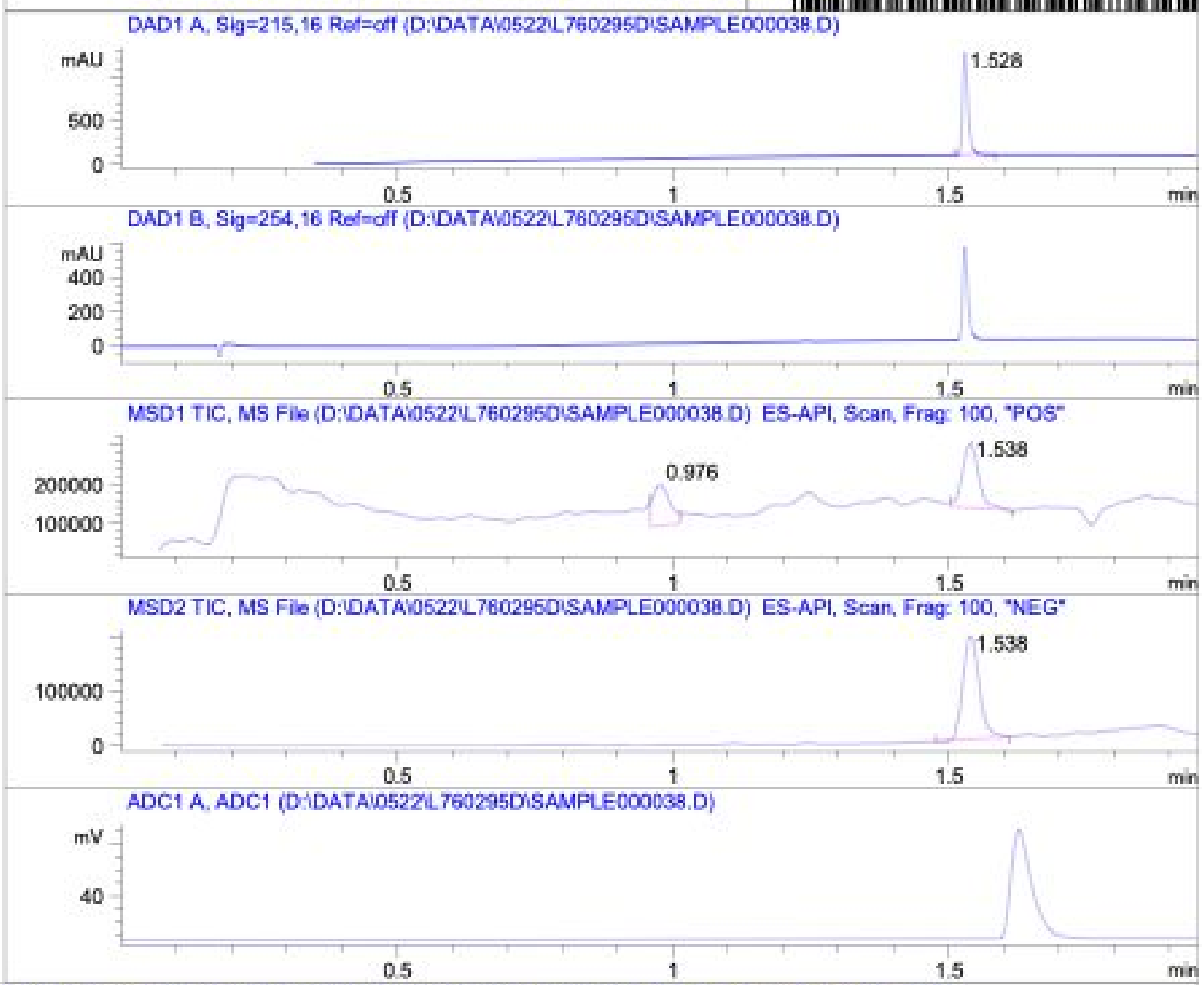

RT 0.976

RT 1.538

RT 1.538

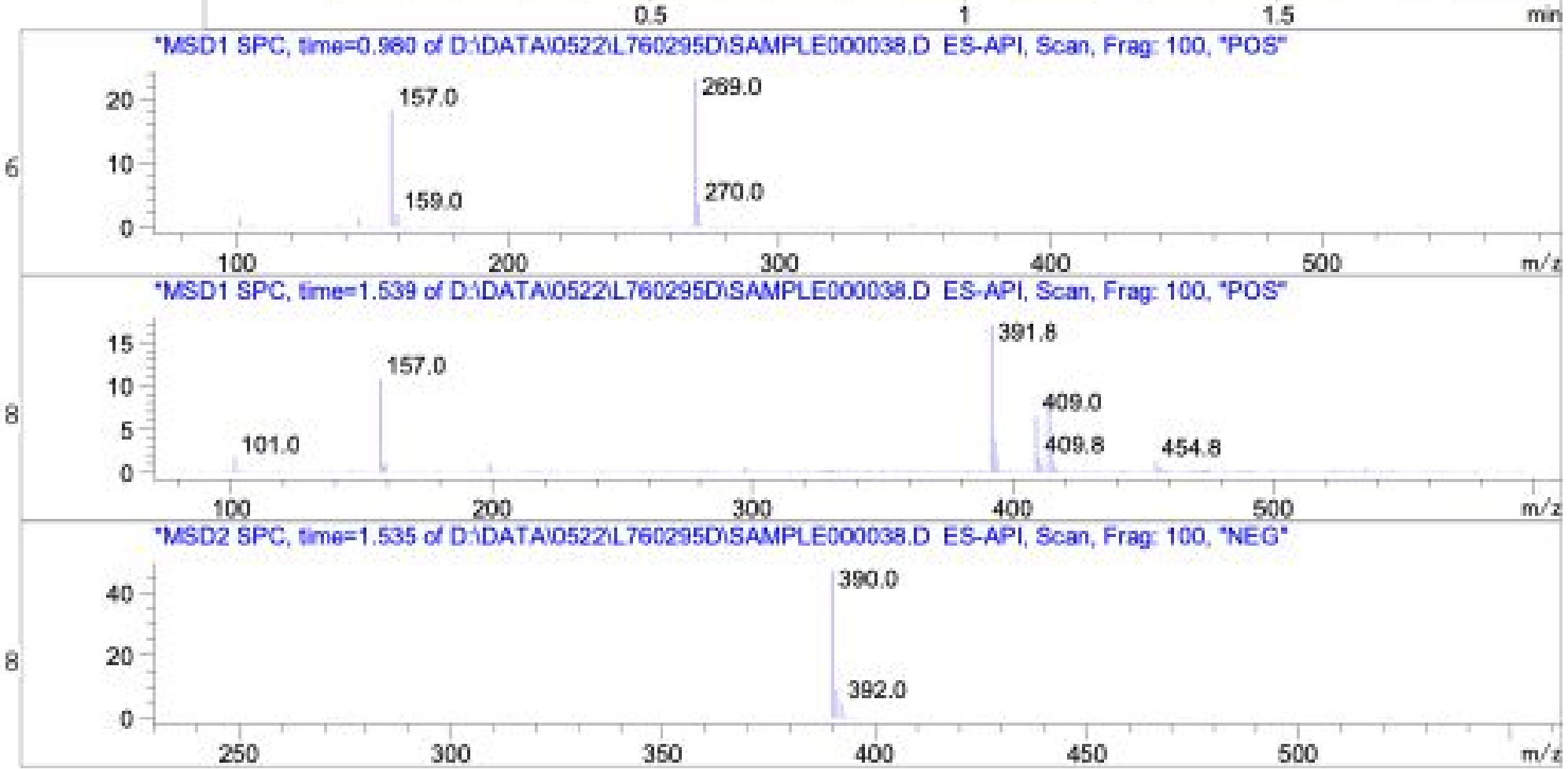

Inj.Date 5/22/2024 - 4 - M
C:\CHEM32\1\METHODS\SUPOR_30.M

Z9103952237

MaxPeak: 100.00%
Ret_Time: 1.204 min

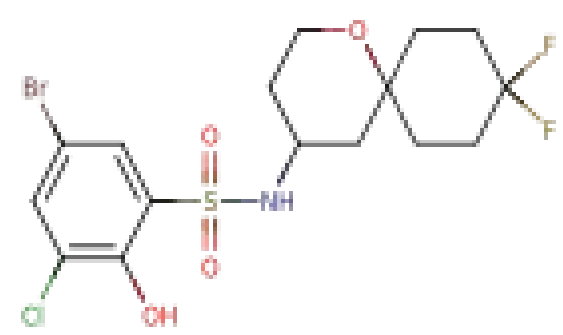

Mol Wt 474.74

Exact Mass 475.01

#	Time	Area%
1	1.204	100.00

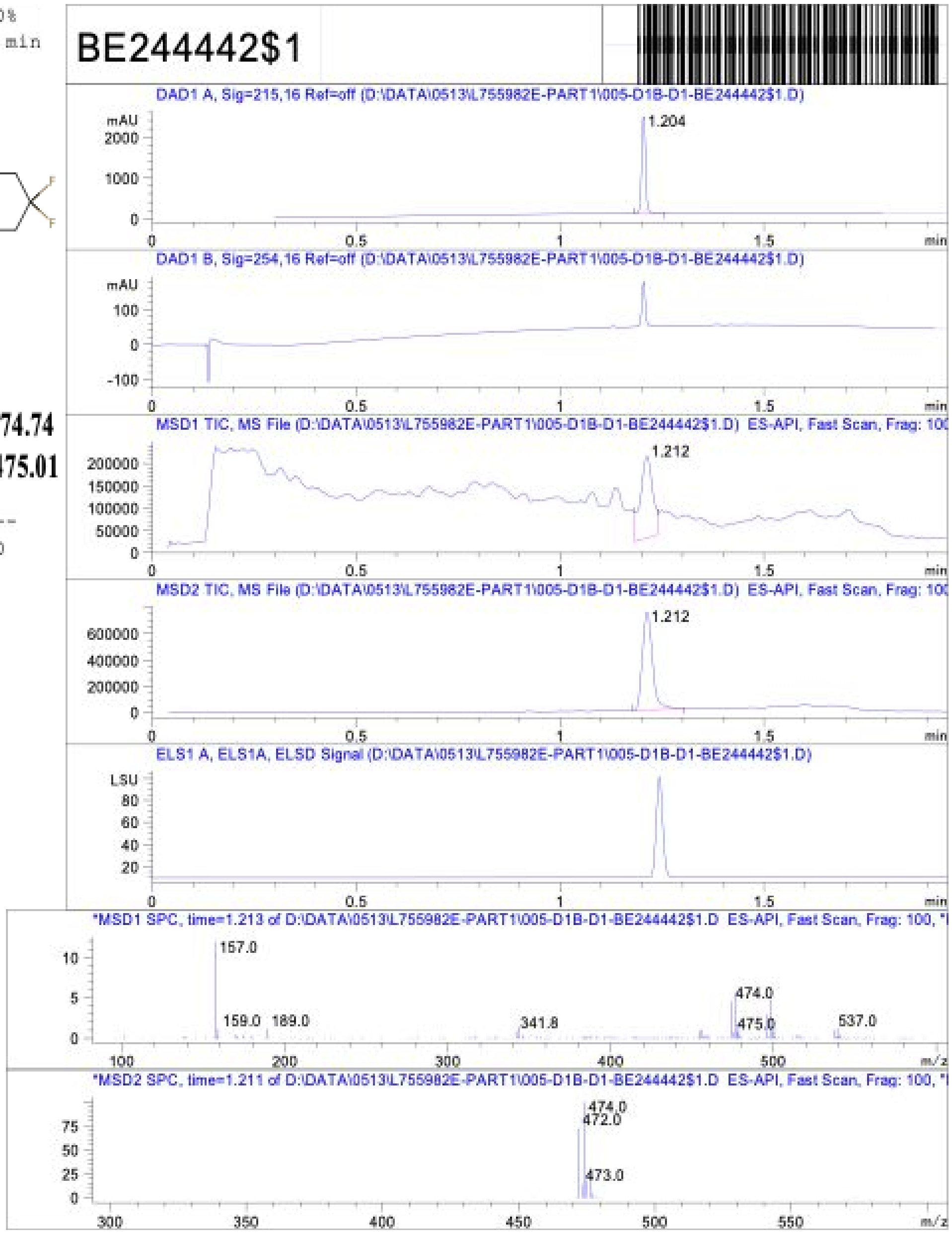

RT 1.212

RT 1.212

Inj.Date 5/13/2024 LB

C:\Users\Public\Documents\ChemStation\1\Data\05_13\1755982E\SUPOR_96.M

Z9103952244

MaxPeak: 100.00%
Ret_Time: 1.412 min

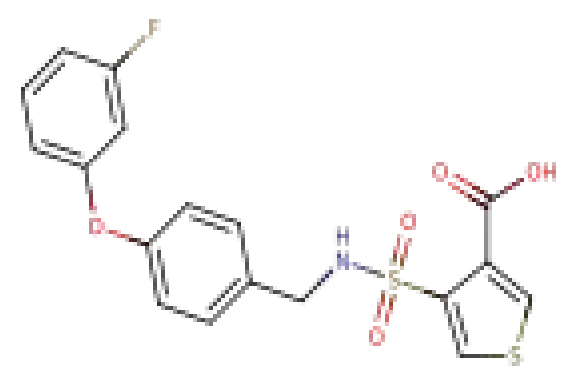

Mol Wt 407.44

Exact Mass 407.03

#	Time	Area%
1	1.412	100.00

RT 1.425

RT 1.427

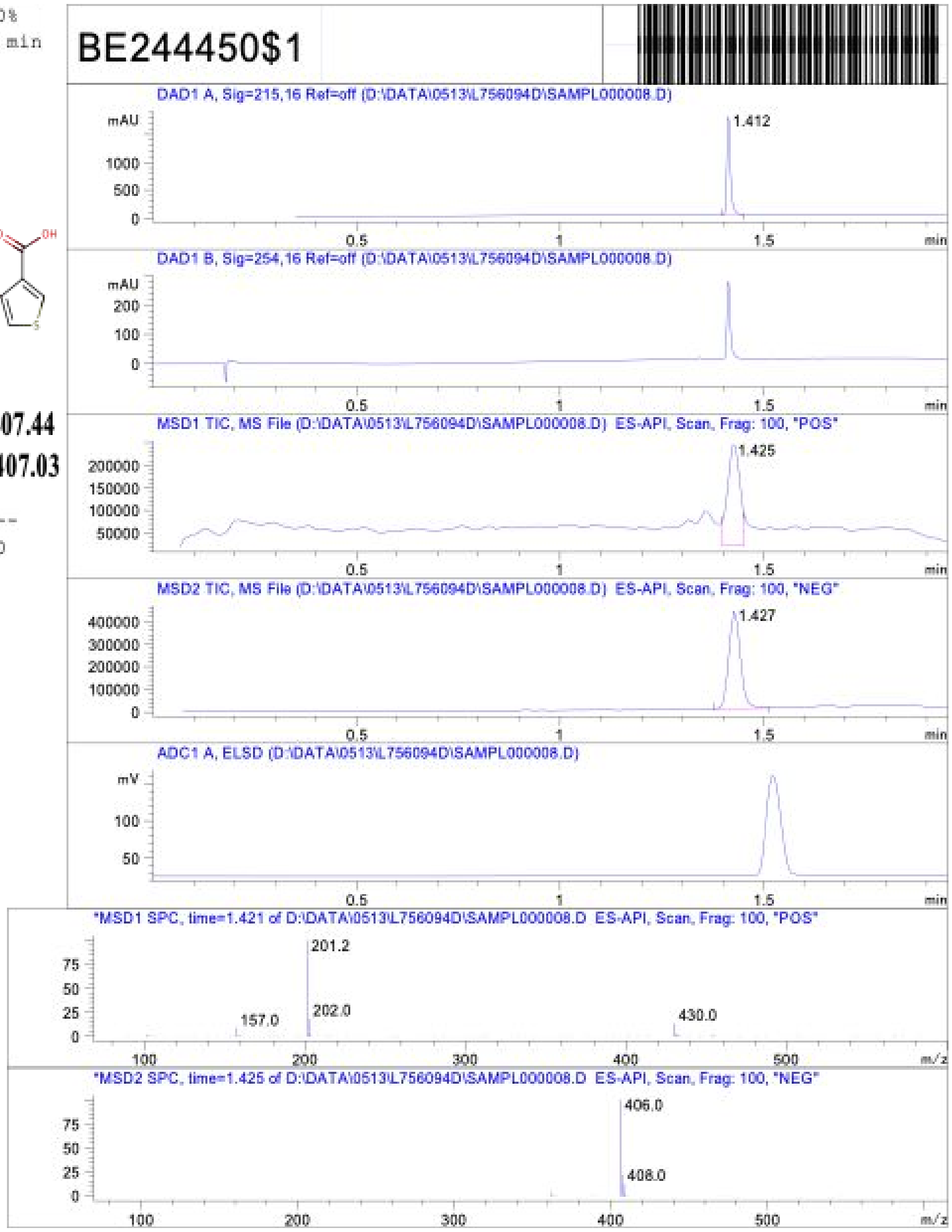

Inj.Date 5/13/2024 LB -6-
C:\CHEM32\1\METHODS\SUPOR_30.M

Z9103952299

MaxPeak: 91.02%
Ret_Time: 1.352 min

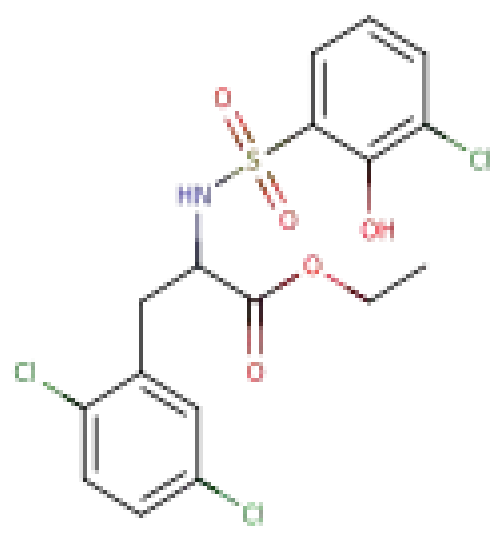

Mol Wt 452.74
Exact Mass 452.99

#	Time	Area%
1	1.314	8.98
2	1.352	91.02

BE244451$4

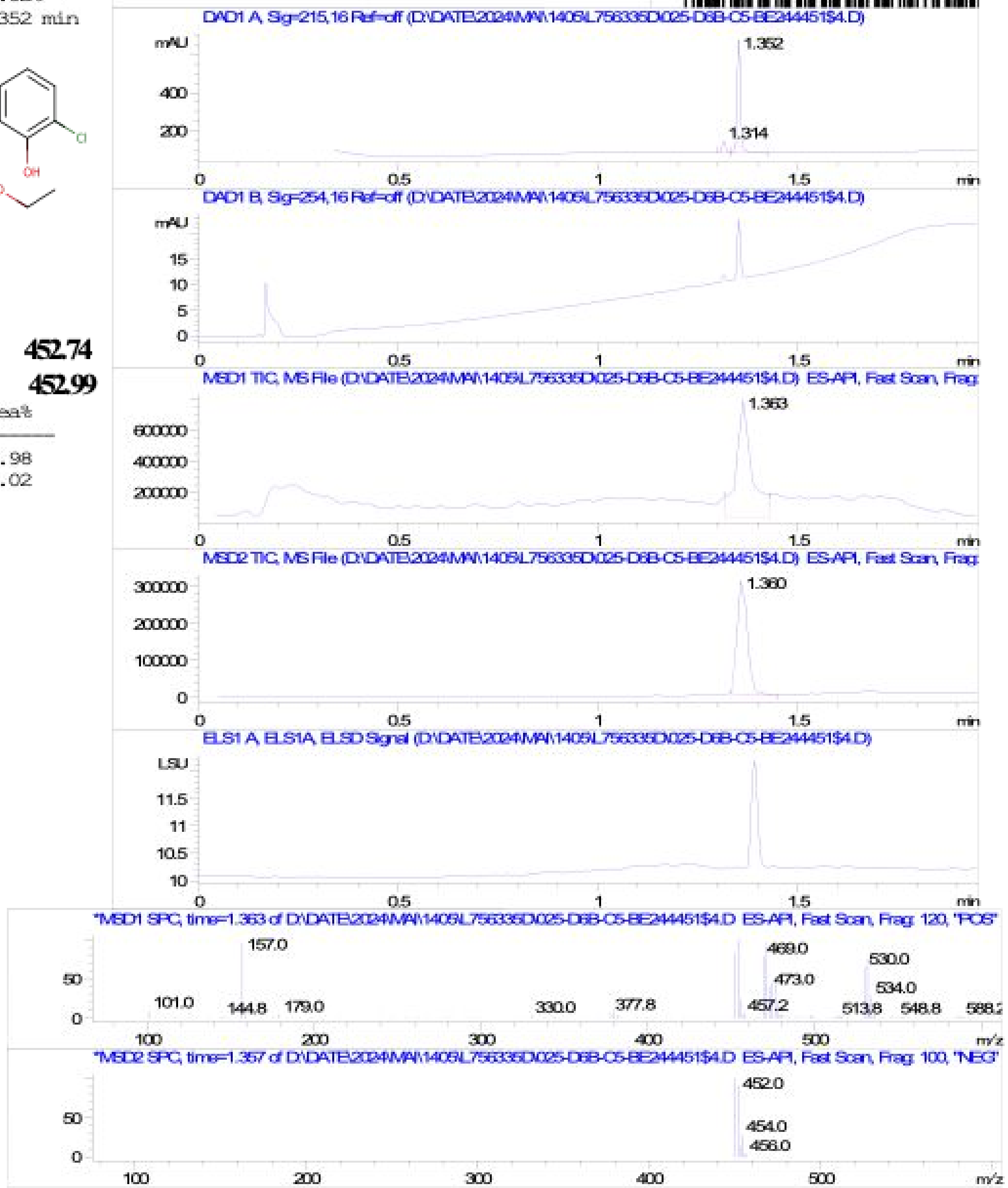

Inj.Date 5/13/2024 AN
C:\Users\Public\Documents\ChemStation\1\Data\05_13\L756335D\SUPOR_30.M

Z9103952303

```
MaxPeak:  95.06%
Ret_Time: 1.147 min
```

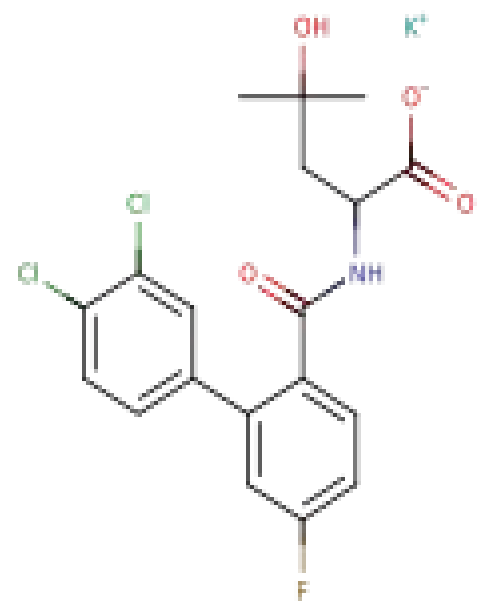

Mol Wt 452.34

Exact Mass 413.08

#	Time	Area%
1	1.147	95.06
2	1.300	4.94

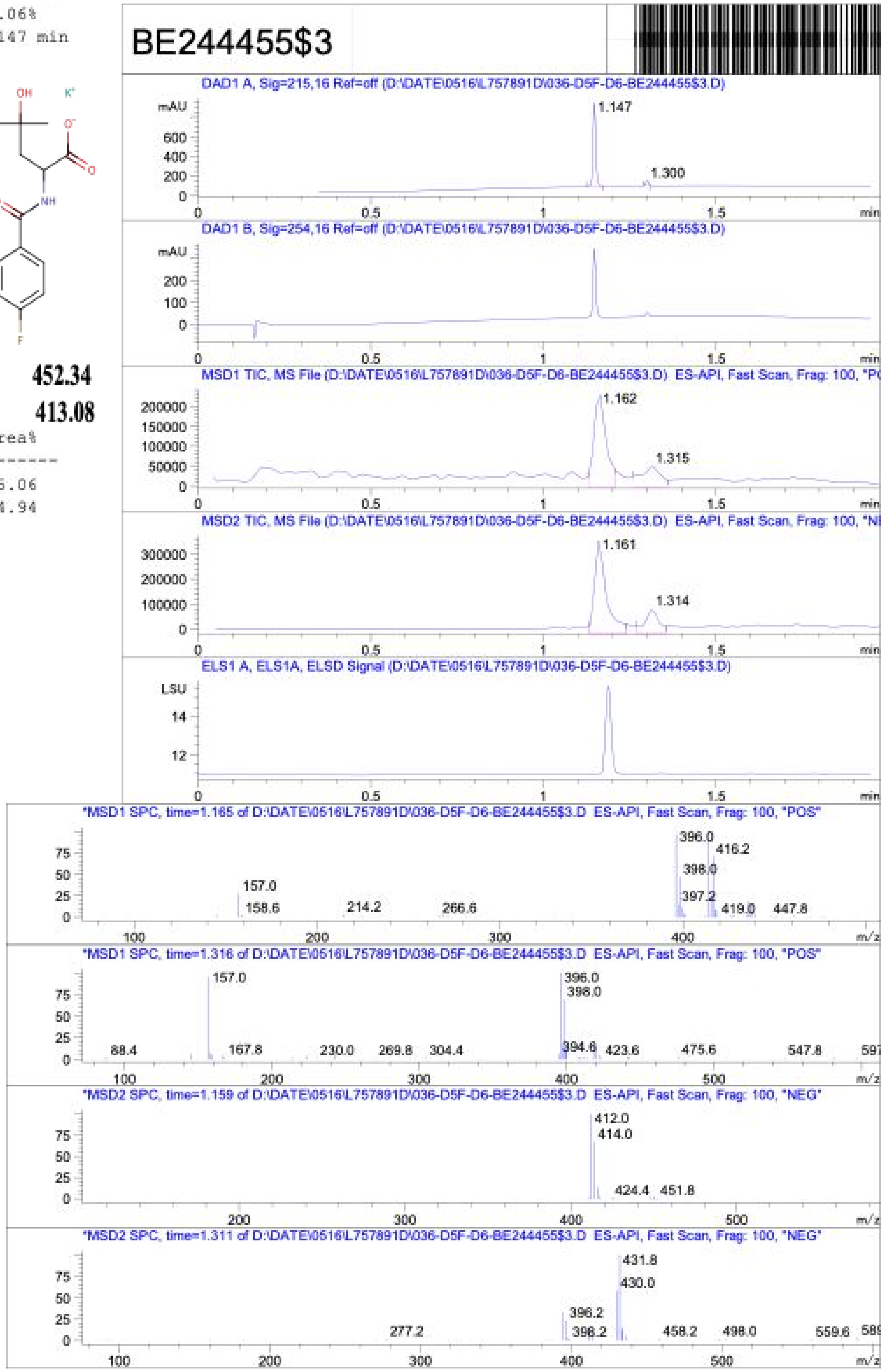

RT 1.162

RT 1.315

RT 1.161

RT 1.314

```
Inj.Date 5/16/2024                         E              23
C:\Users\Public\Documents\ChemStation\1\Data\05_16\L757891D\SUPOR_30.M
```

Z9103952336

BE244457$4

MaxPeak: 100.00%
Ret_Time: 1.232 min

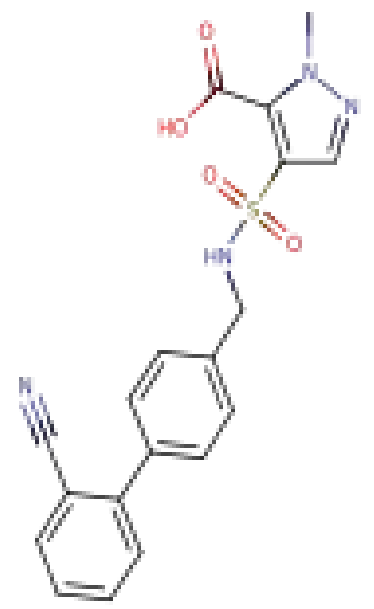

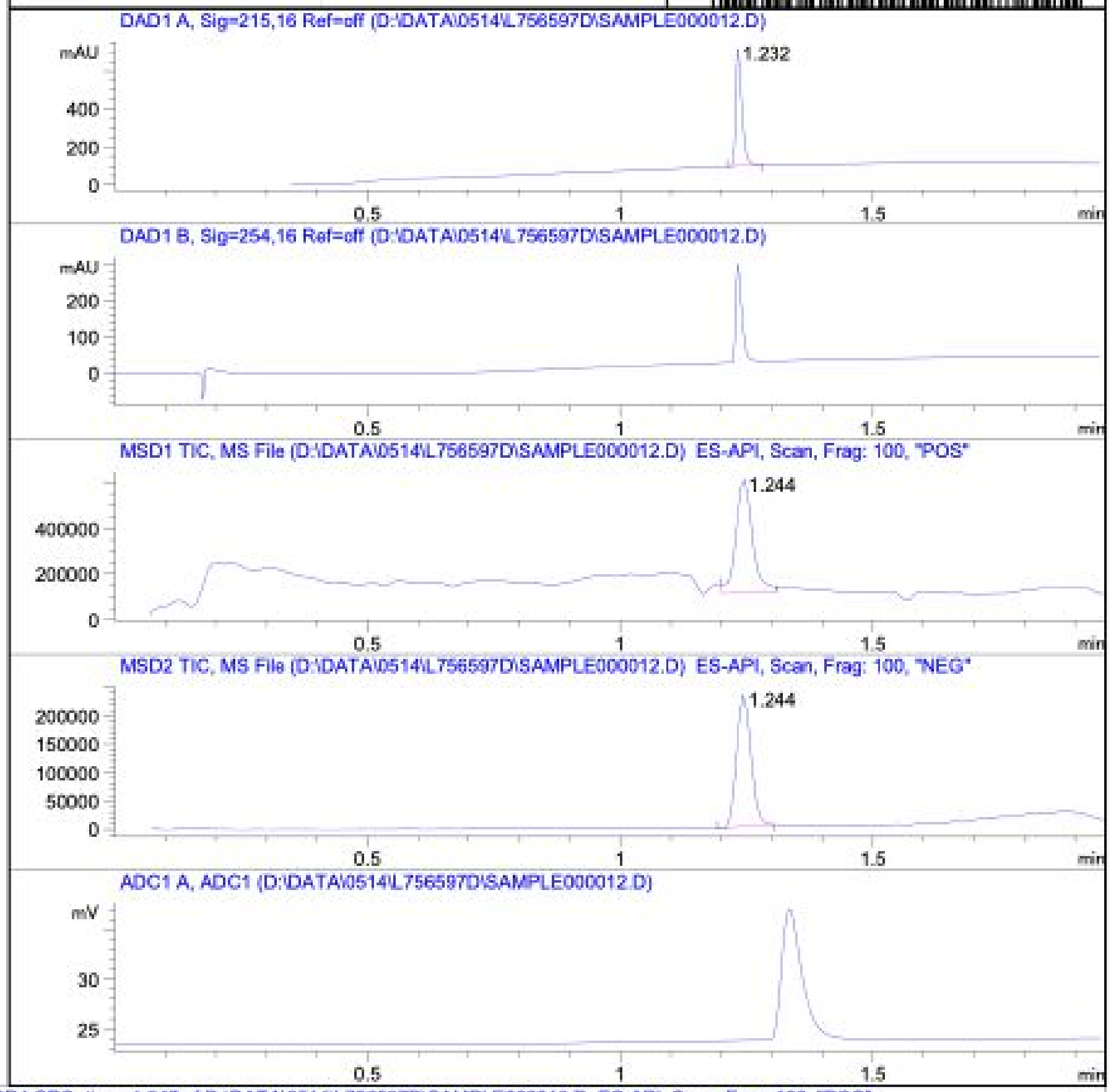

Mol Wt 396.42
Exact Mass 396.09

#	Time	Area%
1	1.232	100.00

RT 1.244

RT 1.244

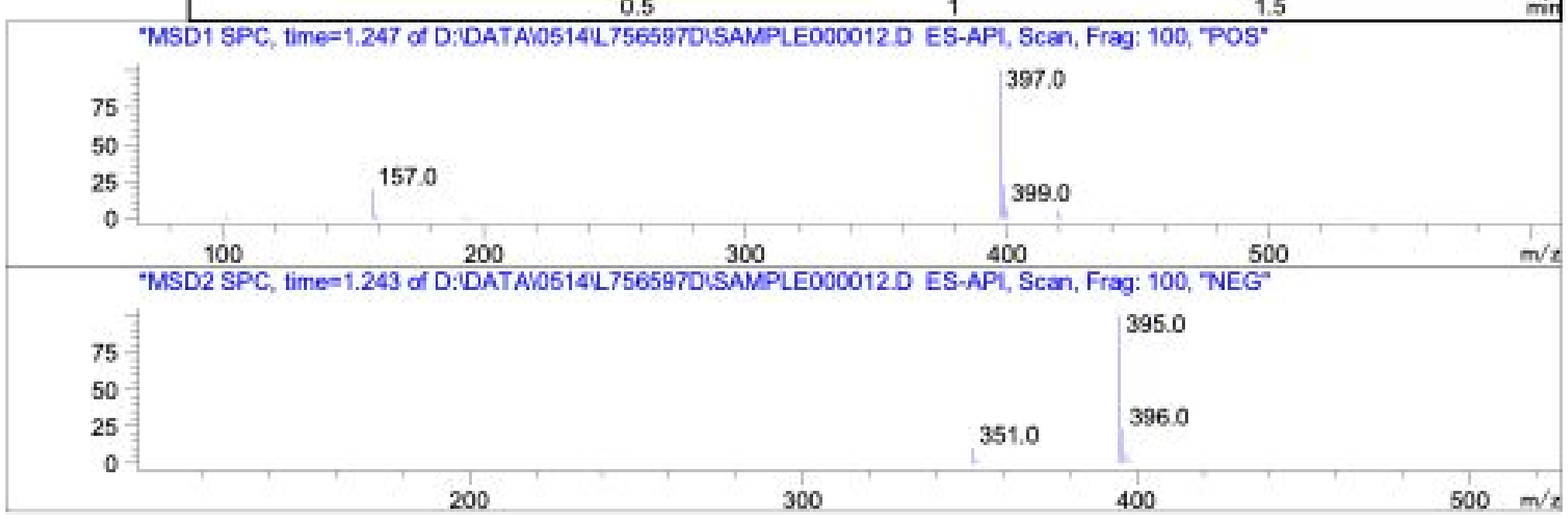

Inj.Date 5/14/2024 VB - 4 -
C:\CHEM32\1\METHODS\SUPOR_30.M

Z9103952349

MaxPeak: 100.00%
Ret_Time: 1.153 min

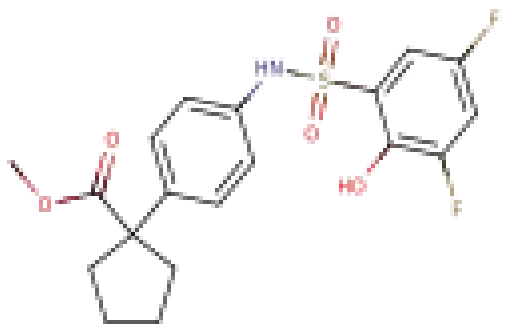

Mol Wt 411.42
Exact Mass 411.11

#	Time	Area%
1	1.153	100.00

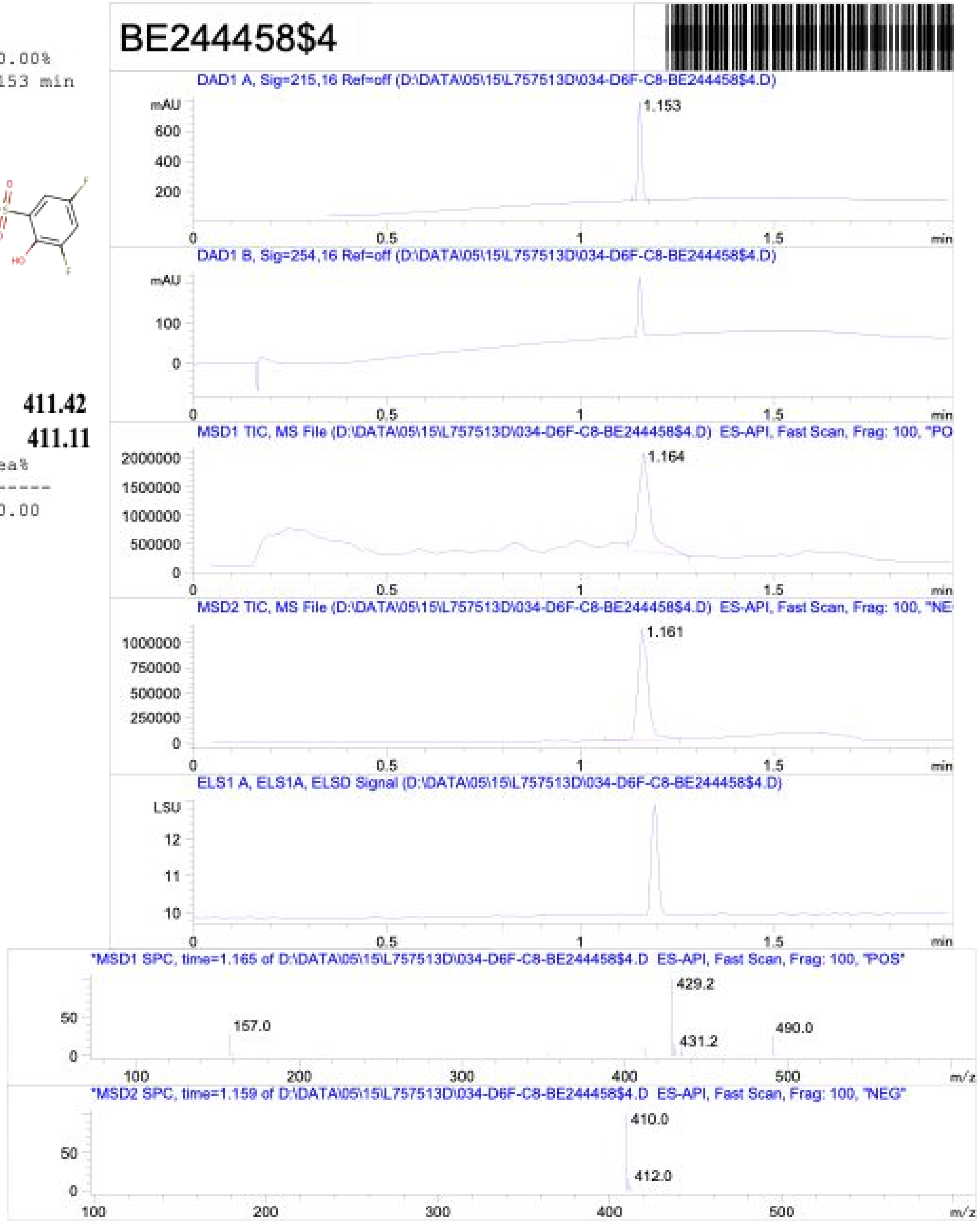

RT 1.164

RT 1.161

Inj.Date 5/15/2024 CH 31
C:\Users\Public\Documents\ChemStation\1\Data\05_15\L757513D\SUPOR_30.M

Z9103952355

MaxPeak: 100.00%
Ret_Time: 1.335 min

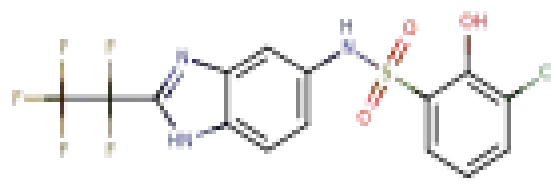

Mol Wt 441.76
Exact Mass 441

#	Time	Area%
1	1.335	100.00

RT 1.345

RT 1.344

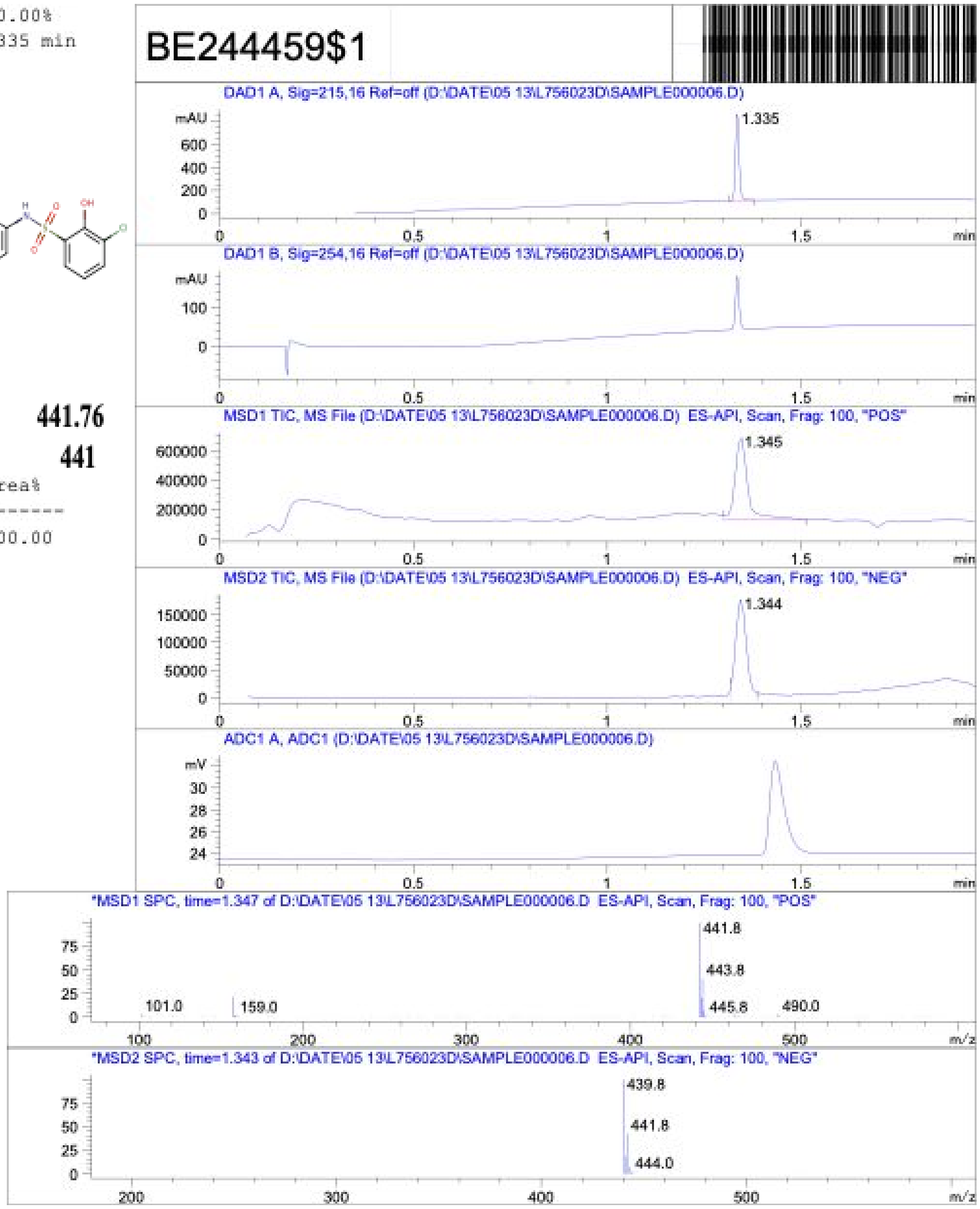

Inj.Date 5/13/2024 E - 4 -
C:\CHEM32\1\METHODS\SUPOR_30.M

Z9103952375

MaxPeak: 100.00%
Ret_Time: 1.453 min

BE244461$4

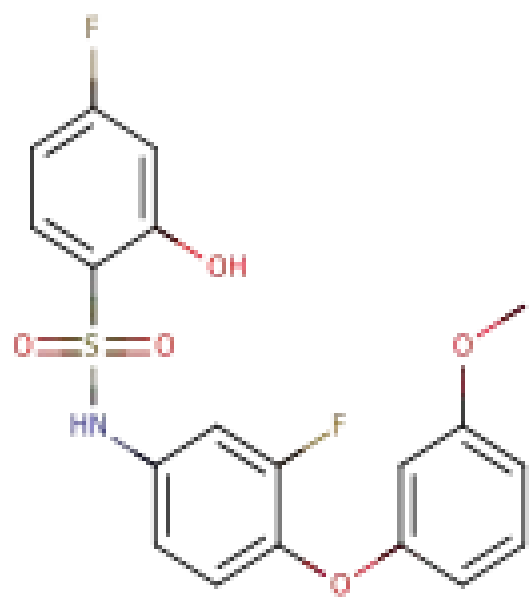

Mol Wt 407.39

Exact Mass 407.07

#	Time	Area%
1	1.453	100.00

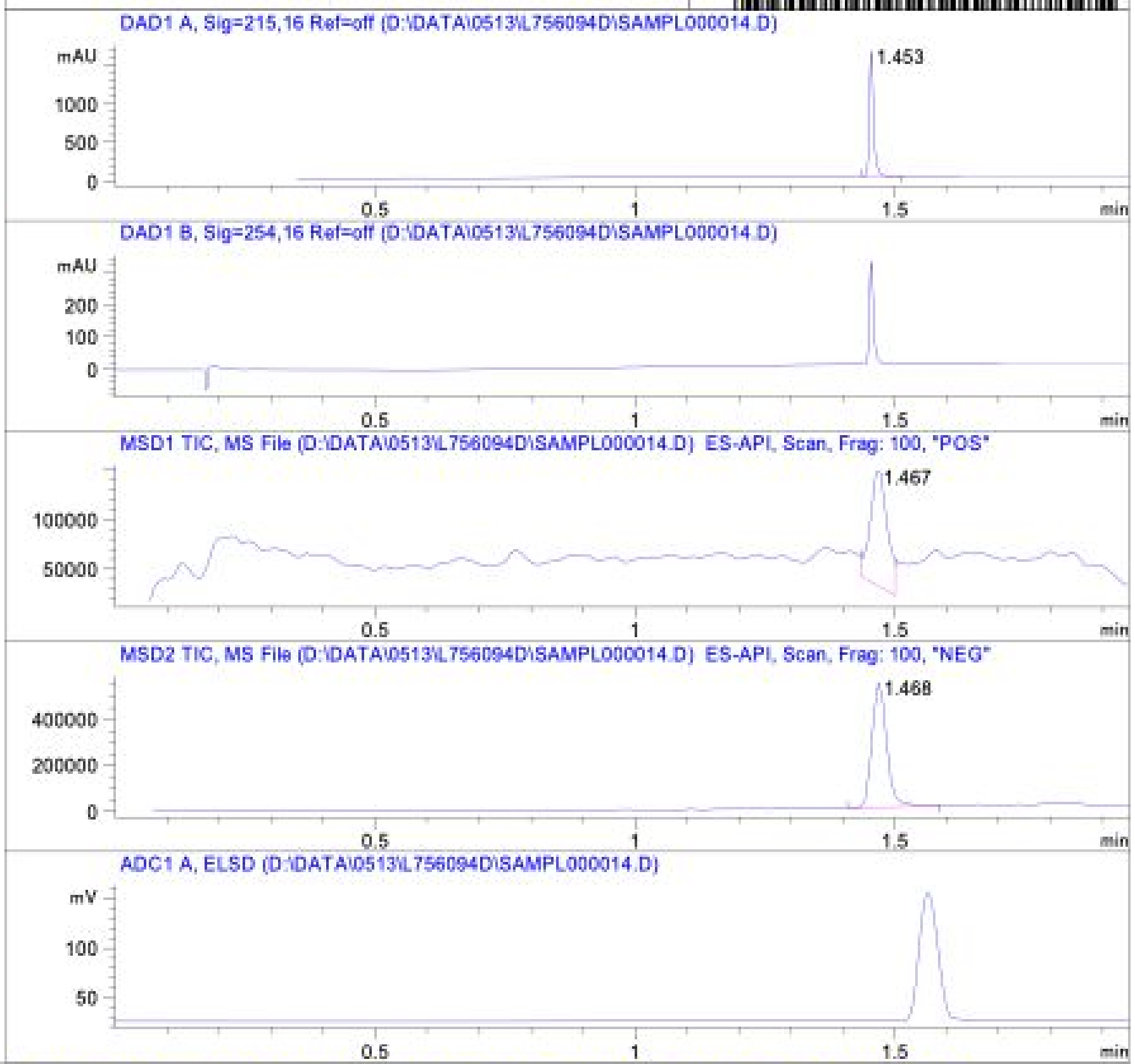

RT 1.467

RT 1.468

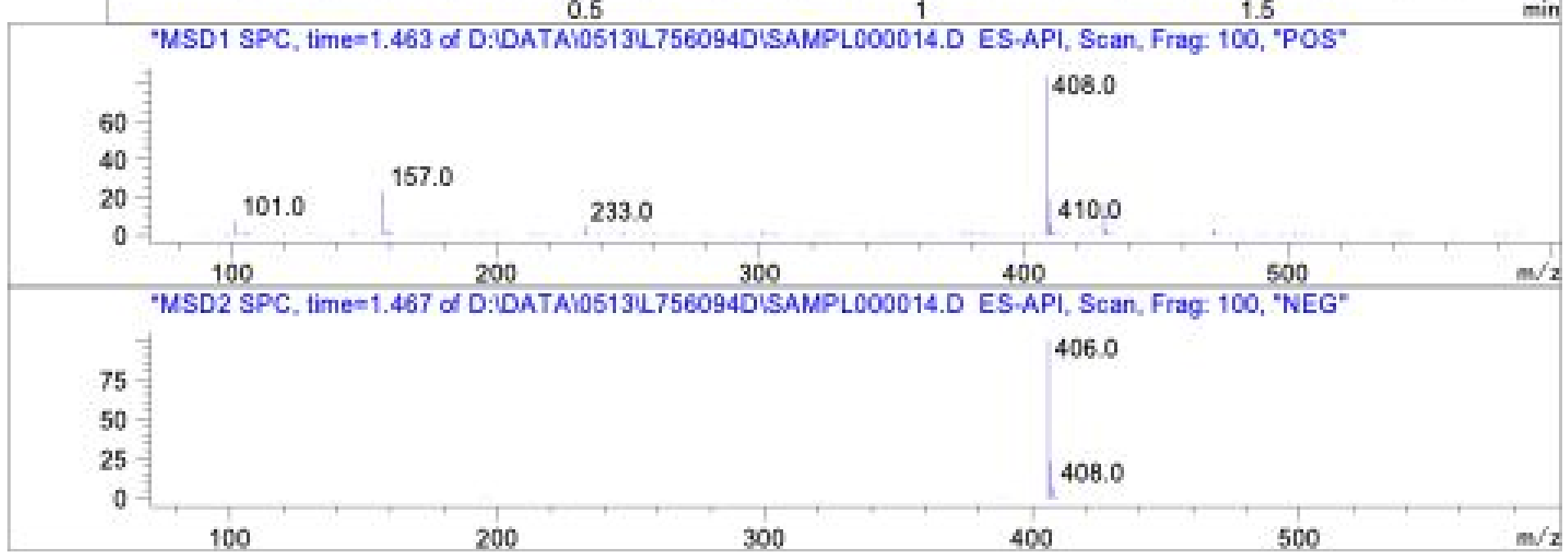

Inj.Date 5/13/2024 LB -6-
C:\CHEM32\1\METHODS\SUPOR_30.M

Z9103952401

MaxPeak: 97.81%
Ret_Time: 1.325 min

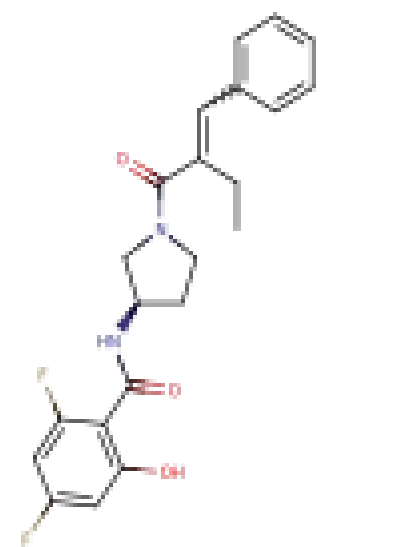

Mol Wt 400.42

Exact Mass 400.19

#	Time	Area%
1	1.312	2.19
2	1.325	97.81

BE245475$4

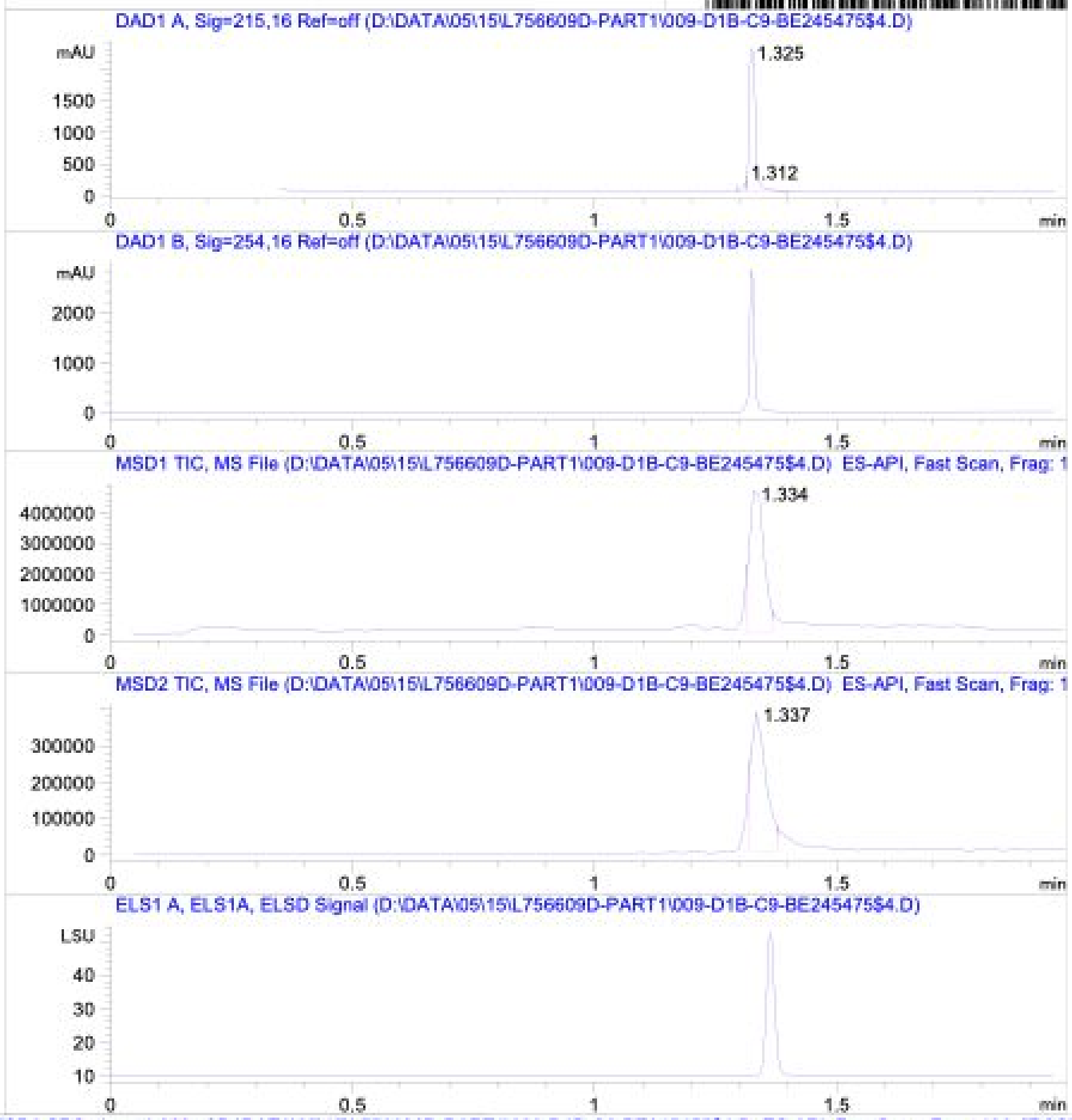

RT 1.334

RT 1.337

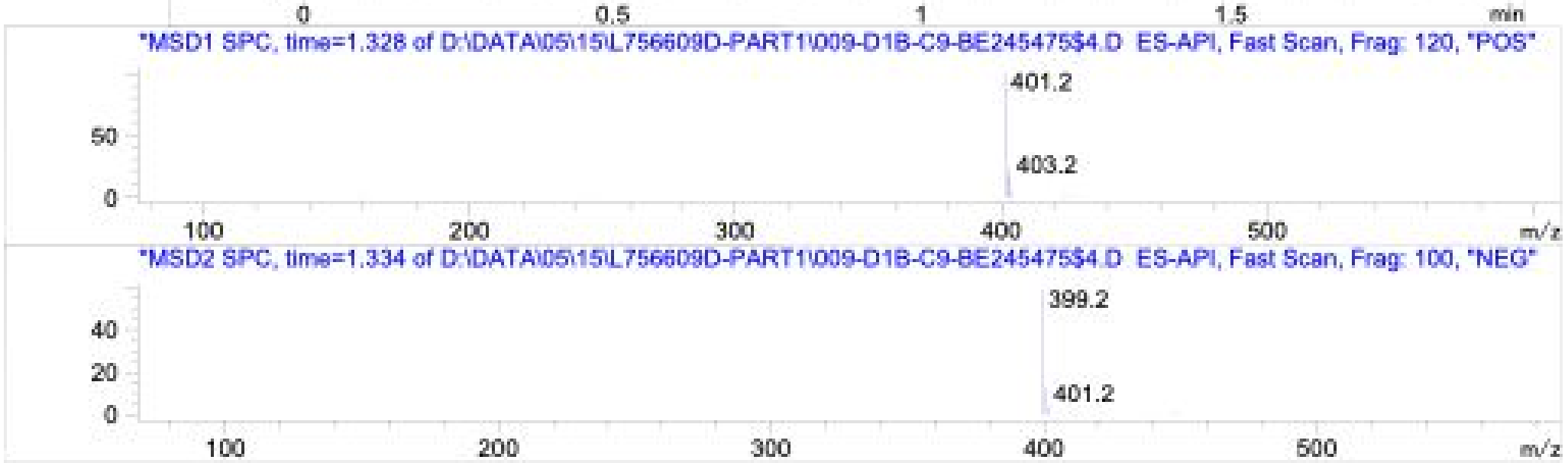

Inj.Date 5/14/2024 CH
C:\Users\Public\Documents\ChemStation\1\Data\05_14\1756609D\SUPOR_96.M

Z9103952406

MaxPeak: 95.87%
Ret_Time: 0.922 min

BE569258$1

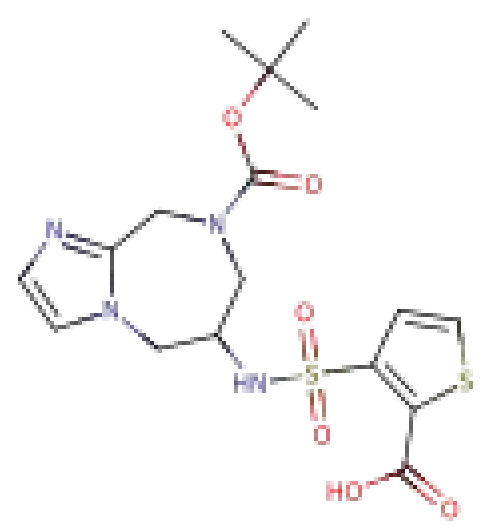

Mol Wt 442.51
Exact Mass 442.1

#	Time	Area%
1	0.761	1.81
2	0.922	95.87
3	1.213	2.32

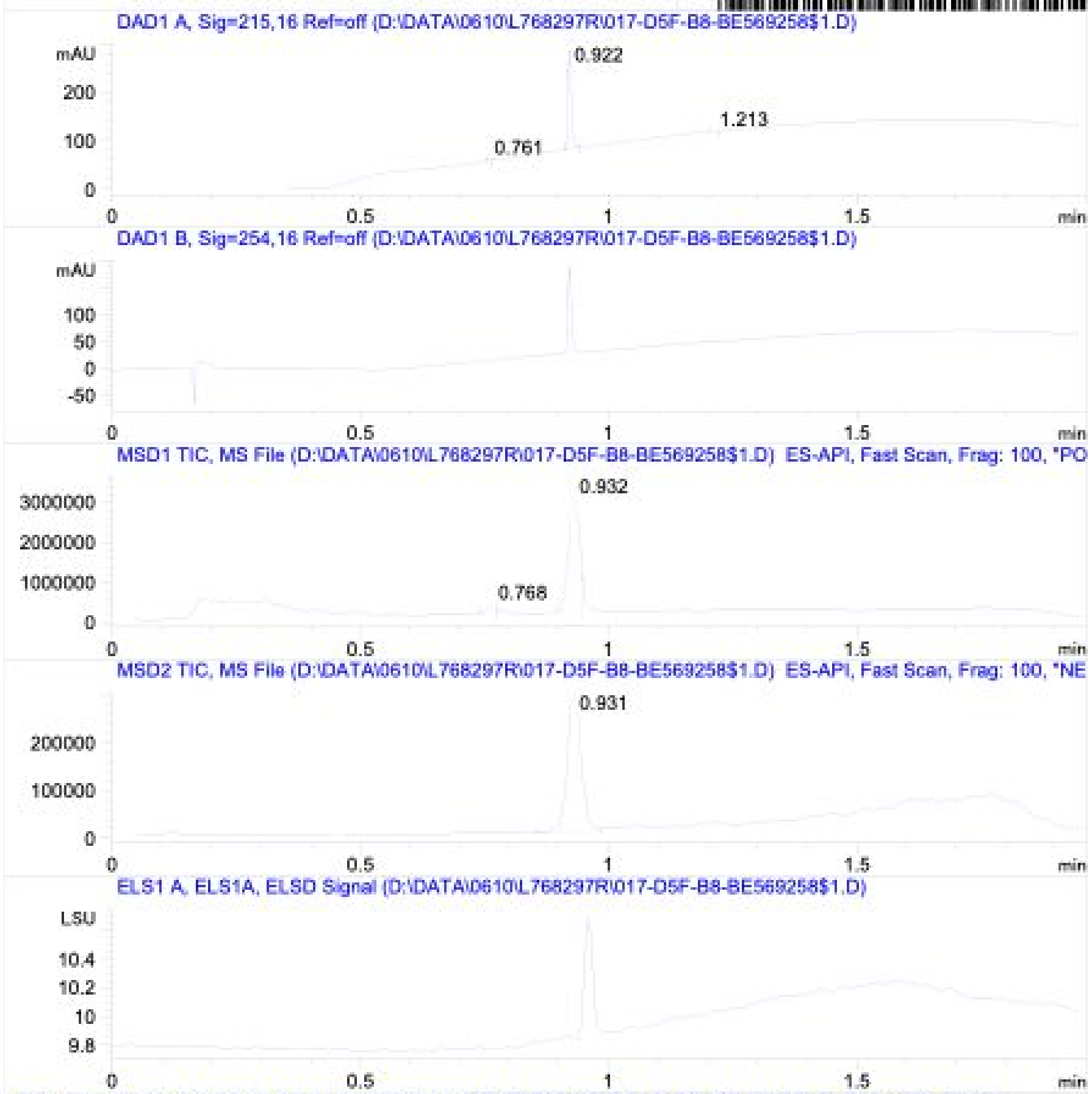

RT 0.768

RT 0.932

RT 0.931

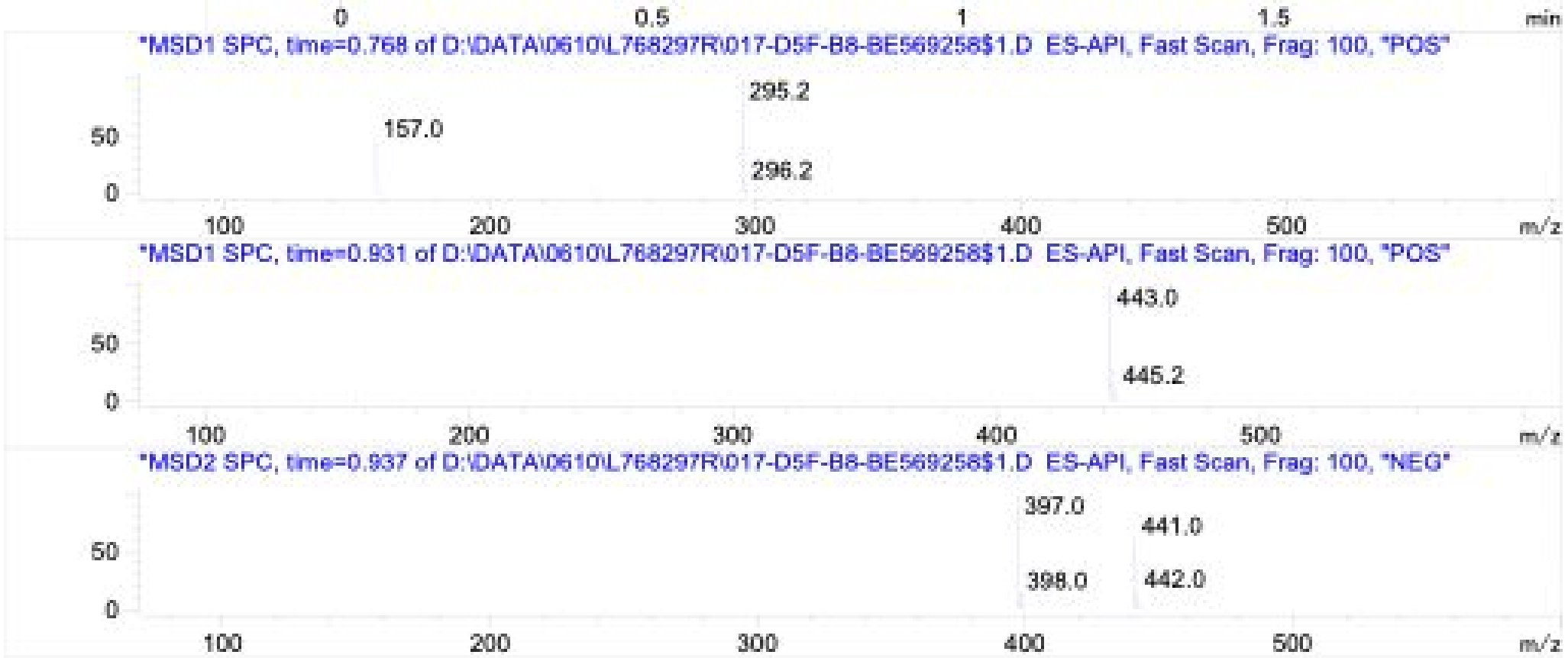

Inj.Date 6/10/2024 H 31
C:\Users\Public\Documents\ChemStation\1\Data\06_10\L768297R\SUPOR_30.M

Z9103952413

MaxPeak: 97.20%
Ret_Time: 1.249 min

BE244463$23

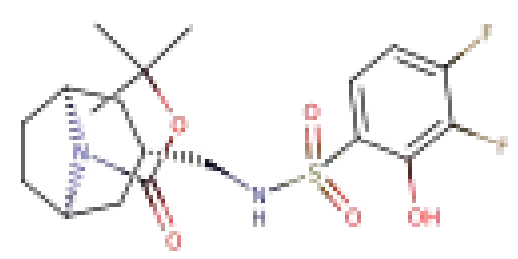

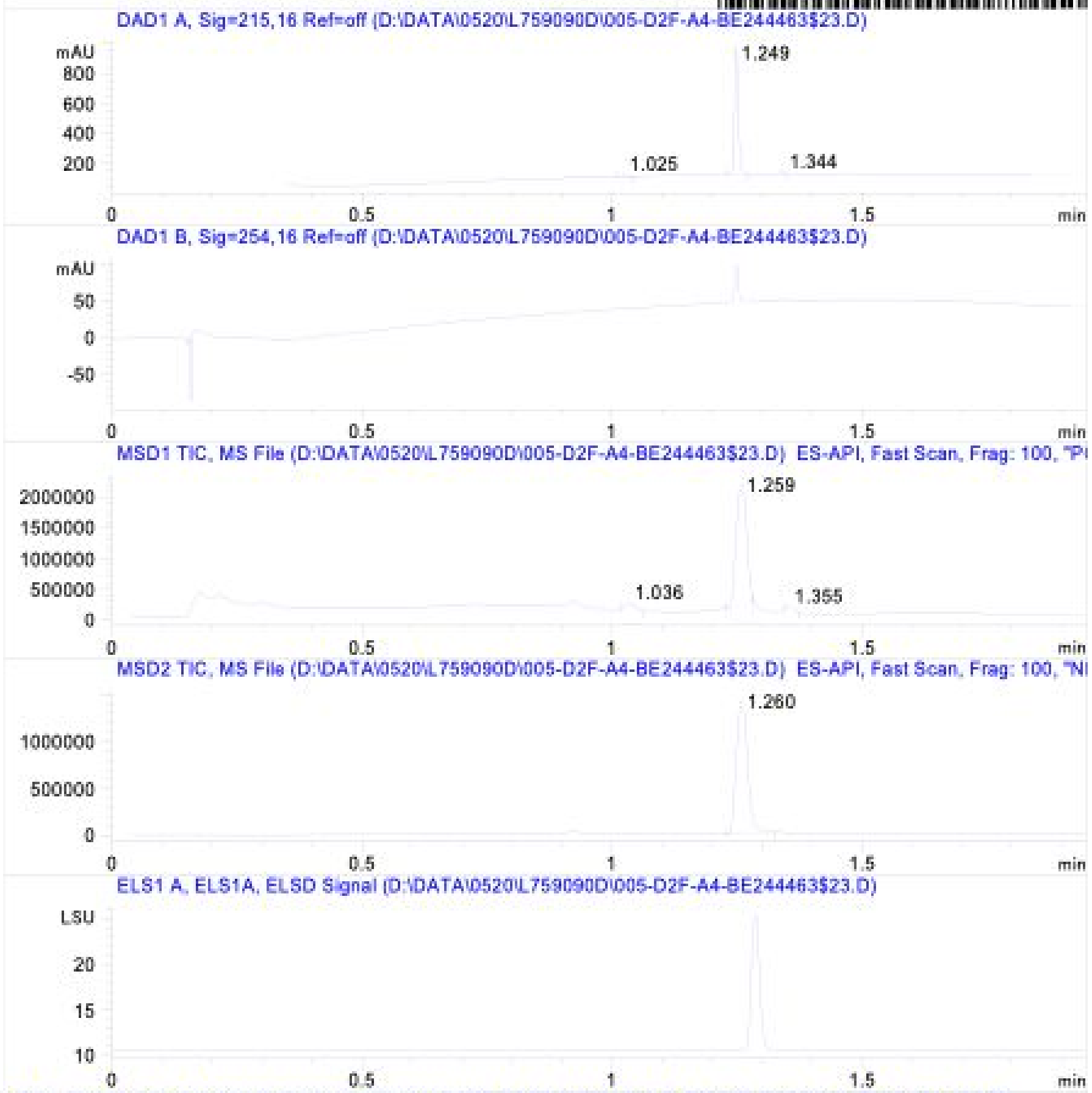

Mol Wt 432.48
Exact Mass 432.18

#	Time	Area%
1	1.025	2.08
2	1.249	97.20
3	1.344	0.72

RT 1.036

RT 1.259

RT 1.355

RT 1.260

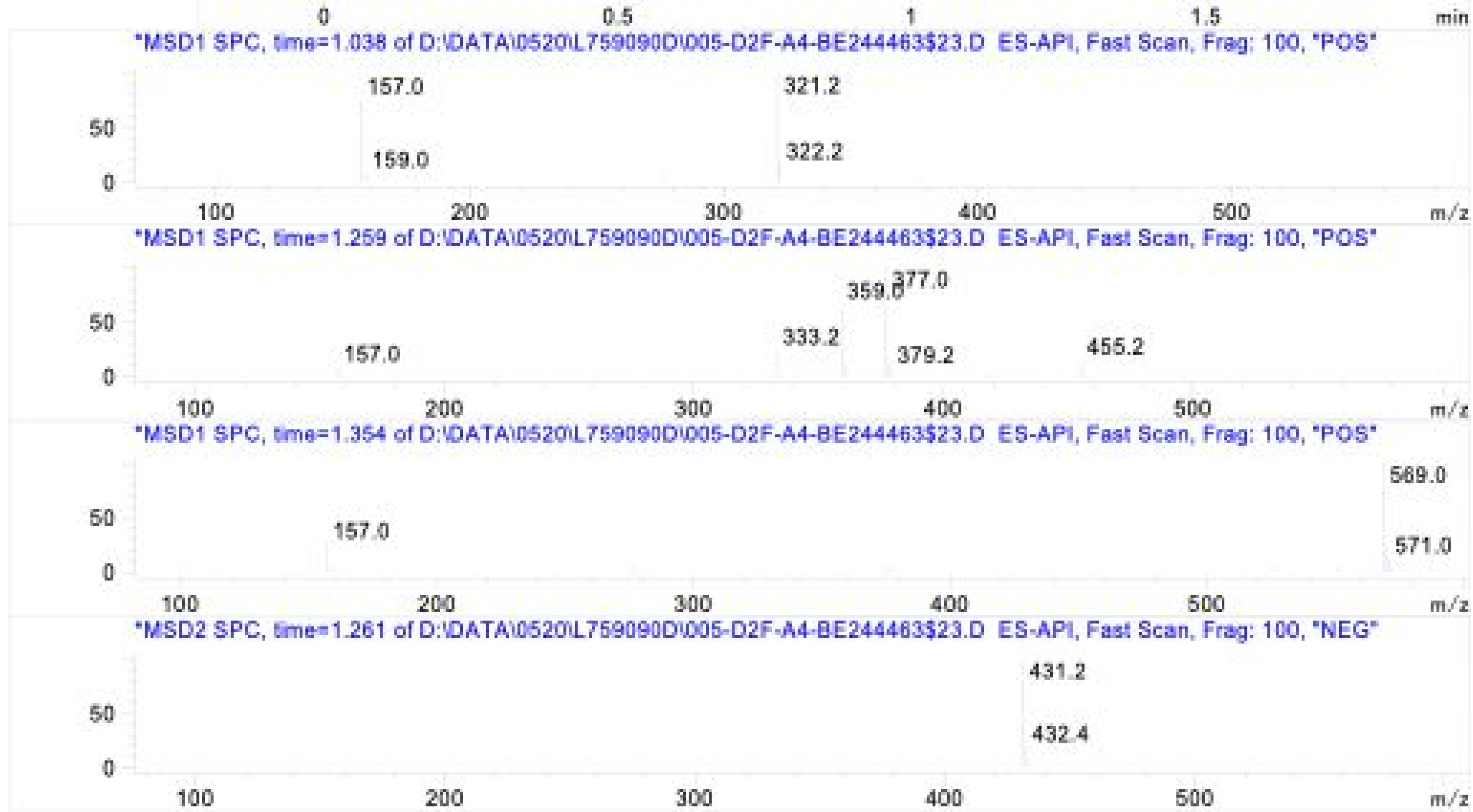

Inj.Date 5/20/2024 H 28
C:\Users\Public\Documents\ChemStation\1\Data\05_20\L759090D\SUPOR_96.M

Z9103952420

MaxPeak: 96.21%
Ret_Time: 1.302 min

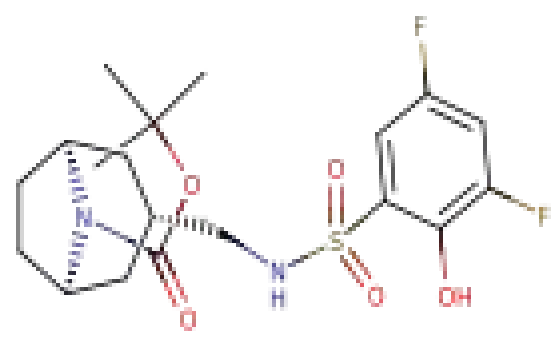

Mol Wt 432.48

Exact Mass 432.18

#	Time	Area%
1	1.000	3.79
2	1.302	96.21

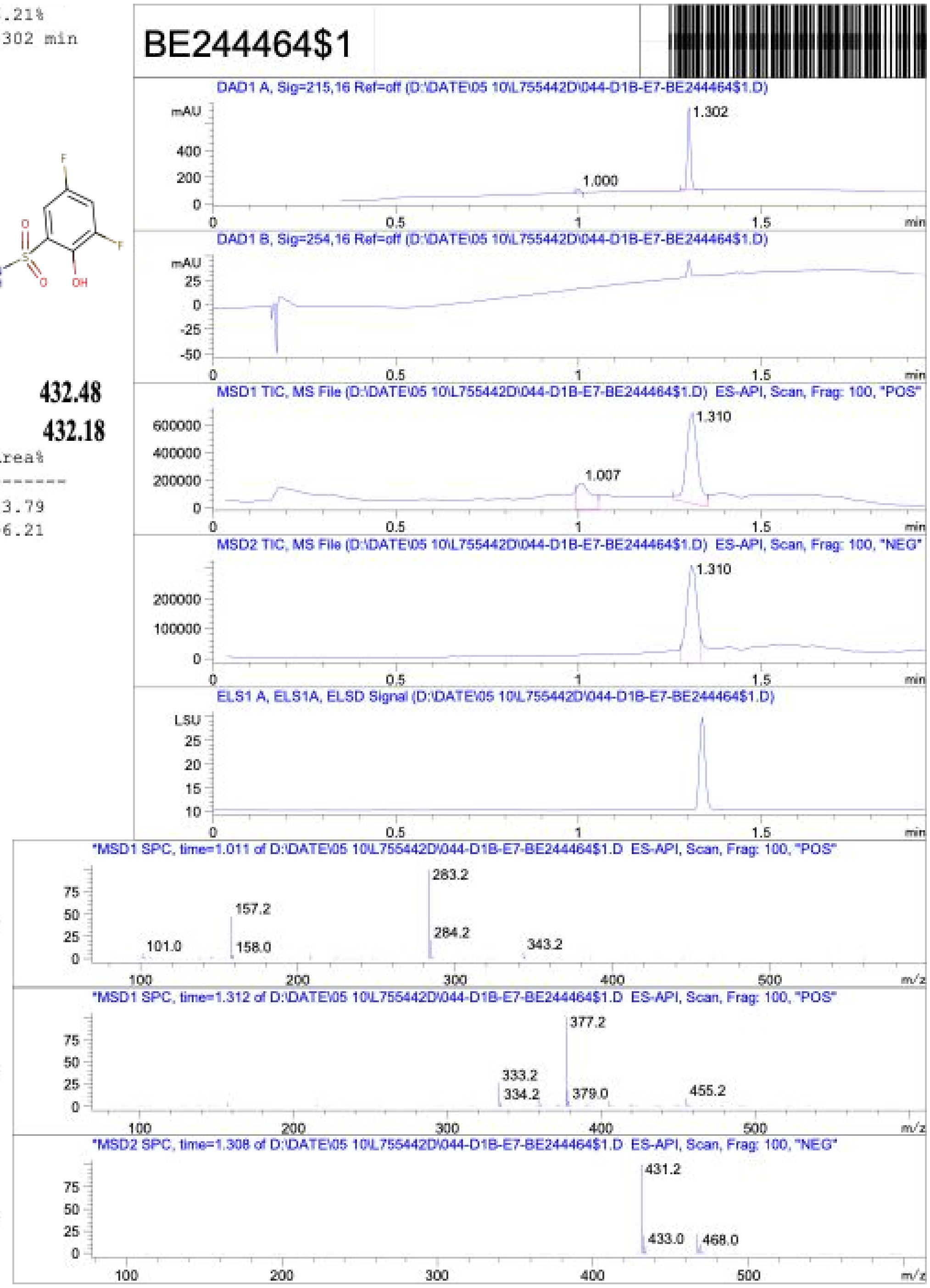

Inj.Date 5/10/2024 E -25-
C:\Users\Public\Documents\ChemStation\1\Data\05_10\L755442D\SUPOR_96.M

Z9103952426

MaxPeak: 100.00%
Ret_Time: 0.988 min

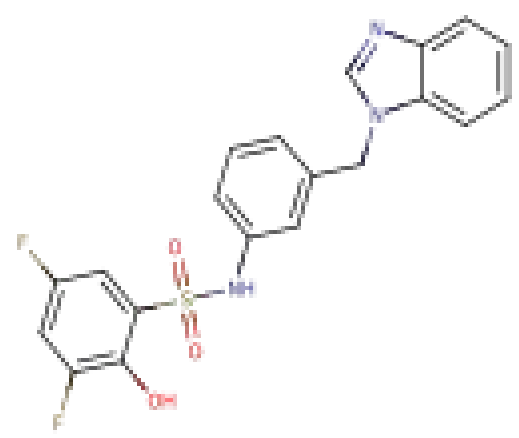

Mol Wt 415.41
Exact Mass 415.09

#	Time	Area%
1	0.988	100.00

BE588482$1

RT 1.000

RT 1.001

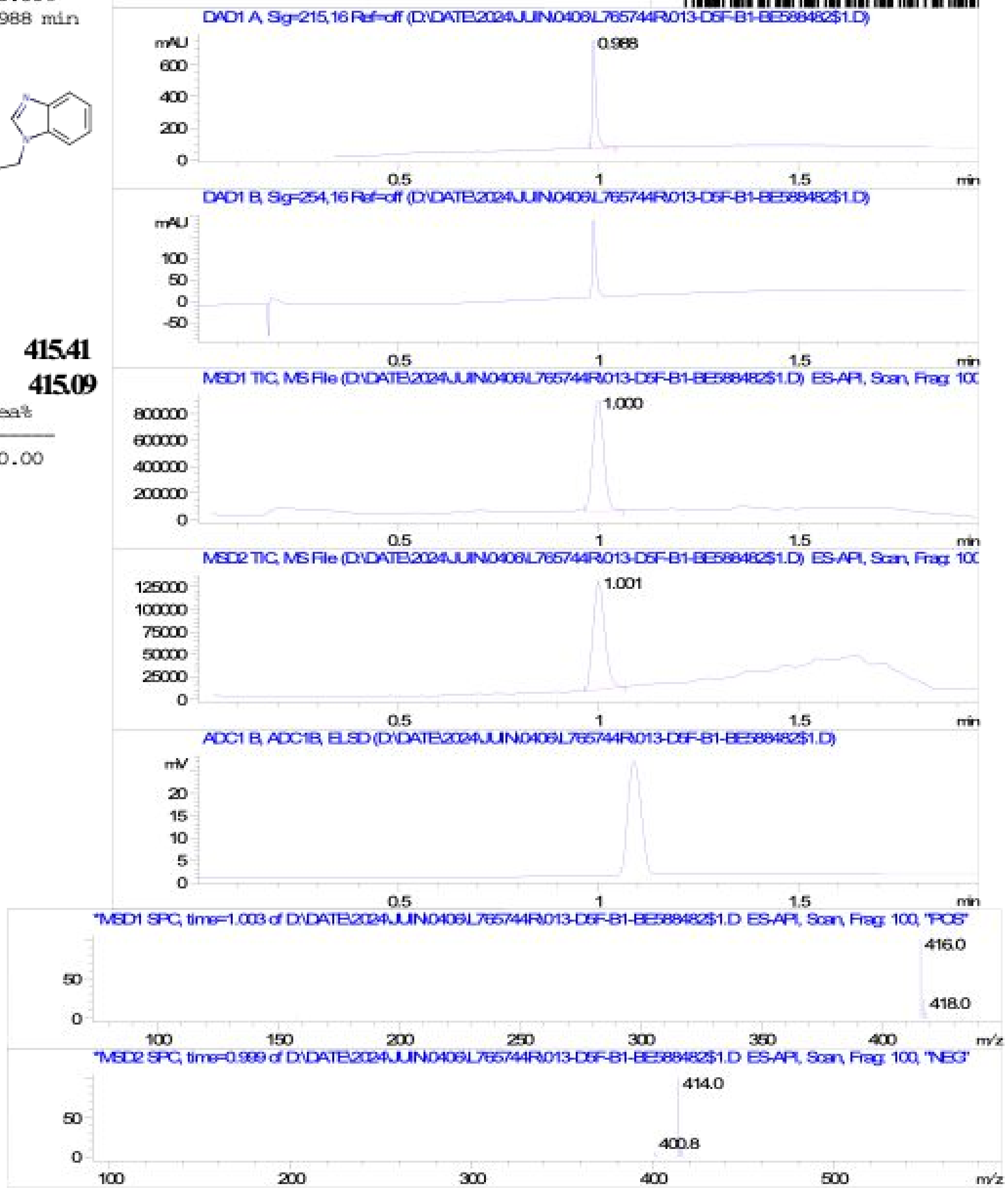

Inj.Date 6/4/2024 AN -25-
C:\Users\Public\Documents\ChemStation\1\Data\06_04\L765744R\SUPOR_30.M

Z9103952451

MaxPeak: 100.00%
Ret_Time: 1.349 min

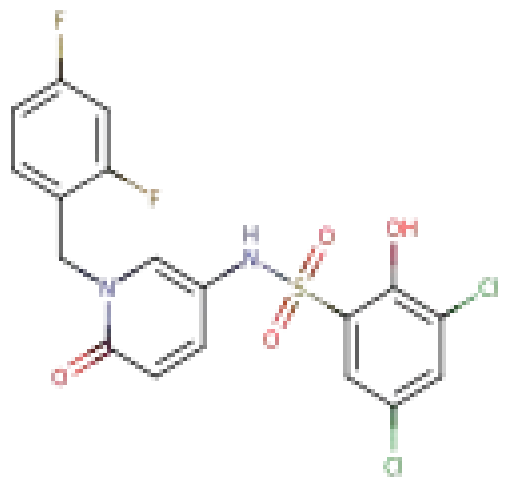

Mol Wt 461.27
Exact Mass 459.99

#	Time	Area%
1	1.349	100.00

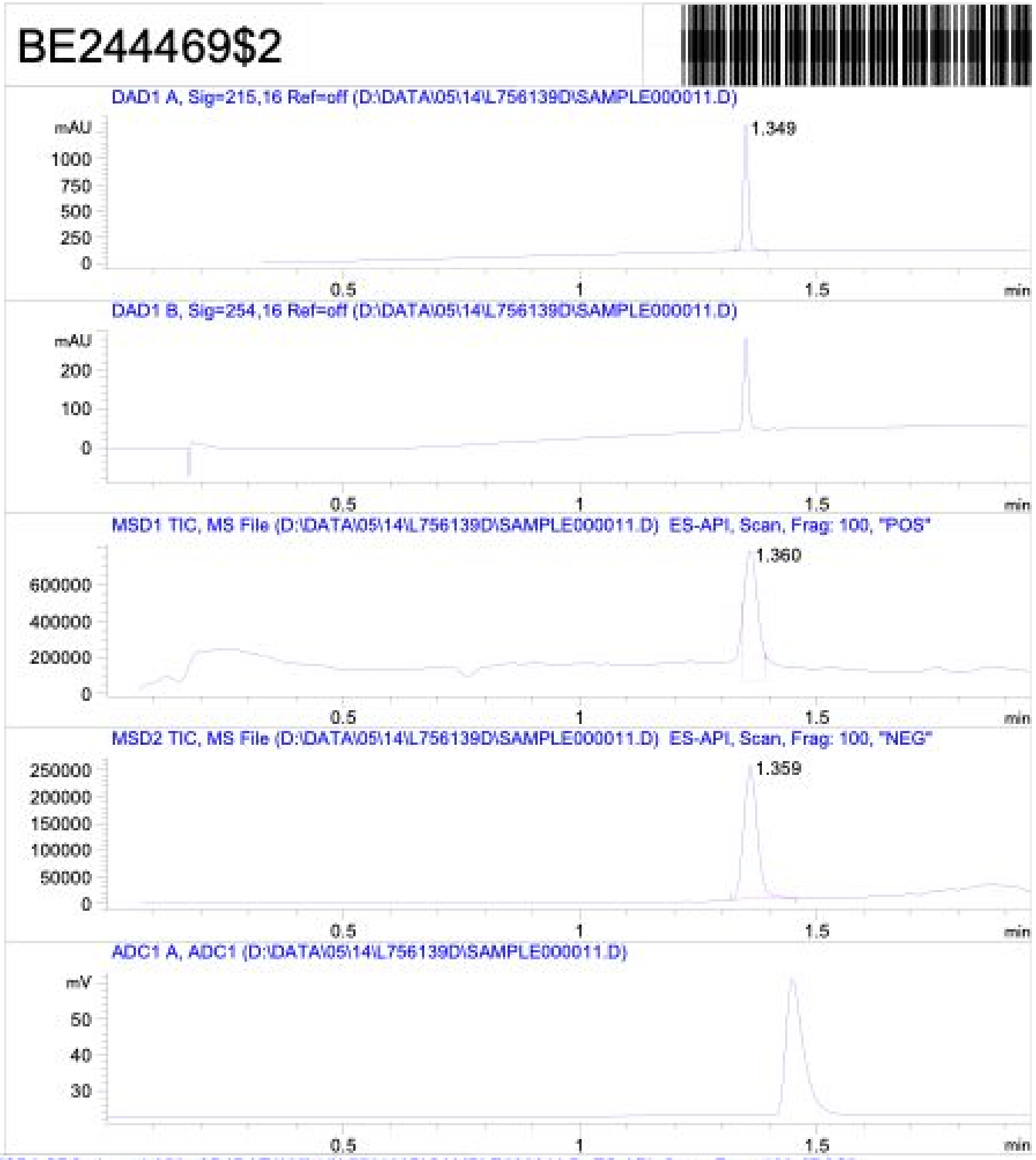

RT 1.360

RT 1.359

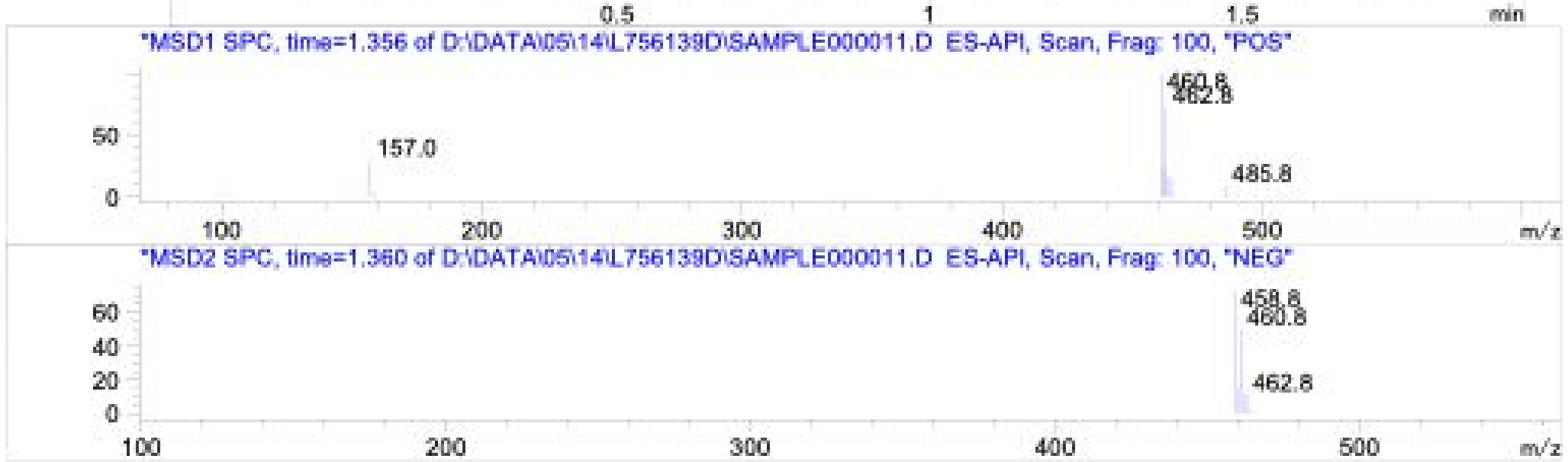

Inj.Date 5/13/2024 CH - 4 -
C:\CHEM32\1\METHODS\SUPOR_30.M

Z9103952464

MaxPeak: 100.00%
Ret_Time: 1.254 min

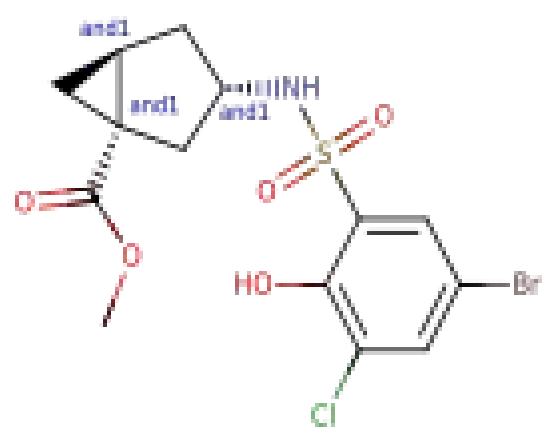

Mol Wt 424.7

Exact Mass 424.96

#	Time	Area%
1	1.254	100.00

BE244982$1

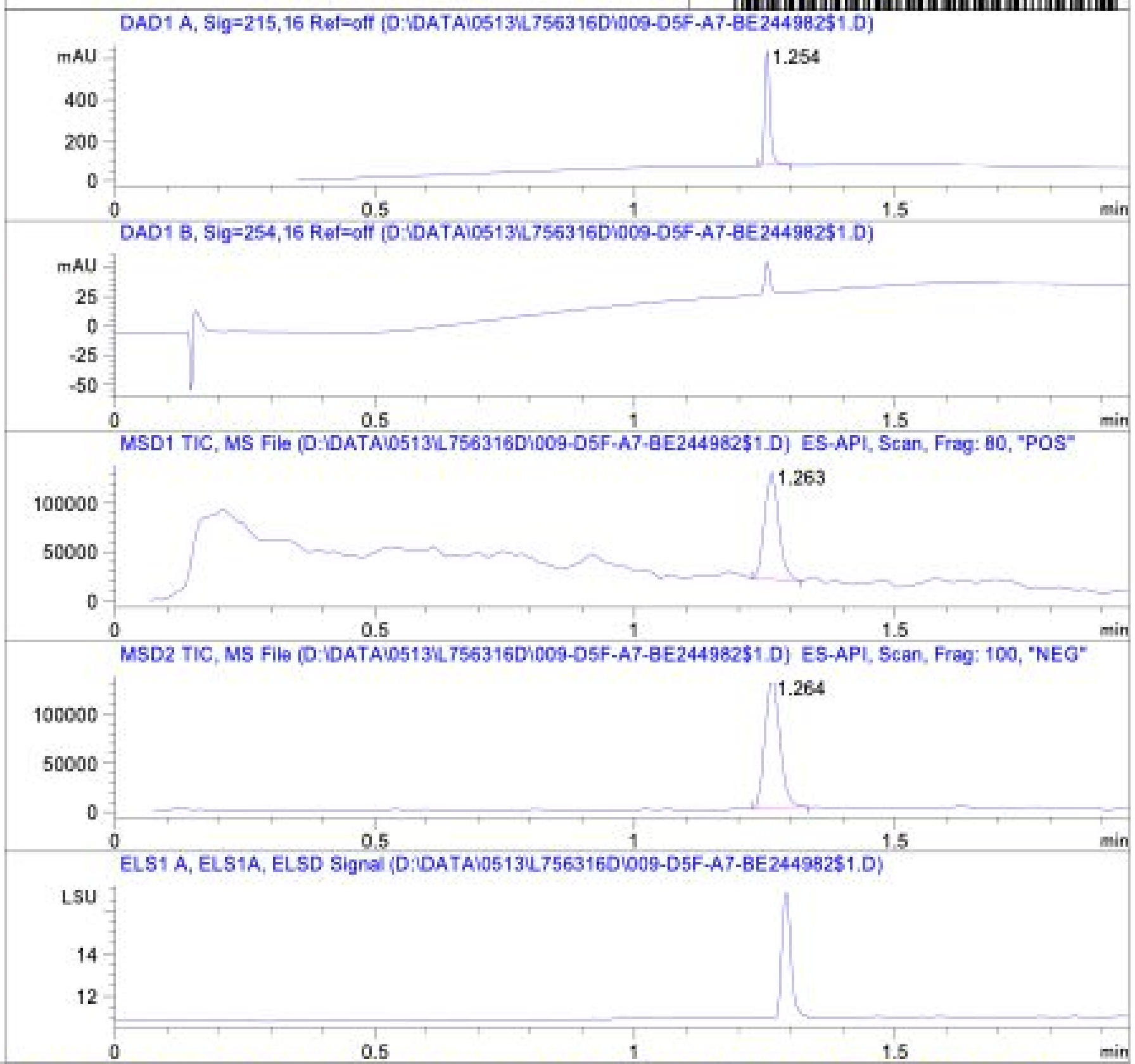

RT 1.263

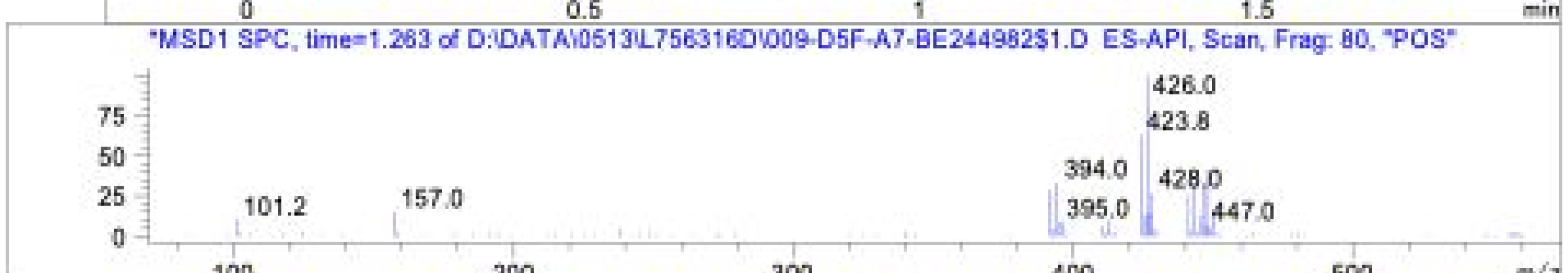

RT 1.264

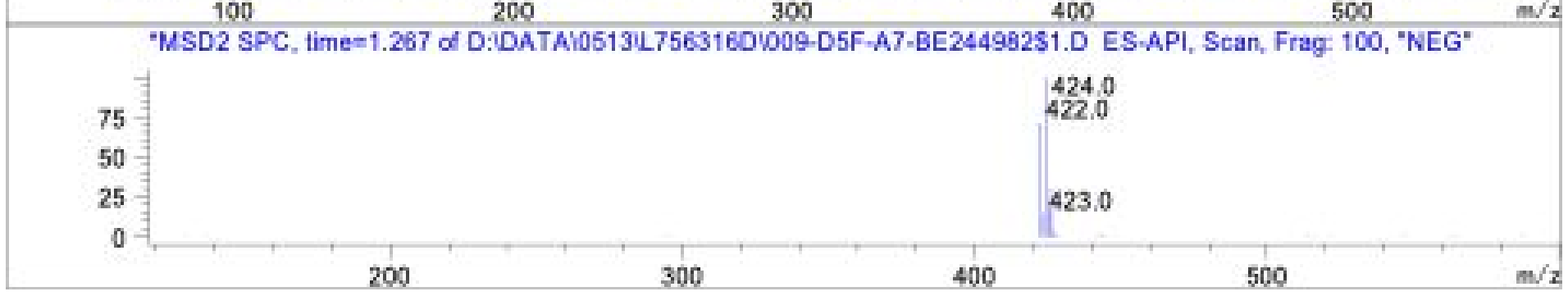

Inj.Date 5/13/2024 LB -16-
C:\Chem32\1\Data\05_13\L756316D\SUPOR_30.M

Z9103996037

MaxPeak: 100.00%
Ret_Time: 1.463 min

BE244983$2

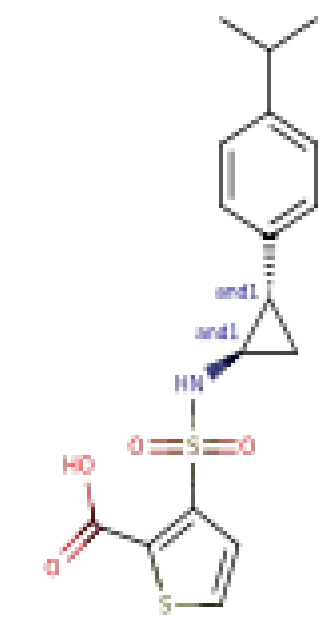

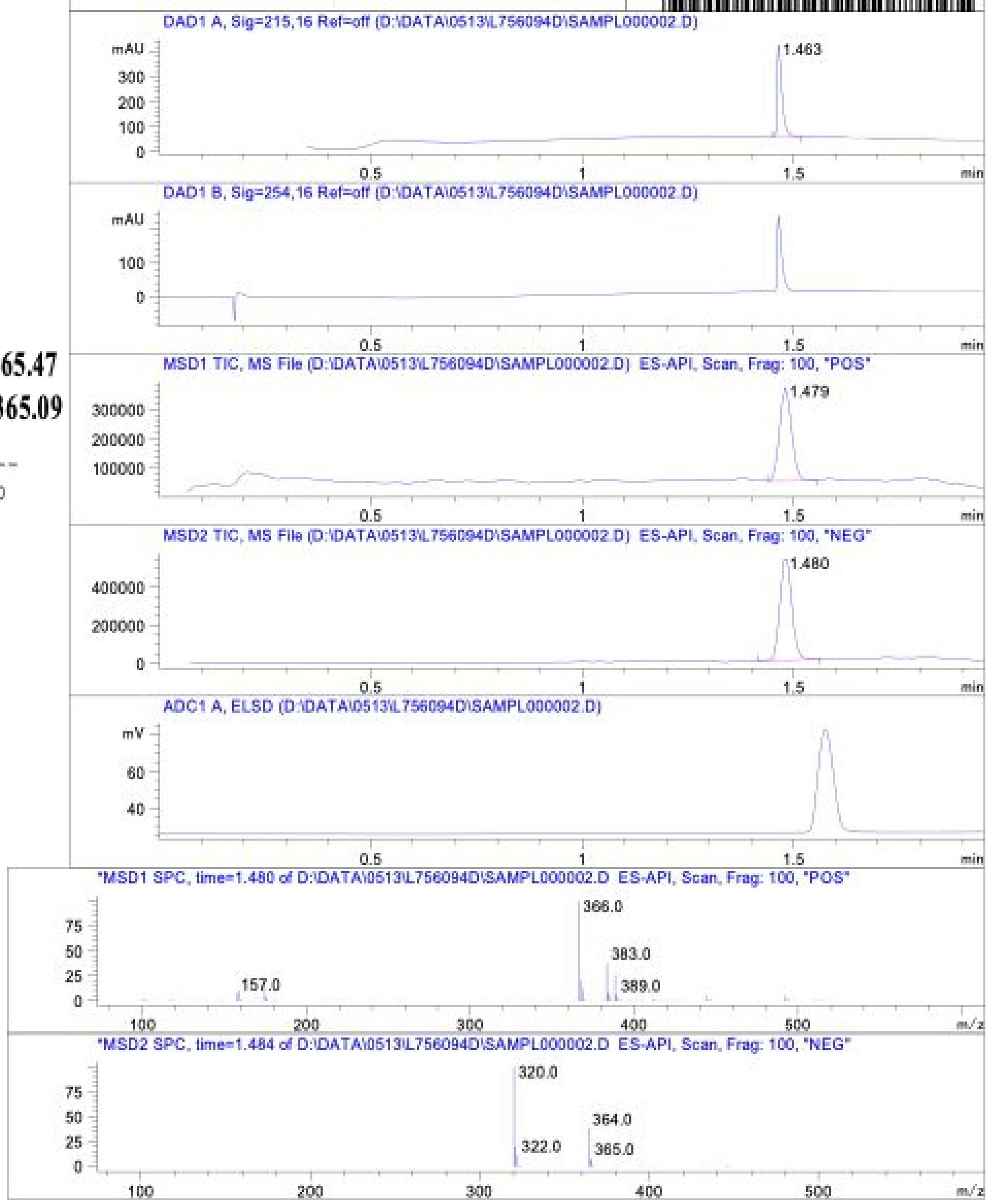

Mol Wt 365.47

Exact Mass 365.09

#	Time	Area%
1	1.463	100.00

RT 1.479

RT 1.480

Inj.Date 5/13/2024 LB -6-

C:\CHEM32\1\METHODS\SUPOR_30.M

Z9103997588

MaxPeak: 100.00%
Ret_Time: 1.239 min

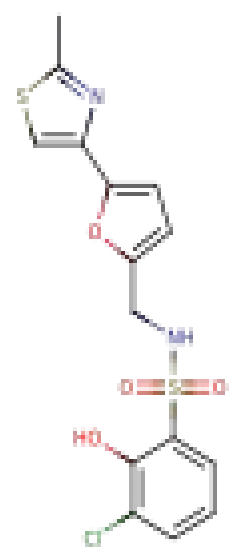

Mol Wt 384.86

Exact Mass 384

#	Time	Area%
1	1.239	100.00

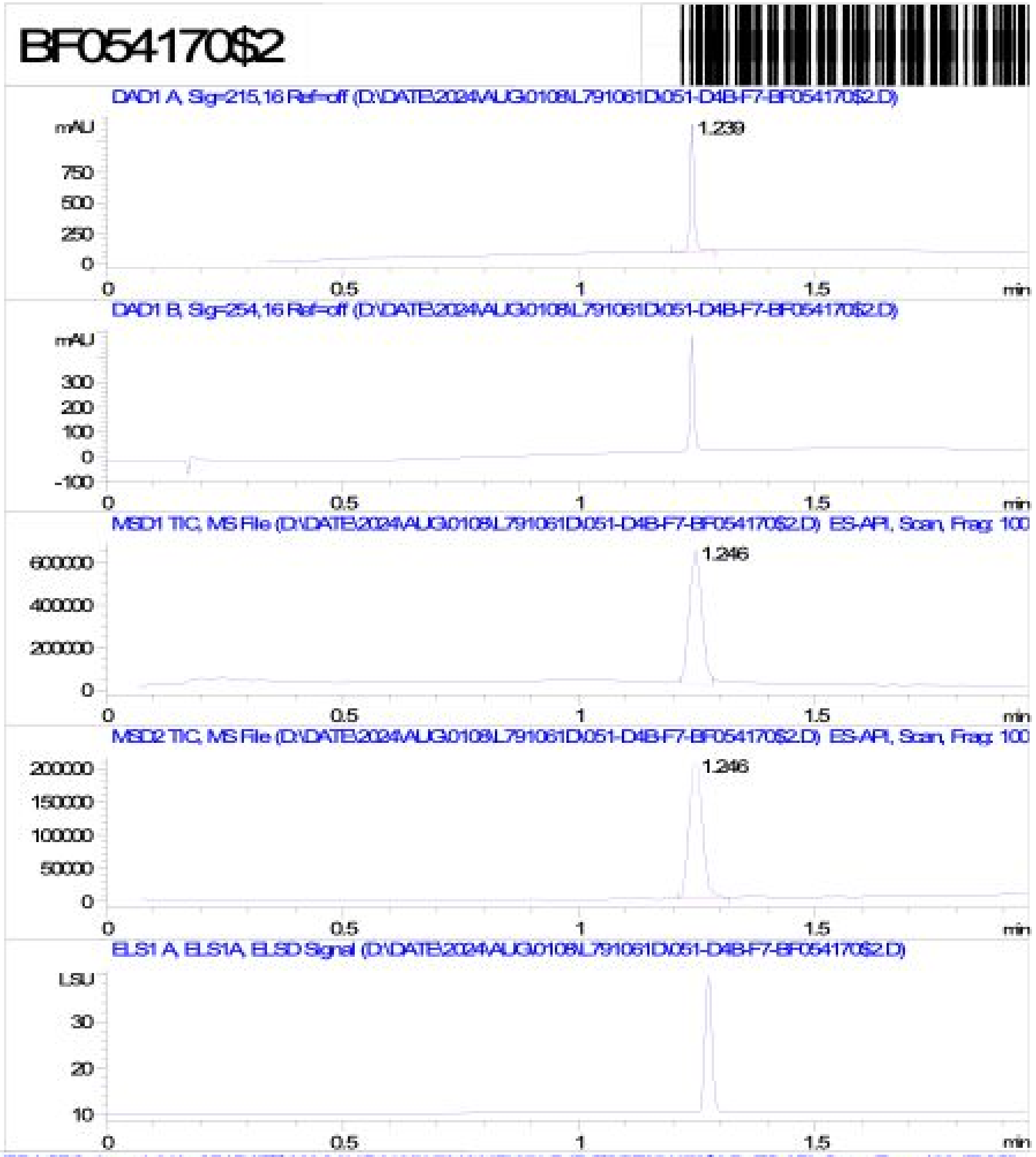

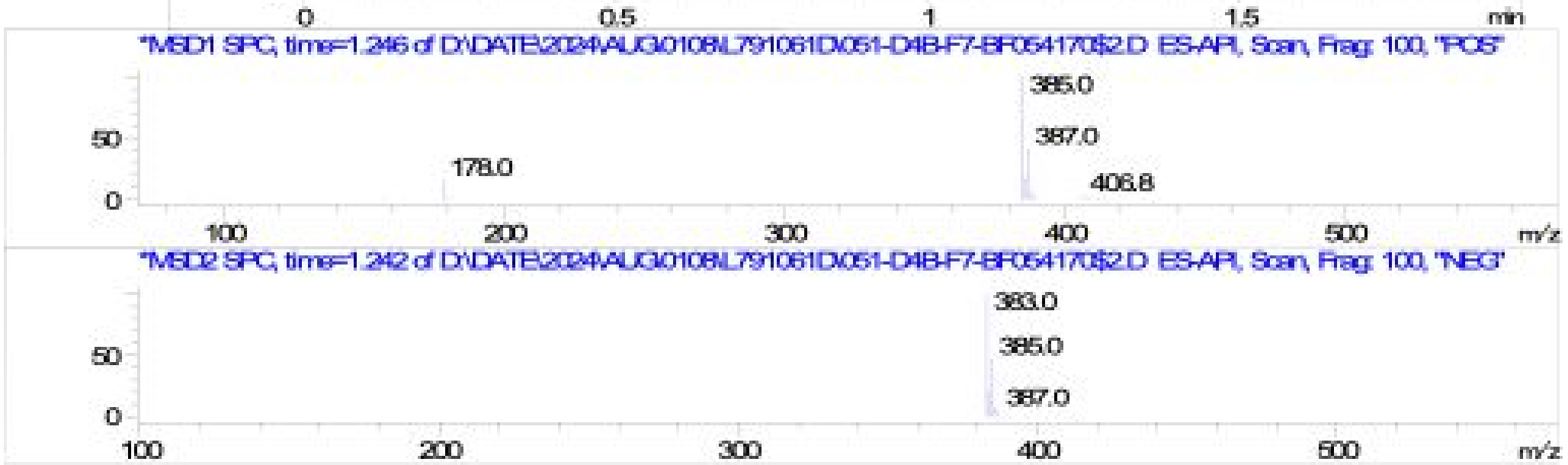

Inj.Date 7/31/2024 AN -15-
C:\Chem32\1\Data\07_31\L791061D\SUPOR_30.M

Z9212924966

MaxPeak: 97.69%
Ret_Time: 1.321 min

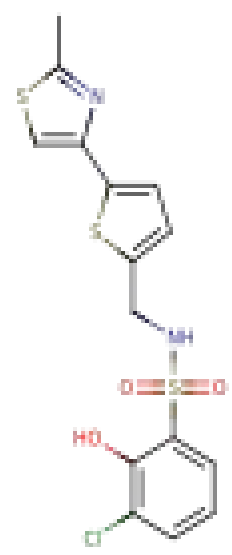

Mol Wt 400.92
Exact Mass 399.98

#	Time	Area%
1	1.321	97.69
2	1.361	2.31

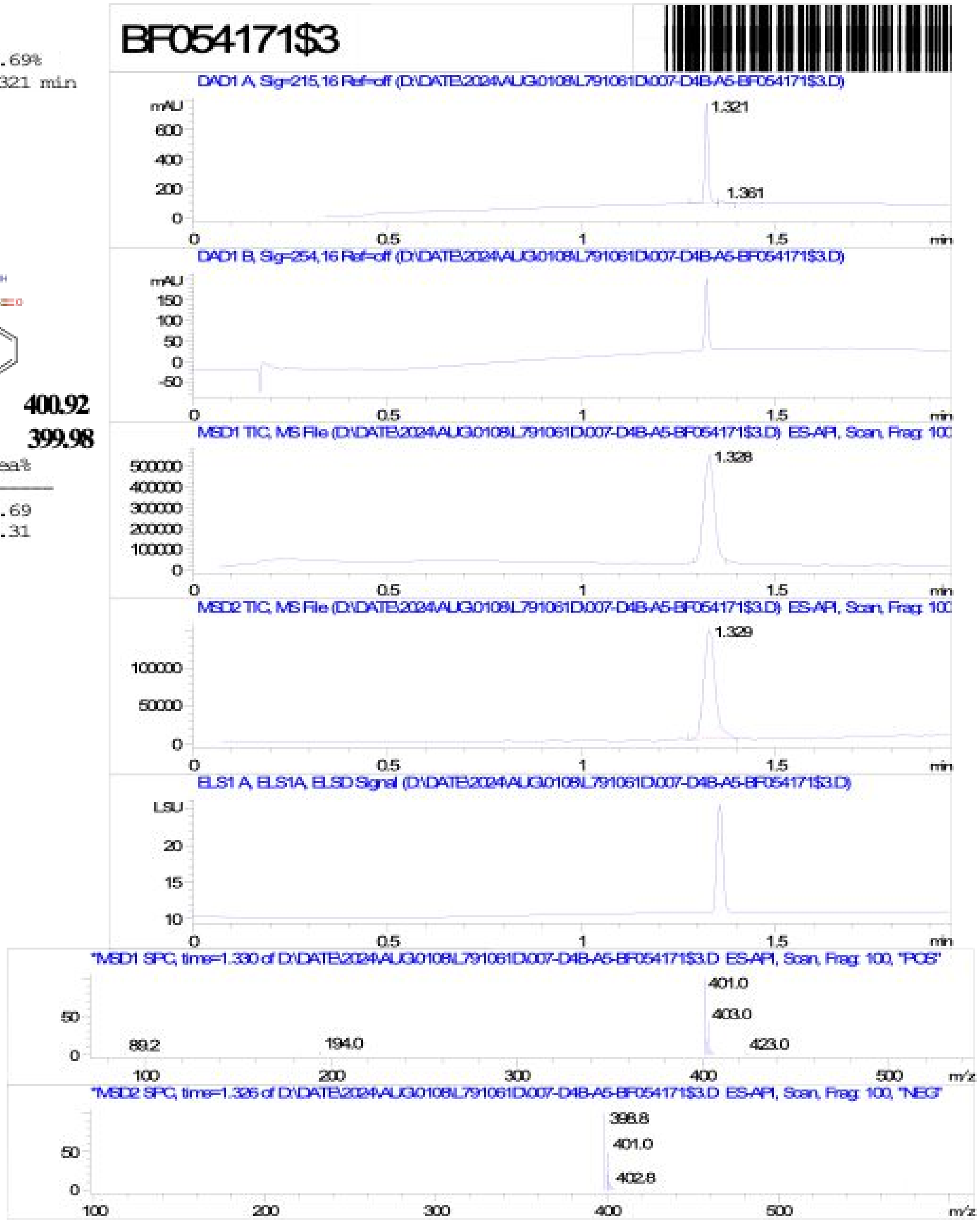

Inj.Date 7/31/2024 AN -15-
C:\Chem32\1\Data\07_31\L791061D\SUPOR_30.M

Z9212924967

MaxPeak: 94.74%
Ret_Time: 1.013 min

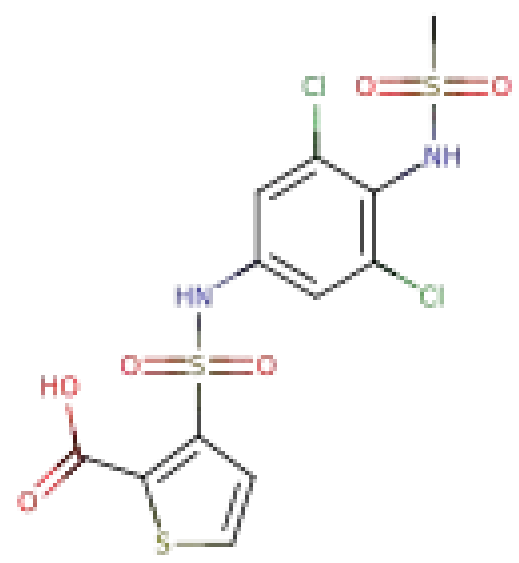

Mol Wt 445.32
Exact Mass 443.89

#	Time	Area%
1	0.913	2.06
2	0.987	2.23
3	1.013	94.74
4	1.274	0.98

BF054172$2

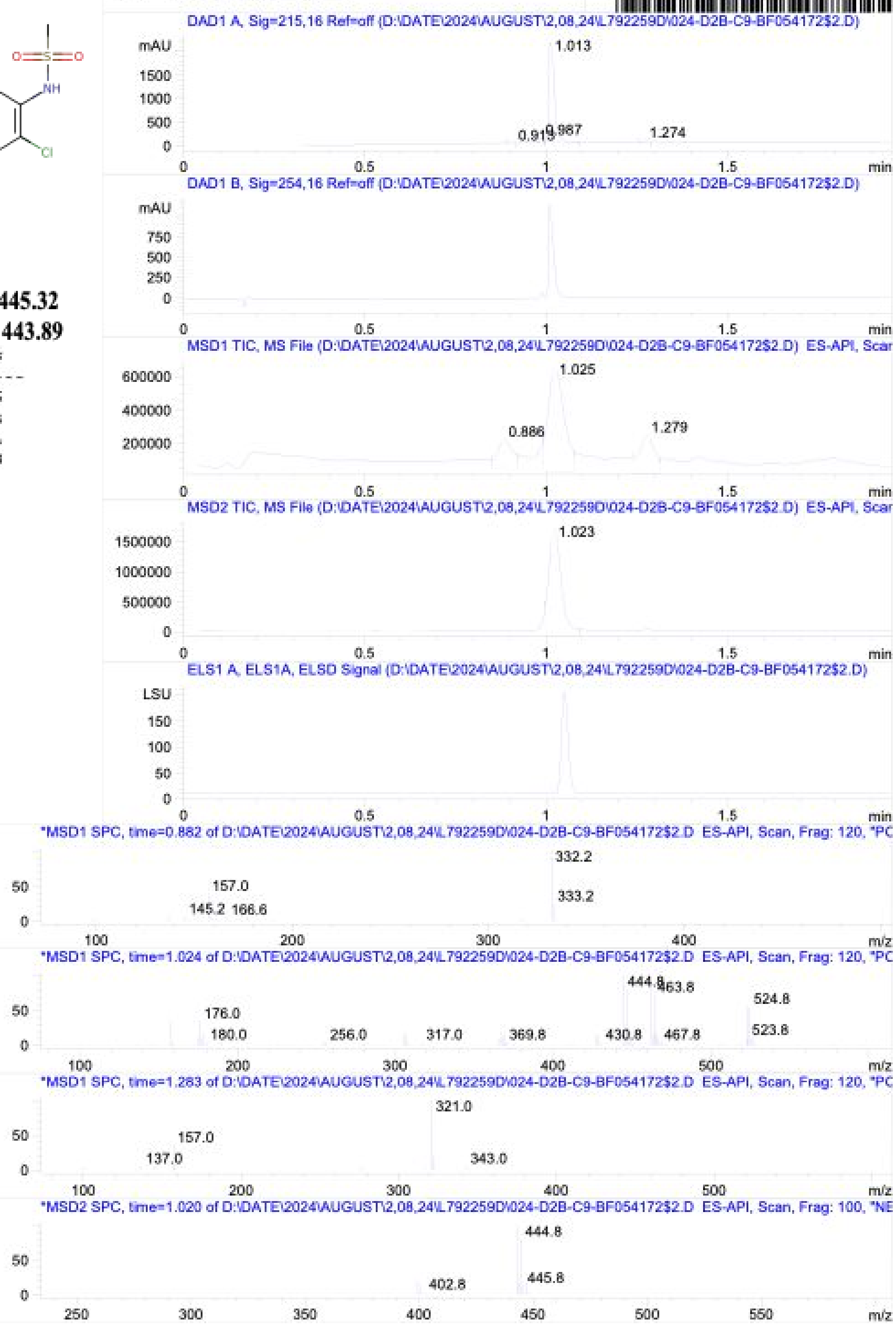

Inj.Date 8/2/2024 T -26- ->
C:\Users\Public\Documents\ChemStation\1\Data\08_02\L792259D\SUPOR_96.M

Z9212924969

MaxPeak: 100.00%
Ret_Time: 1.268 min

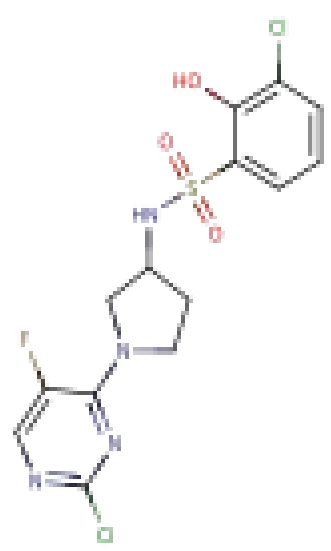

Mol Wt 407.25
Exact Mass 406.01

#	Time	Area%
1	1.268	100.00

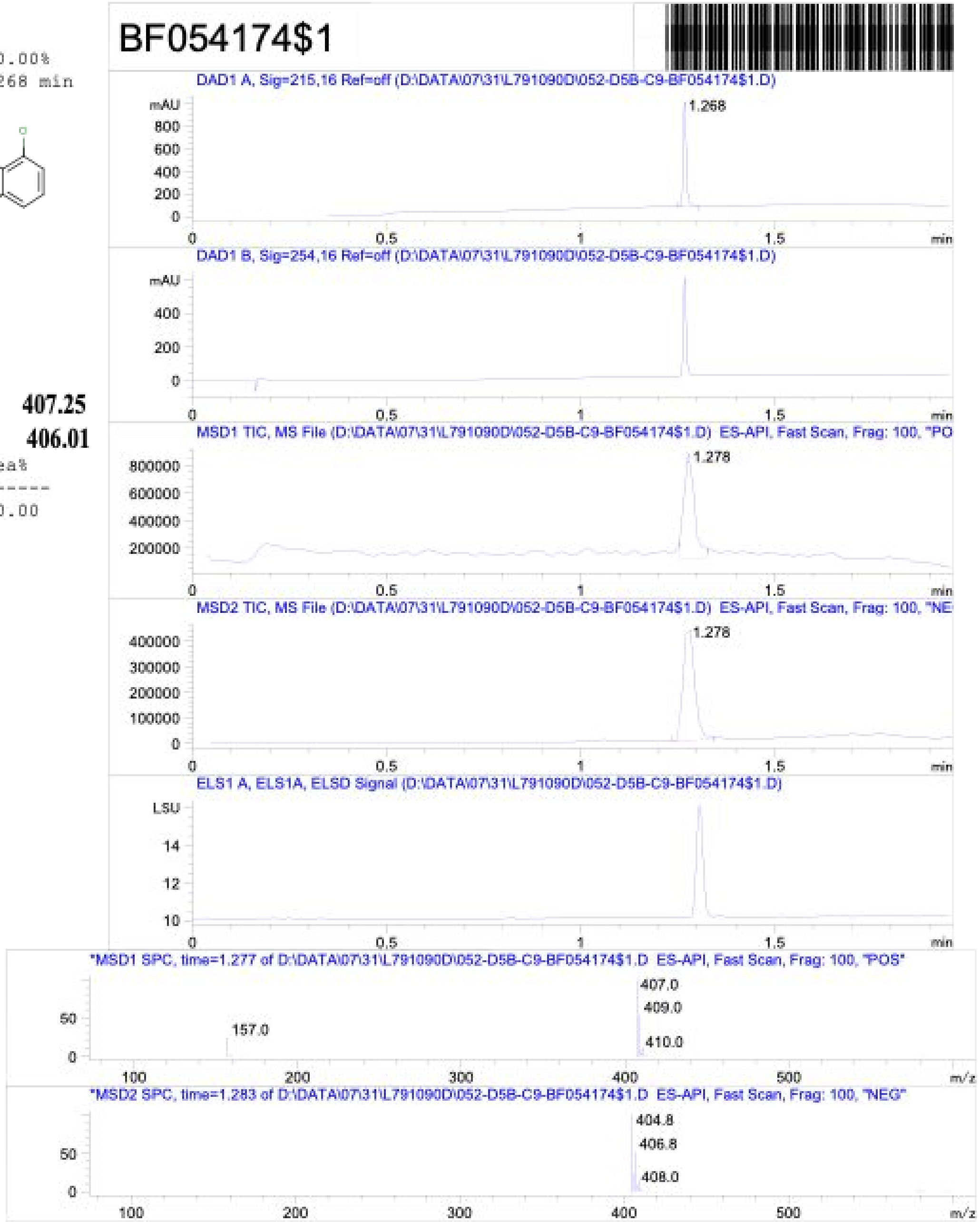

RT 1.278

RT 1.278

Inj.Date 7/31/2024 CH -19-
C:\Users\Public\Documents\ChemStation\1\Data\07_31\L791090D\SUPOR_30.M

Z9212924973

MaxPeak: 100.00%
Ret_Time: 1.388 min

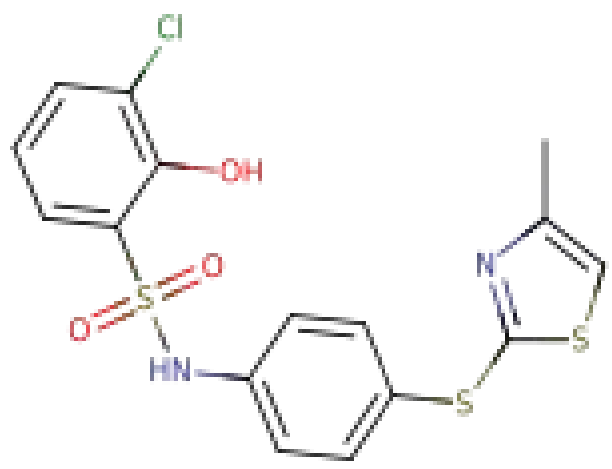

Mol Wt 412.93
Exact Mass 411.98

#	Time	Area%
1	1.388	100.00

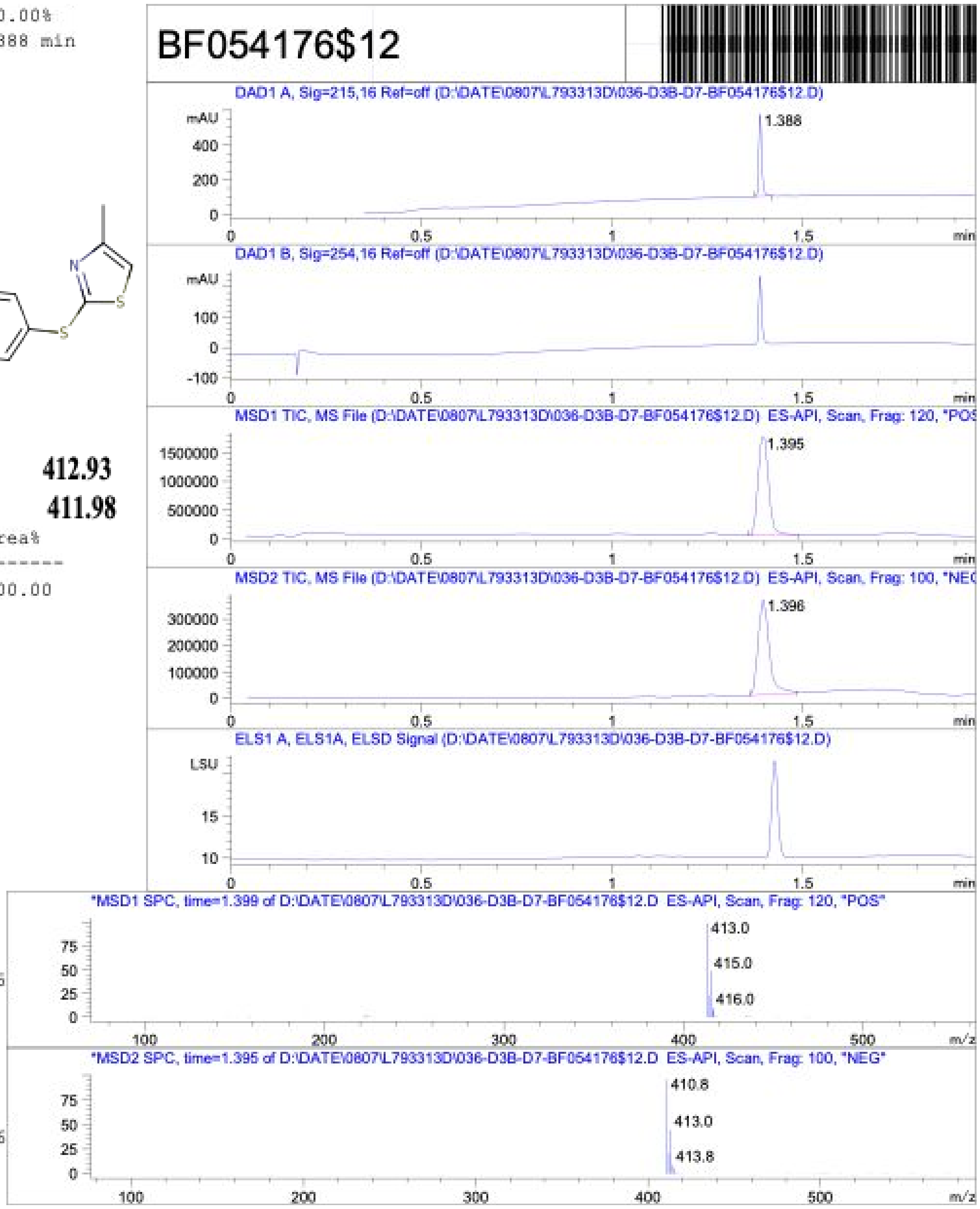

Inj.Date 8/6/2024 CH -26-
C:\Users\Public\Documents\ChemStation\1\Data\08_06\L793313D\SUPOR_30.M

Z9212924975

MaxPeak: 100.00%
Ret_Time: 1.169 min

BF054178$2

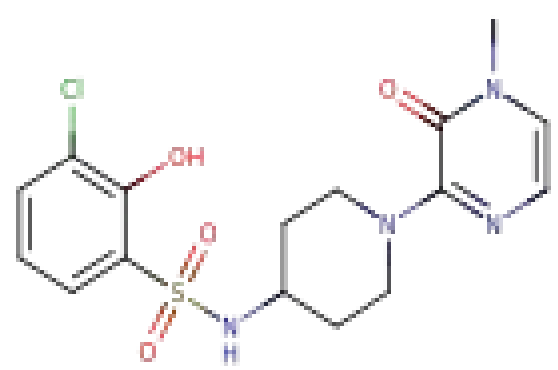

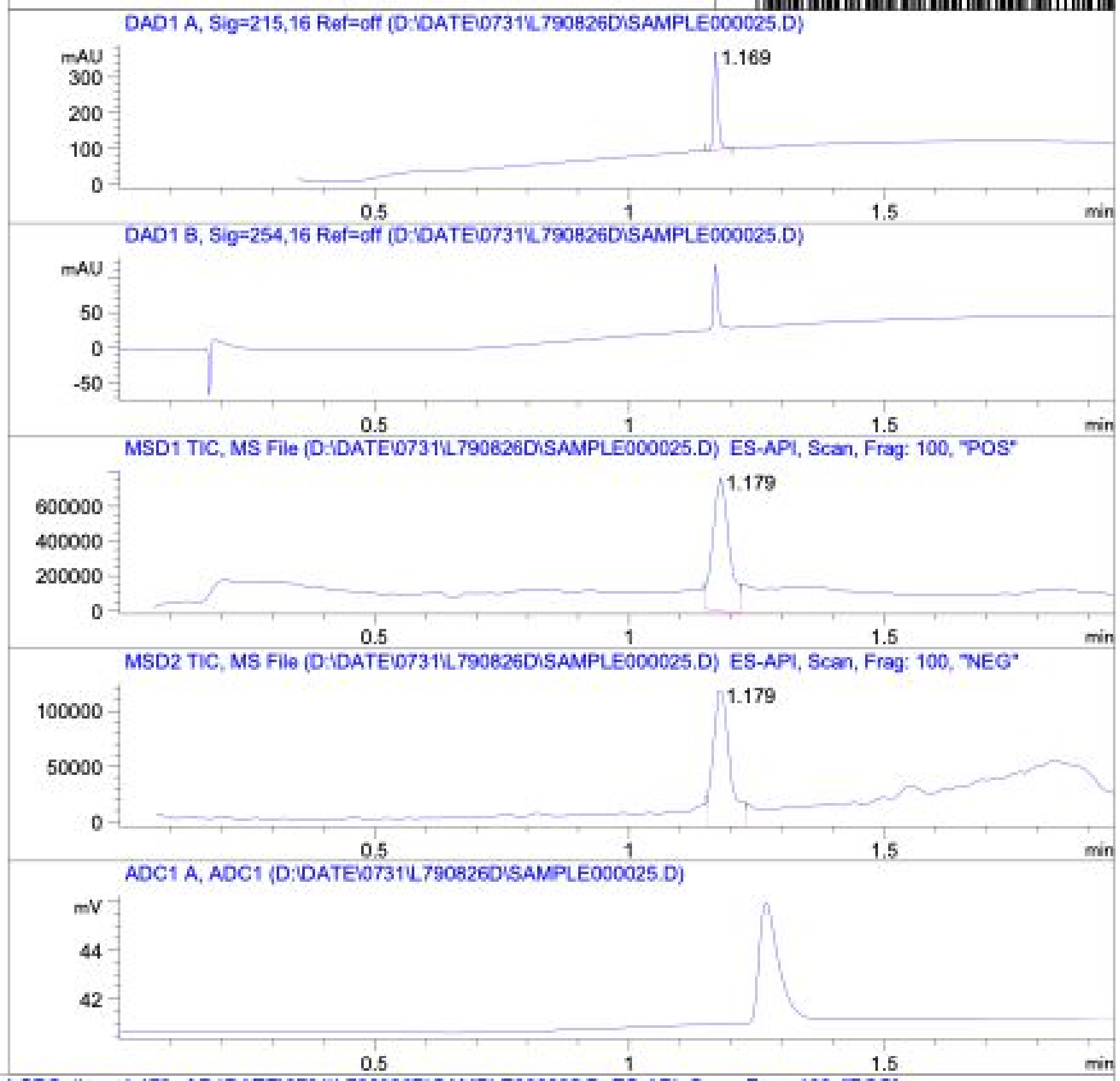

Mol Wt 398.86

Exact Mass 398.09

#	Time	Area%
1	1.169	100.00

RT 1.179

RT 1.179

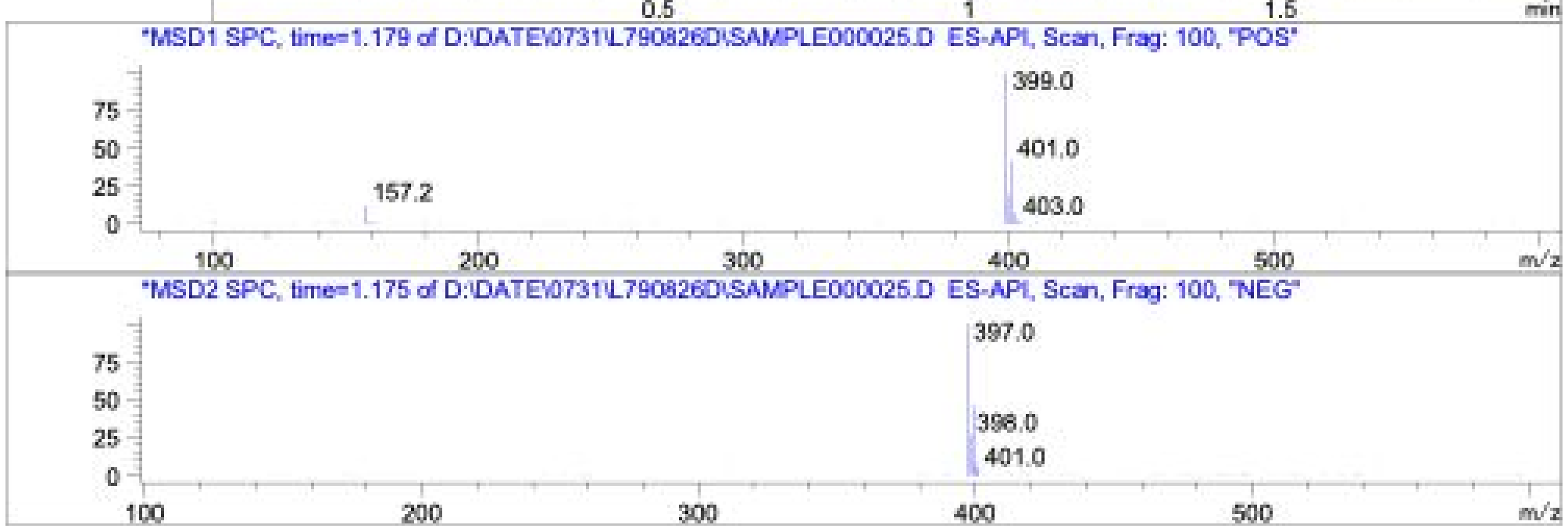

Inj.Date 7/31/2024 E - 4 -
C:\CHEM32\1\METHODS\SUPOR_30.M

Z9212924980

MaxPeak: 98.66%
Ret_Time: 1.293 min

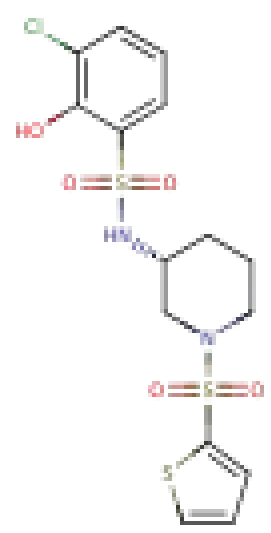

Mol Wt 436.95
Exact Mass 436

#	Time	Area%
1	1.293	98.66
2	1.313	1.34

BF054179$1

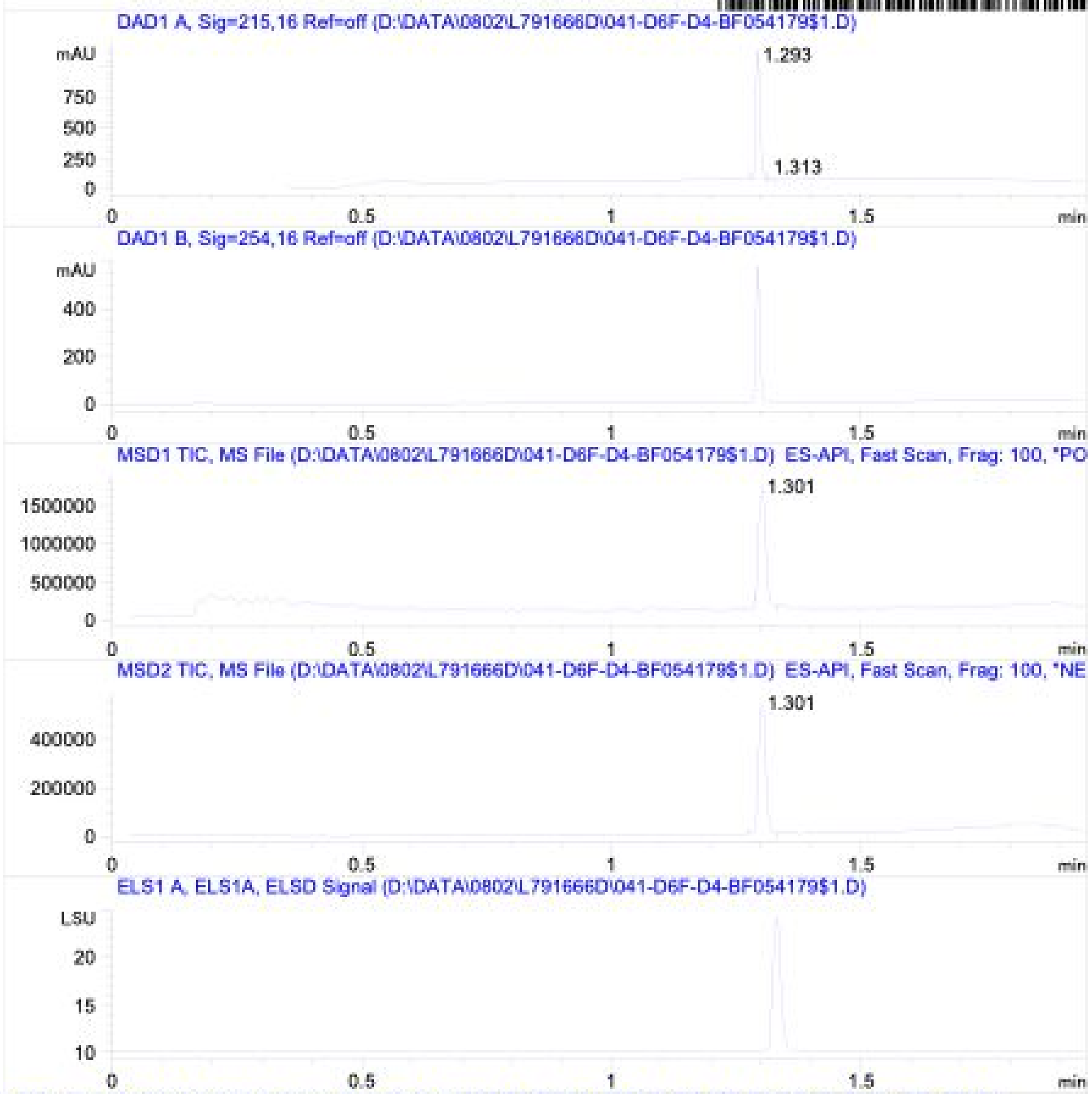

RT 1.301

RT 1.301

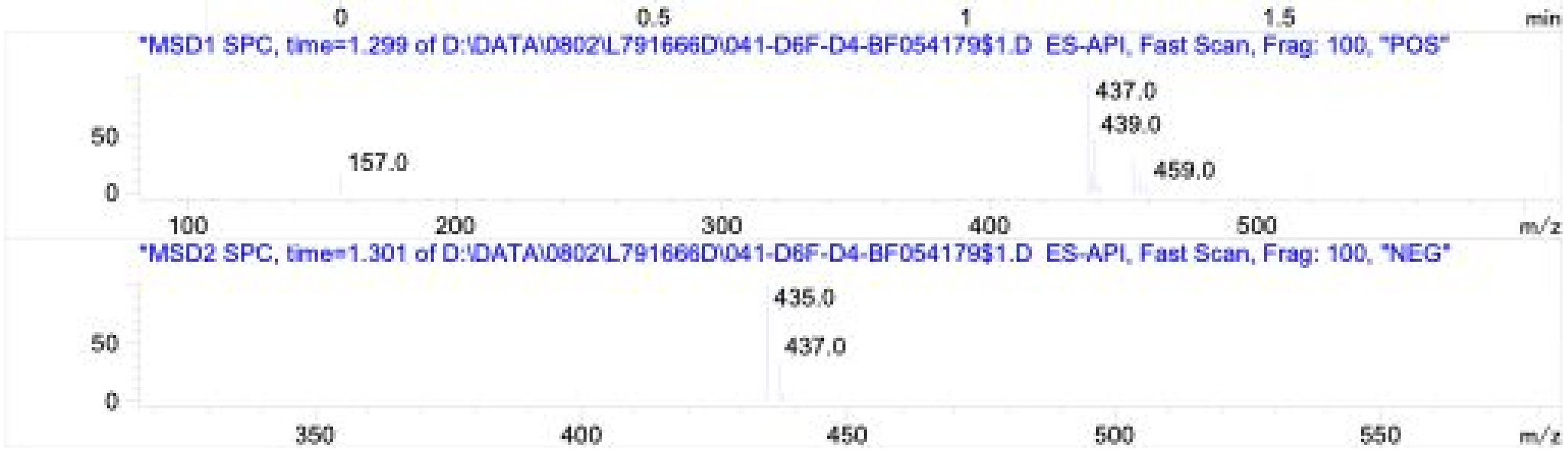

Inj.Date 8/1/2024 H
C:\Users\Public\Documents\ChemStation\1\Data\08_01\L791666D\SUPOR_30.M

Z9212924983

MaxPeak: 100.00%
Ret_Time: 1.323 min

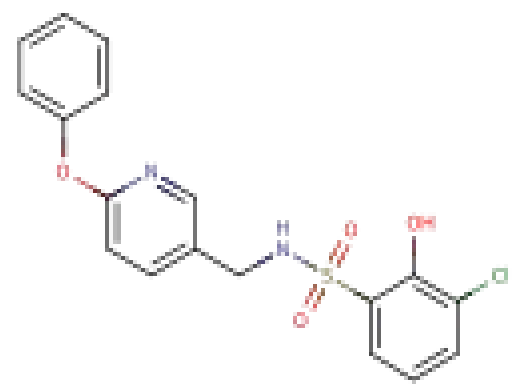

Mol Wt 390.84
Exact Mass 390.05

#	Time	Area%
1	1.323	100.00

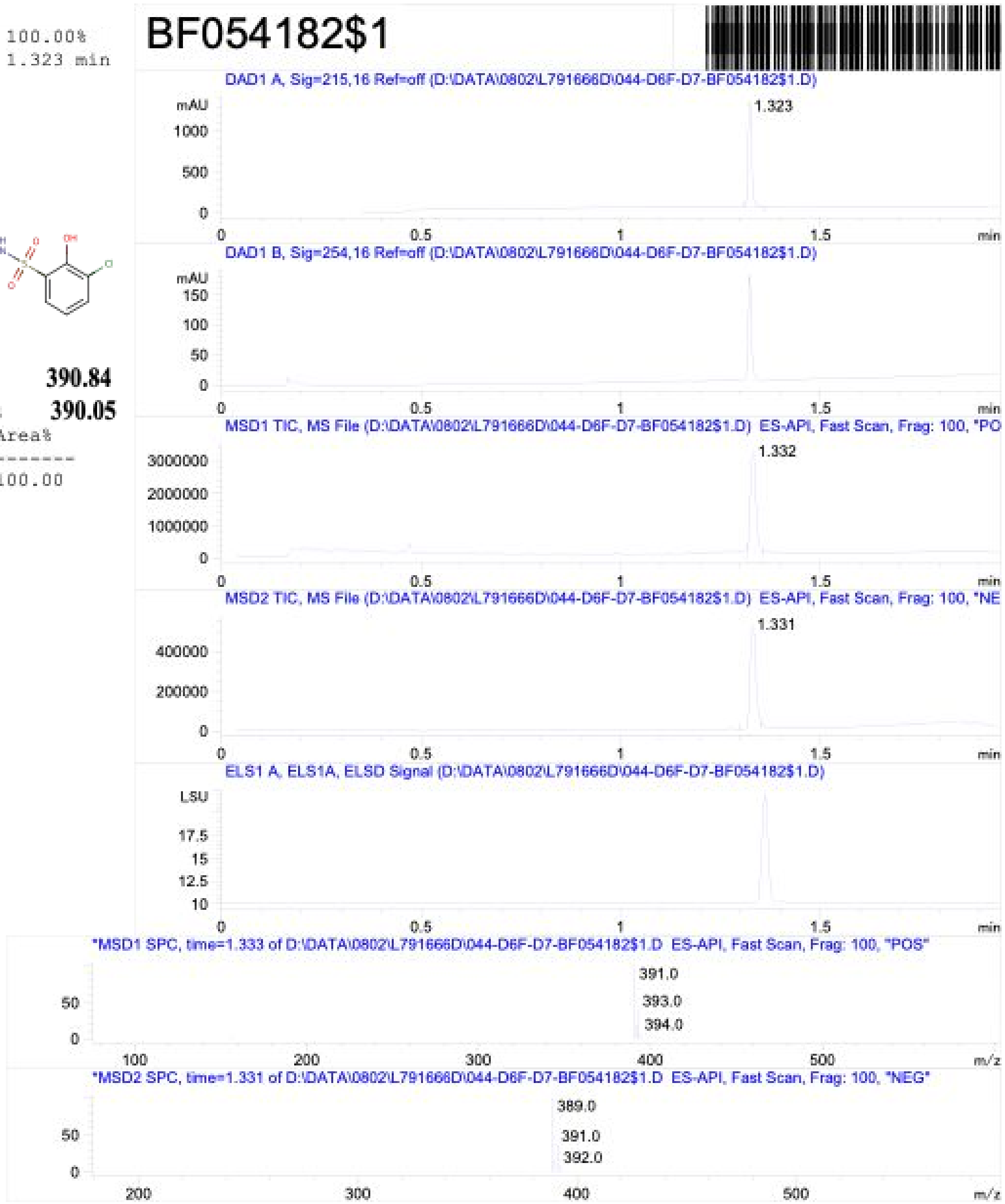

RT 1.332

RT 1.331

Inj.Date 8/1/2024 H
C:\Users\Public\Documents\ChemStation\1\Data\08_01\L791666D\SUPOR_30.M

Z9212924996

MaxPeak: 100.00%
Ret_Time: 1.204 min

BF054183$1

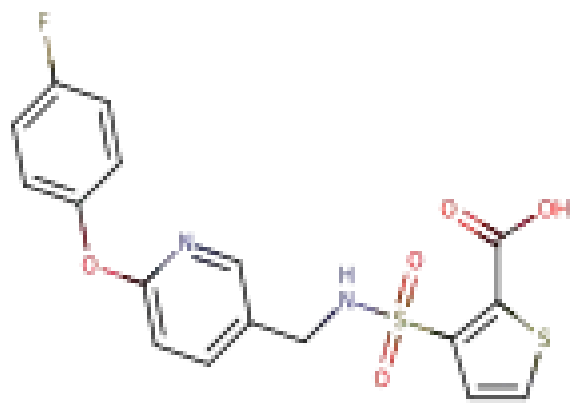

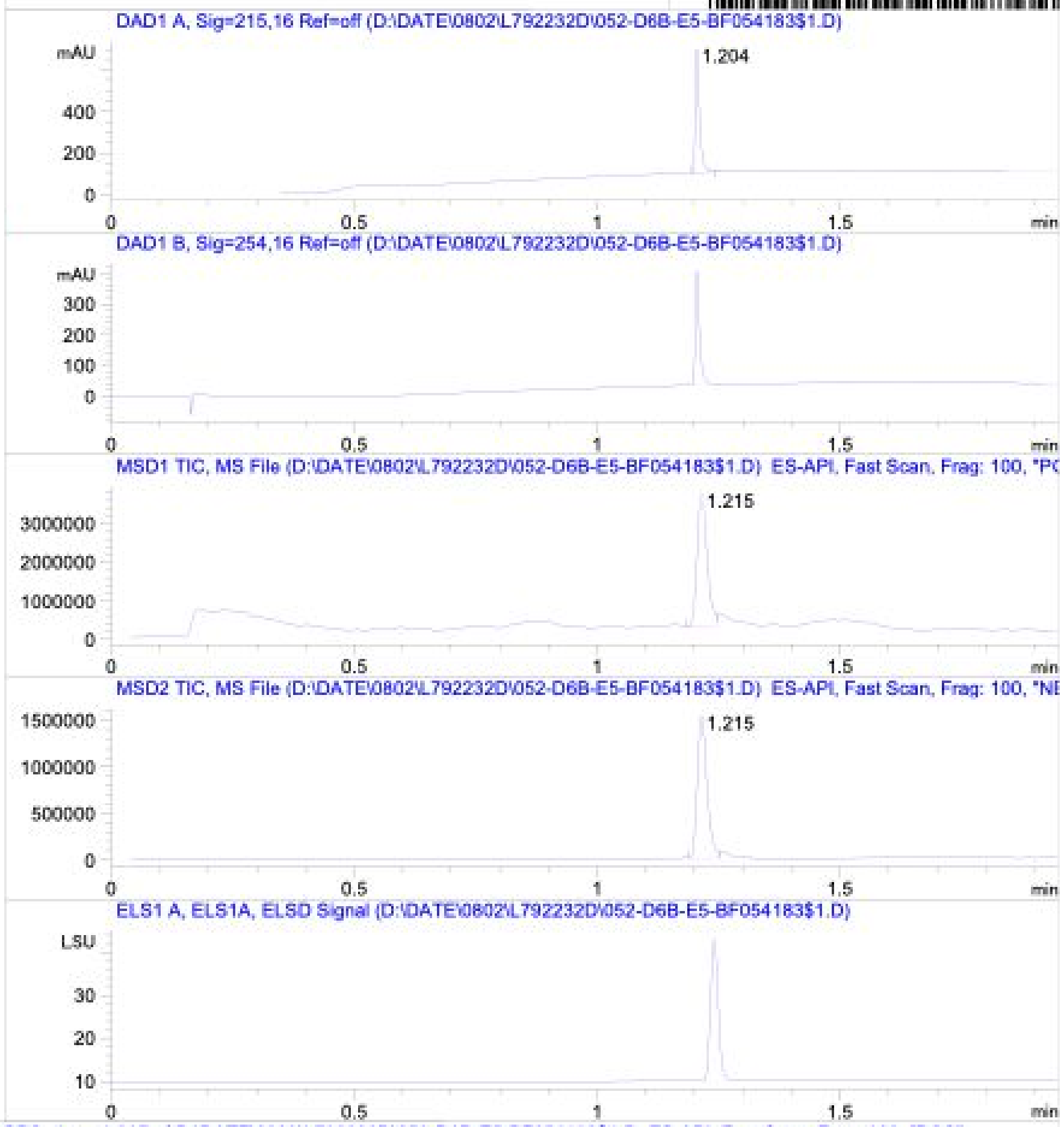

Mol Wt 408.42

Exact Mass 408.02

#	Time	Area%
1	1.204	100.00

RT 1.215

RT 1.215

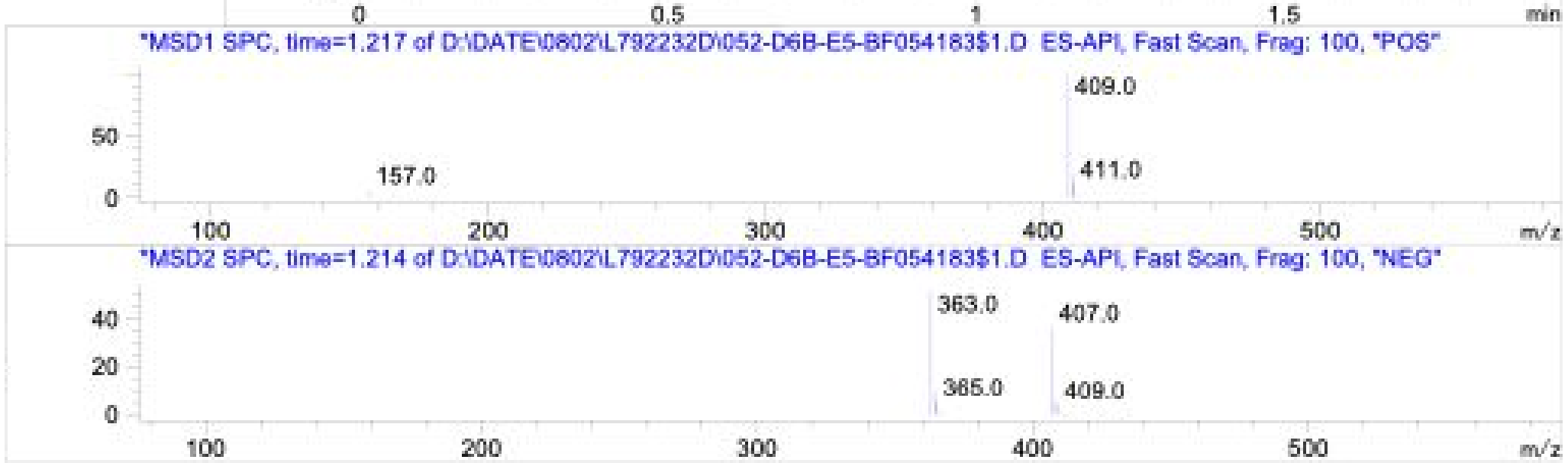

Inj.Date 8/2/2024 O 29
C:\Users\Public\Documents\ChemStation\1\Data\08_02\L792232D\SUPOR_30.M

Z9212924999

MaxPeak: 100.00%
Ret_Time: 1.416 min

BF054184$1

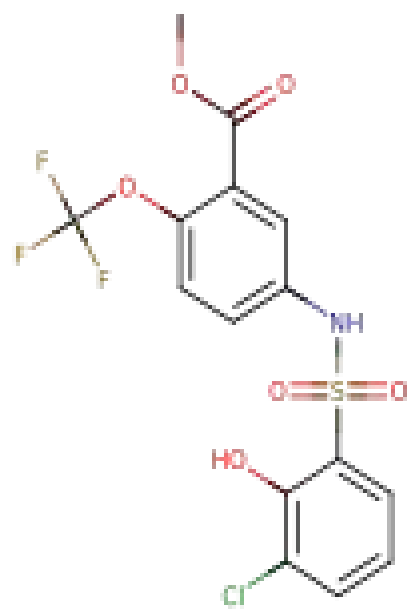

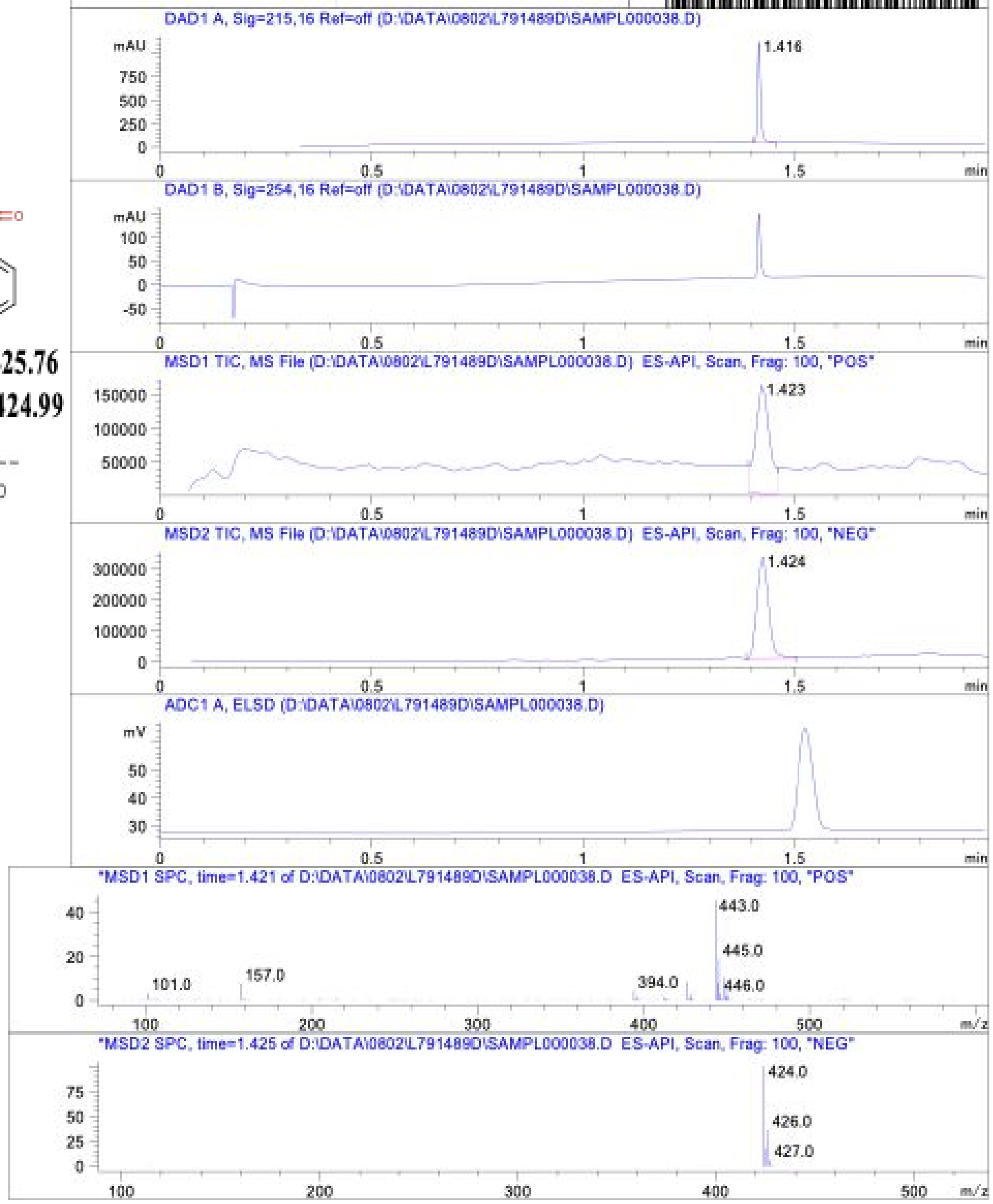

Mol Wt 425.76

Exact Mass 424.99

#	Time	Area%
1	1.416	100.00

RT 1.423

RT 1.424

Inj.Date 8/1/2024 LB -6-
C:\CHEM32\1\METHODS\SUPOR_30.M

Z9212925003

MaxPeak: 100.00%
Ret_Time: 1.463 min

BF054185$2

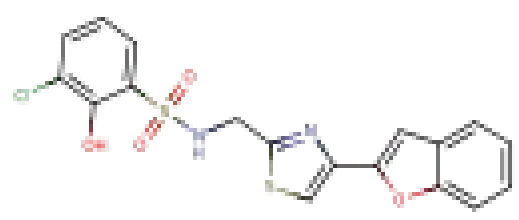

Mol Wt 420.89
Exact Mass 420

#	Time	Area%
1	1.463	100.00

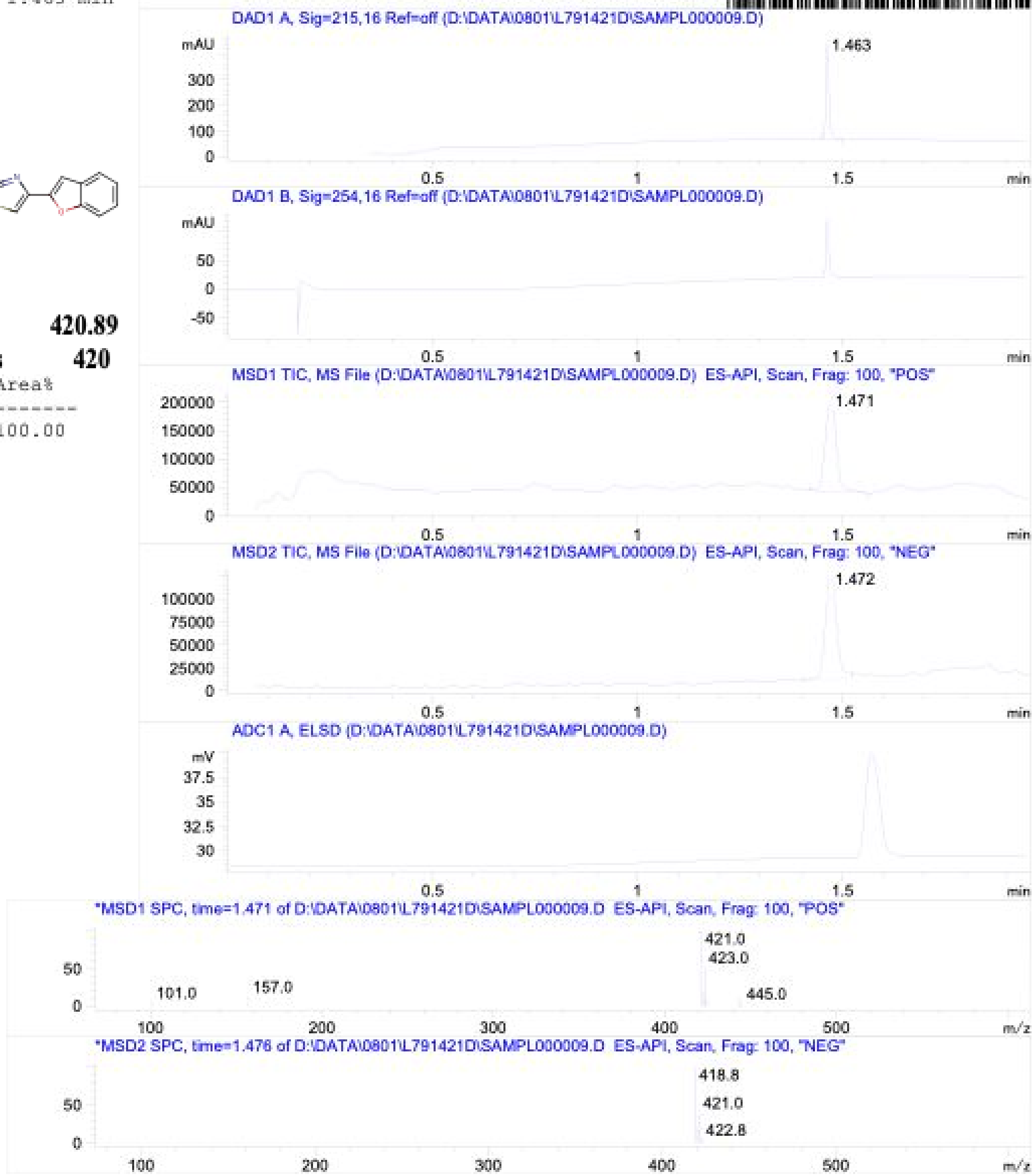

RT 1.471

RT 1.472

Inj.Date 8/1/2024 H -6-
C:\CHEM32\1\METHODS\SUPOR_30.M

Z9212925005

MaxPeak: 100.00%
Ret_Time: 1.269 min

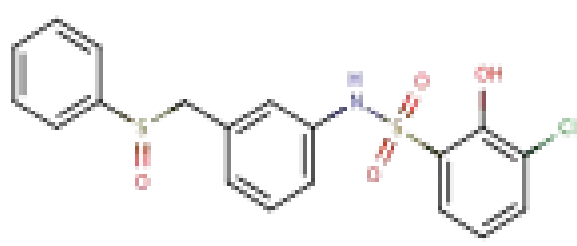

Mol Wt 421.92
Exact Mass 421.03

#	Time	Area%
1	1.269	100.00

BF054186$2

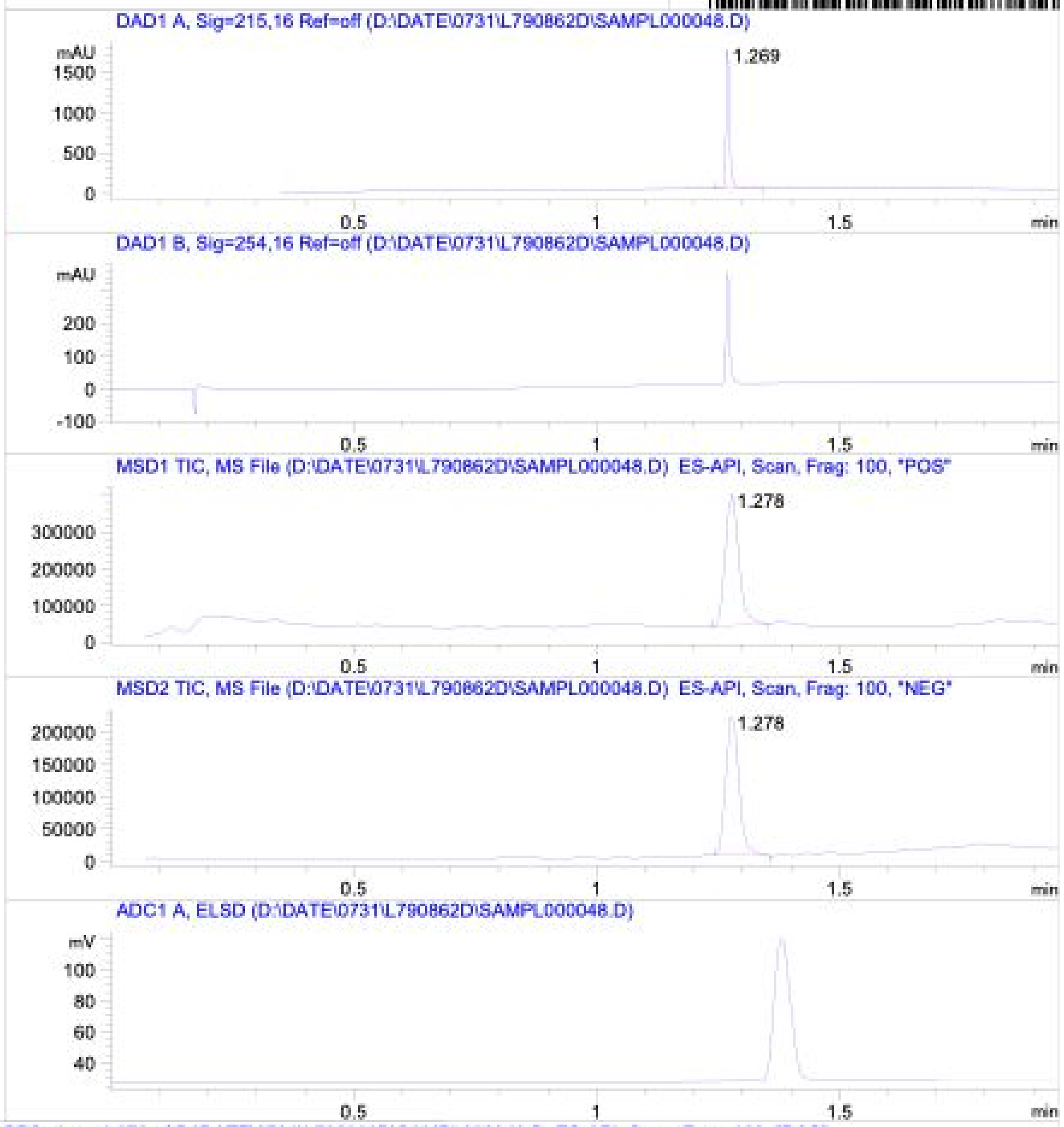

RT 1.278

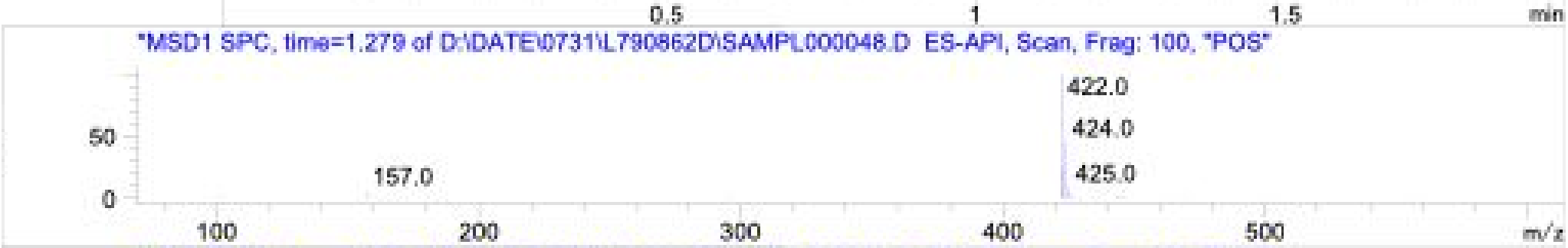

RT 1.278

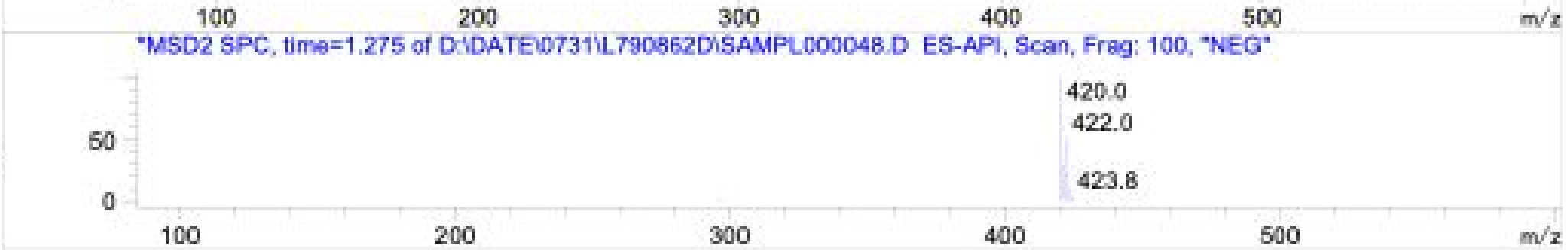

Inj.Date 7/31/2024 O -6-
C:\CHEM32\1\METHODS\SUPOR_30.M

Z9212925007

MaxPeak: 100.00%
Ret_Time: 1.265 min

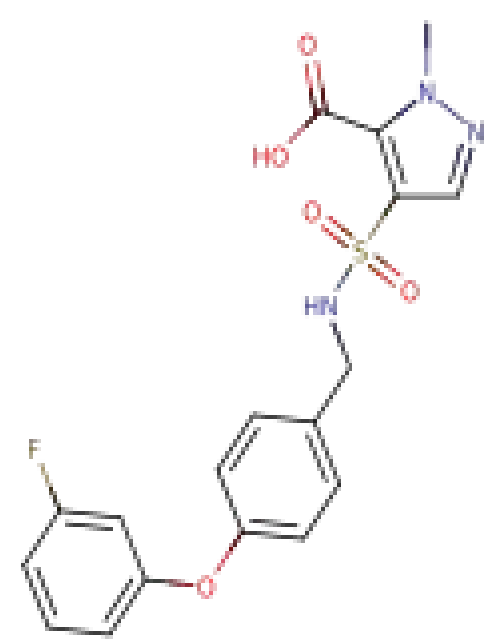

Mol Wt 405.4
Exact Mass 405.08

#	Time	Area%
1	1.265	100.00

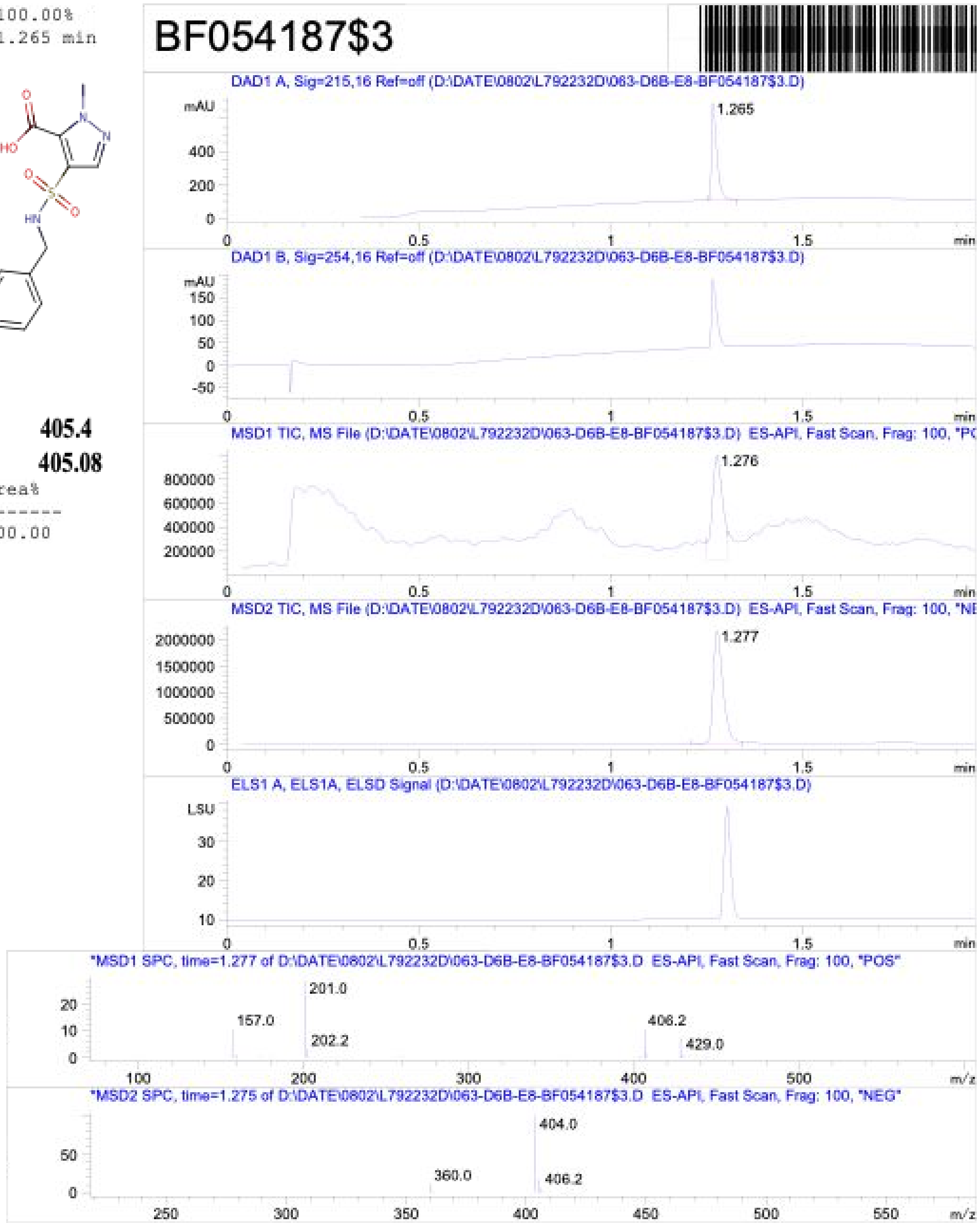

RT 1.276

RT 1.277

Inj.Date 8/2/2024 O 29
C:\Users\Public\Documents\ChemStation\1\Data\08_02\L792232D\SUPOR_30.M

Z9212925009

MaxPeak: 100.00%
Ret_Time: 1.077 min

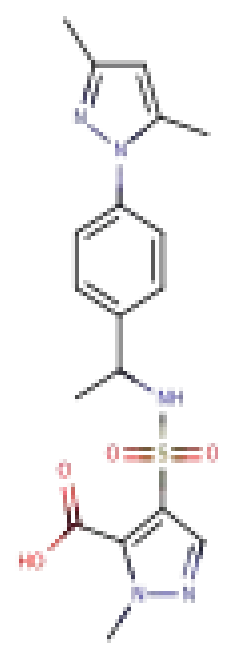

Mol Wt 403.46
Exact Mass 403.14

#	Time	Area%
1	1.077	100.00

BF054188$4

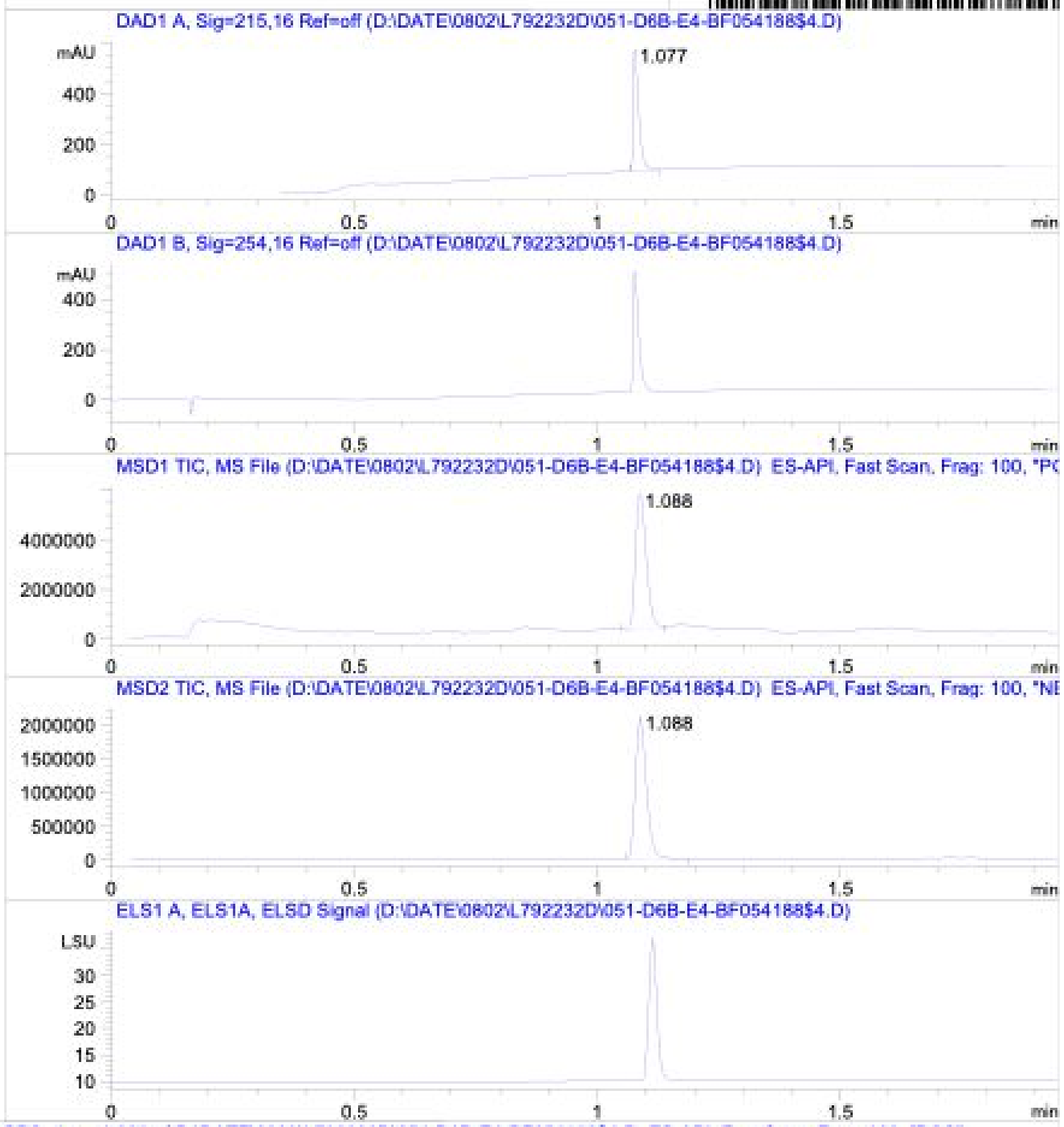

RT 1.088

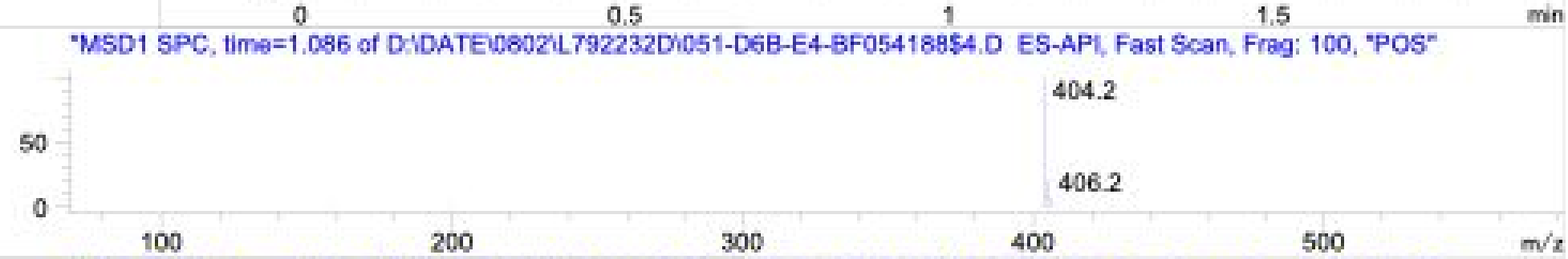

RT 1.088

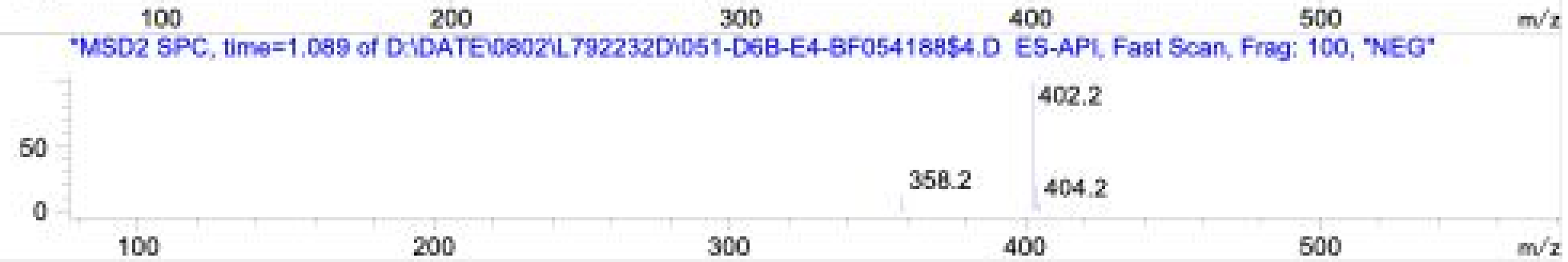

Inj.Date 8/2/2024 O 29
C:\Users\Public\Documents\ChemStation\1\Data\08_02\L792232D\SUPOR_30.M

Z9212925012

MaxPeak: 100.00%
Ret_Time: 1.119 min

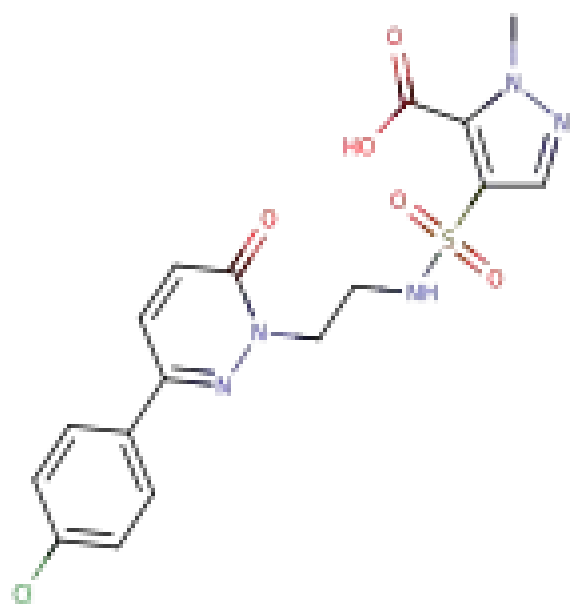

Mol Wt 437.86
Exact Mass 437.05

#	Time	Area%
1	1.119	100.00

RT 1.132

RT 1.131

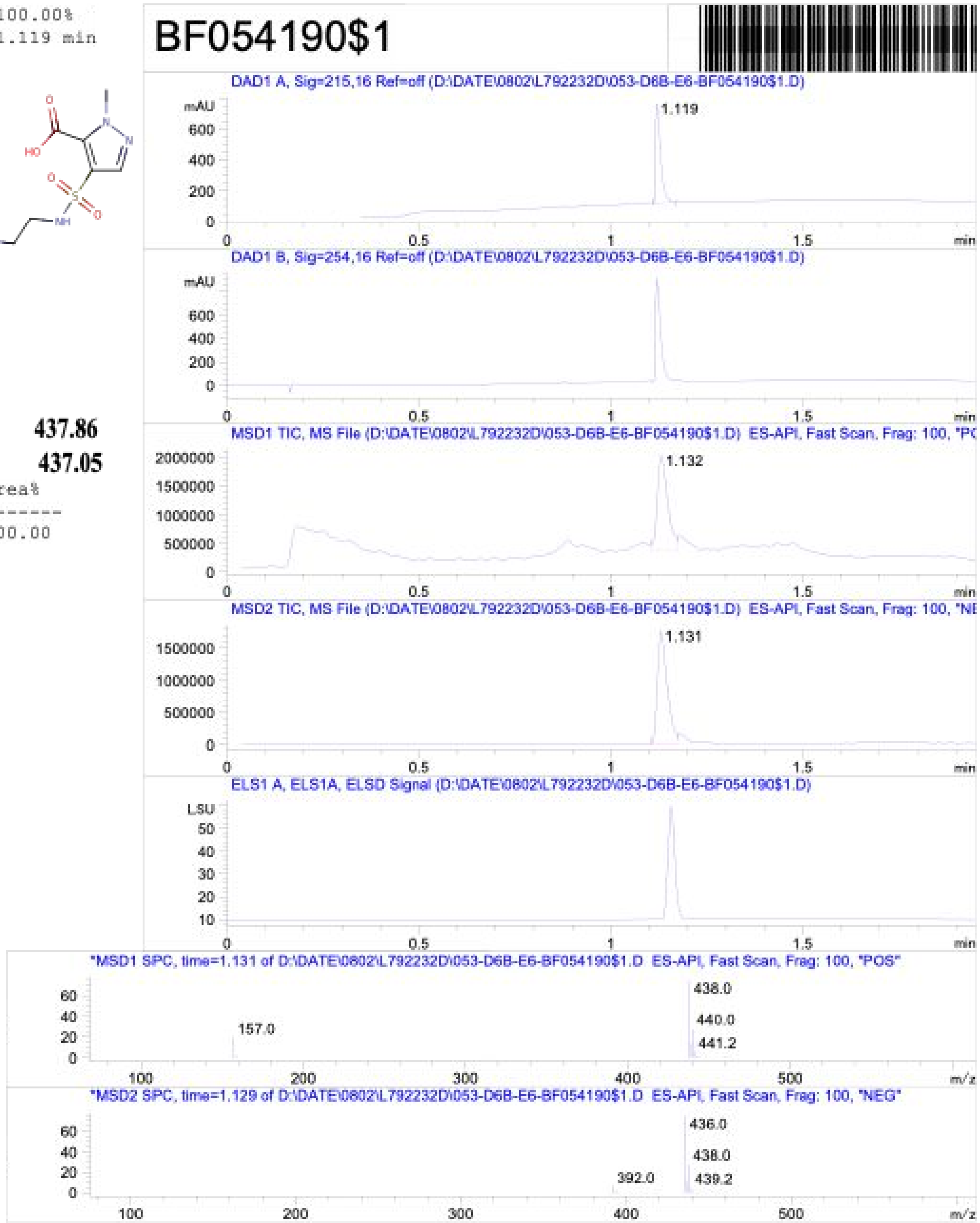

Inj.Date 8/2/2024 O 29
C:\Users\Public\Documents\ChemStation\1\Data\08_02\L792232D\SUPOR_30.M

Z9212925015

MaxPeak: 100.00%
Ret_Time: 1.291 min

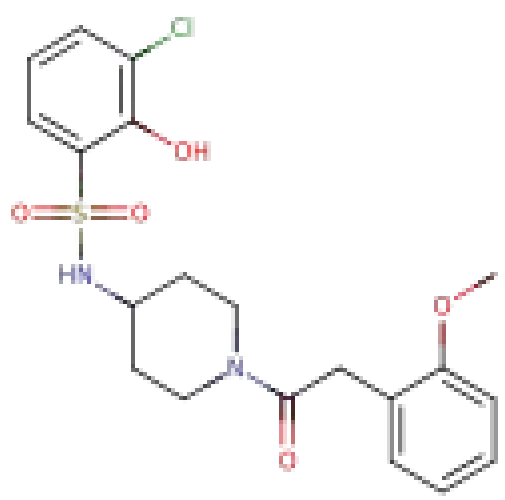

Mol Wt 438.92
Exact Mass 438.12

#	Time	Area%
1	1.291	100.00

RT 1.302

RT 1.299

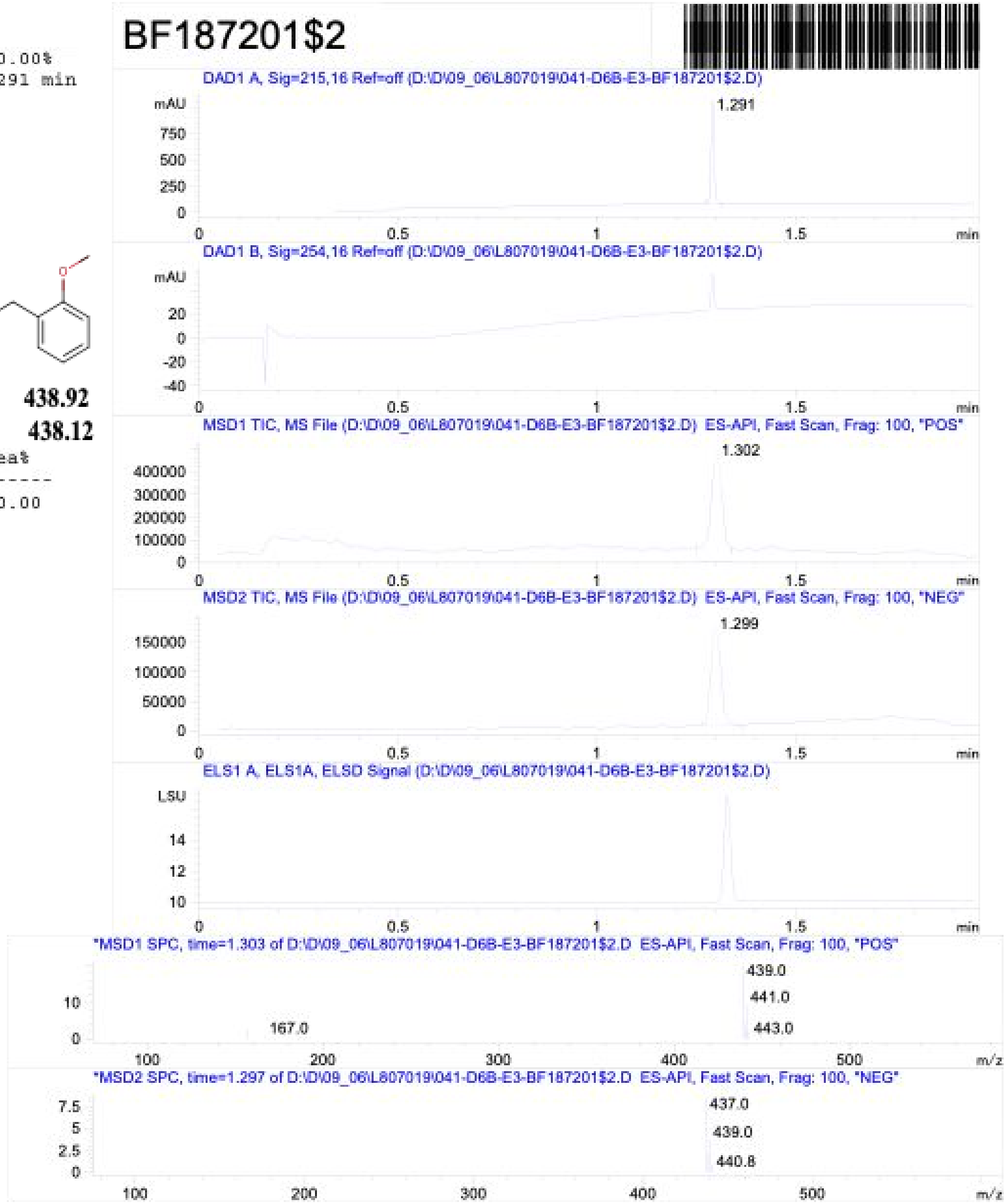

Inj.Date 9/6/2024 N 20
C:\Users\Public\Documents\ChemStation\1\Data\09_05\L807019\SUPOR_30.M

Z9212925018

MaxPeak: 97.12%
Ret_Time: 1.254 min

BG710967$1

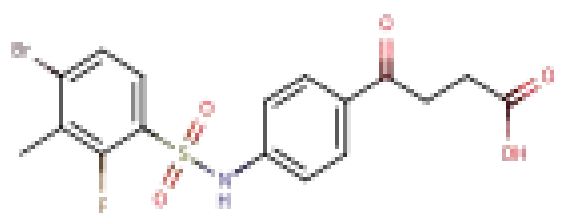

Mol Wt 444.27
Exact Mass 444.99

#	Time	Area%
1	1.149	1.19
2	1.254	97.12
3	1.783	1.69

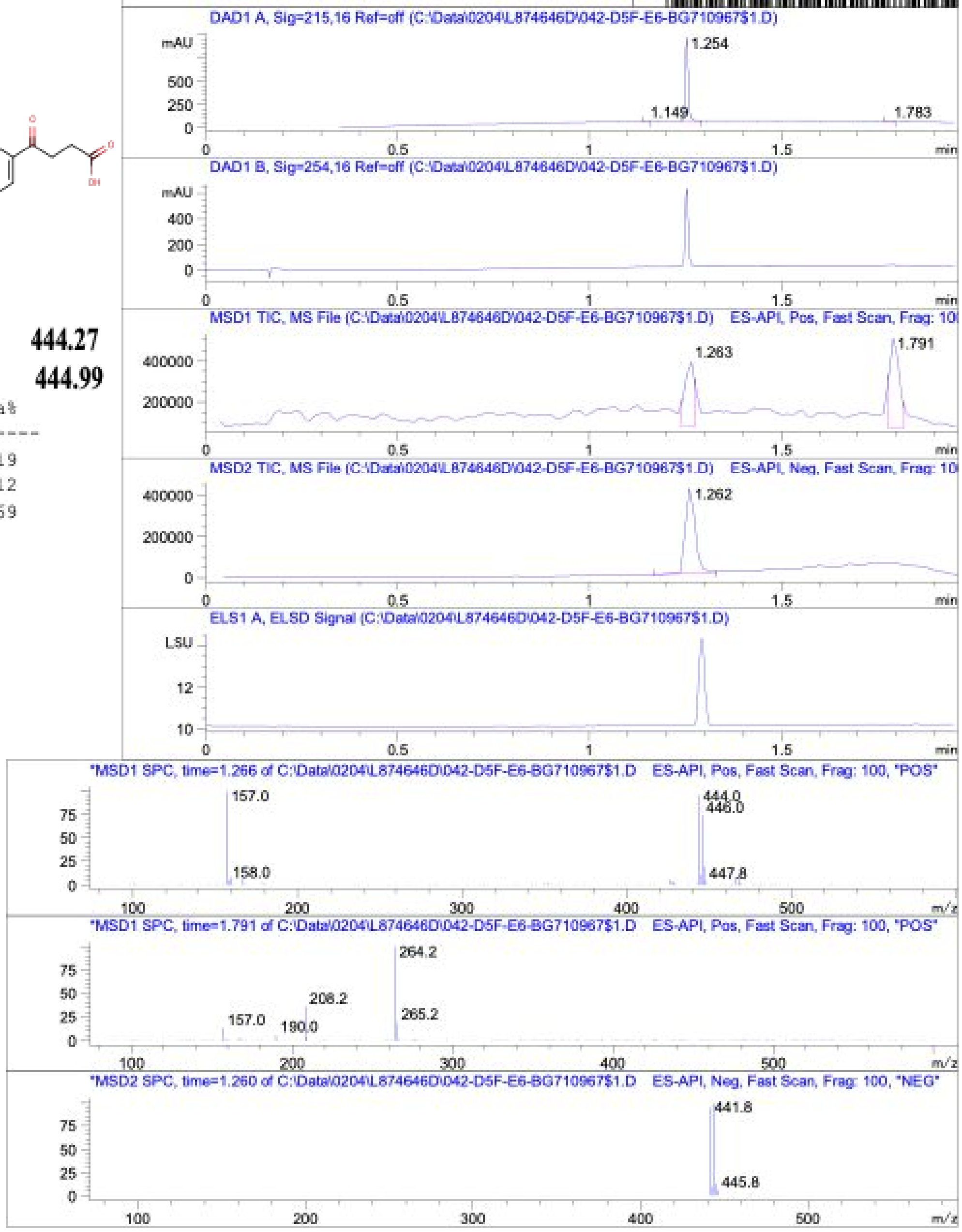

Inj.Date 2/3/2025 NEW
C:\Users\Public\Documents\ChemStation\1\Data\02_03\L874646D\SUPOR_30.M

Z9464814789

MaxPeak: 100.00%
Ret_Time: 1.098 min

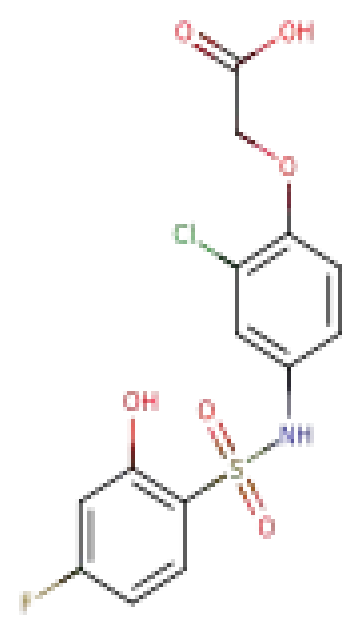

Mol Wt 375.76

Exact Mass 374.99

#	Time	Area%
1	1.098	100.00

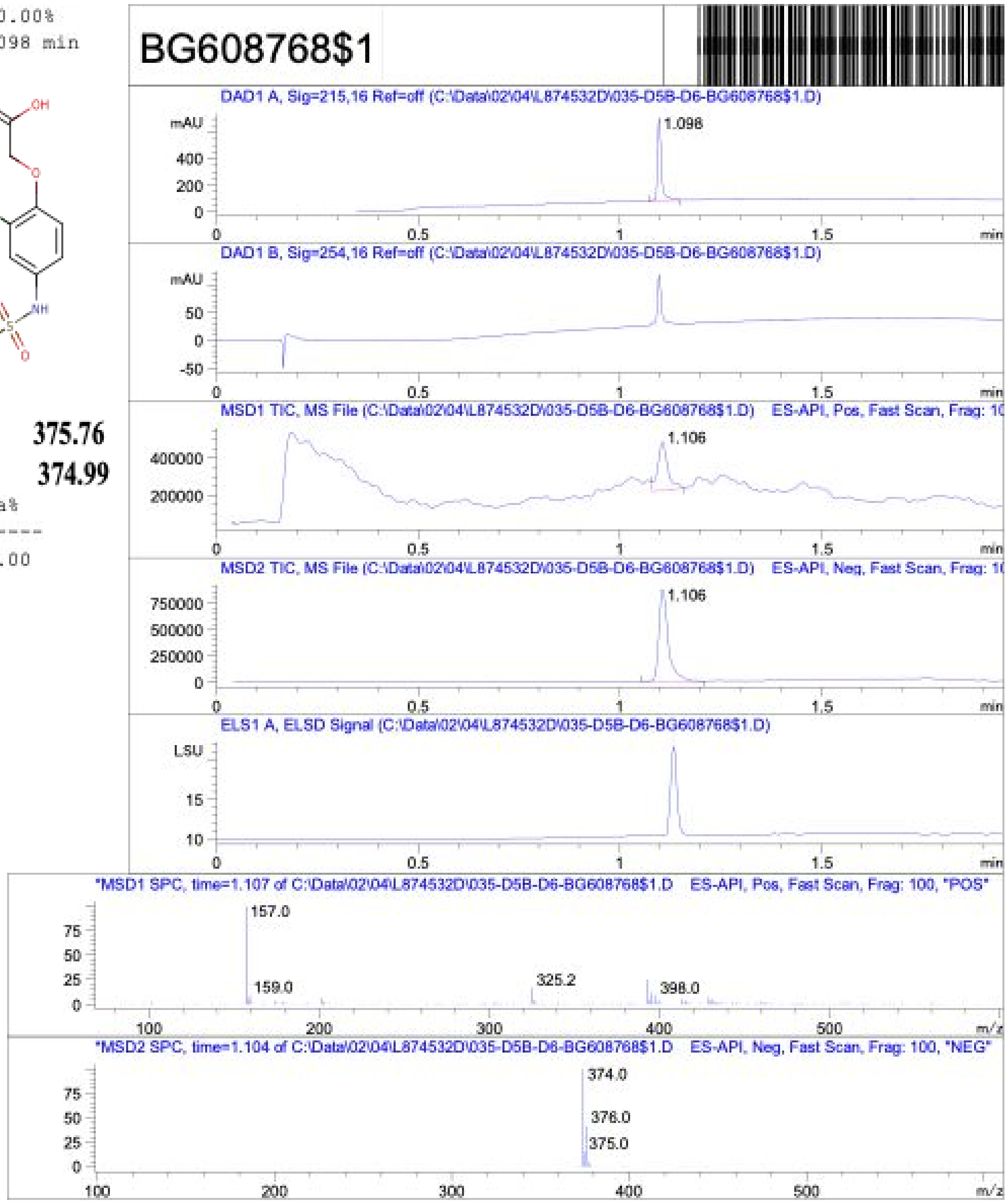

RT 1.106

RT 1.106

Inj.Date 2/4/2025 C -29-
C:\Users\Public\Documents\ChemStation\1\Data\02_03\L874532D\SUPOR_30.M

Z9464814798

MaxPeak: 98.64%
Ret_Time: 1.382 min

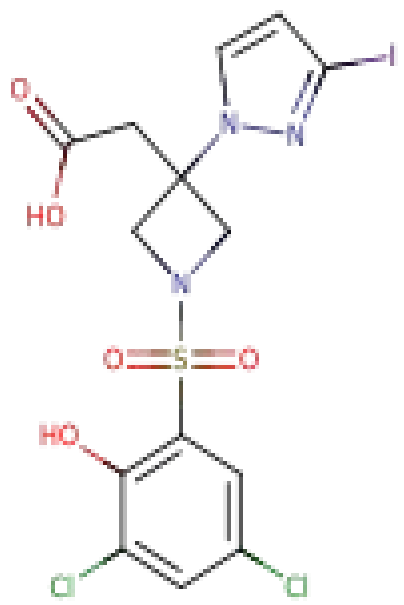

Mol Wt 532.14

Exact Mass 530.88

#	Time	Area%
1	1.369	1.36
2	1.382	98.64

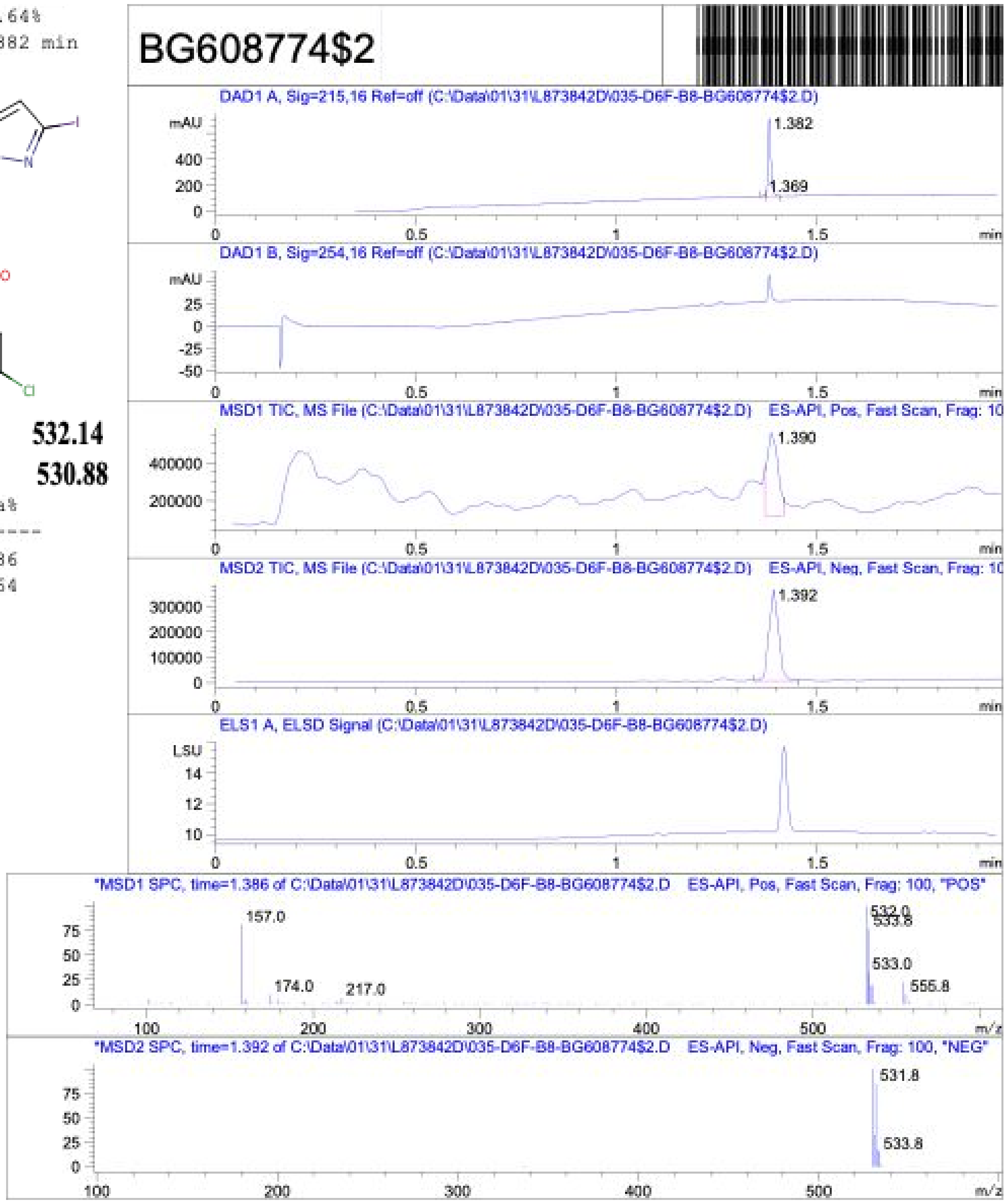

RT 1.390

RT 1.392

Inj.Date 1/31/2025 C

C:\Users\Public\Documents\ChemStation\1\Data\01_31\L873842D\SUPOR_30.M

Z9464814823

MaxPeak: 100.00%
Ret_Time: 1.239 min

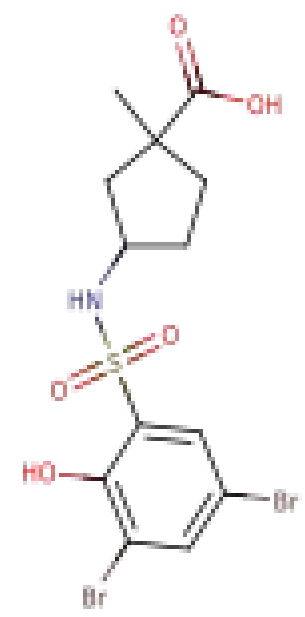

Mol Wt 457.14

Exact Mass 456.91

#	Time	Area%
1	1.239	100.00

BG608780$1

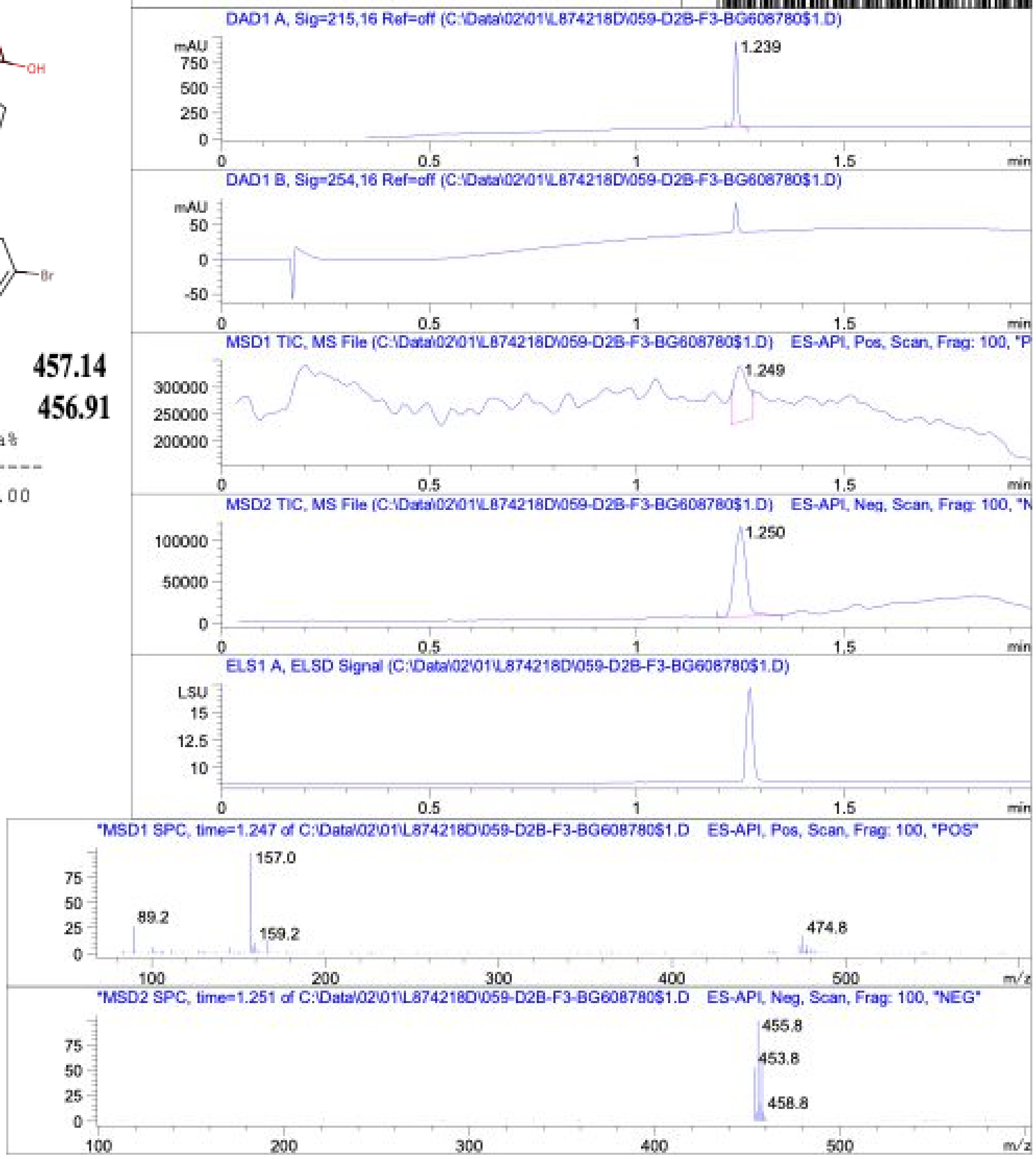

RT 1.249

RT 1.250

Inj.Date 2/1/2025 C -9-

C:\Users\Public\Documents\ChemStation\1\Data\01_31\L874218D\SUPOR_96.M

Z9464814843

MaxPeak: 100.00%
Ret_Time: 1.345 min

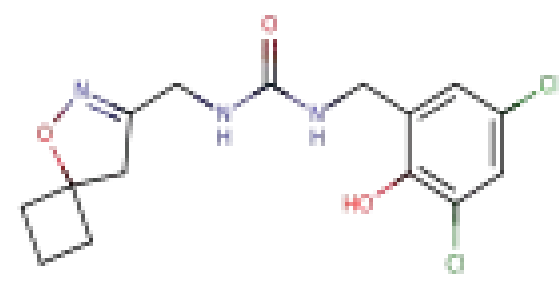

Mol Wt 358.22

Exact Mass 357.08

#	Time	Area%
1	1.345	100.00

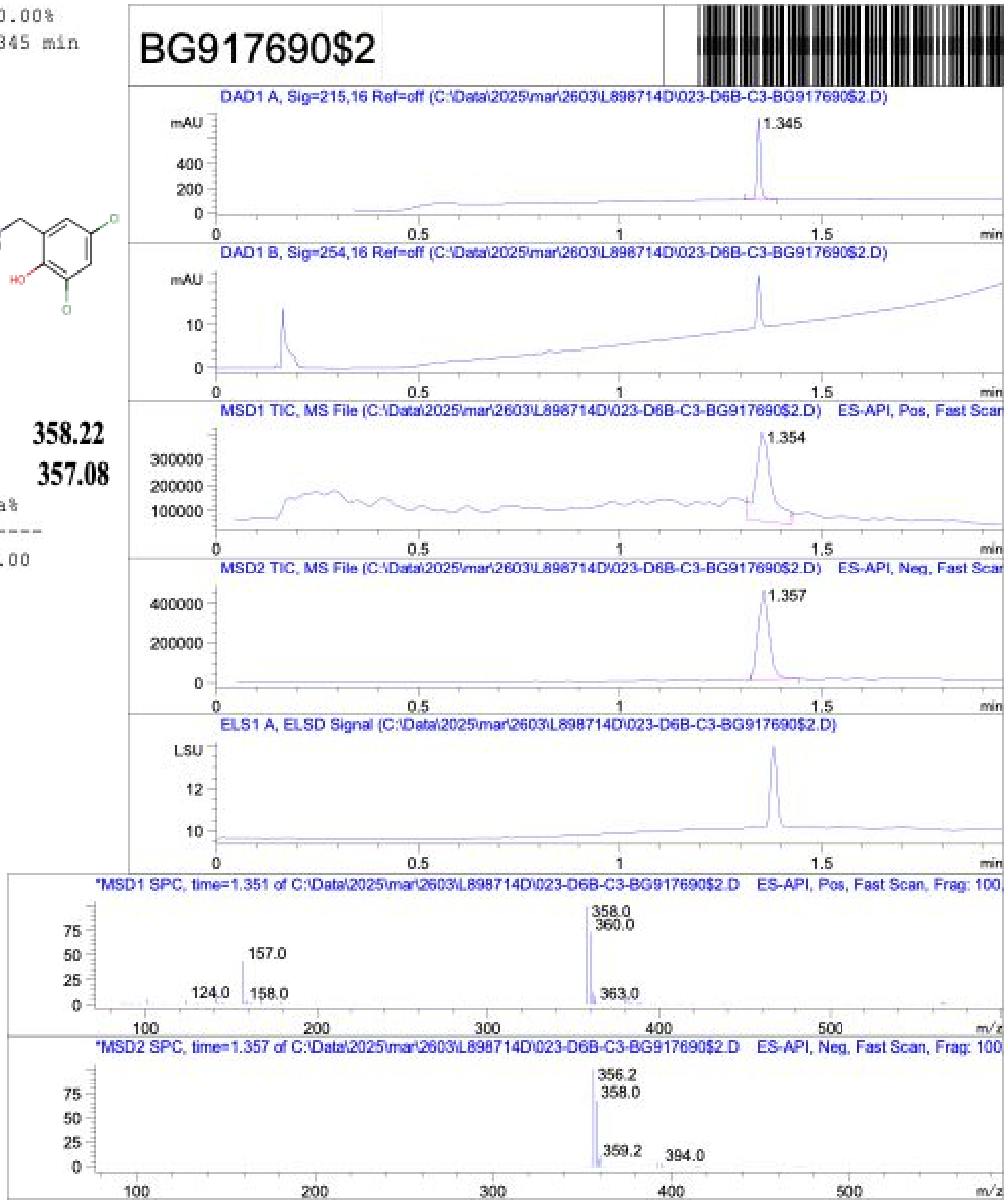

RT 1.354

RT 1.357

Inj.Date 3/25/2025 NEW -33-

C:\Users\Public\Documents\ChemStation\1\Data\03_25\L898714D\SUPOR_30.M

Z9544921678

MaxPeak: 100.00%
Ret_Time: 1.115 min

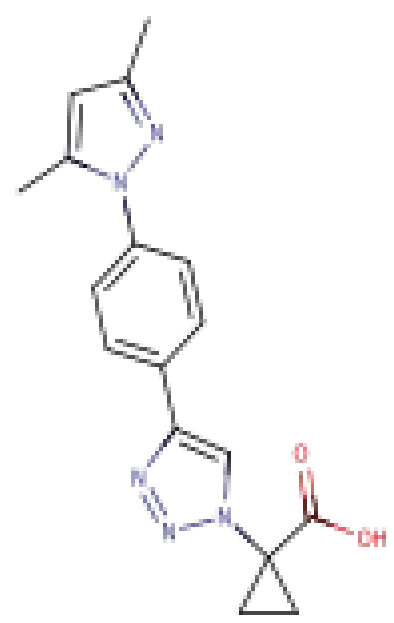

Mol Wt 323.35
Exact Mass 323.15

#	Time	Area%
1	1.115	100.00

BG917691$2

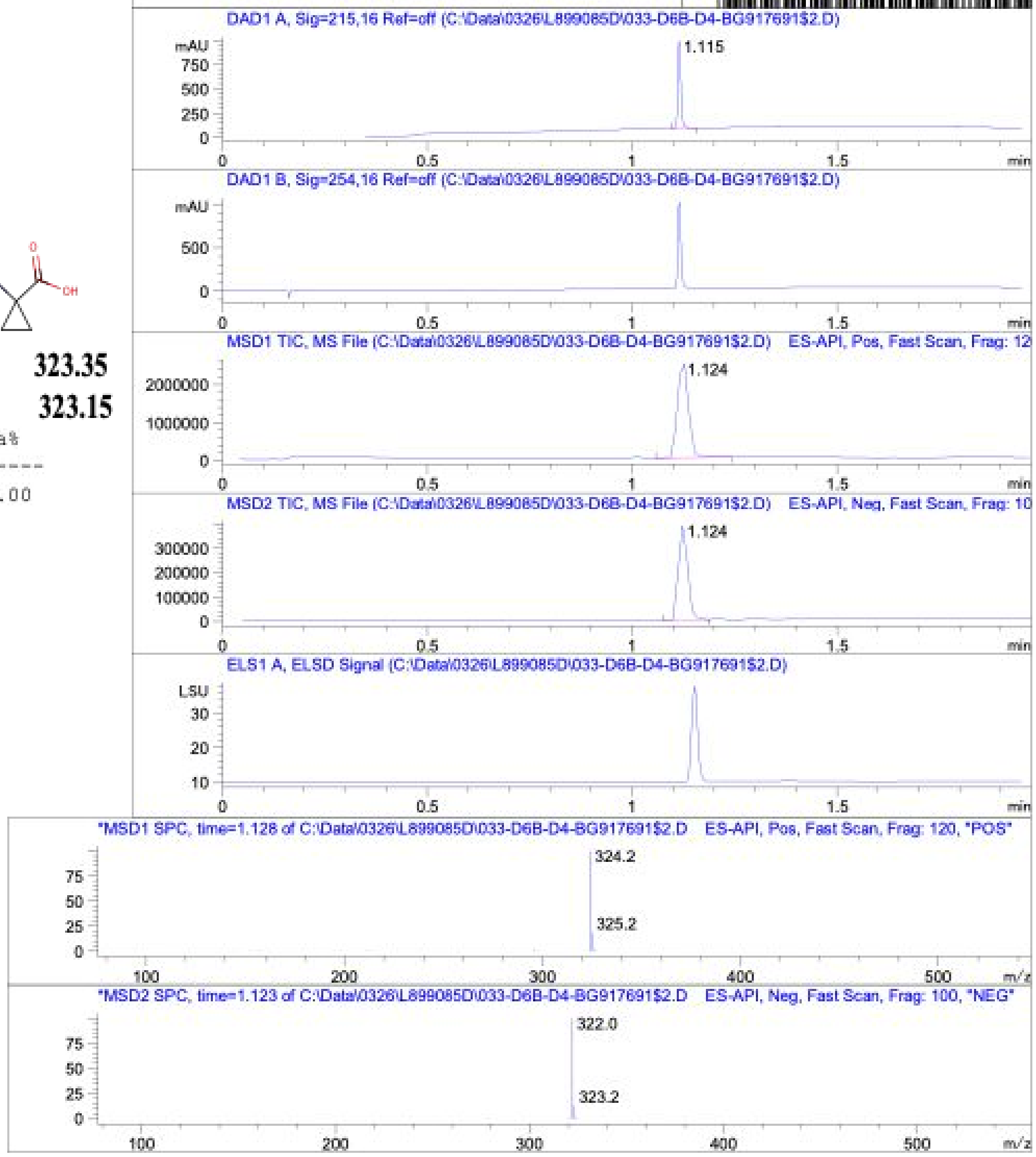

RT 1.124

RT 1.124

Inj.Date 3/25/2025 NEW -1-
C:\Users\Public\Documents\ChemStation\1\Data\03_25\L899085D\SUPOR_30.M

Z9544921685

MaxPeak: 87.84%
Ret_Time: 1.279 min

BG917692$11

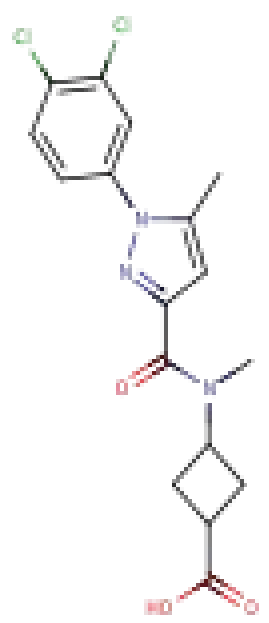

Mol Wt 382.24

Exact Mass 381.08

#	Time	Area%
1	1.252	12.16
2	1.279	87.84

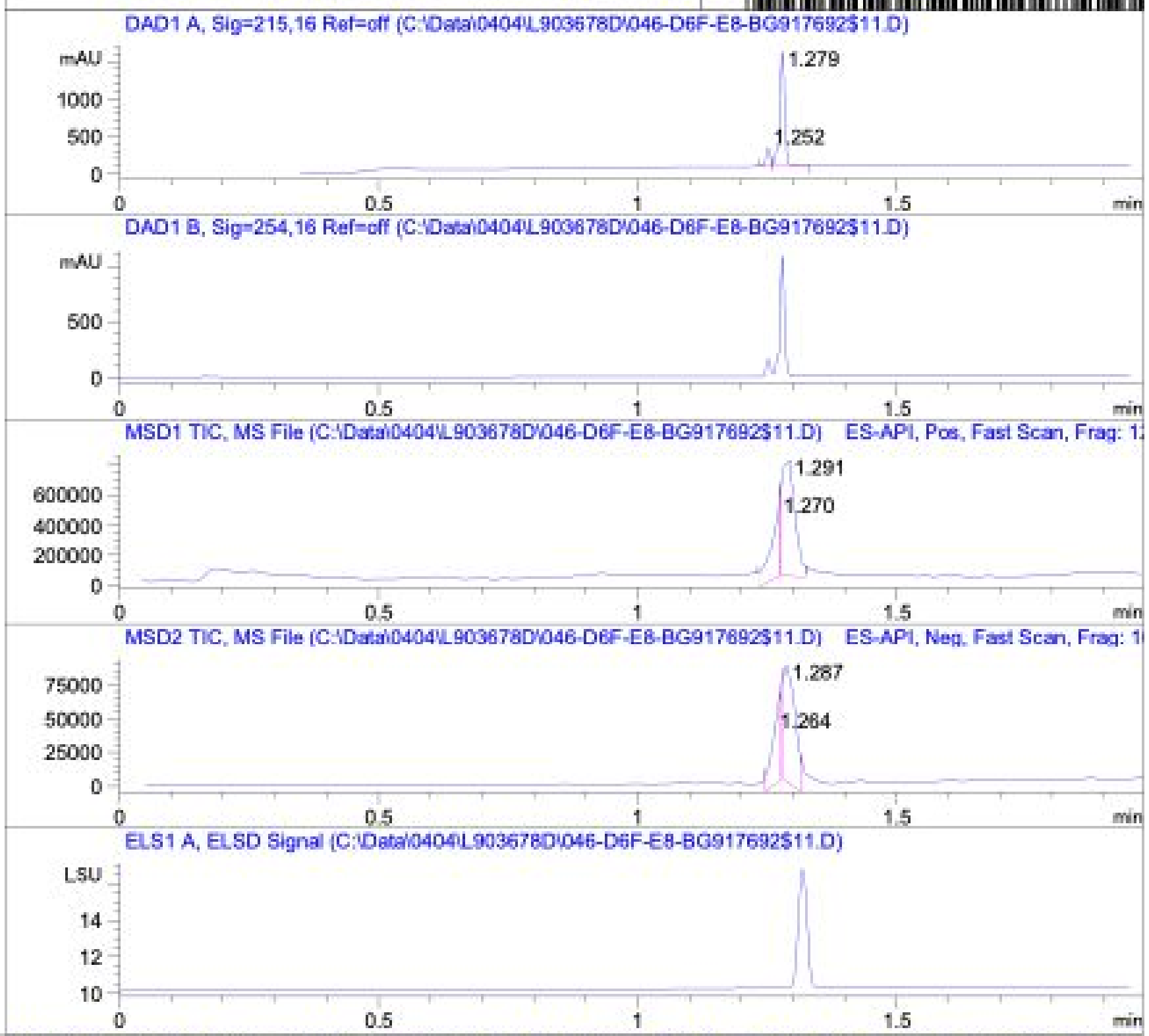

RT 1.270

RT 1.291

RT 1.264

RT 1.287

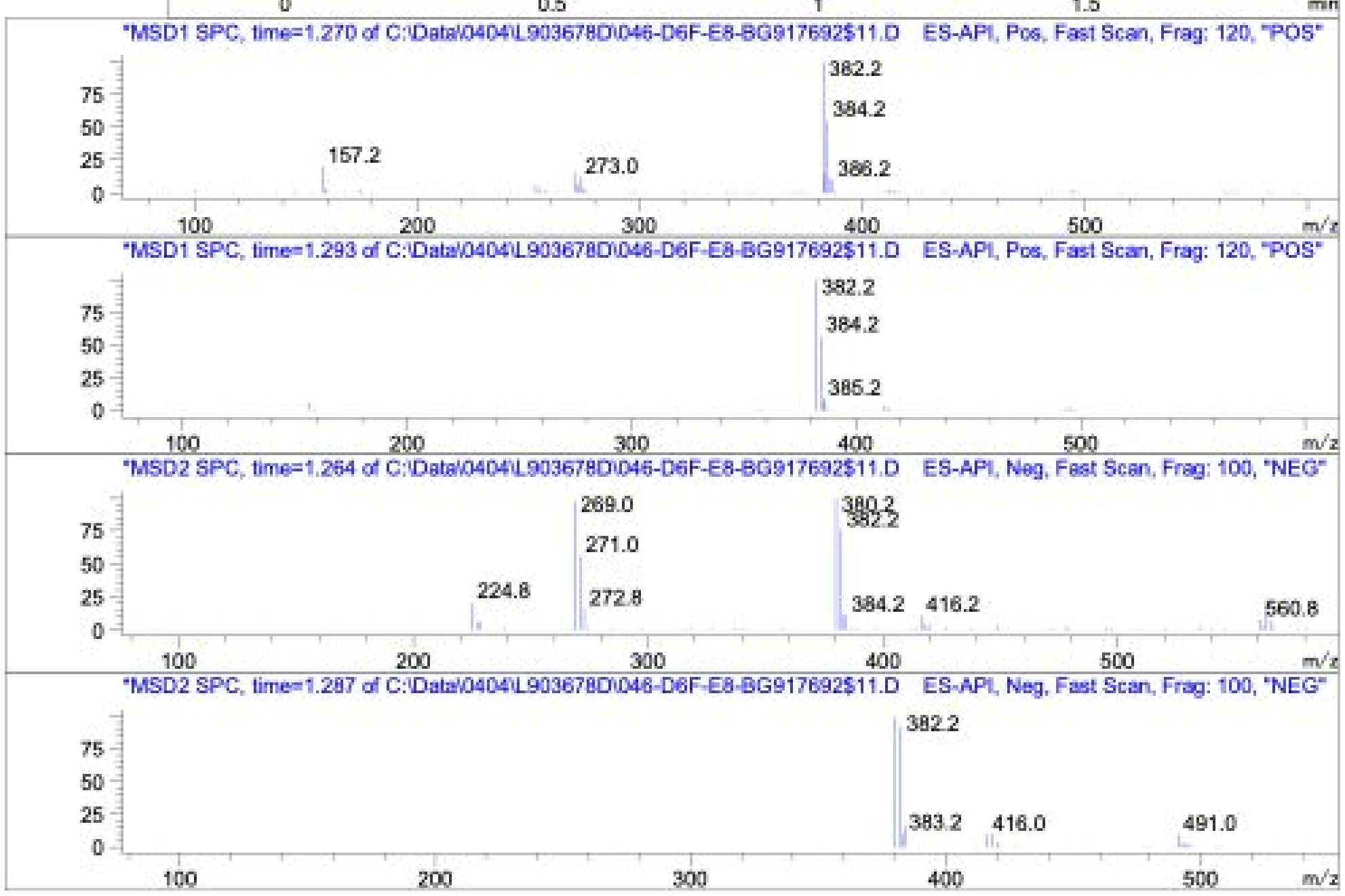

Inj.Date 4/4/2025 KCH -39-
C:\Users\Public\Documents\ChemStation\1\Data\04_03\L903678D\SUPOR_30.M

Z9544921693

MaxPeak: 100.00%
Ret_Time: 1.398 min

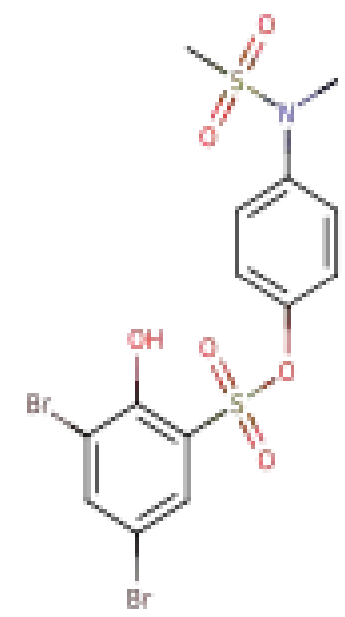

Mol Wt 515.2

Exact Mass 514.85

#	Time	Area%
1	1.398	100.00

BG917697$22

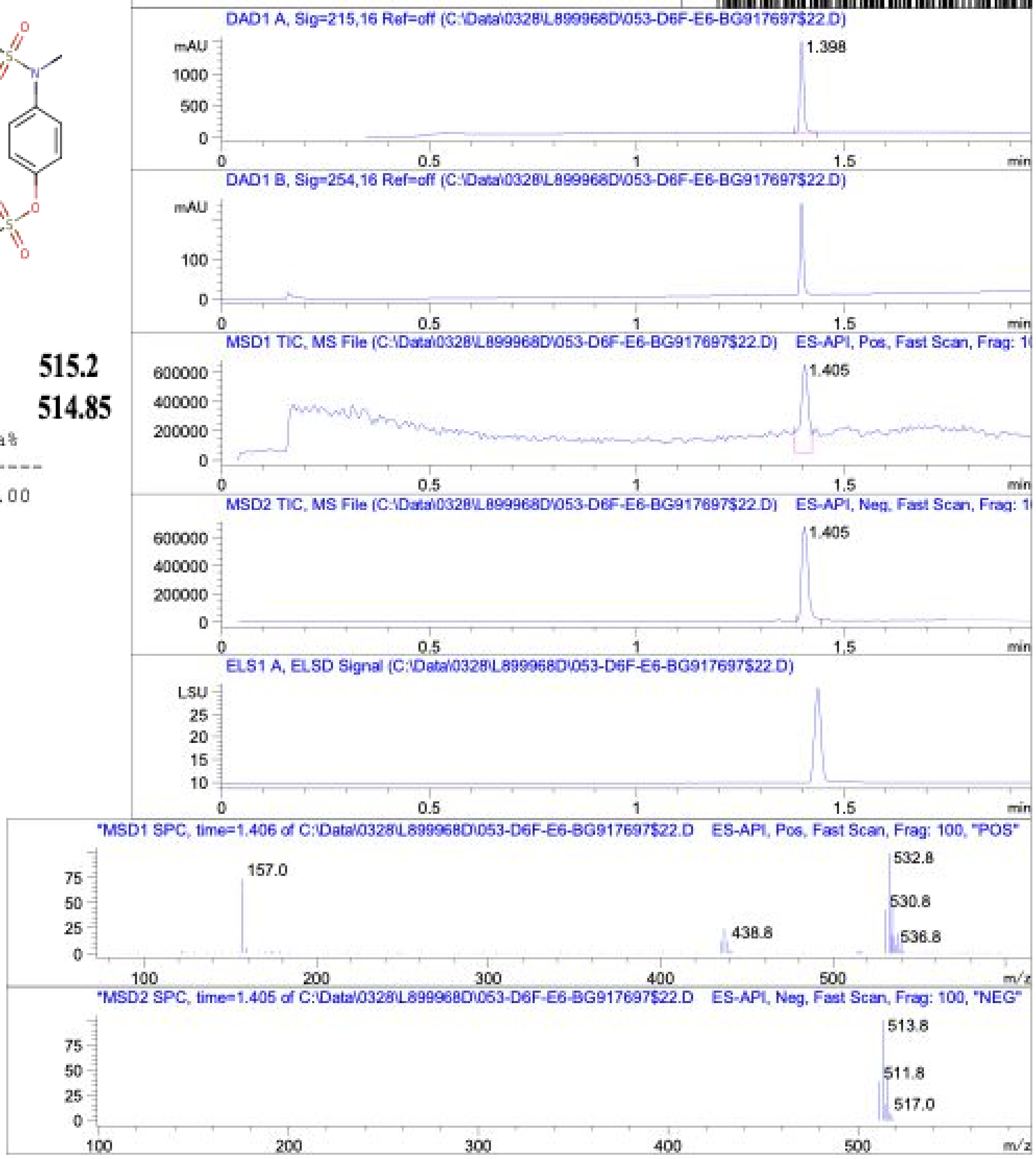

RT 1.405

RT 1.405

Inj.Date 3/27/2025 NEW

C:\Users\Public\Documents\ChemStation\1\Data\03_27\L899968D\SUPOR_30.M

Z9544921726

MaxPeak: 100.00%
Ret_Time: 1.466 min

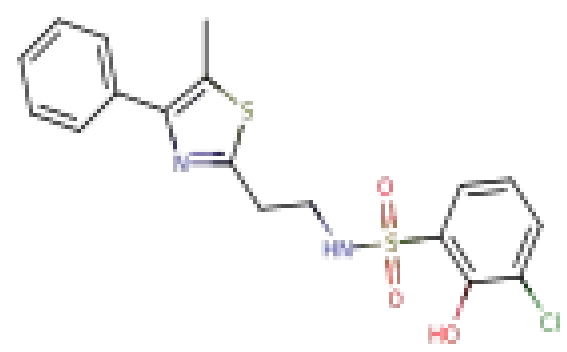

Mol Wt 408.92
Exact Mass 408.05

#	Time	Area%
1	1.466	100.00

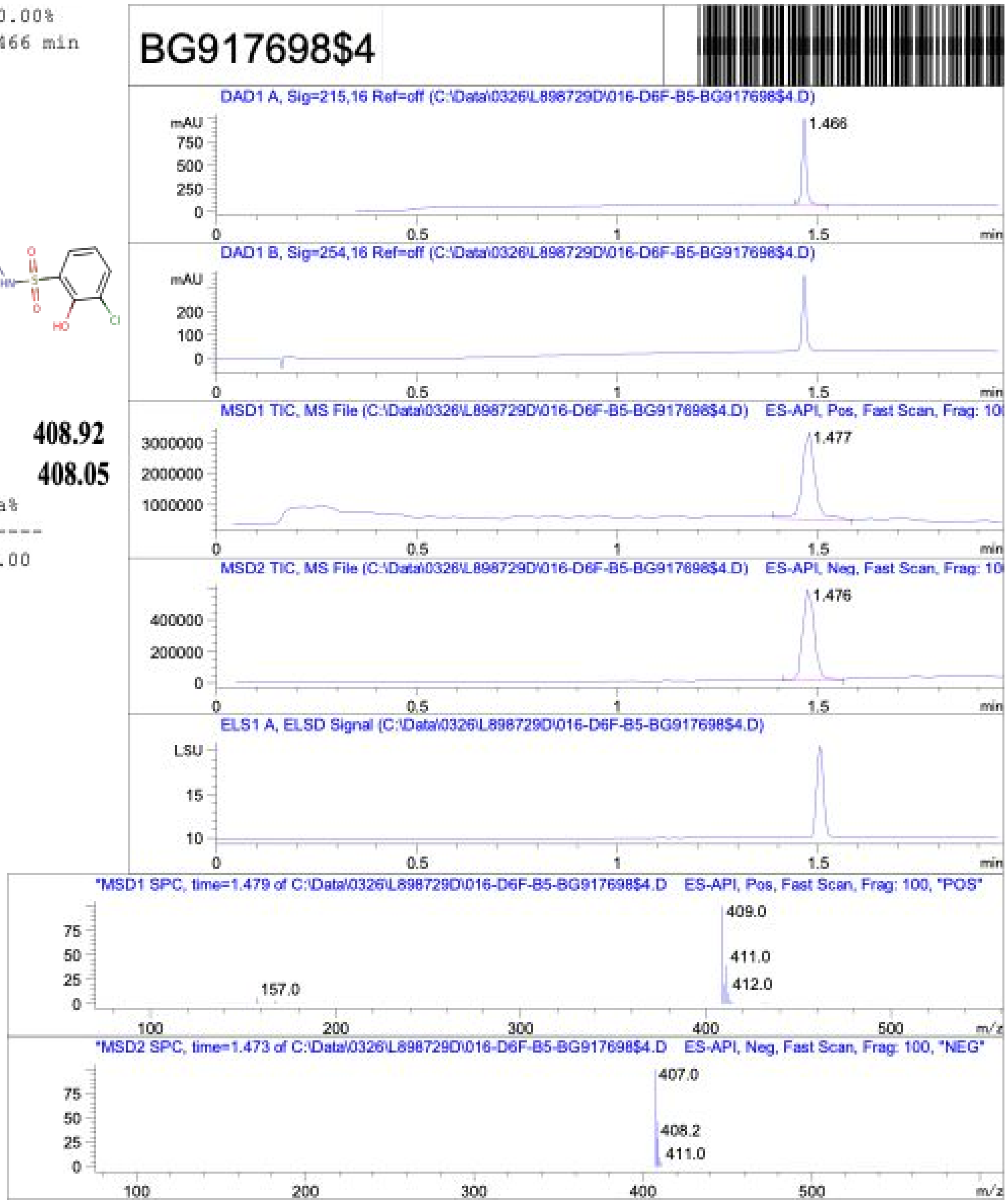

RT 1.477

RT 1.476

Inj.Date 3/25/2025 NEW
C:\Users\Public\Documents\ChemStation\1\Data\03_25\L898729D\SUPOR_30.M

Z9544921729

MaxPeak: 92.43%
Ret_Time: 1.481 min

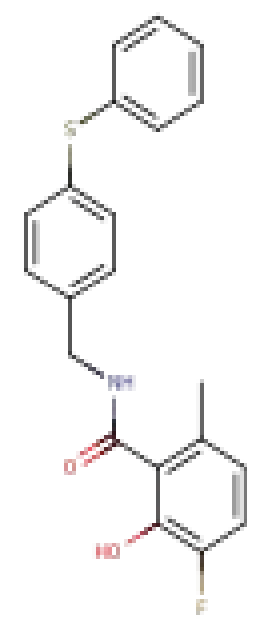

Mol Wt 367.44

Exact Mass 367.13

#	Time	Area%
1	1.018	1.44
2	1.035	1.33
3	1.434	1.61
4	1.447	3.19
5	1.481	92.43

BG917700$3

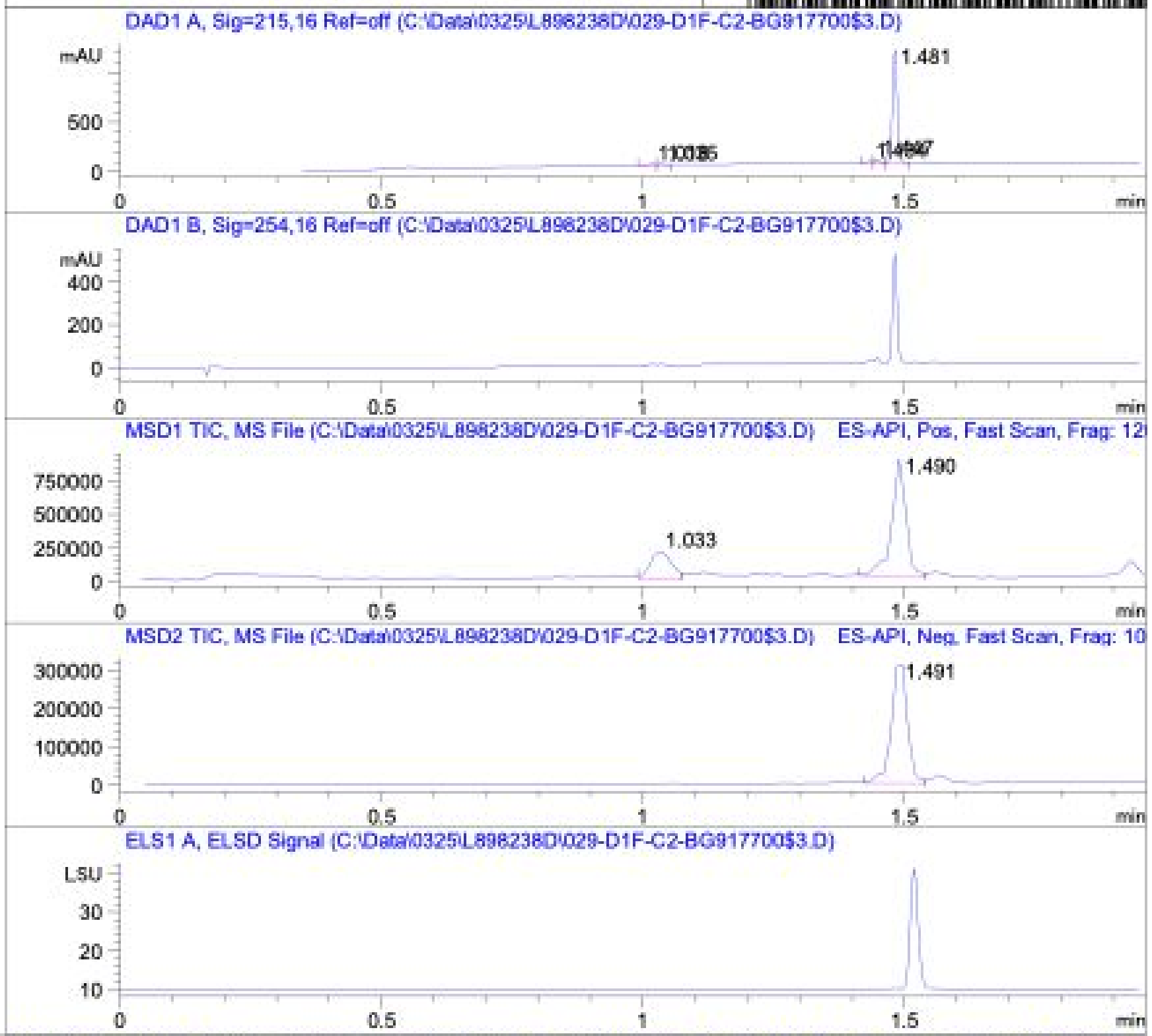

RT 1.033

RT 1.490

RT 1.491

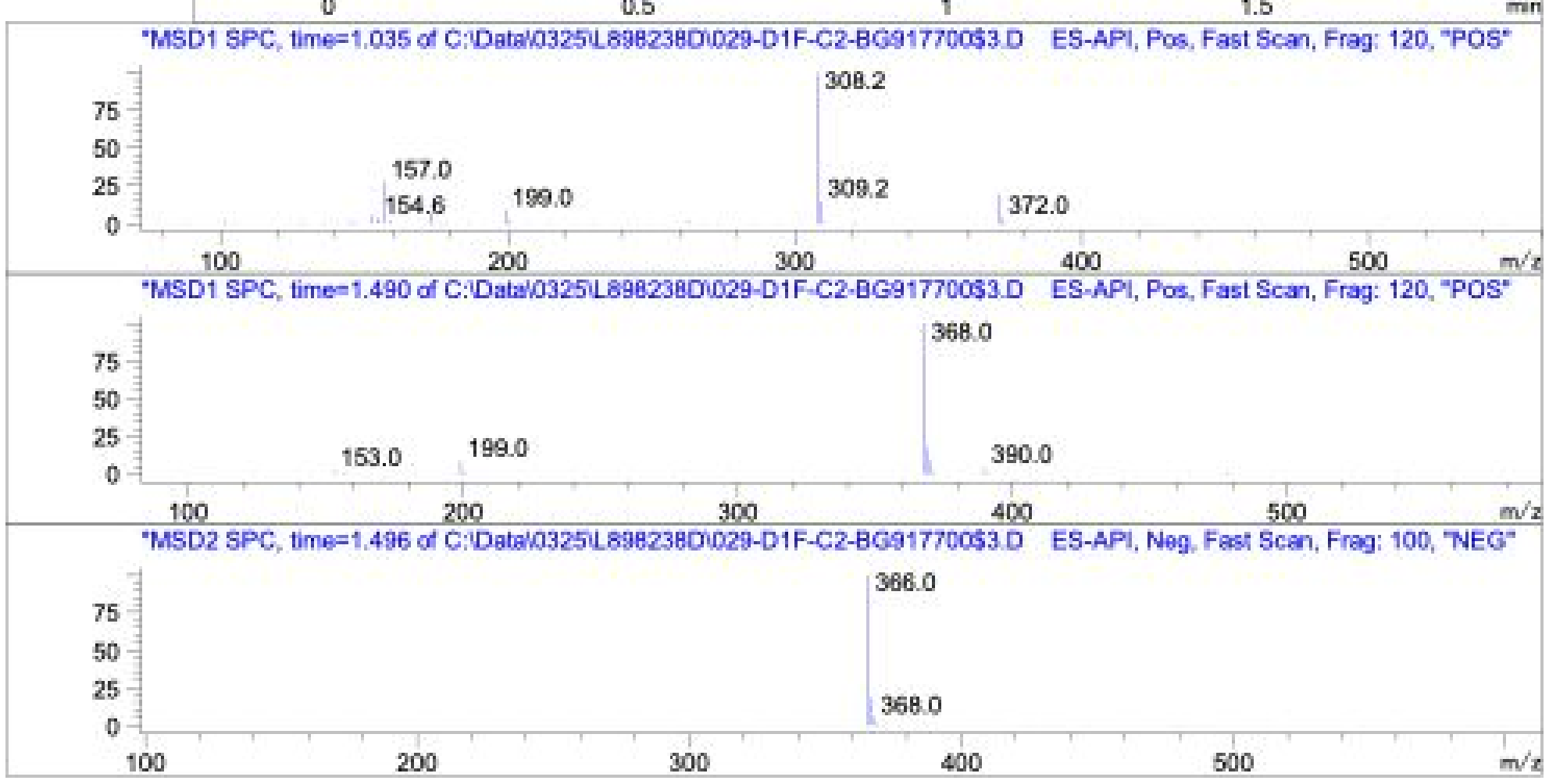

Inj.Date 3/24/2025 NEW -21-
C:\Users\Public\Documents\ChemStation\1\Data\03_24\L898238D\SUPOR_30.M

Z9544921741

MaxPeak: 97.59%
Ret_Time: 1.152 min

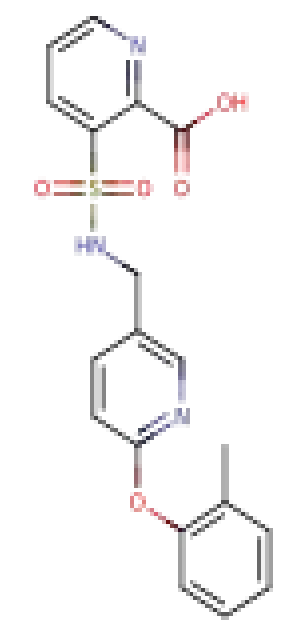

Mol Wt 399.42

Exact Mass 399.09

#	Time	Area%
1	1.152	97.59
2	1.234	2.41

BG917704$41

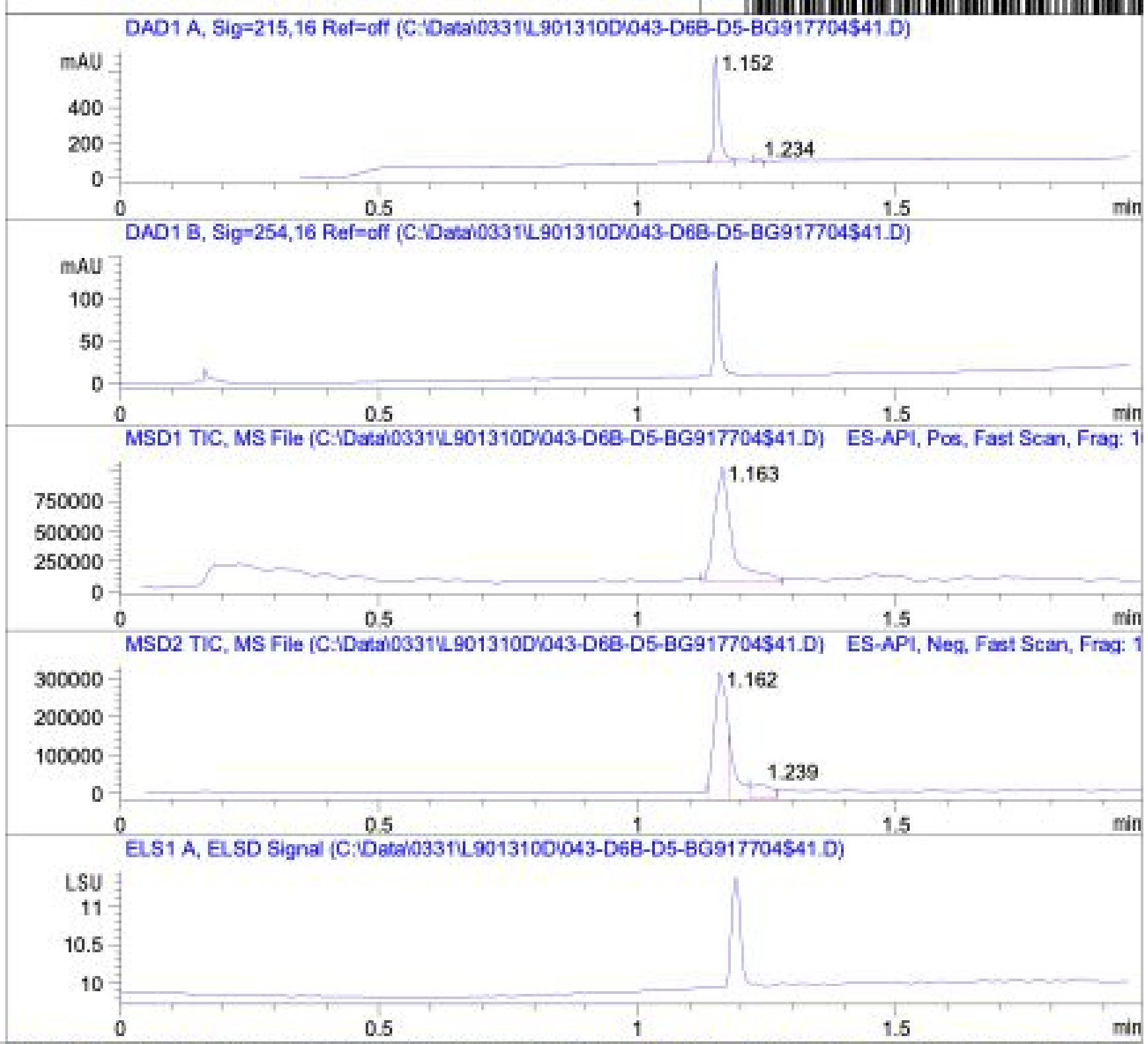

RT 1.163

RT 1.162

RT 1.239

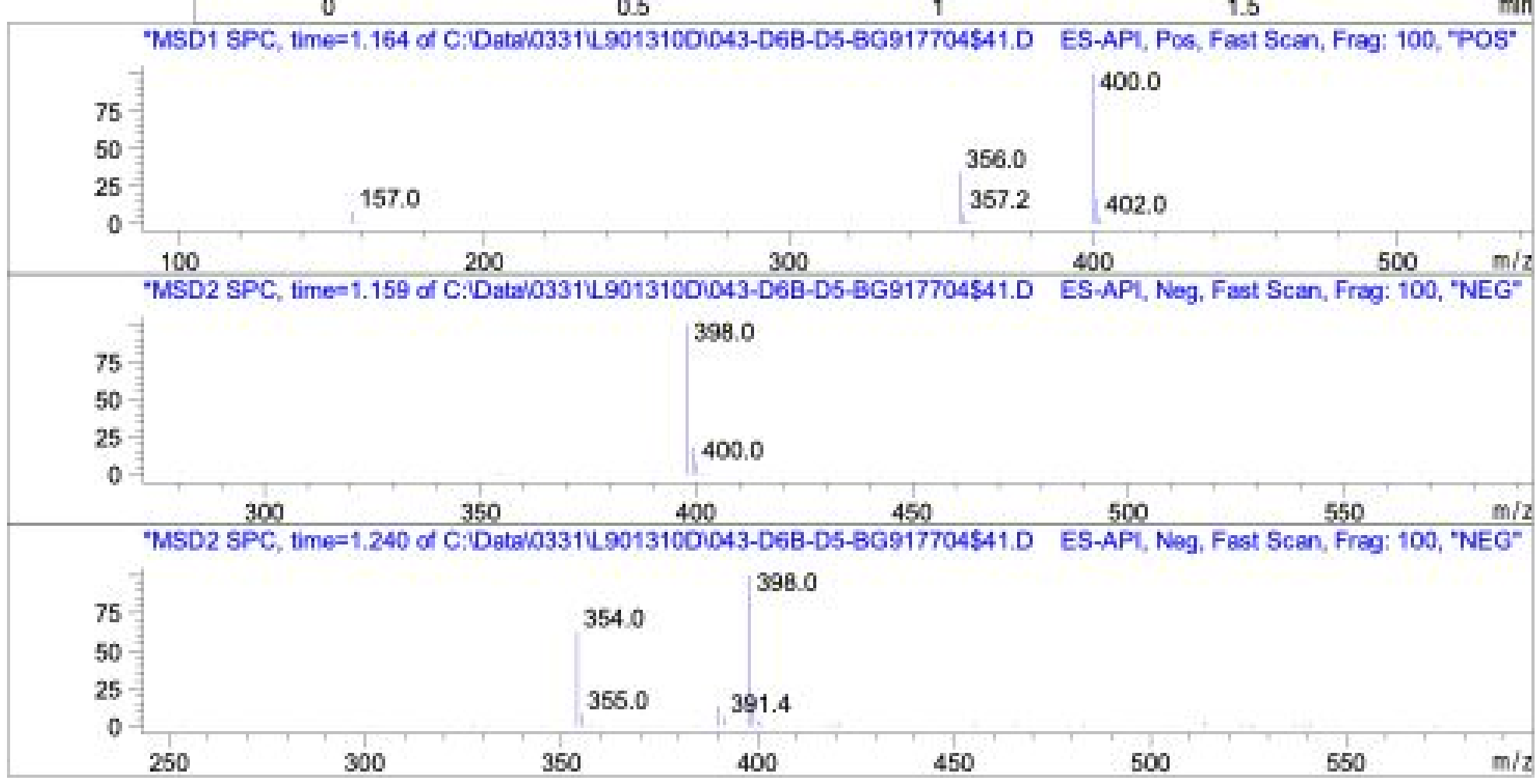

Inj.Date 3/31/2025 NEW -34-
C:\Users\Public\Documents\ChemStation\1\Data\03_31\L901310D\SUPOR_30.M

Z9544921759

MaxPeak: 100.00%
Ret_Time: 0.882 min

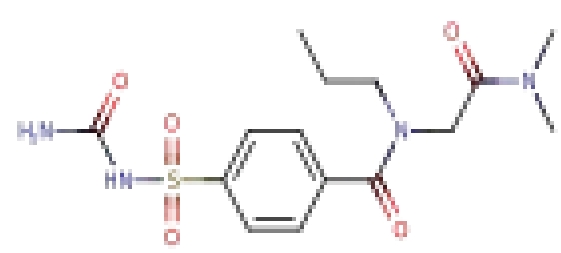

Mol Wt 370.43

Exact Mass 370.14

#	Time	Area%
1	0.882	100.00

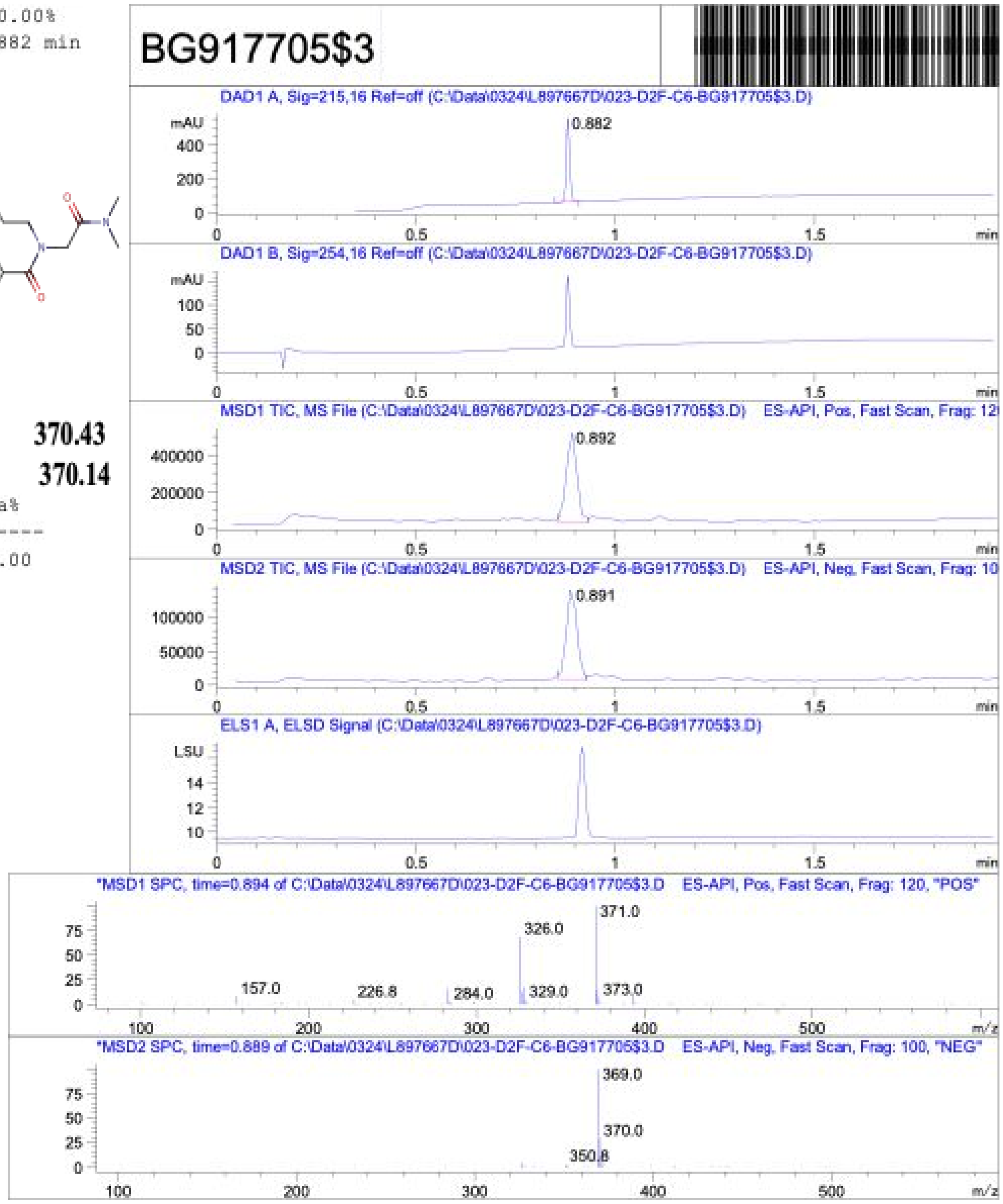

Inj.Date 3/22/2025 NEW -21-
C:\Users\Public\Documents\ChemStation\1\Data\03_21\L897667D\SUPOR_30.M

Z9544921762

MaxPeak: 91.51%
Ret_Time: 1.216 min

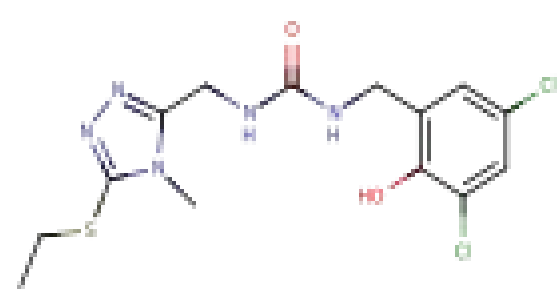

Mol Wt 390.29

Exact Mass 389.06

#	Time	Area%
1	0.581	2.83
2	1.216	91.51
3	1.265	3.77
4	1.616	1.89

BG917709$2

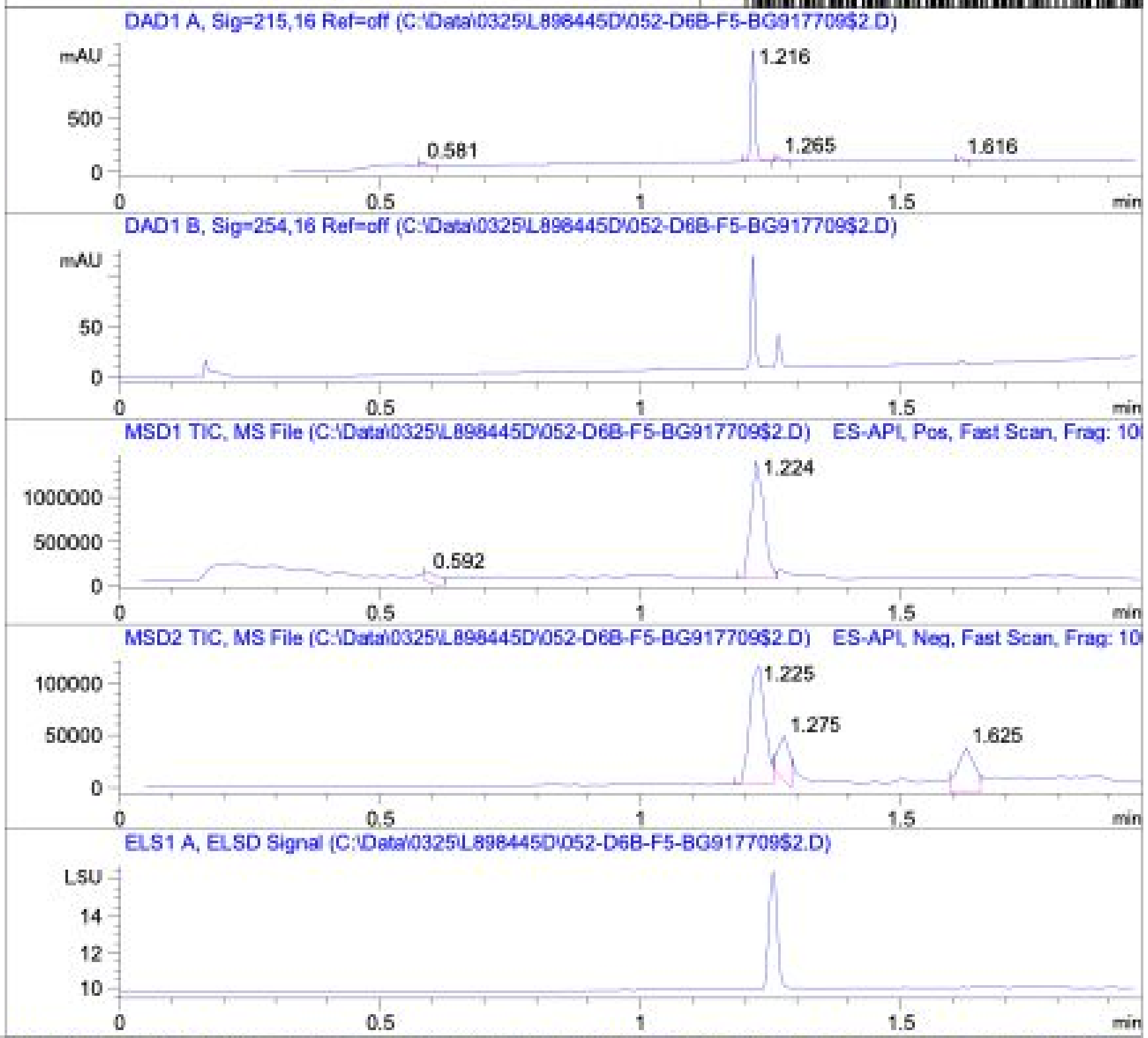

RT 0.592

RT 1.224

RT 1.225

RT 1.275

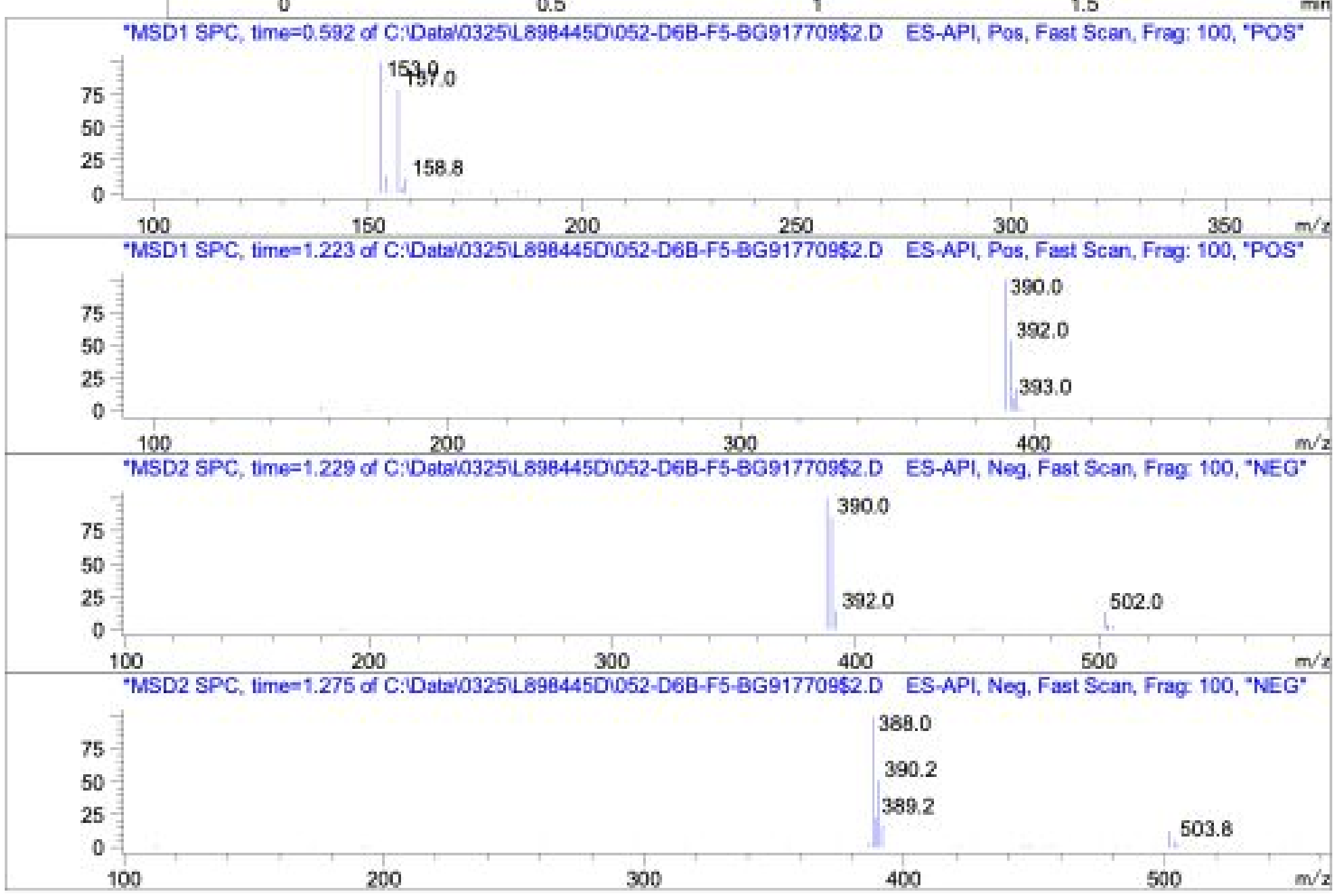

Inj.Date 3/24/2025 NEW -34-
C:\Users\Public\Documents\ChemStation\1\Data\03_24\L898445D\SUPOR_30.M

Z9544921788

MaxPeak: 96.80%
Ret_Time: 1.502 min

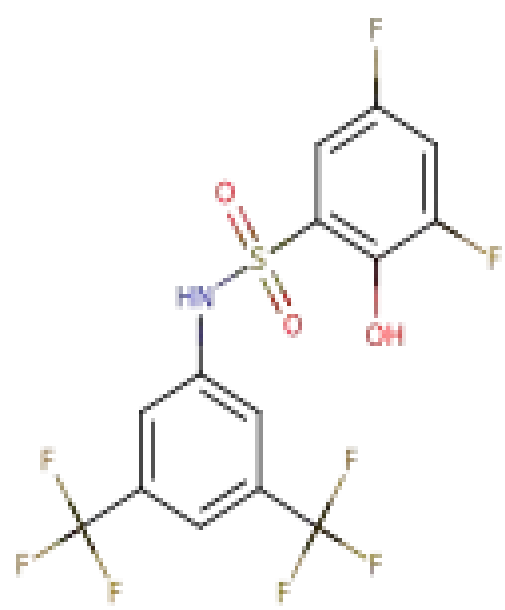

Mol Wt 421.26
Exact Mass 421.01

#	Time	Area%
1	1.051	3.20
2	1.502	96.80

BG917711$1

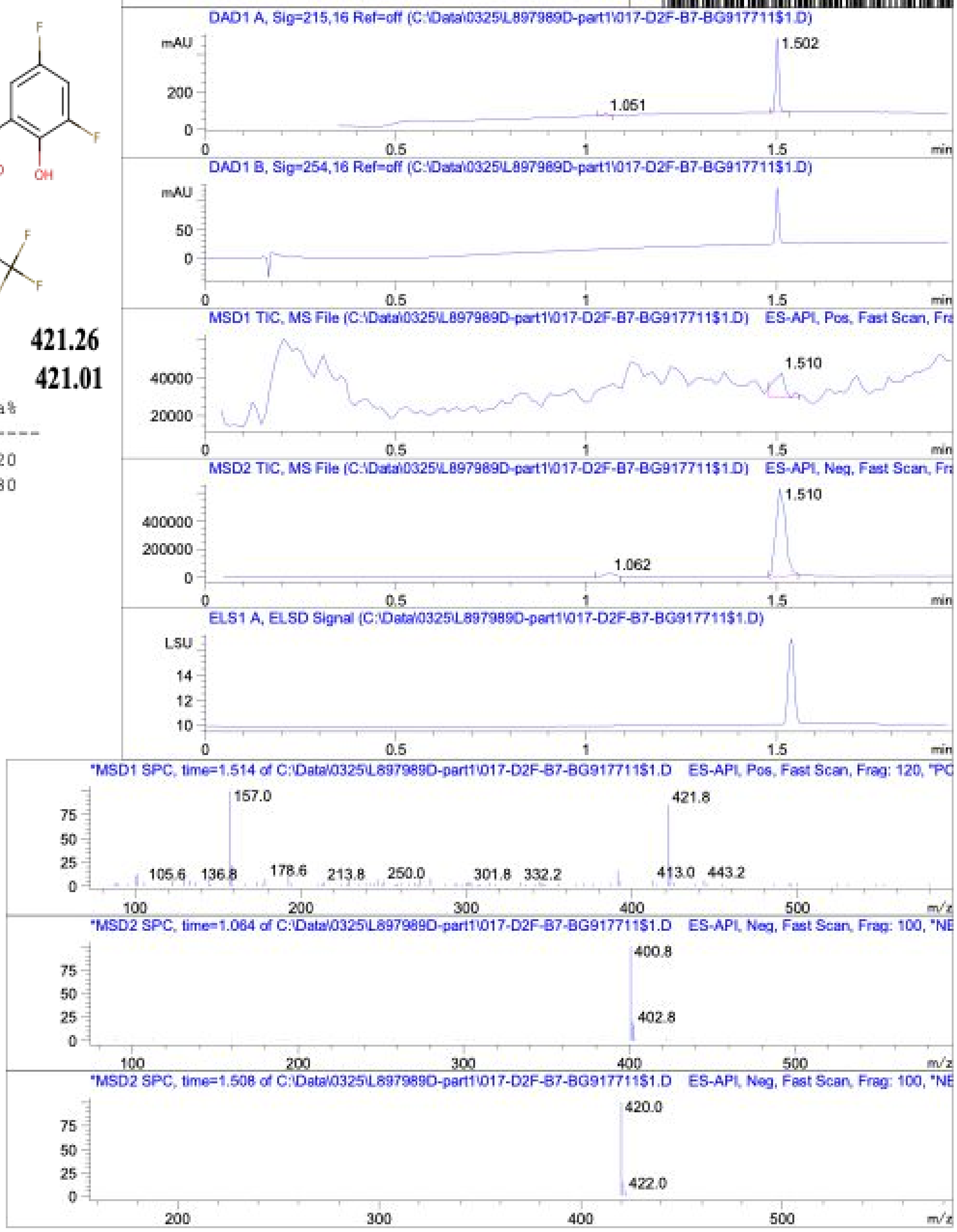

Inj.Date 3/24/2025 NEW -21-
C:\Users\Public\Documents\ChemStation\1\Data\03_24\1897989D\SUPOR_30.M

Z9544921801

MaxPeak: 100.00%
Ret_Time: 1.052 min

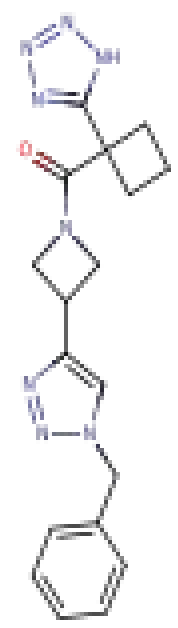

Mol Wt 364.4

Exact Mass 364.19

#	Time	Area%
1	1.052	100.00

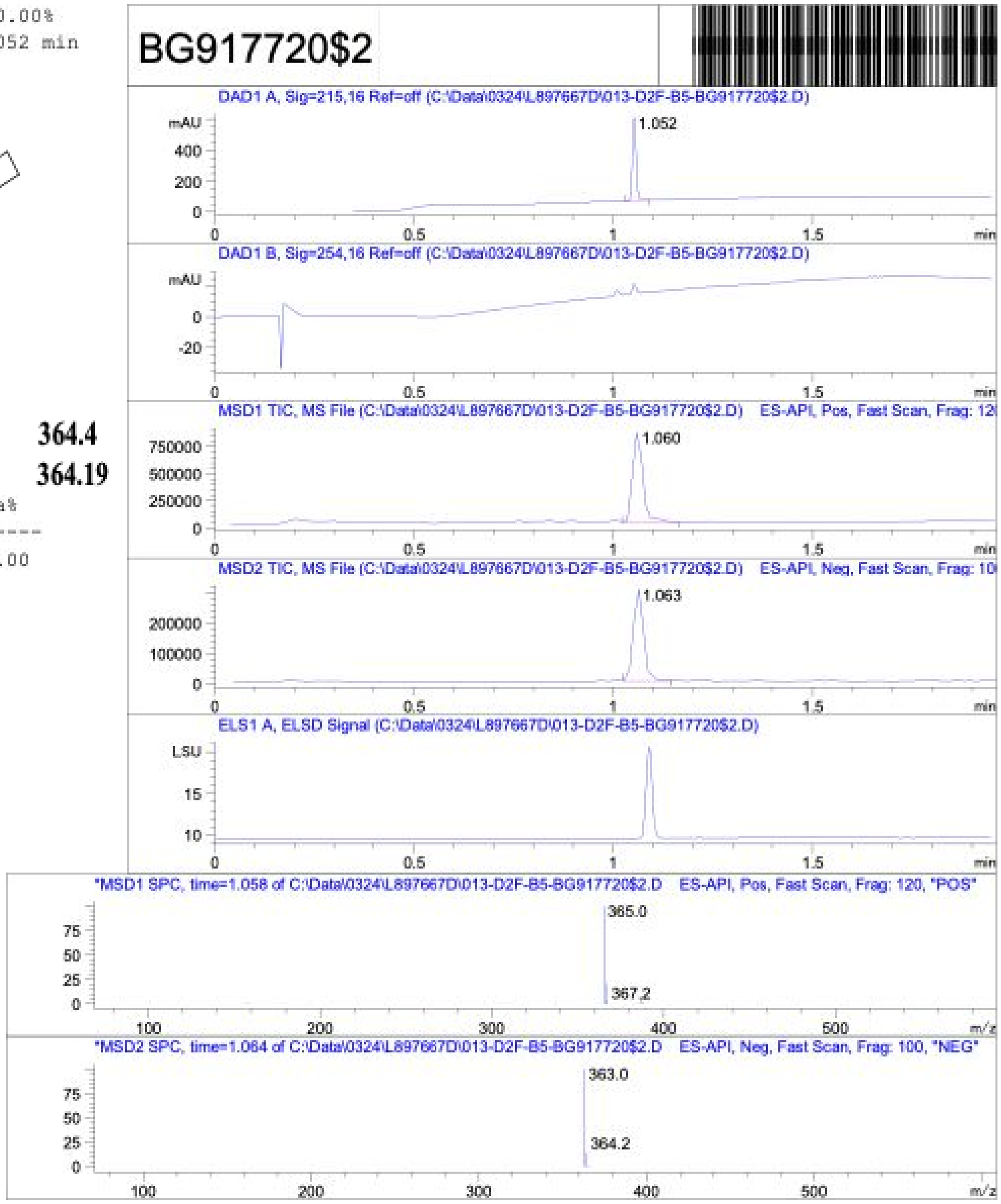

Inj.Date 3/22/2025 NEW -21-
C:\Users\Public\Documents\ChemStation\1\Data\03_21\L897667D\SUPOR_30.M

Z9544921851

MaxPeak: 100.00%
Ret_Time: 1.079 min

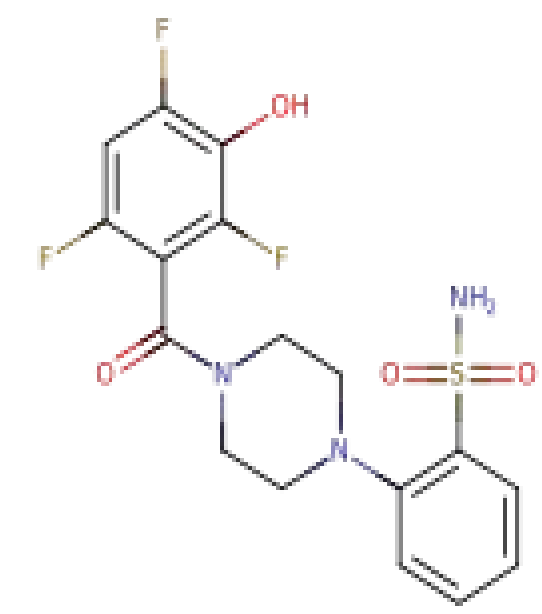

Mol Wt 415.39

Exact Mass 415.09

#	Time	Area%
1	1.079	100.00

BG917721$11

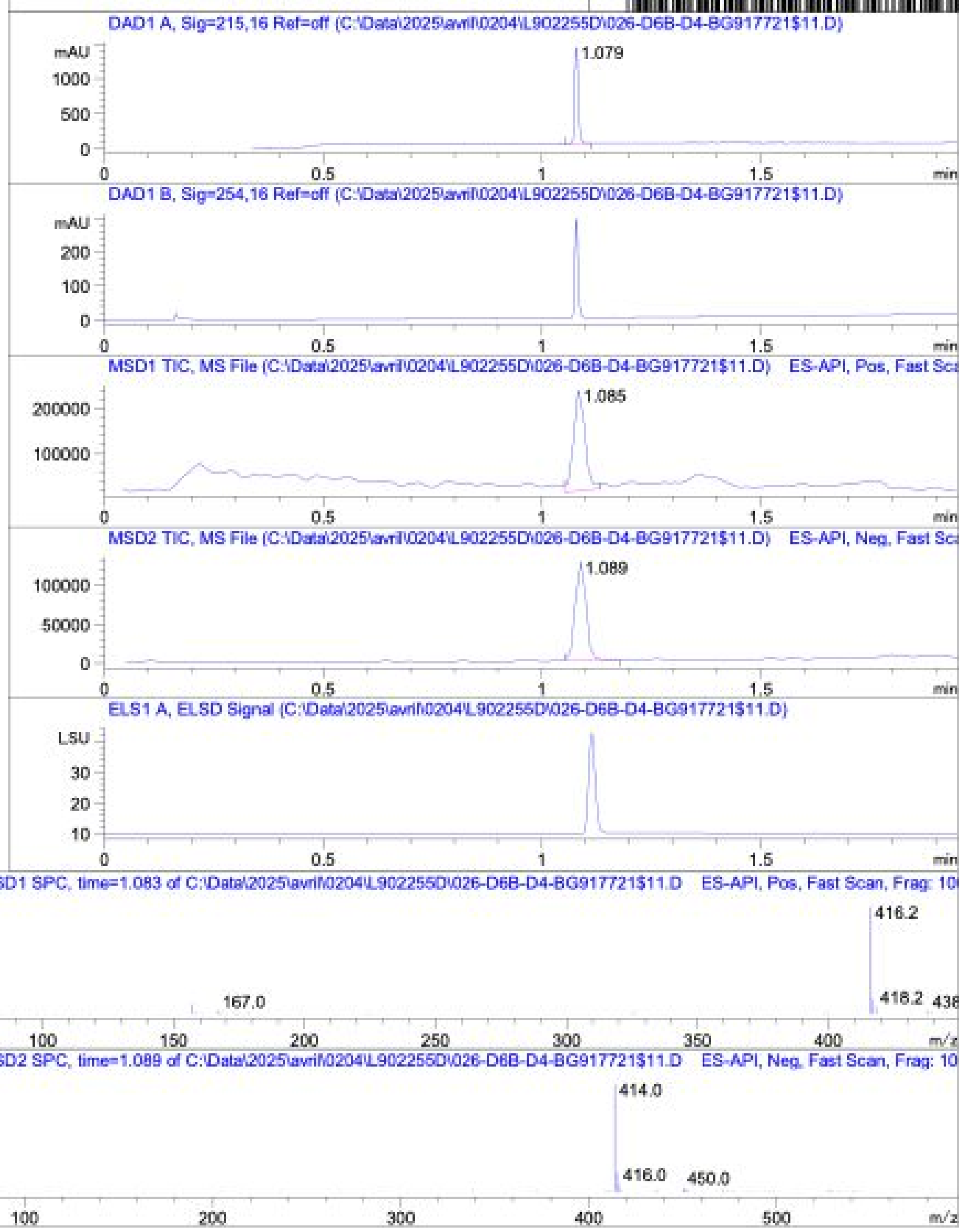

RT 1.085

RT 1.089

Inj.Date 4/2/2025 NEW
C:\Users\Public\Documents\ChemStation\1\Data\04_01\L902255D\SUPOR_30.M

Z9544921860

MaxPeak: 100.00%
Ret_Time: 1.329 min

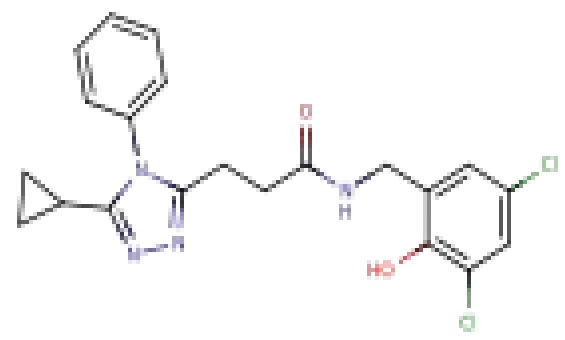

Mol Wt 431.32
Exact Mass 430.12

#	Time	Area%
1	1.329	100.00

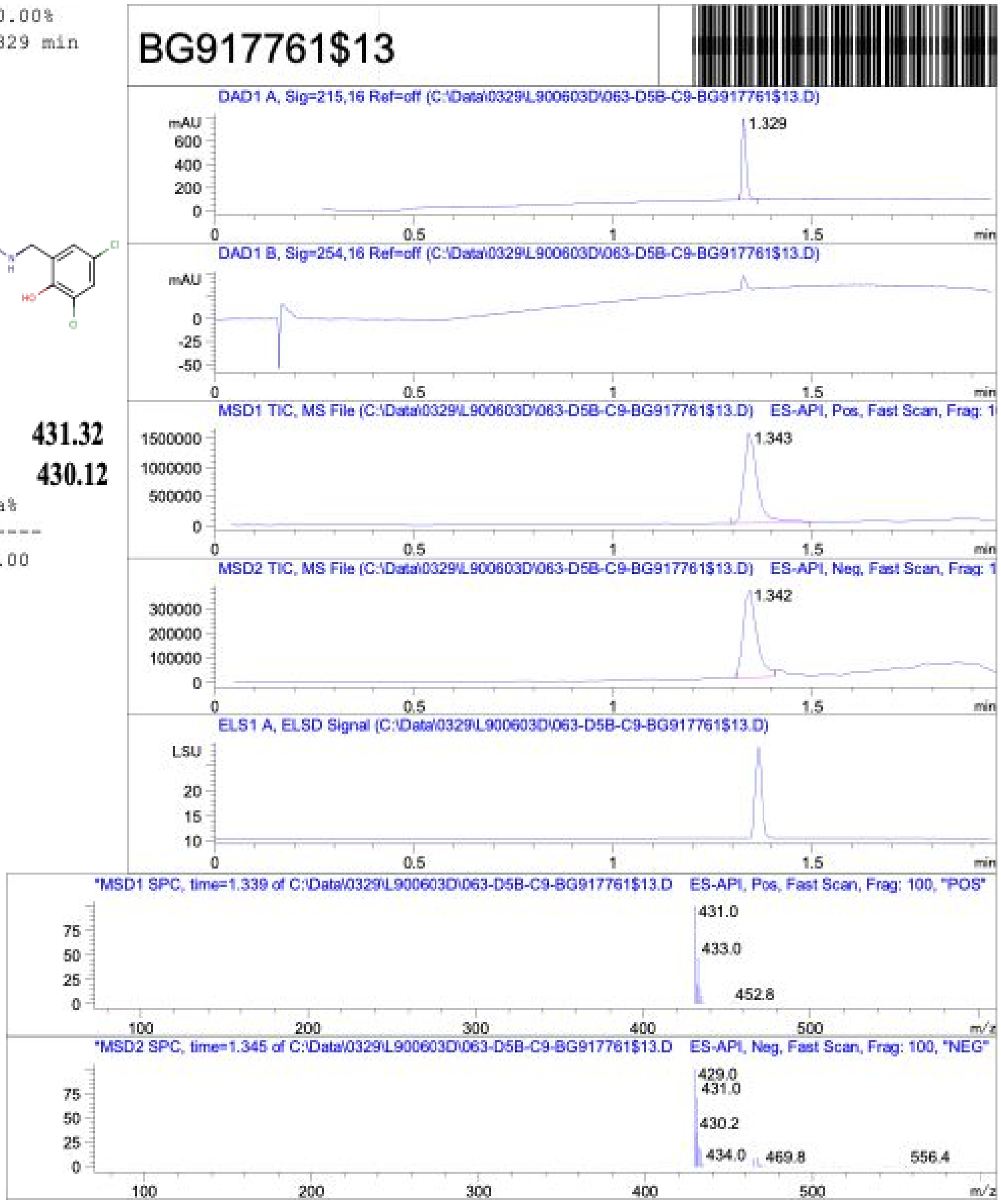

RT 1.343

RT 1.342

Inj.Date 3/28/2025 NEW - 23 -
C:\Users\Public\Documents\ChemStation\1\Data\03_28\L900603D\SUPOR_30.M

Z9544921884

MaxPeak: 100.00%
Ret_Time: 1.211 min

BG917768$1

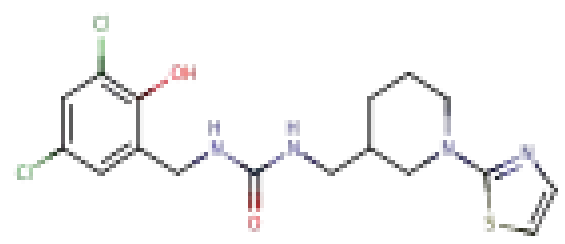

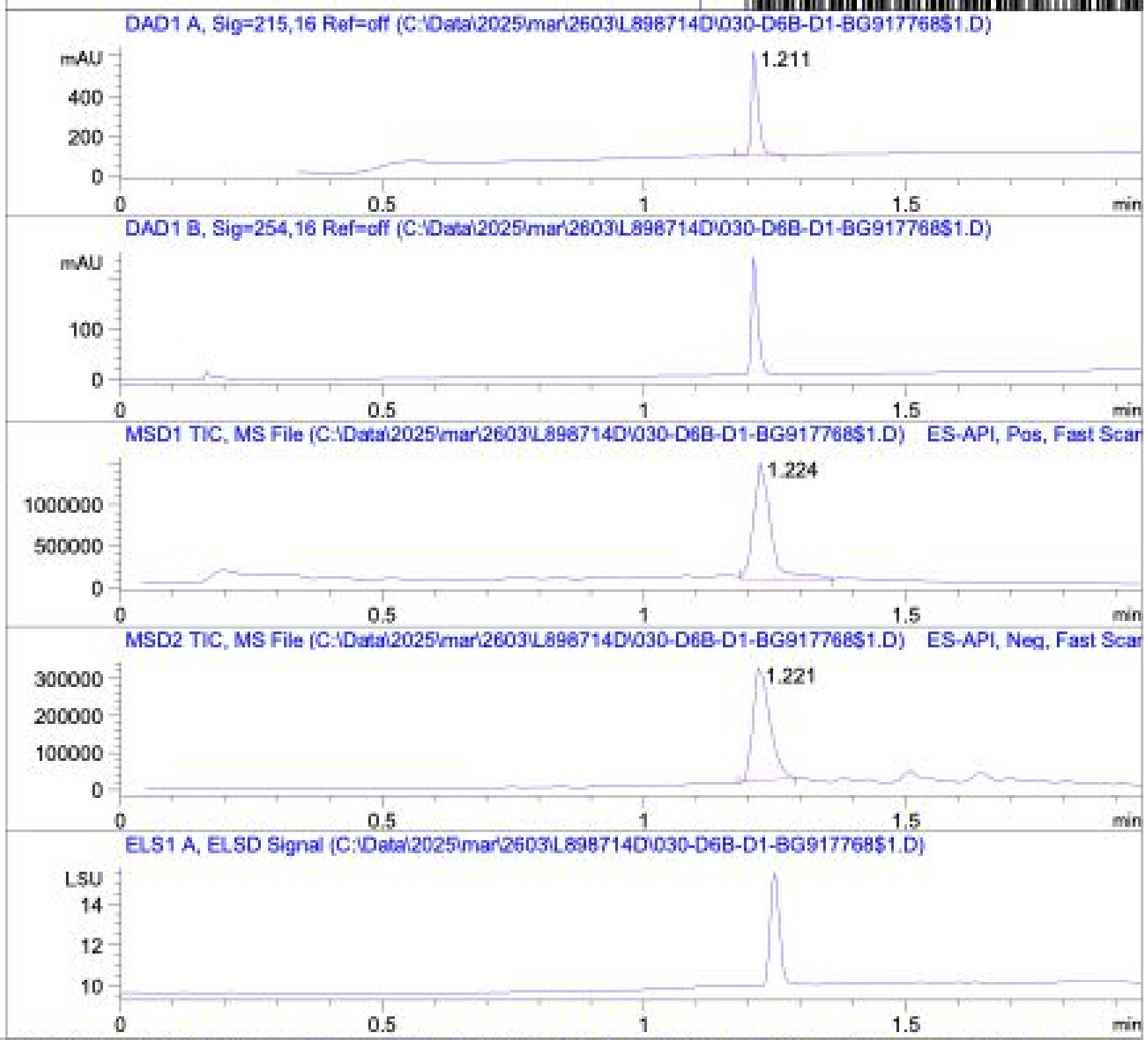

Mol Wt 415.34
Exact Mass 414.09

#	Time	Area%
1	1.211	100.00

RT 1.224

RT 1.221

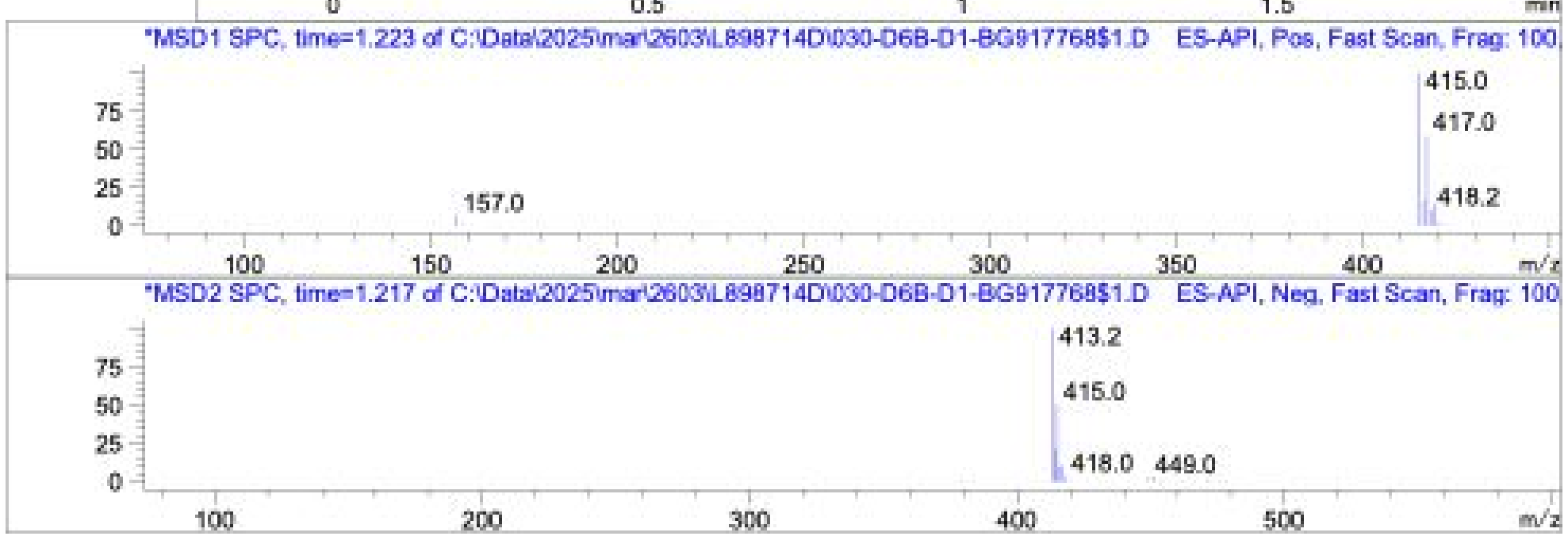

Inj.Date 3/26/2025 NEW -33-
C:\Users\Public\Documents\ChemStation\1\Data\03_25\L898714D\SUPOR_30.M

Z9544921929

MaxPeak: 98.18%
Ret_Time: 1.014 min

BG917770$2

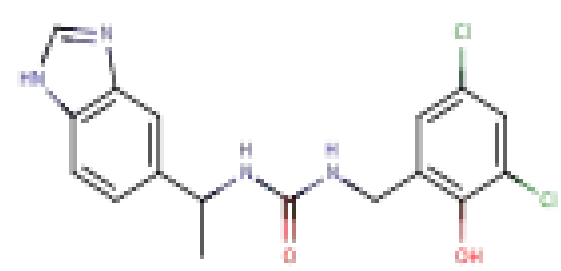

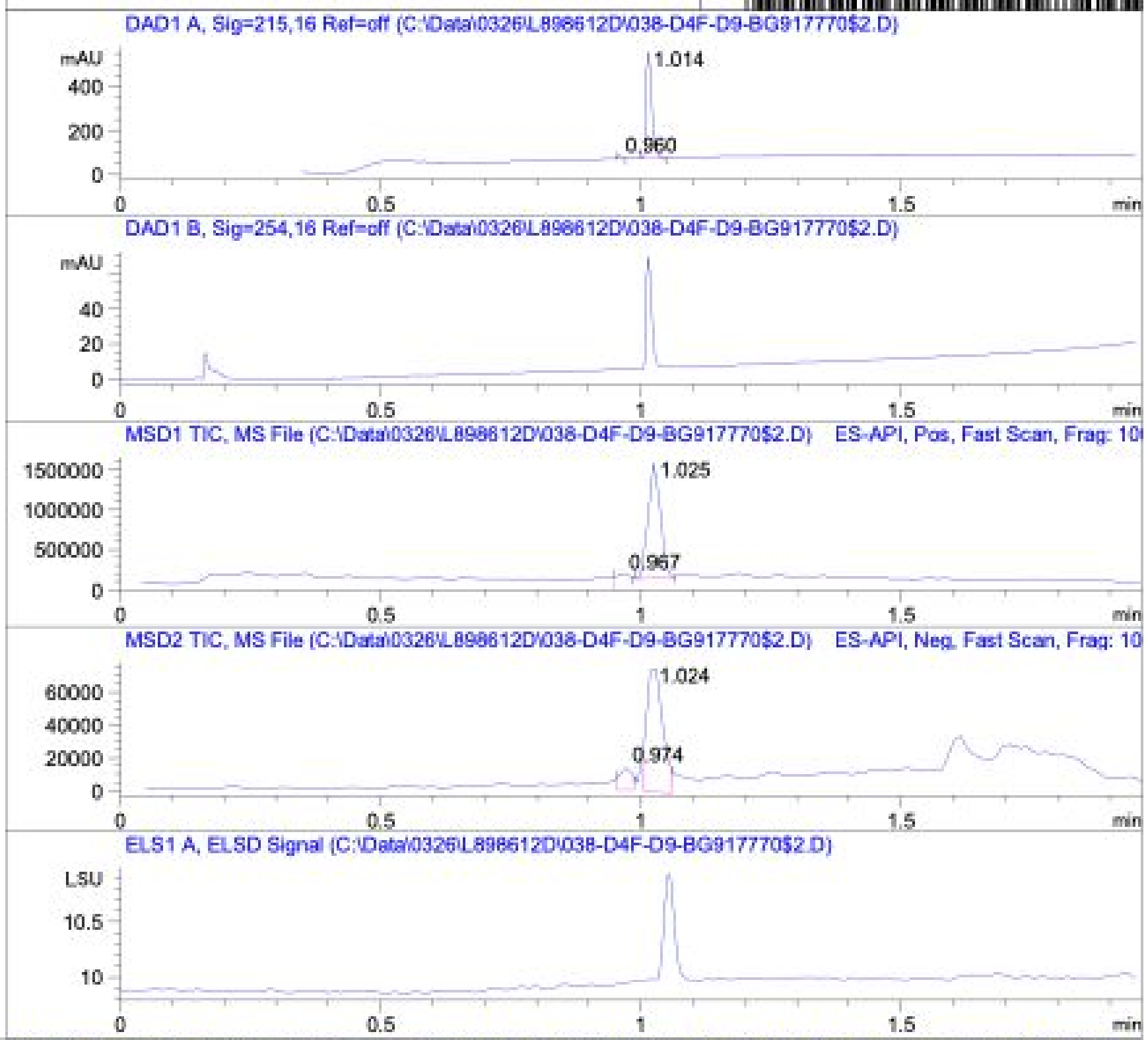

Mol Wt 379.24

Exact Mass 378.08

#	Time	Area%
1	0.960	1.82
2	1.014	98.18

RT 0.967

RT 1.025

RT 0.974

RT 1.024

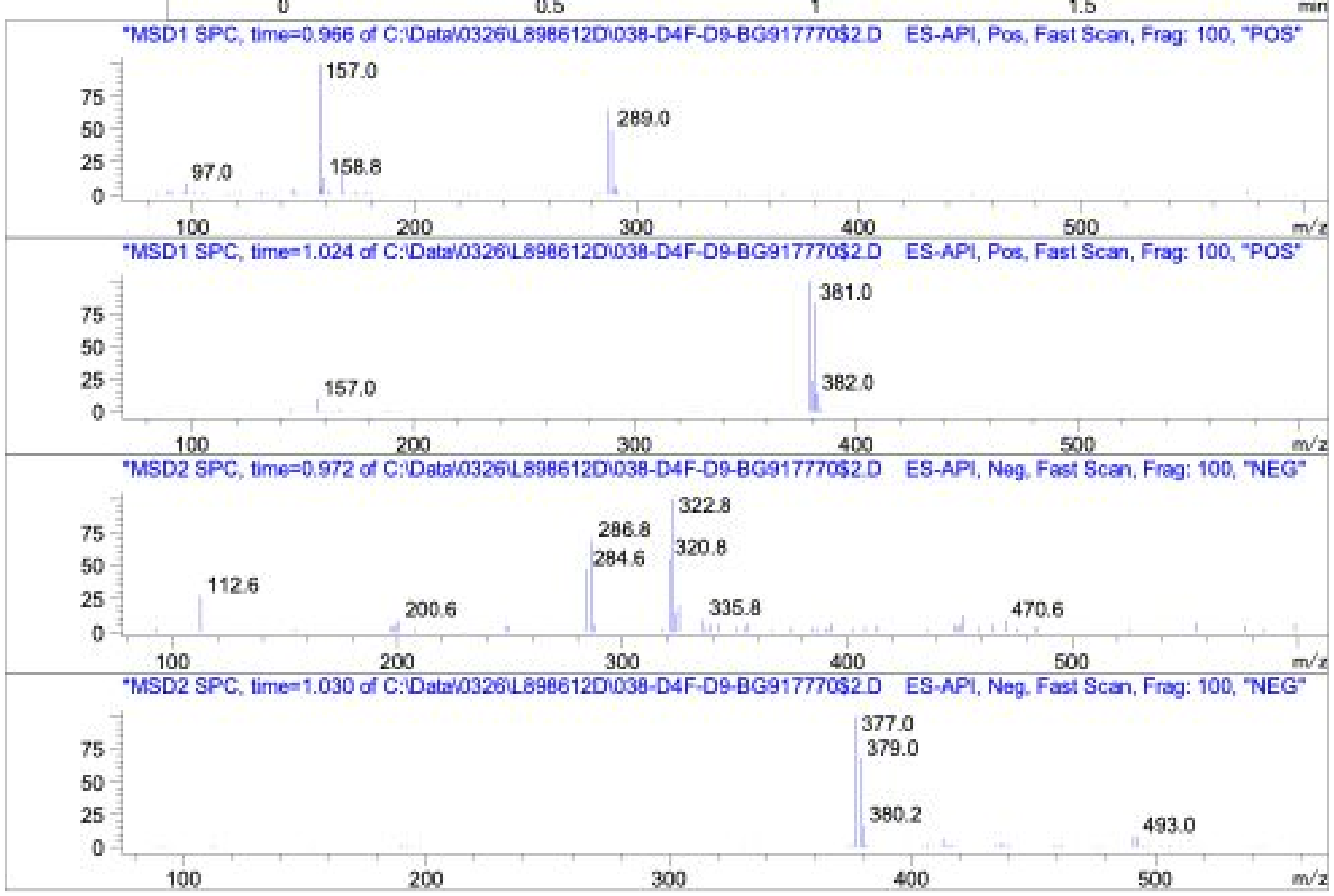

Inj.Date 3/25/2025 KCH
C:\Users\Public\Documents\ChemStation\1\Data\03_25\L898612D\SUPOR_30.M

Z9544921938

MaxPeak: 100.00%
Ret_Time: 1.362 min

BG917772$31

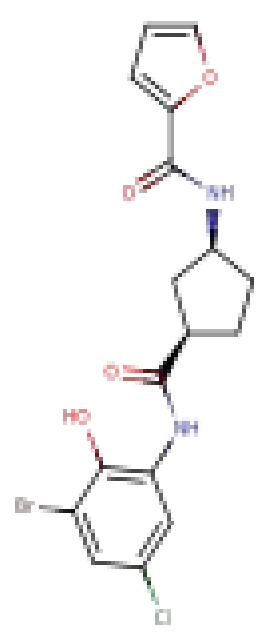

Mol Wt 427.68
Exact Mass 428.01

#	Time	Area%
1	1.362	100.00

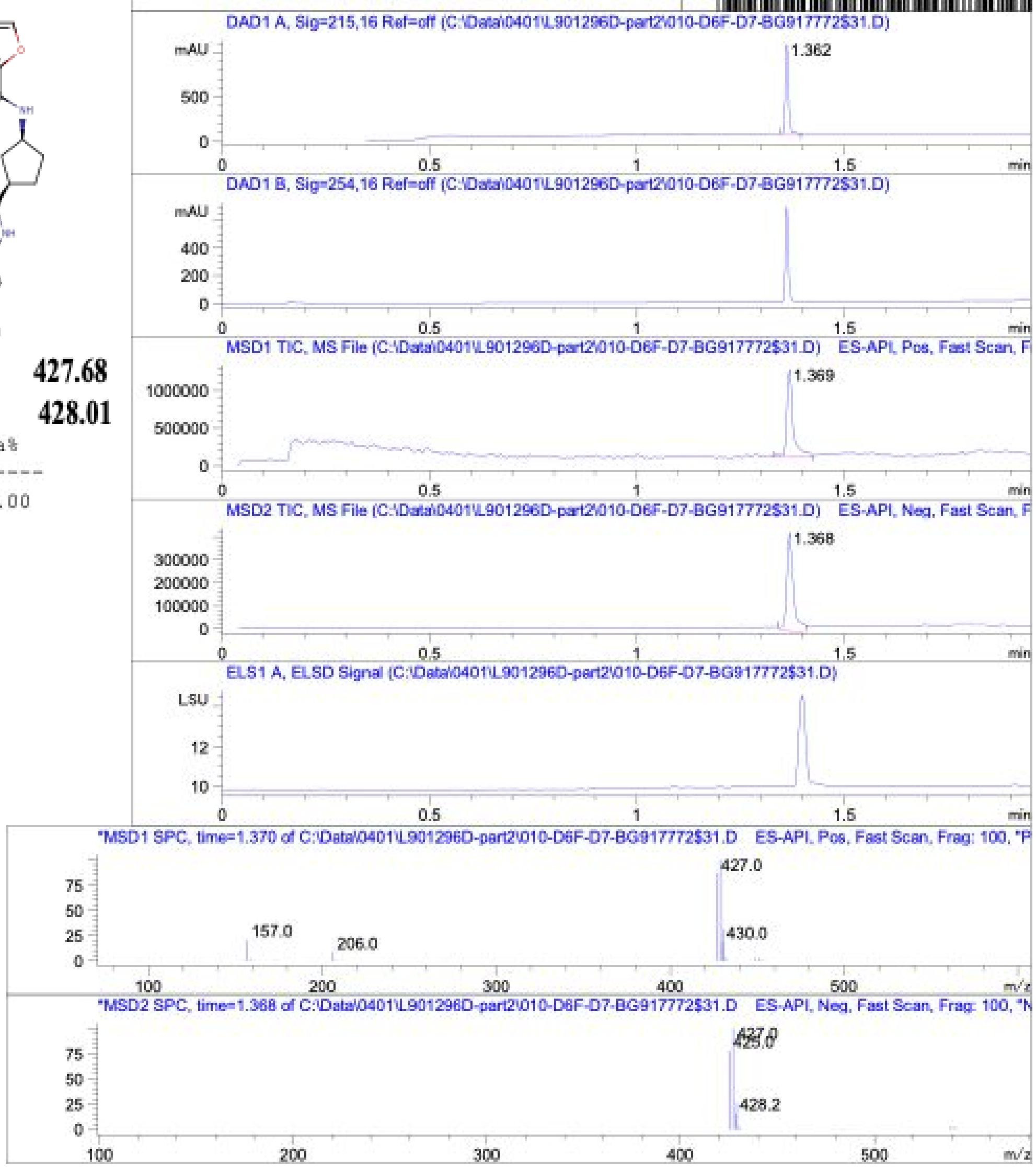

RT 1.369

RT 1.368

Inj.Date 3/31/2025 NEW
C:\Users\Public\Documents\ChemStation\1\Data\03_31\2901296D\SUPOR_30.M

Z9544921944

MaxPeak: 100.00%
Ret_Time: 1.295 min

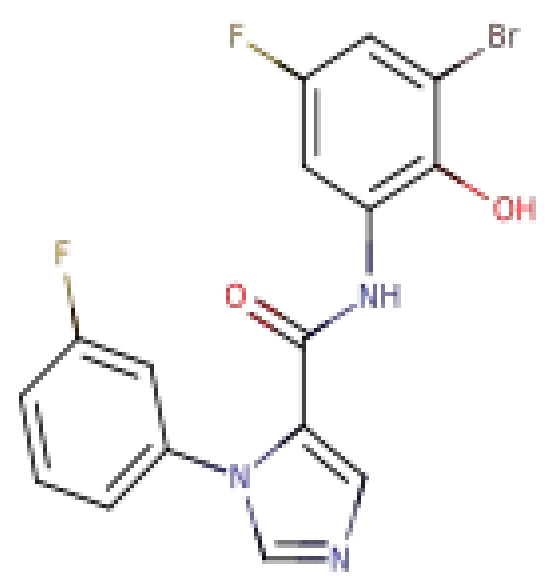

Mol Wt 394.17
Exact Mass 393

#	Time	Area%
1	1.295	100.00

BG917776$42

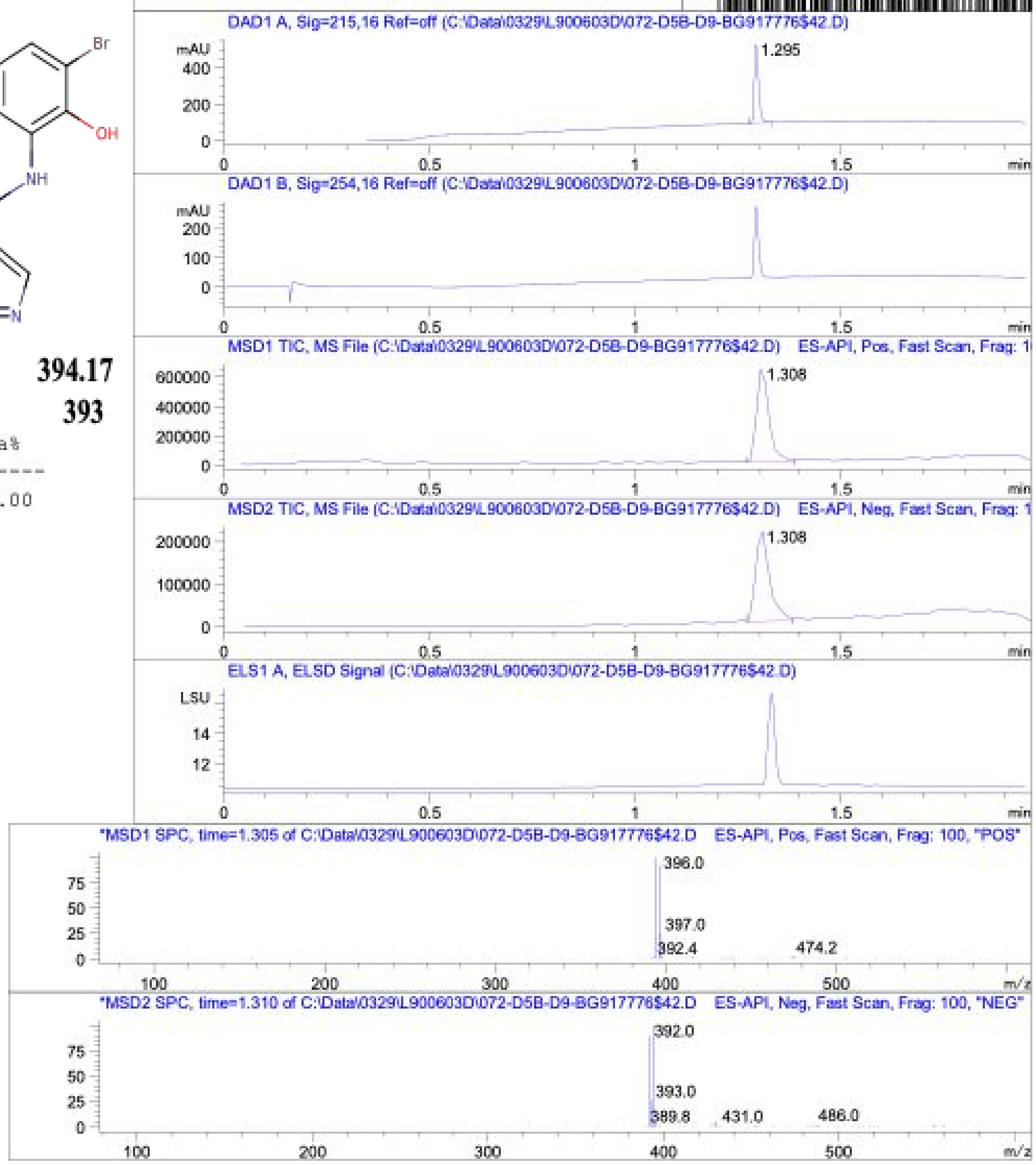

Inj.Date 3/28/2025 NEW - 23 -
C:\Users\Public\Documents\ChemStation\1\Data\03_28\L900603D\SUPOR_30.M

Z9544921971

MaxPeak: 100.00%
Ret_Time: 1.629 min

BG917777$1

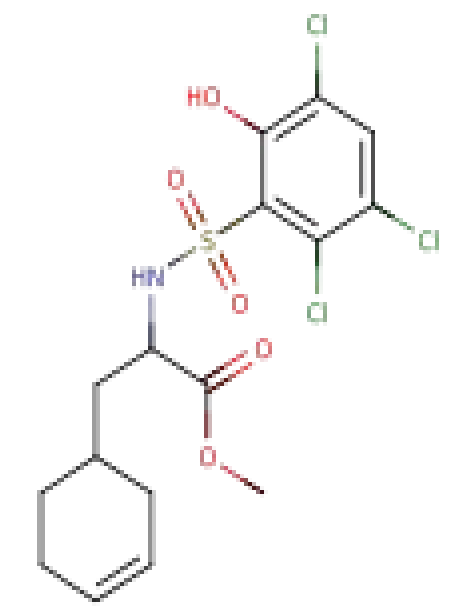

Mol Wt 442.74

Exact Mass 443.01

#	Time	Area%
1	1.629	100.00

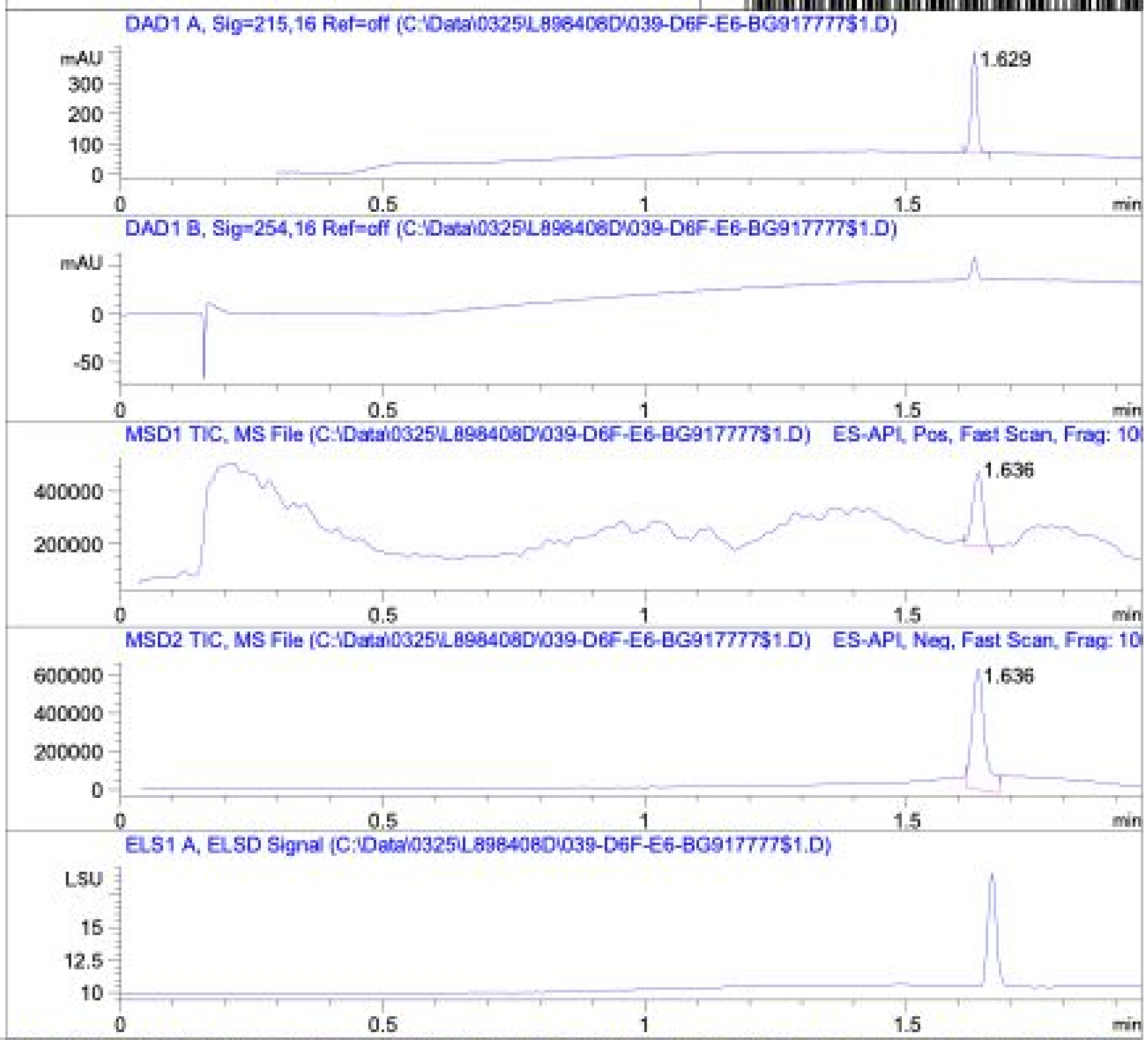

RT 1.636

RT 1.636

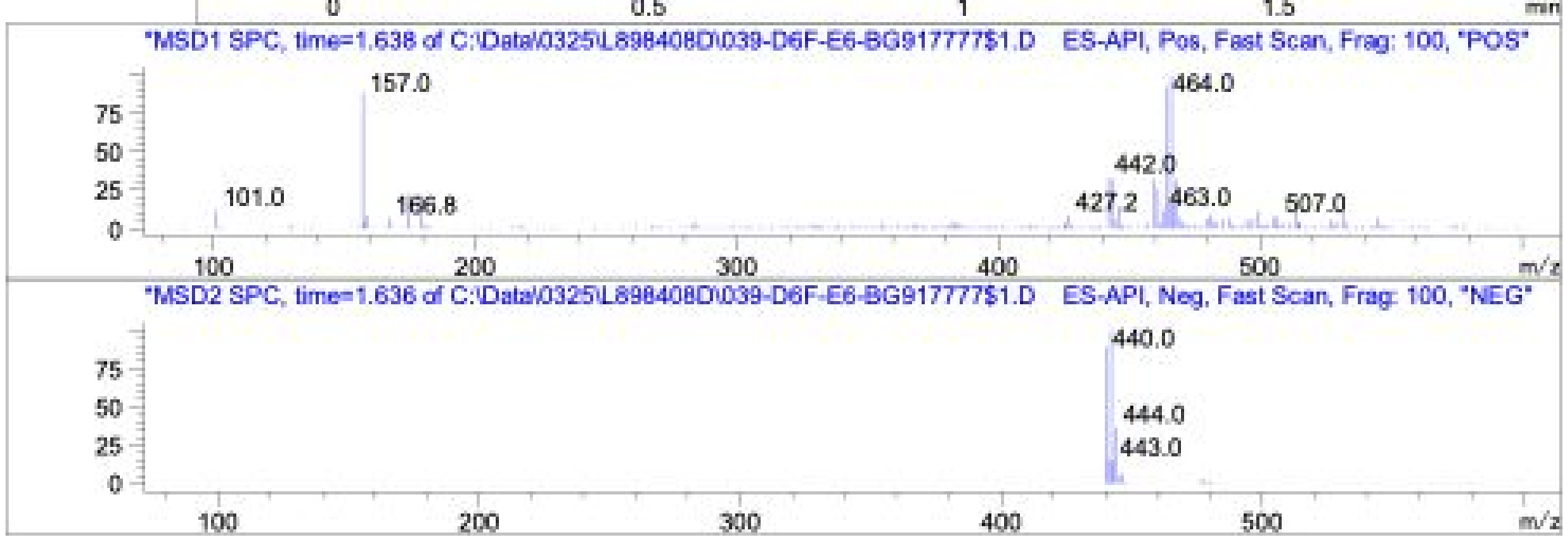

Inj.Date 3/24/2025 NEW -29-
C:\Users\Public\Documents\ChemStation\1\Data\03_24\L898408D\SUPOR_30.M

Z9544921974

MaxPeak: 100.00%
Ret_Time: 1.285 min

BG917783$1

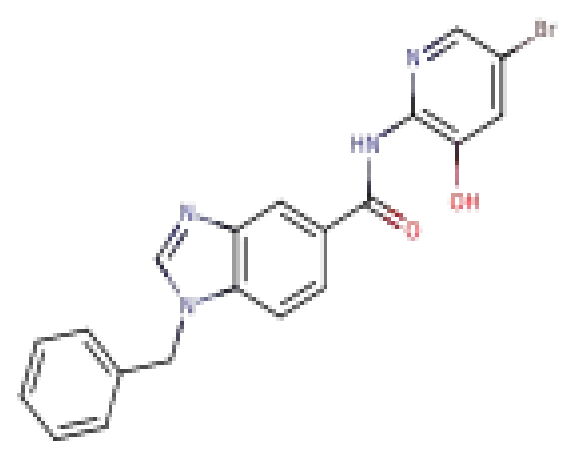

Mol Wt 423.26
Exact Mass 424.05

#	Time	Area%
1	1.285	100.00

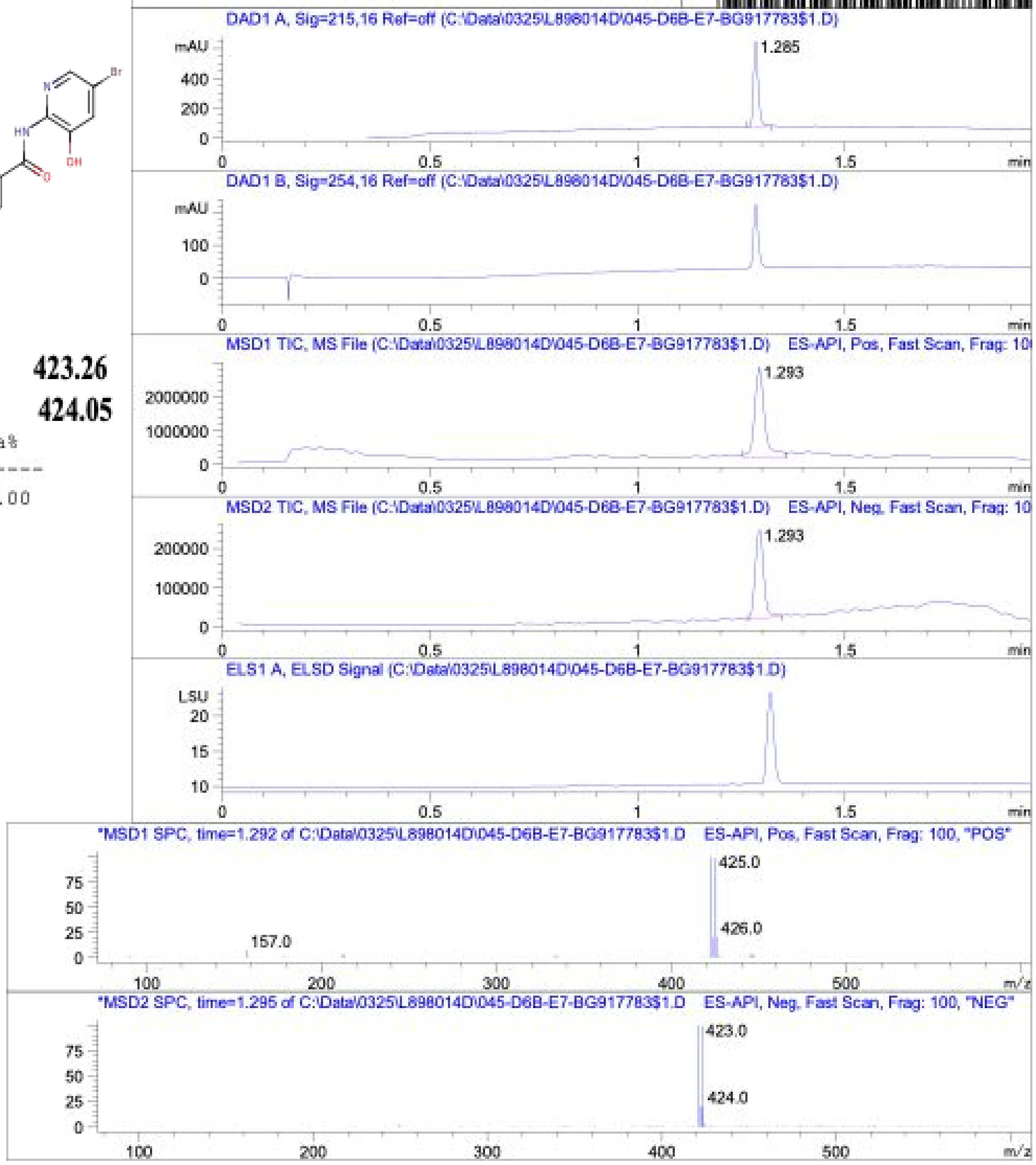

RT 1.293

RT 1.293

Inj.Date 3/24/2025 KCH -29-
C:\Users\Public\Documents\ChemStation\1\Data\03_24\L898014D\SUPOR_30.M

Z9544921992

MaxPeak: 58.16%
Ret_Time: 1.216 min

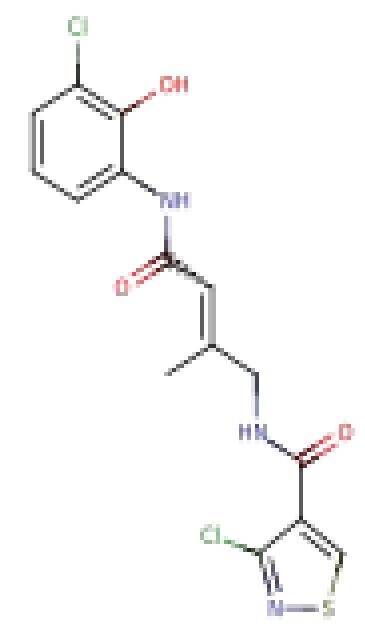

Mol Wt 386.25
Exact Mass 385.01

#	Time	Area%
1	0.904	4.38
2	1.022	3.19
3	1.216	58.16
4	1.224	34.27

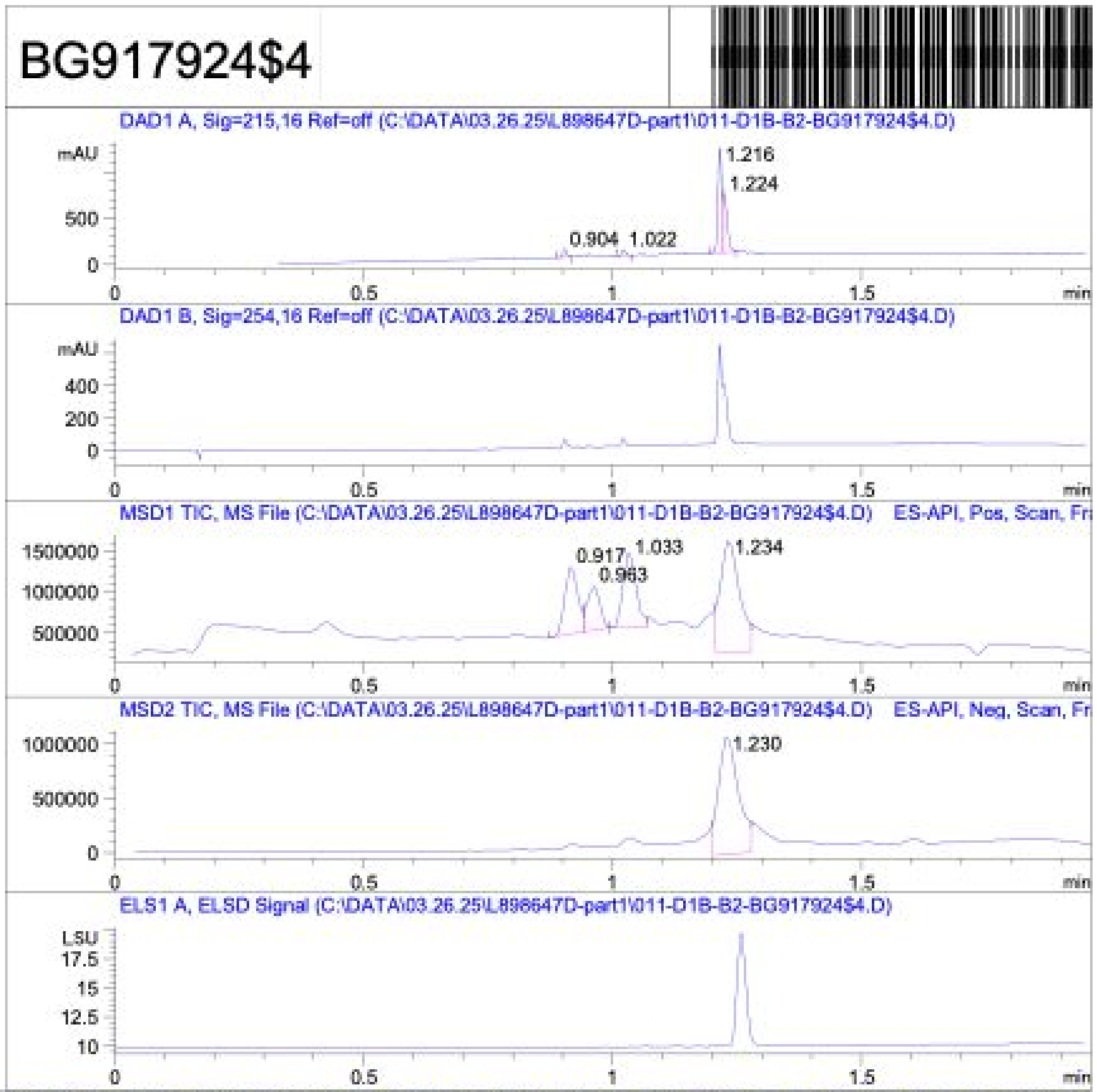

RT 0.917

RT 0.963

RT 1.033

RT 1.234

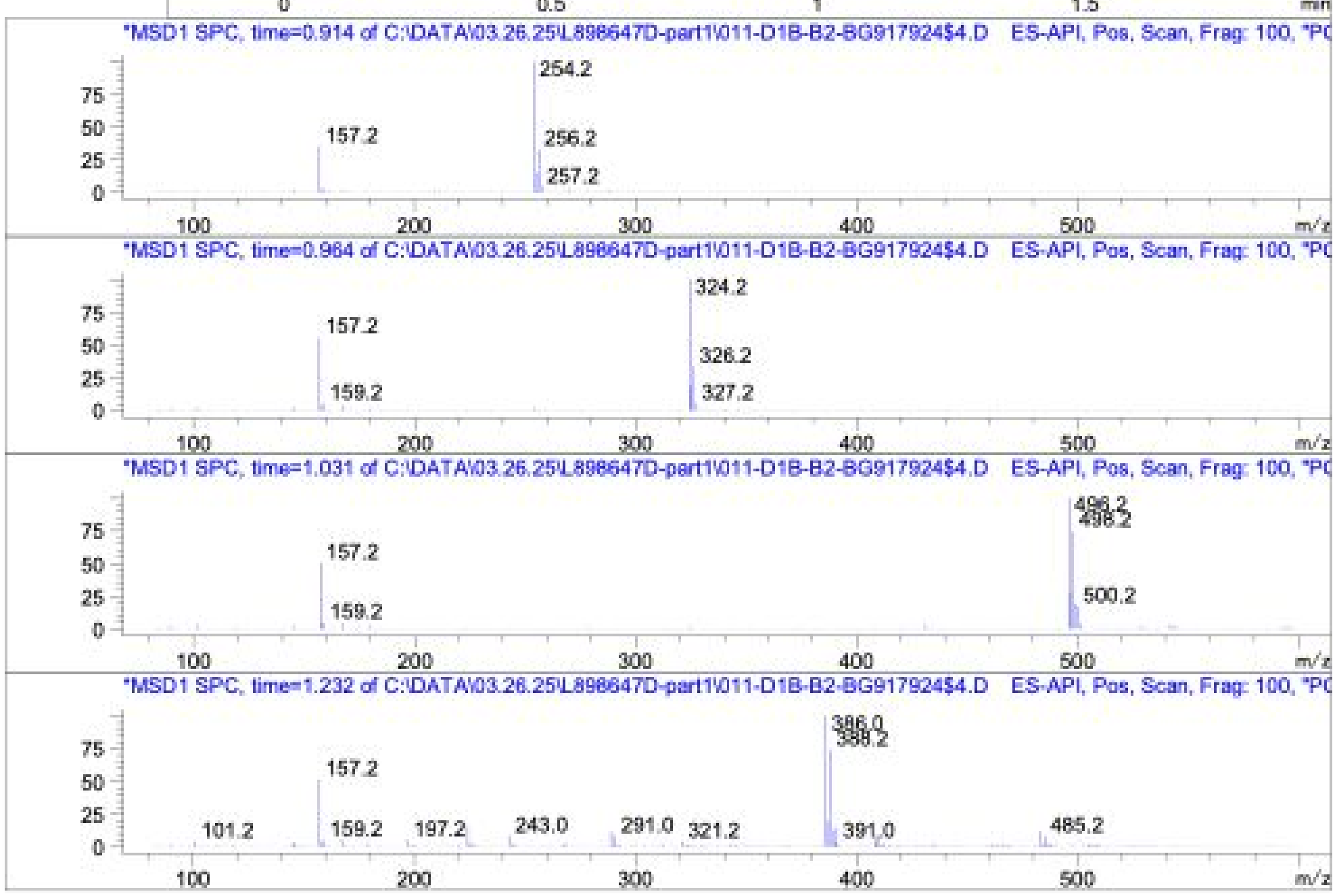

Inj.Date 3/25/2025 NEW -5-
C:\Users\Public\Documents\ChemStation\1\Data\03_25\1898647D\SUPOR_96.M

Z9544922022

MaxPeak: 100.00%
Ret_Time: 0.970 min

BG917790$1

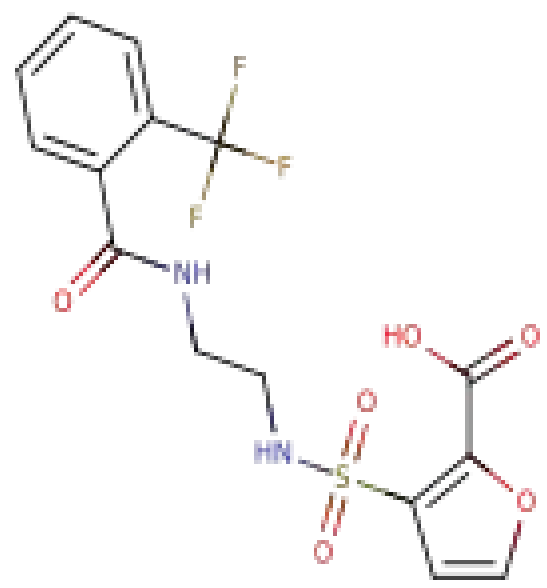

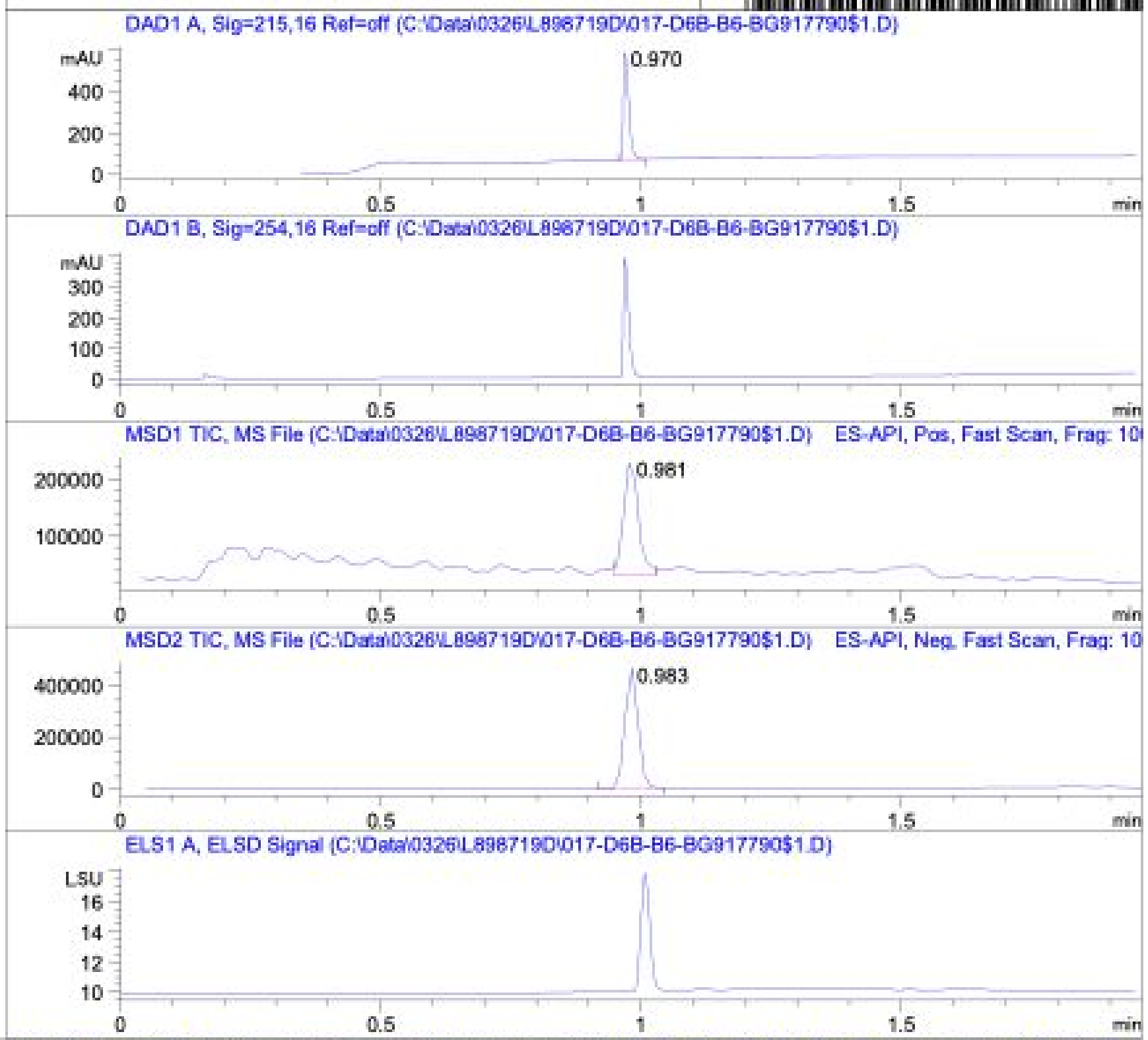

Mol Wt 406.34

Exact Mass 406.04

#	Time	Area%
1	0.970	100.00

RT 0.981

RT 0.983

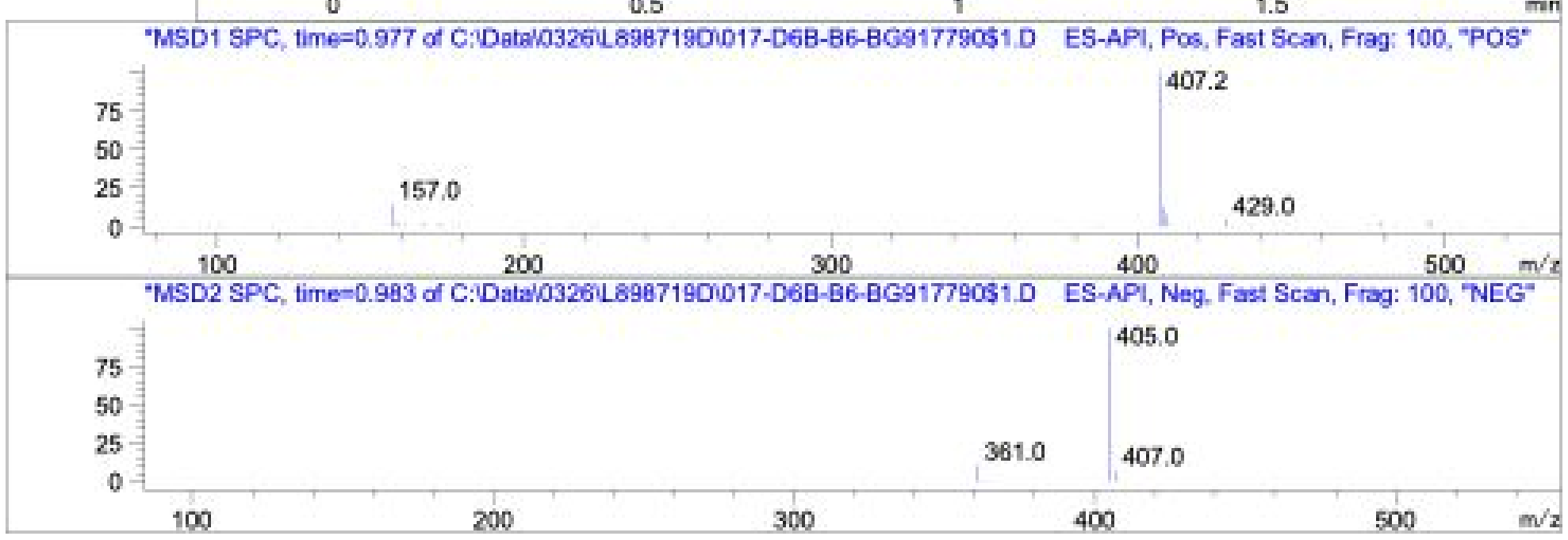

Inj.Date 3/25/2025 NEW
C:\Users\Public\Documents\ChemStation\1\Data\03_25\L898719D\SUPOR_30.M

Z9544922024

MaxPeak: 92.28%
Ret_Time: 1.245 min

T9073785

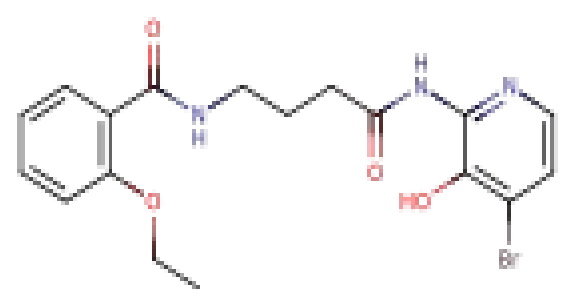

Mol Wt 422.27
Exact Mass 421.08

#	Time	Area%
1	0.493	4.34
2	1.028	3.38
3	1.245	92.28

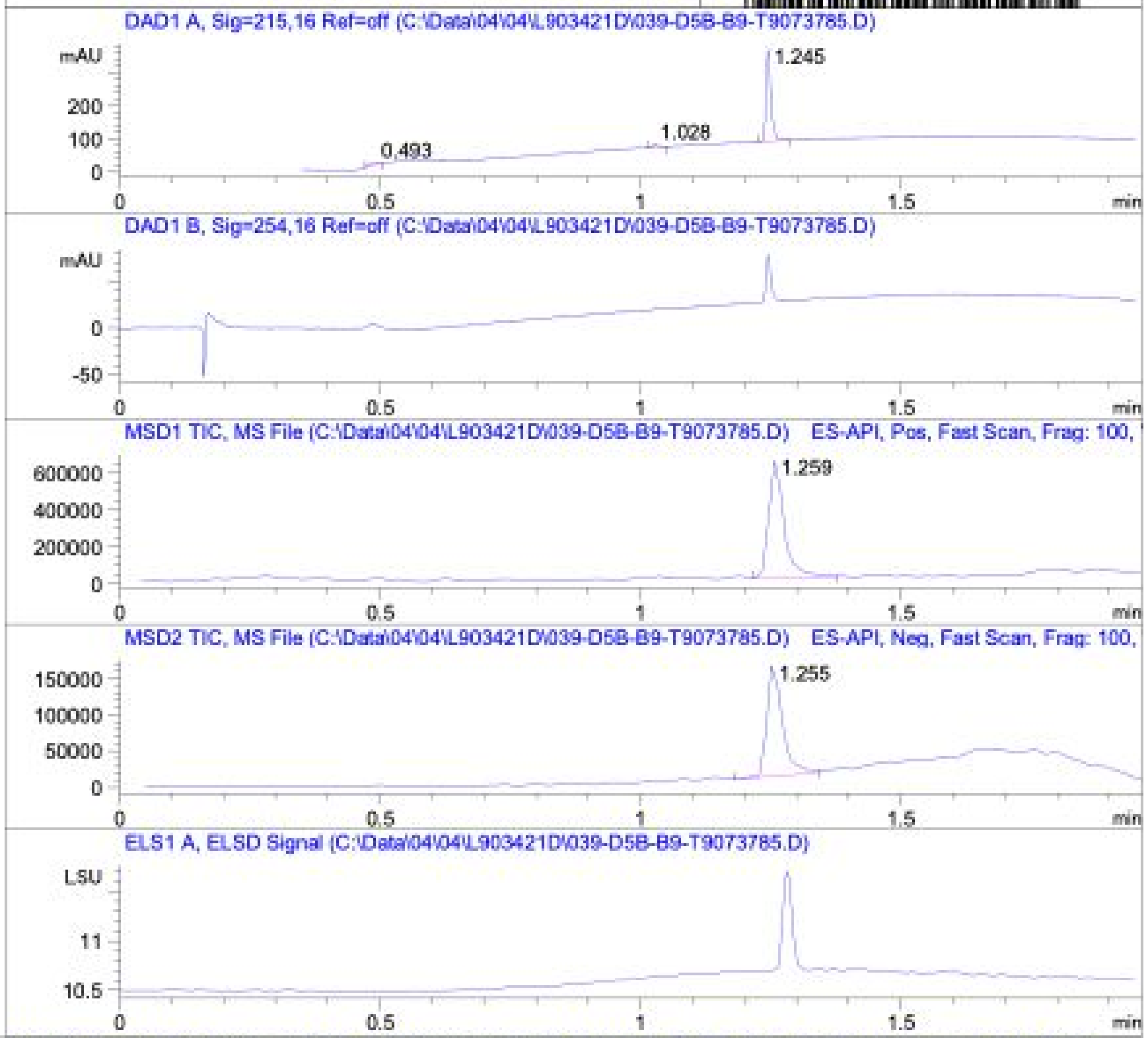

RT 1.259

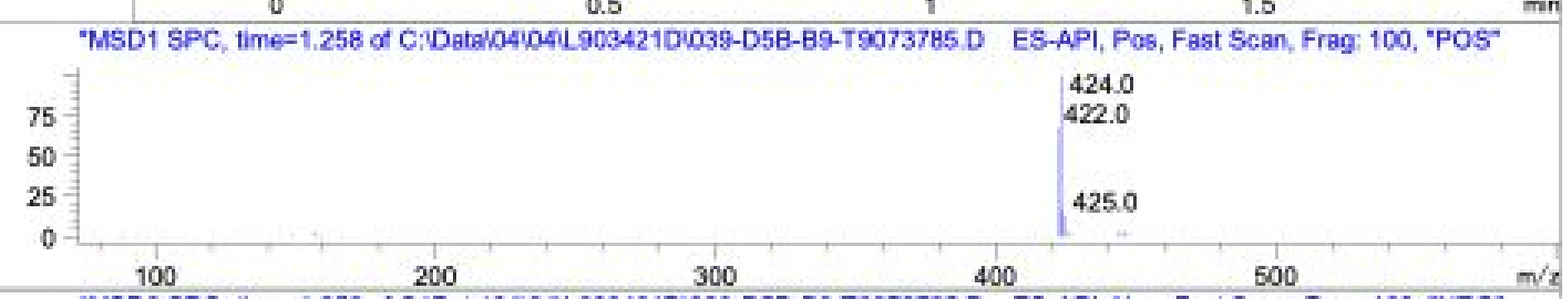

RT 1.255

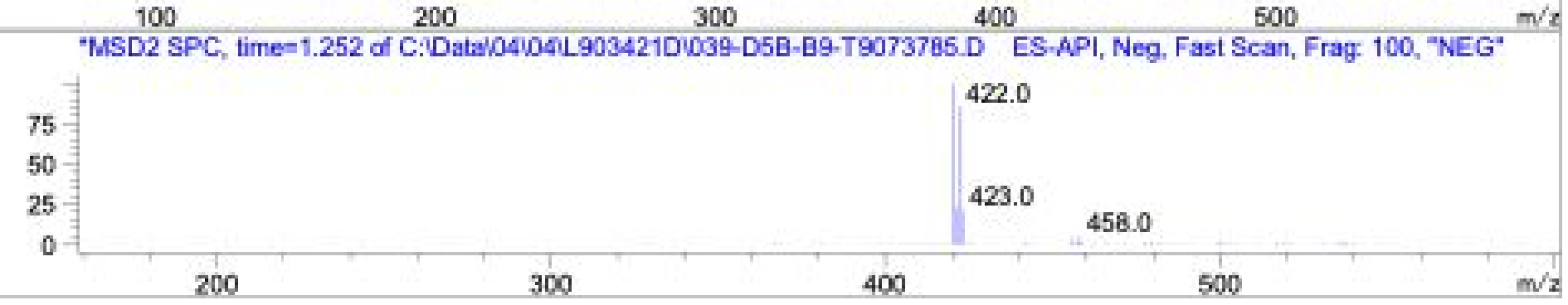

Inj.Date 4/3/2025 C - 23 -
C:\Users\Public\Documents\ChemStation\1\Data\04_03\L903421D\SUPOR_30.M

Z9544922050

MaxPeak: 99.03%
Ret_Time: 1.495 min

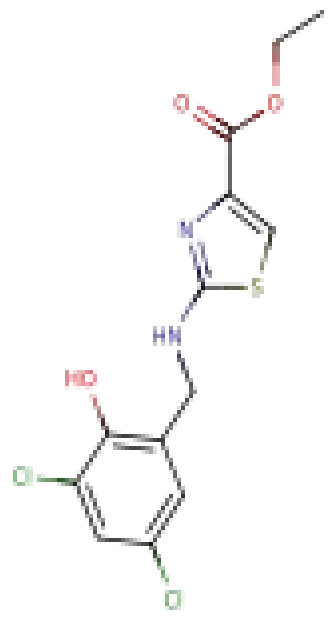

Mol Wt 347.22
Exact Mass 346

#	Time	Area%
1	1.090	0.97
2	1.495	99.03

BH178569$3

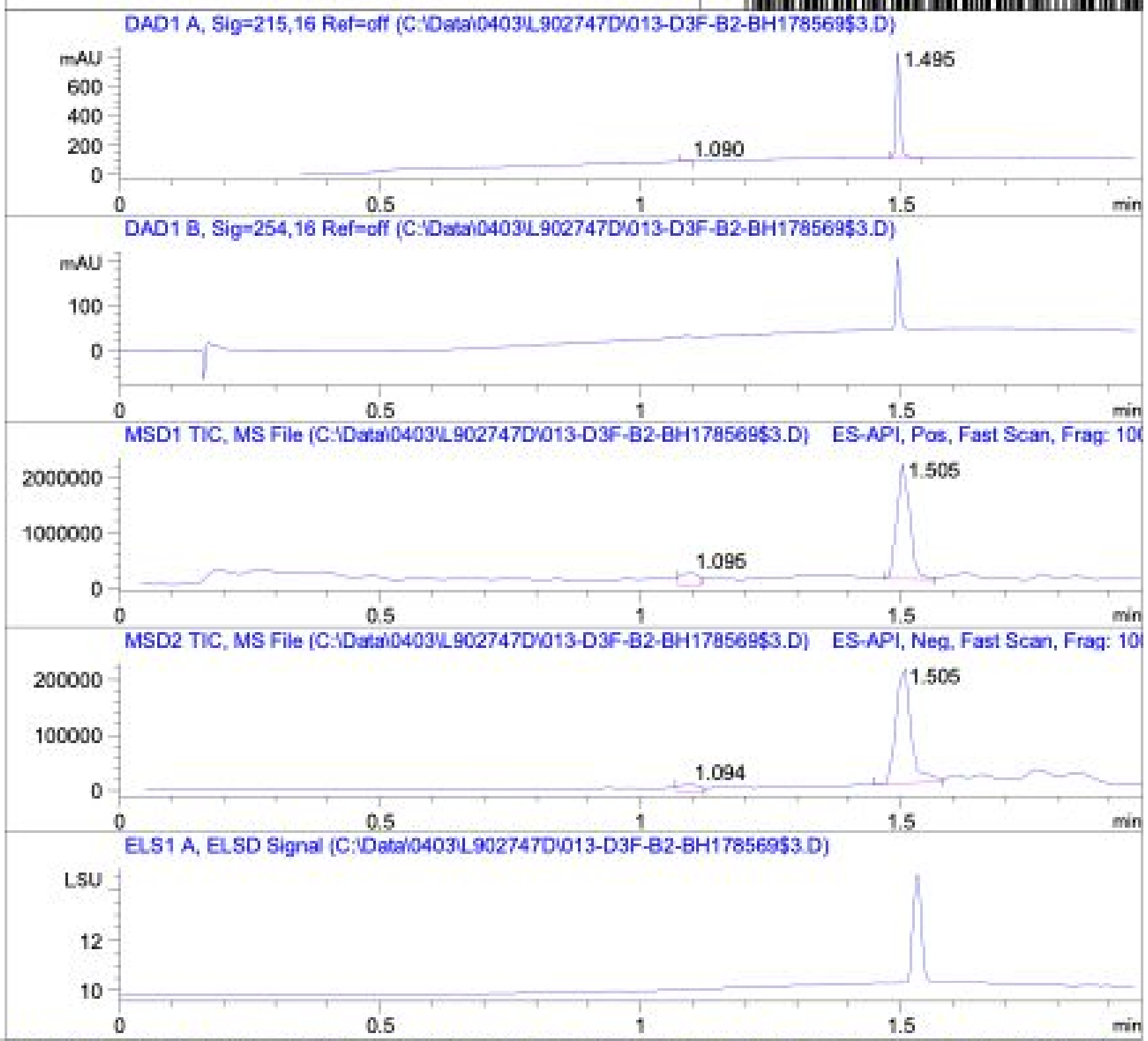

RT 1.095

RT 1.505

RT 1.094

RT 1.505

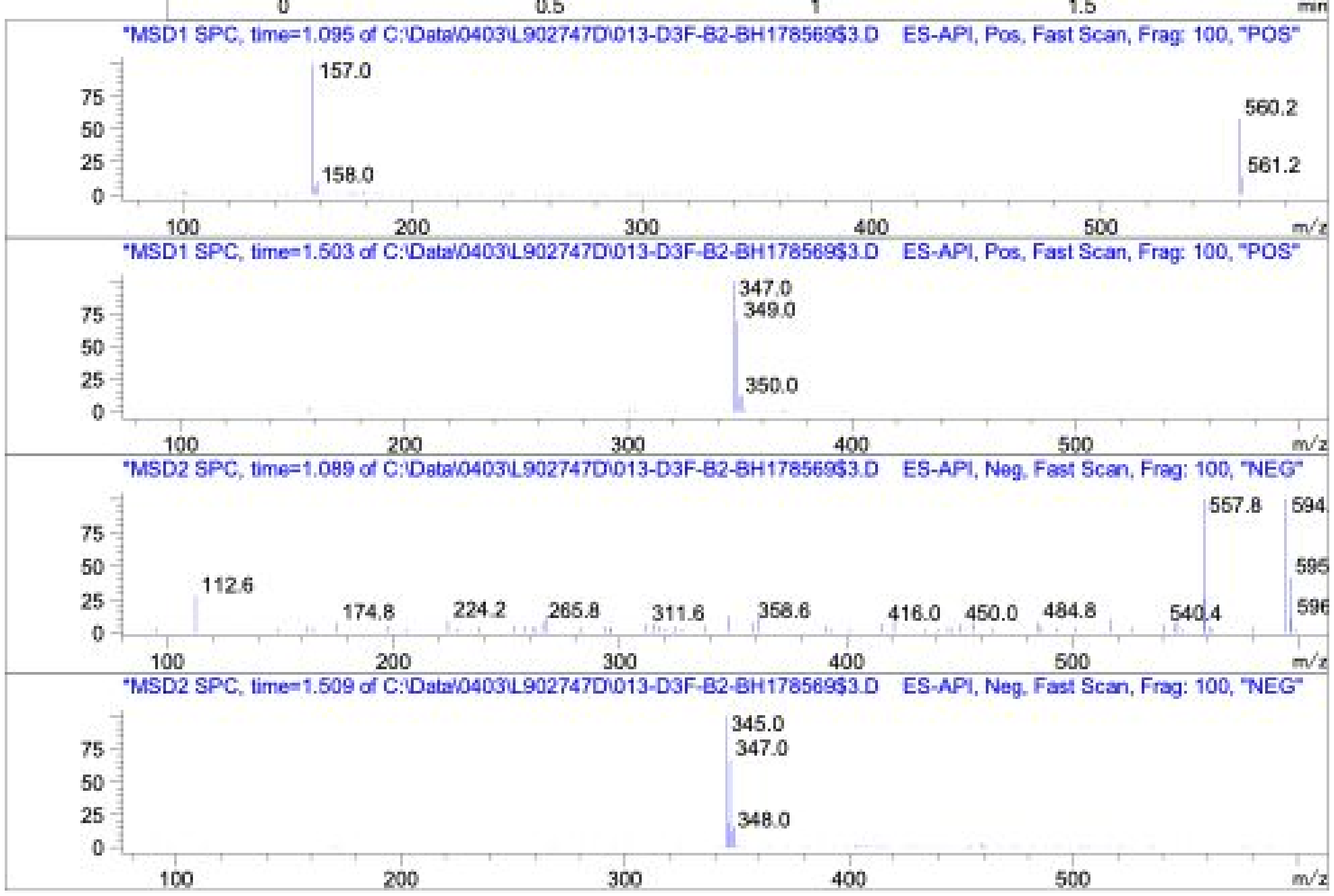

Inj.Date 4/2/2025 NEW
C:\Users\Public\Documents\ChemStation\1\Data\04_02\L902747D\SUPOR_30.M

Z9544922070

MaxPeak: 100.00%
Ret_Time: 1.128 min

BG917801$3

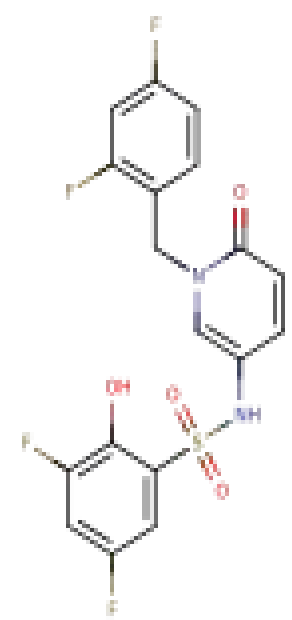

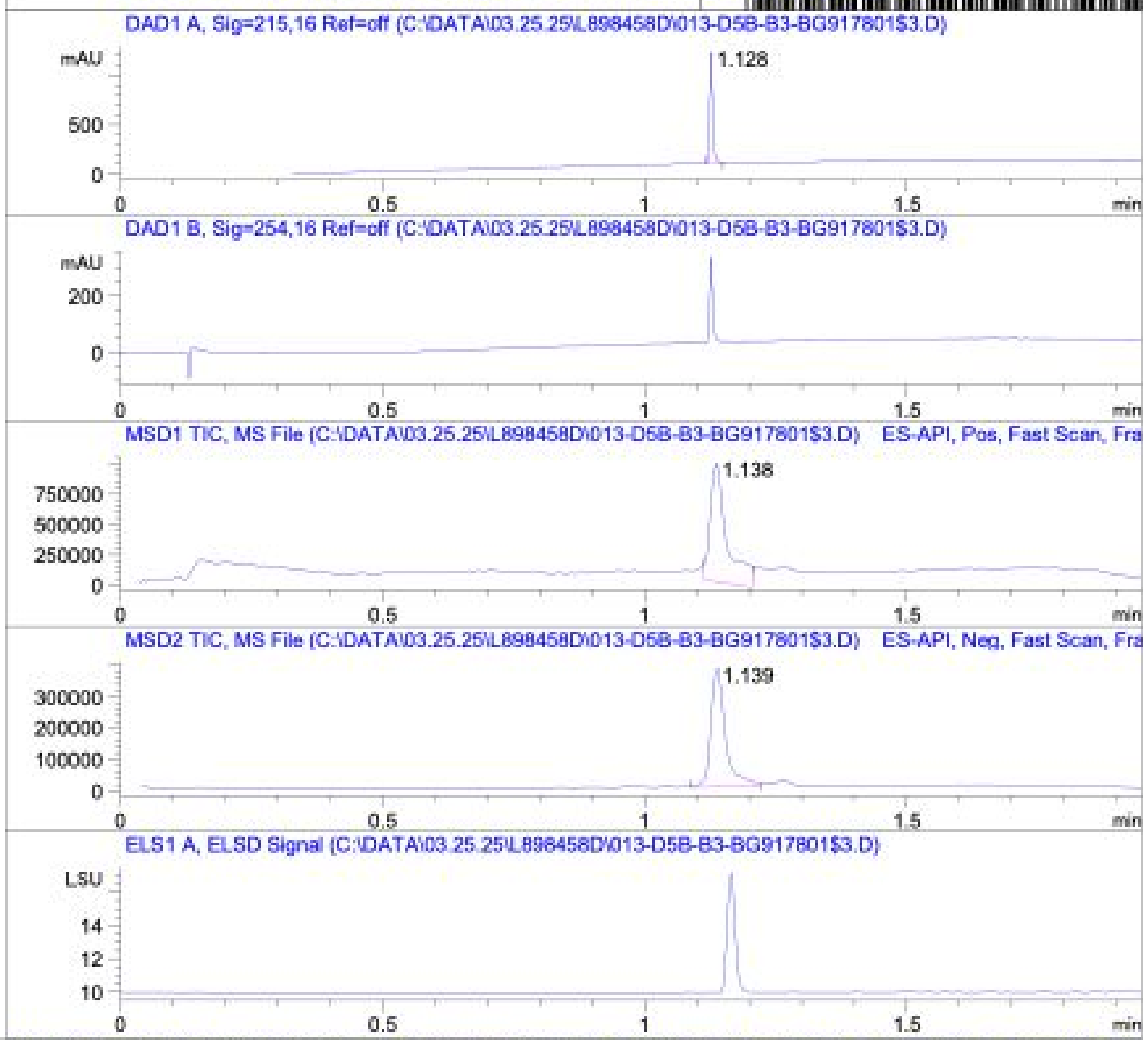

Mol Wt 428.36

Exact Mass 428.05

#	Time	Area%
1	1.128	100.00

RT 1.138

RT 1.139

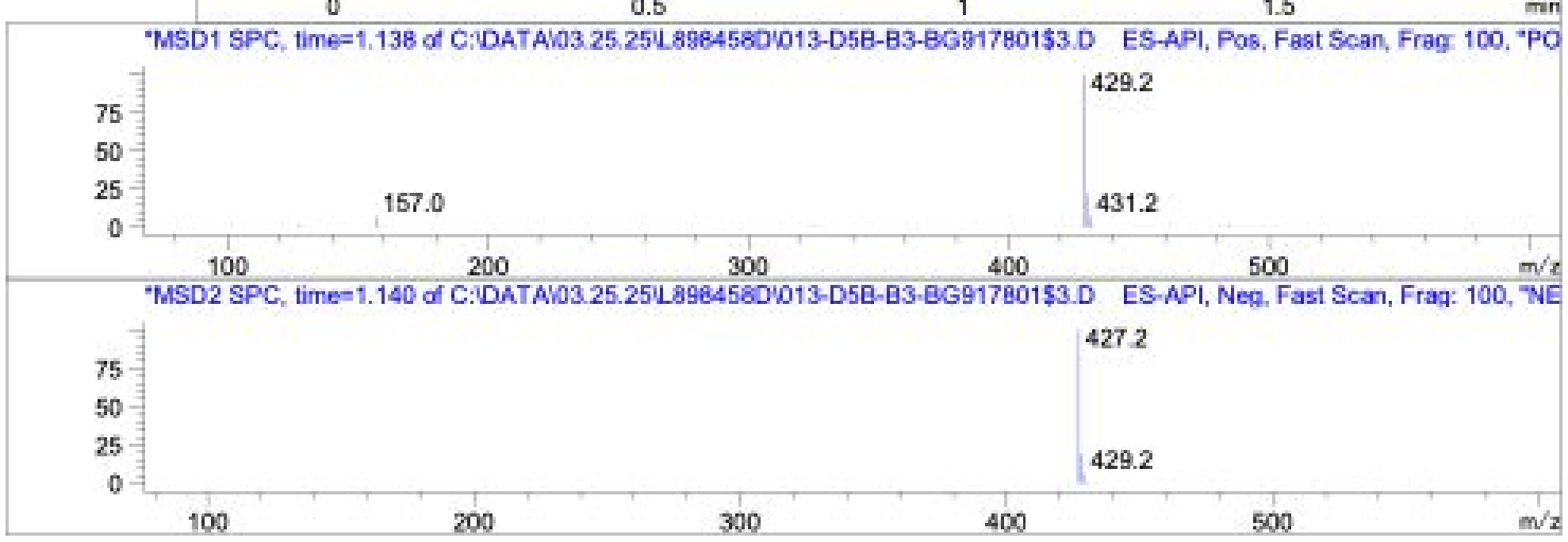

Inj.Date 3/24/2025 NEW
C:\Users\Public\Documents\ChemStation\1\Data\03_24\L898458D\SUPOR_30.M

Z9544922097

MaxPeak: 97.35%
Ret_Time: 1.540 min

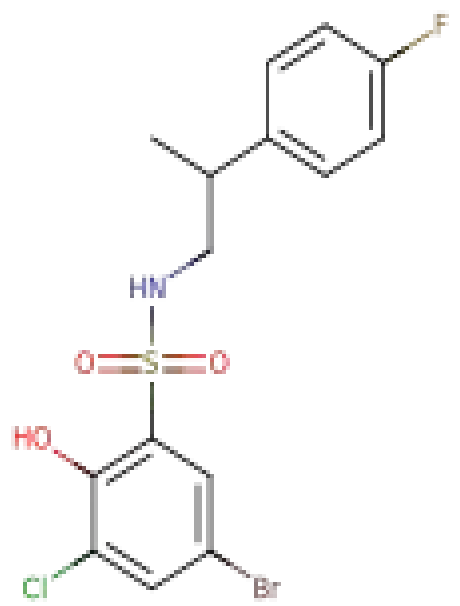

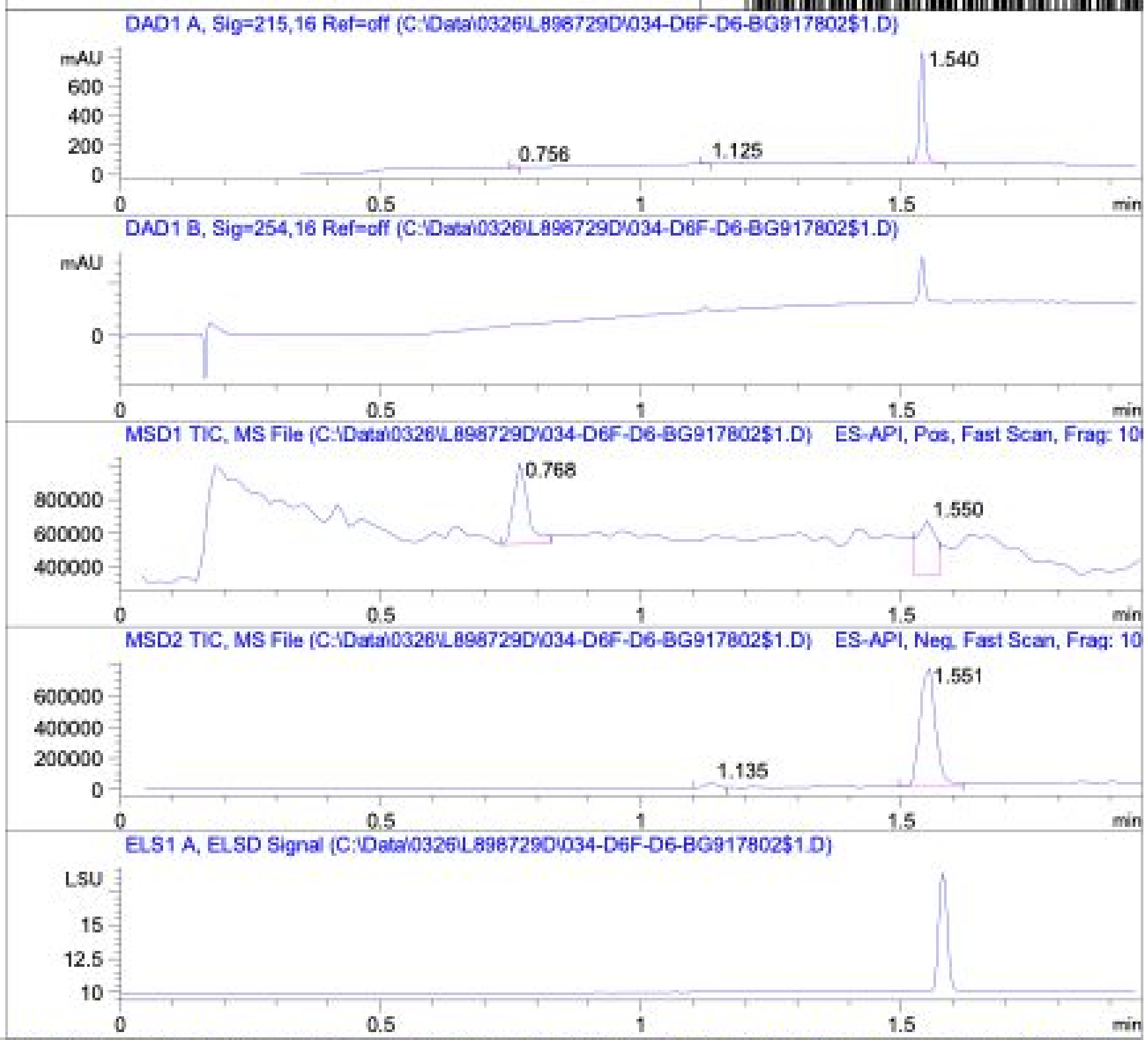

Mol Wt 422.7

Exact Mass 422.97

#	Time	Area%
1	0.756	1.23
2	1.125	1.42
3	1.540	97.35

RT 0.768

RT 1.550

RT 1.135

RT 1.551

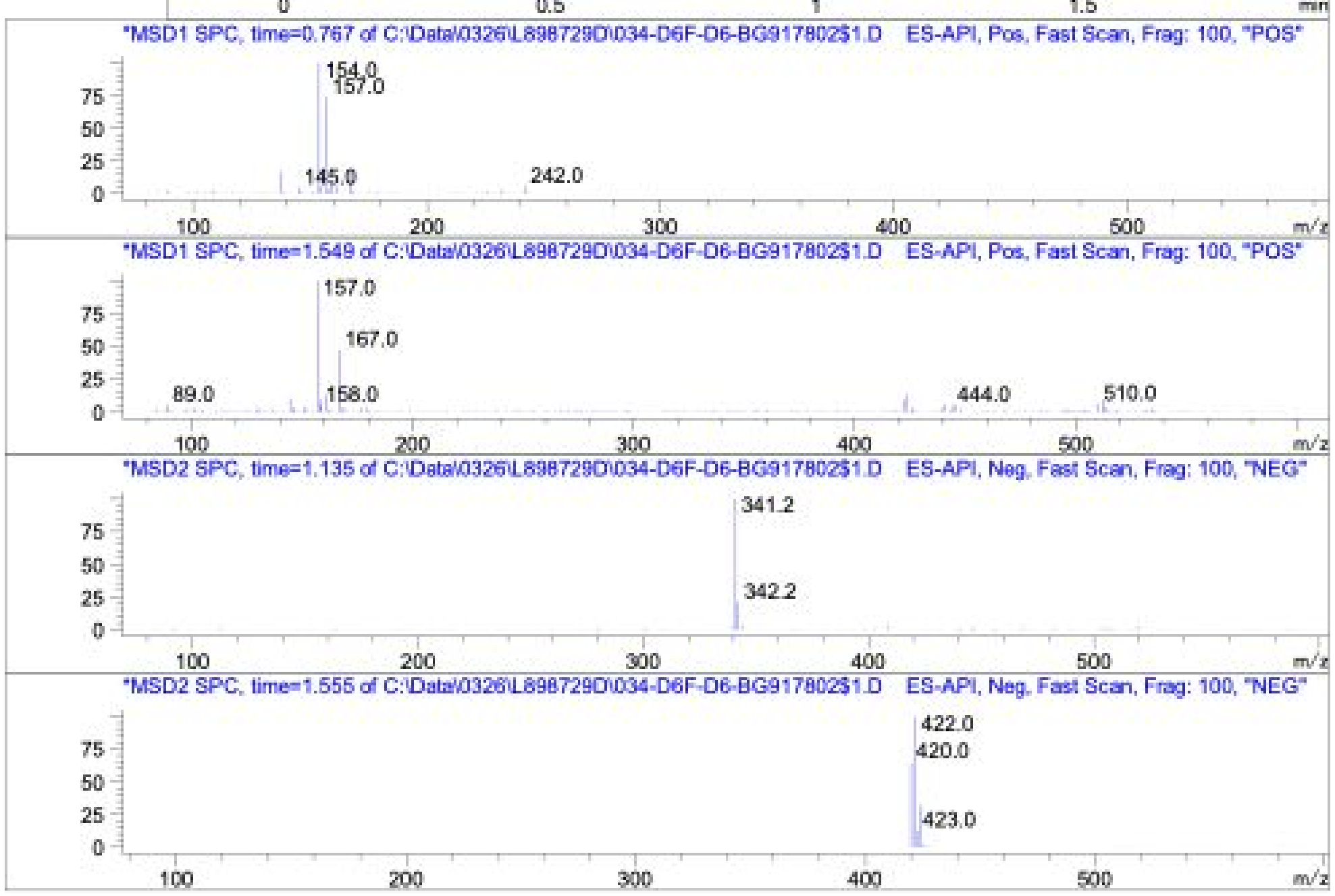

Inj.Date 3/25/2025 NEW
C:\Users\Public\Documents\ChemStation\1\Data\03_25\L898729D\SUPOR_30.M

Z9544922098

MaxPeak: 95.47%
Ret_Time: 1.194 min

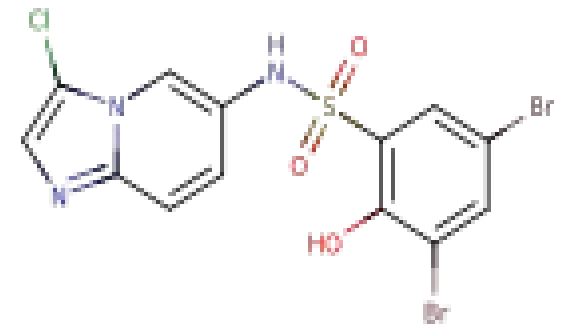

Mol Wt 481.55

Exact Mass 480.83

#	Time	Area%
1	1.194	95.47
2	1.326	4.53

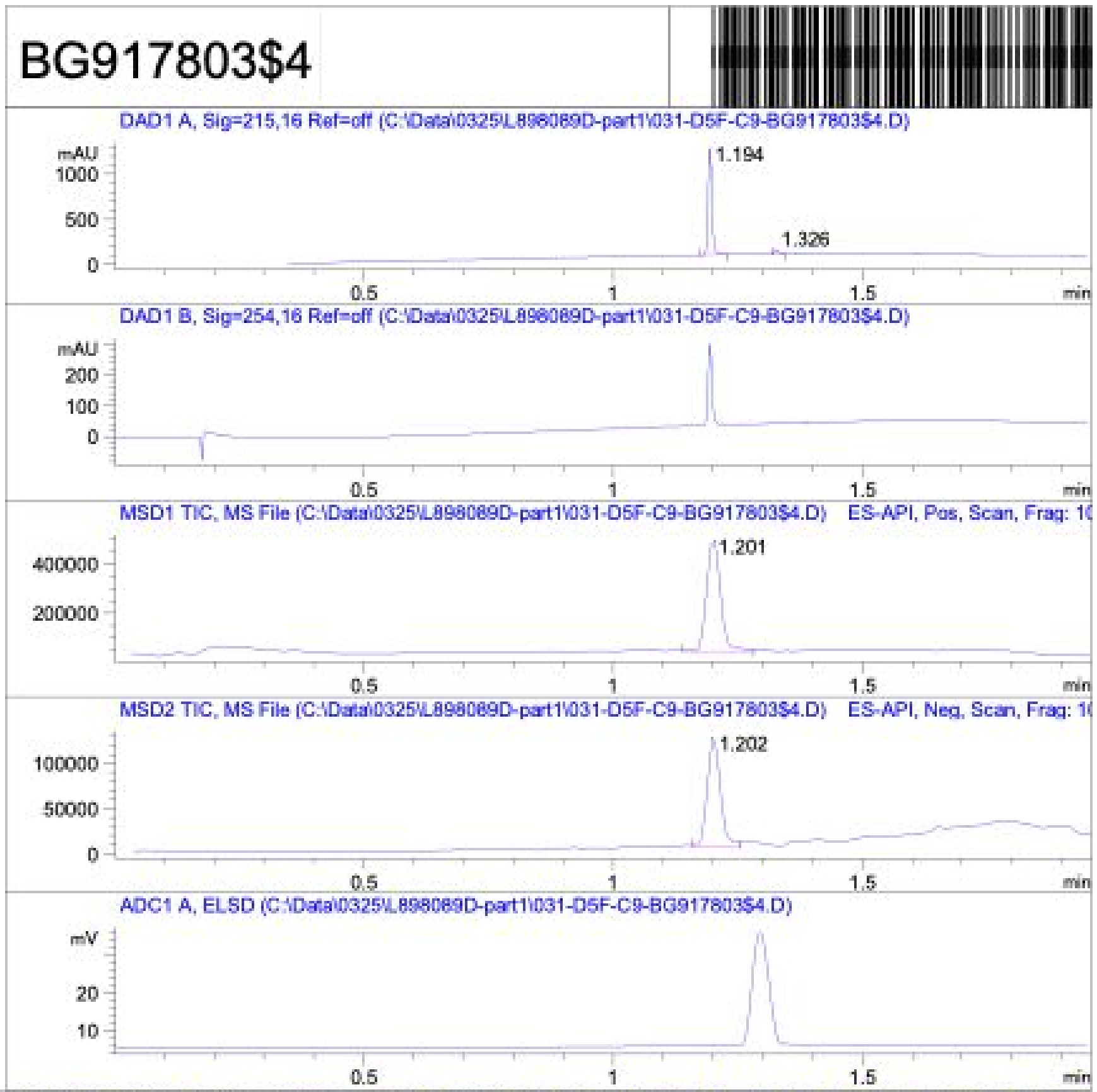

RT 1.201

RT 1.202

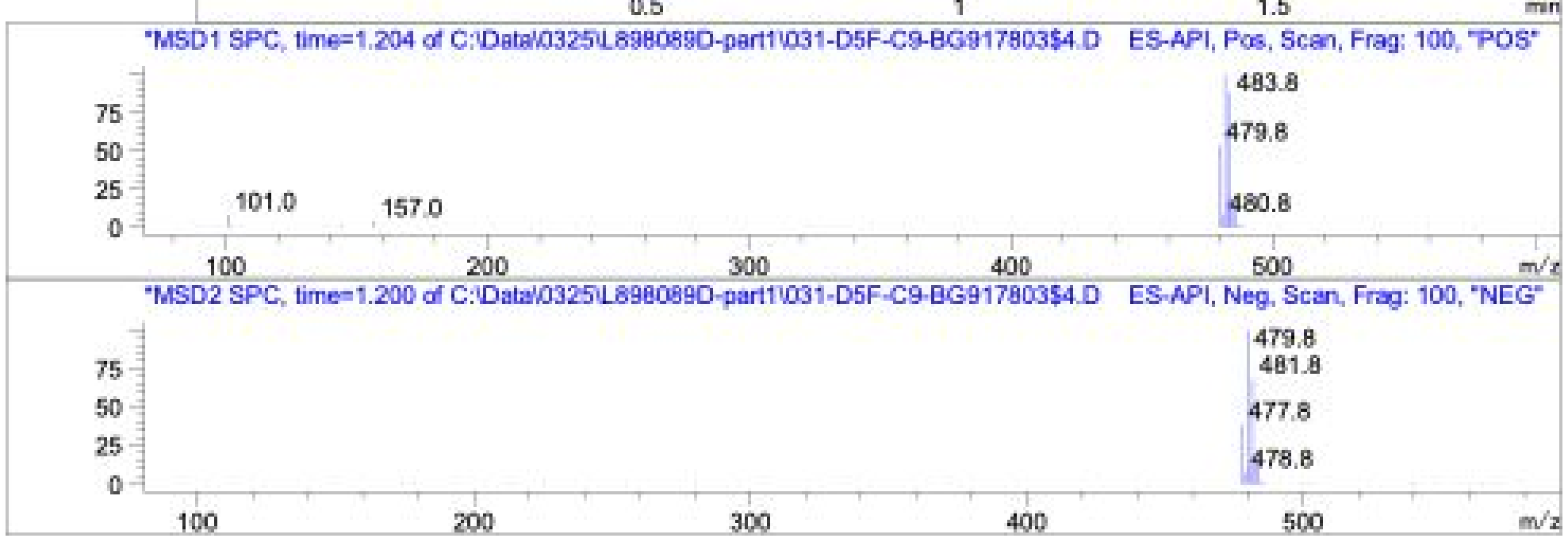

Inj.Date 3/24/2025 NEW -6-
C:\Users\Public\Documents\ChemStation\1\Data\03_24\1898089D\SUPOR_30.M

Z9544922102

MaxPeak: 97.99%
Ret_Time: 1.218 min

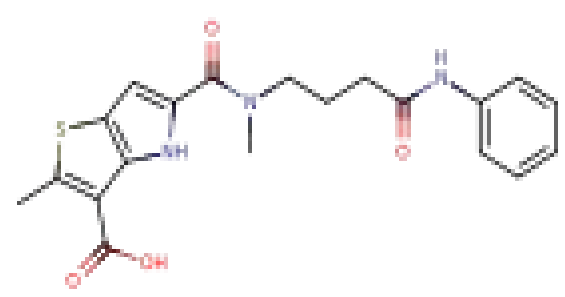

Mol Wt 399.46

Exact Mass 399.14

#	Time	Area%
1	1.071	2.01
2	1.218	97.99

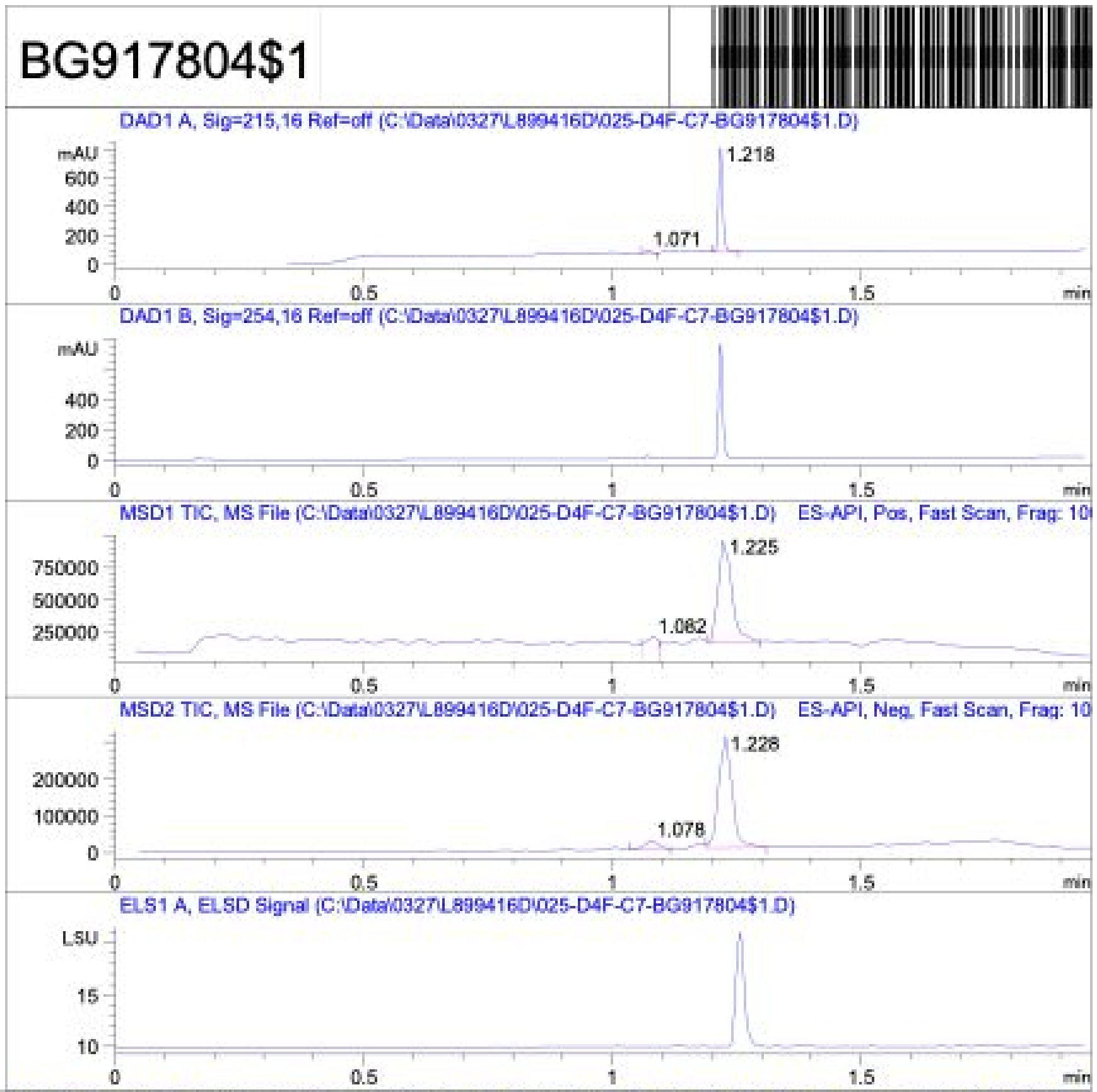

RT 1.082

RT 1.225

RT 1.078

RT 1.228

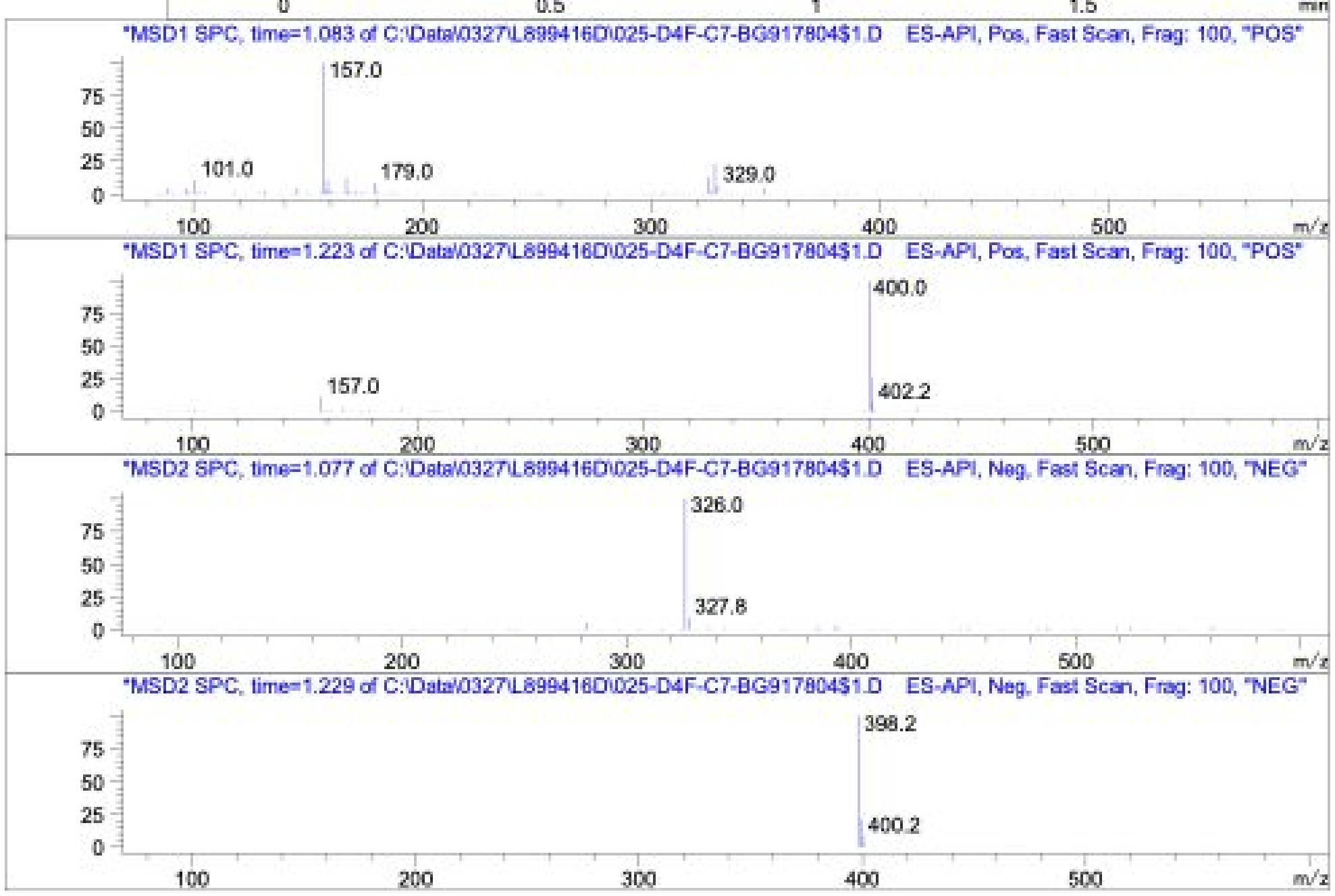

Inj.Date 3/26/2025 NEW
C:\Users\Public\Documents\ChemStation\1\Data\03_26\L899416D\SUPOR_30.M

Z9544922108

MaxPeak: 95.36%
Ret_Time: 1.442 min

BH153543$1

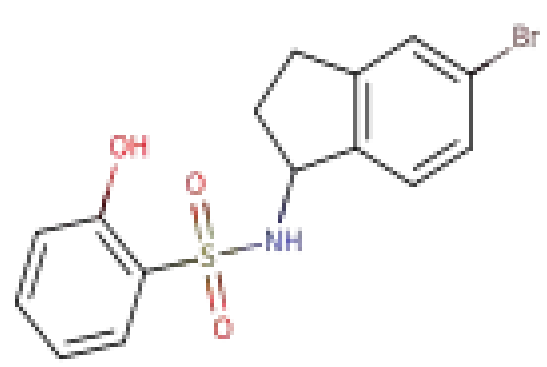

Mol Wt 368.25
Exact Mass 369

#	Time	Area%
1	1.319	1.64
2	1.391	1.43
3	1.442	95.36
4	1.531	1.56

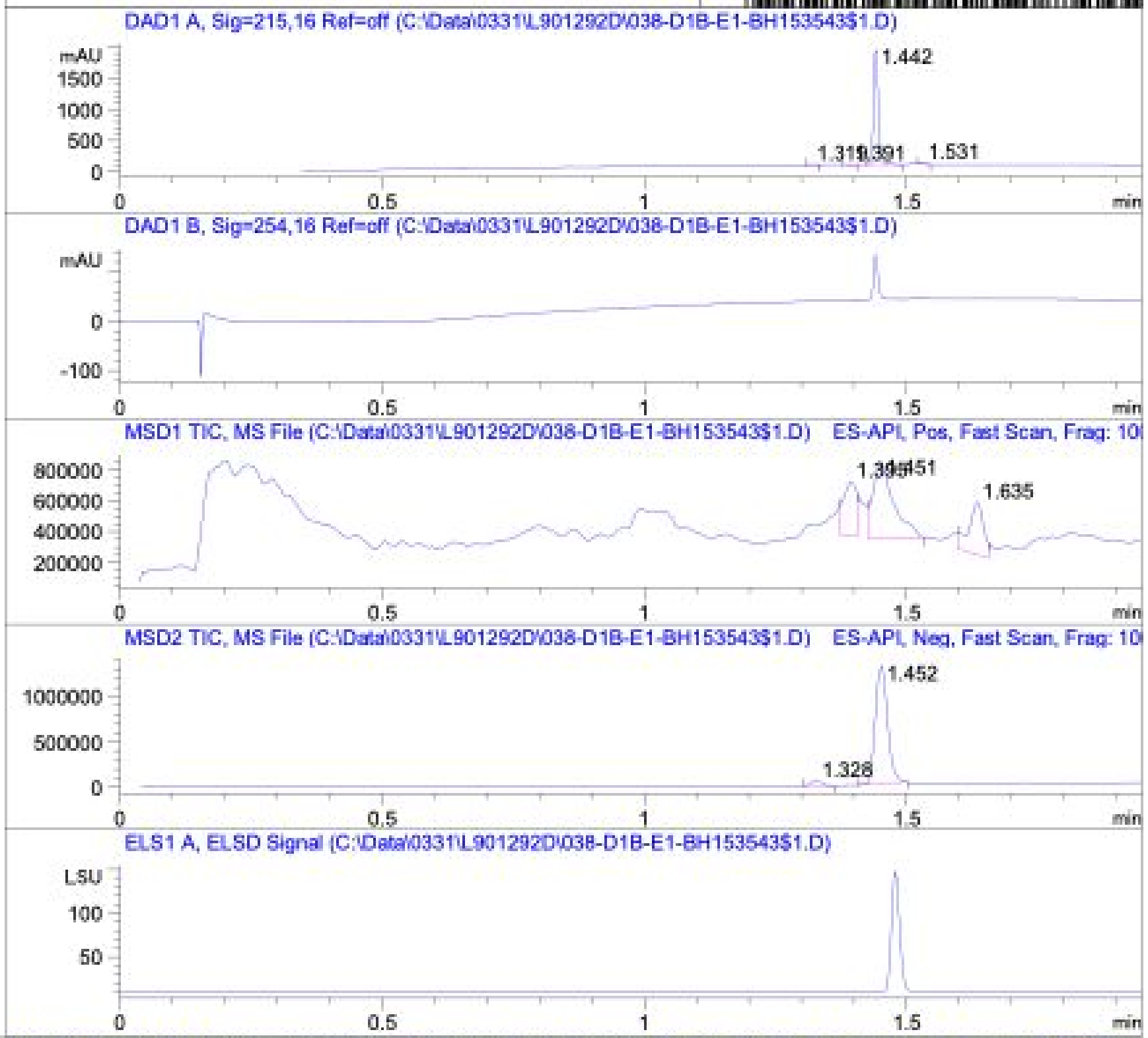

RT 1.395

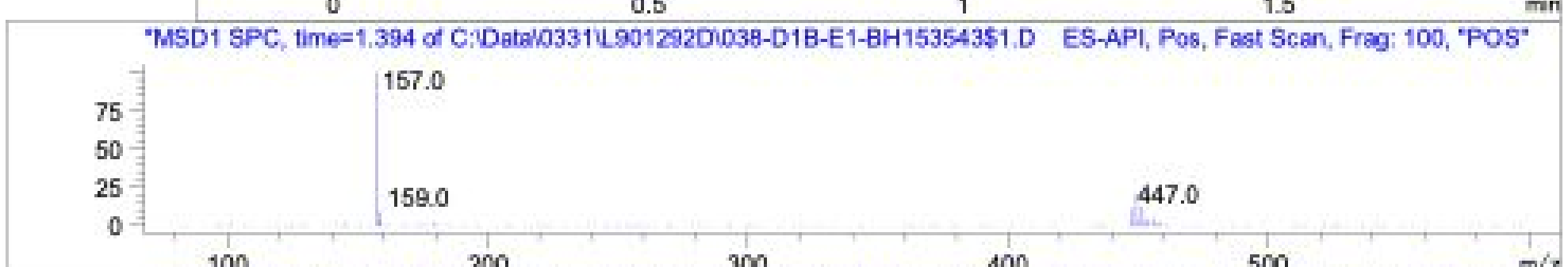

RT 1.451

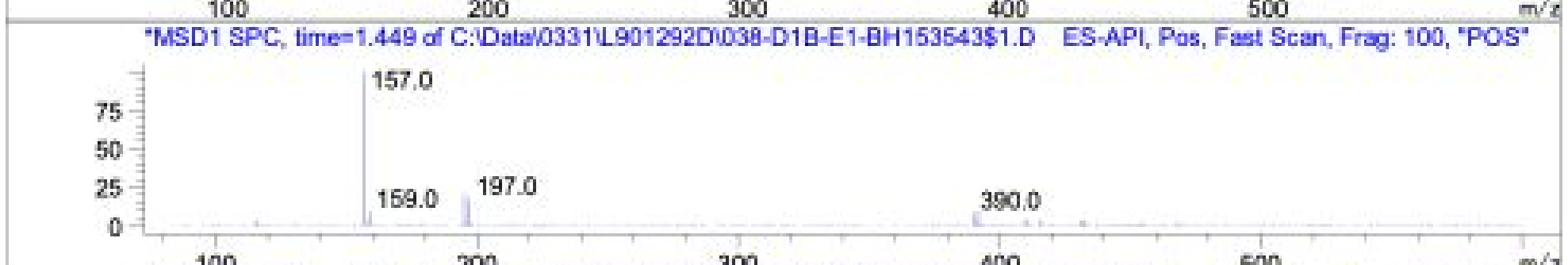

RT 1.635

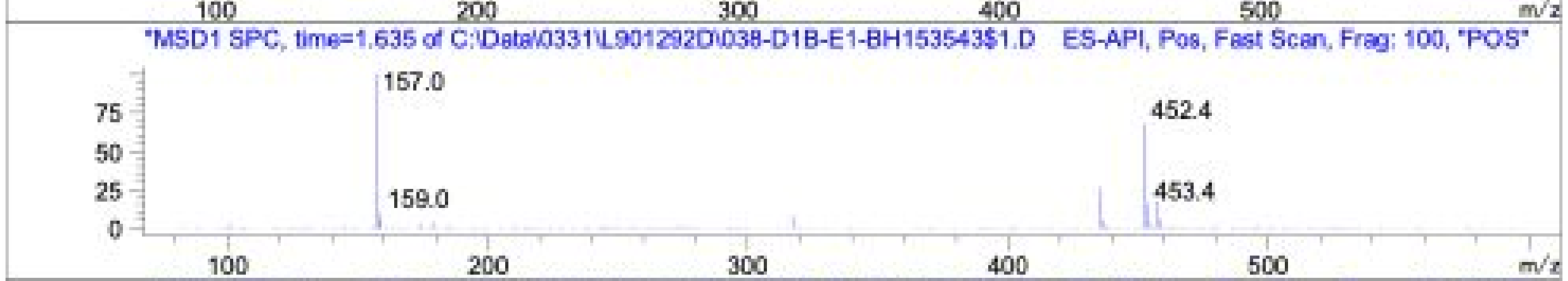

RT 1.328

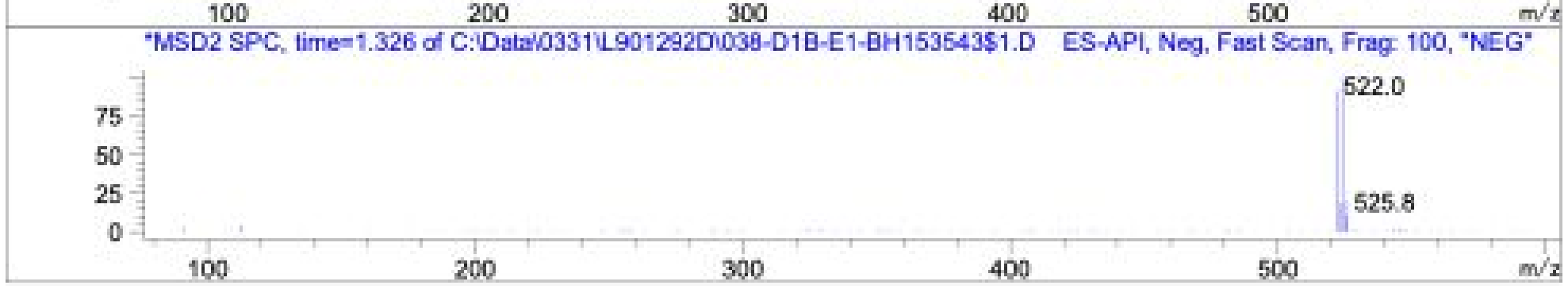

Inj.Date 3/31/2025 NEW -30-
C:\Users\Public\Documents\ChemStation\1\Data\03_31\L901292D\SUPOR_96.M

Z9544922110

MaxPeak: 96.48%
Ret_Time: 1.207 min

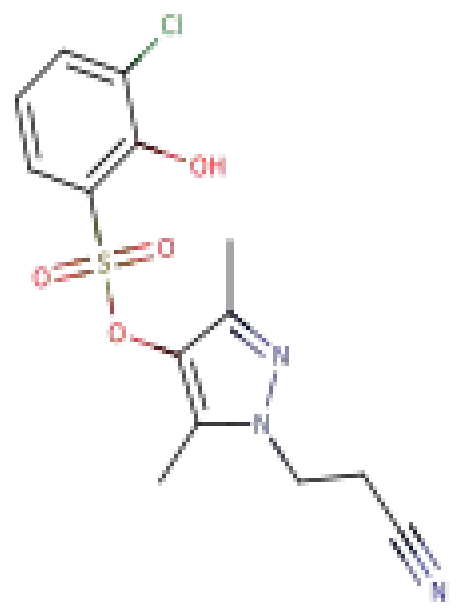

Mol Wt 355.8

Exact Mass 355.04

#	Time	Area%
1	1.207	96.48
2	1.399	1.73
3	1.601	1.79

BG917807$1

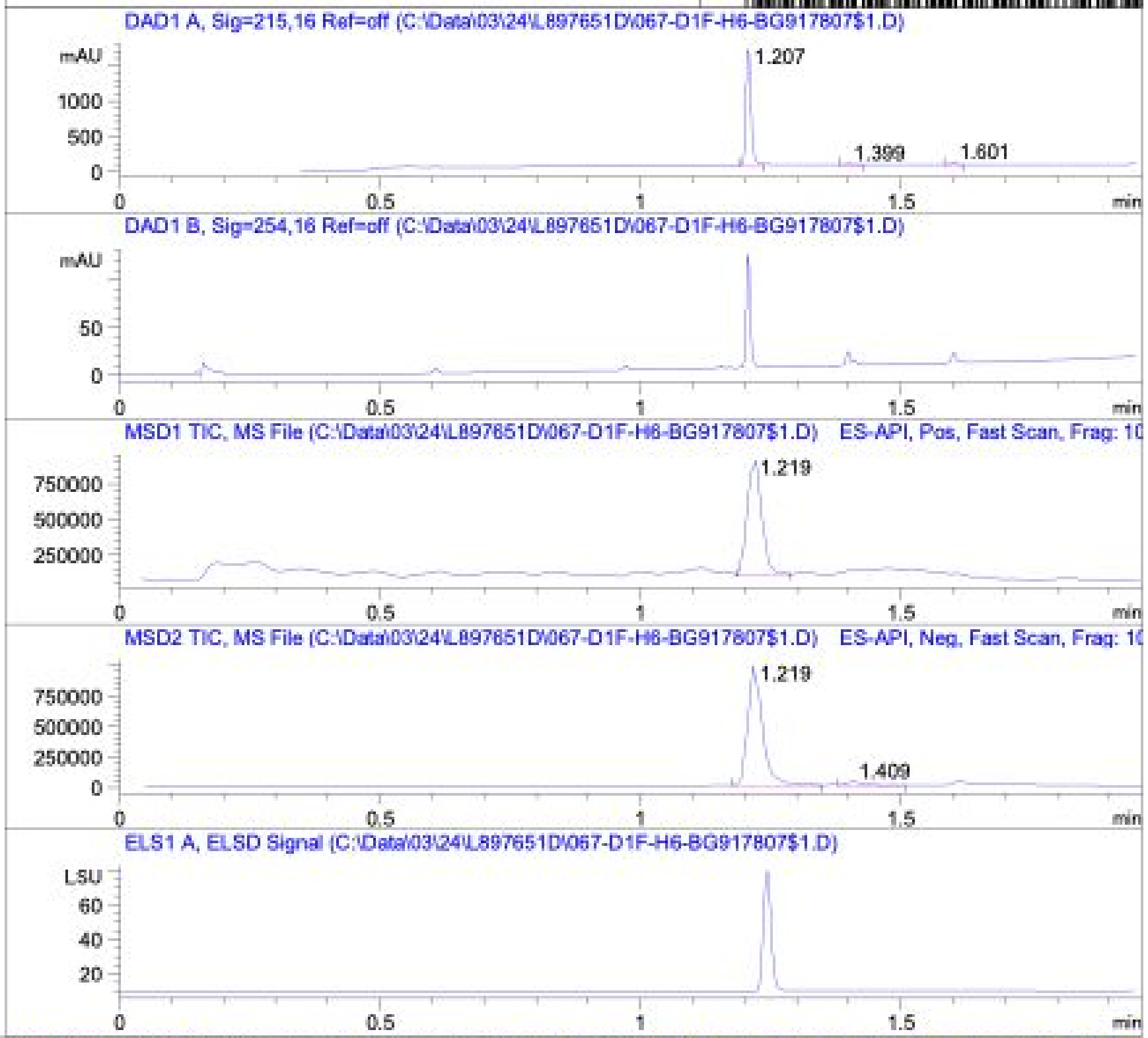

RT 1.219

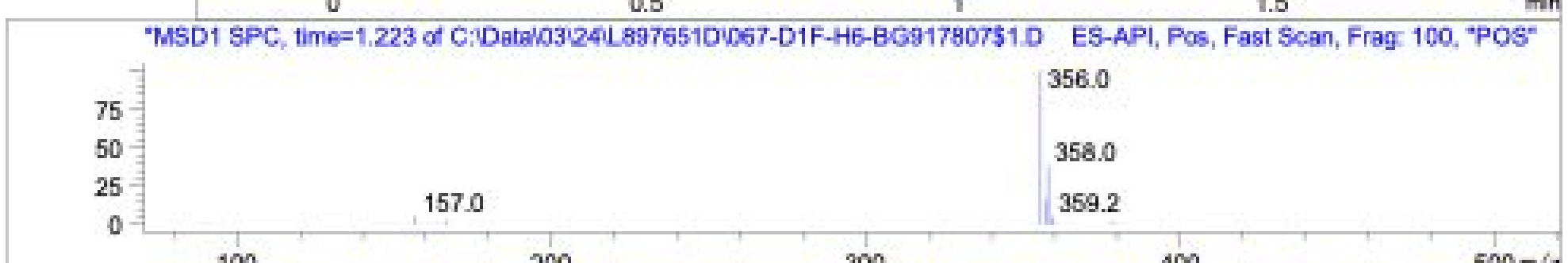

RT 1.219

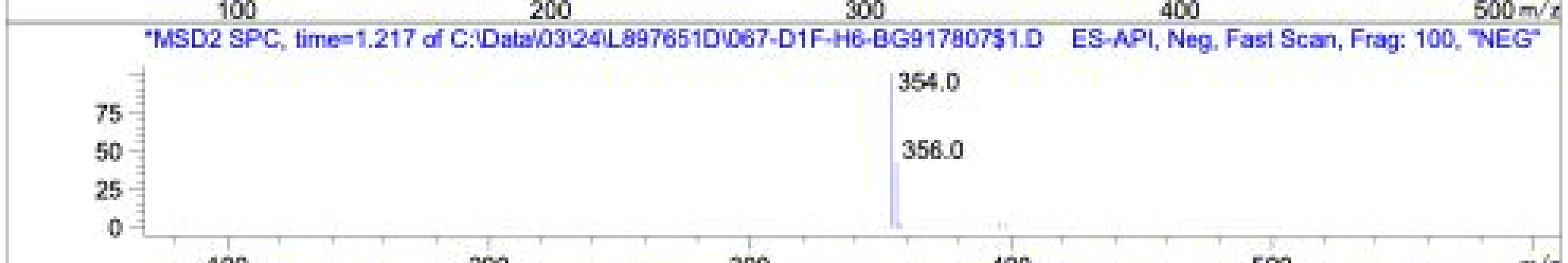

RT 1.409

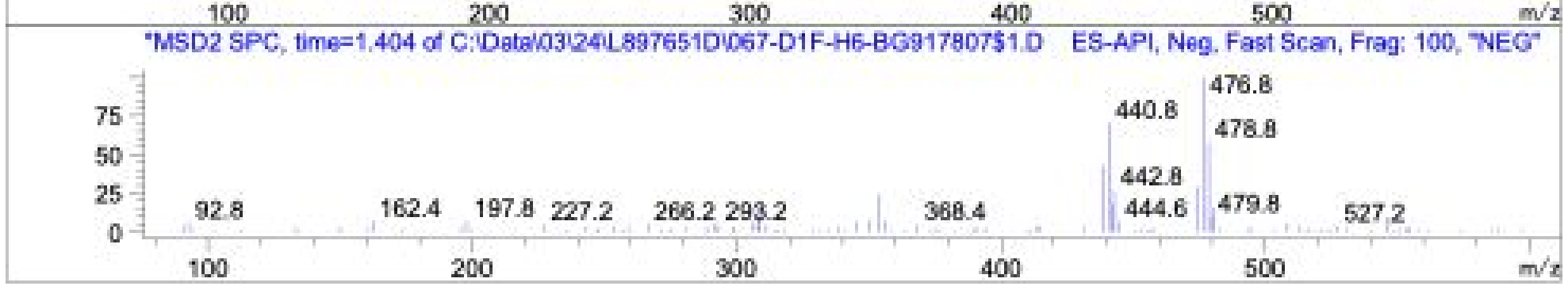

Inj.Date 3/22/2025 C -33-
C:\Users\Public\Documents\ChemStation\1\Data\03_21\L897651D\SUPOR_96.M

Z9544922113

MaxPeak: 100.00%
Ret_Time: 1.401 min

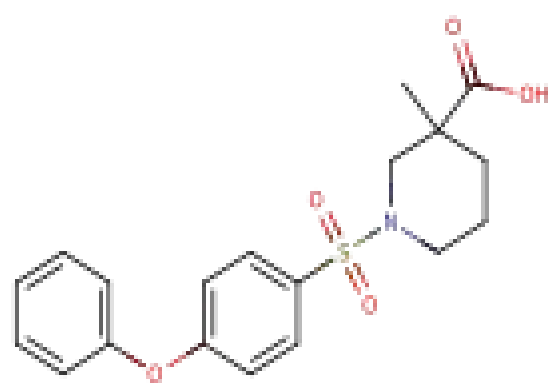

Mol Wt 375.44
Exact Mass 375.13

#	Time	Area%
1	1.401	100.00

BG917808$3

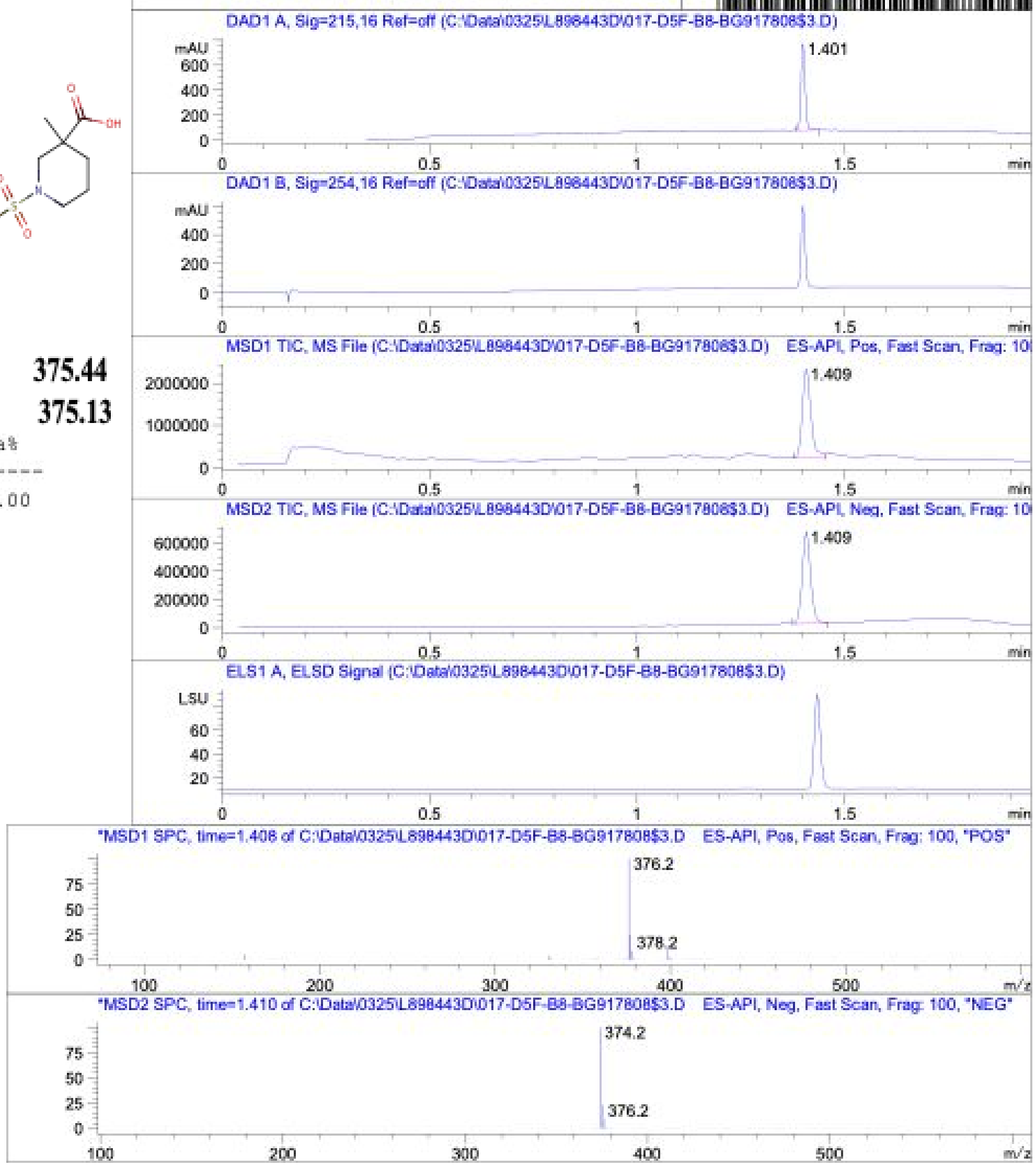

RT 1.409

RT 1.409

Inj.Date 3/25/2025 KCH -29-
C:\Users\Public\Documents\ChemStation\1\Data\03_24\L898443D\SUPOR_30.M

Z9544922116

MaxPeak: 100.00%
Ret_Time: 1.326 min

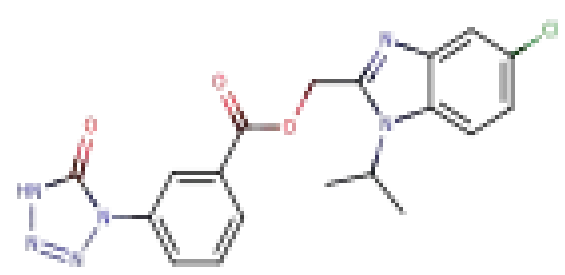

Mol Wt 412.83
Exact Mass 412.11

#	Time	Area%
1	1.326	100.00

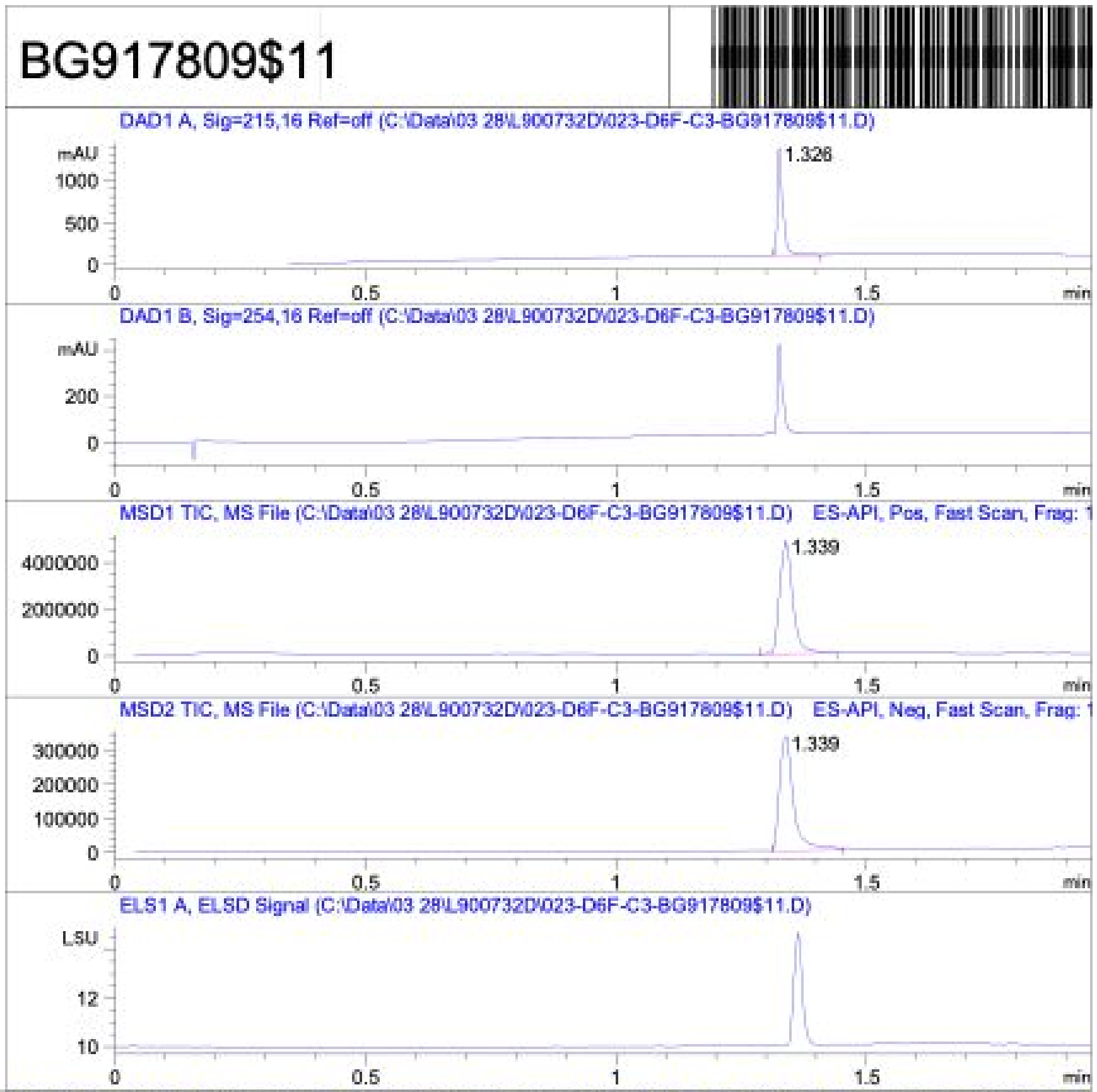

RT 1.339

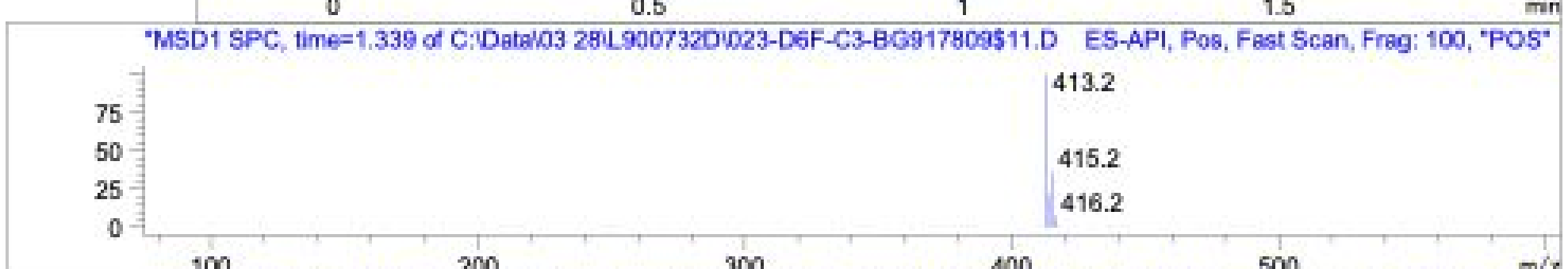

RT 1.339

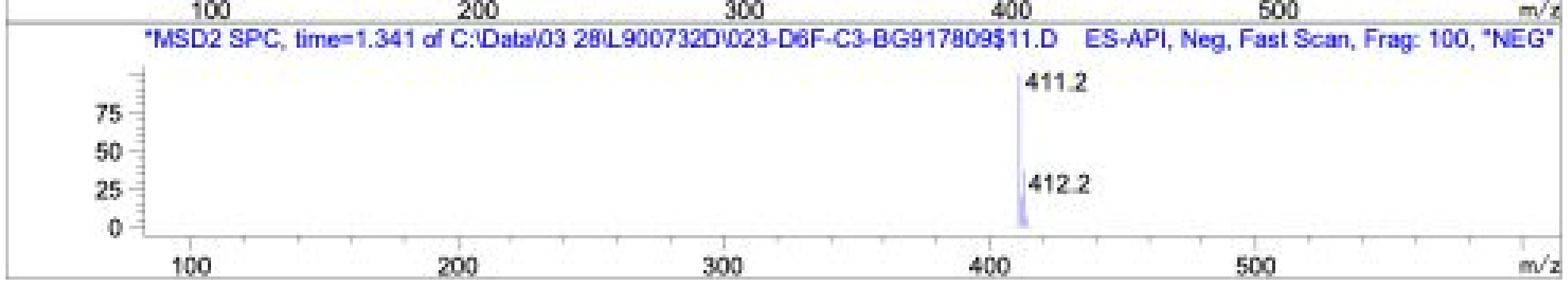

Inj.Date 3/28/2025 KCH
C:\Users\Public\Documents\ChemStation\1\Data\03_28\L900732D\SUPOR_30.M

Z9544922119

MaxPeak: 100.00%
Ret_Time: 1.360 min

BG917811$23

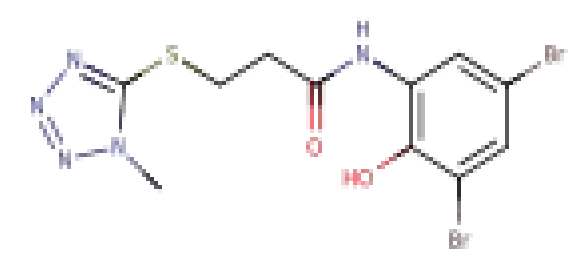

Mol Wt 437.11
Exact Mass 436.9

#	Time	Area%
1	1.360	100.00

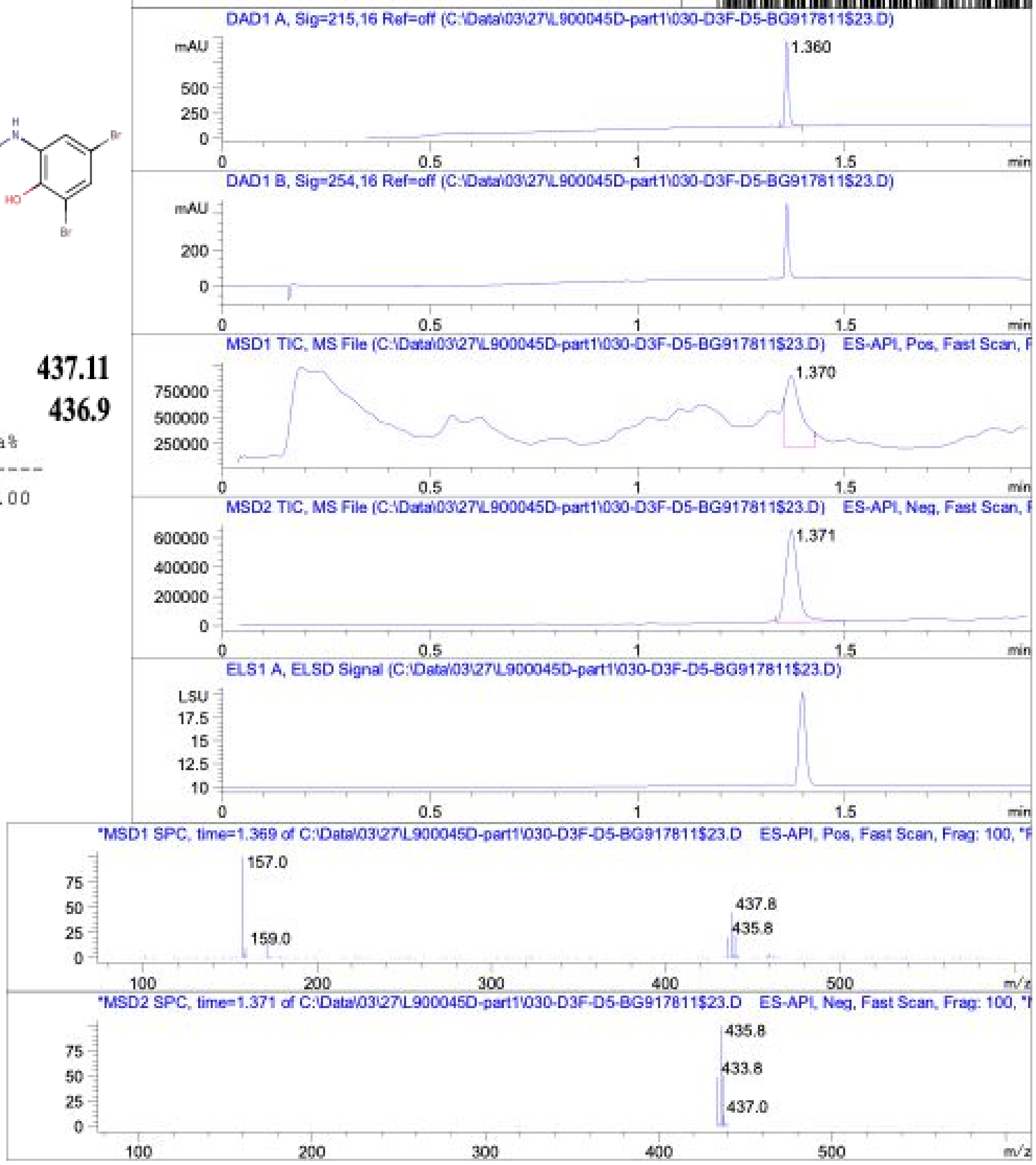

RT 1.370

RT 1.371

Inj.Date 3/27/2025 C -30-
C:\Users\Public\Documents\ChemStation\1\Data\03_27\1900045D\SUPOR_30.M

Z9544922126

MaxPeak: 100.00%
Ret_Time: 0.938 min

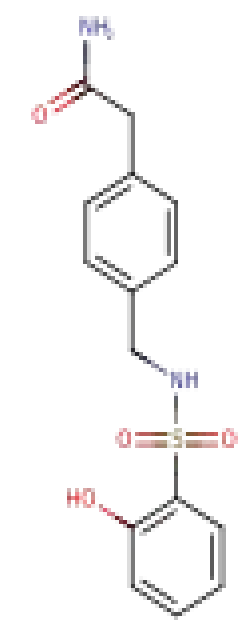

Mol Wt 320.36

Exact Mass 320.09

#	Time	Area%
1	0.938	100.00

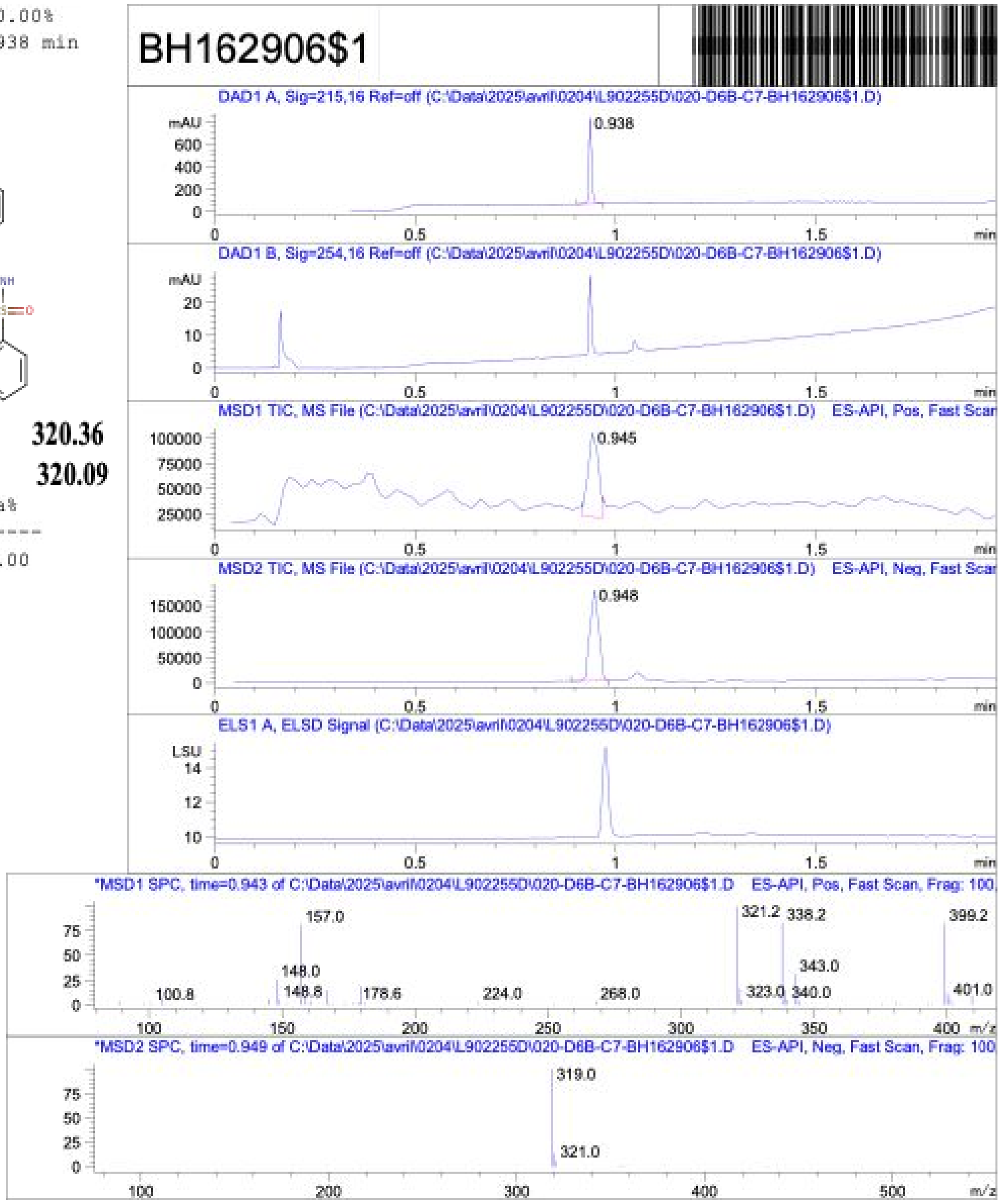

RT 0.945

RT 0.948

Inj.Date 4/2/2025 NEW
C:\Users\Public\Documents\ChemStation\1\Data\04_01\L902255D\SUPOR_30.M

Z9544922135

MaxPeak: 100.00%
Ret_Time: 1.026 min

BG917813$5

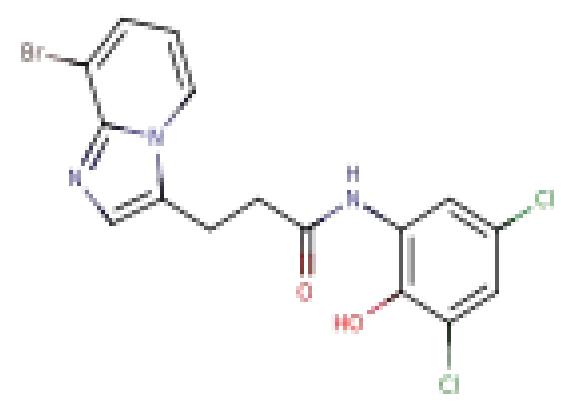

Mol Wt 429.1
Exact Mass 428.96

#	Time	Area%
1	1.026	100.00

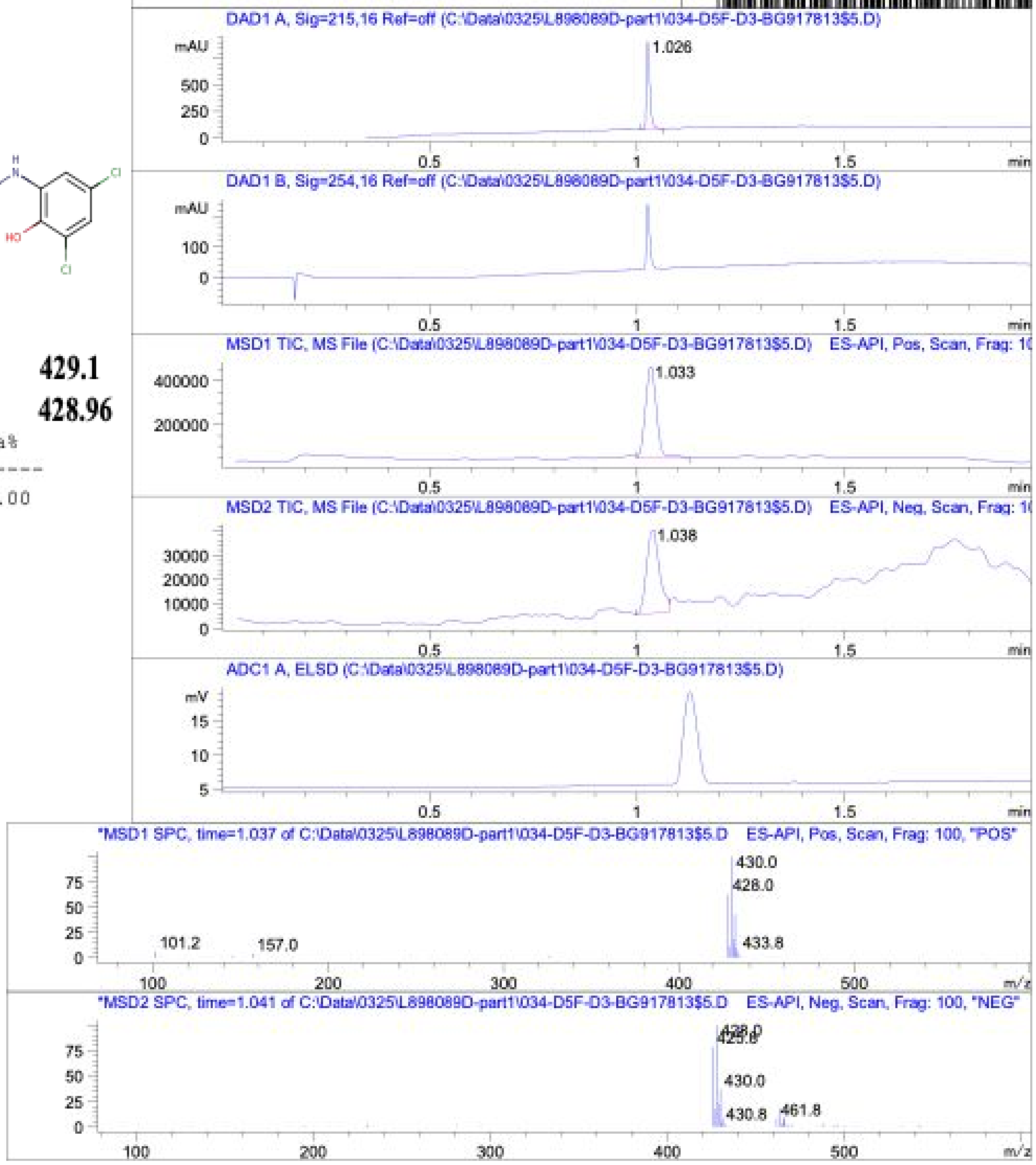

RT 1.033

RT 1.038

Inj.Date 3/24/2025 NEW -6-
C:\Users\Public\Documents\ChemStation\1\Data\03_24\1898089D\SUPOR_30.M

Z9544922144

MaxPeak: 96.35%
Ret_Time: 0.974 min

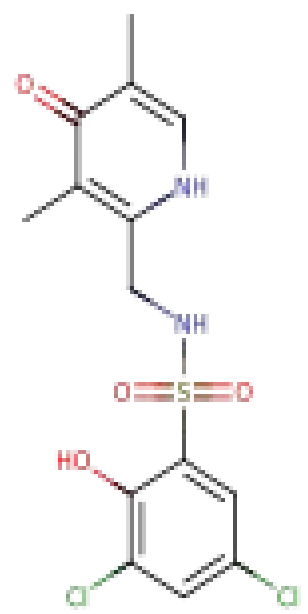

Mol Wt 377.24

Exact Mass 376.01

#	Time	Area%
1	0.974	96.35
2	1.234	3.65

T9072724

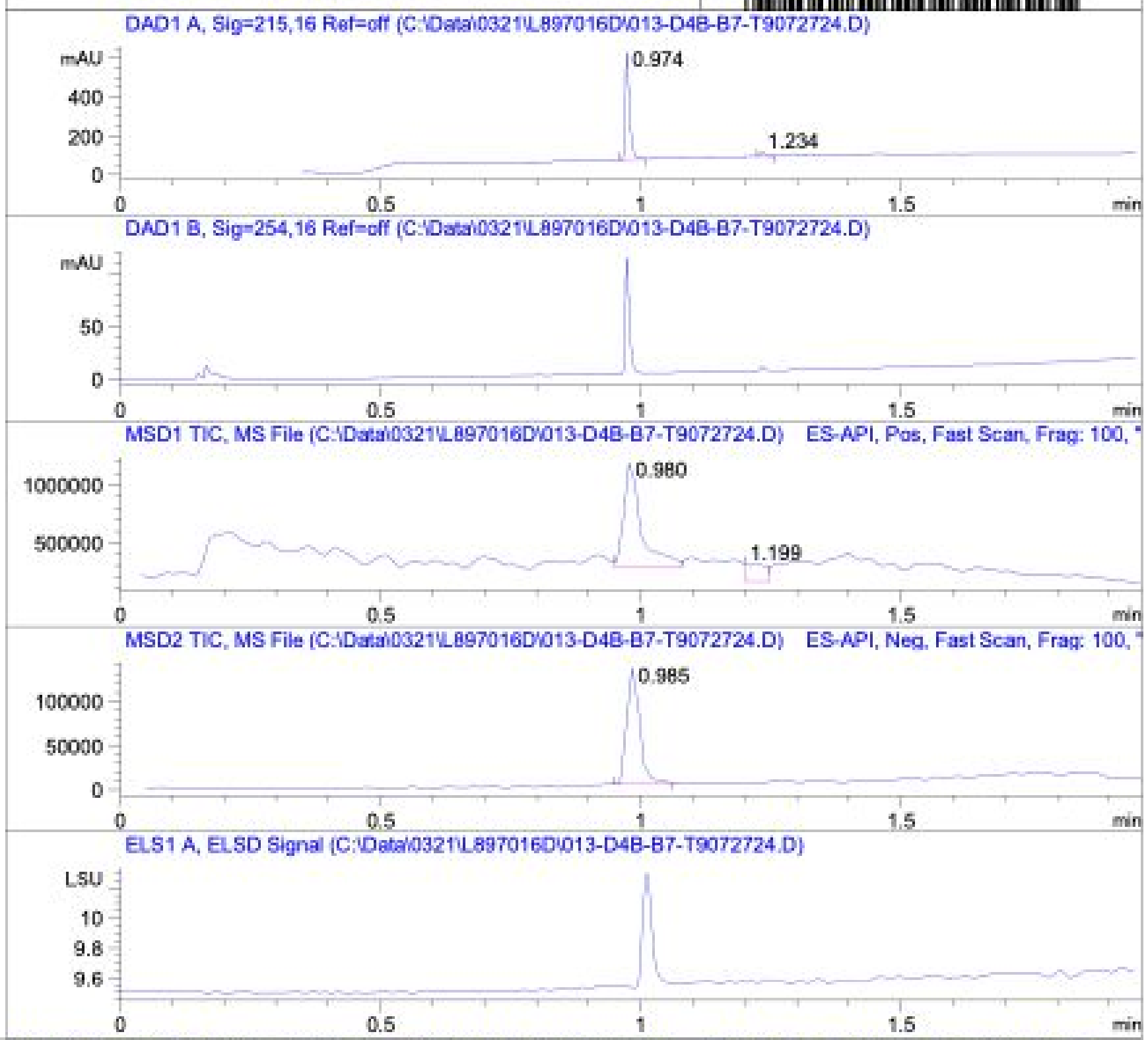

RT 0.980

RT 1.199

RT 0.985

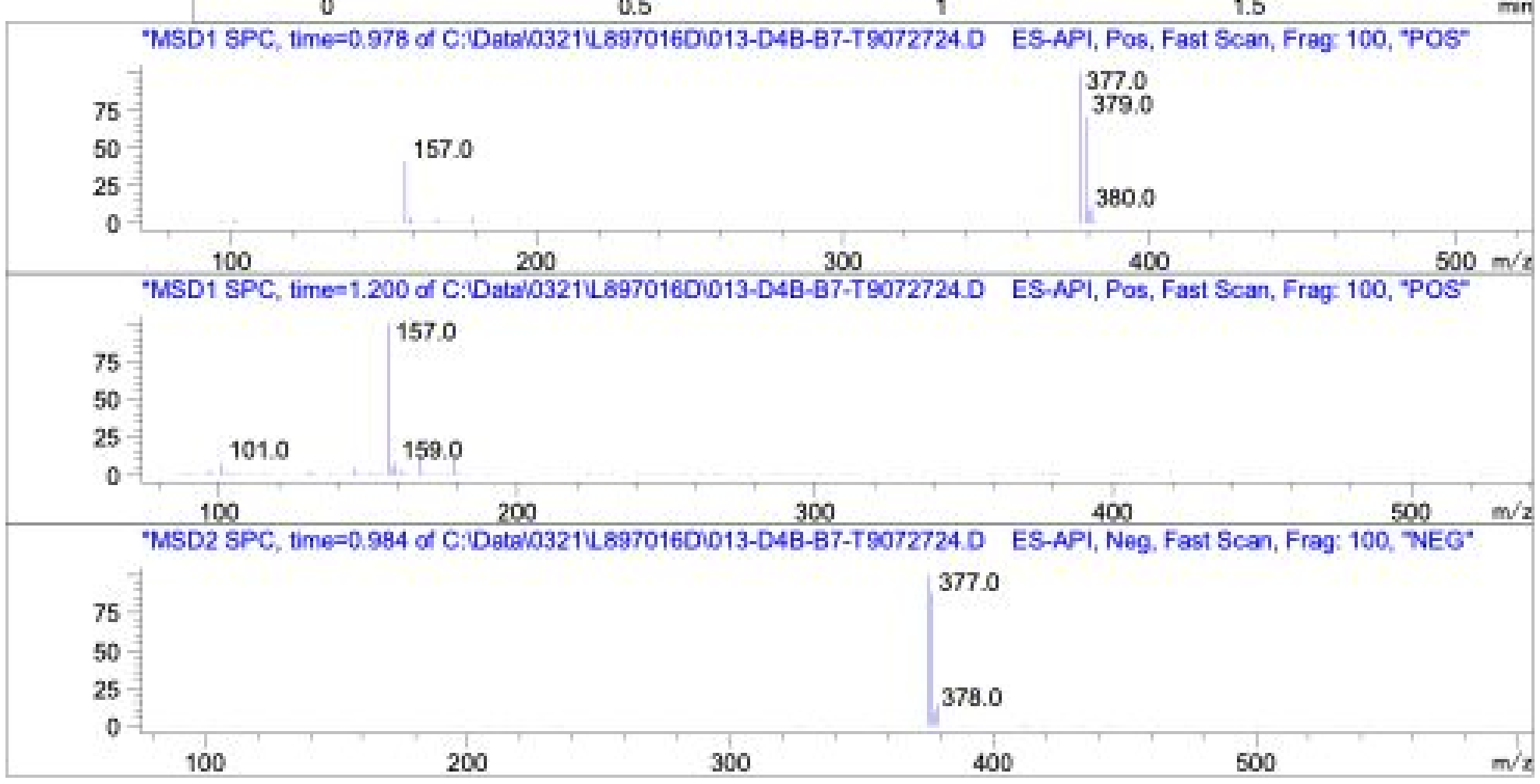

Inj.Date 3/20/2025 NEW
C:\Users\Public\Documents\ChemStation\1\Data\03_20\L897016D\SUPOR_30.M

Z9544922186

MaxPeak: 100.00%
Ret_Time: 1.480 min

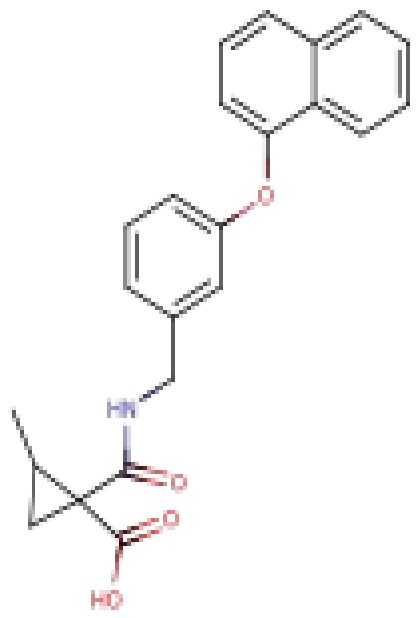

Mol Wt 375.42
Exact Mass 375.17

#	Time	Area%
1	1.480	100.00

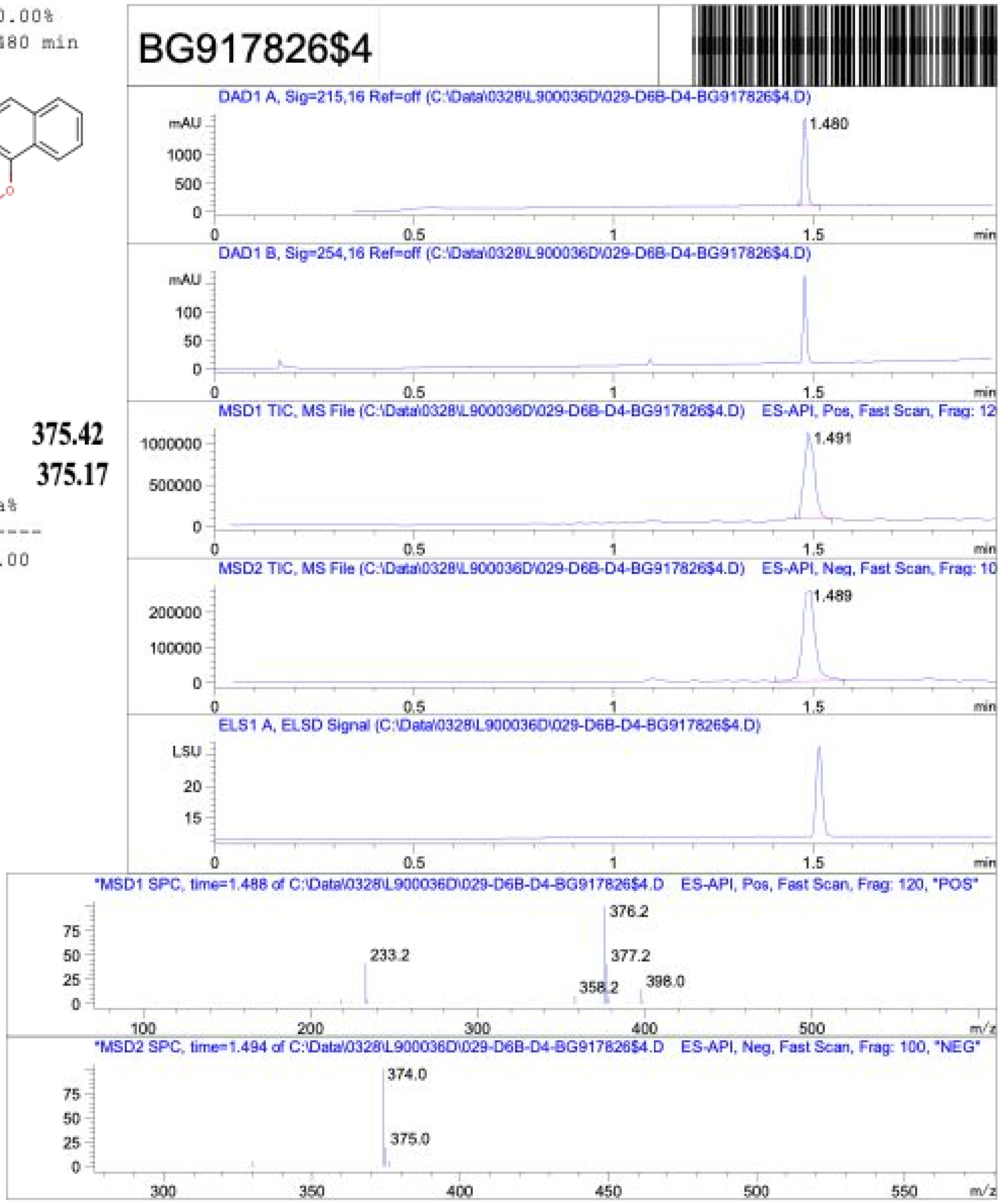

RT 1.491

RT 1.489

Inj.Date 3/27/2025 NEW -2-
C:\Users\Public\Documents\ChemStation\1\Data\03_27\L900036D\SUPOR_30.M

Z9544922231

MaxPeak: 100.00%
Ret_Time: 1.409 min

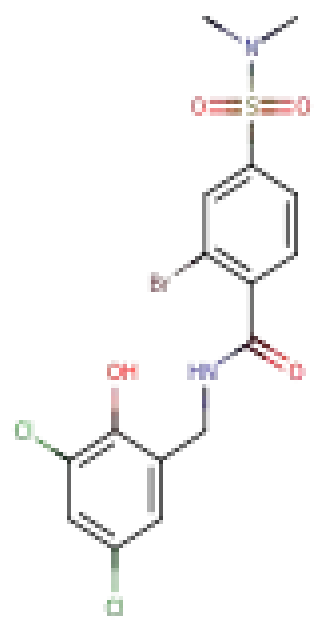

Mol Wt 482.18

Exact Mass 481.94

#	Time	Area%
1	1.409	100.00

BG917830$3

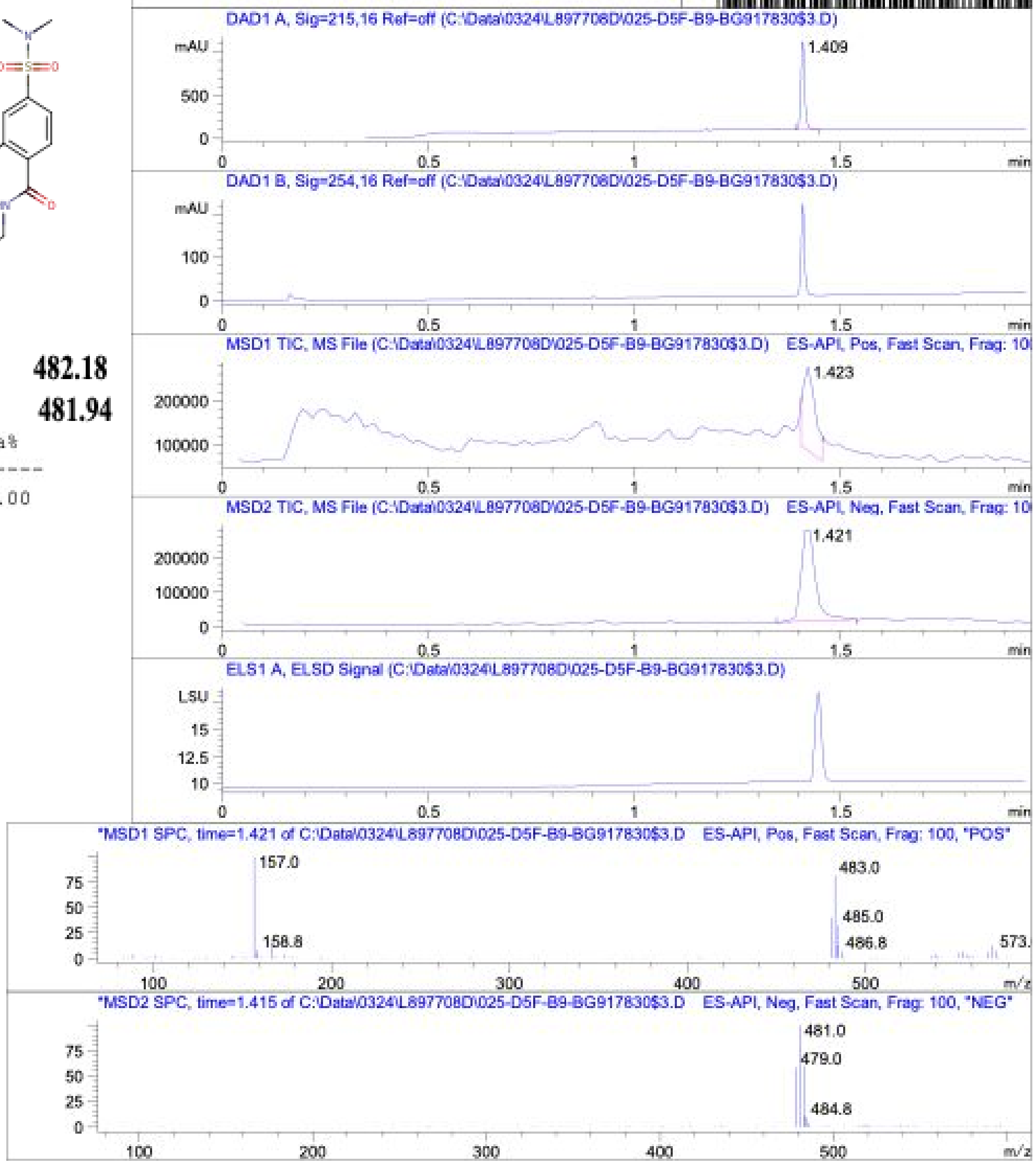

Inj.Date 3/21/2025 NEW -33-
C:\Users\Public\Documents\ChemStation\1\Data\03_21\L897708D\SUPOR_30.M

Z9544922262

MaxPeak: 100.00%
Ret_Time: 1.367 min

BG917835$2

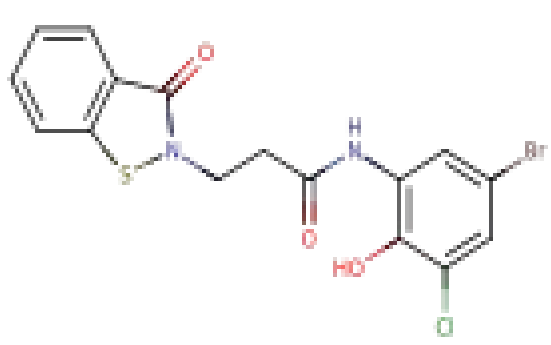

Mol Wt 427.7

Exact Mass 427.95

#	Time	Area%
1	1.367	100.00

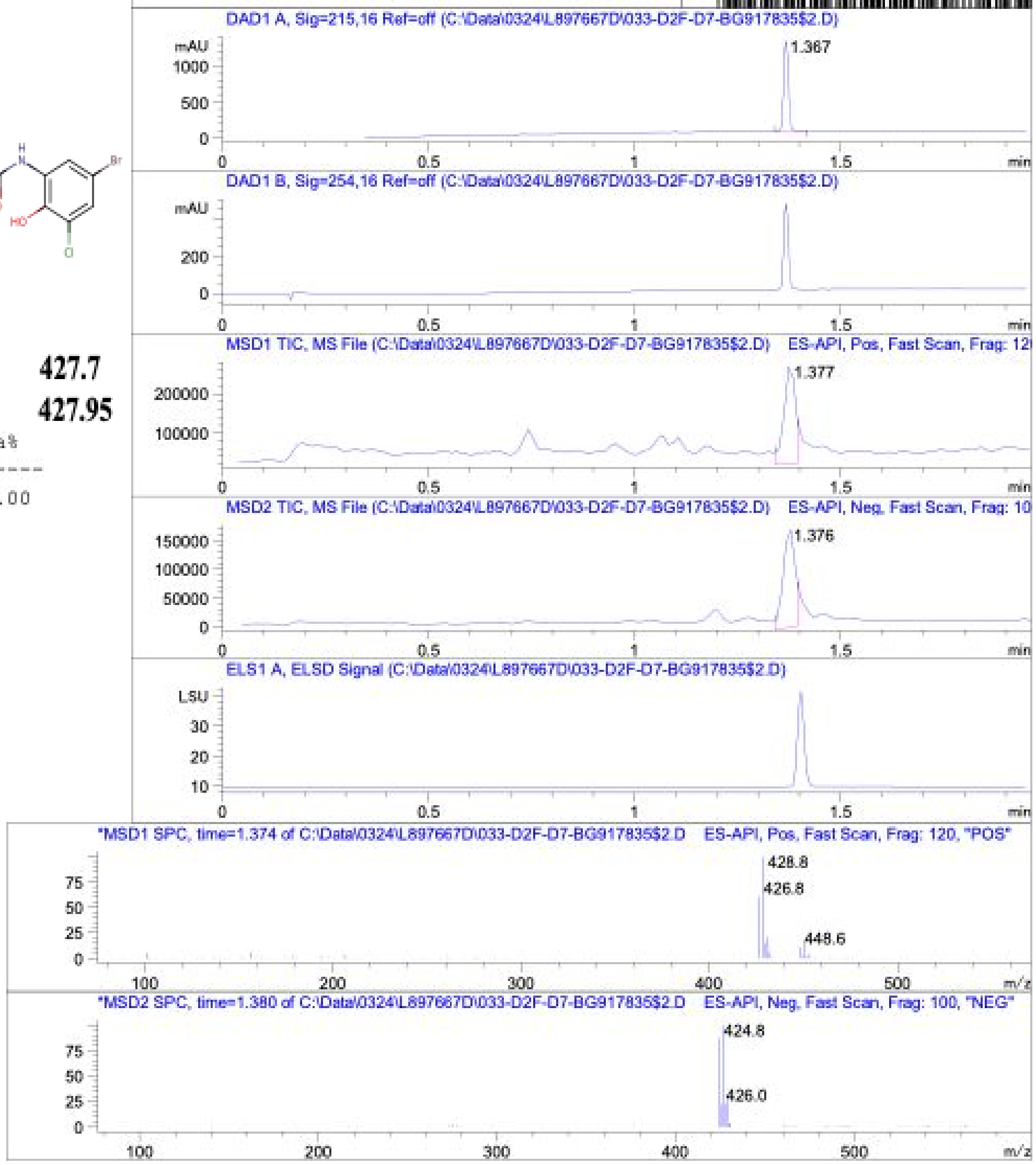

RT 1.377

RT 1.376

Inj.Date 3/22/2025 NEW -21-
C:\Users\Public\Documents\ChemStation\1\Data\03_21\L897667D\SUPOR_30.M

Z9544922281

MaxPeak: 100.00%
Ret_Time: 1.490 min

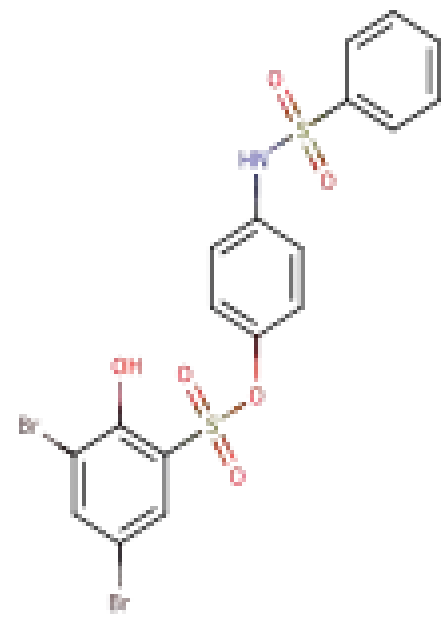

Mol Wt 563.24

Exact Mass 562.85

#	Time	Area%
1	1.490	100.00

BG917837$25

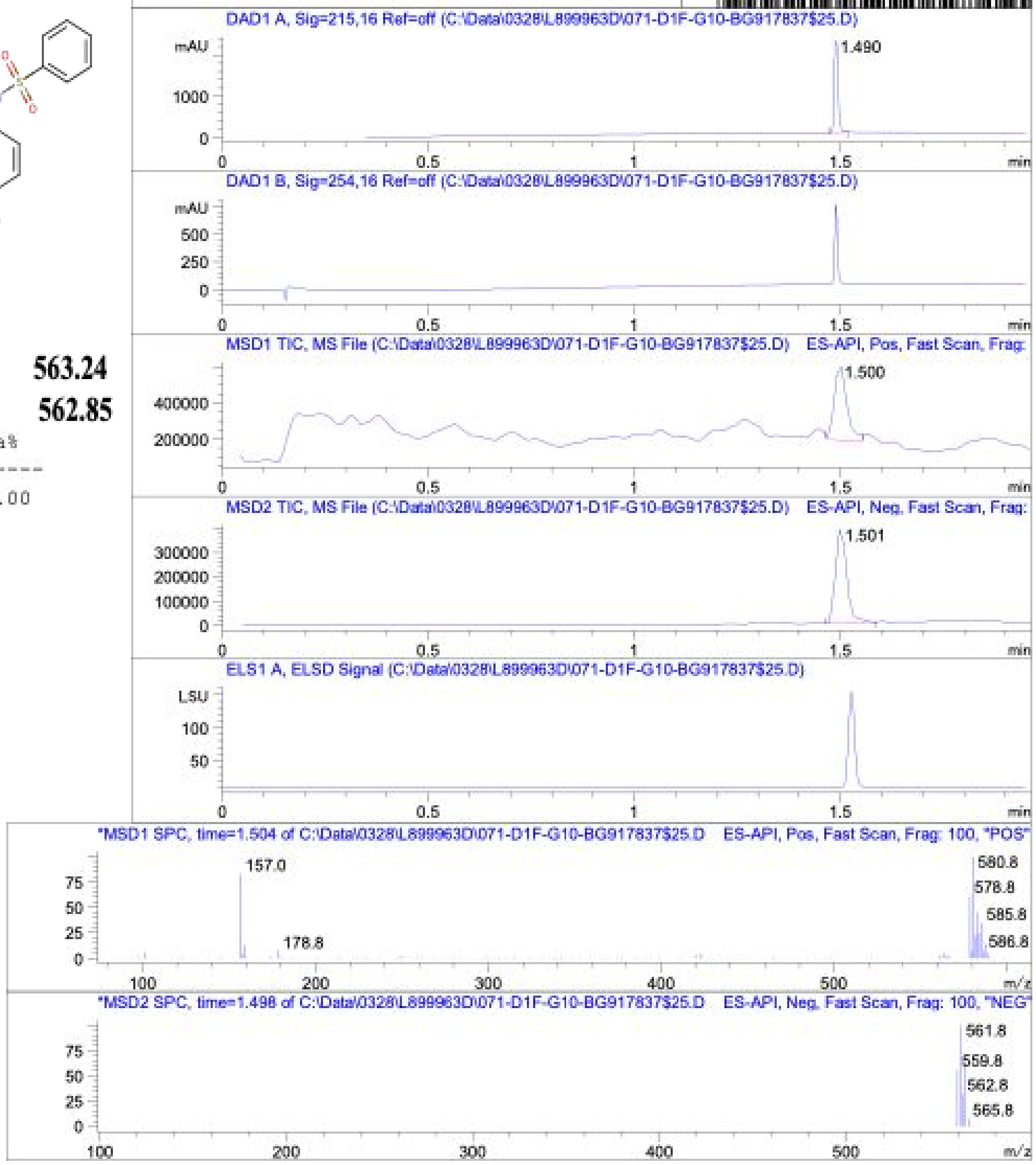

RT 1.500

RT 1.501

Inj.Date 3/27/2025 NEW

C:\Users\Public\Documents\ChemStation\1\Data\03_27\L899963D\SUPOR_96.M

Z9544922287

MaxPeak: 100.00%
Ret_Time: 1.292 min

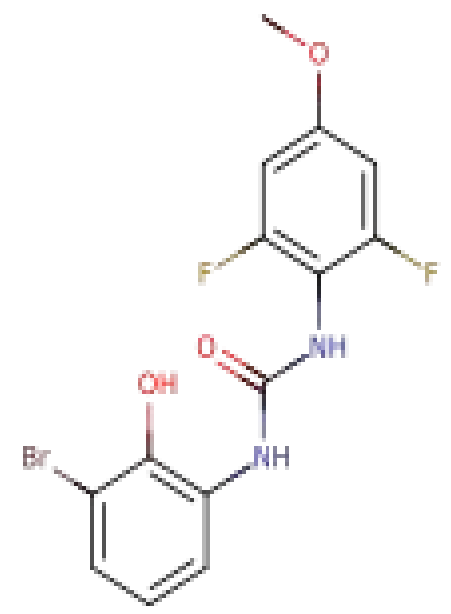

Mol Wt 373.15

Exact Mass 372

#	Time	Area%
1	1.292	100.00

BG917838$2

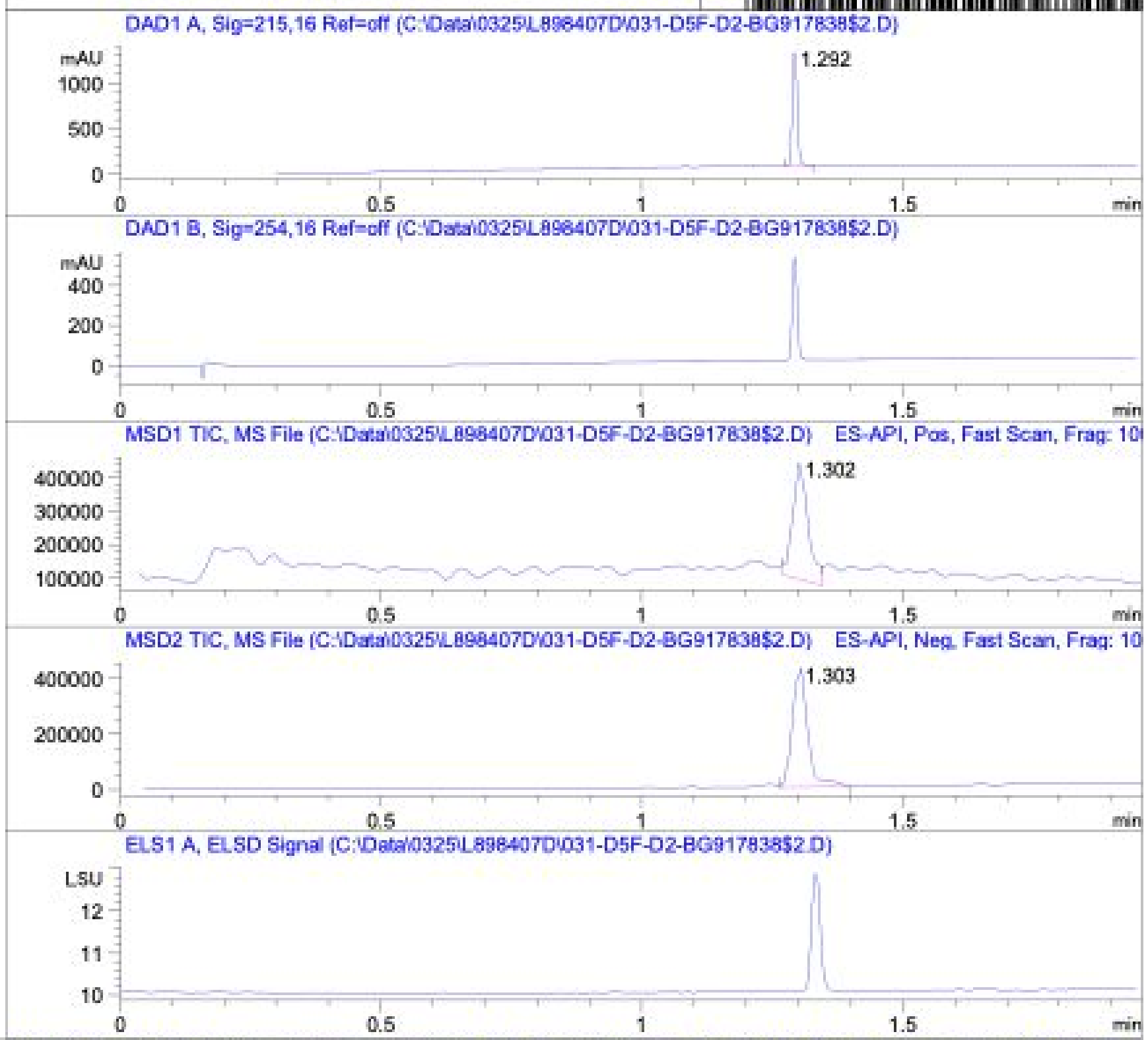

RT 1.302

RT 1.303

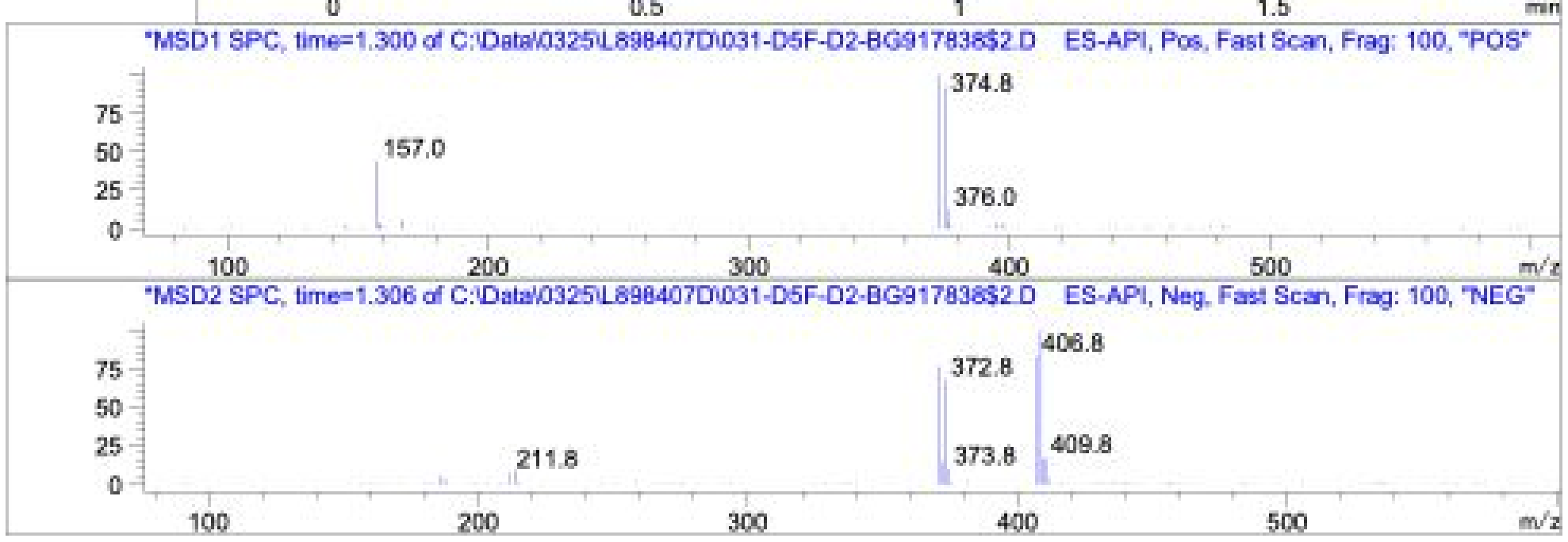

Inj.Date 3/24/2025 NEW
C:\Users\Public\Documents\ChemStation\1\Data\03_24\L898407D\SUPOR_30.M

Z9544922290

MaxPeak: 100.00%
Ret_Time: 1.072 min

BG917840$1

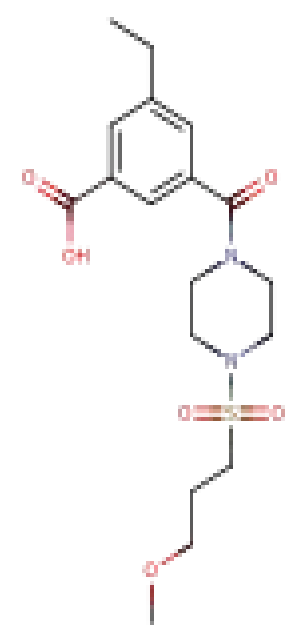

Mol Wt 398.48

Exact Mass 398.17

#	Time	Area%
1	1.072	100.00

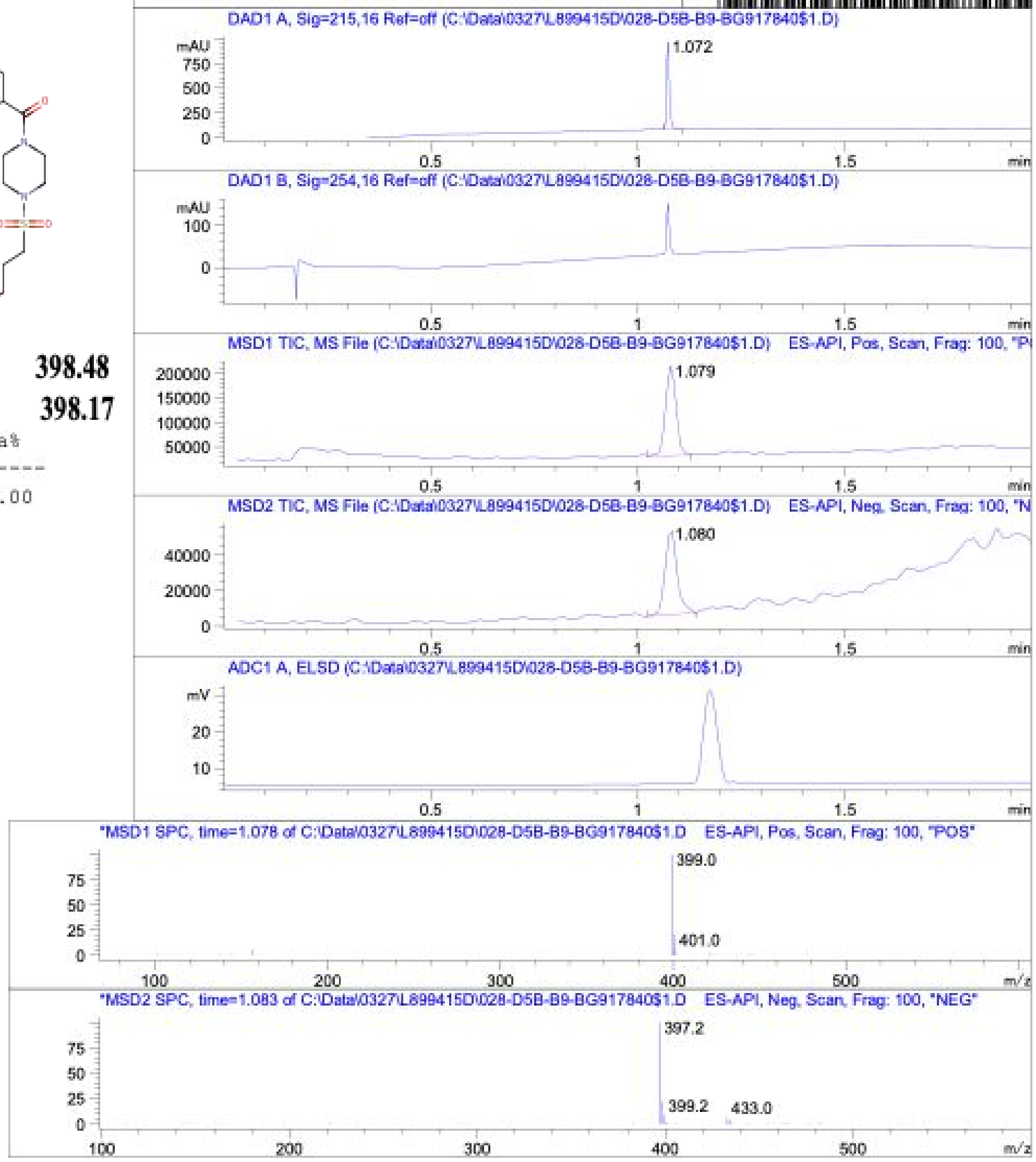

RT 1.079

RT 1.080

Inj.Date 3/26/2025 NEW -6-
C:\Users\Public\Documents\ChemStation\1\Data\03_26\L899415D\SUPOR_30.M

Z9544922296

MaxPeak: 100.00%
Ret_Time: 1.239 min

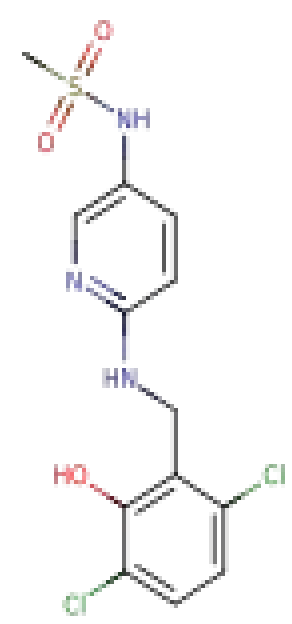

Mol Wt 362.23

Exact Mass 361.01

#	Time	Area%
1	1.239	100.00

BG917845$2

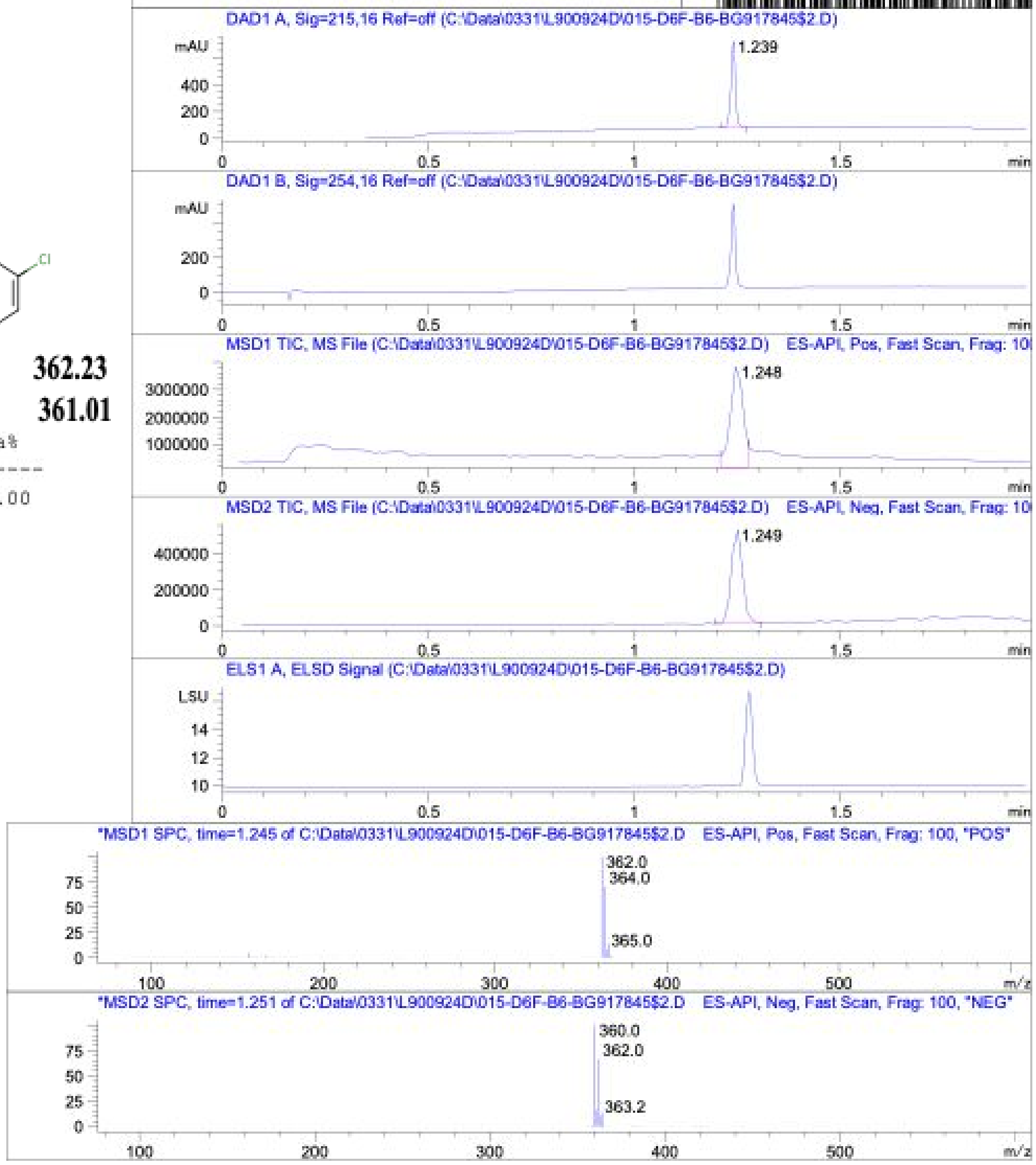

RT 1.248

RT 1.249

Inj.Date 3/28/2025 KCH
C:\Users\Public\Documents\ChemStation\1\Data\03_28\L900924D\SUPOR_30.M

Z9544922311

MaxPeak: 100.00%
Ret_Time: 1.324 min

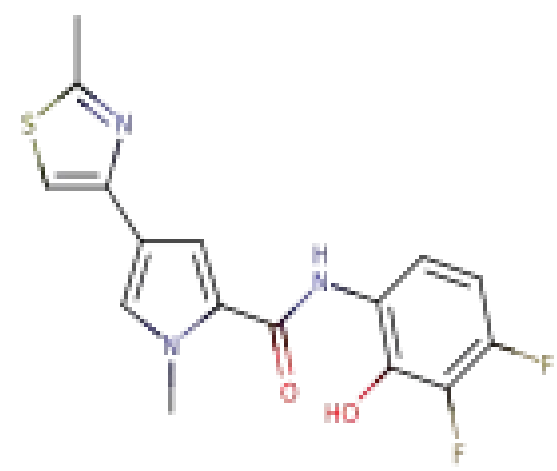

Mol Wt 349.36

Exact Mass 349.08

#	Time	Area%
1	1.324	100.00

BG917849$41

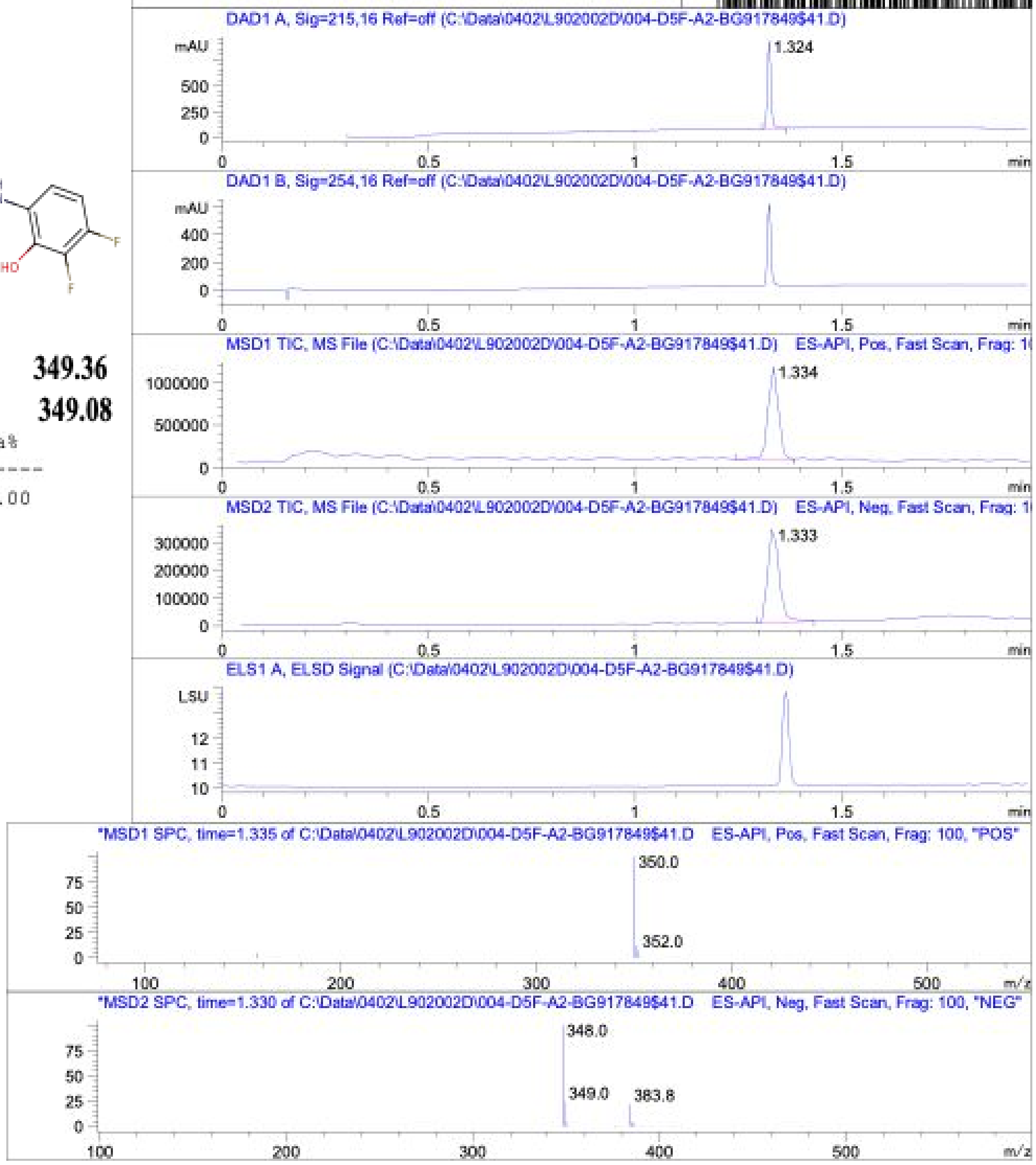

Inj.Date 4/1/2025 NEW
C:\Users\Public\Documents\ChemStation\1\Data\04_01\L902002D\SUPOR_30.M

Z9544922324

MaxPeak: 100.00%
Ret_Time: 1.355 min

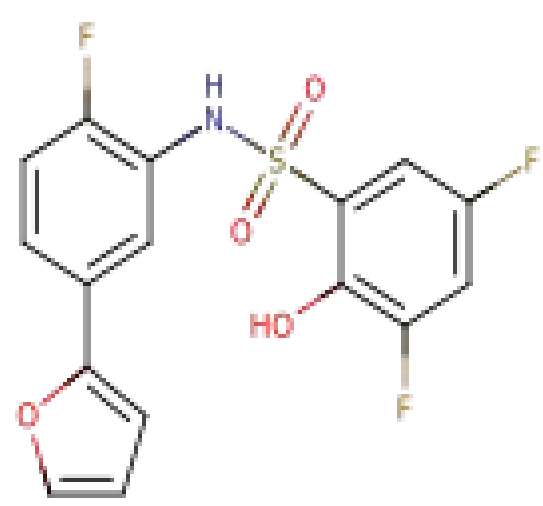

Mol Wt 369.32
Exact Mass 369.03

#	Time	Area%
1	1.355	100.00

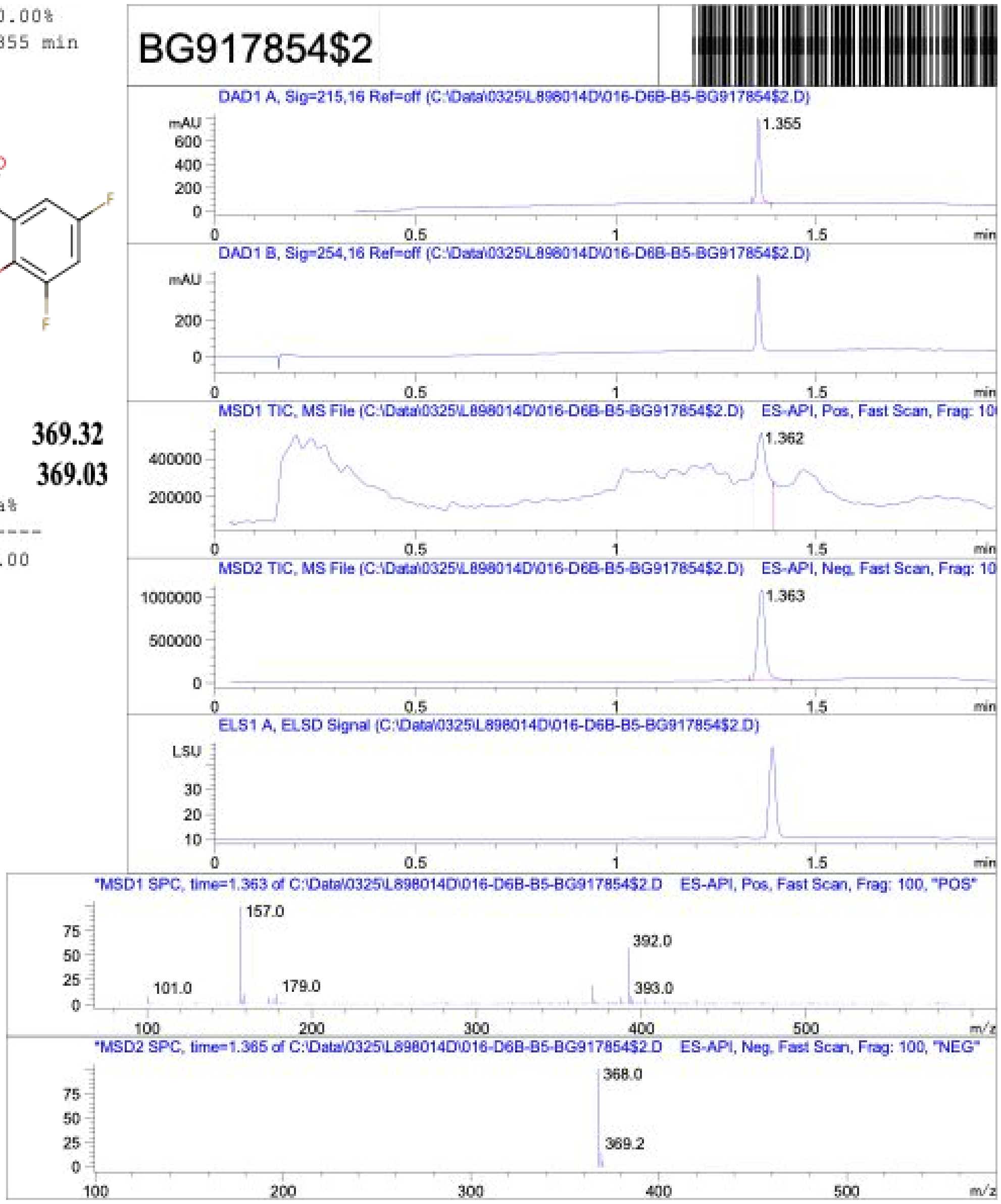

RT 1.362

RT 1.363

Inj.Date 3/24/2025 KCH -29-
C:\Users\Public\Documents\ChemStation\1\Data\03_24\L898014D\SUPOR_30.M

Z9544922335

MaxPeak: 100.00%
Ret_Time: 1.364 min

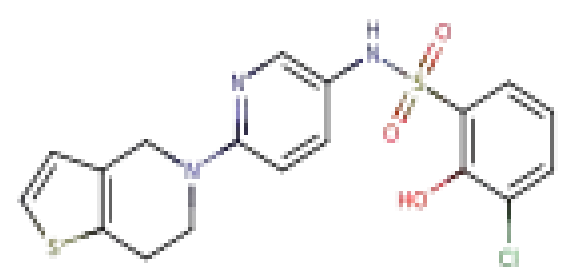

Mol Wt 421.92
Exact Mass 421.04

#	Time	Area%
1	1.364	100.00

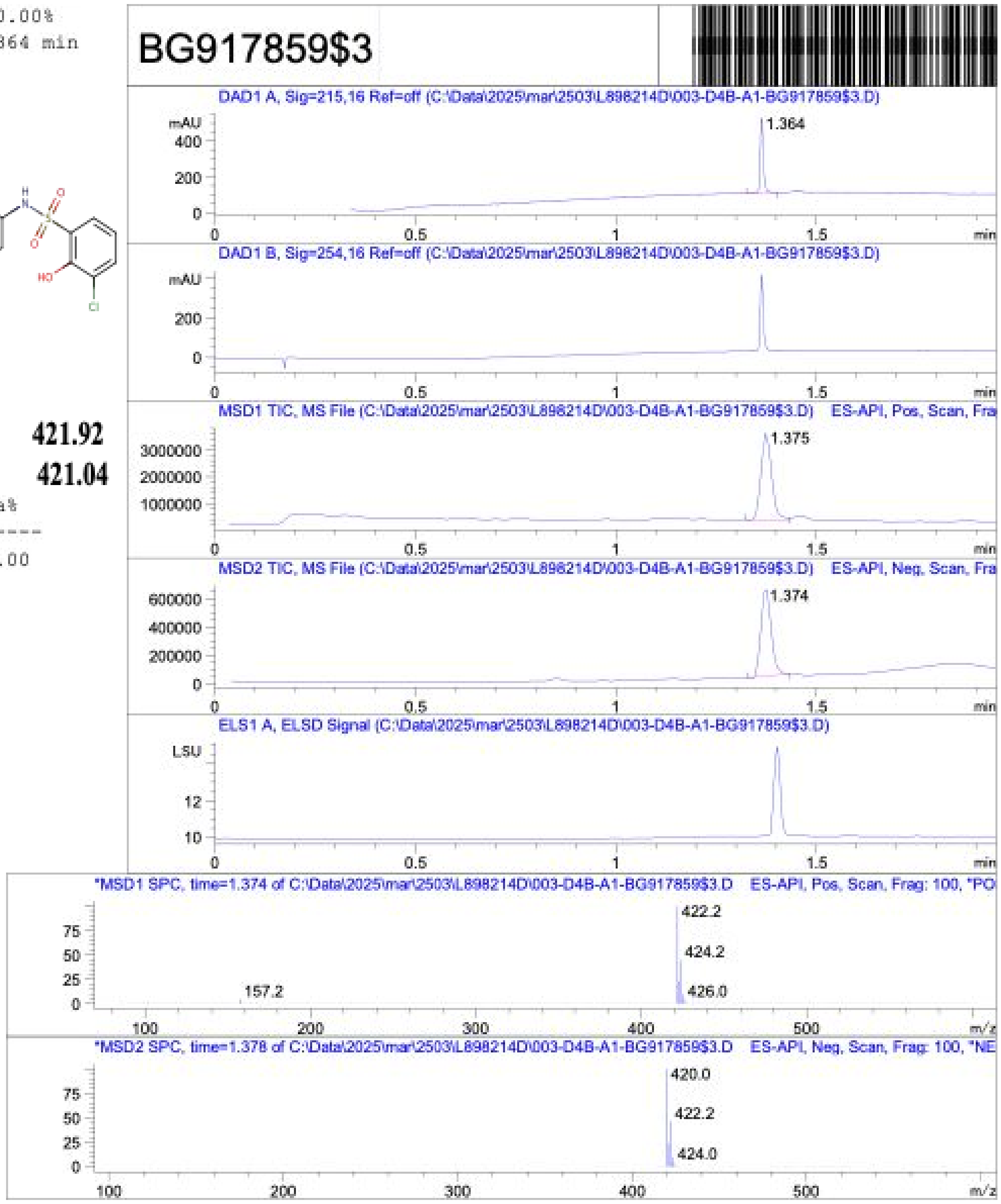

Inj.Date 3/25/2025 NEW -5-
C:\Users\Public\Documents\ChemStation\1\Data\03_24\L898214D\SUPOR_30.M

Z9544922358

MaxPeak: 100.00%
Ret_Time: 1.482 min

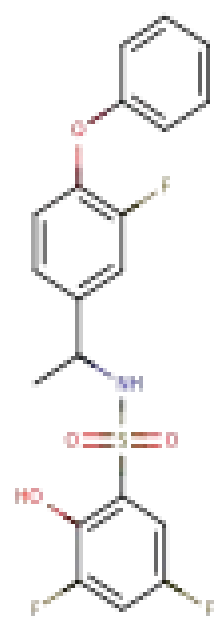

Mol Wt 423.41
Exact Mass 423.09

#	Time	Area%
1	1.482	100.00

BG917862$1

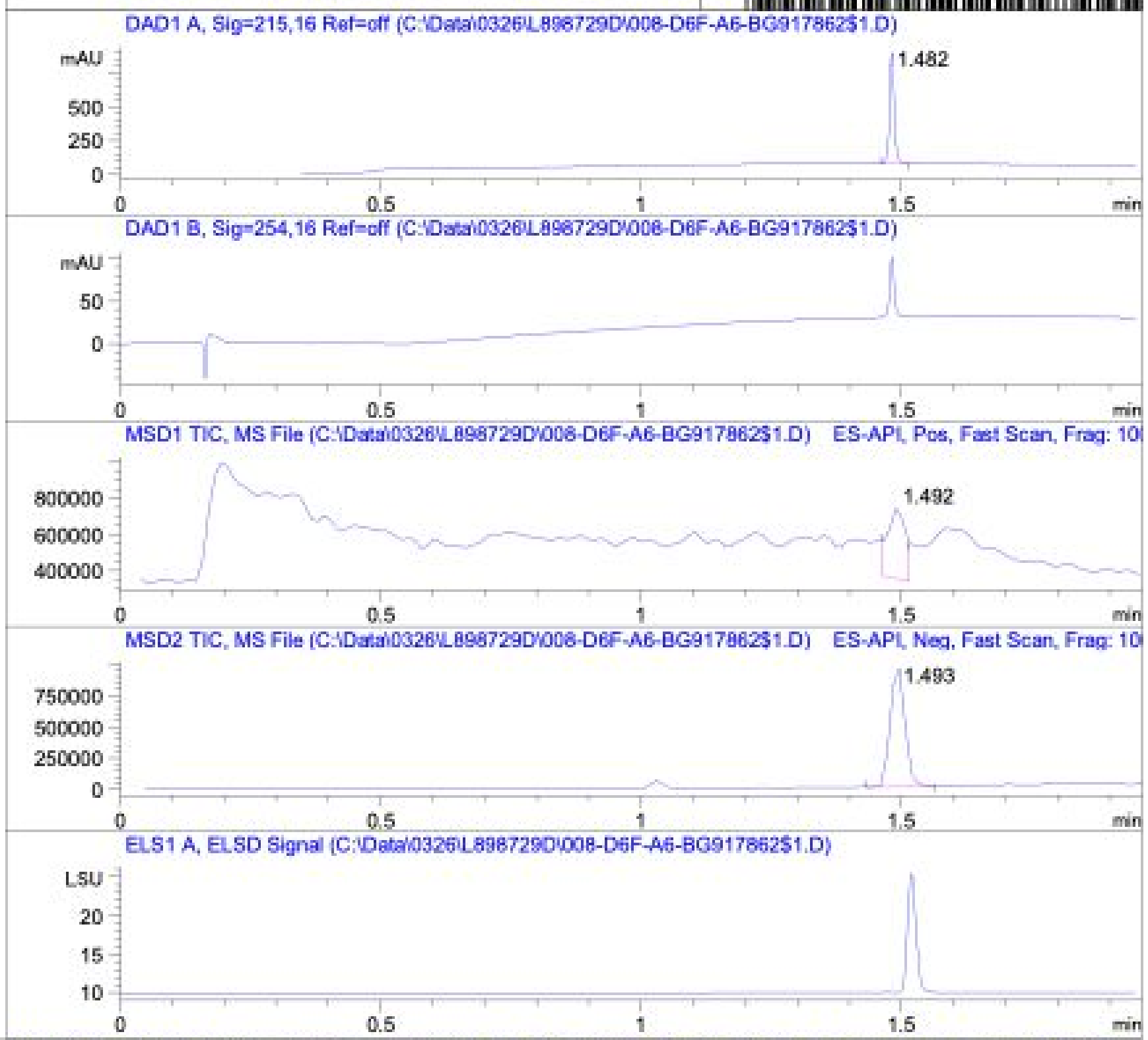

RT 1.492

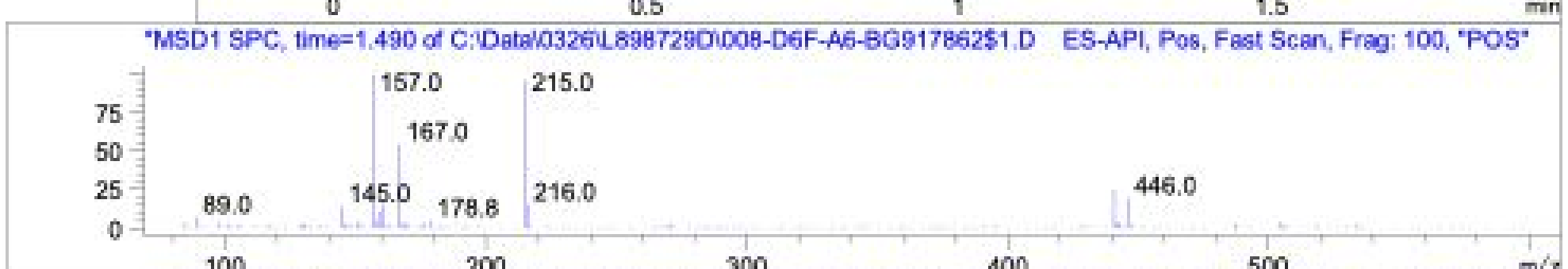

RT 1.493

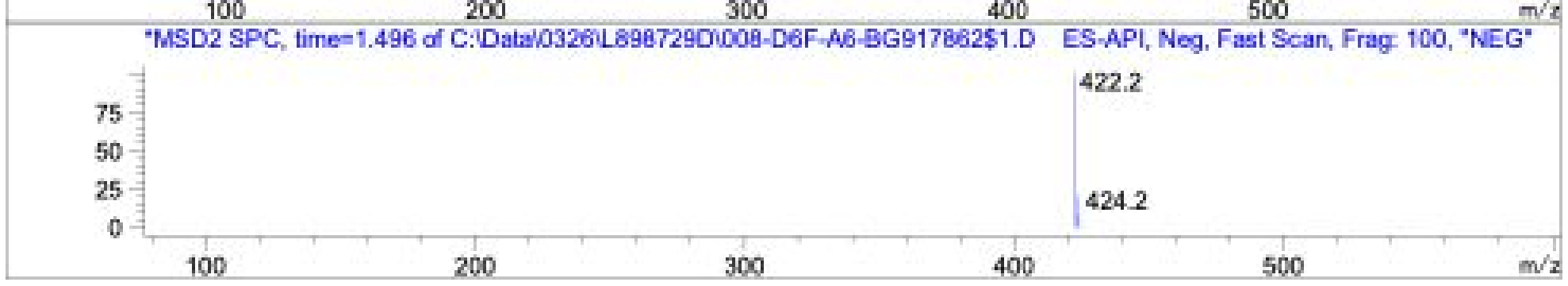

Inj.Date 3/25/2025 NEW
C:\Users\Public\Documents\ChemStation\1\Data\03_25\L898729D\SUPOR_30.M

Z9544922369

MaxPeak: 97.78%
Ret_Time: 0.995 min

BG917865$3

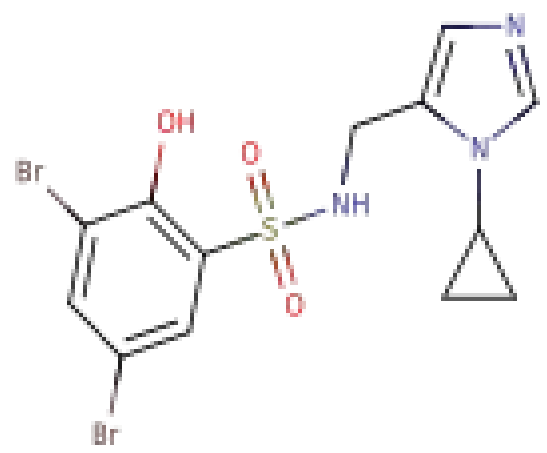

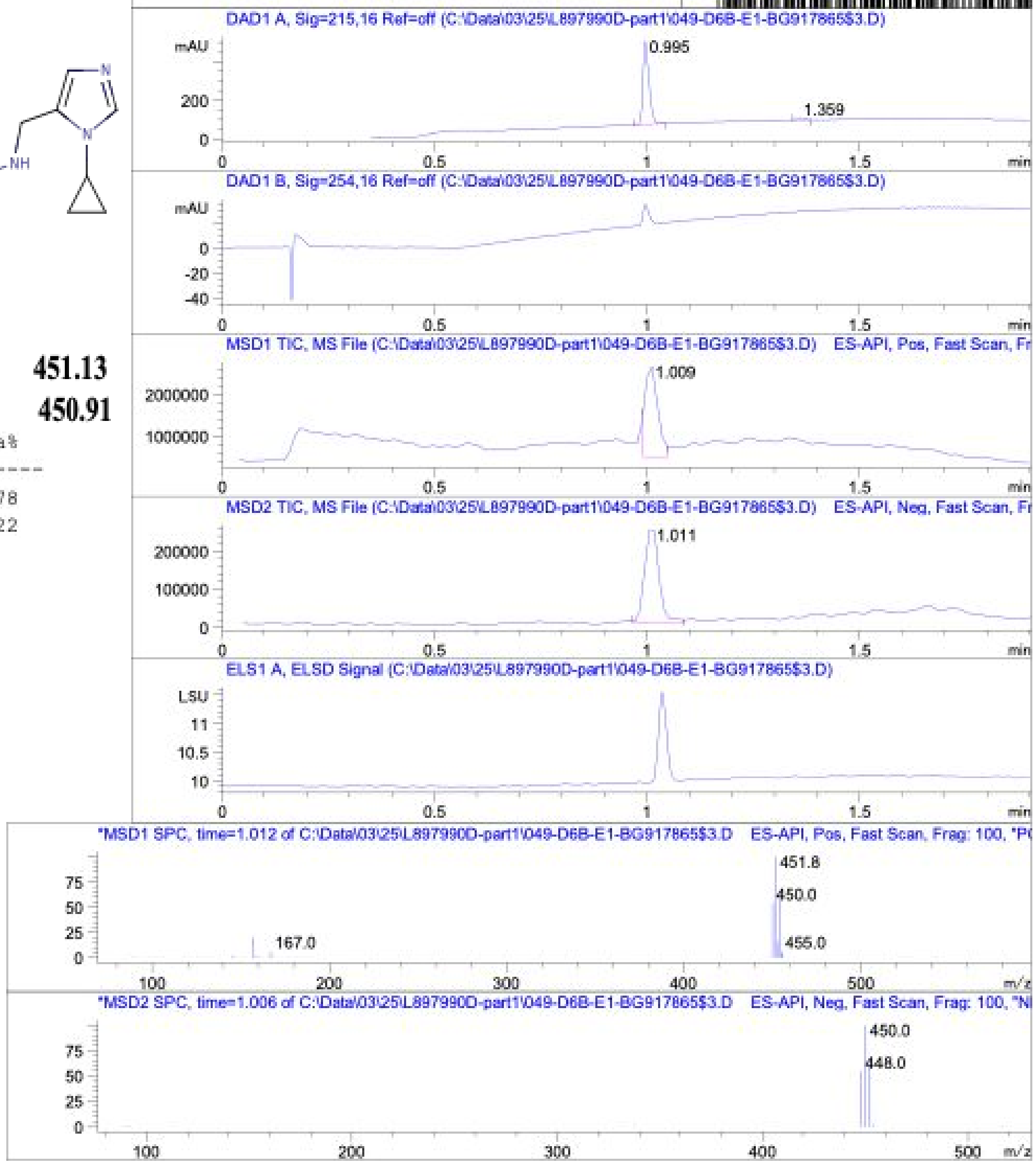

Mol Wt 451.13

Exact Mass 450.91

#	Time	Area%
1	0.995	97.78
2	1.359	2.22

RT 1.009

RT 1.011

Inj.Date 3/24/2025 C
C:\Users\Public\Documents\ChemStation\1\Data\03_24\1897990D\SUPOR_30.M

Z9544922376

MaxPeak: 98.21%
Ret_Time: 1.543 min

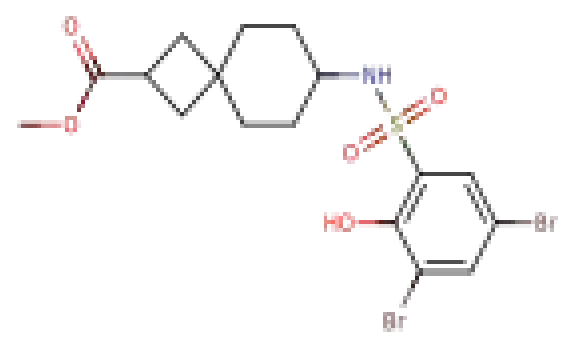

Mol Wt 511.23
Exact Mass 510.97

#	Time	Area%
1	1.512	1.79
2	1.543	98.21

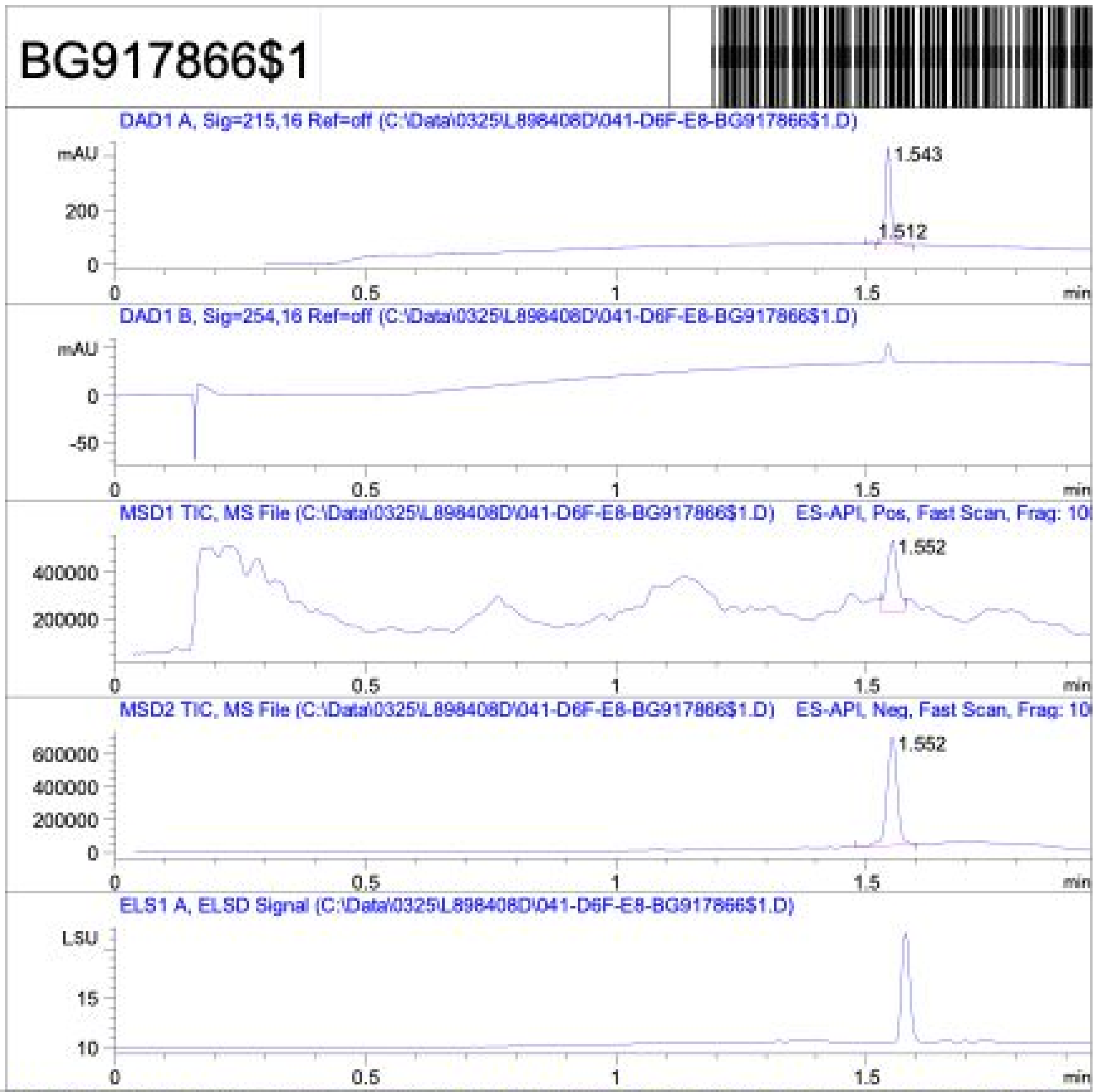

RT 1.552

RT 1.552

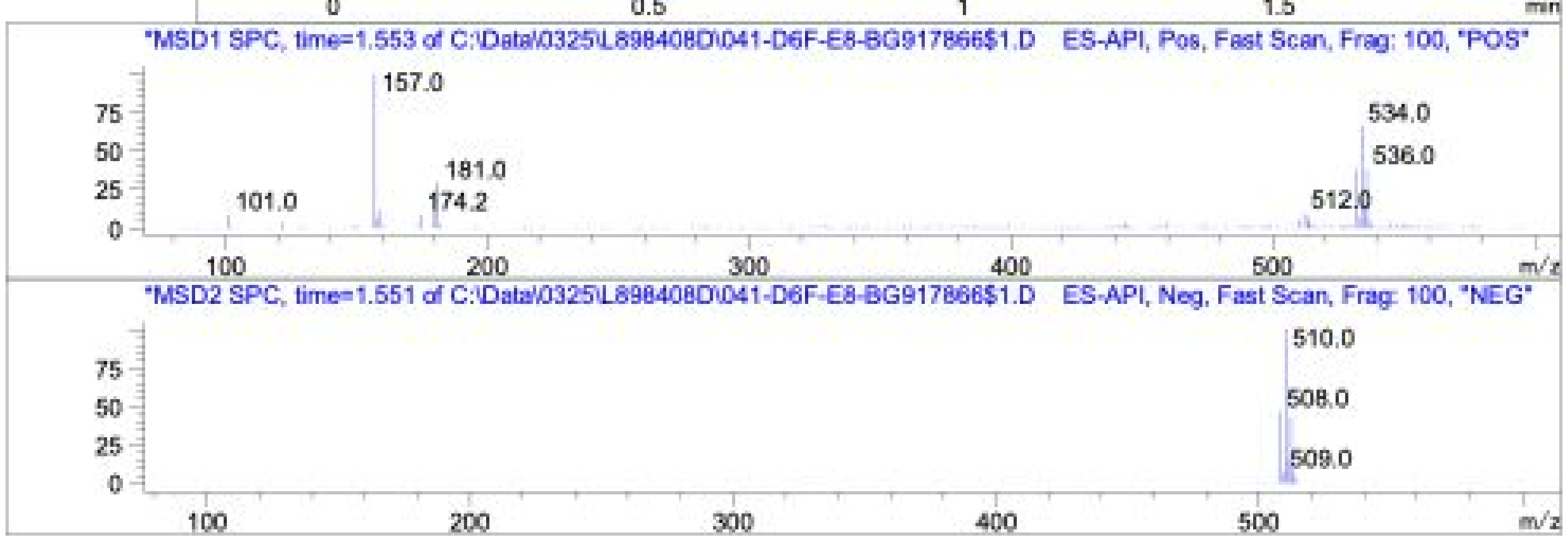

Inj.Date 3/24/2025 NEW -29-
C:\Users\Public\Documents\ChemStation\1\Data\03_24\L898408D\SUPOR_30.M

Z9544922381

MaxPeak: 100.00%
Ret_Time: 1.582 min

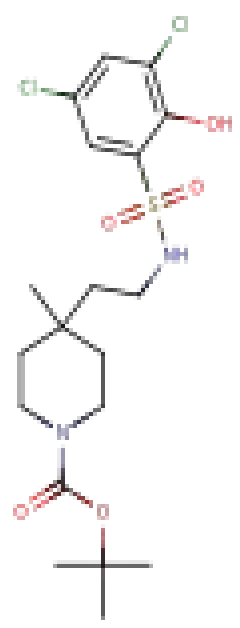

Mol Wt 467.41

Exact Mass 466.14

#	Time	Area%
1	1.582	100.00

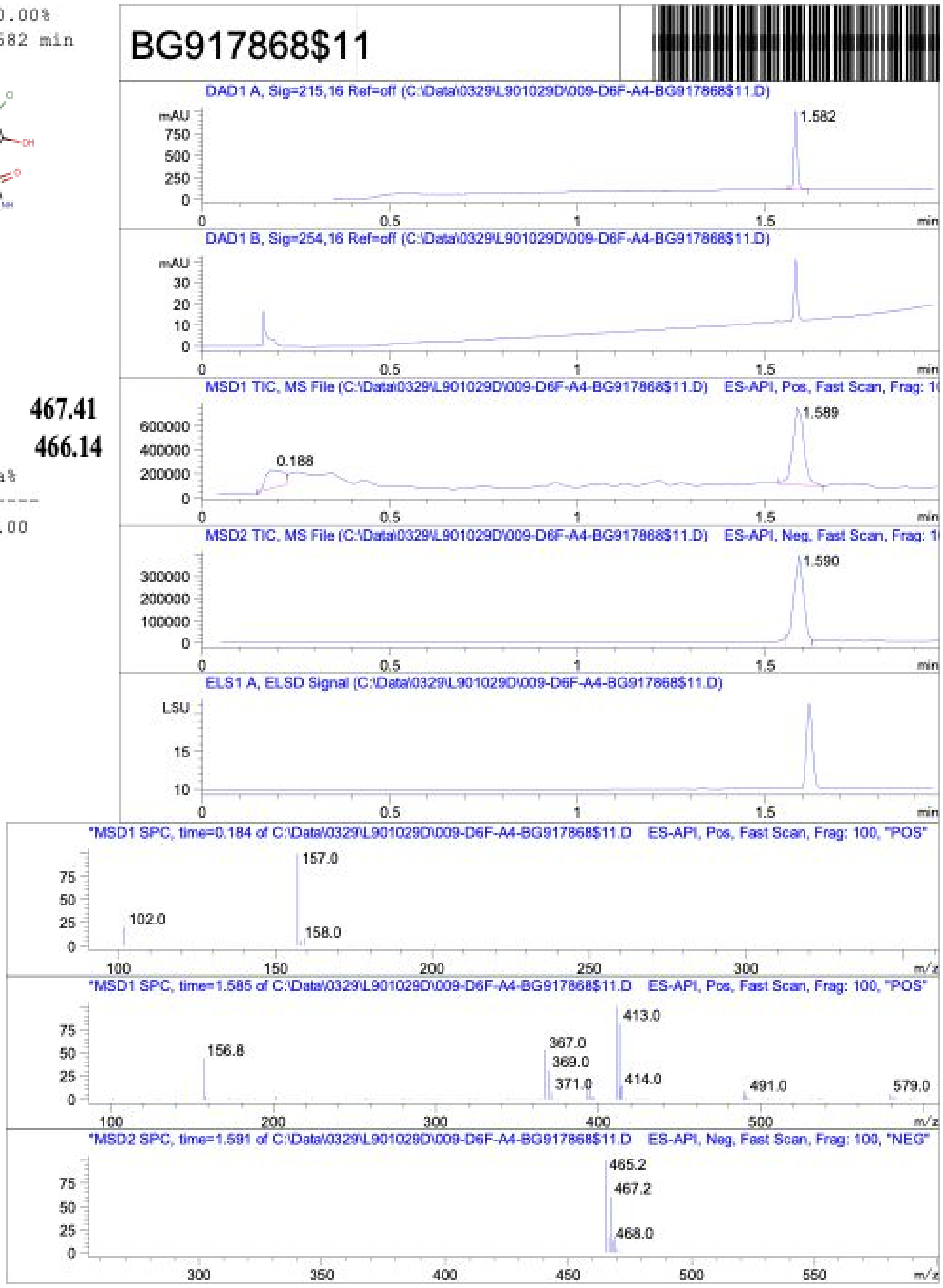

Inj.Date 3/28/2025 NEW -34-
C:\Users\Public\Documents\ChemStation\1\Data\03_28\L901029D\SUPOR_30.M

Z9544922391

MaxPeak: 100.00%
Ret_Time: 1.245 min

BG917876$115

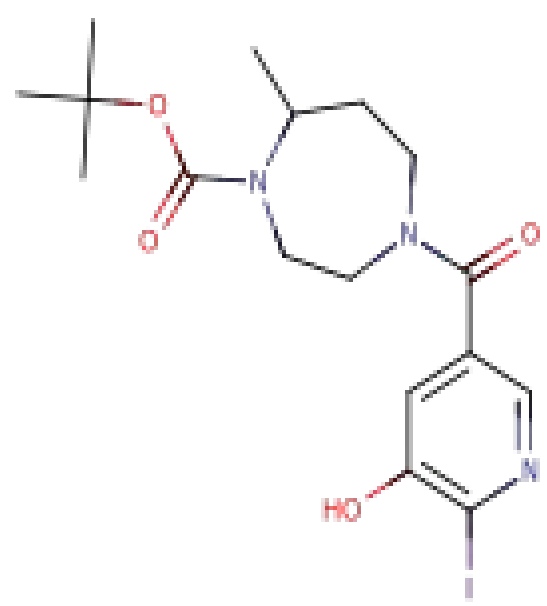

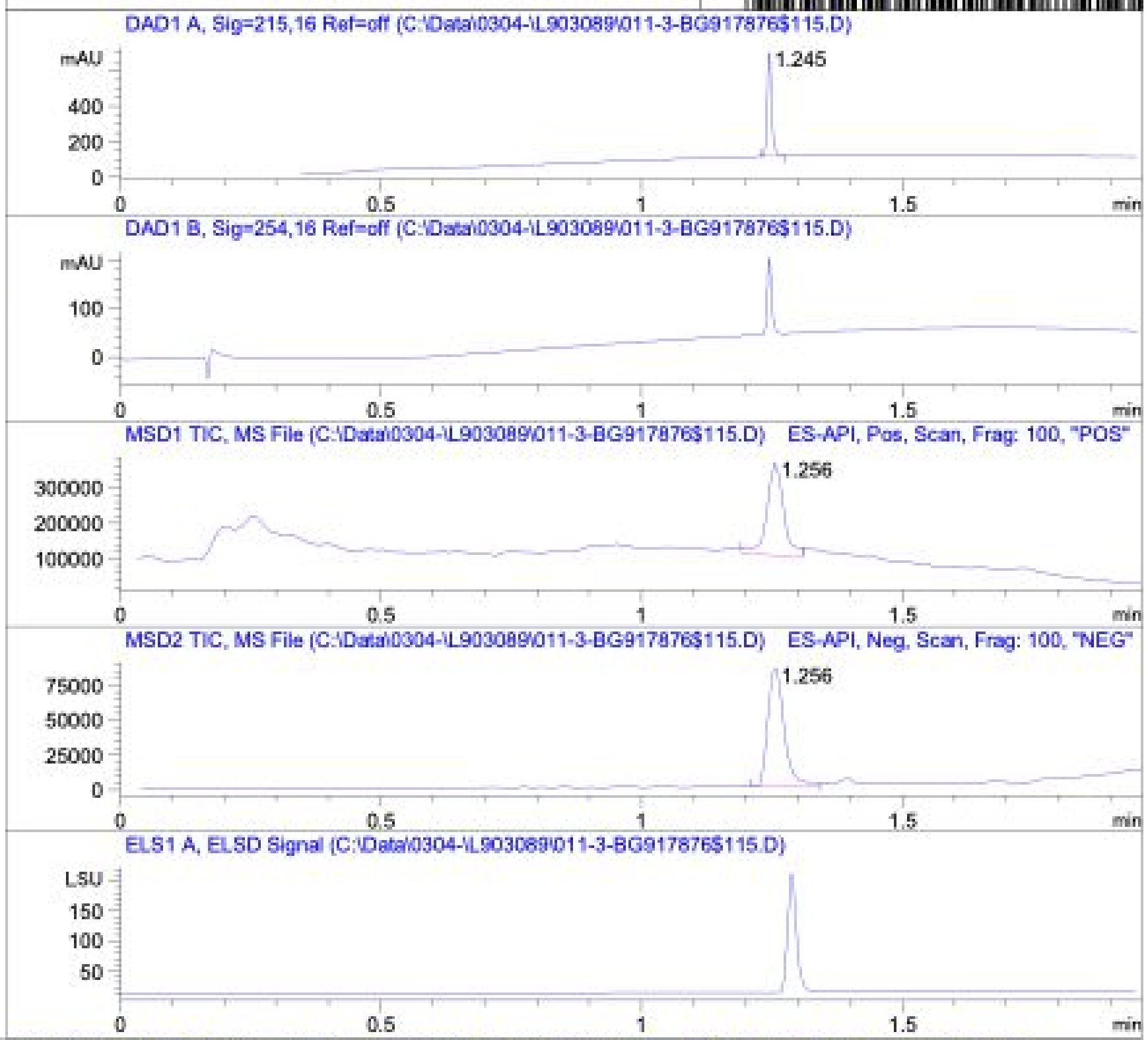

Mol Wt 461.3

Exact Mass 461.1

#	Time	Area%
1	1.245	100.00

RT 1.256

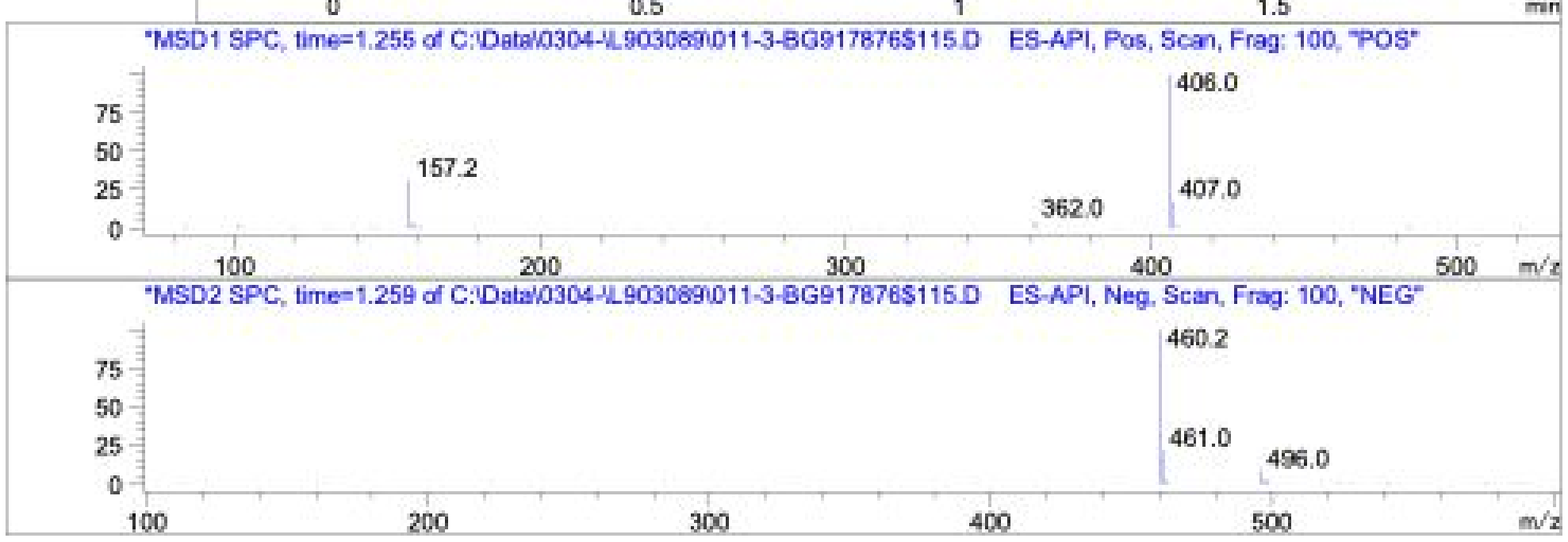

RT 1.256

Inj.Date 4/3/2025 NEW 13
C:\Users\Public\Documents\ChemStation\1\Data\04_03\L903089\MANUAL-SUPOR_30.M

Z9544922431

MaxPeak: 100.00%
Ret_Time: 1.490 min

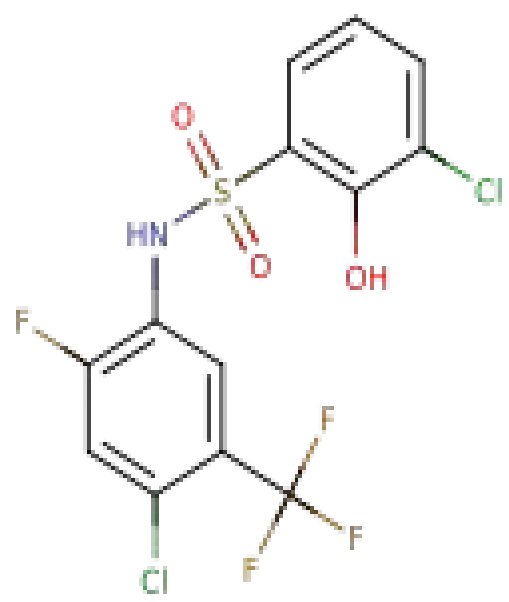

Mol Wt 404.16

Exact Mass 402.95

#	Time	Area%
1	1.490	100.00

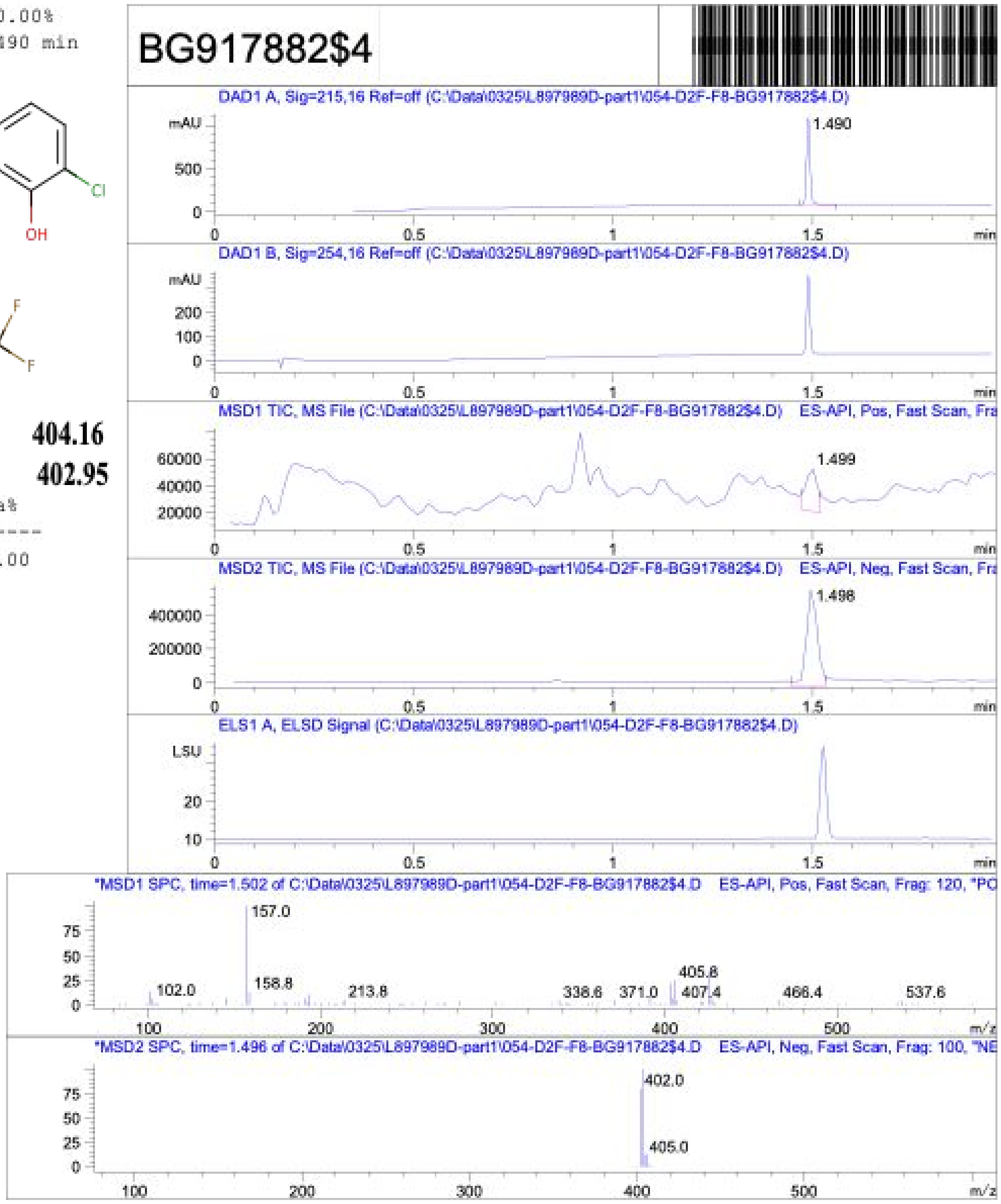

RT 1.499

RT 1.498

Inj.Date 3/24/2025 NEW -21-
C:\Users\Public\Documents\ChemStation\1\Data\03_24\1897989D\SUPOR_30.M

Z9544922461

MaxPeak: 100.00%
Ret_Time: 1.328 min

BG917884$32

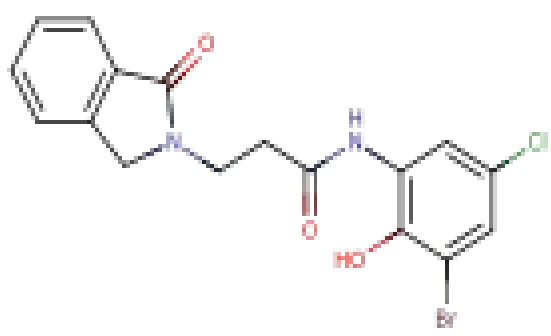

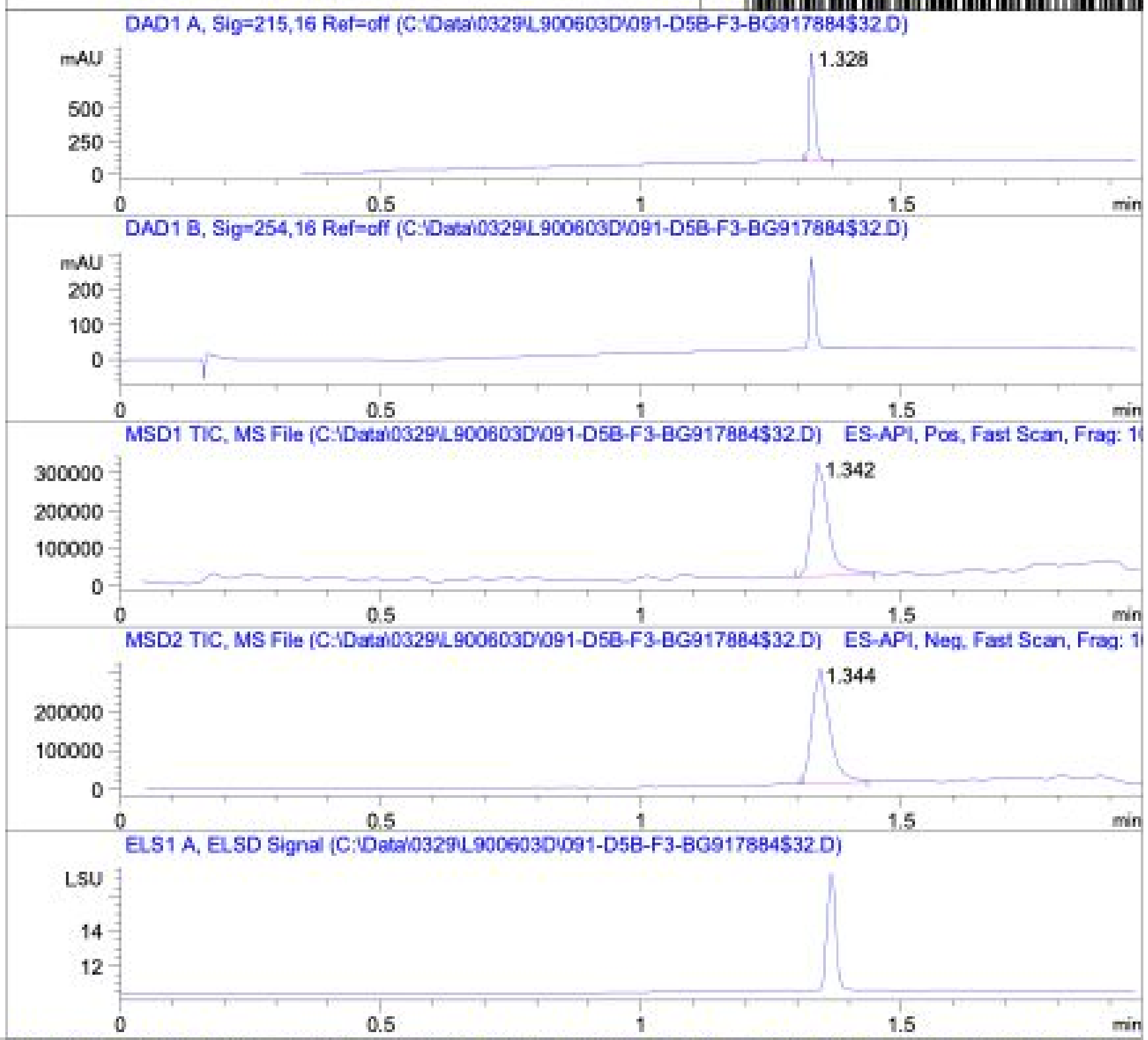

Mol Wt 409.66

Exact Mass 410

#	Time	Area%
1	1.328	100.00

RT 1.342

RT 1.344

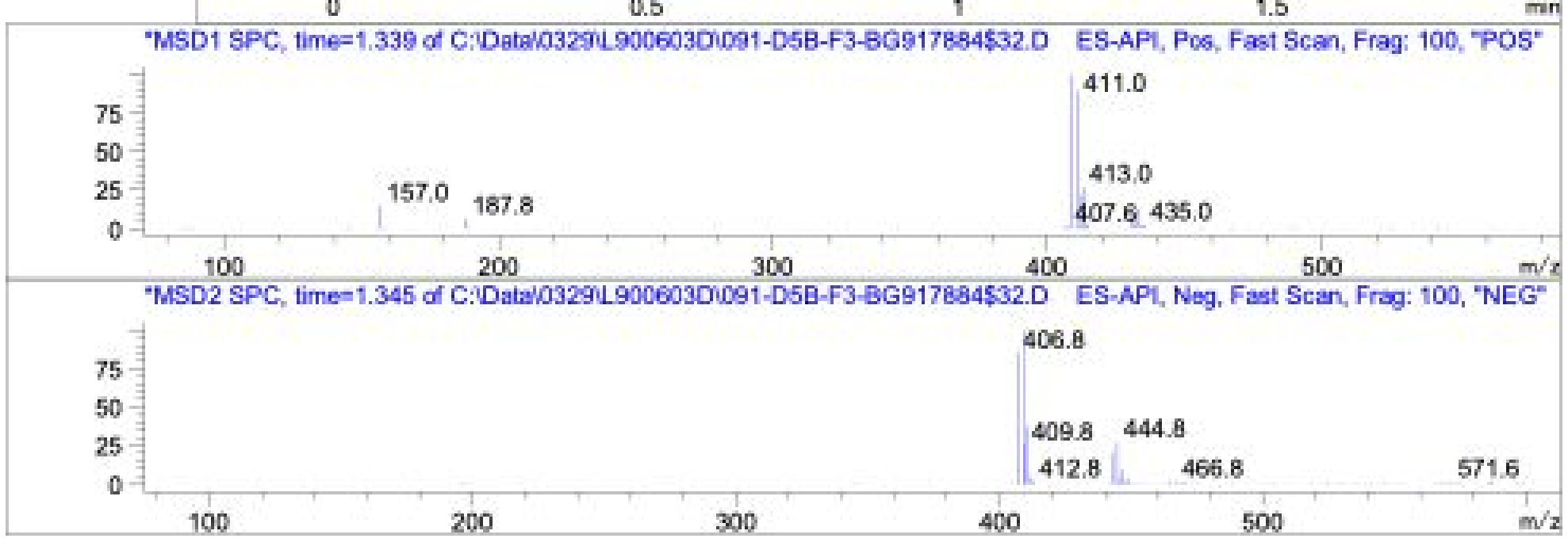

Inj.Date 3/28/2025 NEW - 23 -
C:\Users\Public\Documents\ChemStation\1\Data\03_28\L900603D\SUPOR_30.M

Z9544922472

MaxPeak: 100.00%
Ret_Time: 1.270 min

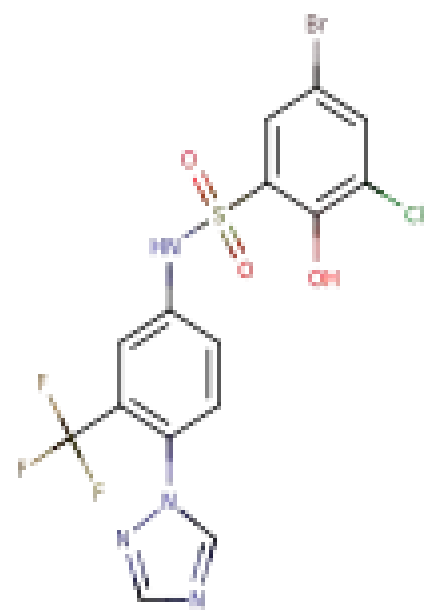

Mol Wt 497.68

Exact Mass 497.92

#	Time	Area%
1	1.270	100.00

BG917885$2

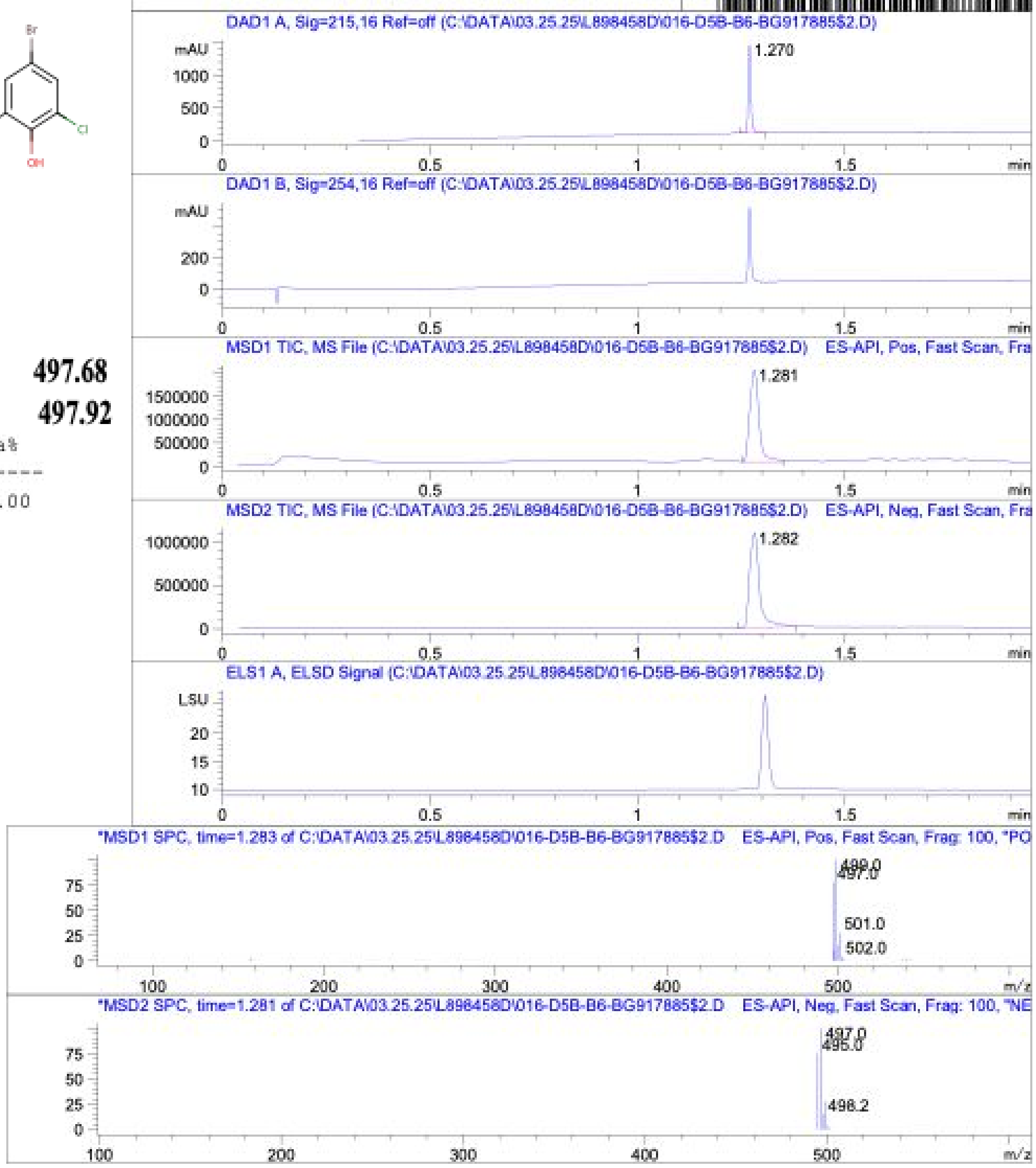

RT 1.281

RT 1.282

Inj.Date 3/24/2025 NEW
C:\Users\Public\Documents\ChemStation\1\Data\03_24\L898458D\SUPOR_30.M

Z9544922478

MaxPeak: 100.00%
Ret_Time: 1.199 min

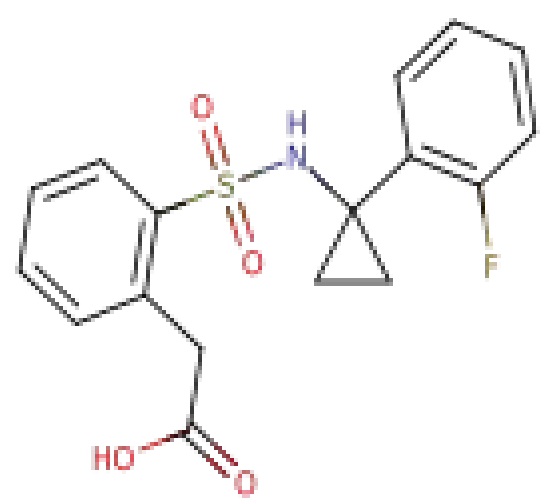

Mol Wt 349.38
Exact Mass 349.09

#	Time	Area%
1	1.199	100.00

BG917944$1

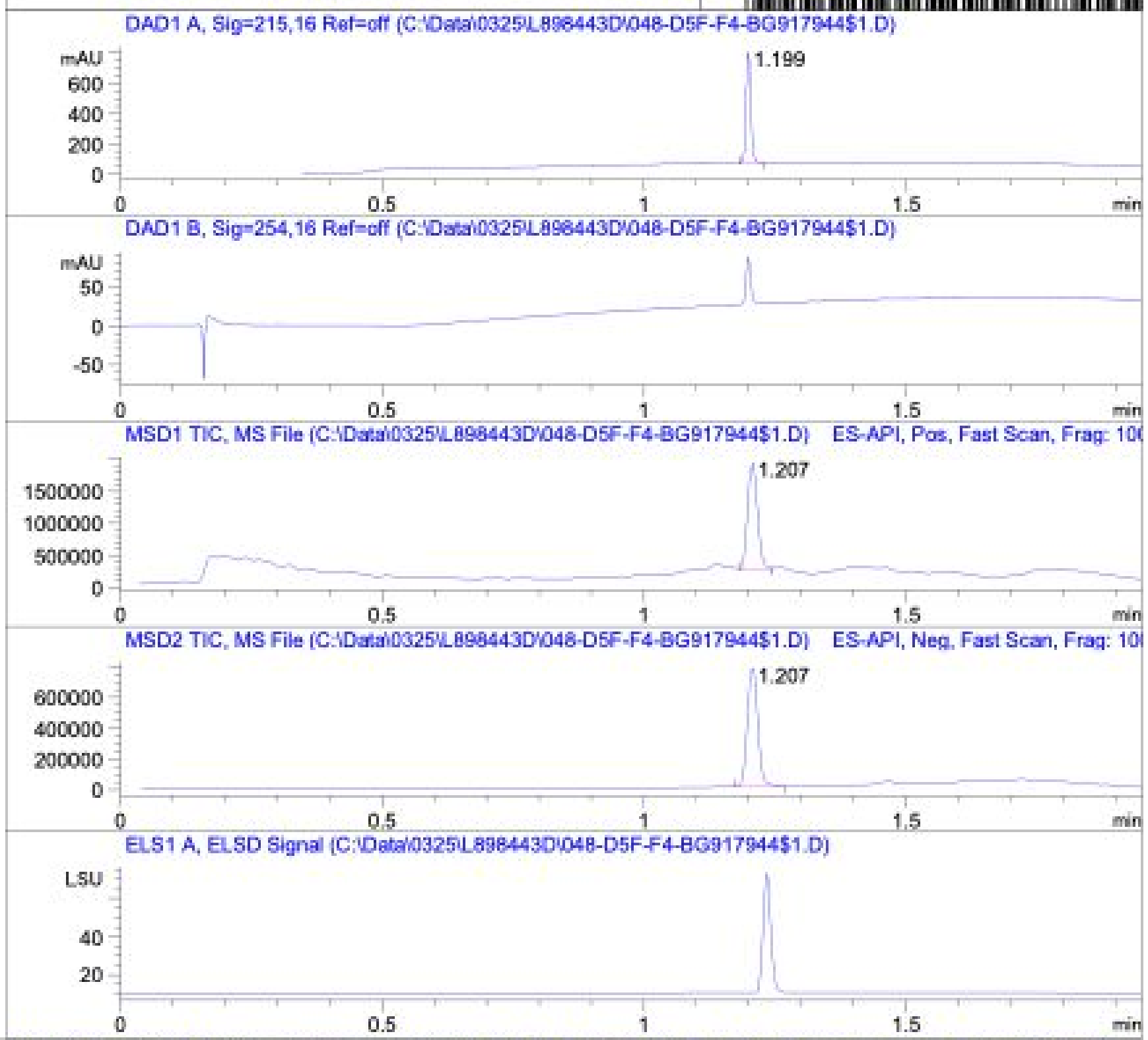

RT 1.207

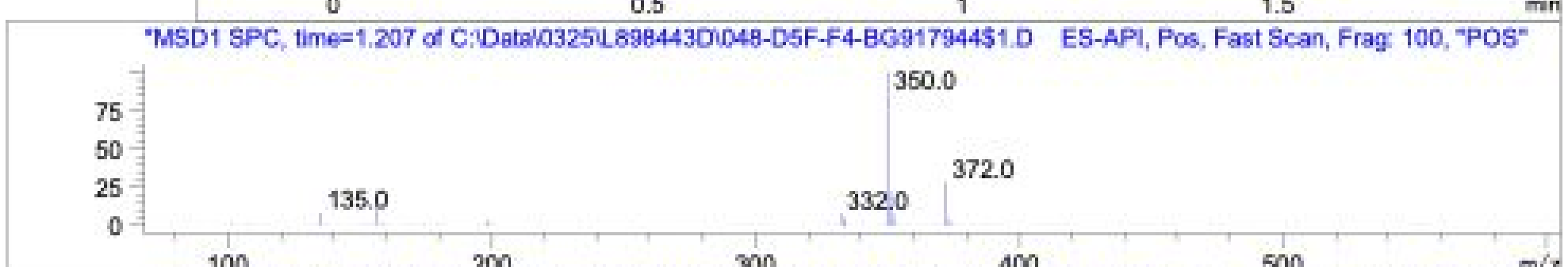

RT 1.207

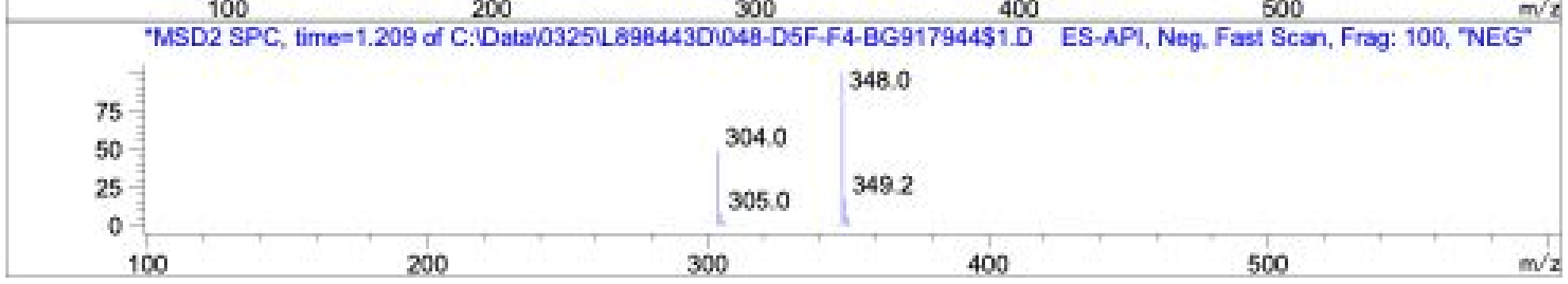

Inj.Date 3/25/2025 KCH -29-
C:\Users\Public\Documents\ChemStation\1\Data\03_24\L898443D\SUPOR_30.M

Z9544922551

MaxPeak: 98.22%
Ret_Time: 1.244 min

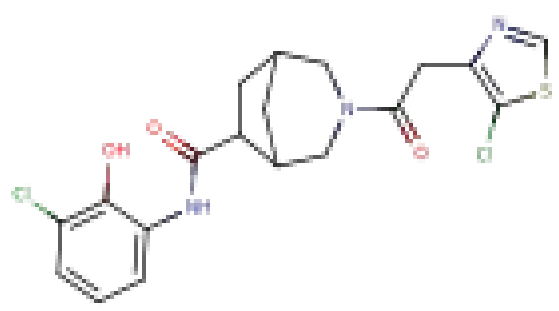

Mol Wt 440.34
Exact Mass 439.07

#	Time	Area%
1	1.179	1.78
2	1.244	98.22

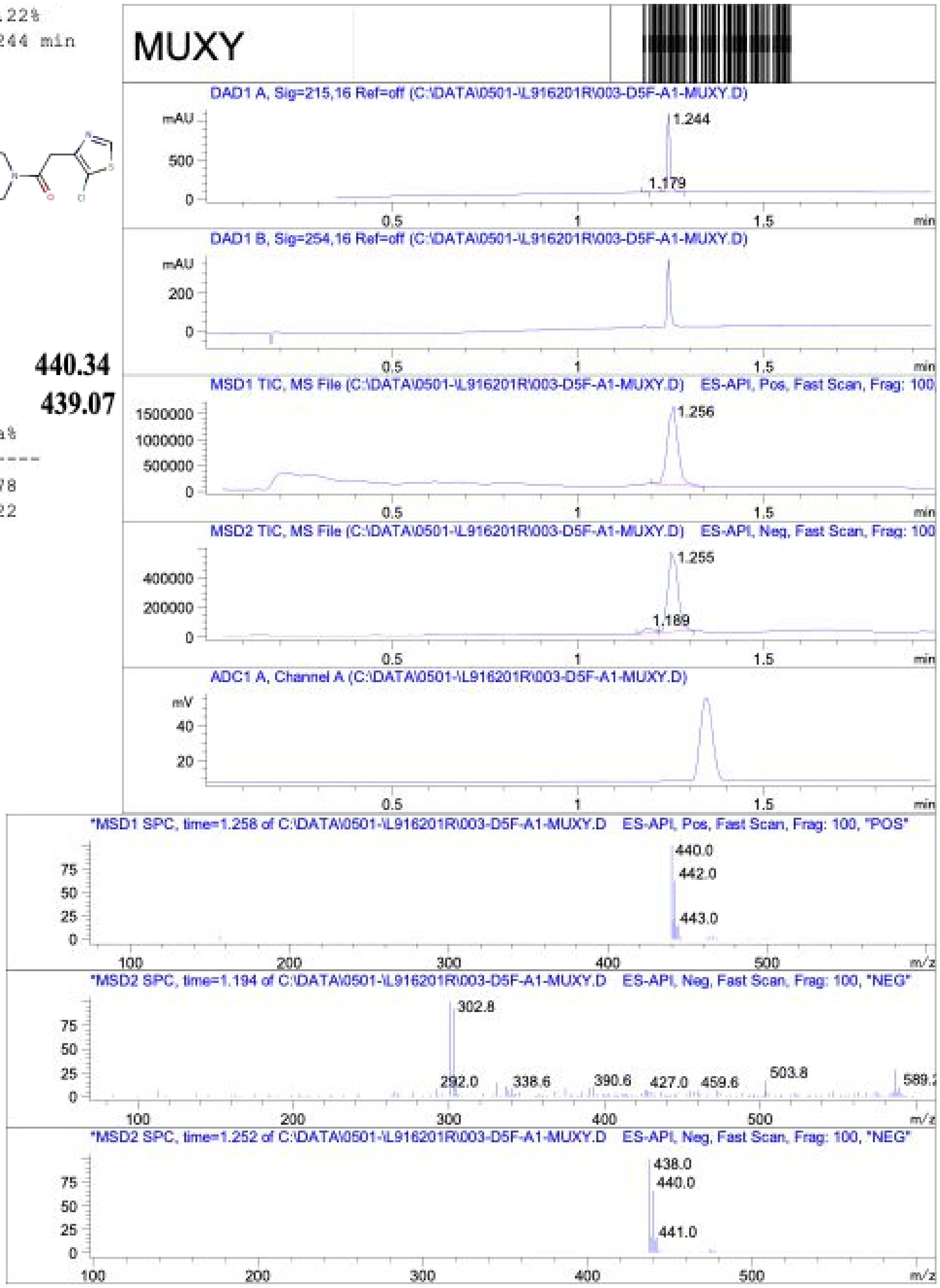

RT 1.256

RT 1.189

RT 1.255

Inj.Date 5/1/2025 NEW -3-
C:\Users\Public\Documents\ChemStation\1\Data\05_01\L916201R\SUPOR_30.M

Z9544957371

MaxPeak: 100.00%
Ret_Time: 1.414 min

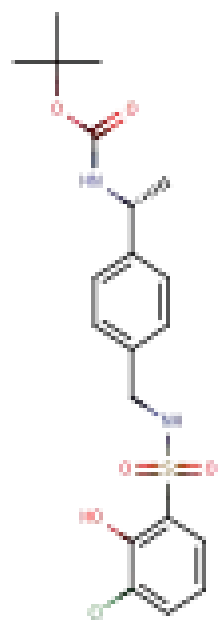

Mol Wt 440.94

Exact Mass 440.14

#	Time	Area%
1	1.414	100.00

BH091708$41

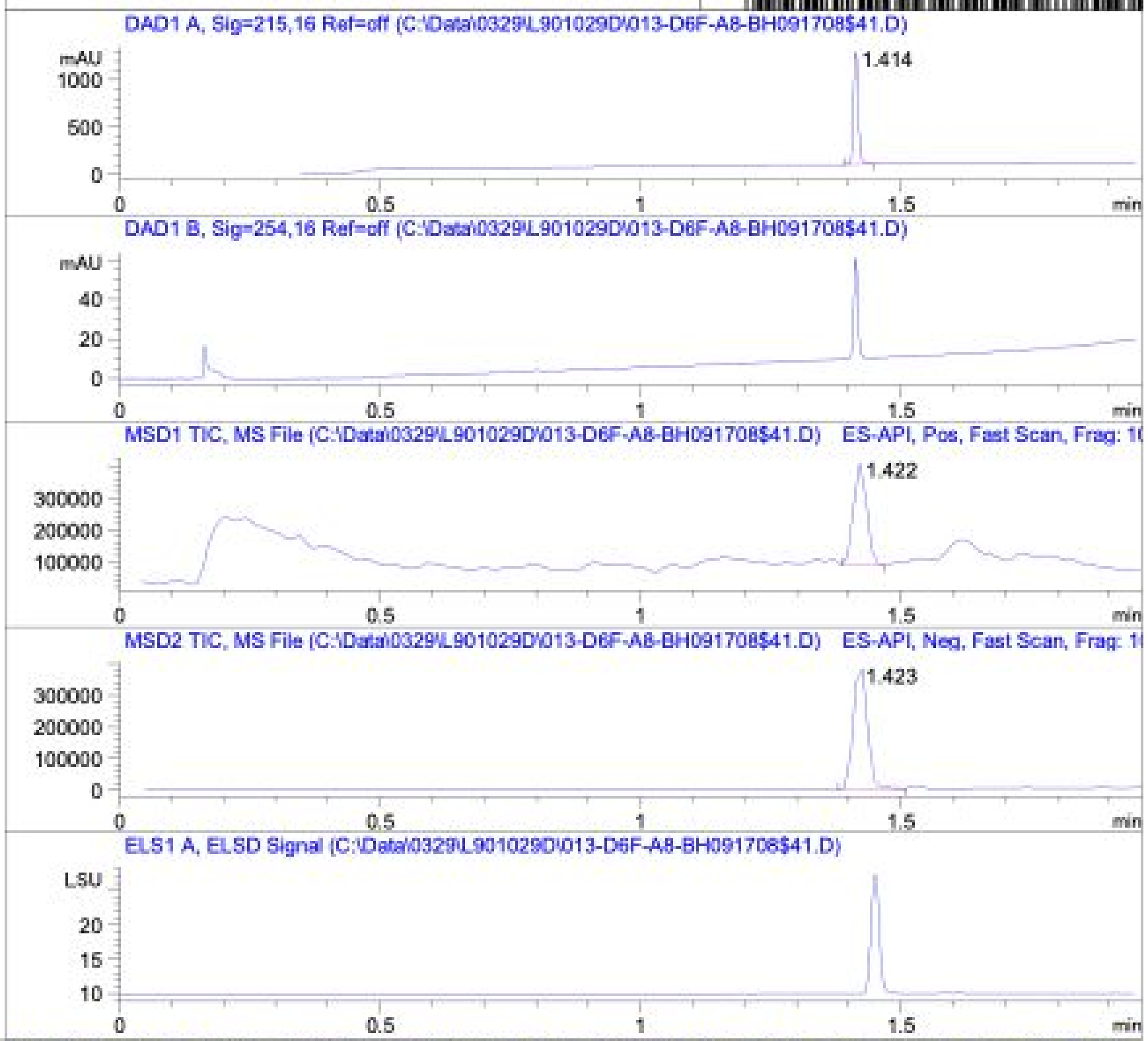

RT 1.422

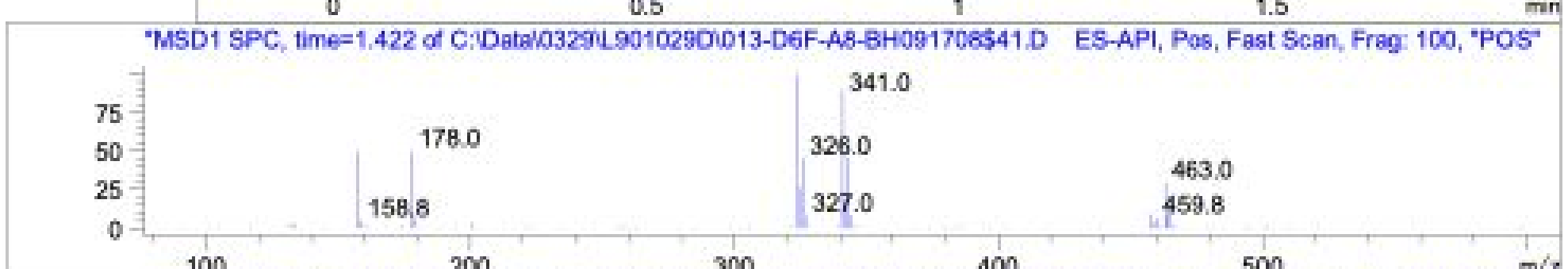

RT 1.423

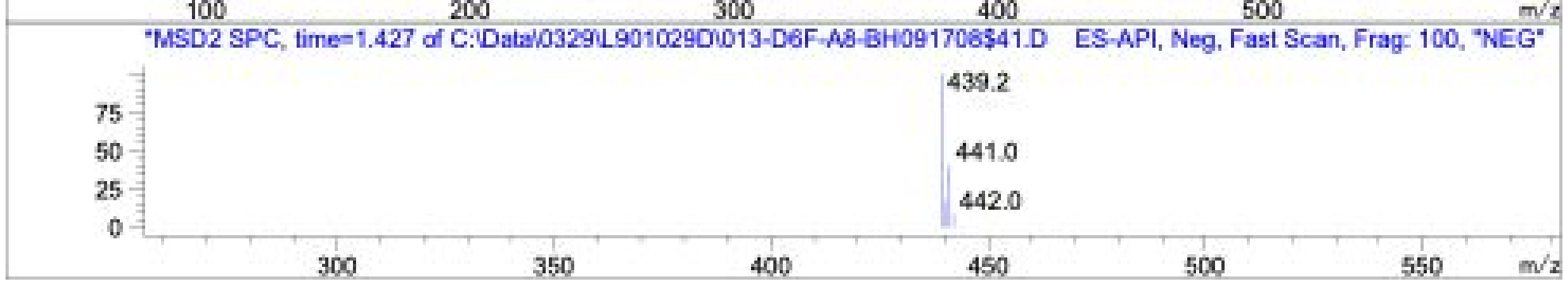

Inj.Date 3/28/2025 NEW -34-
C:\Users\Public\Documents\ChemStation\1\Data\03_28\L901029D\SUPOR_30.M

Z9544960567
